# Zirconium and hafnium catalyzed C–C single bond hydroboration

**DOI:** 10.1038/s41467-024-45697-y

**Published:** 2024-02-28

**Authors:** Sida Li, Haijun Jiao, Xing-Zhong Shu, Lipeng Wu

**Affiliations:** 1 State Key Laboratory for Oxo Synthesis and Selective Oxidation, Lanzhou Institute of Chemical Physics (LICP), Chinese Academy of Sciences, Lanzhou, 730000 PR China; 2 University of Chinese Academy of Sciences, Beijing, 100049 PR China; 3 Leibniz-Institut für Katalyse e. V., Albert-Einstein-Straße 29a, 18059 Rostock, Germany; 4 State Key Laboratory of Applied Organic Chemistry, College of Chemistry and Chemical Engineering, Lanzhou University, Lanzhou, 730000 PR China; 5 College of Material Chemistry and Chemical Engineering, Key Laboratory of Organosilicon Chemistry and Material Technology, Ministry of Education, Hangzhou Normal University, Hangzhou, 311121 PR China

**Keywords:** Homogeneous catalysis, Synthetic chemistry methodology, Catalyst synthesis

## Abstract

Selective cleavage and subsequent functionalization of C−C single bonds present a fundamental challenge in synthetic organic chemistry. Traditionally, the activation of C−C single bonds has been achieved using stoichiometric transition-metal complexes. Recently, examples of catalytic processes were developed in which use is made of precious metals. However, the use of inexpensive and Earth-abundant group IV metals for catalytic C−C single-bond cleavage is largely underdeveloped. Herein, the zirconium-catalyzed C−C single-bond cleavage and subsequent hydroboration reactions is realized using Cp_2_ZrCl_2_ as a catalytic system. A series of structures of various γ-boronated amines are readily obtained, which are otherwise difficult to obtain. Mechanistic studies disclose the formation of a N–Zr^IV^ species, and then a β-carbon elimination route is responsible for C–C single bond activation. Besides zirconium, hafnium exhibits a similar performance for this transformation.

## Abstract (web summary)

Selective cleavage and subsequent functionalization of C−C single bonds present a fundamental challenge, and the use of inexpensive and Earth-abundant group IV metals for catalytic C−C single-bond cleavage is largely underdeveloped. Here, the authors report zirconium-catalyzed C−C single-bond cleavage and subsequent hydroboration reactions.

## Introduction

**Fig. 1:**
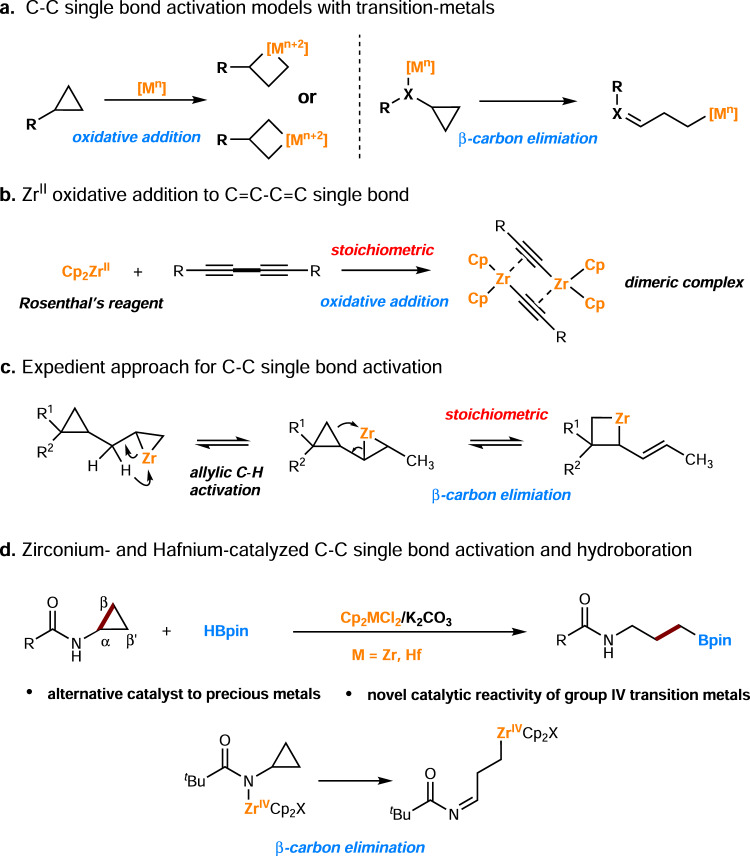
Strategies for C−C single bond activation. **a** Transition metal mediated C−C single bond activation; **b**, **c** stoichiometric amount of zirconium-mediated C−C single bond activation; **d** zirconium and hafnium catalyzed C−C single bond activation.

Selective cleavage and subsequent functionalization of C−C single bonds present a fundamental challenge in catalysis and synthesis^1–11^. This is mainly due to the relatively high bonding energy (BE, about 355 kJ/mol) and directional σ orbitals of C−C bonds. In addition, the competitive C−H bond activation (about 400 kJ/mol, but statistically abundant) also causes chemoselectivity problems^12–22^. Nevertheless, cleavage and functionalization of C−C single bonds are attracting increasing attention in synthetic organic chemistry because it offers a unique and straight route to target molecules/structures. Synthetic chemists have developed various strategies for the activation of C−C single bonds. They are mainly classified into two mechanistic categories: oxidative addition and β-carbon elimination, associated with metal centers (Fig. 1a). Besides the use of stoichiometric transition-metal complexes^23–26^, examples of catalytic processes have been reported in recent years—most involve the use of precious metals^27–45^.

Early-transition metals have different electron configurations from late ones. Thus, their complexes often show other or orthogonal reactivities with late-transition metal complexes^46–61^. In addition, they are also Earth-abundant (e.g., zirconium is almost as abundant as carbon in the Earth’s upper continental crust). However, zirconium usually exists in the Zr^IV^ oxidation state, which is not viable for direct oxidative addition activation of a single chemical bond. Thus, in situ generated or isolable low-valent zirconocene complexes (Zr^II^), such as Negishi’s (Cp_2_ZrBu_2_)^62^ and Rosenthal’s reagents (Cp_2_Zr(py)Me_3_SiC≡CSiMe_3_)^63^, for the activation of B−H^64,65^, Si−H^66–69^ bonds were reported. C−C single bond cleavage by a zirconium species has also been reported intermittently since the 1990s^70–73^. In 1994, Rosenthal described the activation of conjugated C−C single bonds of a 1,3-butadiyne moiety (C≡C−C≡C) using Rosenthal’s reagent, resulting in a dimeric complex (Fig. 1b)^74^. Dimmock and Whitby also found that zirconocene η^2^-alkene and η^2^-imine complexes with adjacent cyclopropane rings could undergo cyclopropane ring cleavage^75^. Then, in 2014, Marek reported an expedient approach, including allylic C−H activations followed by C−C single bond activation (Fig. 1c)^76^. It is also worth mentioning that since the 1990s, Negishi, Takahashi, and Xi have studied the chemistry of zirconacycles, the transformation of which with other unsaturated molecules usually involved a β, β’-C−C bond cleavage^77–81^. All the former instances used (over) stoichiometric amounts of zirconium, and no precedents of catalytic methods using homogeneous zirconium catalysis had been developed—to the best of our knowledge (The use of heterogeneous zirconium catalysis for C−C bond cleavage was reported by Basset^82–84^). Consequently, activating C−C single bonds with zirconium catalysis for chemical transformation remains a significant challenge. It is of considerable scientific and practical interest to synthetic organic chemistry to address this. Herein, we report the development of an unprecedented catalytic system that resulted in the realization of the zirconium- and hafnium-catalyzed C−C single-bond hydroboration (Fig. 1d). Mechanistic studies support the formation of N−Zr^IV^ species and then a β-carbon elimination route for C−C single bond activation. Our work provides an alternative catalytic method for C−C single bonds hydroboration, and establishes the bond activation models and catalytic application of group IV transition metals.

## Results

### Catalytic reaction investigations

As a synthetically significant transformation in organic chemistry, hydroboration of C=C bonds is well-studied. However, catalytic hydroboration of C−C single bonds remains underdeveloped. Only two systems using Ir and Rh are known for the hydroboration of cyclopropanes, as developed by Yamaguchi^85^ and Shi^86,87^. Besides making use of precious metals and *N*- or *P*-ligands, it is noticed that for the Ir system, the careful choice of a chiral *t*Bu-Quinox ligand is crucial for the C−C bond hydroboration over the C−H boration^88–90^. For the Rh system, the PPh_3_ ligand is essential in inhibiting side reactions such as the formation of alkenes. Furthermore, in the former case, cleavage of C_β_−C_β‘_ bond is observed, while the latter cleavages C_α_−C_β_ bond. Thus, it is still highly desirable to develop a facile and inexpensive catalytic system for the hydroboration of C−C single bonds.

We commenced our investigation using 0.2 mmol of *N*-Piv-cyclopropylamines (**1a**) with 1.5 equiv. pinacolborane (HBpin) in 1 mL toluene at 120 °C as the model reaction, using 5 mol% Cp_2_ZrCl_2_ as catalyst (Table 1). Our preliminary investigations unveiled that the addition of 1 equiv. of base is the key for the zirconium-catalyzed C−C bond hydroboration (Supplementary Table 1); K_2_CO_3_ was the optimal choice (Table 1, entry 1, 81% yield of **2a**). Various other zirconium complexes were then tested but were unsuccessful. There was no reaction with the sterically bulkier Cp*_2_ZrCl_2_ (Table 1, entry 2). With Cp_2_ZrHCl as catalyst, a 50% yield of **2a** was obtained, whereas with Cp_2_ZrMe_2_ only 10% **2a** was produced (Table 1, entries 3 and 4). It was evident that without Cp_2_ZrCl_2_ or K_2_CO_3_ no reaction proceeded (Table 1, entries 5 and 6). Interestingly, using Cp_2_TiCl_2_ instead of Cp_2_ZrCl_2_ also gave no reaction (Table 1, entry 7). An attempt was then made to reduce the amount of K_2_CO_3_. Pleasingly, even with 0.1 equiv. K_2_CO_3_ already had a 68% yield of **2a** (Table 1, entry 8). Finally, a much higher yield of **2a** (91%) was obtained with just 0.3 equiv. K_2_CO_3_ (Table 1, entry 9). In addition, 95% yield of **2a** was obtained using 5 mol% Cp_2_ZrH_2_ as catalyst without K_2_CO_3_ (Table 1, entry 10)_._ However, considering the simplicity of using readily available and inexpensive Cp_2_ZrCl_2_ as the catalyst, we conducted the following studies using Cp_2_ZrCl_2_/K_2_CO_3_ system (Table 1, entry 9). The results with Cp_2_ZrH_2_ gave us some clues for the subsequent mechanism studies (vide infra).

**Table 1:** Zr-catalyzed hydroboration of cyclopropylamines—condition optimization^a^


Entry	Catal.	x	2a yield (%)^b^
1	Cp_2_ZrCl_2_	1	81
2	Cp*_2_ZrCl_2_	1	-
3	Cp_2_ZrHCl	1	50
4	Cp_2_ZrMe_2_	1	10
5	Cp_2_ZrCl_2_	0	-
6	-	1	-
7	Cp_2_TiCl_2_	1	-
8	Cp_2_ZrCl_2_	0.1	68
**9**	**Cp**_**2**_**ZrCl**_**2**_	**0.3**	**91**
10	Cp_2_ZrH_2_	0	95

^a^Reaction conditions: 0.2 mmol **1a**, HBpin (1.5 equiv.), catalyst (5 mol%), K_2_CO_3_ (0.1−1 equiv.), and 1 mL toluene in a 15 mL pressure tube at 120 °C for 24 h.

^b^Yields were determined by GC using dodecane as an internal standard.

### Substrates scope studies

**Fig. 2:**
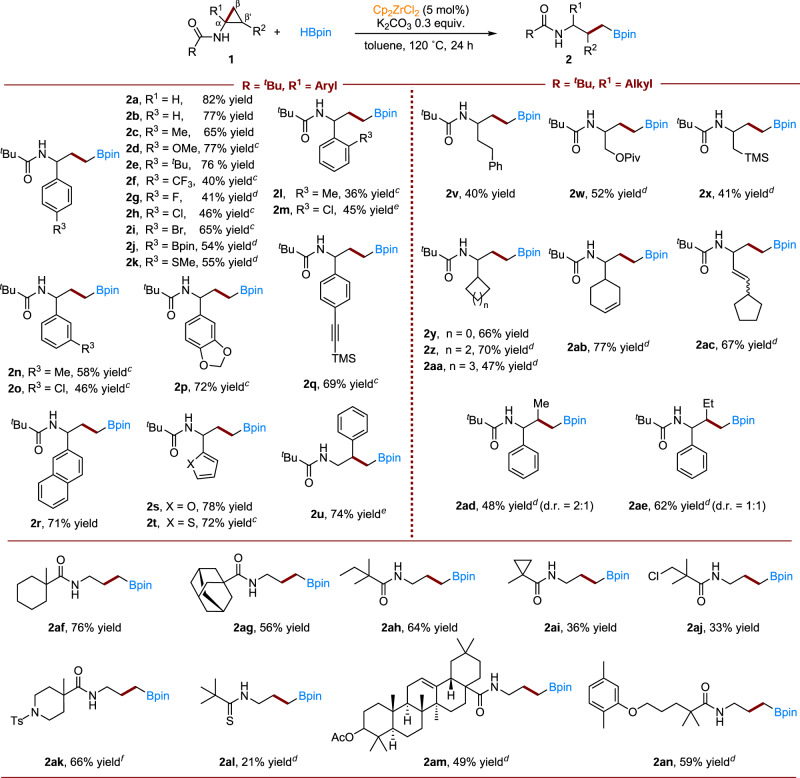
Zirconium-catalyzed hydroboration of cyclopropylamines—generality studies. ^*a,ba*^Reaction conditions: 0.2 mmol **1**, HBpin (1.5 equiv.), K_2_CO_3_ (30 mol%), Cp_2_ZrCl_2_ (5 mol%) and 1 mL toluene in a 15 mL pressure tube at 120 °C for 24 h; ^*b*^Isolated yields are given; ^*c*^130 °C for 24 h; ^*d*^150 °C for 24 h; ^*e*^HBpin (2.0 equiv.), K_2_CO_3_ (60 mol%), Cp_2_ZrCl_2_ (10 mol%) at 150 °C for 24 h; ^*f*^HBpin (2.0 equiv.), Cp_2_ZrH_2_ (5 mol%) at 150 °C for 24 h.

Having the reaction conditions for the Zr-catalyzed hydroboration of cyclopropylamines in hand (Table 1, entry 9), various cyclopropane rings were investigated to establish the generality of our methodology (Fig. 2).

First, the tolerance of substituents on the *para*-position of the phenyl ring was studied. We found that electron-neutral, electron-donating, and electron-withdrawing groups are tolerated; moderate to good yields were obtained (**2a**-**2k**, 40-82% yields). In general, electron-donating groups (−Me, −OMe, −^*t*^Bu, −SMe, **2c**−**2e,** **2k**) gave better results than electron-withdrawing groups such as −CF_3_ (**2f**). Halide substituents −F, −Cl, −Br, which likely undergo competitive hydrodehalogenation or boration reactions, are untouched in our system (**2g**−**2i**). We found that steric effects have some influence on the results, as changing the substituents from the *para*-position to the *meta*- and *ortho*-position, led to slightly decreased yields or the need for higher reaction temperatures (**2l**−**2o**). Significantly, naphthyl, benzodioxole, alkyne, and heteroaromatic rings such as furyl and thiophene substituents are all compatible in our system, with yields in the range of 71 − 78% (**2p**−**2t**). Similarly, good results were obtained when the phenyl group is at the β’ position (**2u**). Changing R^1^ from an aryl to an alkyl group was also successful, with both acyclic and cyclic alkyl substituents (up to 77% yield, **2v**−**2ac**). Pleasingly, products **2q,** **2ab** and **2ac** were obtained in 69%, 77%, and 67% yields, respectively, with no double or triple bond interference. Moreover, cyclopropane rings with two substituents on the R^1^ and R^2^ positions were also suitable (**2ad**−**2ae**).

The effect of the substituent on the amide groups was then studied. When R is 1-methylcyclohexyl, the hydroboration product **2af** was obtained in 76% yield. Changing R to a sterically bulkier adamantly group resulted in a slightly lower yield (**2ag**, 56%). Substrates with 2,2-dimethylbutyl and 1-methylcyclopropyl, and substituents containing chloride are all converted to their corresponding hydroboration products **2ah**−**2aj** in yields of up to 64%. Sulfonamide is also tolerated in our system, which get the hydroboration product **2ak** in 66% yield. The reaction also proceeded with thioamide (**2al**). Finally, we found that cyclopropylamines derived from *Oleanolic Acid* and *Gemfibrozil* also reacted well in our system; the corresponding products, **2am** and **2an**, were obtained in yields of 49% and 59%, respectively.

### Hafnium-catalyzed reaction

**Fig. 3:**
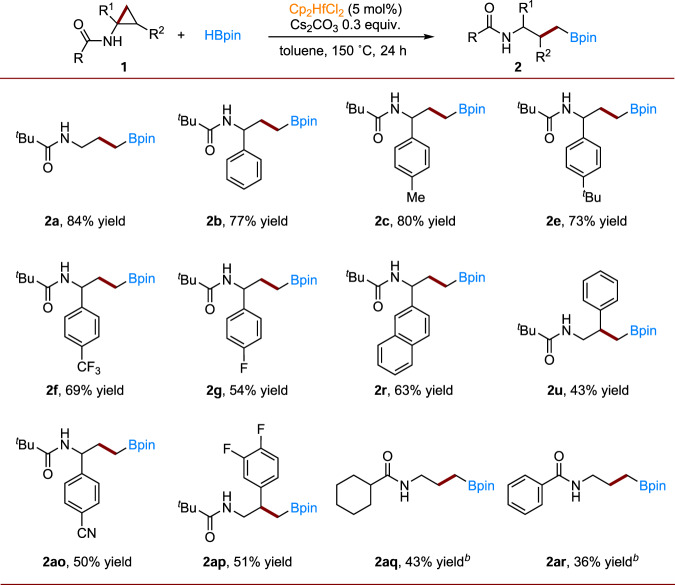
Hafnium-catalyzed hydroboration of cyclopropylamines—generality studies. ^*a,b,c a*^Reaction conditions: 0.2 mmol **1**, HBpin (2.0 equiv.), Cs_2_CO_3_ (30 mol%), Cp_2_HfCl_2_ (5 mol%) and 1 mL toluene in a 15 mL pressure tube at 150 °C for 24 h; ^*b*^HBpin (3.0 equiv.), Cs_2_CO_3_ (60 mol%), Cp_2_HfCl_2_ (10 mol%) and 1 mL toluene in a 15 mL pressure tube at 150 °C for 72 h; ^*c*^Isolated yields are given.

Compared with zirconium, hafnium has received less attention as a homogeneous catalyst in organic reactions. To our knowledge, reactivity toward C−C single bonds activation is also unknown. After successfully establishing zirconium-catalyzed C−C single bond activation of cyclopropylamines and their subsequent hydroboration, we further explored the reactivity of a hafnium complex towards C−C single bonds. We established that the base plays an essential role in tuning the reactivity. Eventually, Cs_2_CO_3_ was found to be the optimal base (Supplementary Table 5). Then, under the optimal reaction conditions, we conducted substrate scope generality studies (Fig. 3). We found that the hafnium system is not only suitable for substrates that work in the zirconium system but also for substrates that do not work there. For example, substrates with a −CN group do not react with the zirconium catalyst, but a 50% yield of product **2ao** was obtained with hafnium. Product **2ap**, with two fluorides on the phenyl ring, was also obtained in 51% yield. Additionally, cyclohexyl- (**2aq**) and phenyl-substituted substrates (**2ar**) were also applicable in the hafnium system.

### Synthetic derivation

**Fig. 4:**
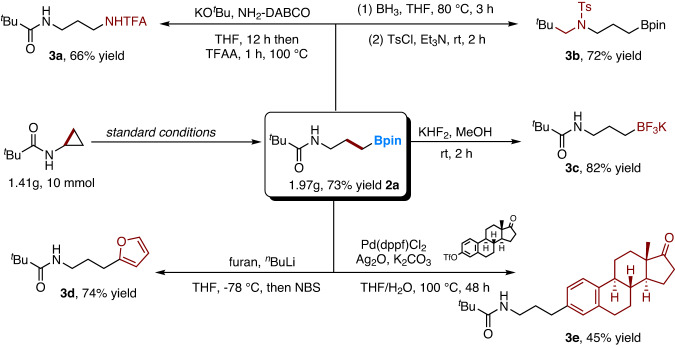
Synthetic Applications. Reaction in gram scale and further derivatization of **2a**. Isolated yields are given; for detailed reaction conditions, please refer to the supplementary information.

The practical utilization of our system was then demonstrated on a gram scale (Fig. 4). When we subjected 10 mmol of **1a** to our standard reaction conditions, we obtained **2a** in 73% yield (1.97 g). Furthermore, the synthetic derivatization of **2a** was demonstrated. Using the aminoazanium of DABCO as an amination reagent^91^, and then protecting the amine with TFAA, the corresponding TFA-amide **3a** was obtained in 66% yield. γ-Boronated amine **3b**, which is otherwise difficult to obtain^92^, was obtained in 72% yield by reducing the amide functional group to an amine and then protecting it with TsCl. Product **2a** can be transformed into potassium trifluoroborate salt **3c** using KHF_2_ (82% yield). Treating **2a** with furan-2-yllithium followed by NBS afforded the arylated product **3d** in 74% yield. Finally, Pd-catalyzed Suzuki−Miyaura coupling of **2a** with Estrone-derived triflate gave **3e** in 45% yield.

### Mechanistic studies

**Fig. 5:**
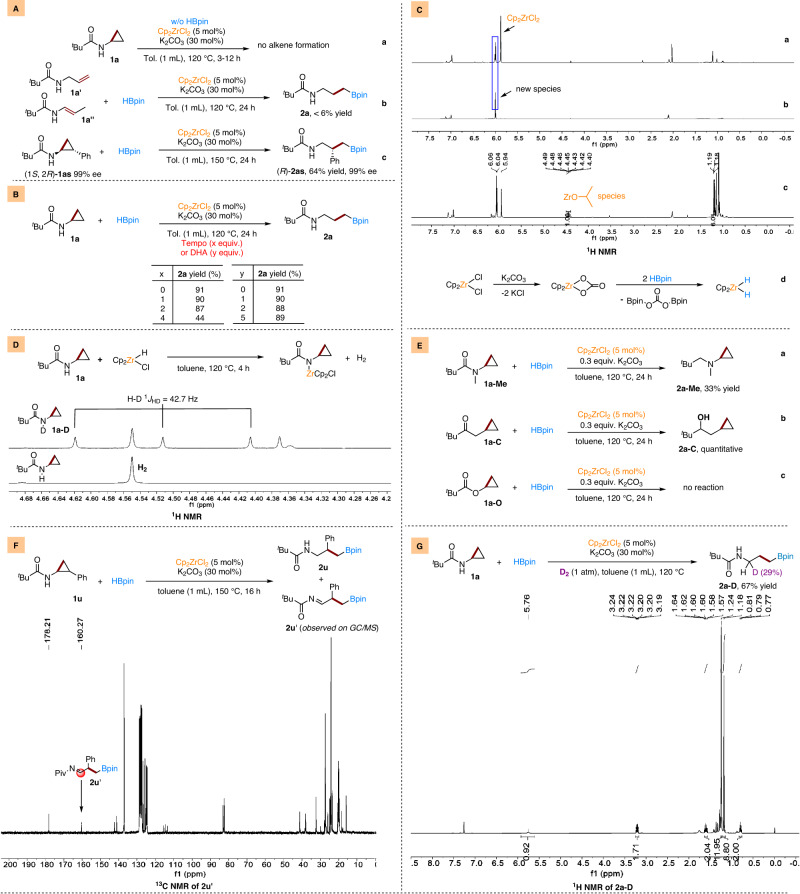
Experimental mechanism studies. **A** possible alkene intermediate formation; **B** possible radical pathway; **C** ^1^H NMR spectra show the formation of Zr−H species; **D** ^1^H NMR spectra show the release of H_2_ or HD by reacting Cp_2_ZrHCl with **1a**; **E** Control experiments to show the importance of N−H bond; **F** Detection of imide intermediate **2u**’ by crude ^13^C NMR spectroscopy; **G** Deuterium labeling experiment by introducing D_2_ in the standard reaction.

To shed light on the reaction mechanism, several control experiments were performed (Fig. 5). The possible formation of an alkene intermediate via ring-opening of cyclopropanes followed by hydroboration was studied. However, no alkenes were detected after 3 or 12 h under standard reaction conditions with or without HBpin (Fig. 5Aa, Supplementary Fig. 1). Utilization of alkenes **1a’** and **1a”** afforded less than 6% **2a** (Fig. 5Ab). When enantioenriched substrate (1*S*, 2*R*)-**1as** was applied, the desired product (*R*)-**2as** was obtained without erosion of the enantioselectivities (Fig. 5Ac, Supplementary Figs. 2, 3). Those results excluded a consecutive cyclopropane ring opening-hydroboration process. Then, the possibility of a reaction pathway that involved a radical species was investigated. TEMPO (2,2,6,6-tetramethylpiperidinyloxyl) (1−2 equiv) had almost no effect on the results. However, upon increasing the amount thereof (4 equiv.) the yields of **2a** decreased to 44% (Fig. 5B). At this point, it should be borne in mind that TEMPO inhibition experiments can sometimes provide ambiguous results^93^. Thus, additional experiments with the addition of 9,10-dihydroanthracene (DHA) were conducted; no effect on the yield of **2a** was detected (Fig. 5B). The results with TEMPO and DHA excluded a radical mechanism.

Then, the active zirconium catalytic species was studied. Upon the combination of Cp_2_ZrCl_2_ and K_2_CO_3_ in d^8^-Tol heated at 120 °C for 12 h, a new species appeared around 6.0 ppm in the ^1^H NMR spectrum (Fig. 5Ca). With 2 equiv. K_2_CO_3_ and heating for a longer reaction time, the Cp_2_ZrCl_2_ was fully converted to this new species (Fig. 5Cb). Then, the isolated new species was characterized by IR spectroscopy and was currently assigned to Cp_2_ZrCO_3_ by comparation with literature data (Supplementary Fig. 4)^94,95^. Nevertheless, upon further adding HBpin to the above solution, we could detect the formation of Zr–H species in the ^1^H NMR spectrum by trapping with acetone (Fig. 5Cc, Supplementary Fig. 5). This finding, together with the fact that Cp_2_ZrHCl or Cp_2_ZrH_2_ can catalyze the C−C bond hydroboration process without K_2_CO_3_ (65% and 95% yields, Supplementary Table 2), we concluded that Zr−H species are essentially the active catalysts via the consecutive reactions of Cp_2_ZrCl_2_, K_2_CO_3_, and HBpin (Fig. 5Cd). According to the work from Ganem^96^, Rosenthal^97^, and Cantat^98^, the active Zr−H species can interact with **1a** to form N−Zr species via metathesis with N−H bond. This is further proved in our case that Cp_2_ZrHCl reacts with the N−H group of **1a** with the release of H_2_ or HD when **1a**-D was used (Fig. 5D, Supplementary Fig. 6). To add further proof of the importance of the N−H, *N*-methylated analog substrate **1a-Me**, and replace the N−H with CH_2_ or O substrates **1a-C,** **1a-O** were subjected to our reaction conditions. As expected, no corresponding C−C bond hydroboration product were observed (Fig. 5E). Keep in mind that β-carbon elimination is one of the main pathway for C−C bond cleavage. It is natural to think that after the formation of N−Zr species, a β-carbon elimination may proceed to cleavage the C−C bond to produce an imino propyl zirconium species. This is consistent with the fact that we can observe the presence of the putative imine intermediate both on GC/MS and ^13^C NMR when substrate **1** **u** was used (Fig. 5F, Supplementary Figs. 7, 8).

**Fig. 6:**
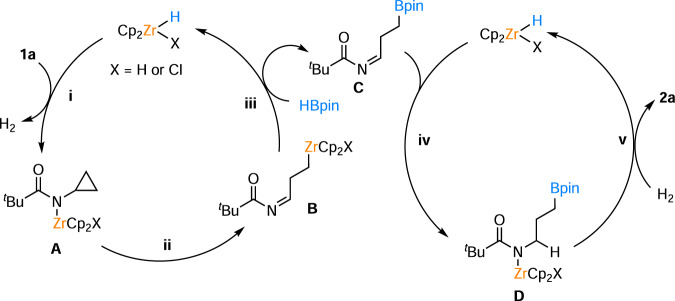
Proposed reaction pathway for the Zr-catalyzed C−C single bond hydroboration. Key steps for the transformation: i) N−H bond metathesis; ii) C−C activation; iii) B−H bond metathesis, iv) Zr−H hydride transfer; v) H_2_ metathesis.

Based on the above mechanistic study and our DFT calculation results (Supplementary Fig. 9), we conclude the following general reaction pathway for our Cp_2_ZrCl_2_/K_2_CO_3_ system (Fig. 6). First, the in-situ formed Zr−H species reacts with N−H bonds of the substrates to form N−Zr^IV^ species **A** via H_2_ release (i). Next, the C−C single bond is cleaved via β-carbon elimination of intermediate **A** to form the imino propyl zirconium species **B** (ii). Subsequently, intermediate **B** reacts with HBpin via C−Zr and H−B bond metathesis to give **C** and regenerate Zr−H species (iii). In the second catalytic cycle, Zr−H hydride transfer to intermediate **C** gives intermediate **D** (iv), which is further reduced by the previously released H_2_ to **2a** with hydrogenolysis or H_2_ metathesis (v). The last step is supported by the experiment that when we introduced 1 atm of deuterium gas into the standard reaction, 29% deuterium labeling at the α-carbon adjacent to N−H of **2a** could be obtained (Fig. 5G, Supplementary Fig. 10), suggesting that hydrogen metathesis occurred^99–101^.

In summary, an unprecedented zirconium- and hafnium-catalyzed C−C single bond activation and subsequent hydroboration is realized using a catalytic system based on Cp_2_ZrCl_2_ and Cp_2_HfCl_2_. Our catalytic approach applies to various cyclopropylamines. Selective cleavage of the proximal C_α_−C_β_ single bond was achieved, with the tolerance of multiple functional groups as well as bio- and medicine-derived substrates. Mechanistic studies disclose that the in-situ generated Zr−H species and the free N−H group of the substrates play key roles in this transformation via Zr−H and N−H metathesis to form N−Zr^IV^ species, and the subsequent C−C single bond activation is realized via a β-carbon elimination route. Our work presents an unprecedented group IV metal-catalyzed C−C single bond activation and hydroboration reaction. The C−C single bond activation model that was well studied for late-transition metals, were also elaborated to be applicable to the group IV metals.

## Methods

### General procedure for the Zr-catalyzed hydroboration of cyclopropylamines

In a nitrogen-filled glovebox, to a 15 mL pressure tube with a magnetic stirrer was added catalytic amount of Cp_2_ZrCl_2_ (0.01 mmol, 2.9 mg), K_2_CO_3_ (0.06 mmol, 8.3 mg), corresponding cyclopropylamine substrates (0.2 mmol), HBpin (0.3 mmol, 43.5 μL), and toluene (1 mL) in a sequence manner. Then, the pressure tube was taken out of the glove box and allowed to stir at 120 °C for 24 h. Upon completion, all the solvent was evaporated, and the crude product was isolated on silica gel using flash chromatography with dichloromethane/ethyl acetate as the eluent to give the corresponding products.

### Supplementary information

Supplementary Information

Peer Review File

## Data Availability

Experimental details, Synthetic Procedures, Tables for condition optimizations (Supplementary Tables 1–5), Figures for mechanistic studies, and DFT calculations, NMR spectra (Supplementary Figs. 1–248), products characterizations are included in the Supplementary Information. All other data are available from the corresponding author upon request.
