# Supplementary material for: Zirconium and hafnium catalyzed C–C single bond hydroboration
Source: Nat Commun. 2024 Feb 28;15:1846. doi: 10.1038/s41467-024-45697-y (PMC10902336; doi:10.1038/s41467-024-45697-y)
Supplement: Supplementary file 1 — Supplementary Information [file 41467_2024_45697_MOESM1_ESM.pdf]

Supplementary Information

Zirconium and Hafnium Catalyzed C−C Single Bond Hydroboration

Sida Li,[1,4] Haijun Jiao,[2,*] Xing-Zhong Shu,[3] and Lipeng Wu[1,5,*]

[1]State Key Laboratory for Oxo Synthesis and Selective Oxidation, Lanzhou Institute of Chemical Physics (LICP), Chinese Academy of Sciences, Lanzhou, 730000 (P. R. China)

[2]Leibniz-Institut für Katalyse e. V. Albert-Einstein-Straße 29a, 18059 Rostock, (Germany)

[3]State Key Laboratory of Applied Organic Chemistry, College of Chemistry and Chemical Engineering, Lanzhou University, Lanzhou, 730000 (P. R. China)

[4]University of Chinese Academy of Sciences, Beijing, 100049 (P. R. China)

[5]College of Material Chemistry and Chemical Engineering, Key Laboratory of Organosilicon Chemistry and Material Technology, Ministry of Education, Hangzhou Normal University, Hangzhou, 311121 (P. R. China)

Email: haijun.jiao@catalysis.de; lipengwu@licp.cas.cn

Table of Contents

General Information.. S2

General Procedure for the Synthesis of Substituted Cyclopropylamines S2

General Procedure for the Zr-catalyzed Hydroboration of Cyclopropanes S3

General Procedure for the Hf-catalyzed Hydroboration of Cyclopropanes............... S3

Procedure for the Zr-catalyzed Hydroboration of Enantioenriched Cyclopropanes .. S4

Synthetic derivatization of **2a**... S4

Supplementary Figures S8

Condition Optimization Tables... S18

Cyclopropylamines and Product Characterization S23

NMR Spectra.. S56

References.. S175

General Information

Manipulations were carried out under an atmosphere of dry and deoxygenated N_2 using Schlenk line or in a glovebox (H_2O and O_2 < 0.01 ppm). Glassware was pre-dried in an oven at 150 °C for several hours and cooled before use. Solvents were purchased as super dry solvent or purified via standard purification operations. Zirconium and hafnium complexes were purchased from Sigma-Aldrich, Bidepharm and Macklin company. HBpin was purchased from Allylchem company. Cyclopropylamine substrates were synthesized from the corresponding cyclopropylamines and acid chloride. Chemicals used for the substrate synthesis were purchased from Sigma-Aldrich, Energy, Adamas, Bidepharm, Macklin company or synthesized via known procedures. ^{1}H NMR, ^{13}C NMR, ^{11}B NMR spectra were recorded on Bruker Advance Neo 400 MHz NMR at room temperature using $CDCl_3$ as a solvent. Chemical shifts (δ) are given in parts per million (ppm). Coupling constants (*J*) are given in Hertz (Hz). Thin-layer chromatography (TLC) employed glass 0.25 mm silica gel plates. Flash chromatography columns were packed with 200-300 mesh silica gel in petroleum (bp. 60-90 °C). High-Resolution MS analyses were performed on Waters Micromass Q-TOF Premier Mass Spectrometer. Electron impact (EI) mass spectra were recorded on SHIMADZU GCMS-QP2010 SE mass spectrometer.

General Procedure for the Synthesis of Substituted Cyclopropylamines

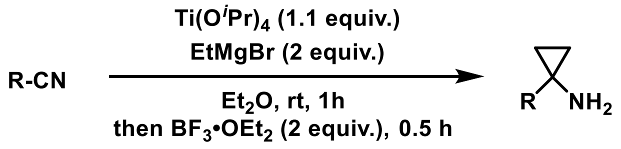

Following a reported procedure,[1] ethylmagnesium bromide (2.2 mmol, 1.0 M in THF) was added to a solution contains nitriles (1 mmol), Ti(O^{i}Pr)$_4$ (1.1 mmol) in THF at -70 °C. The solution was stirred for 10 min and then warmed to room temperature before $BF_3{\cdot}OEt_2$ (2 mmol) was added. After the above mixture was stirred for 1 h, 1 N HCl (3 mL) and additional 15 mL Et_2O were added. NaOH (10% aq.) was then added and the mixture was extracted with ether. The combined ether layers were dried using Na_2SO_4,

filtered and concentrated. The residue was was isolated on silica gel using flash chromatography.

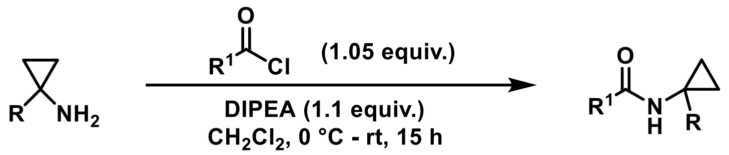

Following a reported procedure,[2-4] a solution of cyclopropylamines (10 mmol) and DIPEA (11.5 mmol) in CH_2Cl_2 (10 mL) was added slowly to a solution of the corresponding acid chloride (11 mmol) in CH_2Cl_2 (10 mL) at 0 °C. The reaction mixture was stirred at room temperature for 15 h. Then saturated aq. $NaHCO_3$ (3 mL) was added, which was extracted with CH_2Cl_2. The combined organic layers were was washed with brine, and dried using Na_2SO_4, filtered and concentrated. The residue was was isolated on silica gel using flash chromatography. A final crystallization from hexane give pure substituted cyclopropylamines.

General Procedure for the Zr-catalyzed Hydroboration of Cyclopropanes

In a nitrogen-filled glovebox, to a 15 mL pressure tube with a magnetic stirrer was added catalytic amount of Cp_2ZrCl_2 (0.01 mmol, 2.9 mg), K_2CO_3 (0.06 mmol, 8.3 mg), corresponding cyclopropylamine substrates (0.2 mmol), HBpin (0.3 mmol, 43.5 μL), and toluene (1 mL) in a sequence manner. Then the pressure tube was taken out of the glove box and allowed to stir at 120 °C for 24 h. Upon completion, all the solvent was evaporated, and the crude product was isolated on silica gel using flash chromatography with dichloromethane/ethyl acetate as the eluent to give the corresponding products.

General Procedure for the Hf-catalyzed Hydroboration of Cyclopropanes

In a nitrogen-filled glovebox, to a 15 mL pressure tube with a magnetic stirrer was added catalytic amount of Cp_2HfCl_2 (0.01 mmol, 3.8 mg), Cs_2CO_3 (0.06 mmol, 19.5 mg), corresponding cyclopropylamine substrates (0.2 mmol), HBpin (0.3 mmol, 43.5 μL), and toluene (1 mL) in a sequence manner. Then the pressure tube was taken out of the glove box and allowed to stir at 120 °C for 24 h. Upon completion, all the solvent

was evaporated, and the crude product was isolated on silica gel using flash chromatography with dichloromethane/ethyl acetate as the eluent to give the corresponding products.

Procedure for the Zr-catalyzed Hydroboration of Enantioenriched Cyclopropanes

In a nitrogen-filled glovebox, to a 15 mL pressure tube with a magnetic stirrer was added catalytic amount of Cp_2ZrCl_2 (0.01 mmol, 2.9 mg), K_2CO_3 (0.06 mmol, 8.3 mg), corresponding cyclopropylamine (1*S*, 2*R*)-**1r** (0.2 mmol, 43.4 mg), HBpin (0.3 mmol, 43.5 μL), and toluene (1 mL) in a sequence manner. Then the pressure tube was taken out of the glove box and allowed to stir at 150 °C for 24 h. Upon completion, all the solvent was evaporated, and the crude product was isolated on silica gel using flash chromatography with dichloromethane/ethyl acetate as the eluent to give (*R*)-**2r** in 99% ee as a colorless oil (ChiralPAK IC column, Hexanes/^{i}PrOH = 90/10, 220 nm, flow: 1 mL/min, T = 25 °C).

Synthetic derivatization of 2a

Procedure for the Synthesis of 3a from 2a

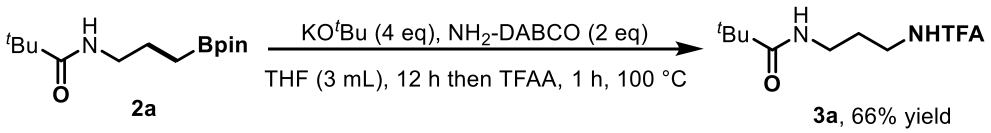

The synthetic procedure was adapted from the literature.[5] In a nitrogen-filled glovebox, to a 15 mL pressure tube with a magnetic stirrer was added KO^{t}Bu (111.7 mg, 1.0 mmol), **2a** (67.3 mg, 0.25 mmol), NH_2-DABCO (192 mg, 0.5 mmol), THF (3 mL) in a sequence manner. Then the pressure tube was taken out of the glove box and allowed to stir at 100 °C for 12 h. Upon completion, TFAA (70 μL, 0.5 mmol) was added, and the reaction was allowed to stir at 100 °C for 1 h. After that, the reaction was cooled to room temperature, diluted with ethyl acetate and water, then the organic solvent was

concentrated in vacuo, and the crude product was isolated on silica gel using flash chromatography with petroleum ether/ethyl acetate (2:1) as the eluent to give the product (41.9 mg, 66% yield).

Procedure for the Synthesis of 3b from 2a

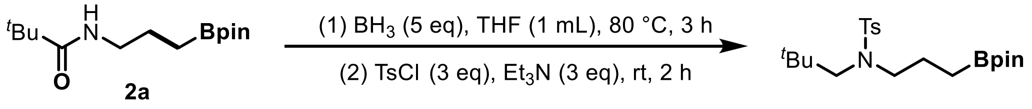

3b, 72% yield

In a nitrogen-filled glovebox, to a 15 mL pressure tube with a magnetic stirrer was added **2a** (53.8 mg, 0.2 mmol) and BH_3 (1.0 M in THF, 1 mL, 1mmol) in a sequence manner. Then the pressure tube was taken out of the glove box and allowed to stir at 80 °C for 3 h. After that, the reaction was cooled to 0 °C, added TsCl (114.0 mg, 0.6 mmol), Et_3N (85.0 μL, 0.6 mmol) at nitrogen atmosphere. Then, the mixture was allowed to warm to room temperature. After that, saturated aq. $NaHCO_3$ (2 mL) was added and the reaction mixture was diluted with Et_2O (15 mL) and water (15 mL). The combined ethereal solution was dried over anhydrous $MgSO_4$. After removal of the solvent, the residue was purified by column chromatography on silica using with petroleum ether/ethyl acetate (5:1) as the eluent to give the product (58.9 mg, 72% yield).

Procedure for the Synthesis of 3c from 2a

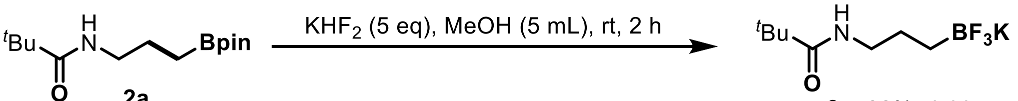

3c, 82% yield

The synthetic procedure was adapted from the literature.[3,4,6] To a solution of **2a** (53.8 mg, 0.2 mmol) in MeOH (5 mL) was added KHF_2 (0.5 mL of saturated aqueous solution (4.0–4.5 M, 1.0 mmol) dropwise at room temperature. The solution was stirred at room temperature for 2 h, then concentrated in vacuo. To the residue was added 60% aqueous MeOH, which was then concentrated in vacuo. This procedure was repeated twice. The resultant white solid was dissolved in hot acetone, then ether was added. The precipitate was washed with ether, and the resultant precipitate was dissolved in

acetone, and concentrated in vacuo overnight to afford potassium trifluoroborate as a white solid (40.8 mg, 82% yield).

Procedure for the Synthesis of 3d from 2a

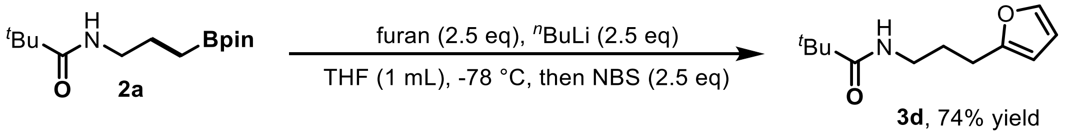

3d, 74% yield

The synthetic procedure was adapted from the literature.[3,4,6] In a nitrogen-filled glovebox, to a 15 mL pressure tube with a magnetic stirrer was added furan (34 mg, 0.5 mmol) and THF (1 mL), *n*-BuLi (0.2 mL, 2.5 M in Hexane, 0.5 mmol) was added at -78 °C and stirred for 2 h. A THF (1 mL) solution of **2a** (53.8 mg, 0.2 mmol) was then added. The resulting mixture was then allowed to stir at -78 °C for another 2 h. A solution of an NBS (89.0 mg, 0.5 mmol) was added dropwise as a solution in THF (1.0 mL). After 1 h at -78 °C, saturated aq. $Na_2S_2O_3$ (2 mL) was added and the reaction mixture was allowed to warm to room temperature. The reaction mixture was diluted with Et_2O (15 mL) and water (15 mL). The combined ethereal solution was dried over anhydrous $MgSO_4$. After removal of the solvent, the residue was purified by column chromatography on silica using petroleum ether/ethyl acetate (5:1) as the eluent to give the product (30.9 mg, 74% yield).

Procedure for the Synthesis of 3e from 2a

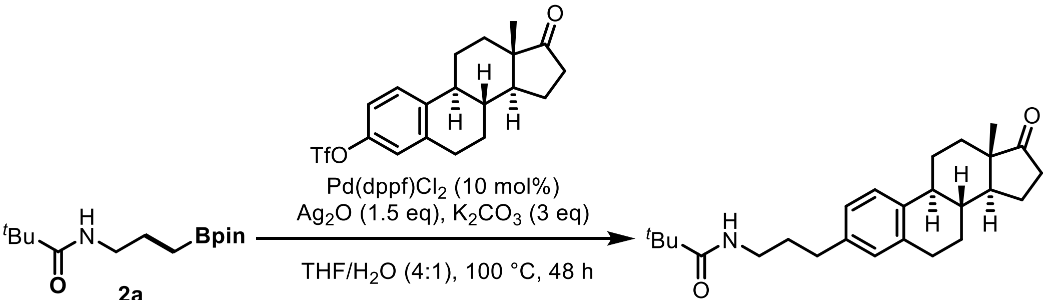

3e, 45% yield

The synthetic procedure was adapted from the literature.[3,4,6] In a nitrogen-filled glovebox, to a 15 mL pressure tube with a magnetic stirrer was added Pd(dppf)Cl_2 (14.6 mg, 0.02 mmol), Ag_2O (69.5 mg, 0.3 mmol), K_2CO_3 (82.9 mg, 0.36 mmol), **2a** (53.8 mg, 0.2 mmol), estrone-derived triflate (120.6 mg, 0.3 mmol), THF/H_2O (1.6

mL/0.2 mL) in a sequence manner. Then the pressure tube was taken out of the glove box and allowed to stir at 100 °C for 48 h. Upon completion, the reaction was then cooled to room temperature, diluted with dichloromethane and concentrated in vacuo, and the crude product was isolated on silica gel using flash chromatography with petroleum ether/ethyl acetate (4:1) as the eluent to give the product (35.6 mg, 45% yield).

Supplementary Figures

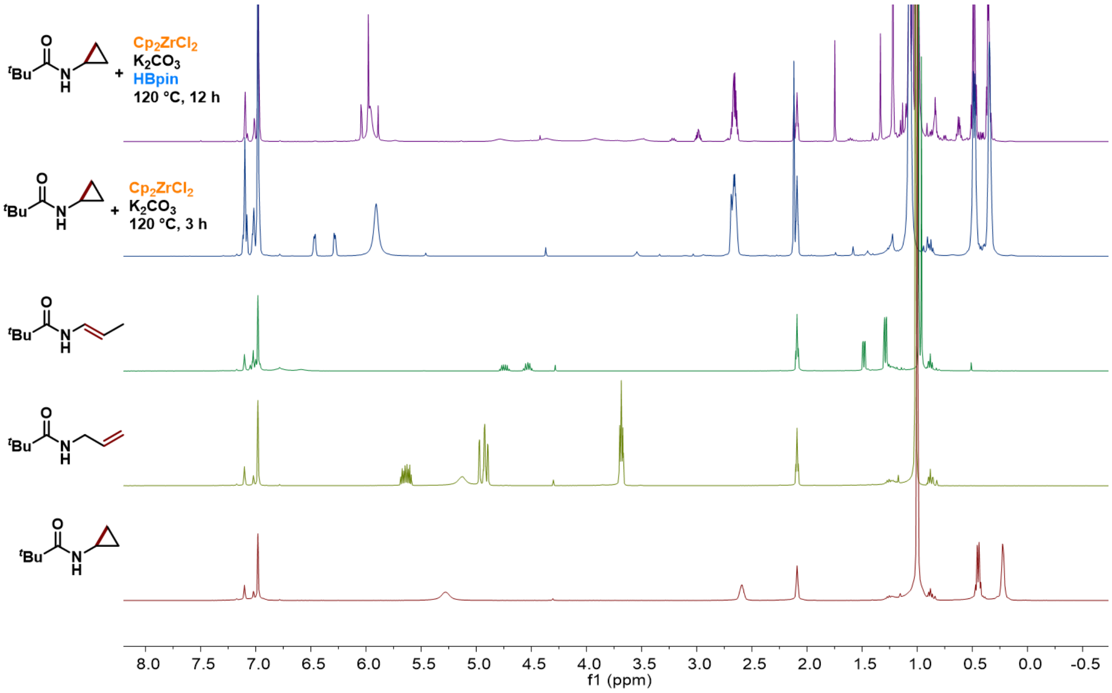

Supplementary Figure 1. ^{1}H NMR spectra of different substrates and possible alkene intermediates in d^8-toluene to show that no alkenes were formed during the catalytic process.

HPLC Analytical Data
OD-H, 1.0 mL/min, 90/10, 220 nm, 25 oC

Sample Name : LSD-7-9-11-RAC
Sample ID : LSD-7-9-11-RAC
Data File : LSD-7-9-11-RAC.lcd
Method File : 1.lcm

Spectrum

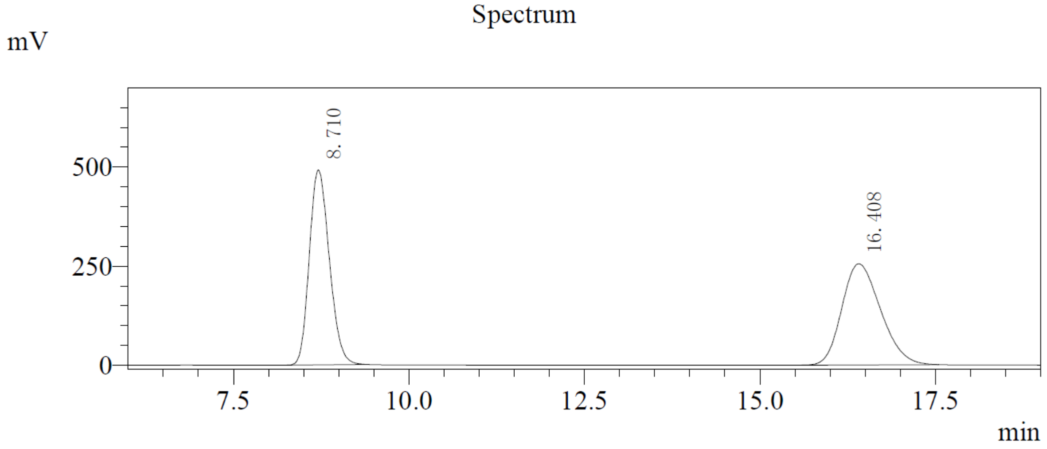

Results

???A Channel 2 220nm

Peak ID	Ret. Time	Height	Area	Area%
1	8.710	491644	9748433	49.911
2	16.408	255050	9783021	50.089
Total		746695	19531454	100.000

HPLC Analytical Data
OD-H, 1.0 mL/min, 90/10, 220 nm, 25 oC

Sample Name : LSD-7-9-11-S,R
Sample ID : LSD-7-9-11-S,R
Data File : LSD-7-9-11-S,R.lcd
Method File : 1.lcm

Spectrum

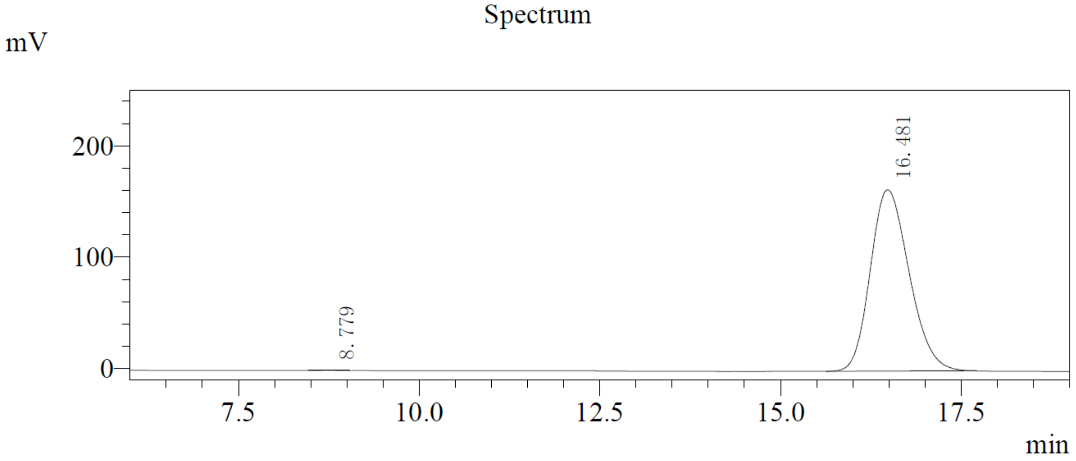

Results

???A Channel 2 220nm

Peak ID	Ret. Time	Height	Area	Area%
1	8.779	277	4840	0.079
2	16.481	162772	6083060	99.921
Total		163049	6087900	100.000

Supplementary Figure 2. HPLC Analytical spectra of racemic and chiral substrate **1as**.

HPLC Analytical Data

IC, 1.0 mL/min, 90/10, 220 nm, 25 oC

Sample Name	: LSD-7-9-12-RAC'
Sample ID	: LSD-7-9-12-RAC'
Data File	: LSD-7-9-12-RAC'.lcd
Method File	: l.lcm

Spectrum

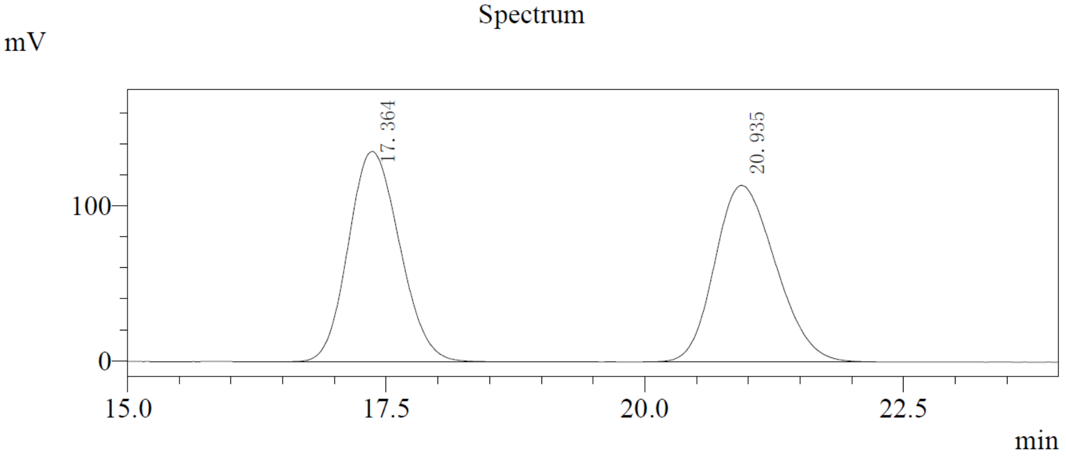

Results

???A Channel 2 220nm

Peak ID	Ret. Time	Height	Area	Area%
1	17.364	135512	4677445	50.101
2	20.935	113701	4658552	49.899
Total		249214	9335997	100.000

HPLC Analytical Data

IC, 1.0 mL/min, 90/10, 220 nm, 25 oC

Sample Name	: LSD-7-9-12-R-
Sample ID	: LSD-7-9-12-R-
Data File	: LSD-7-9-12-R-.lcd
Method File	: l.lcm

Spectrum

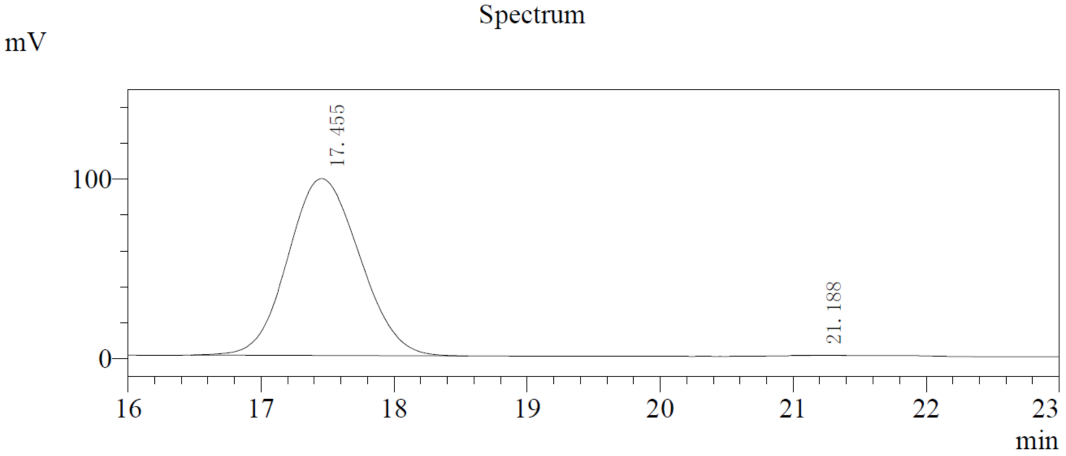

Results

???A Channel 2 220nm

Peak ID	Ret. Time	Height	Area	Area%
1	17.455	98545	3700300	99.953
2	21.188	124	1746	0.047
Total		98669	3702046	100.000

Supplementary Figure 3. HPLC Analytical spectra of racemic and chiral substrate **2as**.

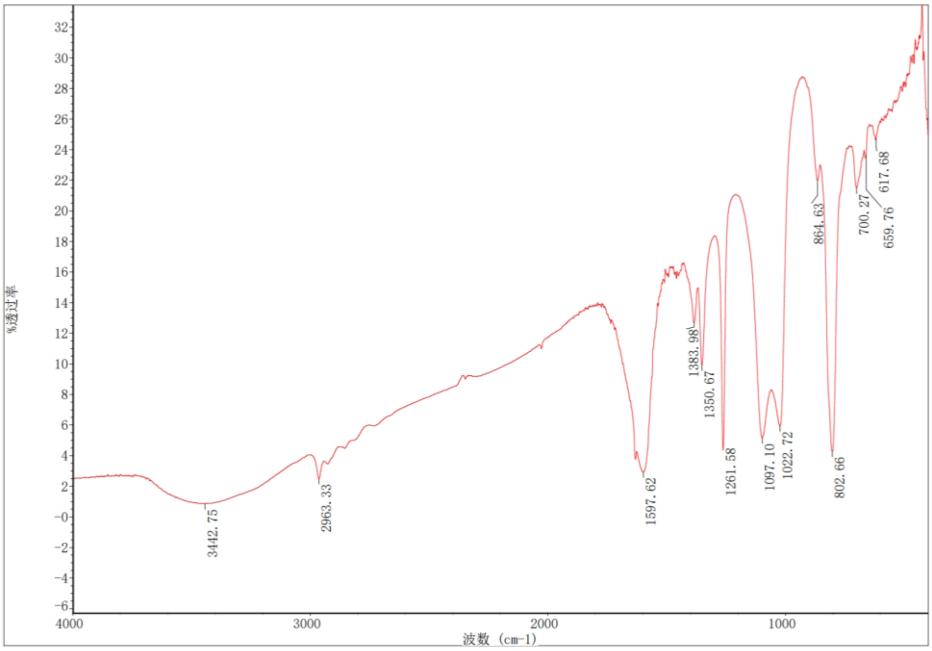

Supplementary Figure 4. IR spectrum of Cp_2ZrCO_3. In the IR spectra, peaks at 1597 and 1350 cm^{-1} could be assigned to the asymmetric stretching mode of CO_3^{2-}; peaks at 1097 and 864 cm^{-1} are designated to the symmetric stretching mode of CO_3^{2-}. The band at 660 cm^{-1} is attributed to the stretching vibration of Zr−O[7,8].

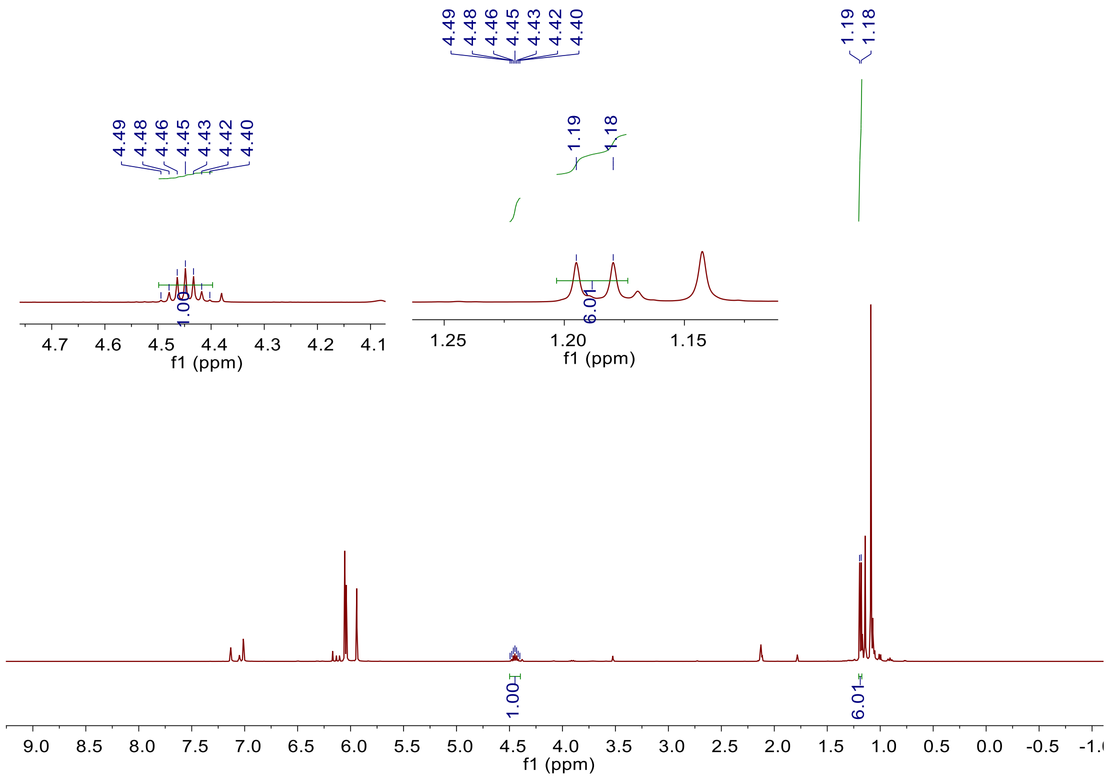

Supplementary Figure 5. ^{1}H NMR spectrum of 0.1 mmol Cp_2ZrCl_2, 0.2 mmol K_2CO_3 and 0.3 mmol HBpin with 0.1 mmol acetone in d^8-toluene after heating at 120 °C for 24 h. The hept peaks at 4.45 ppm with J value of 6.1 Hz indicates the formation of Zr−O^{i}Pr from Zr−H trapped by acetone.[7]

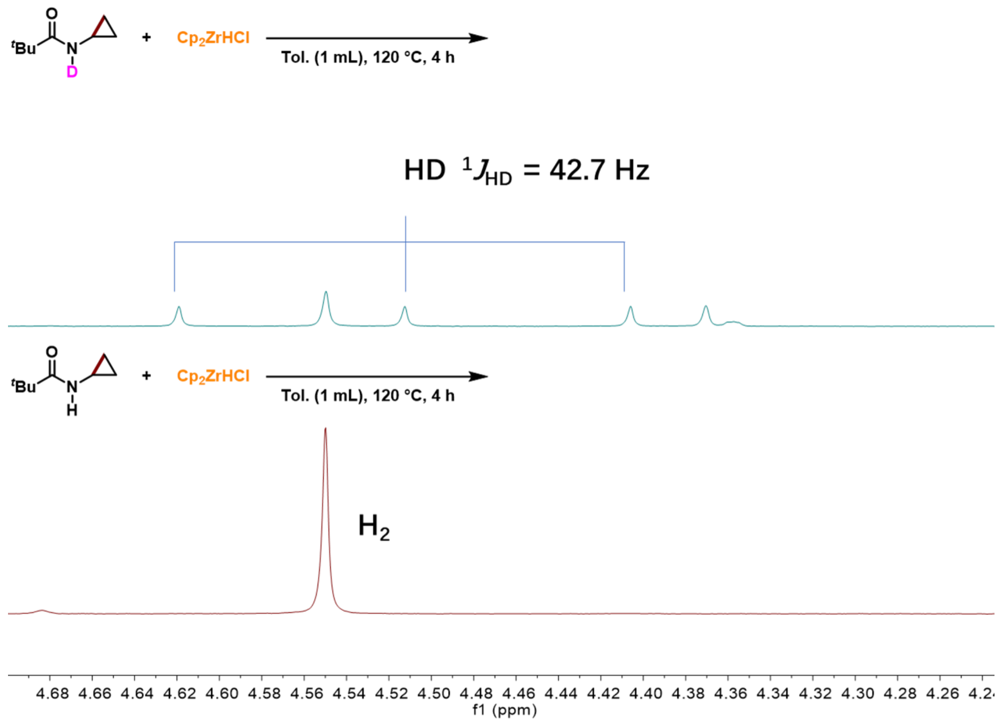

Supplementary Figure 6. ^{1}H NMR spectra of cyclopropylamines **1a** and **1a-D** react with Cp_2ZrHCl in d^8-toluene, the formation of H_2 and HD indicate the reaction of cyclopropylamine with Zr−H species.

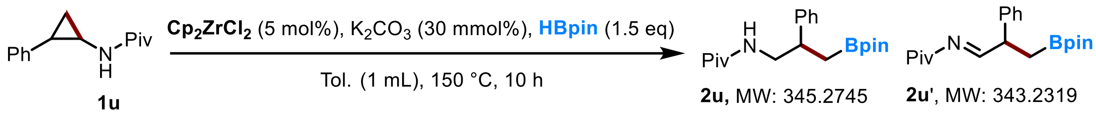

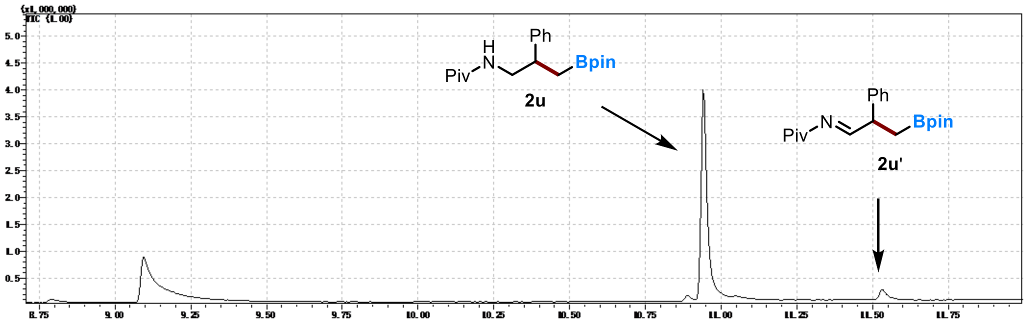

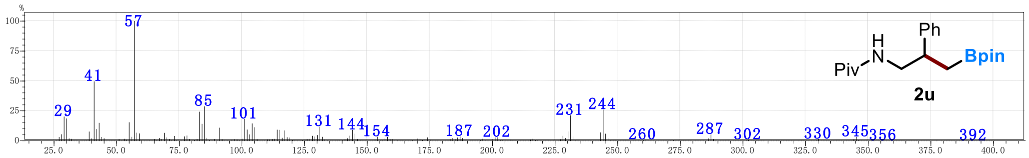

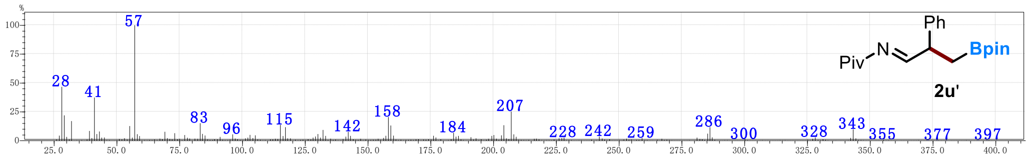

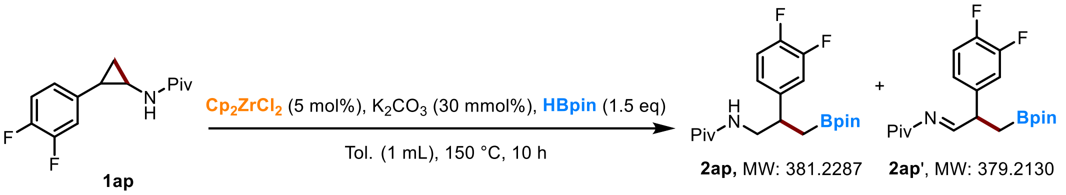

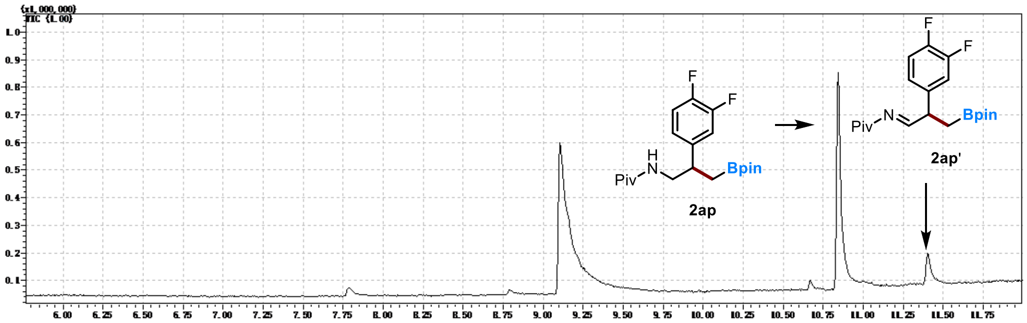

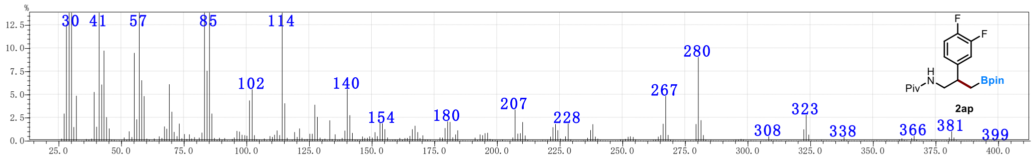

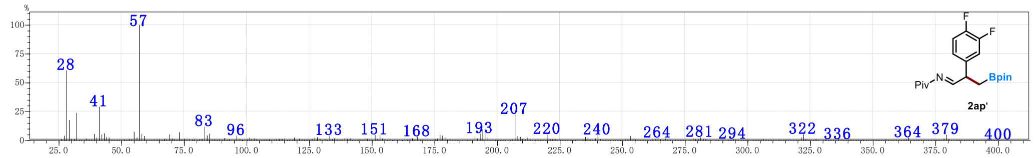

Supplementary Figure 7. GC/MS chromatogram of **2u'** and **2ap'**.

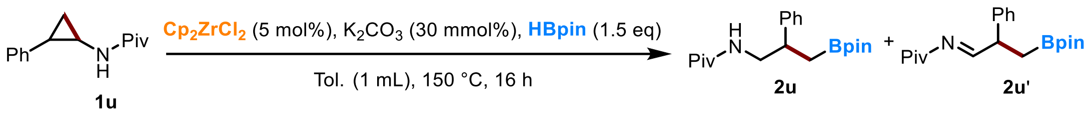

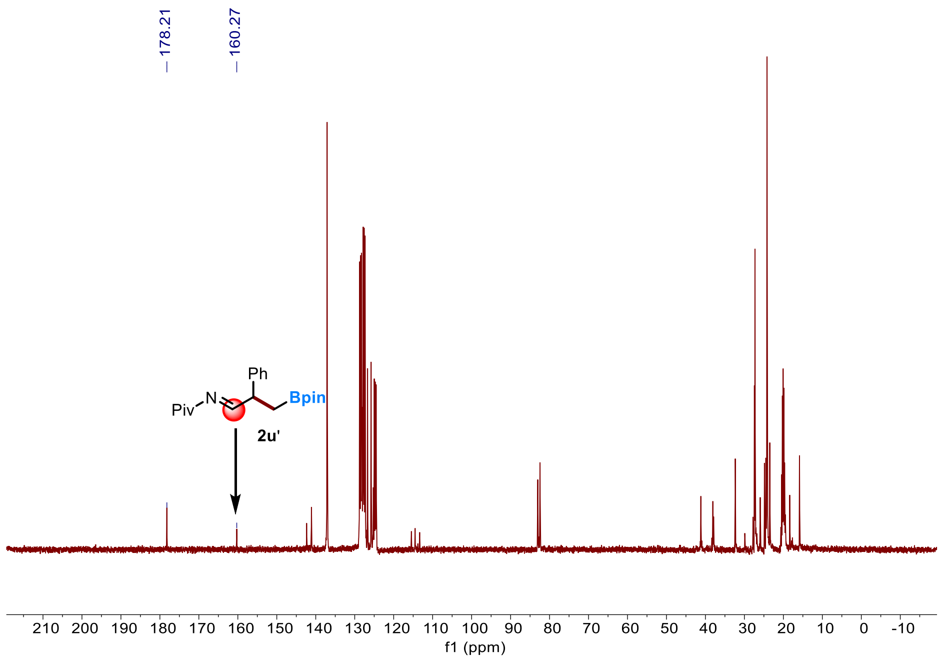

Supplementary Figure 8. Detection of imide intermediate **2u'** by crude ^{13}C NMR spectroscopy.

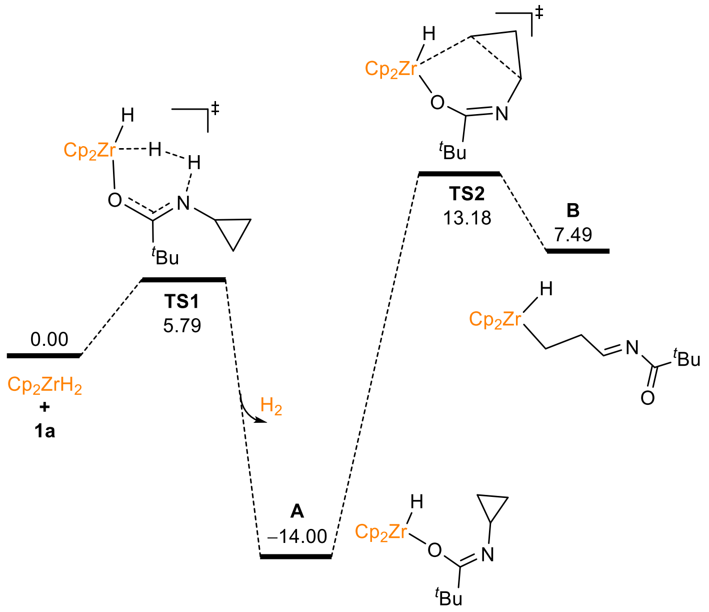

Supplementary Figure 9. BP86-D3-SCRF/TZVP Gibbs free energy diagram of the $Zr^{IV}H$-catalyzed C−C bond cleavage (ΔG, kcal/mol).

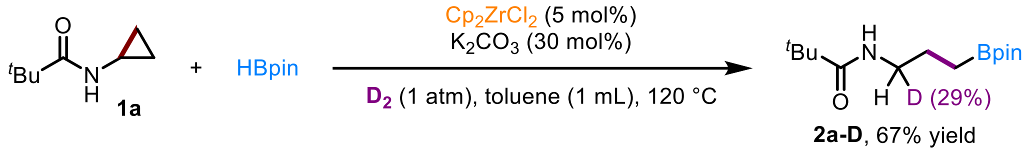

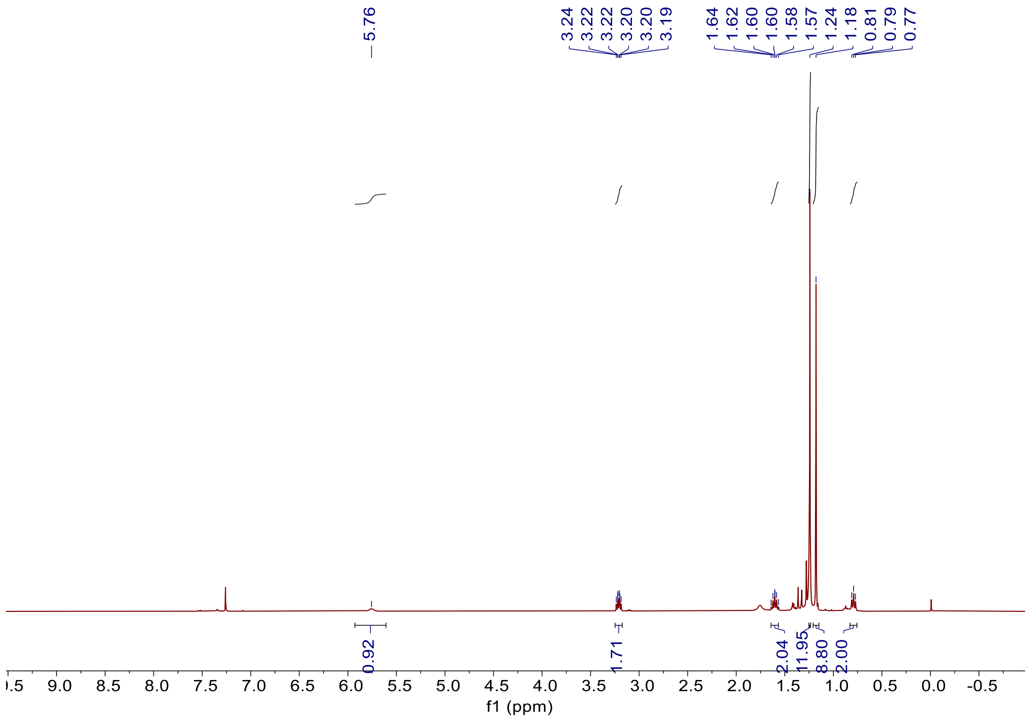

Supplementary Figure 10. ^{1}H NMR spectrum of **2a**-D obtained by deuterium labeling experiment with D_2.

Condition Optimization Tables

Supplementary Table 1. Zirconium-catalyzed hydroboration of cyclopropylamines – effect of bases[a]

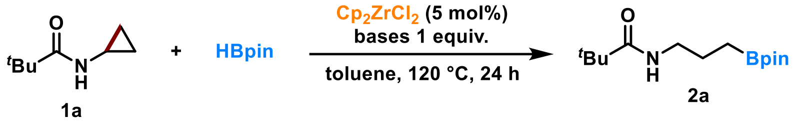

Entries	Bases	Yields (%)[b] **2a**
1	-	-
2	KH_2PO_4	-
3	KF	-
4	CsF	20
5	Cs_2CO_3	35
6	Li_2CO_3	58
7	Na_2CO_3	46
8	K_2CO_3	81

[a]Reaction conditions: 0.2 mmol **1a**, HBpin (1.5 equiv.), Cp_2ZrCl_2. (5 mol%), bases (1 equiv.) and 1 mL toluene in a 15 mL pressure tube at 120 °C for 24 h; [b]Yields were determined by GC with dodecane as internal standard.

Supplementary Table 2. Zirconium-catalyzed hydroboration of cyclopropylamines – effect of catalysts[a]

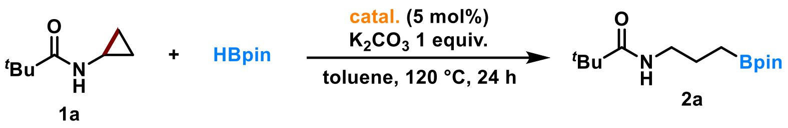

Entries	Catal.	Yields (%)[b] **2a**
1	Cp_2ZrCl_2	81
2	$Cp^*{}_2ZrCl_2$	-
3	Cp_2ZrMe_2	10
4	Cp_2ZrHCl	50
5	$CpZrCl_3$	-
6	-	-
7	Cp_2ZrH_2[c]	95
8	Cp_2ZrHCl[c]	65

[a]Reaction conditions: 0.2 mmol **1a**, HBpin (1.5 equiv.), catal. (5 mol%), K_2CO_3 (1 equiv.) and 1 mL toluene in a 15 mL pressure tube at 120 °C for 24 h; [b]Yields were determined by GC with dodecane as internal standard; [c]No base was added.

Supplementary Table 3. Zirconium-catalyzed hydroboration of cyclopropylamines – effect of solvents[a]

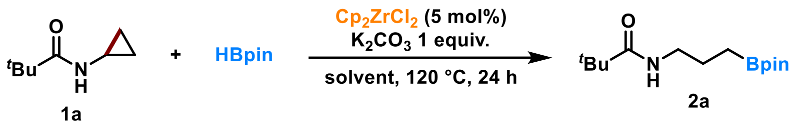

Entries	Solvents	Yields (%)[b] **2a**
1	toluene	81
2	*o*-xylene	48
3	*p*-xylene	59
4	ethylbenzen	47
5	chlorobenze	24
6	mesitylene	55
7	THF	trace
8	dioxane	-
9	DCM	trace
10	MTBE	18
11	MeCN	-
12	hexane	42

[a]Reaction conditions: 0.2 mmol **1a**, HBpin (1.5 equiv.), Cp_2ZrCl_2 (5 mol%), K_2CO_3 (1 equiv.) and 1 mL solvent in a 15 mL pressure tube at 120 °C for 24 h; [b]Yields were determined by GC with dodecane as internal standard.

Supplementary Table 4. Zirconium-catalyzed hydroboration of cyclopropylamines – effect of base amount[a]

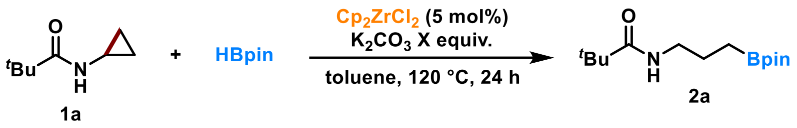

Entries	X	Yields (%)[b] 2a
1	0.05	27
2	0.1	68
3	0.2	71
4	0.3	91
5	0.5	88
6	1	81
7	1.5	71
8	2	65
9	3	53

[a]Reaction conditions: 0.2 mmol **1a**, HBpin (1.5 equiv.), Cp_2ZrCl_2 (5 mol%), K_2CO_3 (X equiv.) and 1 mL toluene in a 15 mL pressure tube at 120 °C for 24 h; [b]Yields were determined by GC with dodecane as internal standard.

Supplementary Table 5. Hafnium-catalyzed hydroboration of cyclopropylamines – effect of bases[a]

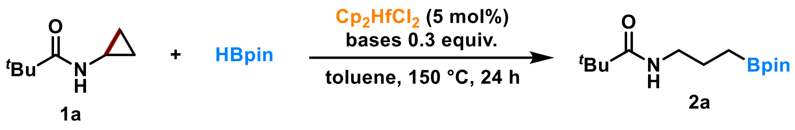

Entries	Bases	Yields (%)[b] **2a**
1	Li_2CO_3	11
2	Na_2CO_3	22
3	K_2CO_3	8
4	Cs_2CO_3	95
5	K_2HPO_4	34
6	^{n}BuLi	37
7	^{n}BuMgCl	13
8	KHMDS	93

[a]Reaction conditions: 0.2 mmol **1a**, HBpin (2.0 equiv.), Cp_2HfCl_2 (5 mol%), bases (0.3 equiv.) and 1 mL toluene in a 15 mL pressure tube at 150 °C for 24 h; [b]Yields were determined by GC with dodecane as internal standard.

Cyclopropylamines and Product Characterization

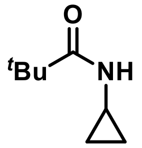

N-cyclopropylpivalamide 1a

Eluent: petroleum ether/ethyl acetate (5:1). White solid (1.27 g, 90% yield). **^{1}H NMR** (400 MHz, Chloroform-*d*) δ 5.70 (s, 1H), 2.72 – 2.66 (m, 1H), 1.16 (s, 9H), 0.80 – 0.73 (m, 2H), 0.47 – 0.41 (m, 2H). **^{13}C{^{1}H} NMR** (101 MHz, Chloroform-*d*) δ 179.9, 38.6, 27.6, 22.9, 6.8. Spectroscopic data are in agreement with those previously reported.[4]

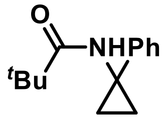

N-(1-phenylcyclopropyl)pivalamide 1b

Eluent: petroleum ether/ethyl acetate (5:1). White solid (1.73 g, 80% yield). **^{1}H NMR** (400 MHz, Chloroform-*d*) δ 7.30 – 7.25 (m, 2H), 7.22 – 7.14 (m, 3H), 6.27 (s, 1H), 1.29 – 1.25 (m, 2H), 1.23 – 1.18 (m, 11H). **^{13}C{^{1}H} NMR** (101 MHz, Chloroform-*d*) δ 178.5, 142.8, 128.4, 126.3, 125.3, 38.8, 34.9, 27.7, 18.1. Spectroscopic data are in agreement with those previously reported.[4]

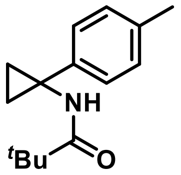

N-(1-(p-tolyl)cyclopropyl)pivalamide 1c

Eluent: petroleum ether/ethyl acetate (5:1). White solid (1.87 g, 81% yield). **^{1}H NMR** (400 MHz, Chloroform-*d*) δ 7.09 (q, *J* = 8.1 Hz, 4H), 6.31 (s, 1H), 2.29 (s, 3H), 1.34 – 1.08 (m, 13H). **^{13}C{^{1}H} NMR** (101 MHz, Chloroform-*d*) δ 178.5, 139.8, 135.8, 129.1, 125.4, 38.7, 34.7, 27.6, 21.0, 17.7. Spectroscopic data are in agreement with those previously reported.[4]

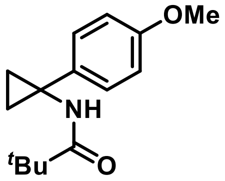

N-(1-(4-methoxyphenyl)cyclopropyl)pivalamide 1d

Eluent: petroleum ether/ethyl acetate (5:1). White solid (1.98 g, 80% yield). **^{1}H NMR** (400 MHz, Chloroform-*d*) δ 7.24 – 7.17 (m, 2H), 6.85 – 6.78 (m, 2H), 6.26 (s, 1H), 3.77 (s, 3H), 1.19 – 1.17 (m, 11H), 1.15 – 1.11 (m, 2H). **^{13}C{^{1}H} NMR** (101 MHz, Chloroform-*d*) δ 178.5, 158.2, 134.9, 127.3, 113.8, 55.4, 38.7, 34.6, 27.6, 17.1. Spectroscopic data are in agreement with those previously reported.[4]

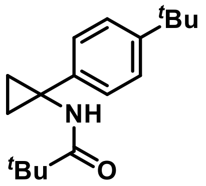

***N*-(1-(4-(*tert*-butyl)phenyl)cyclopropyl)pivalamide 1e**

Eluent: petroleum ether/ethyl acetate (5:1). White solid (2.05 g, 75% yield). **^{1}H NMR** (400 MHz, Chloroform-*d*) δ 7.33 – 7.27 (m, 2H), 7.15 – 7.06 (m, 2H), 6.27 (s, 1H), 1.33 – 1.19 (m, 22H). **^{13}C{^{1}H} NMR** (101 MHz, Chloroform-*d*) δ 178.6, 149.0, 139.7, 125.4, 124.8, 38.8, 34.5, 31.4, 27.7, 18.2. Spectroscopic data are in agreement with those previously reported.[9]

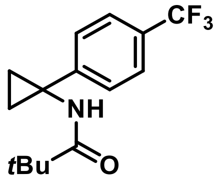

***N*-(1-(4-(trifluoromethyl)phenyl)pivalamide 1f**

Eluent: petroleum ether/ethyl acetate (5:1). White solid (2.36 g, 83% yield). **^{1}H NMR** (400 MHz, Chloroform-*d*) δ 7.52 – 7.50 (m, 2H), 7.28 – 7.22 (m, 2H), 6.41 (s, 1H), 1.32 – 1.25 (m, 4H), 1.21 (s, 9H). **^{13}C{^{1}H} NMR** (101 MHz, Chloroform-*d*) δ 178.8, 147.0, 128.4 (d, *J* = 32.6 Hz), 125.7, 125.4 (q, *J* = 3.8 Hz), 123.0, 38.7, 34.7, 27.6, 19.0. Spectroscopic data are in agreement with those previously reported.[9]

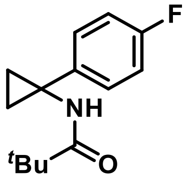

***N*-(1-(4-fluorophenyl)cyclopropyl)pivalamide 1g**

Eluent: petroleum ether/ethyl acetate (5:1). White solid (1.93 g, 83% yield). **^{1}H NMR** (400 MHz, Chloroform-*d*) δ 7.19 – 7.15 (m, 2H), 6.93 – 6.89 (m, 2H), 6.46 (s, 1H), 1.15 (s, 13H). **^{13}C{^{1}H}**

NMR (101 MHz, Chloroform-*d*) δ 178.6, 162.6, 160.2, 138.6, 138.5, 127.4, 127.3, 115.1, 114.9, 38.6, 34.4, 27.5, 17.6. Spectroscopic data are in agreement with those previously reported.[4]

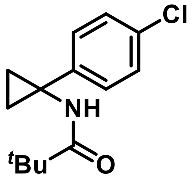

***N*-(1-(4-chlorophenyl)cyclopropyl)pivalamide 1h**

Eluent: petroleum ether/ethyl acetate (5:1). White solid (2.16 g, 86% yield). **^{1}H NMR** (400 MHz, Chloroform-*d*) δ 7.23 – 7.08 (m, 4H), 6.39 (s, 1H), 1.17 (s, 13H). **^{13}C{^{1}H} NMR** (101 MHz, Chloroform-*d*) δ 178.7, 141.4, 131.9, 128.4, 126.9, 38.7, 34.5, 27.6, 18.0. Spectroscopic data are in agreement with those previously reported.[4]

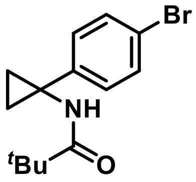

***N*-(1-(4-bromophenyl)cyclopropyl)pivalamide 1i**

Eluent: petroleum ether/ethyl acetate (5:1). White solid (2.36 g, 80% yield). **^{1}H NMR** (400 MHz, Chloroform-*d*) δ 7.41 – 7.34 (m, 2H), 7.11 – 7.05 (m, 2H), 6.28 (s, 1H), 1.22 (dt, *J* = 3.1, 1.4 Hz, 2H), 1.20 – 1.17 (m, 11H). **^{13}C{^{1}H} NMR** (101 MHz, Chloroform-*d*) δ 178.6, 141.9, 131.4, 127.4, 120.1, 38.7, 34.6, 27.6, 18.0. **HRMS** (ESI) m/z: $[M+Na]^+$ calcd for $C_{14}H_{18}BrNONa^+$: 318.0464; found: 318.0461.

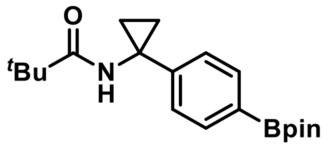

***N*-(1-(4-(4,4,5,5-tetramethyl-1,3,2-dioxaborolan-2-yl)phenyl)cyclopropyl)pivalamide 1j**

Eluent: petroleum ether/ethyl acetate (5:1). White solid (2.64 g, 77% yield). **^{1}H NMR** (400 MHz, Chloroform-*d*) δ 7.75 – 7.70 (m, 2H), 7.20 – 7.16 (m, 2H), 6.27 (s, 1H), 1.32 (s, 12H), 1.29 – 1.22 (m, 4H), 1.20 (s, 9H). **^{13}C{^{1}H} NMR** (101 MHz, Chloroform-*d*) δ 178.5, 146.1, 135.1, 124.5, 83.8, 38.8, 35.0, 27.7, 25.0, 18.5. **^{11}B NMR** (128 MHz, Chloroform-*d*) δ 31.05. Spectroscopic data are in agreement with those previously reported.[9]

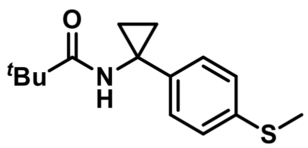

N-(1-(4-(methylthio)phenyl)cyclopropyl)pivalamide 1k

Eluent: petroleum ether/ethyl acetate (5:1). White solid (2.29 g, 87% yield). **^{1}H NMR** (400 MHz, Chloroform-*d*) δ 7.21 – 7.12 (m, 4H), 6.28 (s, 1H), 2.44 (s, 3H), 1.23 – 1.21 (m, 2H), 1.19 – 1.15 (m, 11H). **^{13}C{^{1}H} NMR** (101 MHz, Chloroform-*d*) δ 178.6, 140.0, 136.1, 127.2, 126.2, 38.7, 34.6, 27.6, 17.8, 16.3. Spectroscopic data are in agreement with those previously reported.[9]

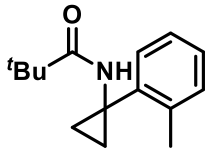

N-(1-(o-tolyl)cyclopropyl)pivalamide 1l

Eluent: petroleum ether/ethyl acetate (5:1). White solid (1.87 g, 81% yield). **^{1}H NMR** (400 MHz, Chloroform-*d*) δ 7.65 – 7.60 (m, 1H), 7.17 – 7.13 (m, 3H), 6.18 (s, 1H), 2.47 (s, 3H), 1.14 (s, 4H), 1.08 (s, 9H). **^{13}C{^{1}H} NMR** (101 MHz, Chloroform-*d*) δ 178.2, 139.4, 137.2, 131.0, 130.1, 127.4, 125.8, 38.7, 35.2, 27.6, 19.5, 14.8. **HRMS** (ESI) m/z: $[M+Na]^+$ calcd for $C_{15}H_{21}NONa^+$: 254.1515; found: 254.1514.

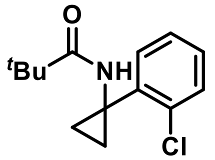

N-(1-(2-chlorophenyl)cyclopropyl)pivalamide 1m

Eluent: petroleum ether/ethyl acetate (5:1). White solid (2.16 g, 86% yield). **^{1}H NMR** (400 MHz, Chloroform-*d*) δ 7.65 (dd, *J* = 7.4, 2.0 Hz, 1H), 7.31 (dd, *J* = 7.5, 1.7 Hz, 1H), 7.23 – 7.13 (m, 2H), 6.60 (s, 1H), 1.16 (dt, *J* = 6.3, 2.3 Hz, 4H), 1.08 (s, 9H) **^{13}C{^{1}H} NMR** (101 MHz, Chloroform-*d*) δ 178.8, 138.8, 135.2, 132.2, 129.2, 128.7, 126.8, 38.6, 34.7, 27.4, 14.8. **HRMS** (ESI) m/z: $[M+Na]^+$ calcd for $C_{14}H_{18}NClONa^+$: 274.0969; found: 274.0969.

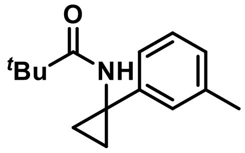

N-(1-(m-tolyl)cyclopropyl)pivalamide 1n

Eluent: petroleum ether/ethyl acetate (5:1). White solid (1.82 g, 79% yield). **^{1}H NMR** (400 MHz,

Chloroform-*d*) δ 7.18 – 7.14 (m, 1H), 7.01 – 6.97 (m, 3H), 6.30 (s, 1H), 2.31 (s, 3H), 1.37 – 1.07 (m, 13H). **^{13}C{^{1}H} NMR** (101 MHz, Chloroform-*d*) δ 178.5, 142.7, 137.9, 128.3, 127.0, 125.9, 122.3, 38.7, 34.7, 27.7, 21.6, 18.1. **HRMS** (ESI) m/z: [M+Na]$^+$ calcd for $C_{15}H_{21}NONa^+$: 254.1515; found: 254.1516.

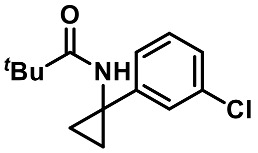

***N*-(1-(3-chlorophenyl)cyclopropyl)pivalamide 1o**

Eluent: petroleum ether/ethyl acetate (5:1). White solid (2.16 g, 86% yield). **^{1}H NMR** (400 MHz, Chloroform-*d*) δ 7.21 – 7.16 (m, 1H), 7.15 – 7.11 (m, 2H), 7.07 – 7.04 (m, 1H), 6.33 (s, 1H), 1.27 – 1.24 (m, 2H), 1.22 – 1.20 (m, 11H). **^{13}C{^{1}H} NMR** (101 MHz, Chloroform-*d*) δ 178.6, 145.0, 134.3, 129.7, 126.4, 125.5, 123.4, 38.7, 34.5, 27.6, 18.5. **HRMS** (ESI) m/z: [M+Na]$^+$ calcd for $C_{14}H_{18}NClONa^+$: 274.0969; found: 274.0967.

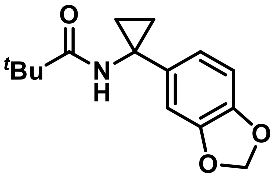

***N*-(1-(benzo[*d*][1,3]dioxol-5-yl)cyclopropyl)pivalamide 1p**

Eluent: petroleum ether/ethyl acetate (5:1). White solid (2.09 g, 80% yield). **^{1}H NMR** (400 MHz, Chloroform-*d*) δ 6.82 – 6.67 (m, 3H), 6.26 (s, 1H), 5.89 (s, 2H), 1.17 – 1.13 (m, 13H). **^{13}C{^{1}H} NMR** (101 MHz, Chloroform-*d*) δ 178.5, 147.7, 146.2, 136.9, 119.4, 108.0, 107.2, 101.0, 38.7, 35.1, 27.6, 17.3. **HRMS** (ESI) m/z: [M+Na]$^+$ calcd for $C_{15}H_{19}NO_3Na^+$: 284.1257; found: 284.1255.

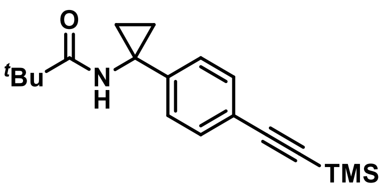

***N*-(1-(4-((trimethylsilyl)ethynyl)phenyl)cyclopropyl)pivalamide 1q**

Eluent: petroleum ether/ethyl acetate (5:1). White solid (2.34 g, 75% yield). **^{1}H NMR** (400 MHz, Chloroform-*d*) δ 7.38 – 7.33 (m, 2H), 7.13 – 7.07 (m, 2H), 6.25 (s, 1H), 1.27 – 1.25 (m, 2H), 1.23 – 1.21 (m, 2H), 1.20 (s, 9H), 0.23 (s, 9H). **^{13}C{^{1}H} NMR** (101 MHz, Chloroform-*d*) δ 178.6, 143.4, 132.1, 125.1, 121.0, 105.2, 93.9, 38.8, 34.8, 27.7, 18.5, 0.1. **HRMS** (ESI) m/z: [M+Na]$^+$

calcd for $C_{19}H_{27}NOSiNa^+$: 336.1754; found: 336.1753.

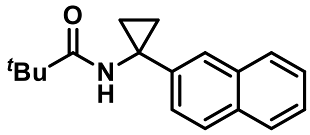

***N*-(1-(naphthalen-2-yl)cyclopropyl)pivalamide 1r**

Eluent: petroleum ether/ethyl acetate (5:1). White solid (2.24 g, 84% yield). **^{1}H NMR** (400 MHz, Chloroform-*d*) δ 7.80 – 7.73 (m, 3H), 7.68 (s, 1H), 7.46 – 7.39 (m, 2H), 7.34 (dd, *J* = 8.6, 2.0 Hz, 1H), 6.42 (s, 1H), 1.39 – 1.36 (m, 2H), 1.29 – 1.23 (m, 11H). **^{13}C{^{1}H} NMR** (101 MHz, Chloroform-*d*) δ 178.6, 140.2, 133.3, 132.2, 128.2, 127.9, 127.5, 126.1, 125.5, 124.2, 124.1, 38.8, 35.2, 27.7, 17.8. Spectroscopic data are in agreement with those previously reported.[4]

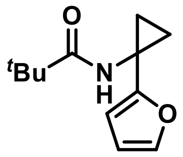

***N*-(1-(furan-2-yl)cyclopropyl)pivalamide 1s**

Eluent: petroleum ether/ethyl acetate (5:1). White solid (1.82g, 88% yield). **^{1}H NMR** (400 MHz, Chloroform-*d*) 7.22 (dd, *J* = 1.9, 0.9 Hz, 1H), 6.26 (dd, *J* = 3.2, 1.9 Hz, 1H), 6.06 (dd, *J* = 3.3, 0.9 Hz, 1H), 1.37 – 1.32 (m, 2H), 1.19 (s, 9H), 1.16 – 1.10 (m, 2H). **^{13}C{^{1}H} NMR** (101 MHz, Chloroform-*d*) δ 178.8, 155.4, 140.9, 110.6, 104.8, 38.8, 30.5, 27.6, 16.0. Spectroscopic data are in agreement with those previously reported.[9]

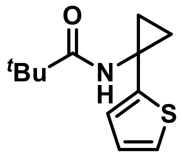

***N*-(1-(thiophen-2-yl)cyclopropyl)pivalamide 1t**

Eluent: petroleum ether/ethyl acetate (5:1). White solid (1.90 g, 85% yield). **^{1}H NMR** (400 MHz, Chloroform-*d*) δ 7.07 (dd, *J* = 5.1, 1.3 Hz, 1H), 6.86 (dd, *J* = 5.1, 3.5 Hz, 1H), 6.80 (dd, *J* = 3.6, 1.3 Hz, 1H), 6.36 (s, 1H), 1.31 – 1.28 (m, 2H), 1.27 – 1.24 (m, 2H), 1.18 (s, 9H). **^{13}C{^{1}H} NMR** (101 MHz, Chloroform-*d*) δ 178.6, 148.4, 126.7, 123.4, 122.9, 38.8, 32.0, 27.6, 18.9. Spectroscopic data are in agreement with those previously reported.[4]

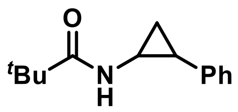

N-(2-phenylcyclopropyl)pivalamide 1u

Eluent: petroleum ether/ethyl acetate (5:1). White solid (1.93 g, 89% yield). **^{1}H NMR** (400 MHz, Chloroform-*d*) δ 7.29 – 7.21 (m, 2H), 7.18 – 7.14 (m, 3H), 5.93 (s, 1H), 2.87 (tt, *J* = 7.4, 3.2 Hz, 1H), 2.01 (ddd, *J* = 9.7, 6.2, 3.5 Hz, 1H), 1.25 – 1.08 (m, 11H). **^{13}C{^{1}H} NMR** (101 MHz, Chloroform-*d*) δ 179.7, 140.6, 128.4, 126.7, 126.2, 38.6, 32.2, 27.6, 24.9, 16.2. Spectroscopic data are in agreement with those previously reported.[4]

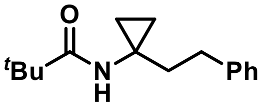

N-(1-phenethylcyclopropyl)pivalamide 1v

Eluent: petroleum ether/ethyl acetate (5:1). White solid (2.16 g, 88% yield). **^{1}H NMR** (400 MHz, Chloroform-*d*) δ 7.29 – 7.25 (m, 2H), 7.21 – 7.14 (m, 3H), 5.75 (s, 1H), 2.74 – 2.65 (m, 2H), 1.96 – 1.88 (m, 2H), 1.10 (s, 9H), 0.67 (dt, *J* = 6.7, 2.1 Hz, 4H). **^{13}C{^{1}H} NMR** (101 MHz, Chloroform-*d*) δ 178.7, 142.2, 128.5, 128.5, 125.9, 38.6, 37.6, 33.3, 33.1, 27.6, 13.9. **HRMS** (ESI) m/z: $[M+Na]^+$ calcd for $C_{16}H_{23}NONa^+$: 268.1672; found: 268.1672.

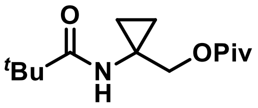

(1-pivalamidocyclopropyl)methyl pivalate 1w

Eluent: petroleum ether/ethyl acetate (5:1). White solid (2.11 g, 83% yield). **^{1}H NMR** (400 MHz, Chloroform-*d*) δ 5.95 (s, 1H), 4.10 (s, 2H), 1.18 (s, 9H), 1.13 (s, 9H), 0.86 – 0.76 (m, 4H). **^{13}C{^{1}H} NMR** (101 MHz, Chloroform-*d*) δ 179.0, 178.3, 67.9, 38.9, 38.6, 32.4, 27.5, 27.3, 12.2. Spectroscopic data are in agreement with those previously reported.[4]

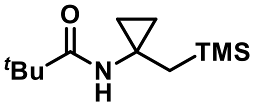

N-(1-((trimethylsilyl)methyl)cyclopropyl)pivalamide 1x

Eluent: petroleum ether/ethyl acetate (5:1). White solid (1.90 g, 84% yield). **^{1}H NMR** (400 MHz, Chloroform-*d*) δ 5.81 (s, 1H), 1.13 (s, 9H), 1.07 (s, 2H), 0.68 – 0.60 (m, 4H), 0.04 (s, 9H). **^{13}C{^{1}H} NMR** (101 MHz, Chloroform-*d*) δ 178.4, 38.6, 31.1, 27.6, 24.6, 15.8, -0.1.

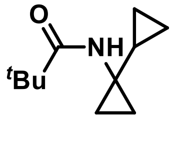

N-([1,1'-bi(cyclopropan)]-1-yl)pivalamide 1y

Eluent: petroleum ether/ethyl acetate (5:1). White solid (1.23 g, 68% yield). **^{1}H NMR** (400 MHz, Chloroform-*d*) δ 5.91 (s, 1H), 1.38 (tt, *J* = 8.3, 5.2 Hz, 1H), 1.15 (s, 9H), 0.67 – 0.55 (m, 4H), 0.45 – 0.31 (m, 2H), 0.17 – 0.07 (m, 2H). **^{13}C{^{1}H} NMR** (101 MHz, Chloroform-*d*) δ 178.7, 38.7, 34.1, 27.7, 15.3, 12.0, 2.8. **HRMS** (ESI) m/z: [M+Na]$^+$ calcd for $C_{11}H_{19}NONa^+$: 204.1359; found: 204.1359.

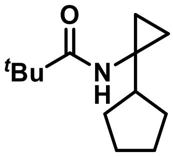

N-(1-cyclopentylcyclopropyl)pivalamide 1z

Eluent: petroleum ether/ethyl acetate (5:1). White solid (1.46g, 70% yield). **^{1}H NMR** (400 MHz, Chloroform-*d*) δ 5.87 (s, 1H), 2.14 (p, *J* = 9.0, 8.6 Hz, 1H), 1.62 – 1.38 (m, 6H), 1.14 (s, 11H), 0.67 (d, *J* = 16.4 Hz, 4H). **^{13}C{^{1}H} NMR** (101 MHz, Chloroform-*d*) δ 178.3, 44.7, 38.6, 34.9, 29.0, 27.6, 25.1, 11.8. **HRMS** (ESI) m/z: [M+Na]$^+$ calcd for $C_{13}H_{23}NONa^+$: 232.1672; found: 232.1671.

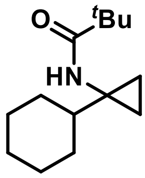

N-(1-cyclohexylcyclopropyl)pivalamide 1aa

Eluent: petroleum ether/ethyl acetate (5:1). White solid (1.56 g, 70% yield). **^{1}H NMR** (400 MHz, Chloroform-*d*) δ 5.80 (s, 1H), 1.79 – 1.70 (m, 4H), 1.65 – 1.61 (m, 3H), 1.15 (s, 9H), 1.14 – 0.93 (m, 4H), 0.67 (dt, *J* = 3.0, 1.9 Hz, 4H). **^{13}C{^{1}H} NMR** (101 MHz, Chloroform-*d*) δ 178.5, 44.4, 38.8, 36.9, 29.8, 27.8, 26.6, 26.5, 12.4. Spectroscopic data are in agreement with those previously reported.[4]

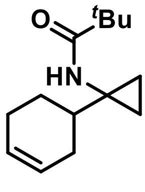

N-(1-(cyclohex-3-en-1-yl)cyclopropyl)pivalamide 1ab

Eluent: petroleum ether/ethyl acetate (5:1). White solid (1.70 g, 77% yield). **^{1}H NMR** (400 MHz,

Chloroform-*d*) δ 5.82 (s, 1H), 5.69 – 5.57 (m, 2H), 2.11 – 2.00 (m, 3H), 1.86 – 1.78 (m, 2H), 1.47 – 1.39 (m, 1H), 1.29 – 1.21 (m, 1H), 1.14 (s, 9H), 0.78 – 0.66 (m, 4H). **^{13}C{^{1}H} NMR** (101 MHz, Chloroform-*d*) δ 178.6, 126.9, 126.6, 40.7, 38.8, 36.3, 28.5, 27.9, 25.9, 25.9, 12.6, 12.4. **HRMS** (ESI) m/z: [M+Na]$^+$ calcd for $C_{14}H_{23}NONa^+$: 244.1672; found: 244.1667.

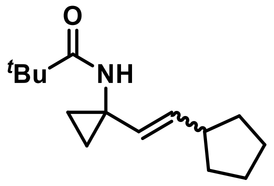

***N*-(1-(2-cyclopentylvinyl)cyclopropyl)pivalamide 1ac**

Eluent: petroleum ether/ethyl acetate (5:1). White solid (1.88 g, 80% yield). Mixture of (Z/E) isomers. **^{1}H NMR** (400 MHz, Chloroform-*d*) δ 6.03 – 5.95 (m, 1H), 5.63 – 5.01 (m, 2H), 3.17 – 2.31 (m, 1H), 1.82 – 1.49 (m, 6H), 1.26 – 1.17 (m, 11H), 0.99 – 0.86 (m, 4H). **^{13}C{^{1}H} NMR** (101 MHz, Chloroform-*d*) δ 178.3, 178.1, 138.7, 131.8, 130.0, 128.9, 42.8, 39.1, 38.8, 38.5, 33.8, 33.3, 33.2, 30.7, 27.8, 27.6, 25.5, 25.1, 16.2, 16.1. **HRMS** (ESI) m/z: [M+Na]$^+$ calcd for $C_{15}H_{25}NONa^+$: 258.1828; found: 258.1828.

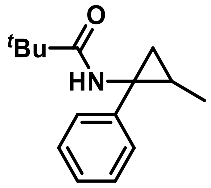

***N*-(2-methyl-1-phenylcyclopropyl)pivalamide 1ad**

Eluent: petroleum ether/ethyl acetate (5:1). White solid (1.82 g, 79% yield). Mixture of diastereomeric isomers. **^{1}H NMR** (400 MHz, Chloroform-*d*) δ 7.29 – 7.23 (m, 2H), 7.20 – 7.11 (m, 3H), 6.12 (s, 1H), 1.53 – 1.39 (m, 2H), 1.24 – 1.23 (m, 12H), 0.88 – 0.80 (m, 1H). **^{13}C{^{1}H} NMR** (101 MHz, Chloroform-*d*) δ 178.8, 143.8, 128.4, 126.1, 125.0, 39.1, 38.4, 27.9, 24.8, 23.0, 13.9. **HRMS** (ESI) m/z: [M+Na]$^+$ calcd for $C_{15}H_{21}NONa^+$: 254.1515; found: 254.1513.

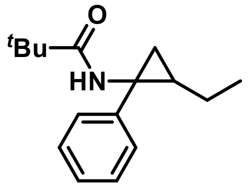

***N*-(2-ethyl-1-phenylcyclopropyl)pivalamide 1ae**

Eluent: petroleum ether/ethyl acetate (5:1). White solid (1.86 g, 76% yield). Mixture of

diastereomeric isomers. **^{1}H NMR** (400 MHz, Chloroform-*d*) δ 7.48 – 7.38 (m, 2H), 7.32 – 7.25 (m, 2H), 7.23 – 7.14 (m, 1H), 6.20 (d, *J* = 59.9 Hz, 1H), 1.46 – 1.27 (m, 3H), 1.12 (s, 9H), 1.07 – 1.02 (m, 1H), 0.94 – 0.86 (m, 3H), 0.84 – 0.72 (m, 1H). **^{13}C{^{1}H} NMR** (101 MHz, Chloroform-*d*) δ 178.3, 140.1, 129.1, 128.4, 128.2, 127.0, 126.1, 125.2, 40.1, 38.7, 30.4, 29.4, 27.7, 23.5, 23.2, 22.5, 18.8, 14.0, 13.3. **HRMS** (ESI) m/z: [M+Na]$^+$ calcd for $C_{16}H_{23}NONa^+$: 268.1672; found: 268.1668.

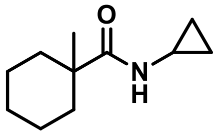

***N*-cyclopropyl-1-methylcyclohexane-1-carboxamide 1af**

Eluent: petroleum ether/ethyl acetate (5:1). White solid (1.59 g, 88% yield). **^{1}H NMR** (400 MHz, Chloroform-*d*) δ 5.73 (s, 1H), 2.73 – 2.66 (m, 1H), 1.86 – 1.79 (m, 2H), 1.57 – 1.23 (m, 9H), 1.09 (s, 3H), 0.81 – 0.70 (m, 2H), 0.48 – 0.37 (m, 2H). **^{13}C{^{1}H} NMR** (101 MHz, Chloroform-*d*) δ 179.2, 42.5, 35.7, 25.9, 22.9, 22.7, 6.8. Spectroscopic data are in agreement with those previously reported.[4]

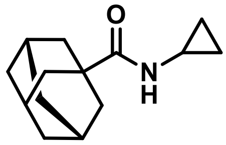

(3*r*,5*r*,7*r*)-*N*-cyclopropyladamantane-1-carboxamide 1ag

Eluent: petroleum ether/ethyl acetate (5:1). White solid (1.97 g, 90% yield). **^{1}H NMR** (400 MHz, Chloroform-*d*) δ 5.67 (s, 1H), 2.71 – 2.65 (m, 1H), 2.05 – 1.97 (m, 3H), 1.80 – 1,79 (m, 6H), 1.75 – 1.61 (m, 6H), 0.76 – 0.71 (m, 2H), 0.46 – 0.38 (m, 2H). **^{13}C{^{1}H} NMR** (101 MHz, Chloroform-*d*) δ 179.4, 40.5, 39.3, 36.6, 28.2, 22.6, 6.7. Spectroscopic data are in agreement with those previously reported.[4]

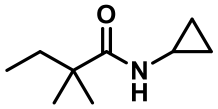

***N*-cyclopropyl-2,2-dimethylbutanamide 1ah**

Eluent: petroleum ether/ethyl acetate (5:1). White solid (1.31g, 85 yield). **^{1}H NMR** (400 MHz, Chloroform-*d*) δ 5.73 (s, 1H), 2.76 – 2.65 (m, 1H), 1.52 (q, *J* = 7.4 Hz, 2H), 1.12 (s, 6H), 0.82 (td, *J* = 7.5, 0.9 Hz, 3H), 0.79 – 0.73 (m, 2H), 0.51 – 0.42 (m, 2H). **^{13}C{^{1}H} NMR** (101 MHz,

Chloroform-*d*) δ 179.3, 42.2, 34.0, 25.0, 22.8, 9.2, 6.8. Spectroscopic data are in agreement with those previously reported.[10]

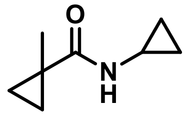

***N*-cyclopropyl-1-methylcyclopropane-1-carboxamide 1ai**

Eluent: petroleum ether/ethyl acetate (5:1). White solid (1.18 g, 85% yield). **^{1}H NMR** (400 MHz, Chloroform-*d*) δ 5.84 (s, 1H), 2.75 – 2.64 (m, 1H), 1.25 (s, 3H), 1.18 (q, *J* = 4.0 Hz, 2H), 0.77 – 0.72 (m, 2H), 0.55 – 0.51 (m, 2H), 0.51 – 0.46 (m, 2H). **^{13}C{^{1}H} NMR** (101 MHz, Chloroform-*d*) δ 176.4, 23.1, 19.8, 18.9, 16.1, 6.7. Spectroscopic data are in agreement with those previously reported.[10]

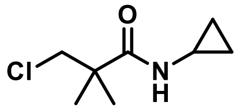

3-chloro-*N*-cyclopropyl-2,2-dimethylpropanamide 1aj

Eluent: petroleum ether/ethyl acetate (5:1). White solid (1.50 g, 86% yield). **^{1}H NMR** (400 MHz, Chloroform-*d*) δ 6.06 (s, 1H), 3.55 (s, 1H), 2.66 (tqd, *J* = 7.1, 3.8, 1.4 Hz, 1H), 1.20 (s, 2H), 0.73 – 0.69 (m, 2H), 0.49 – 0.41 (m, 2H). **^{13}C{^{1}H} NMR** (101 MHz, Chloroform-*d*) δ 176.2, 52.8, 43.9, 23.4, 22.9, 6.6. **HRMS** (ESI) m/z: [M+Na]$^+$ calcd for $C_8H_{14}NClONa^+$: 198.0656; found: 198.0653.

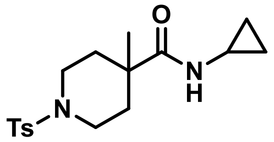

***N*-cyclopropyl-4-methyl-1-tosylpiperidine-4-carboxamide 1ak**

Eluent: petroleum ether/ethyl acetate (3:1). White solid (2.96 g, 88% yield). **^{1}H NMR** (400 MHz, Chloroform-*d*) δ 7.59 (d, *J* = 8.3 Hz, 2H), 7.29 (d, *J* = 8.0 Hz, 3H), 5.69 (s, 1H), 3.35 – 3.26 (m, 2H), 2.68 (ddd, *J* = 12.3, 7.5, 2.7 Hz, 2H), 2.58 (qt, *J* = 7.0, 3.2 Hz, 1H), 2.42 (s, 3H), 2.01 (dddd, *J* = 13.6, 5.0, 3.1, 1.4 Hz, 2H), 1.55 (ddd, *J* = 13.8, 10.1, 3.7 Hz, 2H), 1.08 (s, 3H), 0.77 – 0.65 (m, 2H), 0.39 – 0.27 (m, 2H). **^{13}C{^{1}H} NMR** (101 MHz, Chloroform-*d*) δ 177.1, 143.7, 133.2, 129.8, 127.7, 43.5, 40.3, 34.6, 26.1, 22.8, 21.6, 6.8. **HRMS** (ESI) m/z: [M+Na]$^+$ calcd for $C_{17}H_{24}N_2O_3SNa^+$: 359.1400; found: 359.1396.

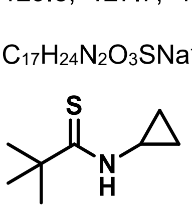

N-cyclopropyl-2,2-dimethylpropanethioamide 1al

Eluent: petroleum ether/ethyl acetate (5:1). Yellow oil (1.25 g, 80% yield). **^{1}H NMR** (400 MHz, Chloroform-*d*) δ 7.36 (s, 1H), 3.14 – 3.07 (m, 1H), 1.21 (s, 9H), 0.85 – 0.78 (m, 2H), 0.60 – 0.54 (m, 2H). **^{13}C {^{1}H} NMR** (101 MHz, Chloroform-*d*) δ 214.8, 43.9, 29.9, 29.1, 7.2. **HRMS** (ESI) m/z: $[M+H]^+$ calcd for $C_8H_{16}NS^+$: 158.0998; found: 158.0999.

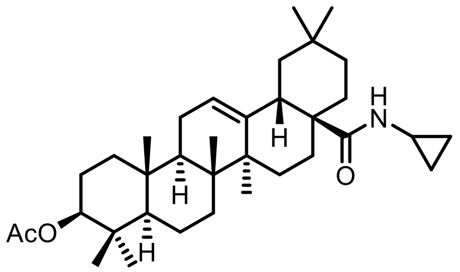

(3*S*,4a*R*,6a*R*,6b*S*,8a*S*,12a*S*,14a*R*,14b*R*)-8a-(cyclopropylcarbamoyl)-4,4,6a,6b,11,11,14b-heptamethyl-1,2,3,4,4a,5,6,6a,6b,7,8,8a,9,10,11,12,12a,14,14a,14b-icosahydropicen-3-yl acetate 1am

Eluent: petroleum ether/ethyl acetate (2:1). White solid (3.22 g, 60% yield). **^{1}H NMR** (400 MHz, Chloroform-*d*) δ 6.00 (d, *J* = 2.8 Hz, 1H), 5.34 (t, *J* = 3.6 Hz, 1H), 4.54 – 4.45 (m, 1H), 2.68 – 2.58 (m, 1H), 2.43 (dd, *J* = 13.2, 4.4 Hz, 1H), 2.05 (s, 3H), 1.91 (dd, *J* = 7.6, 3.6 Hz, 2H), 1.79 – 1.64 (m, 4H), 1.58 – 1.42 (m, 7H), 1.40 – 1.25 (m, 4H), 1.22 – 1.13 (m, 5H), 1.08 – 1.00 (m, 2H), 0.94 (s, 3H), 0.89 (d, *J* = 3.9 Hz, 6H), 0.86 (d, *J* = 4.0 Hz, 7H), 0.79 (s, 3H), 0.76 – 0.66 (m, 2H), 0.47 – 0.35 (m, 2H). **^{13}C{^{1}H} NMR** (101 MHz, Chloroform-*d*) δ 179.9, 171.2, 145.1, 122.8, 81.0, 55.3, 47.6, 46.9, 46.2, 42.4, 42.2, 39.6, 38.3, 37.8, 37.0, 34.2, 33.1, 32.5, 32.5, 30.8, 28.2, 27.4, 25.8, 23.9, 23.7, 23.7, 22.8, 21.4, 18.3, 17.0, 16.8, 15.6, 6.8, 6.1. **HRMS** (ESI) m/z: $[M+Na]^+$ calcd for $C_{35}H_{55}NO_3Na^+$: 560.4074; found: 560.4078.

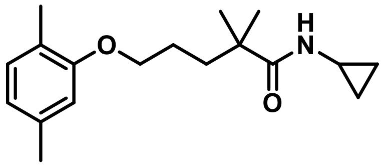

N-cyclopropyl-5-(2,5-dimethylphenoxy)-2,2-dimethylpentanamide 1an

Eluent: petroleum ether/ethyl acetate (5:1). White solid (2.31g, 80% yield). **^{1}H NMR** (400 MHz, Chloroform-*d*) δ 7.00 – 6.99 (m, 1H), 6.83 – 6.43 (m, 2H), 5.79 (s, 1H), 3.91 – 3.90 (m, 2H), 2.70 (s, 1H), 2.30 (s, 3H), 2.17 (s, 3H), 1.70 (q, *J* = 12.0, 10.6 Hz, 4H), 1.18 (s, 6H), 0.84 – 0.70 (m, 2H), 0.45 (s, 2H). **^{13}C{^{1}H} NMR** (101 MHz, Chloroform-*d*) δ 178.9, 157.0, 136.6, 130.4,

123.6, 120.8, 112.2, 68.0, 41.7, 37.6, 25.5, 25.1, 22.8, 21.4, 15.9, 6.8. **HRMS** (ESI) m/z: $[M+Na]^+$ calcd for $C_{18}H_{27}NO_2Na^+$: 312.1934; found: 312.1931.

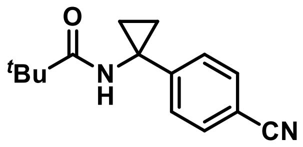

***N*-(1-(4-cyanophenyl)cyclopropyl)pivalamide 1ao**

Eluent: petroleum ether/ethyl acetate (5:1). White solid (2.13 g, 88% yield). **^{1}H NMR** (400 MHz, Chloroform-*d*) δ 7.58 – 7.51 (m, 2H), 7.24 – 7.20 (m, 2H), 6.34 (s, 1H), 1.33 (dd, *J* = 2.8, 1.6 Hz, 2H), 1.30 (dd, *J* = 2.9, 1.7 Hz, 2H), 1.21 (s, 9H). **^{13}C{^{1}H} NMR** (101 MHz, Chloroform-*d*) δ 178.9, 148.7, 132.3, 125.6, 119.1, 109.8, 38.8, 34.8, 19.7. Spectroscopic data are in agreement with those previously reported.[4]

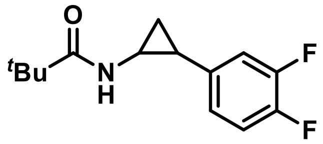

***N*-(2-(3,4-difluorophenyl)cyclopropyl)pivalamide 1ap**

Eluent: petroleum ether/ethyl acetate (5:1). White solid (2.17 g, 86% yield). **^{1}H NMR** (400 MHz, Chloroform-*d*) δ 7.06 – 6.89 (m, 3H), 5.96 (s, 1H), 2.77 – 2.68 (m, 1H), 1.95 (ddd, *J* = 9.6, 6.5, 3.4 Hz, 1H), 1.18 (s, 9H), 1.15 – 1.09 (m, 2H). **^{13}C{^{1}H} NMR** (101 MHz, Chloroform-*d*) δ 180.0, 151.5, 151.4, 150.3, 150.2, 149.0, 148.9, 147.9, 147.7, 137.8, 137.7, 123.2, 123.1, 123.1, 123.1, 117.1, 116.9, 116.2, 116.0, 38.6, 32.1, 27.6, 24.6, 15.4. **^{19}F NMR** (376 MHz, Chloroform-*d*) δ -138.35 – -138.46 (m), -141.21 – -142.22 (m). Spectroscopic data are in agreement with those previously reported.[4]

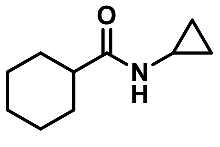

***N*-cyclopropylcyclohexanecarboxamide 1aq**

Eluent: petroleum ether/ethyl acetate (5:1). White solid (1.43 g, 86% yield). **^{1}H NMR** (400 MHz, Chloroform-*d*) δ 5.59 (s, 1H), 2.69 (tq, *J* = 7.1, 3.7 Hz, 1H), 2.00 (tt, *J* = 11.8, 3.4 Hz, 1H), 1.86 – 1.72 (m, 5H), 1.65 (ddq, *J* = 6.9, 3.8, 2.0 Hz, 1H), 1.47 – 1.32 (m, 2H), 1.31 – 1.16 (m, 2H), 0.80 – 0.70 (m, 2H), 0.49 – 0.40 (m, 2H). **^{13}C{^{1}H} NMR** (101 MHz, Chloroform-*d*) δ 177.6, 45.5,

29.8, 25.8, 22.6, 6.8. Spectroscopic data are in agreement with those previously reported.[10]

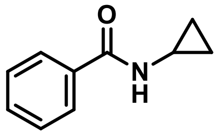

***N*-cyclopropylbenzamide 1ar**

Eluent: petroleum ether/ethyl acetate (5:1). White solid (1.43 g, 89% yield). **^{1}H NMR** (400 MHz, Chloroform-*d*) δ 7.76 – 7.70 (m, 2H), 7.51 – 7.45 (m, 1H), 7.43 – 7.37 (m, 2H), 6.33 (s, 1H), 2.90 (tq, *J* = 7.1, 3.6 Hz, 1H), 0.89 – 0.83 (m, 2H), 0.65 – 0.56 (m, 2H). **^{13}C{^{1}H} NMR** (101 MHz, Chloroform-*d*) δ 169.1, 134.6, 131.6, 128.7, 127.0, 23.3, 6.9. Spectroscopic data are in agreement with those previously reported.[10]

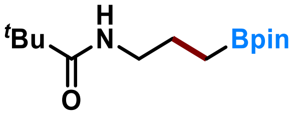

***N*-(3-(4,4,5,5-tetramethyl-1,3,2-dioxaborolan-2-yl)propyl)pivalamide 2a**

Eluent: dichloromethane/ethyl acetate (15:1). White solid (44.1 mg, 82% yield). **^{1}H NMR** (400 MHz, Chloroform-*d*) δ 5.77 (s, 1H), 3.20 (td, *J* = 7.2, 5.5 Hz, 2H), 1.65 – 1.53 (m, 2H), 1.23 (s, 12H), 1.17 (s, 9H), 0.78 (t, *J* = 7.9 Hz, 2H). **^{13}C{^{1}H} NMR** (101 MHz, Chloroform-*d*) δ 178.4, 83.3, 41.7, 38.7, 27.7, 24.9, 23.9. **^{11}B NMR** (128 MHz, Chloroform-*d*) δ 34.01. Spectroscopic data are in agreement with those previously reported.[3,4]

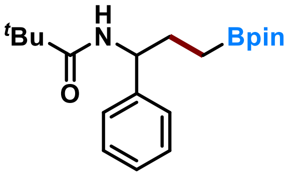

***N*-(1-phenyl-3-(4,4,5,5-tetramethyl-1,3,2-dioxaborolan-2-yl)propyl)pivalamide 2b**

Eluent: dichloromethane/ethyl acetate (15:1). White solid (53.1 mg, 77% yield). **^{1}H NMR** (400 MHz, Chloroform-*d*) δ 7.33 – 7.27 (m, 2H), 7.25 – 7.19 (m, 3H), 5.99 (d, *J* = 7.8 Hz, 1H), 4.85 (q, *J* = 7.4 Hz, 1H), 1.94 – 1.82 (m, 2H), 1.22 (s, 12H), 1.19 (s, 9H), 0.86 – 0.65 (m, 2H). **^{13}C{^{1}H} NMR** (101 MHz, Chloroform- *d*) δ 177.7, 142.9, 128.6, 127.1, 126.5, 83.3, 55.2, 38.8, 30.8, 27.7, 25.0, 24.9. **^{11}B NMR** (128 MHz, Chloroform-*d*) δ 34.44. Spectroscopic data are in agreement with those previously reported.[3,4]

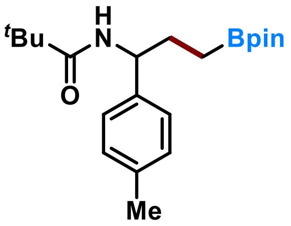

N -(3-(4,4,5,5-tetramethyl-1,3,2-dioxaborolan-2-yl)-1-(p-tolyl)propyl)pivalamide 2c

Eluent: dichloromethane/ethyl acetate (15:1). White solid (46.7 mg, 65% yield). **^{1}H NMR** (400 MHz, Chloroform-*d*) δ 7.16 – 7.04 (m, 4H), 5.95 (d, *J* = 7.8 Hz, 1H), 4.81 (q, *J* = 7.4 Hz, 1H), 2.30 (s, 3H), 1.87 – 1.78 (m, 2H), 1.22 (s, 12H), 1.18 (s, 9H), 0.88 – 0.62 (m, 2H). **^{13}C{^{1}H} NMR** (101 MHz, Chloroform-*d*) δ 177.6, 139.8, 136.6, 129.3, 126.4, 83.3, 54.9, 38.7, 30.7, 27.7, 25.0, 24.9, 21.1. **^{11}B NMR** (128 MHz, Chloroform-*d*) δ 33.78. Spectroscopic data are in agreement with those previously reported.[3,4]

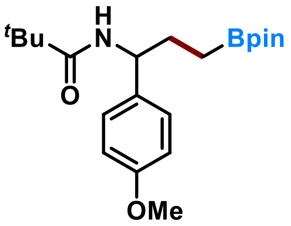

***N*-(1-(4-methoxyphenyl)-3-(4,4,5,5-tetramethyl-1,3,2-dioxaborolan-2-yl)propyl)pivalamide 2d**

Eluent: dichloromethane/ethyl acetate (15:1). White solid (57.7 mg, 77% yield). **^{1}H NMR** (400 MHz, Chloroform-*d*) δ 7.18 – 7.13 (m, 2H), 6.86 – 6.81 (m, 2H), 5.92 (d, *J* = 7.8 Hz, 1H), 4.78 (q, *J* = 7.4 Hz, 1H), 3.76 (s, 3H), 1.83 (tq, *J* = 9.3, 6.9 Hz, 2H), 1.21 (s, 12H), 1.17 (s, 9H), 0.81 – 0.63 (m, 2H). **^{13}C{^{1}H} NMR** (101 MHz, Chloroform-*d*) δ 177.6, 158.7, 135.0, 127.6, 114.0, 83.3, 55.3, 54.6, 38.7, 30.7, 27.7, 25.0, 24.9. **^{11}B NMR** (128 MHz, Chloroform-*d*) δ 34.42. Spectroscopic data are in agreement with those previously reported.[3,4]

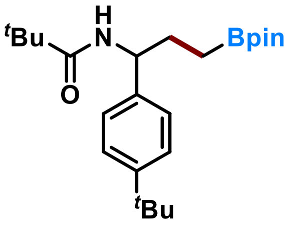

***N*-(1-(4-(*tert*-butyl)phenyl)-3-(4,4,5,5-tetramethyl-1,3,2-dioxaborolan-2-yl)propyl)pivalamide 2e**

Eluent: dichloromethane/ethyl acetate (15:1). White solid (60.9 mg, 76% yield). **^{1}H NMR** (400 MHz, Chloroform-*d*) δ 7.46 – 7.28 (m, 2H), 7.18 – 7.13 (m, 2H), 5.96 (d, *J* = 7.9 Hz, 1H), 4.83 (q, *J* = 7.4 Hz, 1H), 1.92 – 1.78 (m, 2H), 1.28 (s, 9H), 1.21 (s, 12H), 1.19 (s, 9H), 0.83 – 0.65 (m, 2H). **^{13}C{^{1}H} NMR** (101 MHz, Chloroform-*d*) δ 177.6, 149.8, 139.6, 126.1, 125.4, 83.2, 54.8, 38.7, 34.5, 31.4, 30.7, 27.7, 24.9, 24.8. **^{11}B NMR** (128 MHz, Chloroform-*d*) δ 33.67. **HRMS** (ESI) m/z: $[M+Na]^+$ calcd for $C_{24}H_{40}BNO_3Na^+$: 424.2993; found: 424.3001.

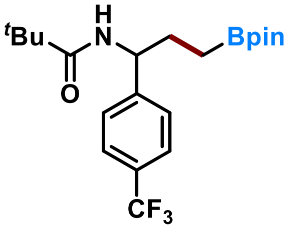

***N*-(3-(4,4,5,5-tetramethyl-1,3,2-dioxaborolan-2-yl)-1-(4-(trifluoromethyl)phenyl)propyl)pivalamide 2f**

Eluent: dichloromethane/ethyl acetate (15:1). White solid (33.0 mg, 40% yield). **^{1}H NMR** (400 MHz, Chloroform-*d*) δ 7.55 (d, *J* = 8.1 Hz, 2H), 7.34 (d, *J* = 8.0 Hz, 2H), 6.10 (d, *J* = 7.2 Hz, 1H), 4.86 (q, *J* = 7.3 Hz, 1H), 1.90 – 1.82 (m, 2H), 1.23 (s, 12H), 1.20 (s, 9H), 0.86 – 0.68 (m, 2H). **^{13}C{^{1}H} NMR** (101 MHz, Chloroform-*d*) δ 178.0, 147.2, 126.7, 125.6 (q, *J* = 3.9 Hz), 83.5, 55.1, 38.8, 30.6, 27.7, 25.0, 24.8. **^{11}B NMR** (128 MHz, Chloroform-*d*) δ 34.15. **^{19}F NMR** (376 MHz, Chloroform-*d*) δ -62.46. **HRMS** (ESI) m/z: $[M+Na]^+$ calcd for $C_{21}H_{31}BF_3NO_3Na^+$: 436.2241; found: 436.2248.

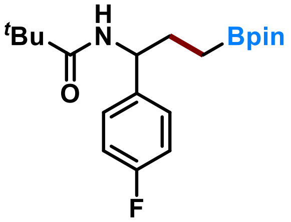

***N*-(1-(4-fluorophenyl)-3-(4,4,5,5-tetramethyl-1,3,2-dioxaborolan-2-yl)propyl)pivalamide 2g**

Eluent: dichloromethane/ethyl acetate (15:1). White solid (29.7 mg, 41% yield). **^{1}H NMR** (400 MHz, Chloroform-*d*) δ 7.22 – 7.17 (m, 2H), 7.02 – 6.93 (m, 2H), 5.97 (d, *J* = 7.6 Hz, 1H), 4.81 (q, *J* = 7.4 Hz, 1H), 1.84 (q, *J* = 7.8 Hz, 2H), 1.23 (s, 12H), 1.18 (s, 9H), 0.83 – 0.66 (m, 2H). **^{13}C{^{1}H} NMR** (101 MHz, Chloroform-*d*) δ 177.8, 160.7, 138.8 (d, *J* = 3.2 Hz), 128.0 (d, *J* = 8.0

Hz), 115.4 (d, J = 21.4 Hz), 83.4, 54.7, 38.7, 30.7, 27.7, 25.0, 24.9. **^{11}B NMR** (128 MHz, Chloroform-*d*) δ 34.39. **^{19}F NMR** (376 MHz, Chloroform-d) δ -116.06 (td, J = 9.0, 4.5 Hz). Spectroscopic data are in agreement with those previously reported.[3,4]

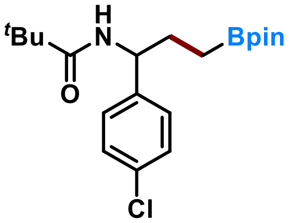

***N*-(1-(4-chlorophenyl)-3-(4,4,5,5-tetramethyl-1,3,2-dioxaborolan-2-yl)propyl)pivalamide 2h**

Eluent: dichloromethane/ethyl acetate (15:1). White solid (34.9 mg, 46% yield). **^{1}H NMR** (400 MHz, Chloroform-*d*) δ 7.28 – 7.25 (m, 2H), 7.20 – 7.16 (m, 2H), 6.01 (d, J = 7.5 Hz, 1H), 4.80 (q, J = 7.3 Hz, 1H), 1.89 – 1.79 (m, 2H), 1.23 (s, 12H), 1.19 (s, 9H), 0.83 – 0.67 (m, 2H). **^{13}C{^{1}H} NMR** (101 MHz, Chloroform-*d*) δ 177.8, 141.6, 132.7, 128.7, 127.8, 83.4, 54.7, 38.7, 30.6, 27.7, 25.0, 24.9. **^{11}B NMR** (128 MHz, Chloroform-*d*) δ 34.08. Spectroscopic data are in agreement with those previously reported.[3,4]

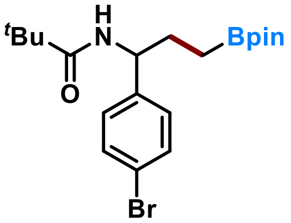

***N*-(1-(4-bromophenyl)-3-(4,4,5,5-tetramethyl-1,3,2-dioxaborolan-2-yl)propyl)pivalamide 2i**

Eluent: dichloromethane/ethyl acetate (15:1). White solid (55.0 mg, 65% yield). **^{1}H NMR** (400 MHz, Chloroform-*d*) δ 7.42 – 7.38 (m, 2H), 7.13 – 7.07 (m, 2H), 6.01 (d, J = 7.5 Hz, 1H), 4.77 (q, J = 7.3 Hz, 1H), 1.82 (h, J = 7.1, 6.7 Hz, 2H), 1.22 (s, 12H), 1.17 (s, 9H), 0.82 – 0.64 (m, 2H). **^{13}C{^{1}H} NMR** (101 MHz, Chloroform-*d*) δ 177.8, 142.1, 131.6, 128.2, 120.8, 83.4, 54.8, 38.7, 30.5, 27.7, 24.9, 24.8. **^{11}B NMR** (128 MHz, Chloroform-*d*) δ 33.47. **HRMS** (ESI) m/z: $[M+Na]^+$ calcd for $C_{20}H_{31}BBrNO_3Na^+$: 446.1473; found: 446.1482.

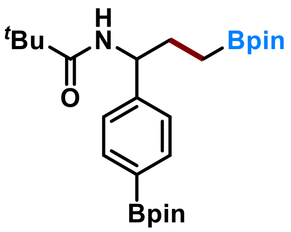

***N*-(3-(4,4,5,5-tetramethyl-1,3,2-dioxaborolan-2-yl)-1-(4-(4,4,5,5-tetramethyl-1,3,2-dioxaborolan-2-yl)phenyl)propyl)pivalamide 2j**

Eluent: dichloromethane/ethyl acetate (10:1). Colorless oil (50.9 mg, 54% yield). **^{1}H NMR** (400 MHz, Chloroform-*d*) δ 7.76 – 7.73 (m, 2H), 7.25 – 7.22 (m, 2H), 6.01 (d, *J* = 7.8 Hz, 1H), 4.85 (q, *J* = 7.4 Hz, 1H), 1.87 – 1.81 (m, 2H), 1.32 (s, 12H), 1.21 (s, 12H), 1.17 (s, 9H), 0.79 – 0.66 (m, 2H). **^{13}C{^{1}H} NMR** (101 MHz, Chloroform-*d*) δ 177.7, 146.1, 135.2, 125.9, 83.8, 83.3, 55.3, 38.7, 30.6, 27.7, 24.9, 24.8. **^{11}B NMR** (128 MHz, Chloroform-*d*) δ 33.23, 30.69. **HRMS** (ESI) m/z: $[M+Na]^+$ calcd for $C_{26}H_{43}B_2NO_5Na^+$: 494.3220; found: 494.3222.

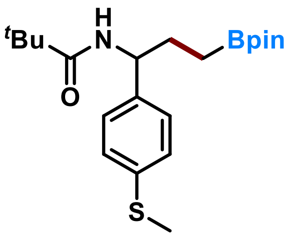

***N*-(1-(4-(methylthio)phenyl)-3-(4,4,5,5-tetramethyl-1,3,2-dioxaborolan-2-yl)propyl)pivalamide 2k**

Eluent: dichloromethane/ethyl acetate (15:1). White solid (43.0 mg, 55% yield). **^{1}H NMR** (400 MHz, Chloroform-*d*) δ 7.20 – 7.13 (m, 4H), 5.97 (d, *J* = 7.7 Hz, 1H), 4.79 (q, *J* = 7.4 Hz, 1H), 2.44 (s, 3H), 1.86 – 1.79 (m, 2H), 1.21 (s, 12H), 1.17 (s, 9H), 0.81- 0.85 (m, 2H). **^{13}C{^{1}H} NMR** (101 MHz, Chloroform-*d*) δ 177.7, 139.9, 136.9, 127.0, 83.3, 54.8, 38.7, 30.6, 27.7, 24.9, 24.8, 16.1. **^{11}B NMR** (128 MHz, Chloroform-*d*) δ 33.99. **HRMS** (ESI) m/z: $[M+Na]^+$ calcd for $C_{21}H_{34}BNSO_3Na^+$: 414.2245; found: 414.2252.

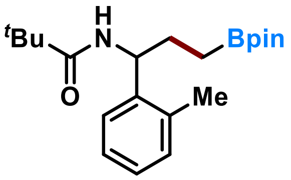

N-(3-(4,4,5,5-tetramethyl-1,3,2-dioxaborolan-2-yl)-1-(o-tolyl)propyl)pivalamide 2l

Eluent: dichloromethane/ethyl acetate (15:1). White solid (25.8 mg, 36% yield). **^{1}H NMR** (400 MHz, Chloroform-*d*) δ 7.18 – 7.08 (m, 4H), 5.97 (d, *J* = 7.8 Hz, 1H), 5.08 (q, *J* = 7.4 Hz, 1H), 2.41 (s, 3H), 1.86 – 1.75 (m, 2H), 1.23 (d, J = 1.6 Hz, 12H), 1.18 (s, 9H), 0.90 – 0.65 (m, 2H). **^{13}C{^{1}H} NMR** (101 MHz, Chloroform-*d*) δ 177.6, 141.3, 136.2, 130.7, 126.9, 126.2, 124.7, 83.4, 51.3, 38.7, 30.3, 27.7, 25.0, 24.9, 19.4. **^{11}B NMR** (128 MHz, Chloroform-*d*) δ 33.93. **HRMS** (ESI) m/z: $[M+Na]^+$ calcd for $C_{21}H_{34}BNO_3Na^+$: 382.2524; found: 382.2530.

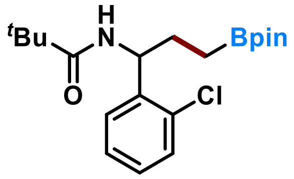

N-(1-(2-chlorophenyl)-3-(4,4,5,5-tetramethyl-1,3,2-dioxaborolan-2-yl)propyl)pivalamide 2m

Eluent: dichloromethane/ethyl acetate (15:1). White solid (34.1 mg, 45% yield). **^{1}H NMR** (400 MHz, Chloroform-*d*) δ 7.33 – 7.29 (m, 1H), 7.21 – 7.13 (m, 3H), 6.37 (d, *J* = 7.7 Hz, 1H), 5.15 (td, *J* = 7.9, 6.4 Hz, 1H), 1.97 – 1.85 (m, 2H), 1.23 (s, 12H), 1.19 (s, 9H), 0.84 (ddd, *J* = 16.1, 10.1, 5.8 Hz, 1H), 0.72 (ddd, *J* = 16.4, 10.1, 6.6 Hz, 1H). **^{13}C{^{1}H} NMR** (101 MHz, Chloroform-*d*) δ 177.7, 140.0, 132.9, 130.2, 128.2, 126.9, 83.4, 53.8, 38.8, 29.1, 27.7, 25.0, 24.9. **^{11}B NMR** (128 MHz, Chloroform-*d*) δ 34.30. **HRMS** (ESI) m/z: $[M+Na]^+$ calcd for $C_{20}H_{31}BClNO_3Na^+$: 402.1978; found: 402.1986.

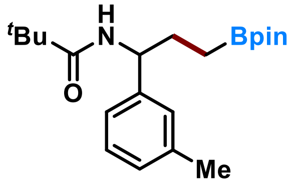

N-(3-(4,4,5,5-tetramethyl-1,3,2-dioxaborolan-2-yl)-1-(m-tolyl)propyl)pivalamide 2n

Eluent: dichloromethane/ethyl acetate (15:1). White solid (41.6 mg, 58% yield). **^{1}H NMR** (400 MHz, Chloroform-*d*) δ 7.18 (t, *J* = 7.6 Hz, 1H), 7.02 (d, *J* = 7.8 Hz, 3H), 5.97 (d, *J* = 7.9 Hz, 1H), 4.81 (q, *J* = 7.4 Hz, 1H), 2.31 (s, 3H), 1.94 – 1.78 (m, 2H), 1.22 (s, 12H), 1.19 (s, 10H), 0.86 – 0.63 (m, 2H). **^{13}C{^{1}H} NMR** (101 MHz, Chloroform-*d*) δ 178.1, 143.2, 138.5, 128.9, 128.3, 127.8, 123.7, 83.7, 55.6, 39.2, 31.2, 28.1, 25.4, 25.3, 22.0. **^{11}B NMR** (128 MHz, Chloroform-*d*) δ 33.65. **HRMS** (ESI) m/z: $[M+Na]^+$ calcd for $C_{21}H_{34}BNO_3Na^+$: 382.2524; found: 382.2523.

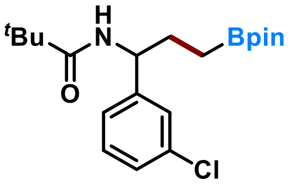

***N*-(1-(3-chlorophenyl)-3-(4,4,5,5-tetramethyl-1,3,2-dioxaborolan-2-yl)propyl)pivalamide 2o**

Eluent: dichloromethane/ethyl acetate (15:1). White solid (34.8 mg, 46% yield). **^{1}H NMR** (400 MHz, Chloroform-*d*) δ 7.24 – 7.16 (m, 3H), 7.12 – 7.10 (m, 1H), 6.04 (d, *J* = 7.6 Hz, 1H), 4.81 (q, *J* = 7.3 Hz, 1H), 1.83 (tq, *J* = 9.2, 6.9 Hz, 2H), 1.23 (s, 12H), 1.19 (s, 9H), 0.82 – 0.66 (m, 2H). **^{13}C{^{1}H} NMR** (101 MHz, Chloroform-*d*) δ 177.9, 145.2, 134.4, 129.8, 127.2, 126.5, 124.8, 83.5, 54.8, 38.7, 30.6, 27.7, 25.0, 24.8. **^{11}B NMR** (128 MHz, Chloroform-*d*) δ 33.26. **HRMS** (ESI) m/z: $[M+Na]^+$ calcd for $C_{20}H_{31}BClNO_3Na^+$: 402.1978; found: 402.1978.

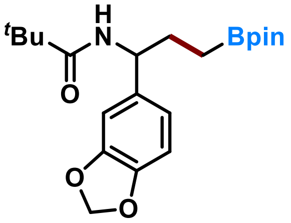

***N*-(1-(benzo[*d*][1,3]dioxol-5-yl)-3-(4,4,5,5-tetramethyl-1,3,2-dioxaborolan-2-yl)propyl)pivalamide 2p**

Eluent: dichloromethane/ethyl acetate (15:1). White solid (56.0 mg, 72% yield). **^{1}H NMR** (400 MHz, Chloroform-*d*) δ 6.71 (d, *J* = 2.4 Hz, 3H), 5.94 (d, *J* = 7.4 Hz, 1H), 5.90 (s, 2H), 4.73 (q, *J* = 7.4 Hz, 1H), 1.80 (dtd, *J* = 9.2, 6.9, 4.5 Hz, 2H), 1.22 (s, 12H), 1.17 (s, 9H), 0.79 – 0.64 (m, 2H). **^{13}C{^{1}H} NMR** (101 MHz, Chloroform-*d*) δ 177.7, 147.8, 146.6, 136.9, 119.8, 108.3, 106.9, 101.0, 83.3, 55.1, 38.7, 30.8, 27.7, 24.9, 24.8. **^{11}B NMR** (128 MHz, Chloroform-*d*) δ 33.64. **HRMS** (ESI) m/z: $[M+Na]^+$ calcd for $C_{21}H_{32}BNO_5Na^+$: 412.2266; found: 412.2272.

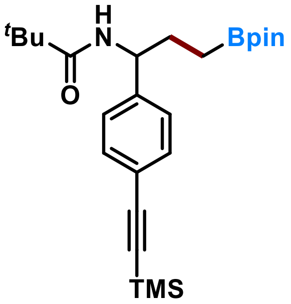

***N*-(3-(4,4,5,5-tetramethyl-1,3,2-dioxaborolan-2-yl)-1-(4 ((trimethylsilyl)ethynyl)phenyl)propyl)pivalamide 2q**

Eluent: dichloromethane/ethyl acetate (15:1). White solid (60.8 mg, 69% yield). **^{1}H NMR** (400 MHz, Chloroform-*d*) δ 7.40 – 7.35 (m, 2H), 7.17 – 7.12 (m, 2H), 6.00 (d, *J* = 7.7 Hz, 1H), 4.82 (q, *J* = 7.4 Hz, 1H), 1.86 – 1.77 (m, 2H), 1.21 (s, 12H), 1.16 (s, 9H), 0.82 – 0.62 (m, 2H), 0.21 (s, 9H). **^{13}C{^{1}H} NMR** (101 MHz, Chloroform-*d*) δ 177.7, 143.4, 132.2, 126.3, 121.8, 105.1, 93.9, 83.3, 54.9, 38.7, 30.4, 27.6, 24.9, 24.8, 0.1. **^{11}B NMR** (128 MHz, Chloroform-*d*) δ 33.26. **HRMS** (ESI) m/z: $[M+Na]^+$ calcd for $C_{25}H_{40}BNO_3SiNa^+$: 464.2763; found: 464.2770.

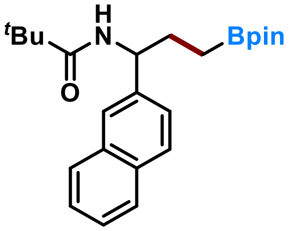

***N*-(1-(naphthalen-2-yl)-3-(4,4,5,5-tetramethyl-1,3,2-dioxaborolan-2-yl)propyl)pivalamide 2r**

Eluent: dichloromethane/ethyl acetate (15:1). White solid (56.1 mg, 79% yield). **^{1}H NMR** (400 MHz, Chloroform-*d*) δ 7.90 – 7.74 (m, 4H), 7.69 (d, J = 1.8 Hz, 1H), 7.48 – 7.34 (m, 4H), 6.09 (d, *J* = 7.9 Hz, 1H), 5.03 (q, *J* = 7.4 Hz, 1H), 1.97 (dt, *J* = 8.7, 7.3 Hz, 2H), 1.24 – 1.18 (m, 21H), 0.79 (dq, *J* = 33.0, 8.6, 8.0 Hz, 2H). **^{13}C{^{1}H} NMR** (101 MHz, Chloroform-*d*) δ 177.8, 140.2, 133.5, 132.8, 128.4, 128.0, 127.7, 126.1, 125.7, 125.2, 124.8, 83.4, 55.3, 38.8, 30.6, 27.7, 25.0, 24.9. **^{11}B NMR** (128 MHz, Chloroform-*d*) δ 34.48. Spectroscopic data are in agreement with those previously reported.[3,4]

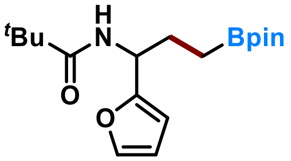

***N*-(1-(furan-2-yl)-3-(4,4,5,5-tetramethyl-1,3,2-dioxaborolan-2-yl)propyl)pivalamide 2s**

Eluent: dichloromethane/ethyl acetate (15:1). White solid (52.3 mg, 78% yield). **^{1}H NMR** (400 MHz, Chloroform-*d*) (400 MHz, Chloroform-d) δ 7.29 (dd, *J* = 2.0, 0.9 Hz, 1H), 6.25 (dd, *J* = 3.2, 1.9 Hz, 1H), 6.10 (d, *J* = 3.2 Hz, 1H), 5.93 (d, *J* = 8.6 Hz, 1H), 5.00 (dt, *J* = 8.8, 7.2 Hz, 1H), 1.97 – 1.76 (m, 2H), 1.20 (s, 12H), 1.16 (s, 9H), 0.80 – 0.62 (m, 2H). **^{13}C{^{1}H} NMR** (101 MHz, Chloroform-*d*) δ 177.6, 154.7, 141.7, 110.1, 106.1, 83.2, 48.9, 38.8, 28.4, 27.6, 24.9, 24.8. **^{11}B**

NMR (128 MHz, Chloroform-*d*) δ 33.65. **HRMS** (ESI) m/z: $[M+Na]^+$ calcd for $C_{18}H_{30}BNO_4Na^+$: 358.2160; found: 358.2168.

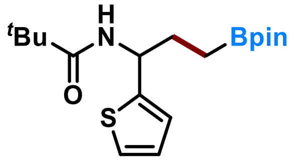

***N*-(3-(4,4,5,5-tetramethyl-1,3,2-dioxaborolan-2-yl)-1-(thiophen-2-yl)propyl)pivalamide 2t**

Eluent: dichloromethane/ethyl acetate (15:1). Yellow solid (50.5 mg, 72% yield). **^{1}H NMR** (400 MHz, Chloroform-*d*) (400 MHz, Chloroform-d) δ 7.15 – 7.13 (m, 1H), 6.92 – 6.90 (m, 2H), 5.92 (d, *J* = 8.4 Hz, 1H), 5.19 (td, *J* = 8.0, 6.3 Hz, 1H), 2.02 – 1.85 (m, 2H), 1.22 (s, 12H), 1.18 (s, 9H), 0.90 – 0.72 (m, 2H). **^{13}C{^{1}H} NMR** (101 MHz, Chloroform-*d*) δ 177.6, 146.9, 126.8, 124.0, 123.7, 83.3, 50.6, 38.7, 31.0, 27.6, 24.9, 24.8. **^{11}B NMR** (128 MHz, Chloroform-*d*) δ 33.52. Spectroscopic data are in agreement with those previously reported.[3,4]

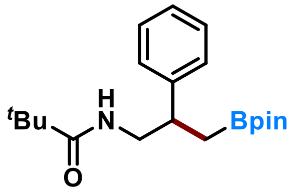

***N*-(2-phenyl-3-(4,4,5,5-tetramethyl-1,3,2-dioxaborolan-2-yl)propyl)pivalamide 2u**

Eluent: dichloromethane/ethyl acetate (15:1). Yellow oil (51.1 mg, 74% yield). **^{1}H NMR** (400 MHz, Chloroform-*d*) δ 7.32 – 7.25 (m, 2H), 7.25 – 7.14 (m, 3H), 5.46 (d, *J* = 5.8 Hz, 1H), 3.65 – 3.54 (m, 1H), 3.24 (ddd, *J* = 13.5, 9.1, 5.1 Hz, 1H), 3.14 – 2.98 (m, 1H), 1.17 (dd, *J* = 7.9, 2.6 Hz, 2H), 1.08 (d, J = 9.0 Hz, 12H), 1.04 (s, 9H). **^{13}C{^{1}H} NMR** (101 MHz, Chloroform-*d*) δ 178.1, 144.1, 128.5, 127.7, 126.7, 83.3, 46.6, 41.2, 38.7, 27.5, 24.8, 24.6. **^{11}B NMR** (128 MHz, Chloroform-*d*) δ 33.86. Spectroscopic data are in agreement with those previously reported.[3,4]

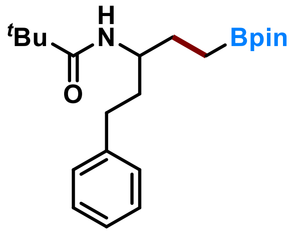

***N*-(1-phenyl-5-(4,4,5,5-tetramethyl-1,3,2-dioxaborolan-2-yl)pentan-3-yl)pivalamide 2v**

Eluent: dichloromethane/ethyl acetate (15:1). White solid (29.8 mg, 40% yield). **^{1}H NMR** (400 MHz, Chloroform-*d*) δ 7.30 – 7.23 (m, 2H), 7.17 (d, *J* = 7.4 Hz, 3H), 5.39 (d, *J* = 8.9 Hz, 1H),

3.94 (qt, J = 8.2, 5.1 Hz, 1H), 2.62 (t, J = 8.1 Hz, 2H), 1.86 – 1.78 (m, 1H), 1.73 – 1.63 (m, 2H), 1.56 – 1.46 (m, 1H), 1.24 (s, 12H), 1.18 (s, 9H), 0.81 – 0.74 (m, 2H). **$^{13}C\{^1H\}$ NMR** (101 MHz, Chloroform-*d*) δ 178.1, 142.3, 128.5, 125.9, 83.3, 50.7, 38.8, 36.8, 32.4, 29.1, 27.8, 25.0, 24.8. **^{11}B NMR** (128 MHz, Chloroform-*d*) δ 33.81. **HRMS** (ESI) m/z: $[M+Na]^+$ calcd for $C_{22}H_{36}BNO_3Na^+$: 396.2680; found: 396.2682.

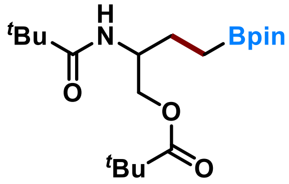

2-pivalamido-4-(4,4,5,5-tetramethyl-1,3,2-dioxaborolan-2-yl)butyl pivalate 2w

Eluent: dichloromethane/ethyl acetate (15:1). Yellow solid (39.8 mg, 52% yield). **^1H NMR** (400 MHz, Chloroform-*d*) δ 5.72 (d, J = 7.7 Hz, 1H), 4.15 – 4.08 (m, 2H), 4.08 – 4.01 (m, 1H), 1.59 (ddd, J = 29.7, 14.1, 7.6 Hz, 1H), 1.23 (s, 12H), 1.19 (s, 7H), 1.17 (s, 8H), 0.79 (t, J = 8.2 Hz, 2H). **$^{13}C\{^1H\}$ NMR** (101 MHz, Chloroform-*d*) δ 178.6, 178.1, 83.4, 65.5, 50.1, 39.0, 38.8, 27.7, 27.3, 25.9, 25.0, 24.8. **^{11}B NMR** (128 MHz, Chloroform-*d*) δ 34.25. Spectroscopic data are in agreement with those previously reported.[3,4]

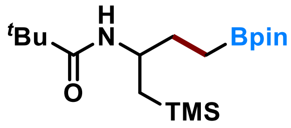

***N*-(4-(4,4,5,5-tetramethyl-1,3,2-dioxaborolan-2-yl)-1-(trimethylsilyl)butan-2-yl)pivalamide 2x**

Eluent: dichloromethane/ethyl acetate (15:1). White solid (29.1 mg, 41% yield). **^1H NMR** (400 MHz, Chloroform-*d*) δ 5.37 (d, J = 8.8 Hz, 1H), 4.06 – 3.93 (m, 1H), 1.65 – 1.57 (m, 1H), 1.51 – 1.43 (m, 1H), 1.23 (s, 12H), 1.16 (s, 9H), 0.82 – 0.66 (m, 4H), 0.02 (s, 9H). **$^{13}C\{^1H\}$ NMR** (101 MHz, Chloroform-*d*) δ 177.2, 83.2, 48.1, 38.6, 32.4, 29.8, 27.7, 25.0, 24.9, 23.5, -0.7. **^{11}B NMR** (128 MHz, Chloroform-*d*) δ 34.18. **HRMS** (ESI) m/z: $[M+Na]^+$ calcd for $C_{18}H_{38}BNO_3SiNa^+$: 378.2606; found: 378.2614.

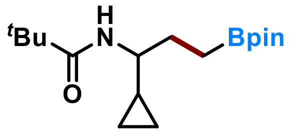

N-(1-cyclopropyl-3-(4,4,5,5-tetramethyl-1,3,2-dioxaborolan-2-yl)propyl)pivalamide 2y

Eluent: dichloromethane/ethyl acetate (15:1). White solid (40.8 mg, 66% yield). **^{1}H NMR** (400 MHz, Chloroform-*d*) δ 5.49 (d, *J* = 8.7 Hz, 1H), 3.26 (qd, *J* = 8.3, 6.1 Hz, 1H), 1.76 – 1.51 (m, 2H), 1.21 (s, 12H), 1.16 (s, 9H), 0.84 – 0.68 (m, 3H), 0.47 (dtt, *J* = 11.9, 5.1, 2.6 Hz, 1H), 0.36 – 0.29 (m, 1H), 0.27 – 0.18 (m, 2H). **^{13}C{^{1}H} NMR** (101 MHz, Chloroform-*d*) δ 177.9, 83.2, 54.7, 38.8, 29.7, 27.8, 24.9, 24.8, 15.9, 3.9, 2.0. **^{11}B NMR** (128 MHz, Chloroform-*d*) δ 33.74. **HRMS** (ESI) m/z: $[M+Na]^+$ calcd for $C_{17}H_{32}BNO_3Na^+$: 332.2367; found: 332.2373.

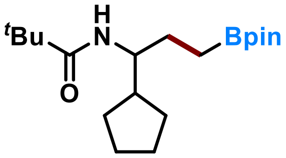

N-(1-cyclopentyl-3-(4,4,5,5-tetramethyl-1,3,2-dioxaborolan-2-yl)propyl)pivalamide 2z

Eluent: dichloromethane/ethyl acetate (15:1). White solid (47.2 mg, 70% yield). **^{1}H NMR** (400 MHz, Chloroform-*d*) δ 5.35 (d, *J* = 9.5 Hz, 1H), 3.84 – 3.72 (m, 1H), 1.87 – 1.79 (m, 1H), 1.73 – 1.43 (m, 7H), 1.37 – 1.30 (m, 1H), 1.22 (s, 14H), 1.17 (s, 9H), 0.78 – 0.67 (m, 2H). **^{13}C{^{1}H} NMR** (101 MHz, Chloroform-*d*) δ 178.0, 83.1, 44.8, 38.9, 29.9, 28.9, 28.4, 27.9, 25.6, 25.4, 25.0, 24.8. **^{11}B NMR** (128 MHz, Chloroform-*d*) δ 34.02. **HRMS** (ESI) m/z: $[M+Na]^+$ calcd for $C_{19}H_{36}BNO_3Na^+$: 360.2680; found: 360.2685.

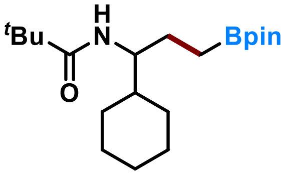

N-(1-cyclohexyl-3-(4,4,5,5-tetramethyl-1,3,2-dioxaborolan-2-yl)propyl)pivalamide 2aa

Eluent: dichloromethane/ethyl acetate (15:1). White solid (33.0 mg, 47% yield). **^{1}H NMR** (400 MHz, Chloroform-*d*) δ 5.33 (d, *J* = 9.5 Hz, 1H), 3.72 (tt, *J* = 9.6, 4.9 Hz, 1H), 1.75 – 1.59 (m, 7H), 1.40 – 1.33 (m, 3H), 1.23 (s, 12H), 1.18 (s, 9H), 0.93 (tdd, *J* = 23.4, 11.9, 8.4 Hz, 3H), 0.75 – 0.63 (m, 2H). **^{13}C{^{1}H} NMR** (101 MHz, Chloroform-*d*) δ 178.0, 83.2, 55.0, 42.0, 38.9, 29.9, 28.4, 27.9, 26.6, 26.4, 26.4, 26.2, 25.0, 24.8. **^{11}B NMR** (128 MHz, Chloroform-*d*) δ 34.41. **HRMS** (ESI) m/z: $[M+Na]^+$ calcd for $C_{20}H_{38}BNO_3Na^+$: 374.2837; found: 374.2844.

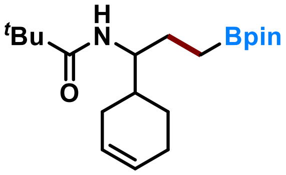

***N*-(1-(cyclohex-3-en-1-yl)-3-(4,4,5,5-tetramethyl-1,3,2-dioxaborolan-2-yl)propyl)pivalamide 2ab**

Eluent: dichloromethane/ethyl acetate (15:1). White solid (53.7 mg, 77% yield). ^{1}**H NMR** (400 MHz, Chloroform-*d*) δ 5.66 – 5.56 (m, 2H), 5.35 (dd, *J* = 9.4, 5.8 Hz, 1H), 3.81 (qt, *J* = 9.5, 4.8 Hz, 1H), 2.10 – 1.95 (m, 4H), 1.79 – 1.59 (m, 3H), 1.21 (s, 11H), 1.17 (d, *J* = 1.7 Hz, 8H), 0.76 – 0.67 (m, 2H). ^{13}**C{^{1}H} NMR** (101 MHz, Chloroform-*d*) δ 178.0, 177.9, 127.1, 126.8, 126.4, 126.0, 83.0, 54.4, 54.1, 38.8, 37.9, 37.9, 28.7, 27.7, 26.9, 26.4, 26.1, 25.9, 25.5, 25.4, 24.8, 24.7, 24.2. ^{11}**B NMR** (128 MHz, Chloroform-*d*) δ 33.60. **HRMS** (ESI) m/z: $[M+Na]^+$ calcd for $C_{20}H_{36}BNO_3Na^+$: 372.2680; found: 372.2684.

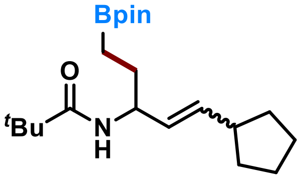

***N*-(1-cyclopentyl-5-(4,4,5,5-tetramethyl-1,3,2-dioxaborolan-2-yl)pent-1-en-3-yl)pivalamide 2ac**

Eluent: dichloromethane/ethyl acetate (15:1). Colorless oil (48.6 mg, 67% yield). Mixture of (Z/E) isomers. ^{1}**H NMR** (400 MHz, Chloroform-*d*) δ 5.55 – 5.36 (m, 2H), 5.31 – 5.01 (m, 1H), 4.67 – 4.28 (m, 1H), 2.87 – 2.34 (m, 1H), 1.77 – 1.46 (m, 9H), 1.23 (s, 13H), 1.16 (d, *J* = 9.8 Hz, 9H), 0.77 – 0.70 (m, 2H). ^{13}**C{^{1}H} NMR** (101 MHz, Chloroform-*d*) δ 177.5, 177.2, 138.7, 136.1, 128.3, 128.2, 83.2, 83.2, 52.2, 48.7, 43.0, 38.7, 38.6, 34.0, 33.9, 33.1, 33.1, 30.1, 29.5, 27.8, 27.7, 25.5, 25.4, 25.1, 24.9, 24.9, 24.9. ^{11}**B NMR** (128 MHz, Chloroform-*d*) δ 34.66. **HRMS** (ESI) m/z: $[M+Na]^+$ calcd for $C_{21}H_{38}BNNaO_3^+$: 386.2837; found: 386.2836.

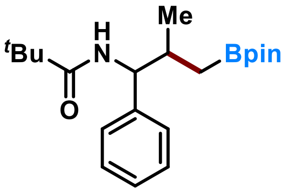

***N*-(2-methyl-1-phenyl-3-(4,4,5,5-tetramethyl-1,3,2-dioxaborolan-2-**

yl)propyl)pivalamide 2ad

Eluent: dichloromethane/ethyl acetate (15:1). Colorless oil (34.5 mg, 48% yield). Mixture of isomers (*d.r.* = 2:1). **^{1}H NMR** (400 MHz, Chloroform-*d*) δ 7.31 – 7.26 (m, 2H), 7.20 (tt, *J* = 5.7, 3.0 Hz, 3H), 6.24 (dd, *J* = 128.8, 8.6 Hz, 1H), 4.84 (dt, *J* = 8.6, 6.3 Hz, 1H), 2.18 (tdd, *J* = 11.2, 5.7, 3.7 Hz, 1H), 1.25 (d, *J* = 1.9 Hz, 9H), 1.23 – 1.20 (m, 12H), 0.92 (t, *J* = 7.1 Hz, 4H), 0.60 (ddd, *J* = 15.7, 11.6, 9.4 Hz, 1H). **^{13}C{^{1}H} NMR** (101 MHz, Chloroform-*d*) δ 177.7, 177.4, 142.1, 141.8, 128.2, 128.2, 126.8, 126.7, 126.7,126.6, 83.3, 83.1, 58.7, 38.7, 34.8, 27.6, 27.6, 25.0, 24.9, 24.6, 24.5, 19.5, 18.1. **^{11}B NMR** (128 MHz, Chloroform-*d*) δ 33.96. **HRMS** (ESI) m/z: $[M+Na]^+$ calcd for $C_{21}H_{34}BNNaO_3^+$: 382.2524; found: 382.2525.

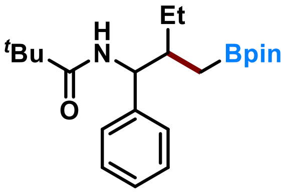

***N*-(1-phenyl-2-((4,4,5,5-tetramethyl-1,3,2-dioxaborolan-2-yl)methyl)butyl)pivalamide 2ae**

Eluent: dichloromethane/ethyl acetate (15:1). Colorless oil (46.2 mg, 62% yield). Mixture of isomers (*d.r.* = 1:1). **^{1}H NMR** (400 MHz, Chloroform-*d*) δ 7.28 (dd, *J* = 8.2, 6.8 Hz, 2H), 7.22 – 7.18 (m, 3H), 6.54 (dd, *J* = 233.4, 8.6 Hz, 1H), 5.05 (ddd, *J* = 39.4, 8.6, 6.0 Hz, 1H), 2.07 – 1.97 (m, 1H), 1.52 – 1.31 (m, 2H), 1.28 – 1.19 (m, 21H), 0.91 (dt, *J* = 15.2, 7.4 Hz, 3H), 0.75 – 0.64 (m, 2H). **^{13}C{^{1}H} NMR** (101 MHz, Chloroform-*d*) δ 177.8, 177.3, 142.7, 141.8, 128.2, 128.1, 126.8, 126.6, 126.4, 126.2, 83.5, 83.1, 56.9, 55.4, 41.0, 40.9, 38.8, 38.6, 27.6, 27.5, 26.4, 25.2, 24.8, 24.8, 24.7, 24.5, 11.6, 11.3. **^{11}B NMR** (128 MHz, Chloroform-*d*) δ 33.43. **HRMS** (ESI) m/z: $[M+Na]^+$ calcd for $C_{22}H_{36}BNNaO_3^+$: 396.2680; found: 396.2677.

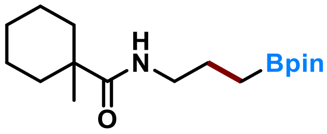

1-methyl-*N*-(3-(4,4,5,5-tetramethyl-1,3,2-dioxaborolan-2-yl)propyl)cyclohexane-1-carboxamide 2af

Eluent: dichloromethane/ethyl acetate (15:1). White solid (47.0 mg, 76% yield). **^{1}H NMR** (400 MHz, Chloroform-*d*) δ 5.79 (s, 1H), 3.24 – 3.19 (m, 2H), 1.86 (ddd, *J* = 13.3, 7.1, 3.0 Hz, 2H),

1.59 (p, J = 7.5 Hz, 2H), 1.53 – 1.29 (m, 8H), 1.22 (s, 12H), 1.10 (s, 3H), 0.78 (t, J = 7.8 Hz, 2H). **^{13}C{^{1}H} NMR** (101 MHz, Chloroform-*d*) δ 177.7, 83.36,42.7, 41.6, 35.8, 26.0, 24.9, 24.0, 23.0. **^{11}B NMR** (128 MHz, Chloroform-*d*) δ 33.66. Spectroscopic data are in agreement with those previously reported.[3,4]

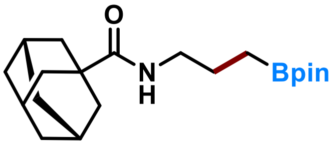

***N*-(3-(4,4,5,5-tetramethyl-1,3,2-dioxaborolan-2-yl)propyl)adamantane-1-carboxamide 2ag**

Eluent: dichloromethane/ethyl acetate (15:1). White solid (38.8 mg, 56% yield). **^{1}H NMR** (400 MHz, Chloroform-*d*) δ 5.72 (s, 1H), 3.19 (td, J = 7.2, 5.5 Hz, 2H), 2.03 – 1.98 (m, 3H), 1.82 (d, J = 2.9 Hz, 6H), 1.73 – 1.64 (m, 6H), 1.63 – 1.51 (m, 2H), 1.22 (s, 12H), 0.76 (t, J = 7.9 Hz, 2H). **^{13}C{^{1}H} NMR** (101 MHz, Chloroform-*d*) δ 177.9, 83.3, 41.4, 40.6, 39.4, 36.6, 28.2, 24.9, 23.9. **^{11}B NMR** (128 MHz, Chloroform-*d*) δ 33.68. Spectroscopic data are in agreement with those previously reported.[3,4]

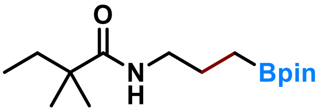

2,2-dimethyl-*N*-(3-(4,4,5,5-tetramethyl-1,3,2-dioxaborolan-2-yl)propyl)butanamide 2ah

Eluent: dichloromethane/ethyl acetate (15:1). Yellow oil (36.2 mg, 64% yield). **^{1}H NMR** (400 MHz, Chloroform-*d*) δ5.74 (s, 1H), 3.21 (td, J = 7.2, 5.6 Hz, 2H), 1.59 (q, J = 7.5 Hz, 2H), 1.52 (q, J = 7.5 Hz, 2H), 1.23 (s, 12H), 1.12 (s, 6H), 0.86 – 0.77 (m, 5H). **^{13}C{^{1}H} NMR** (101 MHz, Chloroform-*d*) δ 177.7, 83.3, 42.4, 41.6, 34.0, 25.1, 24.9, 24.0, 9.3. **^{11}B NMR** (128 MHz, Chloroform-*d*) δ 34.27. **HRMS** (ESI) m/z: $[M+Na]^+$ calcd for $C_{15}H_{30}BNO_3Na^+$: 306.2211; found: 306.2215.

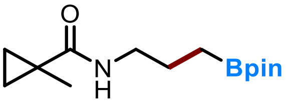

1-methyl-*N*-(3-(4,4,5,5-tetramethyl-1,3,2-dioxaborolan-2-yl)propyl)cyclopropane-1-carboxamide 2ai

Eluent: dichloromethane/ethyl acetate (15:1). White solid (19.2 mg, 36% yield). **^{1}H NMR** (400 MHz, Chloroform-*d*) δ 5.85 (s, 1H), 3.24 (td, J = 7.2, 5.7 Hz, 1H), 1.61 (q, J = 7.4, 6.9 Hz, 2H), 1.30 (s, 3H), 1.24 (s, 12H), 1.17 (q, J = 3.9 Hz, 2H), 0.81 (t, J = 7.8 Hz, 2H), 0.53 (q, J = 3.8 Hz,

2H). **^{13}C{^{1}H} NMR** (101 MHz, Chloroform-*d*) δ 174.7, 83.2, 41.9, 24.8, 23.9, 19.8, 18.9, 15.8. **^{11}B NMR** (128 MHz, Chloroform-*d*) δ 34.31. **HRMS** (ESI) m/z: $[M+Na]^+$ calcd for $C_{14}H_{26}BNO_3Na^+$: 290.1890; found: 290.1900.

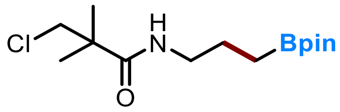

3-chloro-2,2-dimethyl-*N*-(3-(4,4,5,5-tetramethyl-1,3,2-dioxaborolan-2-yl)propyl)propanamide 2aj

Eluent: dichloromethane/ethyl acetate (15:1). White solid (20.0 mg, 33% yield). **^{1}H NMR** (400 MHz, Chloroform-*d*) δ 5.91 (s, 1H), 3.61 (s, 2H), 3.24 (td, *J* = 7.1, 5.6 Hz, 2H), 1.68 – 1.58 (m, 2H), 1.27 (s, 6H), 1.24 (s, 12H), 0.80 (t, *J* = 7.8 Hz, 2H). **^{13}C{^{1}H} NMR** (101 MHz, Chloroform-*d*) δ 174.7, 83.4, 53.0, 44.2, 41.9, 25.0, 24.9, 23.8, 23.6. **^{11}B NMR** (128 MHz, Chloroform-*d*) δ 34.26. **HRMS** (ESI) m/z: $[M+Na]^+$ calcd for $C_{14}H_{27}BClNO_3Na^+$: 326.1665; found: 326.1669.

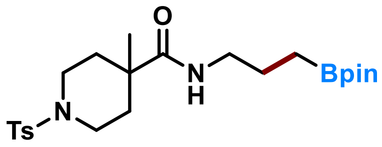

4-methyl-N-(3-(4,4,5,5-tetramethyl-1,3,2-dioxaborolan-2-yl)propyl)-1-tosylpiperidine-4-carboxamide 2ak

Eluent: dichloromethane/ethyl acetate (4:1). Colorless oil (61.2 mg, 66% yield). **^{1}H NMR** (400 MHz, Chloroform-*d*) δ 7.68 – 7.50 (m, 2H), 7.36 – 7.18 (m, 2H), 5.83 (t, *J* = 5.5 Hz, 1H), 3.28 (dt, *J* = 12.1, 4.6 Hz, 2H), 3.10 (td, *J* = 7.1, 5.5 Hz, 2H), 2.69 (ddd, *J* = 12.3, 9.9, 2.9 Hz, 2H), 2.39 (s, 3H), 2.08 – 1.99 (m, 2H), 1.58 – 1.45 (m, 4H), 1.20 (s, 12H), 0.68 (t, *J* = 7.7 Hz, 2H). **^{13}C{^{1}H} NMR** (101 MHz, Chloroform-*d*) δ 175.4, 143.5, 133.3, 129.8, 127.6, 83.3, 43.4, 41.6, 40.4, 34.5, 26.1, 24.9, 23.6, 21.6. **^{11}B NMR** (128 MHz, Chloroform-*d*) δ 33.27. **HRMS** (ESI) m/z: $[M+Na]^+$ calcd for $C_{21}H_{39}BN_2O_4Na^+$: 417.2895; found: 417.2900.

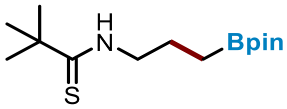

2,2-dimethyl-*N*-(3-(4,4,5,5-tetramethyl-1,3,2-dioxaborolan-2-yl)propyl)propanethioamide 2al

Eluent: dichloromethane/ethyl acetate (15:1). Yellow oil (12.0 mg, 21% yield). **^{1}H NMR** (400 MHz, Chloroform-*d*) δ 7.53 (s, 1H), 3.64 (td, *J* = 7.2, 5.0 Hz, 2H), 1.77 (p, *J* = 7.5 Hz, 2H), 1.34 (s, 9H), 1.25 (s, 12H), 0.85 (t, *J* = 7.7 Hz, 2H). **^{13}C{^{1}H} NMR** (101 MHz, Chloroform-*d*) δ 213.1, 83.5, 48.7, 44.5, 30.3, 24.9, 22.3. **^{11}B NMR** (128 MHz, Chloroform-*d*) δ 34.05. **HRMS** (ESI) m/z: $[M+Na]^+$ calcd for $C_{14}H_{28}BNSO_2Na^+$: 308.1826; found: 308.1828.

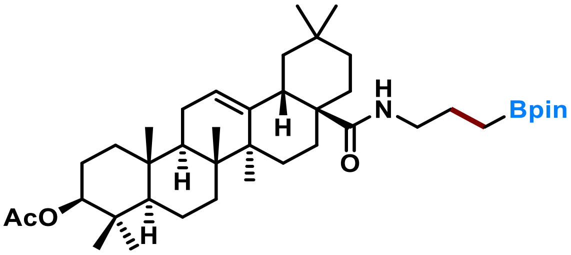

(3*S*,4a*R*,6a*R*,6b*S*,8a*S*,12a*S*,14a*R*,14b*R*)-4,4,6a,6b,11,11,14b-heptamethyl-8a-((3-(4,4,5,5-tetramethyl-1,3,2-dioxaborolan-2-yl)propyl)carbamoyl)-1,2,3,4,4a,5,6,6a,6b,7,8,8a,9,10,11,12,12a,14,14a,14b-icosahydropicen-3-yl acetate 2am

Eluent: dichloromethane/ethyl acetate (15:1). Colorless oil (65.2 mg, 49% yield). **^{1}H NMR** (400 MHz, Chloroform-*d*) δ 5.93 (dd, *J* = 6.6, 4.3 Hz, 1H), 5.40 – 5.28 (m, 1H), 4.47 (dd, *J* = 9.6, 6.3 Hz, 1H), 3.41 – 3.29 (m, 1H), 2.98 – 2.87 (m, 1H), 2.49 (dd, *J* = 13.1, 4.2 Hz, 1H), 2.02 (s, 3H), 1.96 – 1.86 (m, 3H), 1.76 – 1.31 (m, 16H), 1.22 (s, 14H), 1.15 –1.13 (m, 4H), 1.07 – 0.98 (m, 2H), 0.91 (s, 3H), 0.88 (s, 6H), 0.85 – 0.83 (m, 6H), 0.76 – 0.72 (m, 5H). **^{13}C{^{1}H} NMR** (101 MHz, Chloroform-*d*) δ 178.0, 171.1, 145.1, 122.6, 83.2, 80.9, 75.0, 55.3, 47.6, 46.8, 46.3, 42.3, 42.1, 41.4, 39.4, 38.2, 37.7, 36.9, 34.2, 33.1, 32.6, 32.4, 30.8, 28.1, 27.4, 25.8, 24.9, 24.9, 23.9, 23.8, 23.7, 23.6, 21.4, 18.2, 17.0, 16.7, 15.5. **^{11}B NMR** (128 MHz, Chloroform-*d*) δ 34.71. **HRMS** (ESI) m/z: $[M+Na]^+$ calcd for $C_{41}H_{68}BNO_5Na^+$: 688.5088; found: 688.5091.

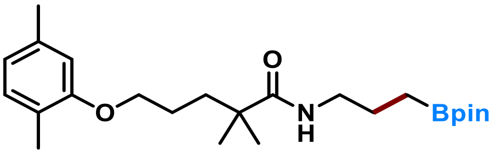

5-(2,5-dimethylphenoxy)-2,2-dimethyl-*N*-(3-(4,4,5,5-tetramethyl-1,3,2-dioxaborolan-2-yl)propyl)pentanamide 2an

Eluent: dichloromethane/ethyl acetate (15:1). White solid (49.2 mg, 59% yield). **^{1}H NMR** (400 MHz, Chloroform-*d*) δ 6.98 (dd, *J* = 7.3, 0.9 Hz, 1H), 6.69 – 6.62 (m, 1H), 6.60 (d, *J* = 1.6 Hz, 1H), 5.82 (t, *J* = 5.7 Hz, 1H), 3.90 (t, *J* = 5.9 Hz, 2H), 3.22 (td, *J* = 7.2, 5.6 Hz, 2H), 2.29 (s, 3H),

2.16 (s, 3H), 1.77 – 1.65 (m, 4H), 1.64 – 1.55 (m, 2H), 1.24 (s, 12H), 1.20 (s, 6H), 0.79 (t, *J* = 7.8 Hz, 2H). **^{13}C{^{1}H} NMR** (101 MHz, Chloroform-*d*) δ 177.4, 157.0, 136.5, 130.3, 123.6, 120.8, 112.1, 83.3, 68.1, 41.9, 41.7, 37.7, 25.6, 25.2, 24.9, 23.9, 21.4, 15.9. **^{11}B NMR** (128 MHz, Chloroform-*d*) δ 33.87. **HRMS** (ESI) m/z: $[M+Na]^+$ calcd for $C_{24}H_{40}BNO_4Na^+$: 440.2948; found: 440.2954.

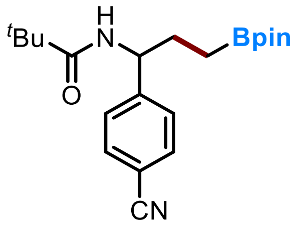

***N*-(1-(4-cyanophenyl)-3-(4,4,5,5-tetramethyl-1,3,2-dioxaborolan-2-yl)propyl)pivalamide 2ao**

Eluent: dichloromethane/ethyl acetate (10:1). White solid (37.0 mg, 50% yield). **^{1}H NMR** (400 MHz, Chloroform-*d*) δ 7.62 – 7.55 (m, 2H), 7.36 – 7.30 (m, 2H), 6.15 (d, *J* = 7.0 Hz, 1H), 4.82 (q, *J* = 7.1 Hz, 1H), 1.93 – 1.74 (m, 2H), 1.23 (s, 12H), 1.19 (s, 9H), 0.83 – 0.67 (m, 2H). **^{13}C{^{1}H} NMR** (101 MHz, Chloroform-*d*) δ 178.1, 148.8, 132.5, 127.1, 119.0, 110.8, 83.6, 55.3, 38.7, 30.4, 27.6, 25.0, 24.8. **^{11}B NMR** (128 MHz, Chloroform-*d*) δ 34.43. Spectroscopic data are in agreement with those previously reported.[3,4]

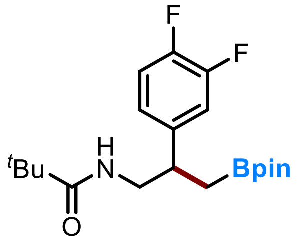

***N*-(2-(3,4-difluorophenyl)-3-(4,4,5,5-tetramethyl-1,3,2-dioxaborolan-2-yl)propyl)pivalamide 2ap**

Eluent: dichloromethane/ethyl acetate (15:1). Yellow oil (38.9 mg, 51% yield). **^{1}H NMR** (400 MHz, Chloroform-*d*) δ 7.12 – 6.99 (m, 2H), 6.94 – 6.90 (m, 1H), 5.53 (t, *J* = 6.1 Hz, 1H), 3.52 (dt, *J* = 13.3, 6.0 Hz, 1H), 3.24 (ddd, *J* = 13.3, 8.9, 5.6 Hz, 1H), 3.05 (tt, *J* = 9.1, 6.1 Hz, 1H), 1.23 (s, 2H), 1.10 (d, J = 11.1 Hz, 12H), 1.06 (s, 9H). **^{13}C{^{1}H} NMR** (101 MHz, Chloroform-*d*) δ 178.3, 151.9 – 147.5 (m), 143.0 – 140.5 (m), 123.6 (dd, *J* = 6.0, 3.5 Hz), 117.1 (d, *J* = 16.9 Hz), 116.5 (d, *J* = 16.9 Hz), 83.5, 46.5, 40.7, 38.7, 27.6, 24.8 (d, *J* = 18.5 Hz). **^{11}B NMR** (128 MHz,

Chloroform-*d*) δ 33.96. **^{19}F NMR** (376 MHz, Chloroform-d) δ -137.88 (ddd, *J* = 20.6, 11.6, 8.2 Hz), -140.24 – -141.43 (m). Spectroscopic data are in agreement with those previously reported.[3,4]

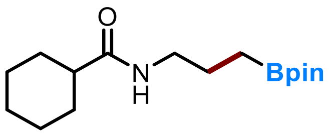

N-(3-(4,4,5,5-tetramethyl-1,3,2-dioxaborolan-2-yl)propyl)cyclohexanecarboxamide 2aq

Eluent: dichloromethane/ethyl acetate (15:1). Colorless oil (25.4 mg, 43% yield). **^{1}H NMR** (400 MHz, Chloroform-d) δ 5.64 (s, 1H), 3.21 (tdd, *J* = 7.2, 5.5, 1.7 Hz, 2H), 2.02 (dddd, *J* = 12.4, 10.8, 3.9, 2.2 Hz, 1H), 1.88 – 1.74 (m, 5H), 1.62 – 1.56 (m, 3H), 1.47 – 1.34 (m, 4H), 1.24 (s, 12H), 0.84 – 0.74 (m, 2H). **^{13}C{^{1}H} NMR** (101 MHz, Chloroform-d) δ 176.0, 83.3, 45.8, 41.4, 29.9, 25.9, 24.9, 23.9. **^{11}B NMR** (128 MHz, Chloroform-*d*) δ 34.35. **HRMS** (ESI) m/z: $[M+Na]^+$ calcd for $C_{16}H_{30}BNO_3Na^+$: 318.2211; found: 318.2214.

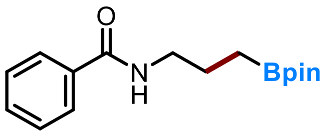

N-(3-(4,4,5,5-tetramethyl-1,3,2-dioxaborolan-2-yl)propyl)benzamide 2ar

Eluent: dichloromethane/ethyl acetate (15:1). Yellow oil (20.8 mg, 36% yield). **^{1}H NMR** (400 MHz, Chloroform-d) δ 7.79 – 7.74 (m, 2H), 7.50 – 7.39 (m, 3H), 6.48 (s, 1H), 3.45 (td, *J* = 6.9, 5.4 Hz, 2H), 1.73 (q, *J* = 7.2 Hz, 2H), 1.22 (s, 12H), 0.89 (t, J = 7.6 Hz, 2H). **^{13}C{^{1}H} NMR** (101 MHz, Chloroform-d) δ 167.7, 135.1, 131.3, 128.5, 127.0, 83.4, 42.3, 24.9, 23.7. **^{11}B NMR** (128 MHz, Chloroform-*d*) δ 34.34. **HRMS** (ESI) m/z: $[M+Na]^+$ calcd for $C_{16}H_{24}BNO_3Na^+$: 312.1741; found: 312.1740.

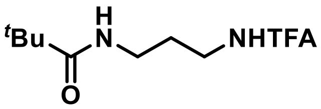

N-(3-(2,2,2-trifluoroacetamido)propyl)pivalamide 3a

Eluent: petroleum ether/ethyl acetate (2:1). White solid (41.9 mg, 66% yield). **^{1}H NMR** (400 MHz, Chloroform-*d*) δ 8.19 (s, 1H), 6.21 (s, 1H), 3.31 (qd, *J* = 6.4, 2.6 Hz, 4H), 1.73 – 1.58 (m, 2H), 1.20 (s, 9H). **^{13}C{^{1}H} NMR** (101 MHz, Chloroform-*d*) δ 180.4, 158.0, 157.6, 157.2, 117.5,

114.7, 38.9, 36.0, 35.8, 29.4, 27.6. **^{19}F NMR** (376 MHz, Chloroform-*d*) δ -75.94. **HRMS** (ESI) m/z: $[M+Na]^+$ calcd for $C_{10}H_{17}F_3N_2O_2Na^+$: 277.1134; found: 277.1132.

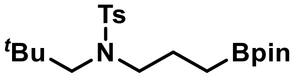

4-methyl-*N*-neopentyl-*N*-(3-(4,4,5,5-tetramethyl-1,3,2-dioxaborolan-2-yl)propyl)benzenesulfonamide 3b

Eluent: petroleum ether/ethyl acetate (1:1). White solid (58.9 mg, 72% yield). **^{1}H NMR** (400 MHz, Chloroform-*d*) δ 7.72 – 7.66 (m, 2H), 7.30 – 7.23 (m, 2H), 3.11 – 3.02 (m, 2H), 2.94 (s, 2H), 2.40 (s, 3H), 1.69 – 1.57 (m, 2H), 1.21 (s, 12H), 0.97 (s, 9H), 0.60 (t, *J* = 7.7 Hz, 2H). **^{13}C{^{1}H} NMR** (101 MHz, Chloroform-*d*) δ 142.9, 137.2, 129.5, 127.5, 83.2, 60.0, 53.1, 33.1, 28.4, 24.9, 22.6, 21.5. **^{11}B NMR** (128 MHz, Chloroform-*d*) δ 33.22. **HRMS** (ESI) m/z: $[M+Na]^+$ calcd for $C_{21}H_{36}BNO_4SNa^+$: 432.2350; found: 432.2351.

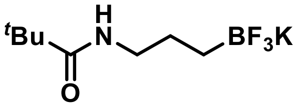

***N*-(3-(potassium trifluoroboratel)propyl)pivalamide 3c**

White solid (40.8 mg, 82% yield). **^{1}H NMR** (400 MHz, Acetone-*d*6) δ 6.84 (s, 1H), 3.15 – 3.05 (m, 2H), 1.42 (p, *J* = 7.3 Hz, 2H), 1.13 (s, 9H), 0.13 (h, *J* = 6.9 Hz, 2H). **^{13}C{^{1}H} NMR** (101 MHz, Acetone-*d*6) δ 177.0, 42.0, 38.0, 27.1, 25.5. **^{11}B NMR** (128 MHz, Acetone-*d*6) δ 5.42. **^{19}F NMR** (376 MHz, Acetone-*d*6) δ -140.03.

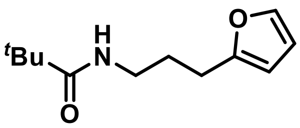

***N*-(3-(furan-2-yl)propyl)pivalamide 3d**

Eluent: petroleum ether/ethyl acetate (4:1). Yellow oil (30.9 mg, 74% yield). **^{1}H NMR** (400 MHz, Chloroform-*d*) δ 7.29 (dd, *J* = 1.8, 0.9 Hz, 1H), 6.27 (dd, *J* = 3.2, 1.9 Hz, 1H), 6.04 – 5.98 (m, 1H), 5.71 (s, 1H), 3.28 (td, *J* = 7.0, 5.8 Hz, 2H), 2.66 (t, *J* = 7.3 Hz, 2H), 1.84 (q, *J* = 7.1 Hz, 2H), 1.16 (s, 9H). **^{13}C{^{1}H} NMR** (101 MHz, Chloroform-*d*) δ 178.5, 155.3, 141.1, 110.3, 105.3, 39.1, 38.7, 28.0, 27.6, 25.6. **HRMS** (ESI) m/z: $[M+Na]^+$ calcd for $C_{12}H_{19}NO_2Na^+$: 232.1313; found: 232.1309.

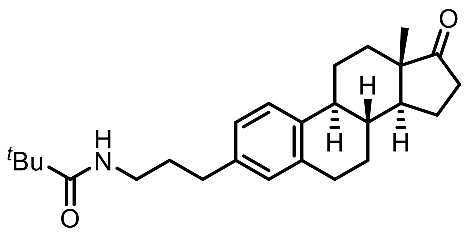

N-(3-((8R,9S,13S,14S)-13-methyl-17-oxo-7,8,9,11,12,13,14,15,16,17-decahydro-6H-cyclopenta[a]phenanthren-3-yl)propyl)pivalamide 3e

Eluent: petroleum ether/ethyl acetate (3:1). White solid (35.6 mg, 45% yield). **^{1}H NMR** (400 MHz, Chloroform-*d*) δ 7.22 (d, *J* = 8.0 Hz, 1H), 6.98 (dd, *J* = 7.9, 2.0 Hz, 1H), 6.92 (s, 1H), 5.53 (s, 1H), 3.28 (td, *J* = 7.0, 5.6 Hz, 2H), 2.89 (dd, *J* = 9.0, 4.2 Hz, 2H), 2.60 (t, *J* = 7.5 Hz, 2H), 2.50 (dd, *J* = 18.7, 8.6 Hz, 1H), 2.44 – 2.39 (m, 1H), 2.29 (d, *J* = 10.4 Hz, 1H), 2.21 – 2.11 (m, 1H), 2.07 – 1.94 (m, 3H), 1.83 (p, J = 7.2 Hz, 2H), 1.62 (s, 3H), 1.55 – 1.47 (m, 2H), 1.13 (s, 9H), 0.90 (s, 3H). **^{13}C{^{1}H} NMR** (101 MHz, Chloroform-*d*) δ 178.4, 139.1, 137.6, 136.7, 129.1, 125.9, 125.7, 50.6, 48.1, 44.4, 39.5, 38.7, 38.3, 36.0, 33.1, 31.7, 31.2, 29.8, 29.5, 27.6, 26.6, 25.9, 21.7, 13.9. **HRMS** (ESI) m/z: $[M+Na]^+$ calcd for $C_{26}H_{37}NO_2Na^+$: 418.2717; found: 418.2714.

NMR Spectra

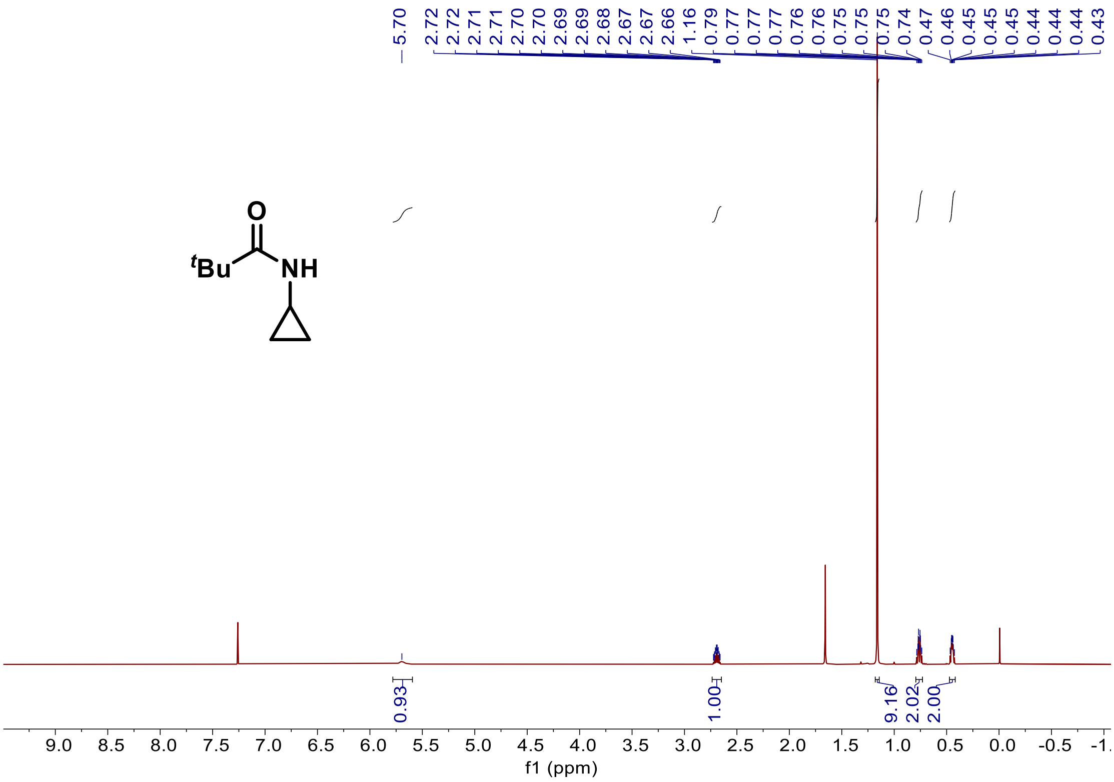

Supplementary Figure 11. ^{1}H NMR spectrum of **1a** (400 MHz, Chloroform-*d*)

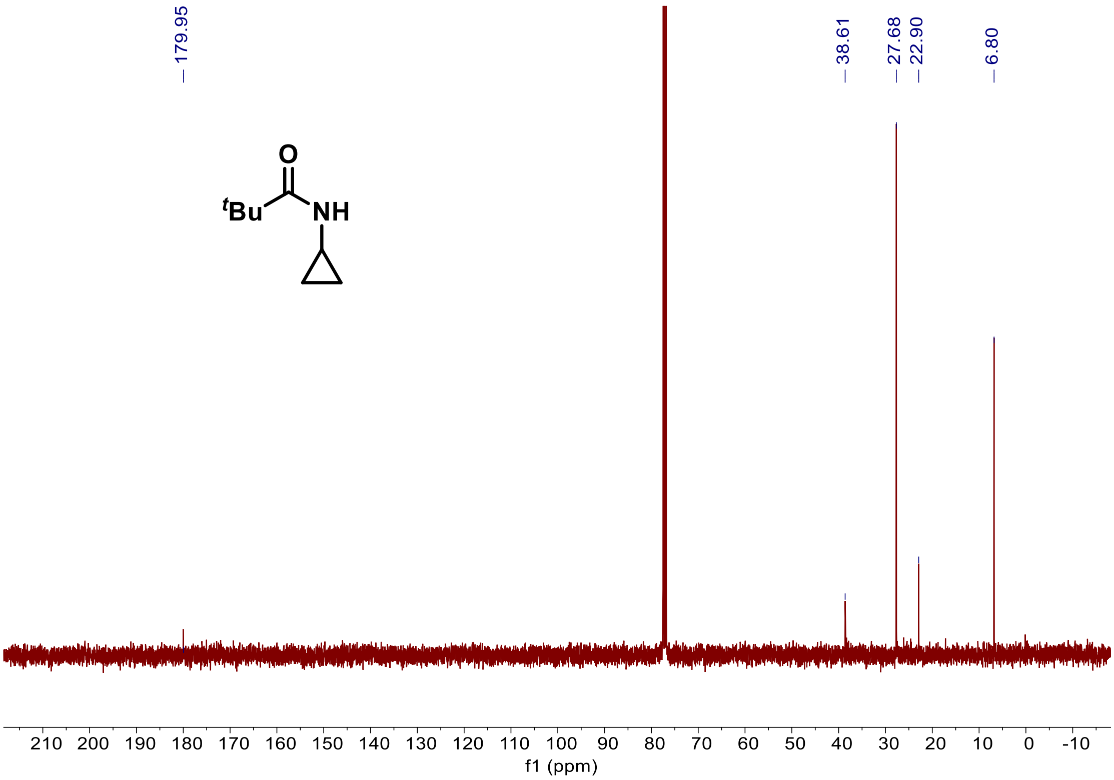

Supplementary Figure 12. $^{13}C\{^{1}H\}$ NMR spectrum of **1a** (101 MHz, Chloroform-*d*)

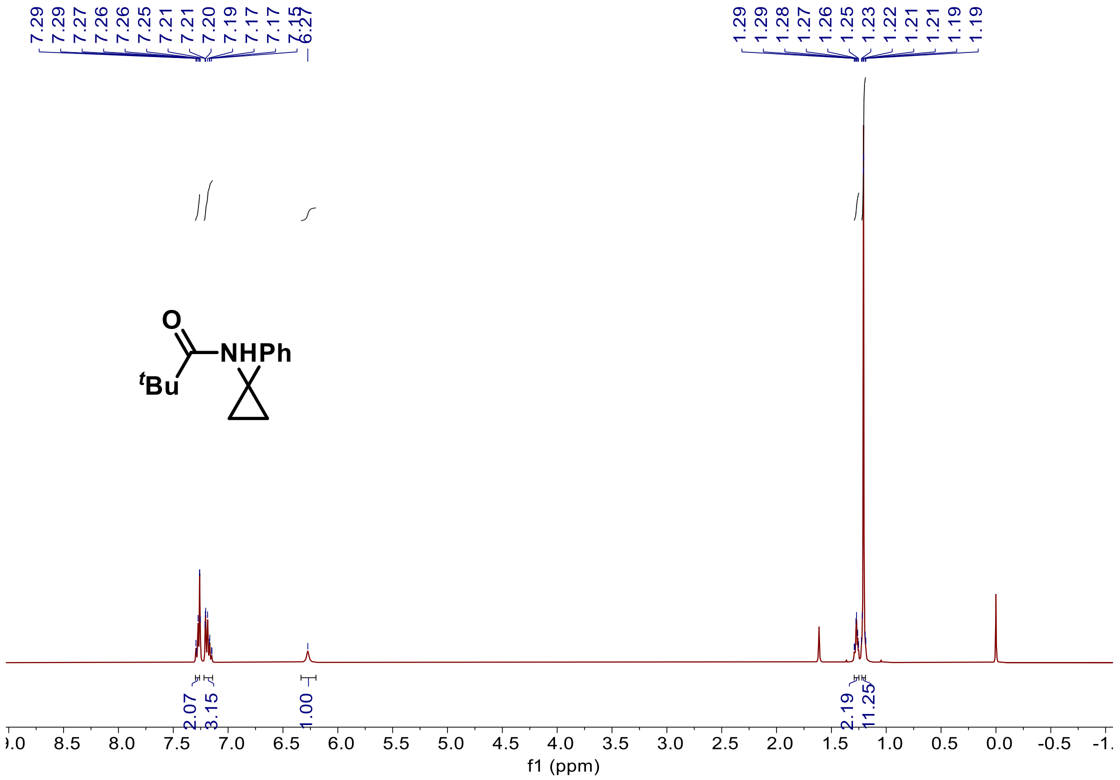

Supplementary Figure 13. ^{1}H NMR spectrum of **1b** (400 MHz, Chloroform-*d*)

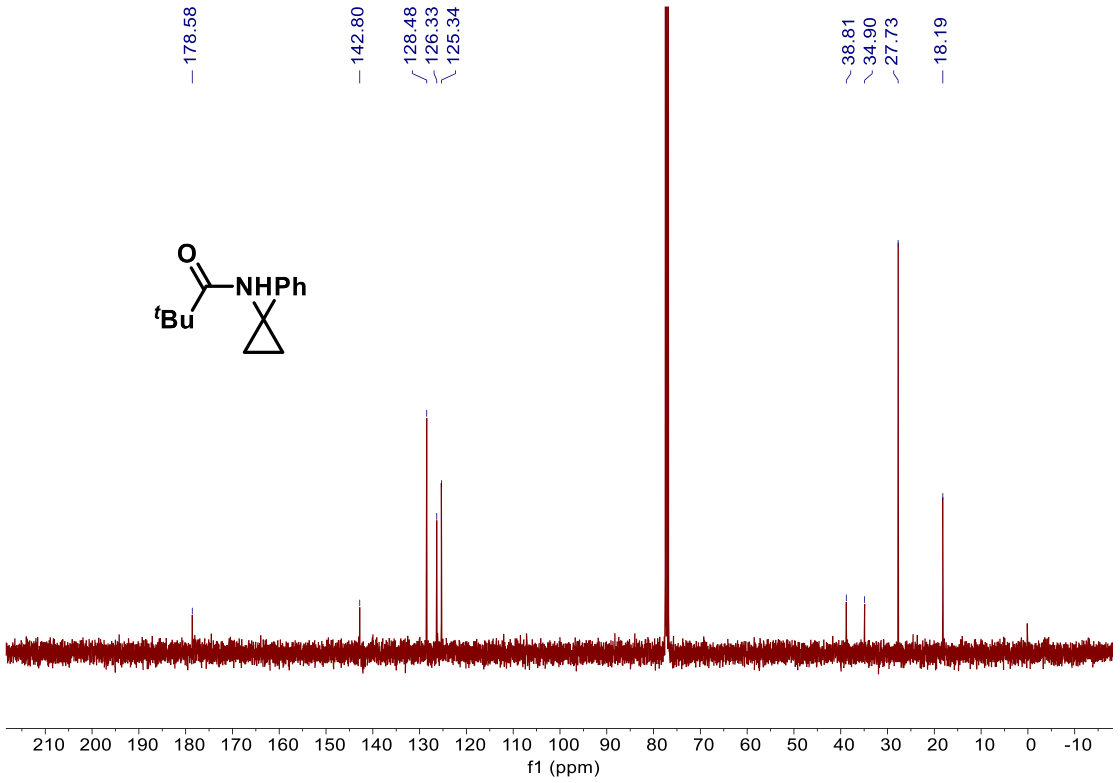

Supplementary Figure 14. ^{13}C{^{1}H} NMR spectrum of **1b** (101 MHz, Chloroform-*d*)

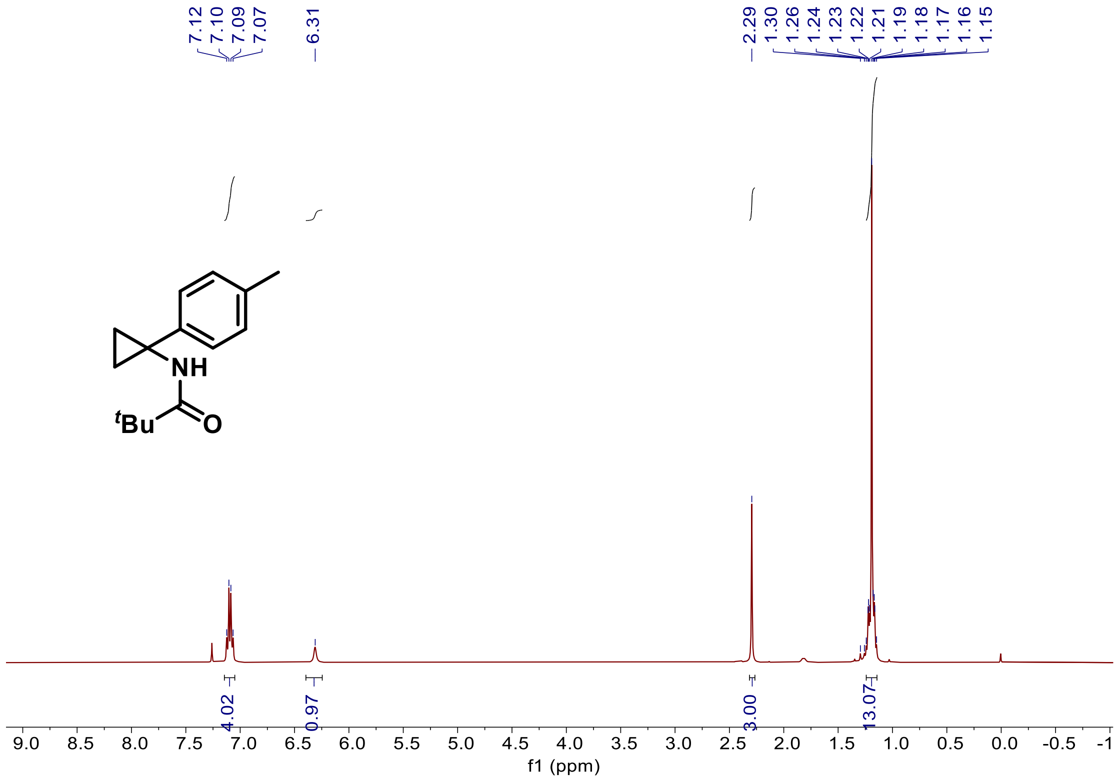

Supplementary Figure 15. ^{1}H NMR spectrum of **1c** (400 MHz, Chloroform-*d*)

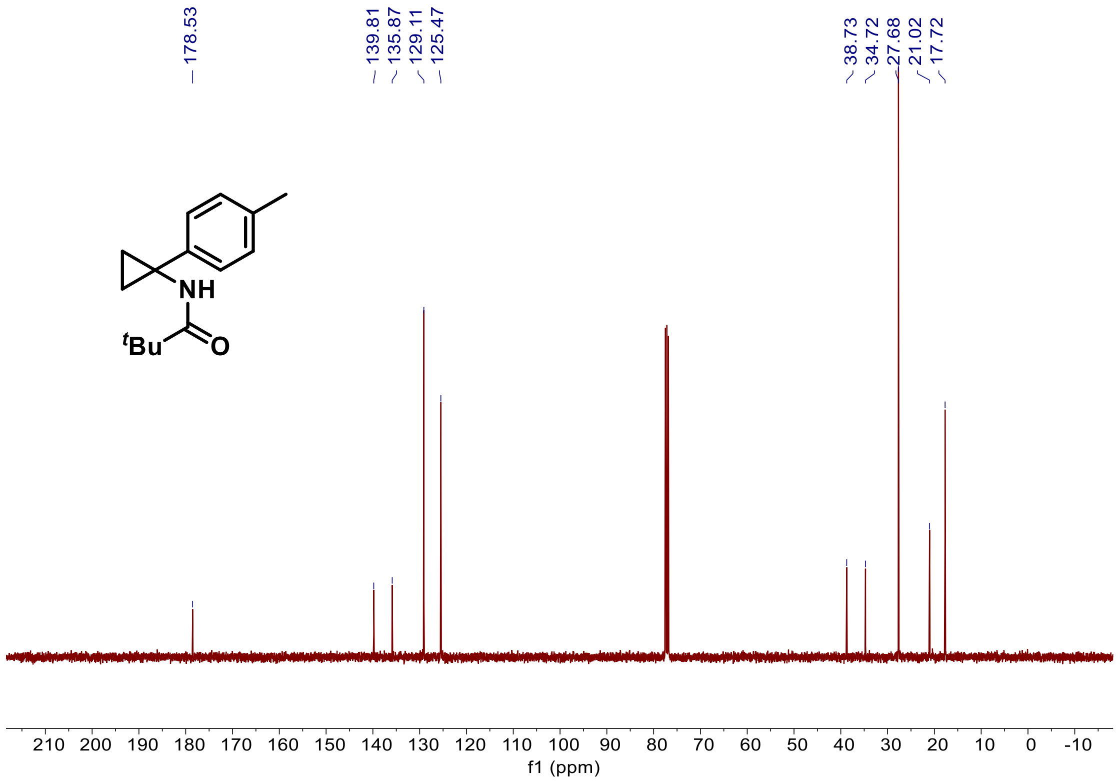

Supplementary Figure 16. $^{13}C\{^{1}H\}$ NMR spectrum of **1c** (101 MHz, Chloroform-*d*)

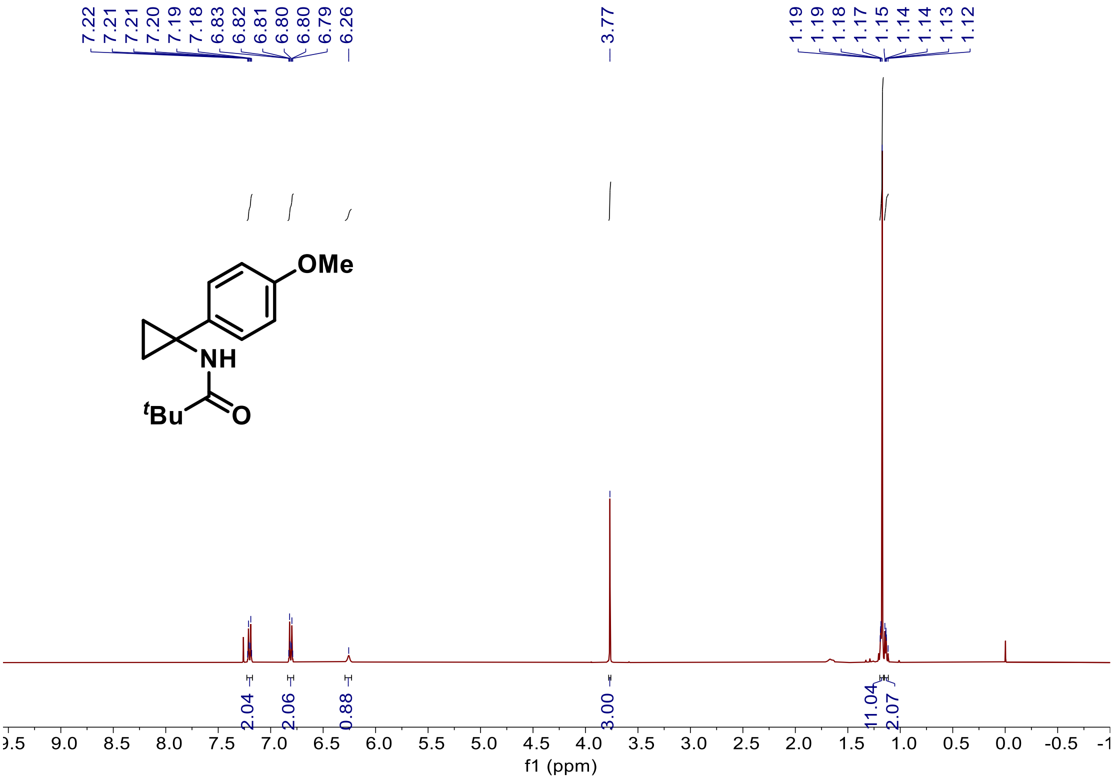

Supplementary Figure 17. ^{1}H NMR spectrum of **1d** (400 MHz, Chloroform-*d*)

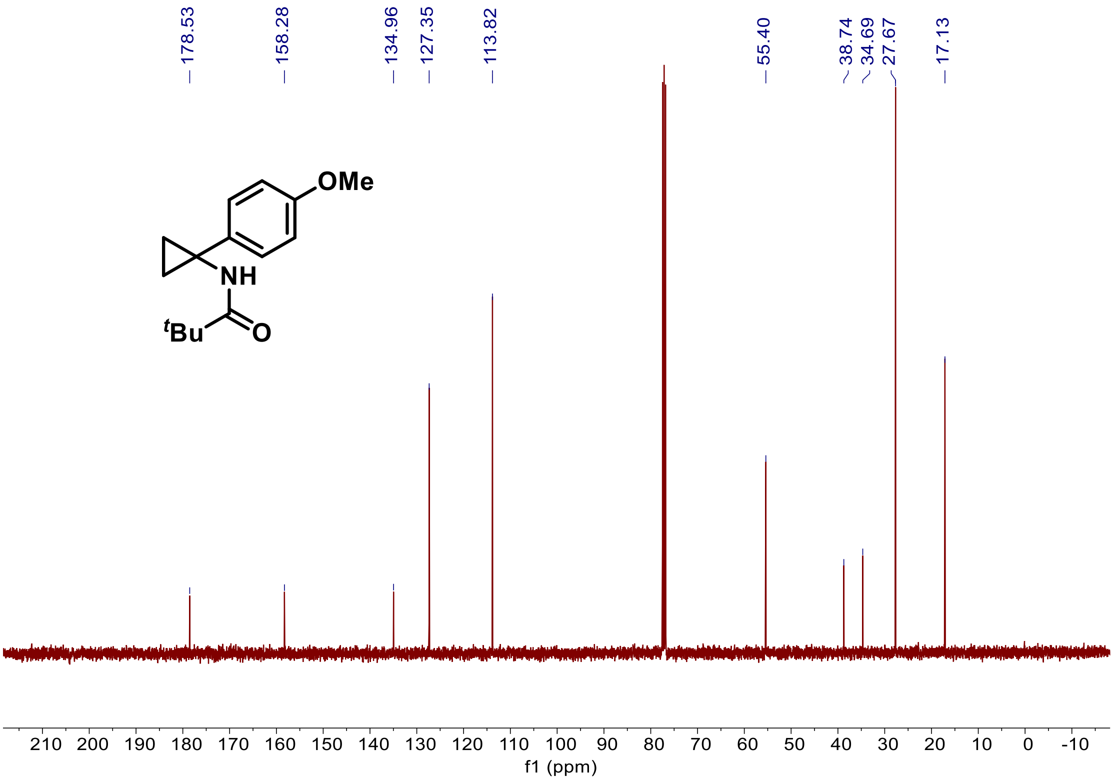

Supplementary Figure 18. $^{13}C\{^{1}H\}$ NMR spectrum of **1d** (101 MHz, Chloroform-*d*)

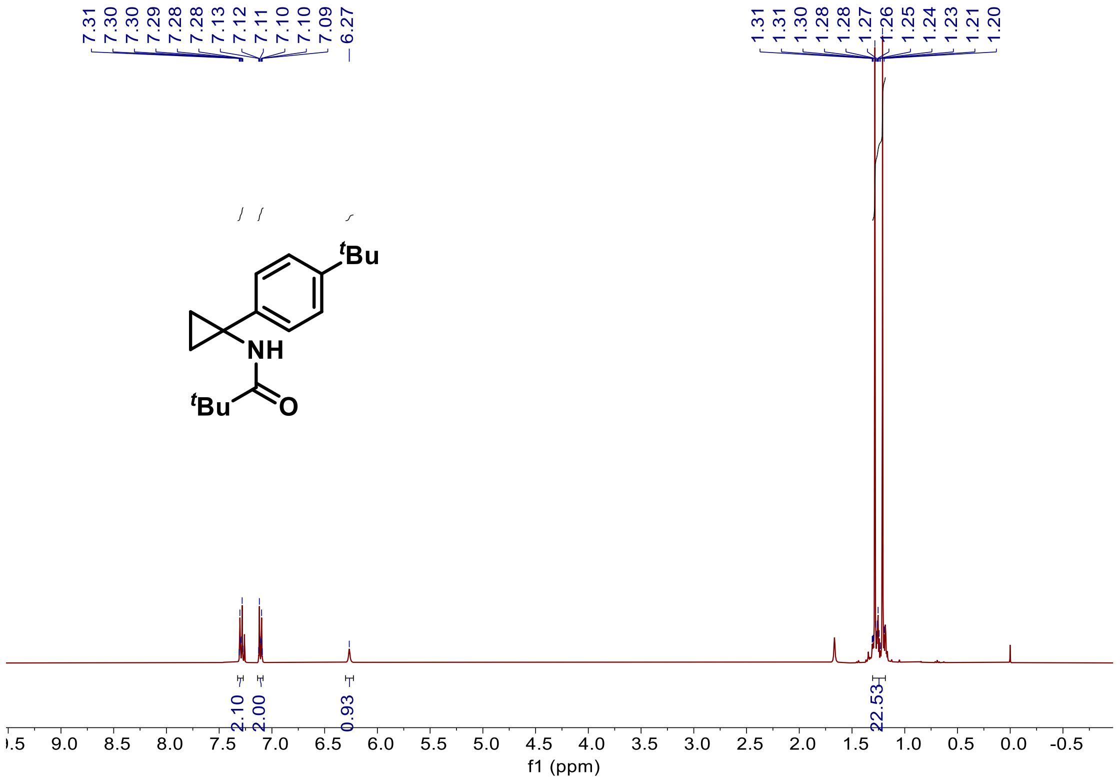

Supplementary Figure 19. ^{1}H NMR spectrum of **1e** (400 MHz, Chloroform-*d*)

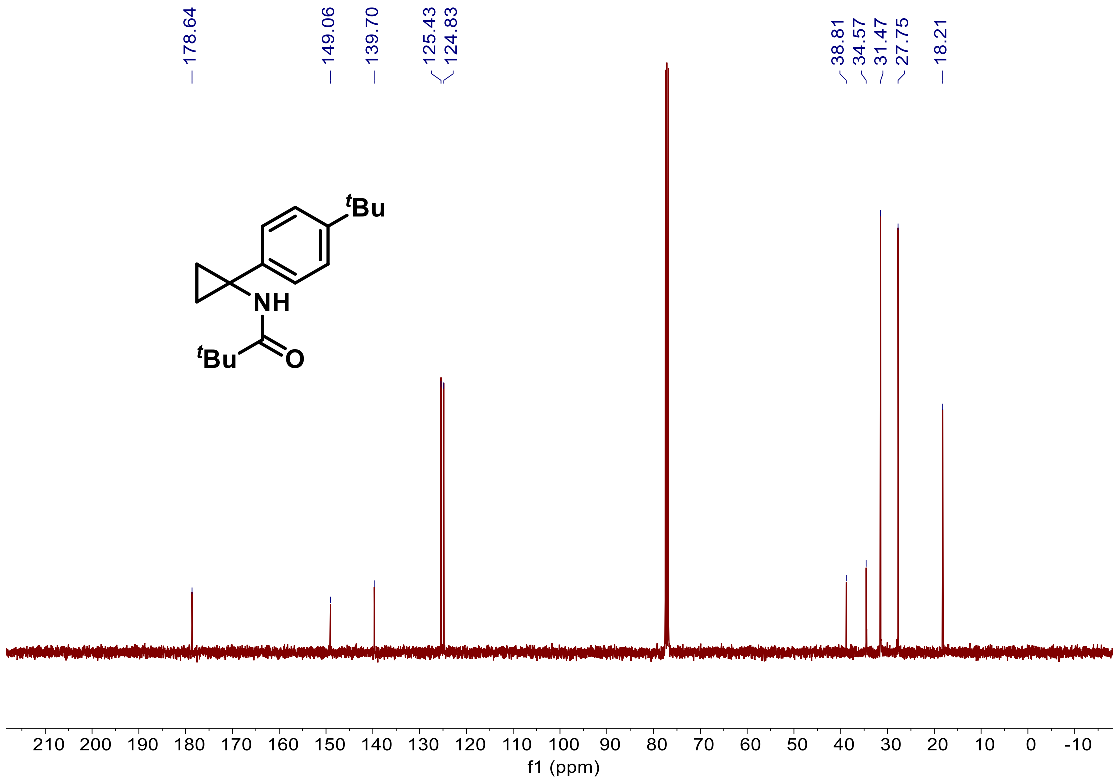

Supplementary Figure 20. $^{13}C\{^{1}H\}$ NMR spectrum of **1e** (101 MHz, Chloroform-*d*)

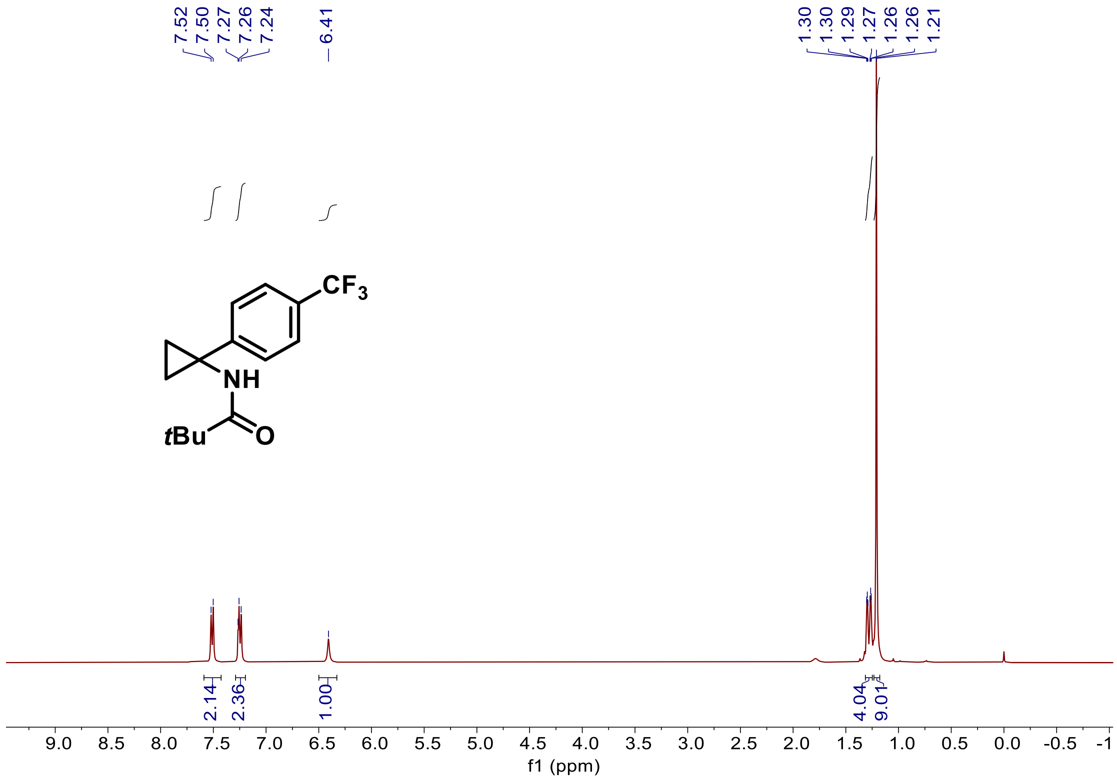

Supplementary Figure 21. ^{1}H NMR spectrum of **1f** (400 MHz, Chloroform-*d*)

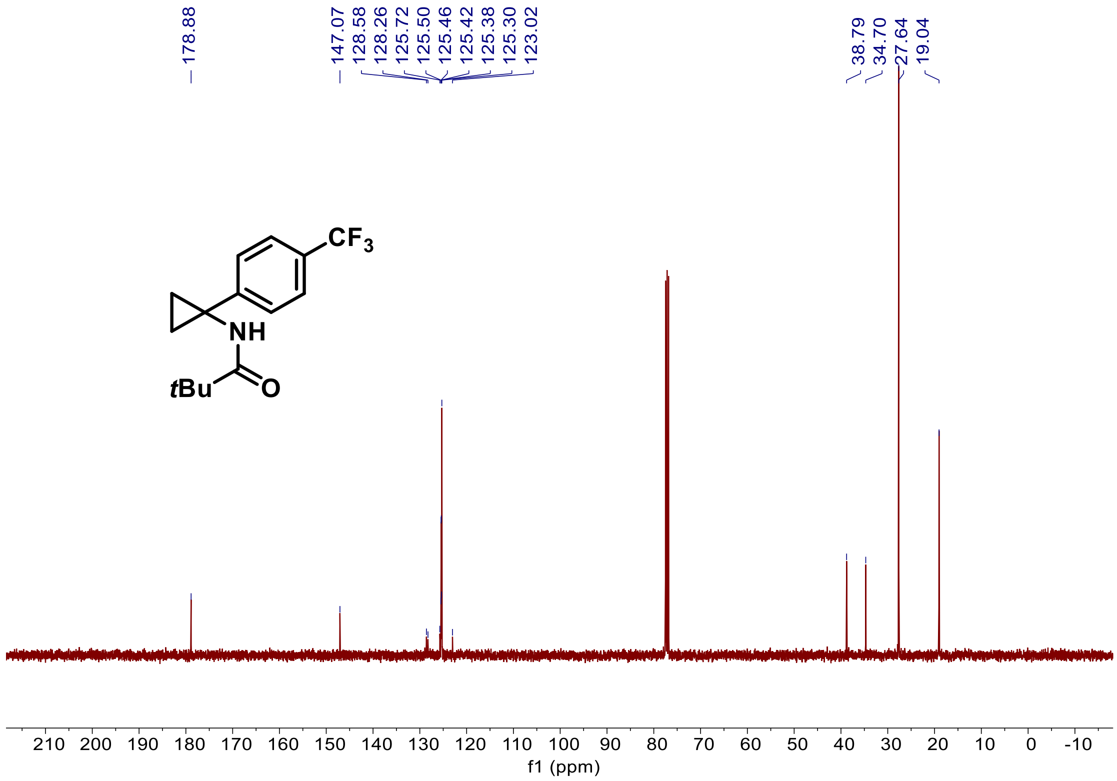

Supplementary Figure 22. ^{13}C{^{1}H} NMR spectrum of **1f** (101 MHz, Chloroform-*d*)

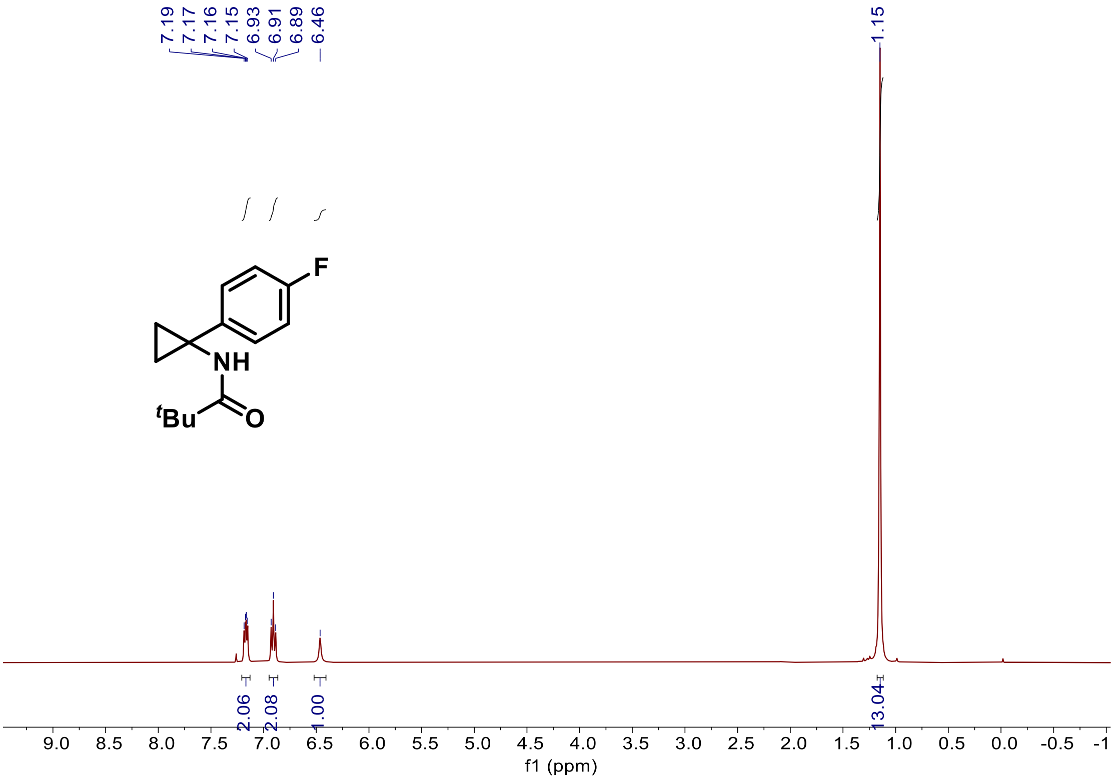

Supplementary Figure 23. ^{1}H NMR spectrum of **1g** (400 MHz, Chloroform-*d*)

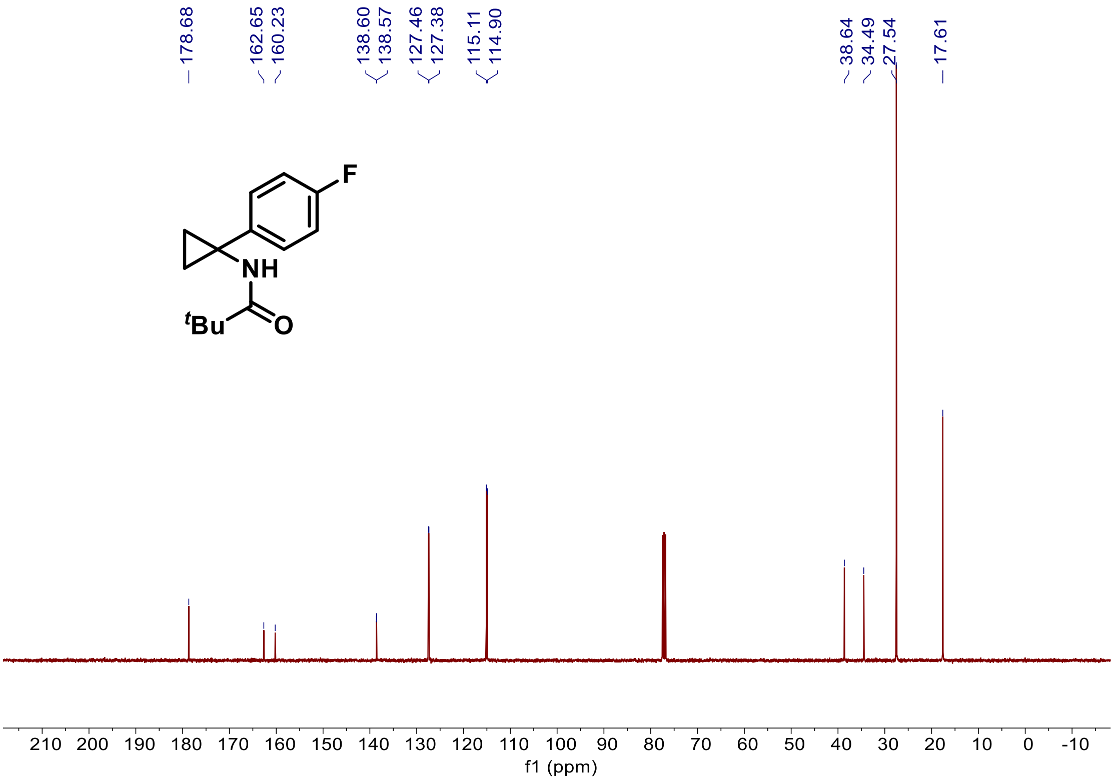

Supplementary Figure 24. ^{13}C{^{1}H} NMR spectrum of **1g** (101 MHz, Chloroform-*d*)

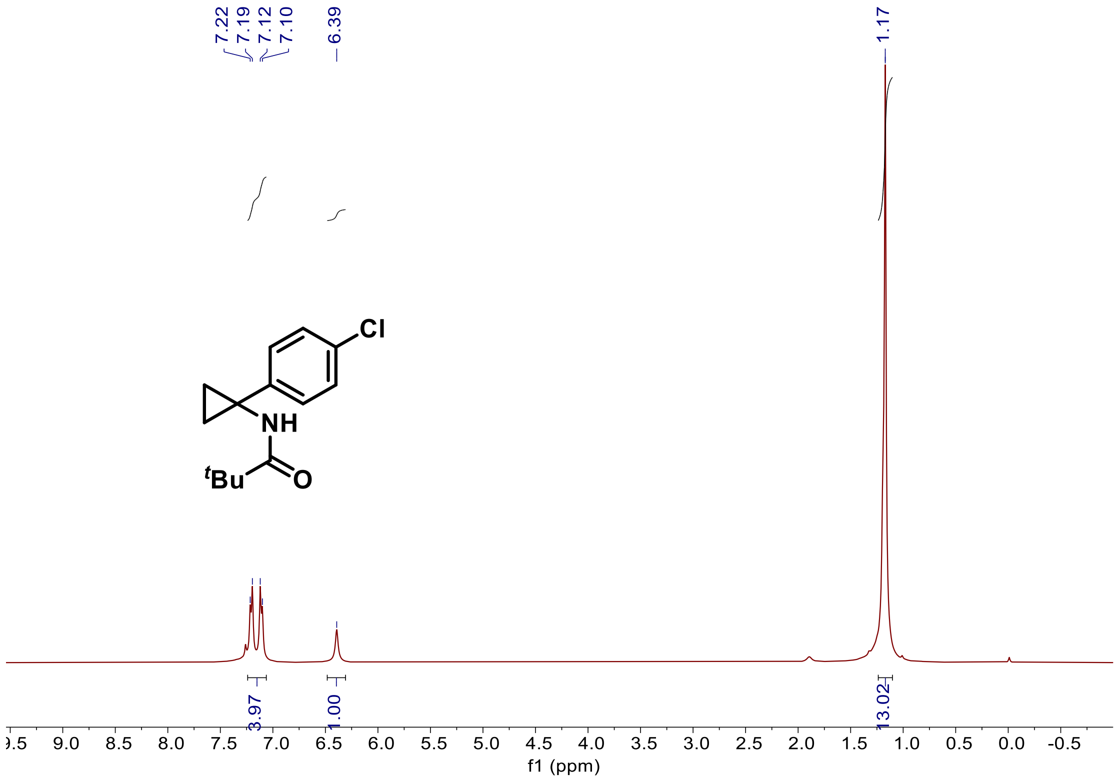

Supplementary Figure 25. ^{1}H NMR spectrum of **1h** (400 MHz, Chloroform-*d*)

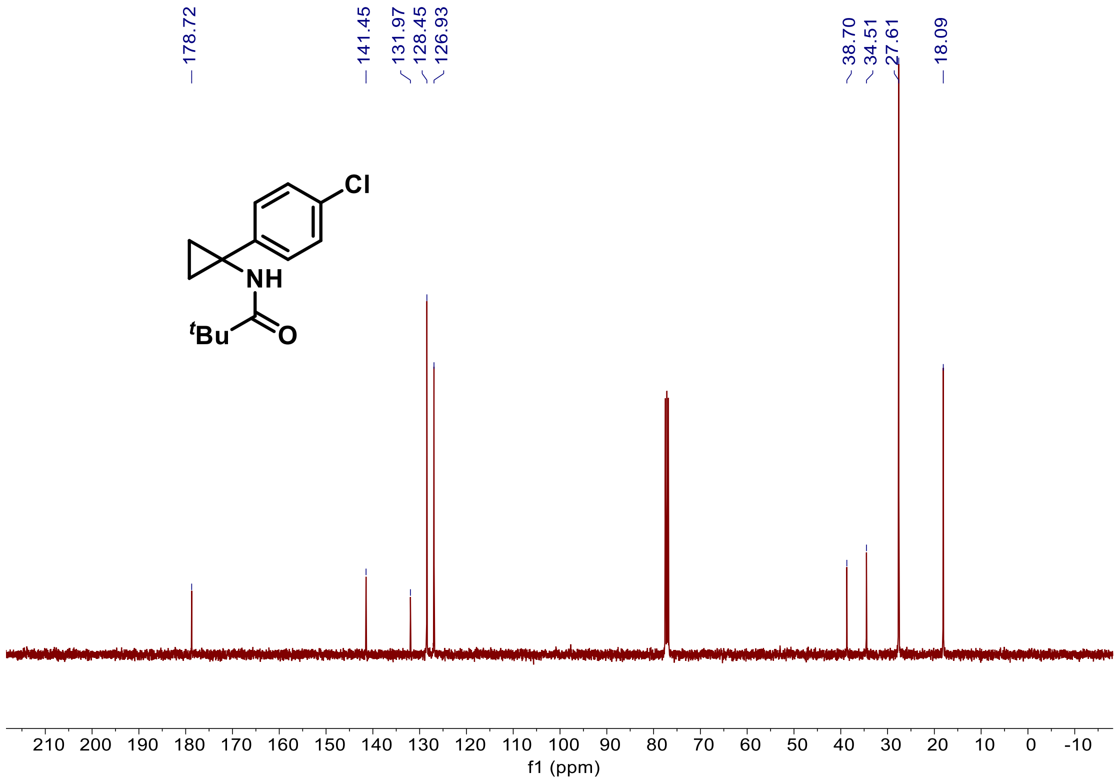

Supplementary Figure 26. $^{13}C\{^{1}H\}$ NMR spectrum of **1h** (101 MHz, Chloroform-*d*)

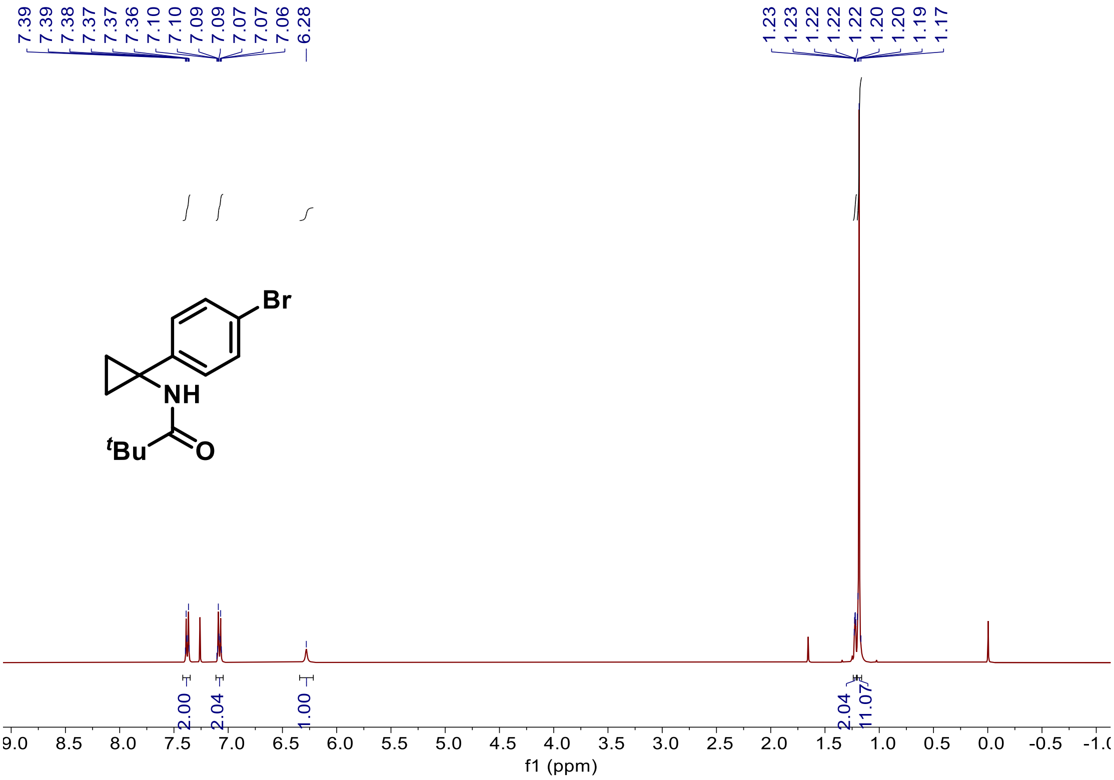

Supplementary Figure 27. ^{1}H NMR spectrum of **1i** (400 MHz, Chloroform-*d*)

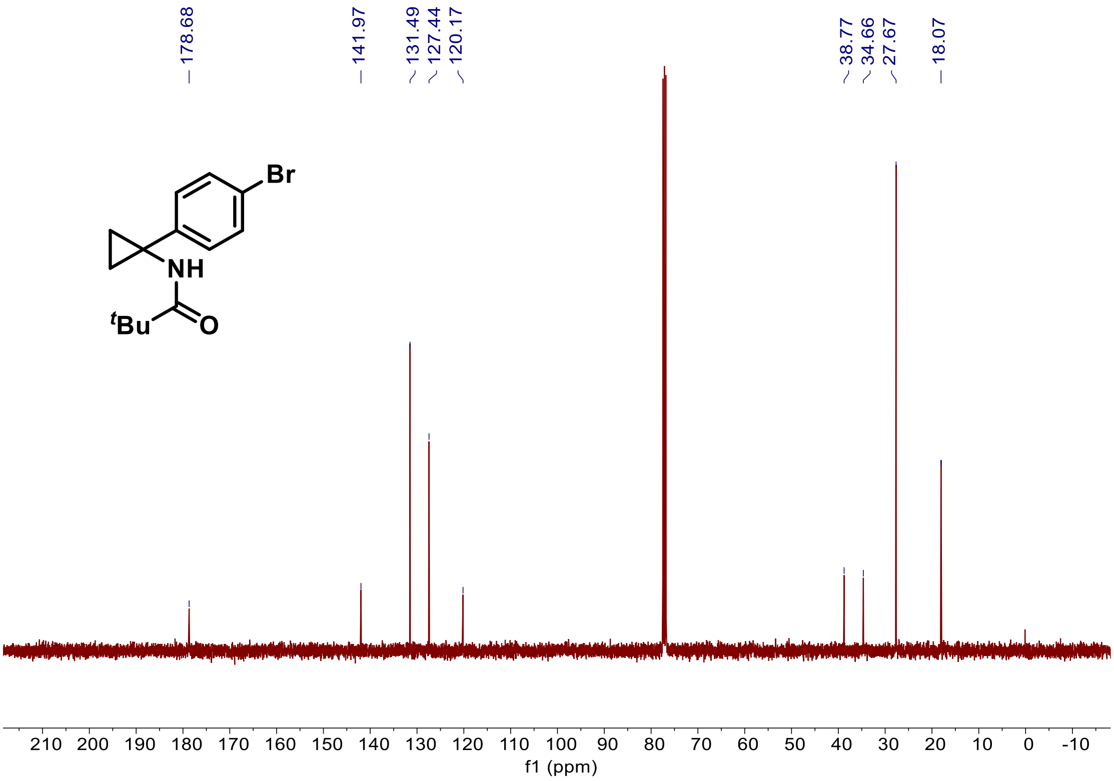

Supplementary Figure 28. $^{13}C\{^{1}H\}$ NMR spectrum of **1i** (101 MHz, Chloroform-*d*)

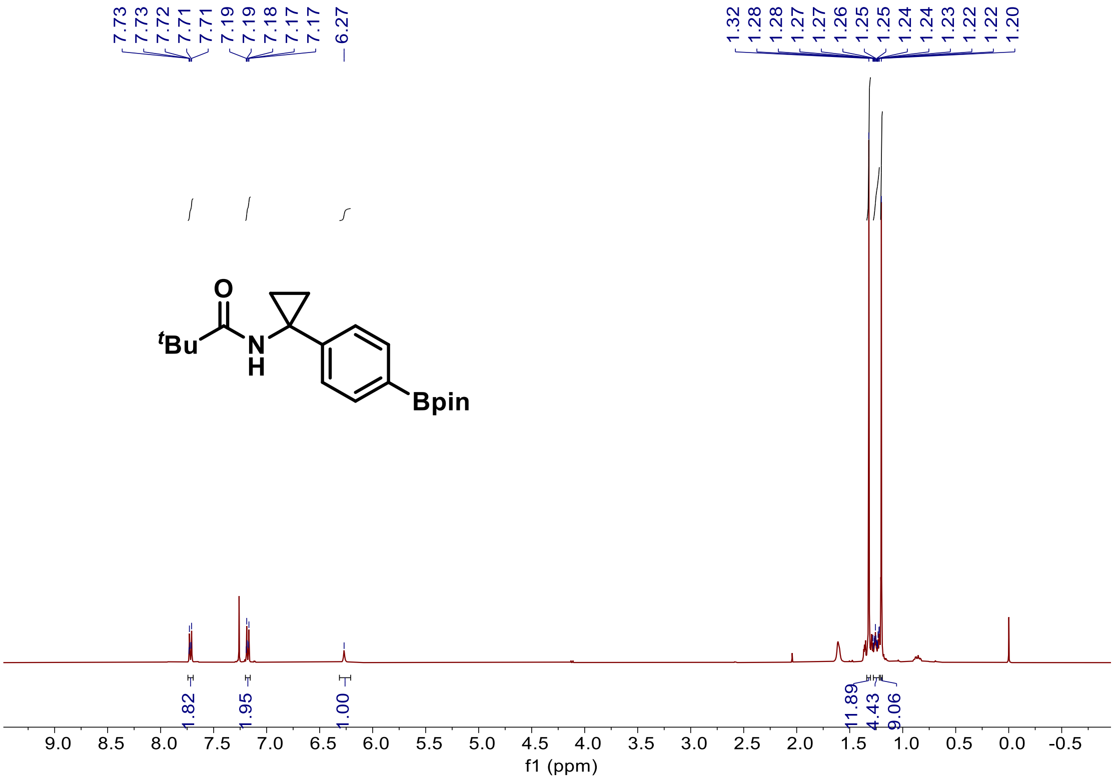

Supplementary Figure 29. ^{1}H NMR spectrum of **1j** (400 MHz, Chloroform-*d*)

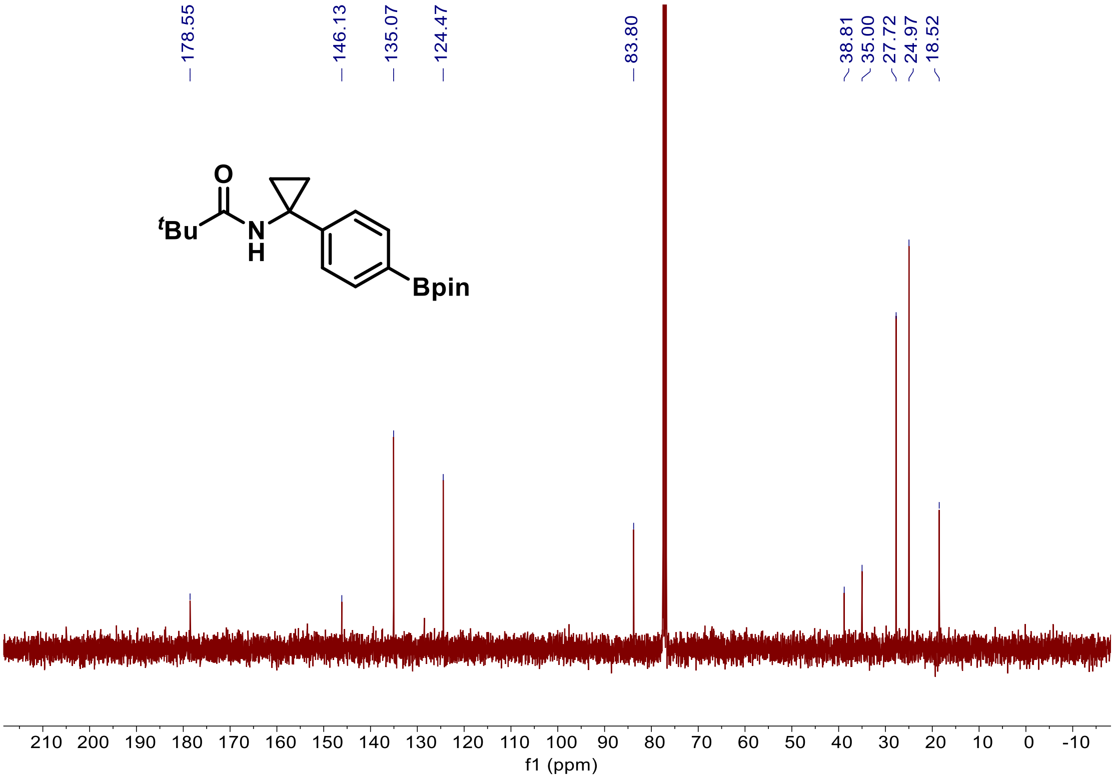

Supplementary Figure 30. ^{13}C{^{1}H} NMR spectrum of **1j** (101 MHz, Chloroform-*d*)

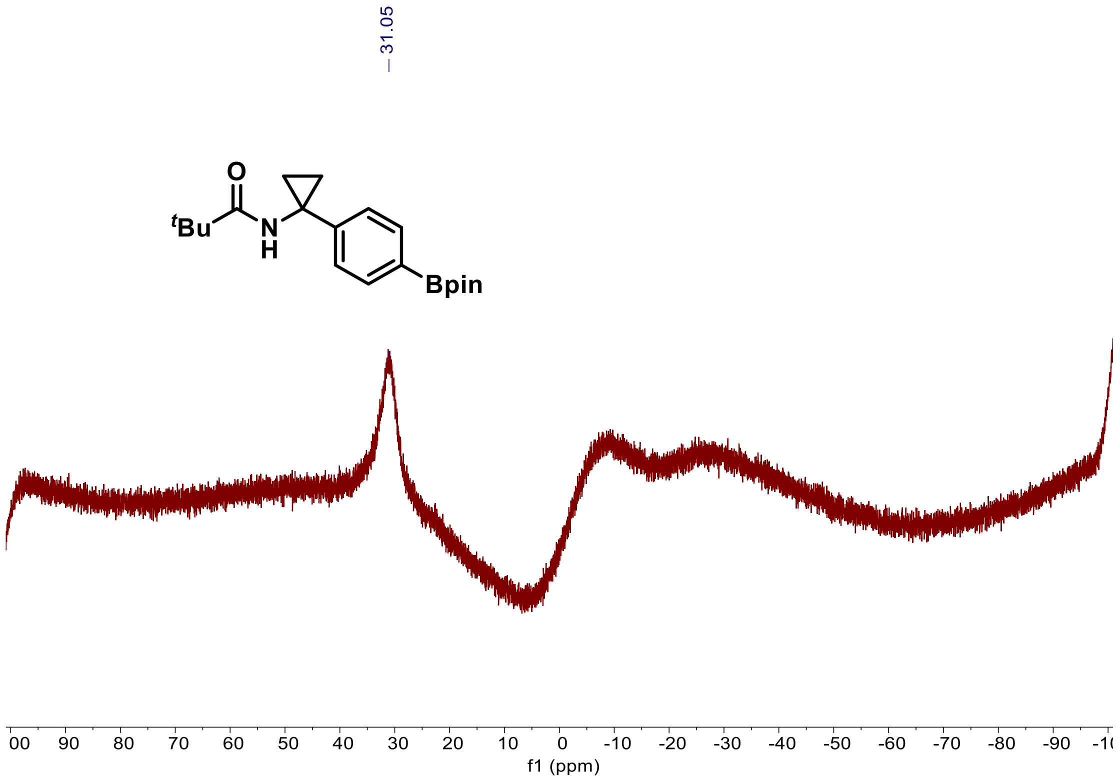

Supplementary Figure 31. ^{11}B NMR spectrum of **1j** (128 MHz, Chloroform-*d*)

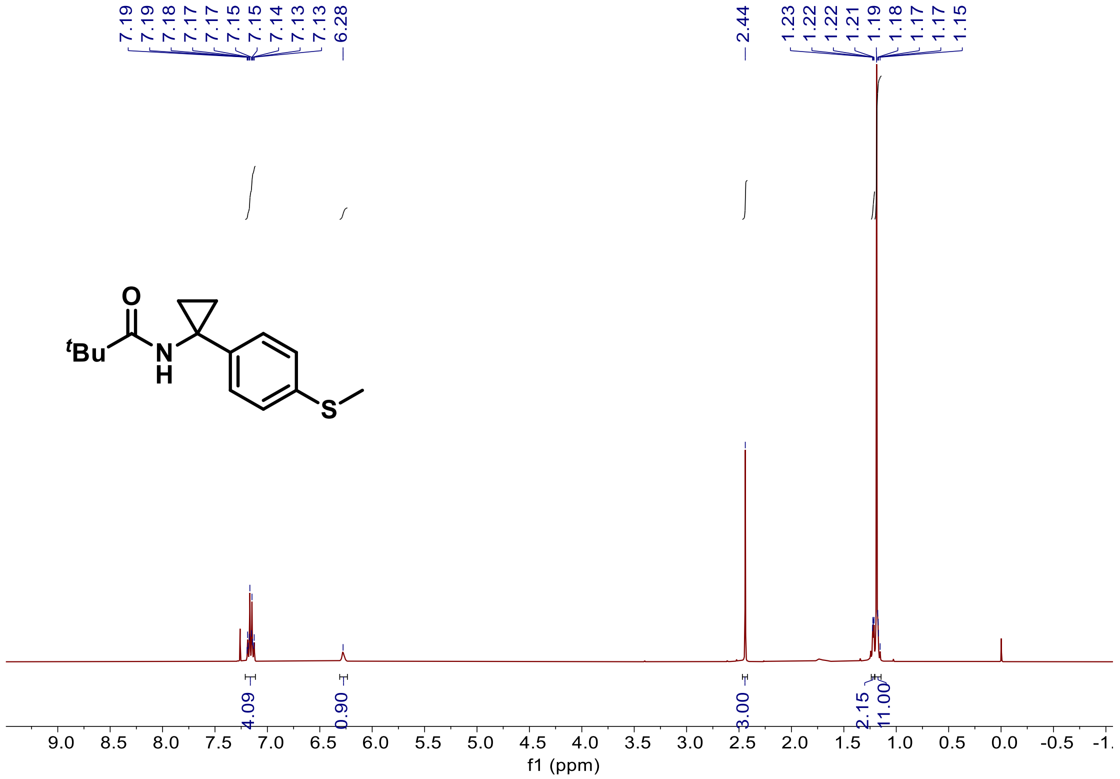

Supplementary Figure 32. ^{1}H NMR spectrum of **1k** (400 MHz, Chloroform-*d*)

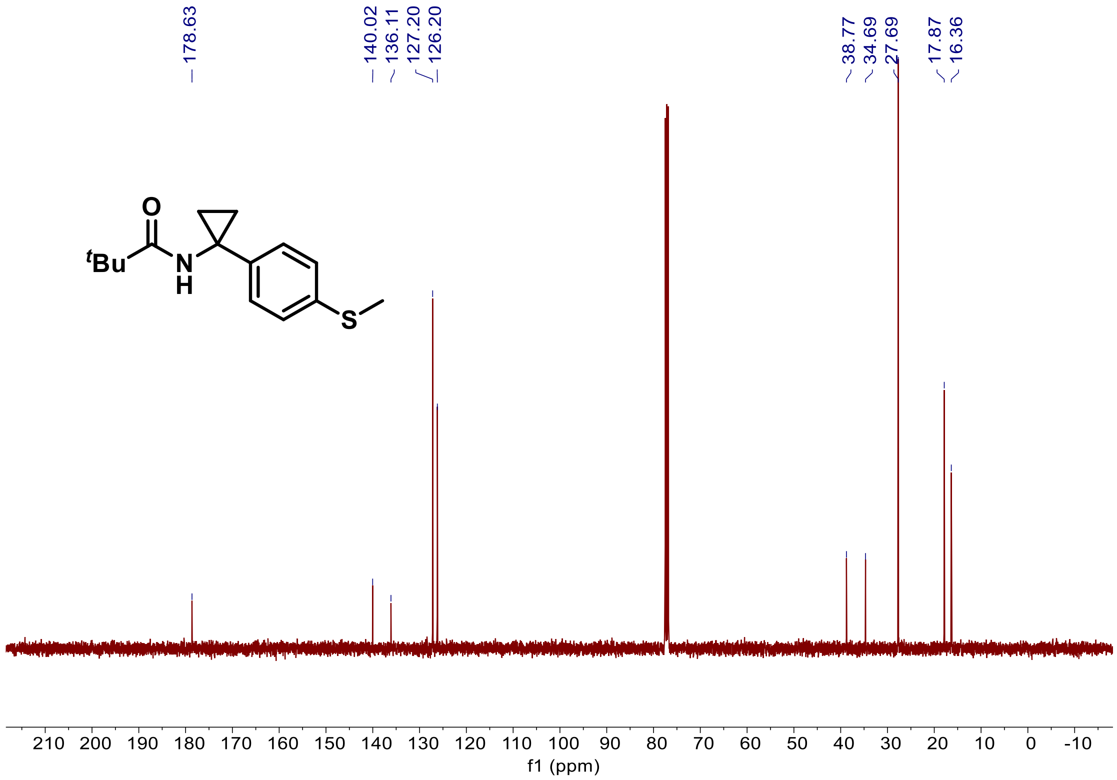

Supplementary Figure 33. $^{13}C\{^{1}H\}$ NMR spectrum of **1k** (101 MHz, Chloroform-*d*)

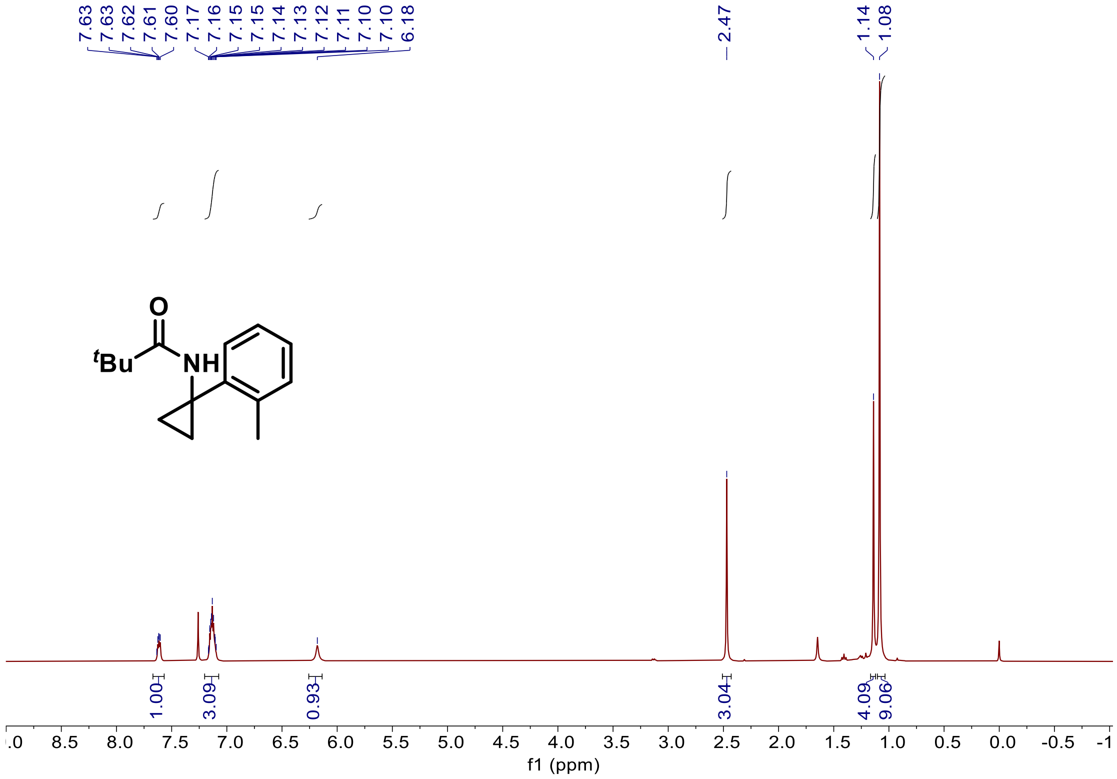

Supplementary Figure 34. ^{1}H NMR spectrum of **1l** (400 MHz, Chloroform-*d*)

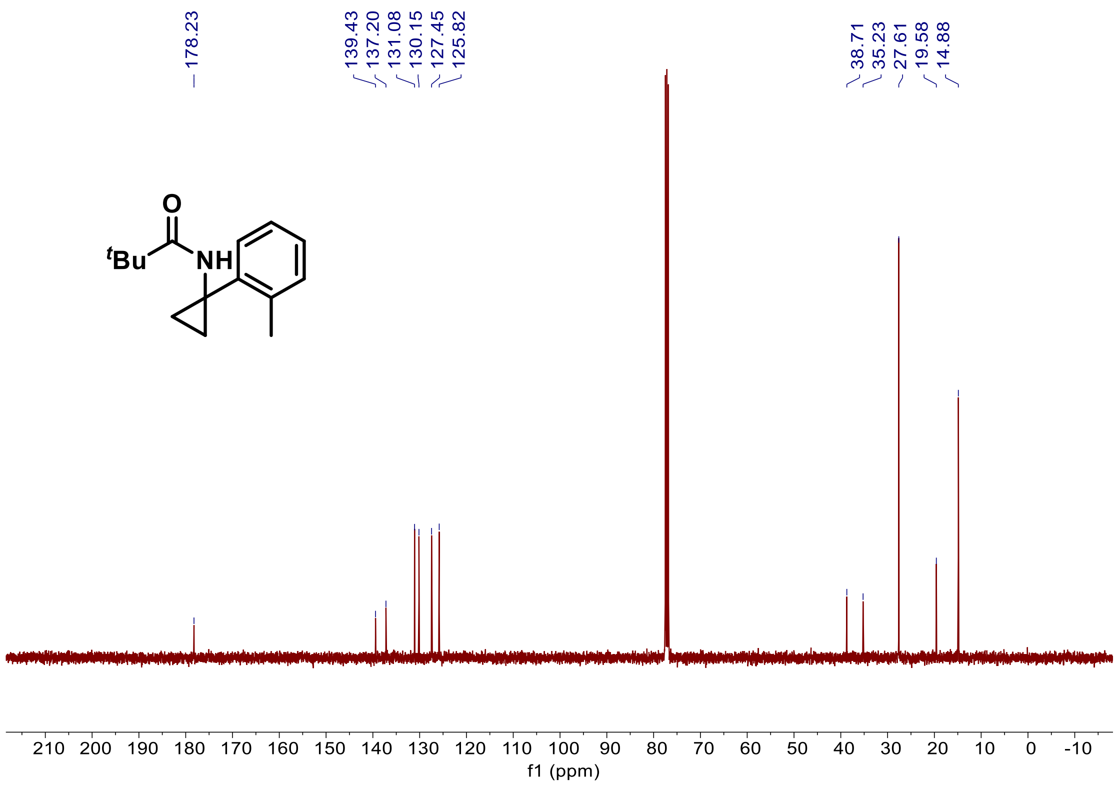

Supplementary Figure 35. $^{13}C\{^{1}H\}$ NMR spectrum of **1l** (101 MHz, Chloroform-*d*)

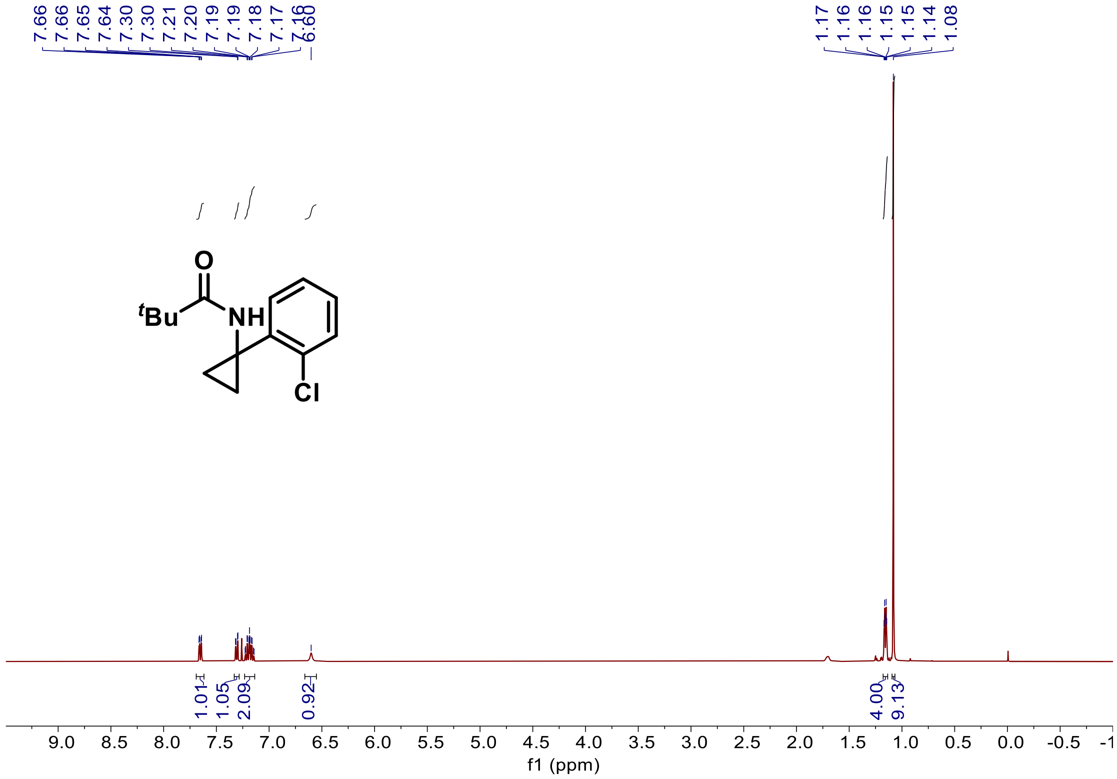

Supplementary Figure 36. ^{1}H NMR spectrum of **1m** (400 MHz, Chloroform-*d*)

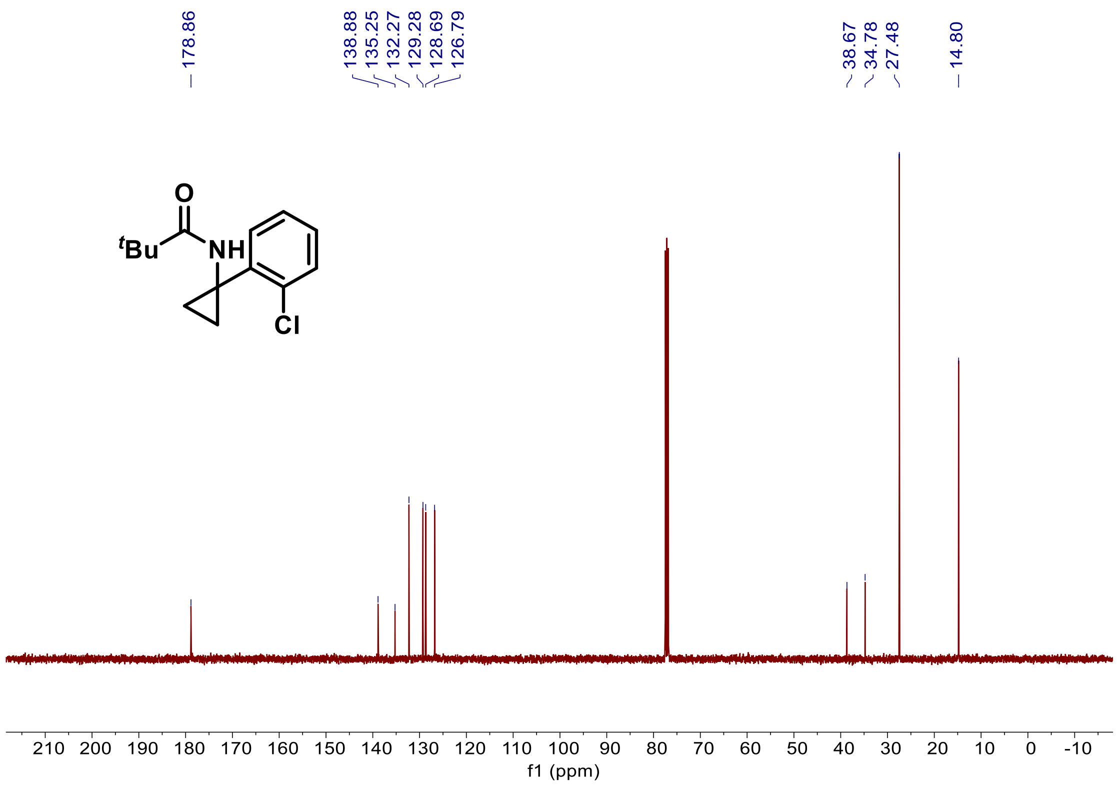

Supplementary Figure 37. $^{13}C\{^{1}H\}$ NMR spectrum of **1m** (101 MHz, Chloroform-*d*)

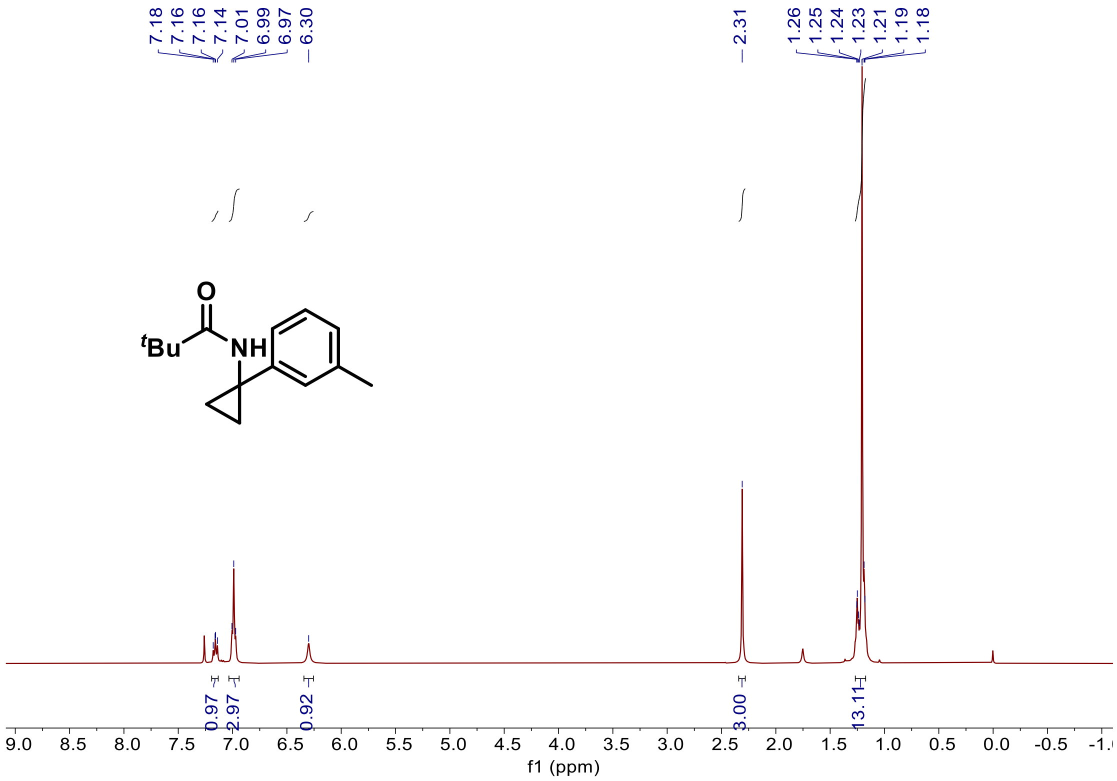

Supplementary Figure 38. ^{1}H NMR spectrum of **1n** (400 MHz, Chloroform-*d*)

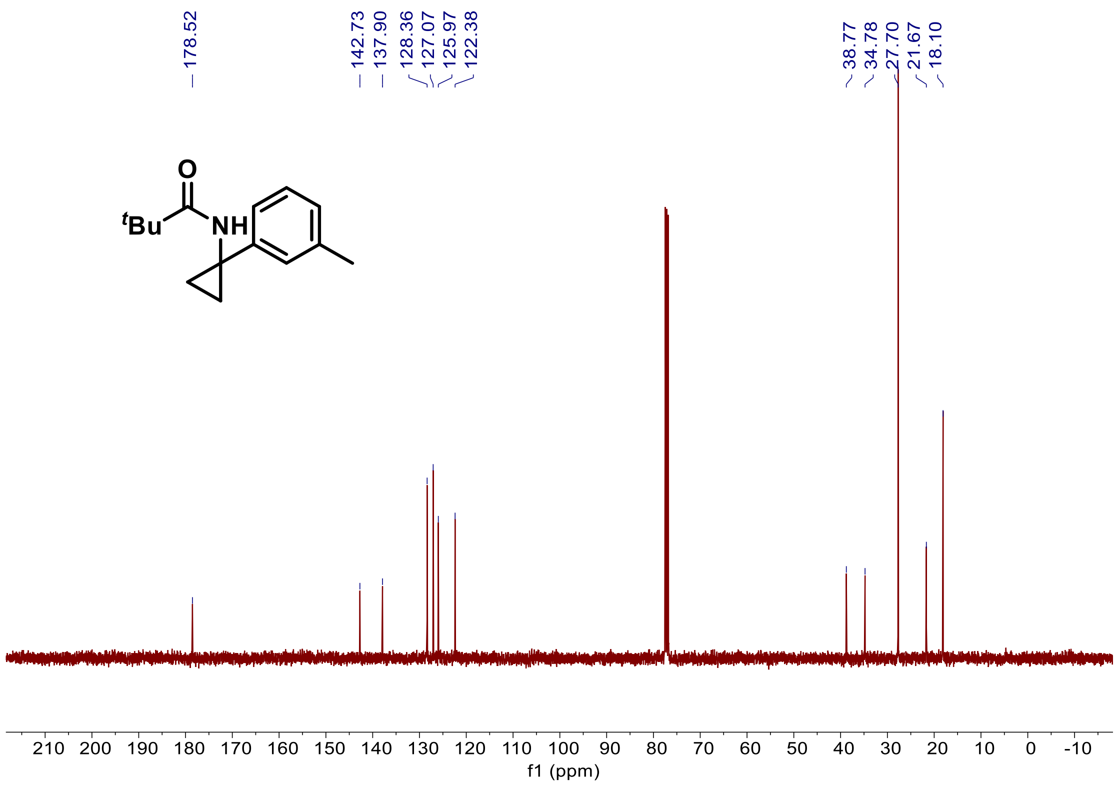

Supplementary Figure 39. $^{13}C\{^{1}H\}$ NMR spectrum of **1n** (101 MHz, Chloroform-*d*)

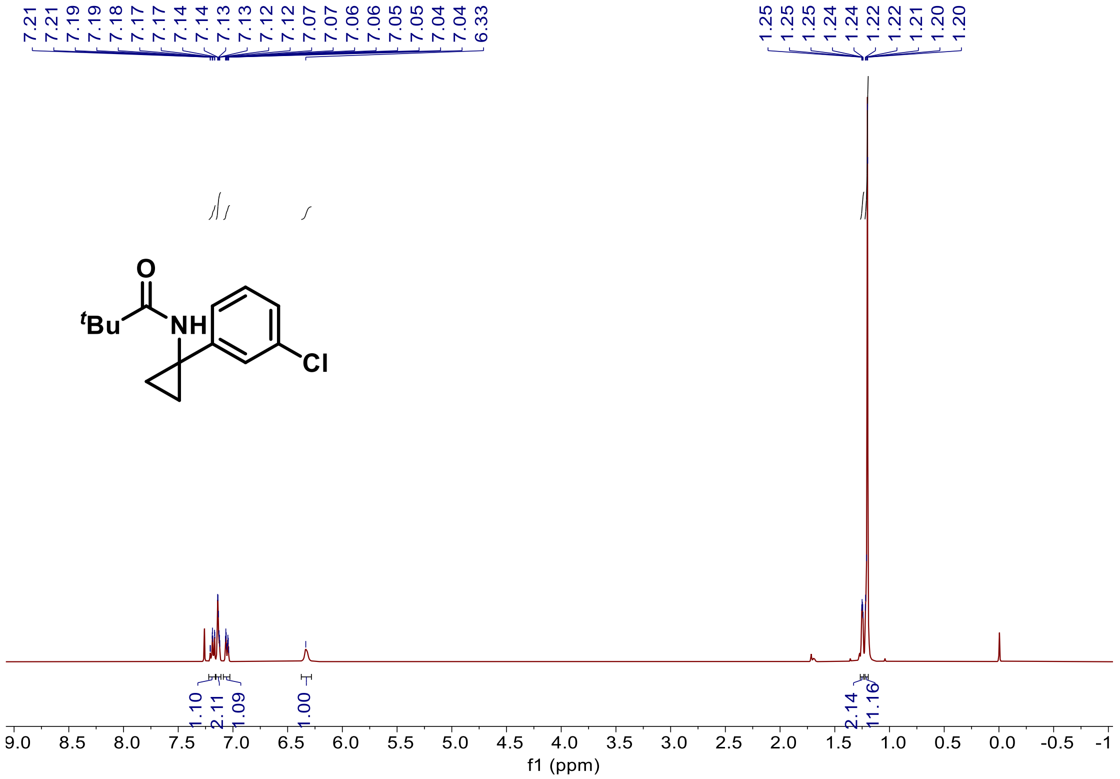

Supplementary Figure 40. ^{1}H NMR spectrum of **1o** (400 MHz, Chloroform-*d*)

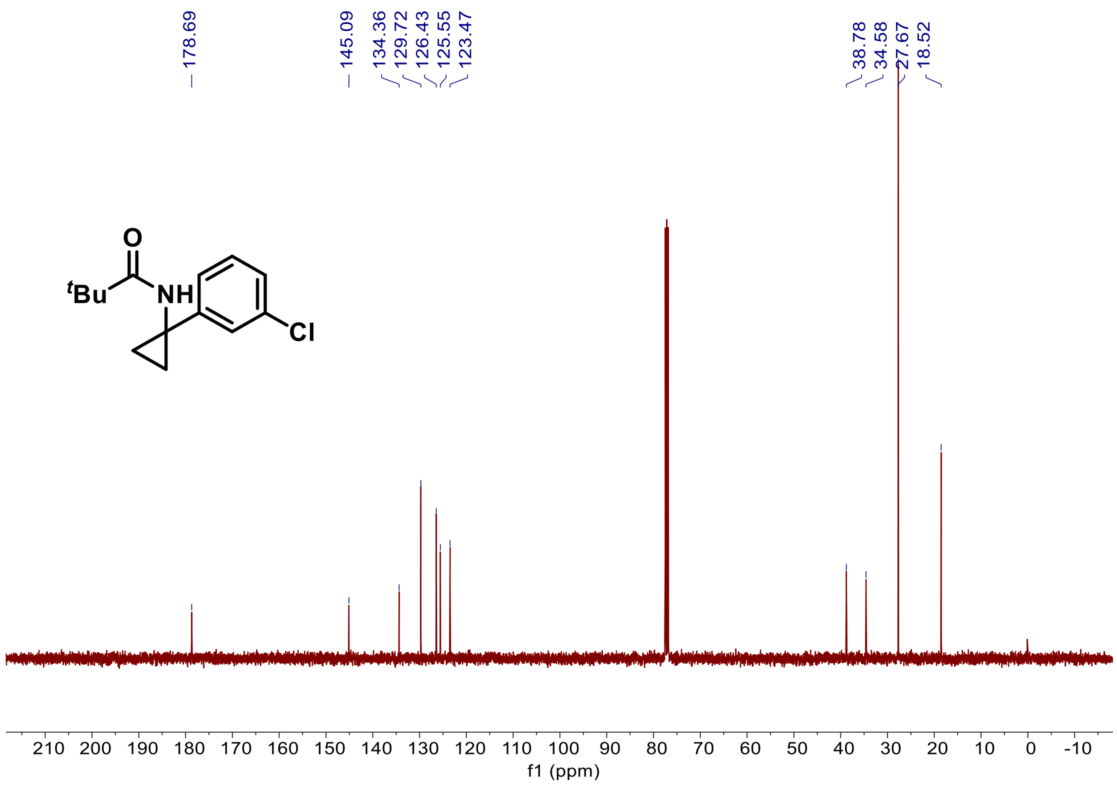

Supplementary Figure 41. $^{13}C\{^{1}H\}$ NMR spectrum of **1o** (101 MHz, Chloroform-*d*)

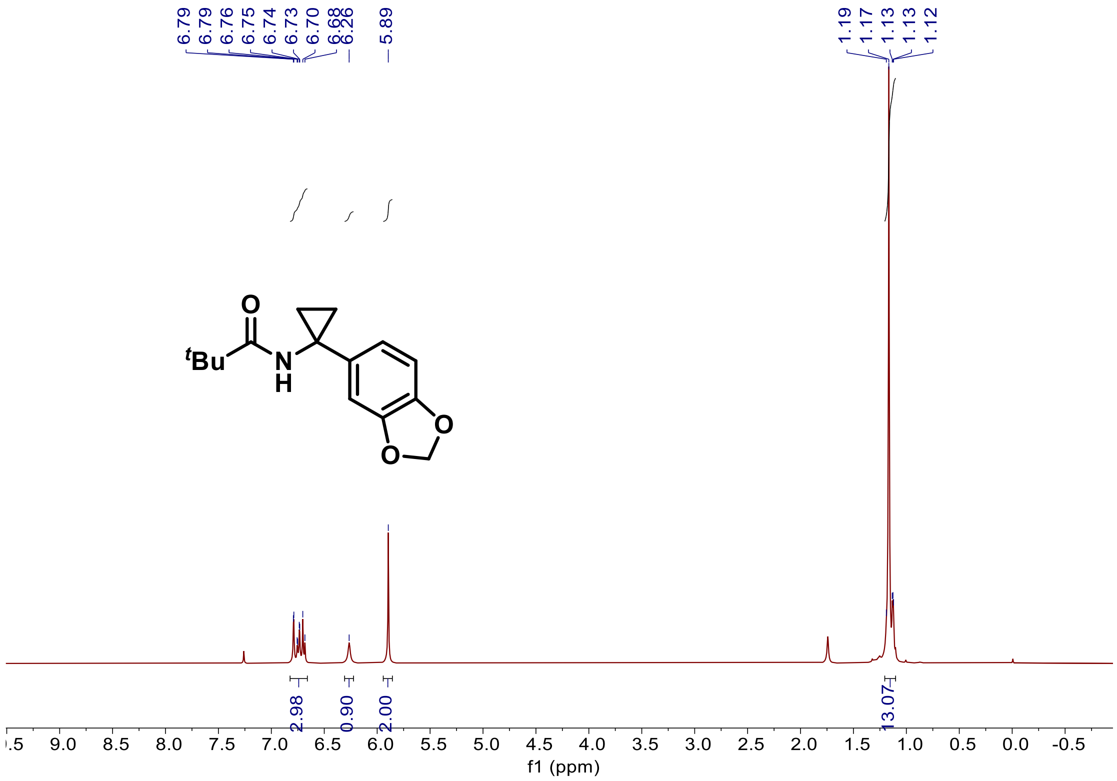

Supplementary Figure 42. ^{1}H NMR spectrum of **1p** (400 MHz, Chloroform-*d*)

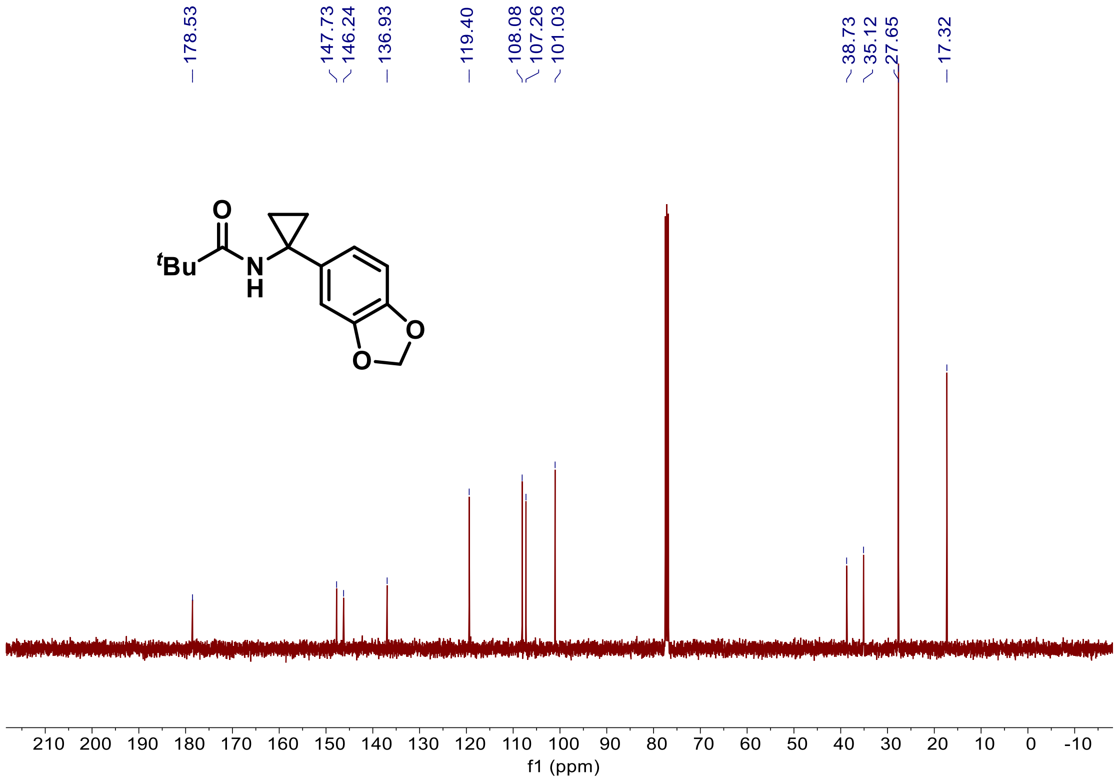

Supplementary Figure 43. $^{13}C\{^1H\}$ NMR spectrum of **1p** (101 MHz, Chloroform-*d*)

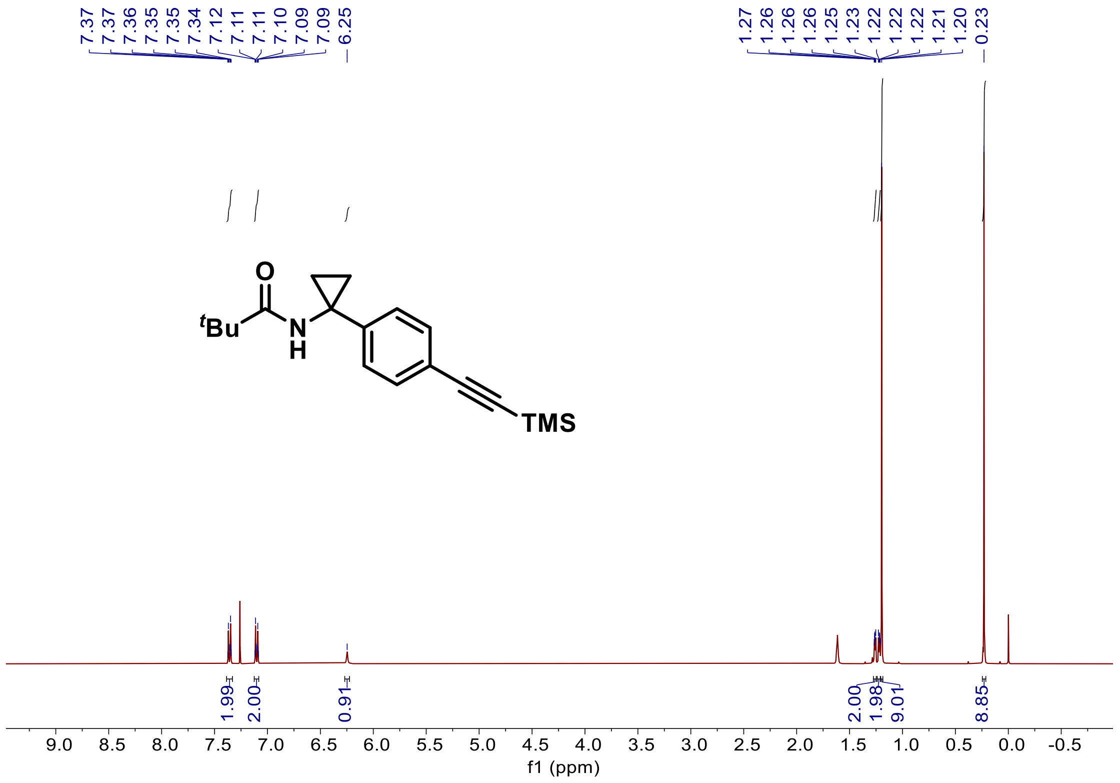

Supplementary Figure 44. ^1H NMR spectrum of **1q** (400 MHz, Chloroform-*d*)

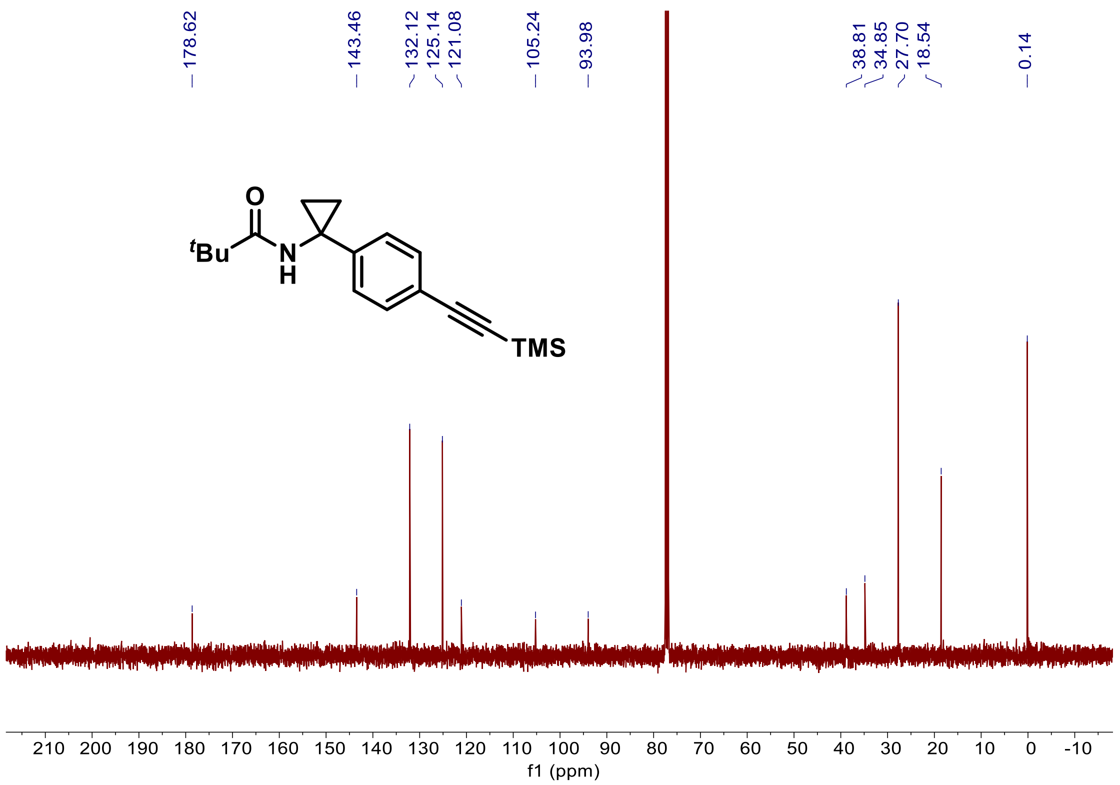

Supplementary Figure 45. $^{13}C\{^{1}H\}$ NMR spectrum of **1q** (101 MHz, Chloroform-*d*)

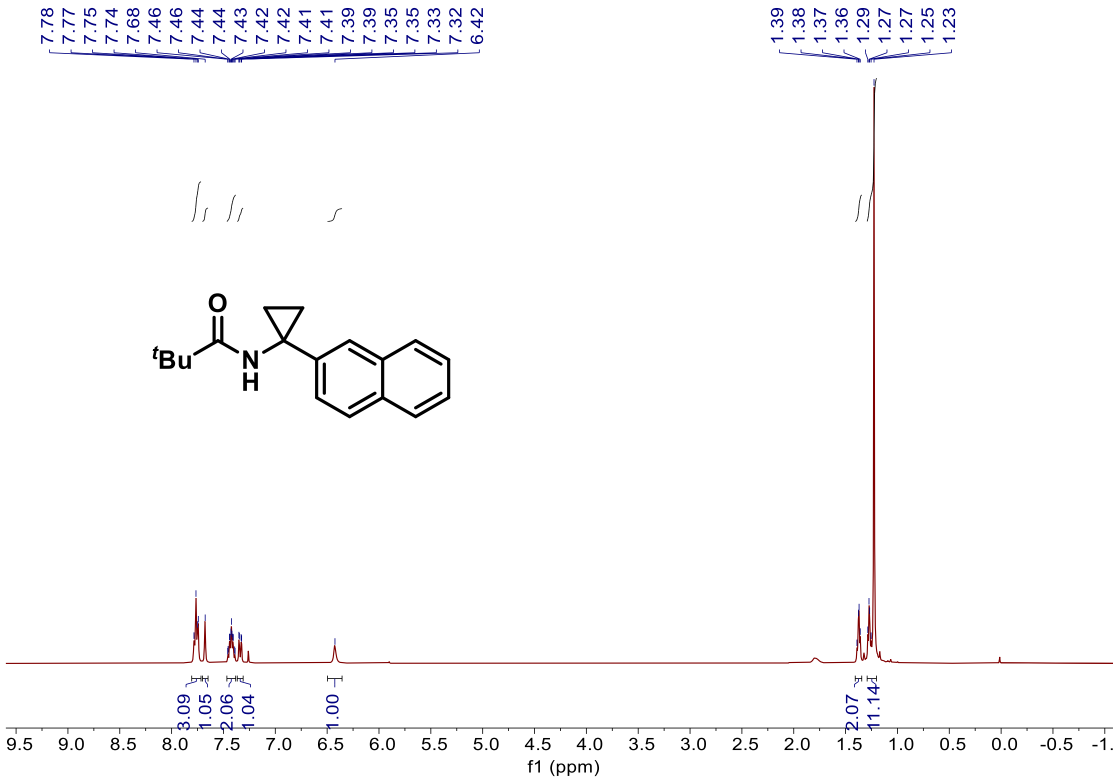

Supplementary Figure 46. ^{1}H NMR spectrum of **1r** (400 MHz, Chloroform-*d*)

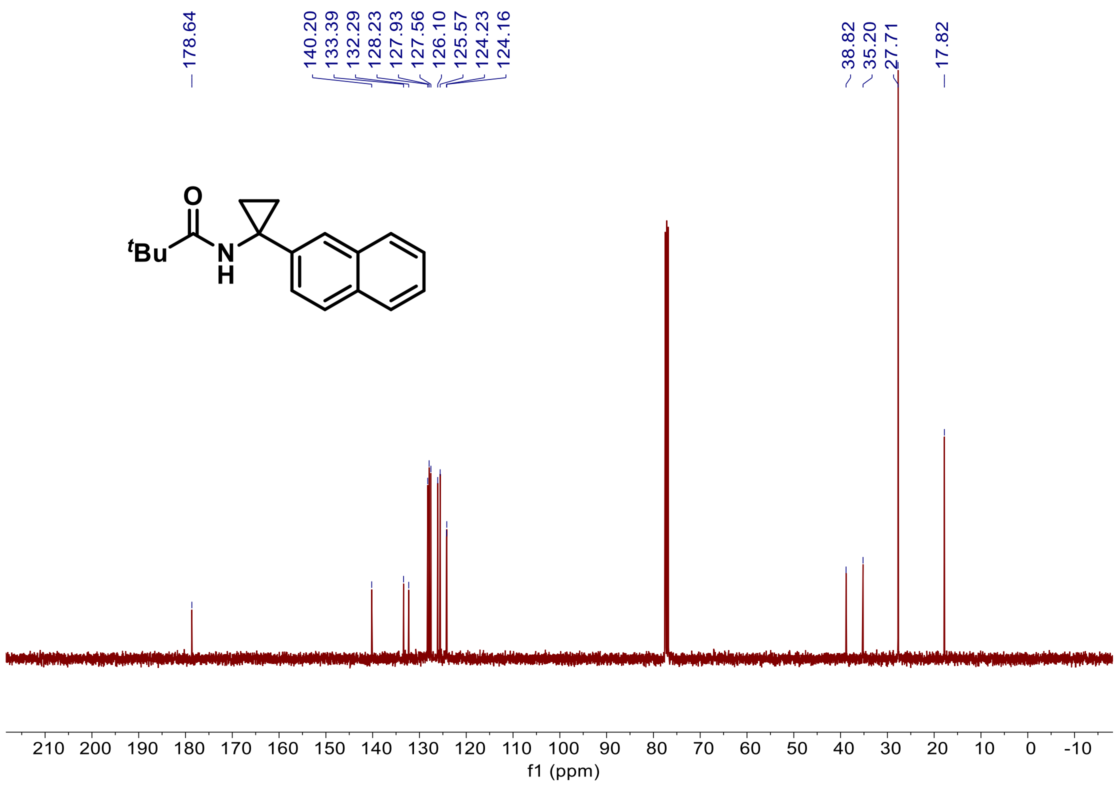

Supplementary Figure 47. $^{13}C\{^{1}H\}$ NMR spectrum of **1r** (101 MHz, Chloroform-*d*)

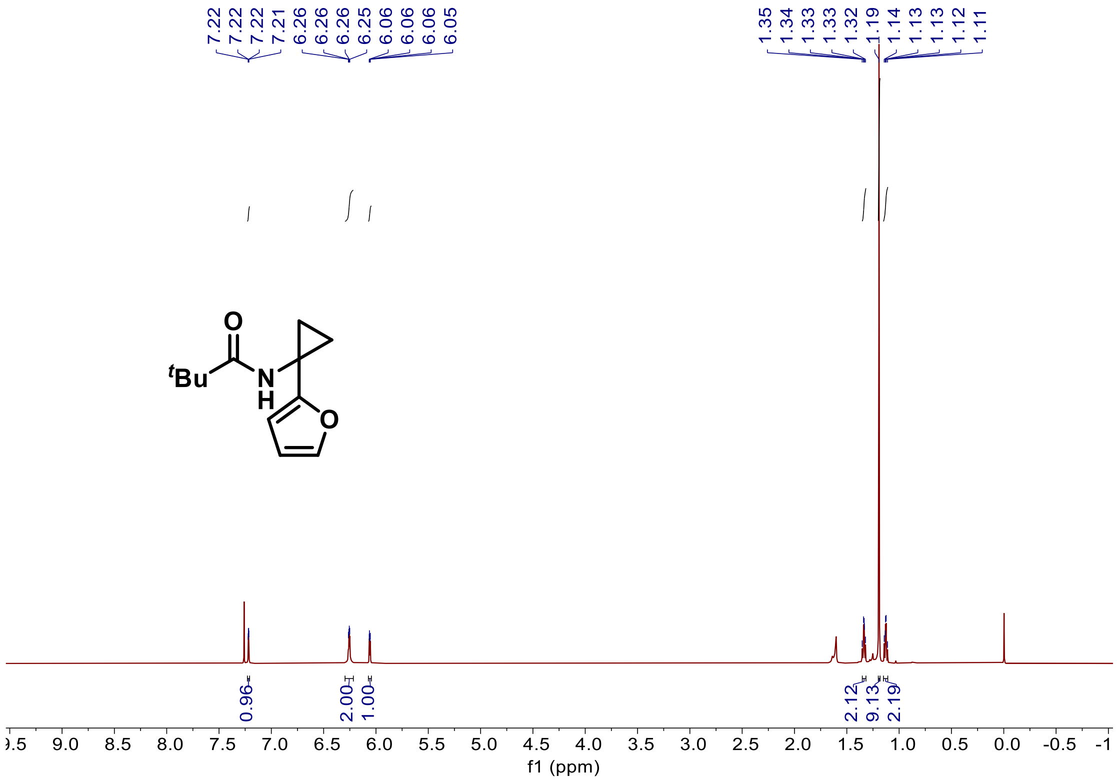

Supplementary Figure 48. ^{1}H NMR spectrum of **1s** (400 MHz, Chloroform-*d*)

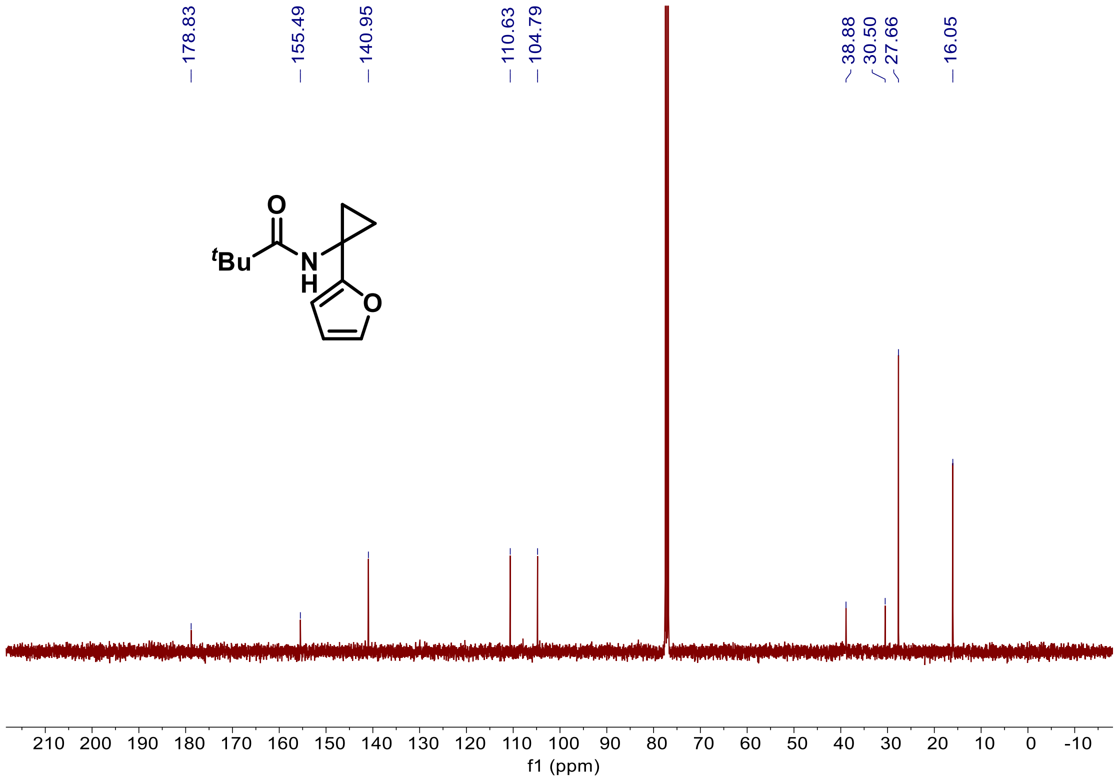

Supplementary Figure 49. $^{13}C\{^1H\}$ NMR spectrum of **1s** (101 MHz, Chloroform-*d*)

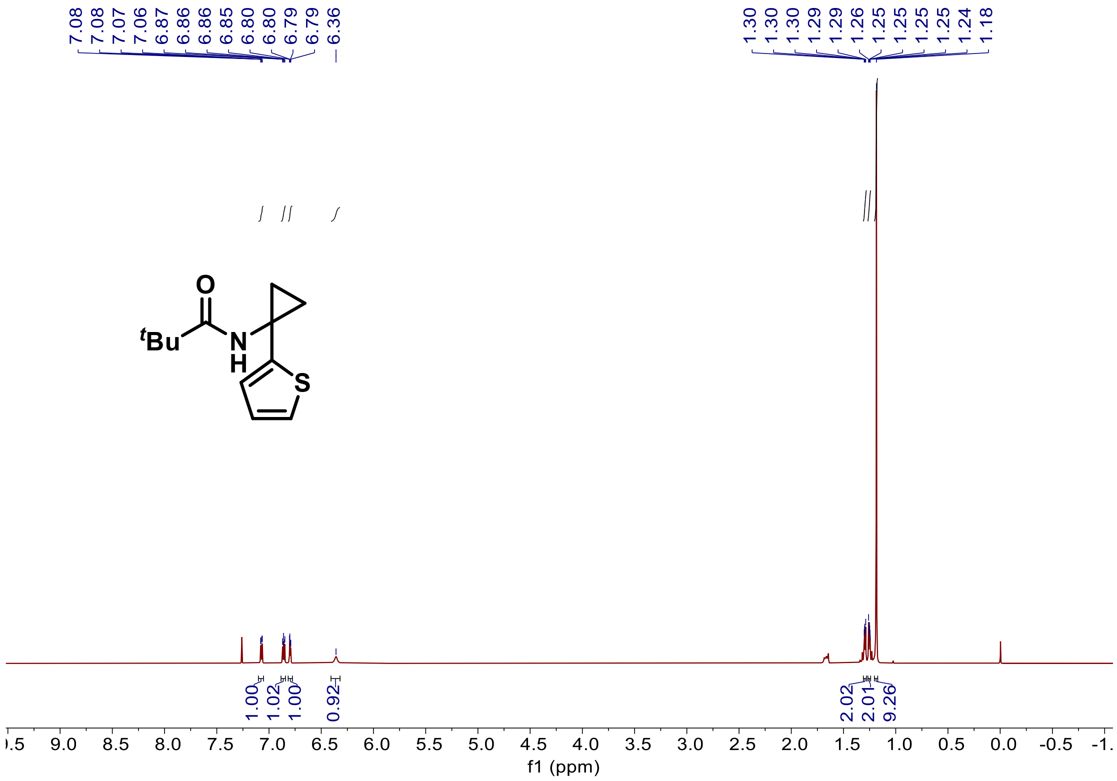

Supplementary Figure 50. ^1H NMR spectrum of **1t** (400 MHz, Chloroform-*d*)

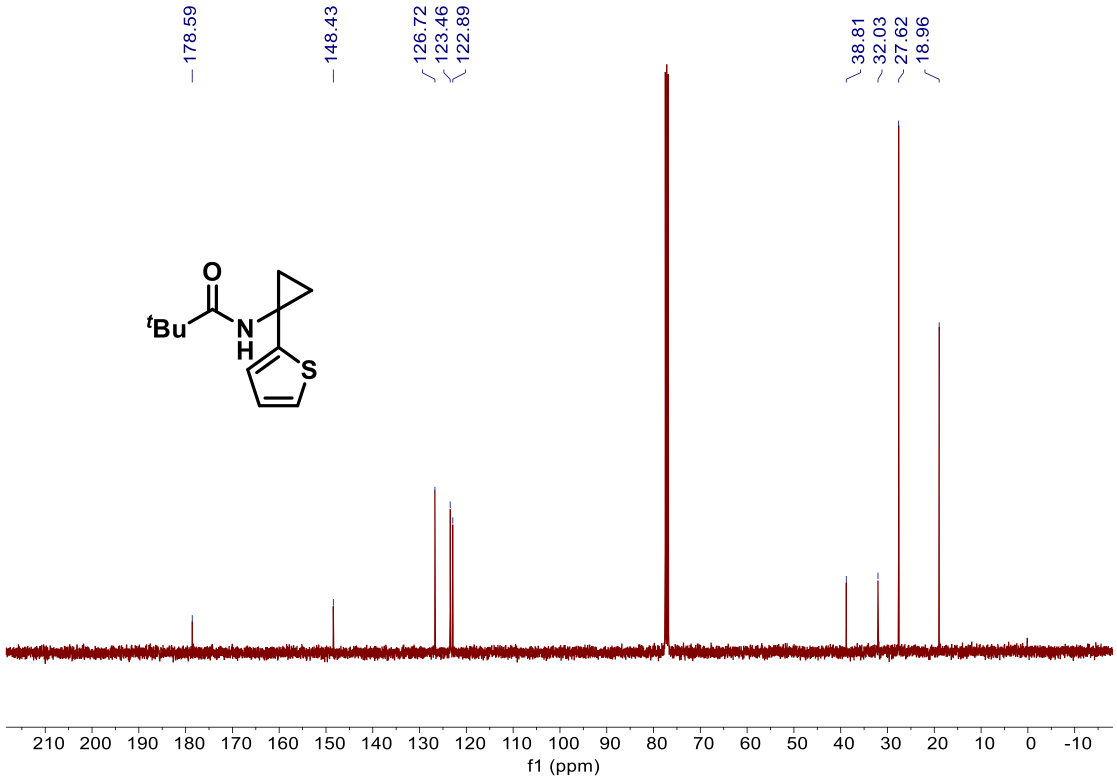

Supplementary Figure 51. $^{13}C\{^{1}H\}$ NMR spectrum of **1t** (101 MHz, Chloroform-*d*)

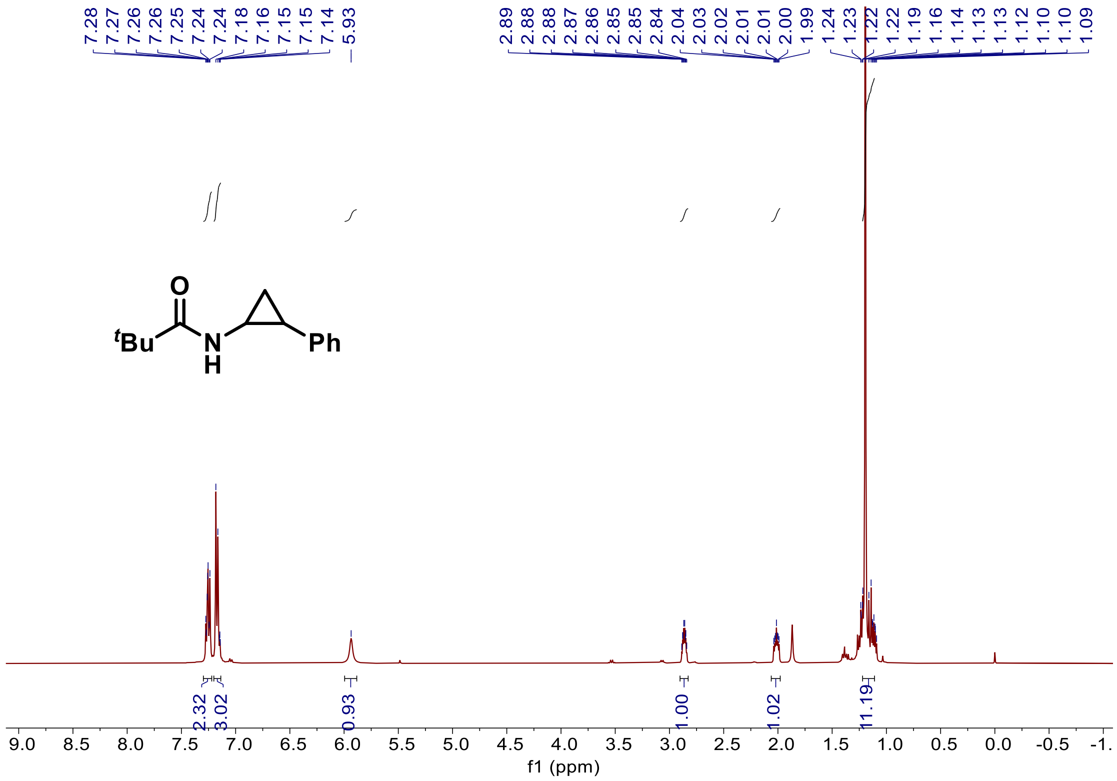

Supplementary Figure 52. ^{1}H NMR spectrum of **1u** (400 MHz, Chloroform-*d*)

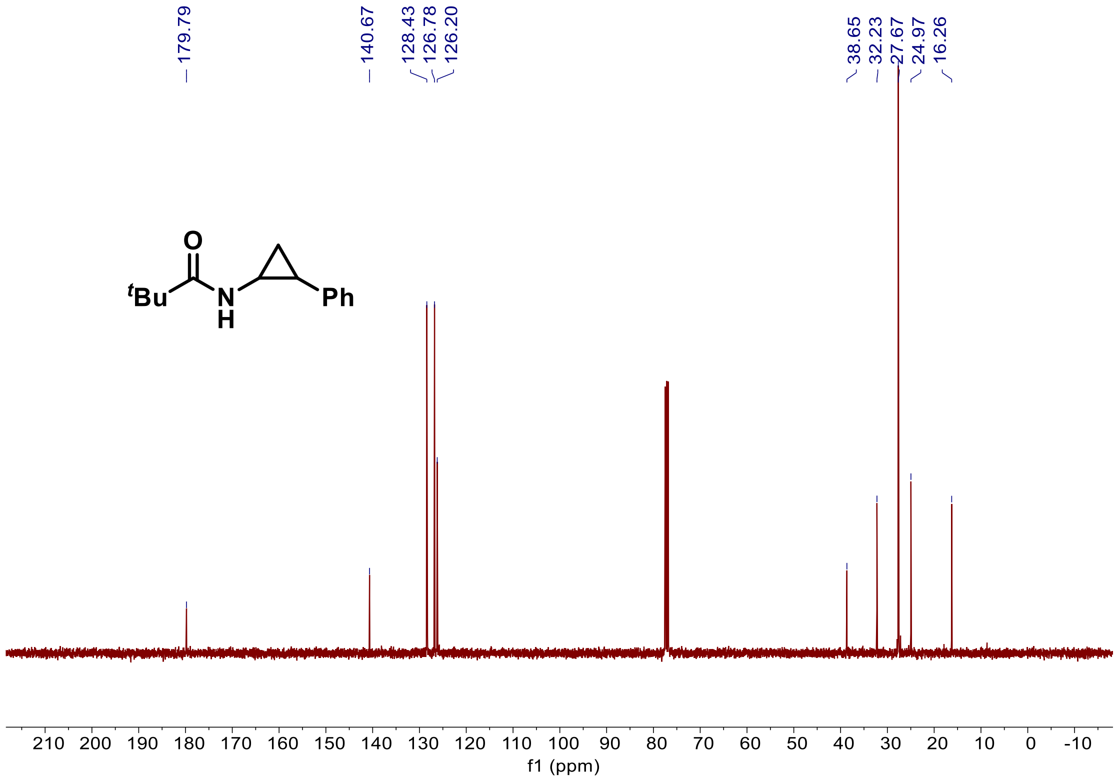

Supplementary Figure 53. $^{13}C\{^1H\}$ NMR spectrum of **1u** (101 MHz, Chloroform-*d*)

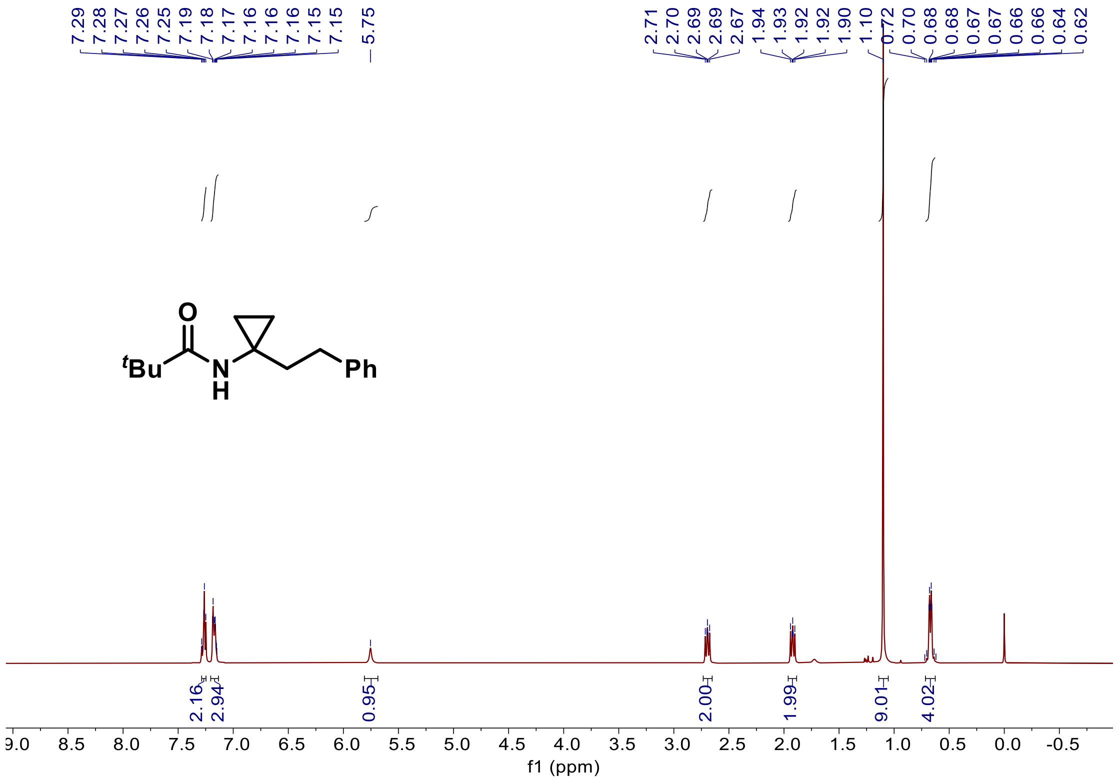

Supplementary Figure 54. ^{1}H NMR spectrum of **1v** (400 MHz, Chloroform-*d*)

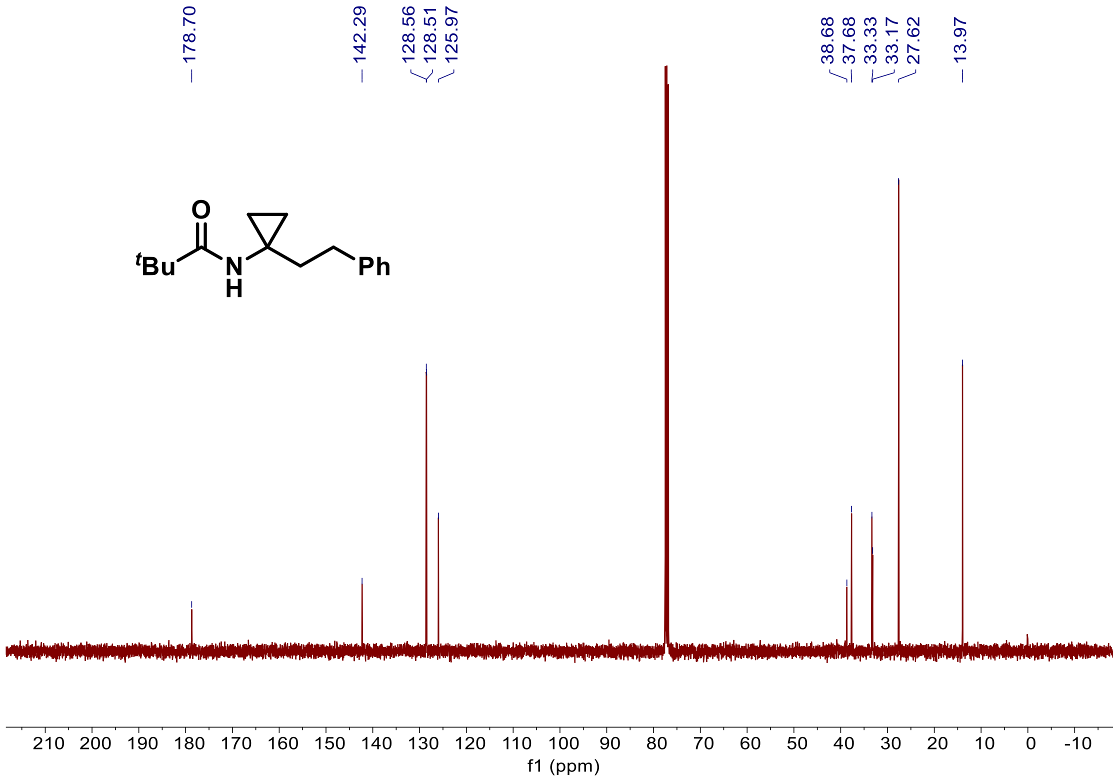

Supplementary Figure 55. $^{13}C\{^{1}H\}$ NMR spectrum of **1v** (101 MHz, Chloroform-*d*)

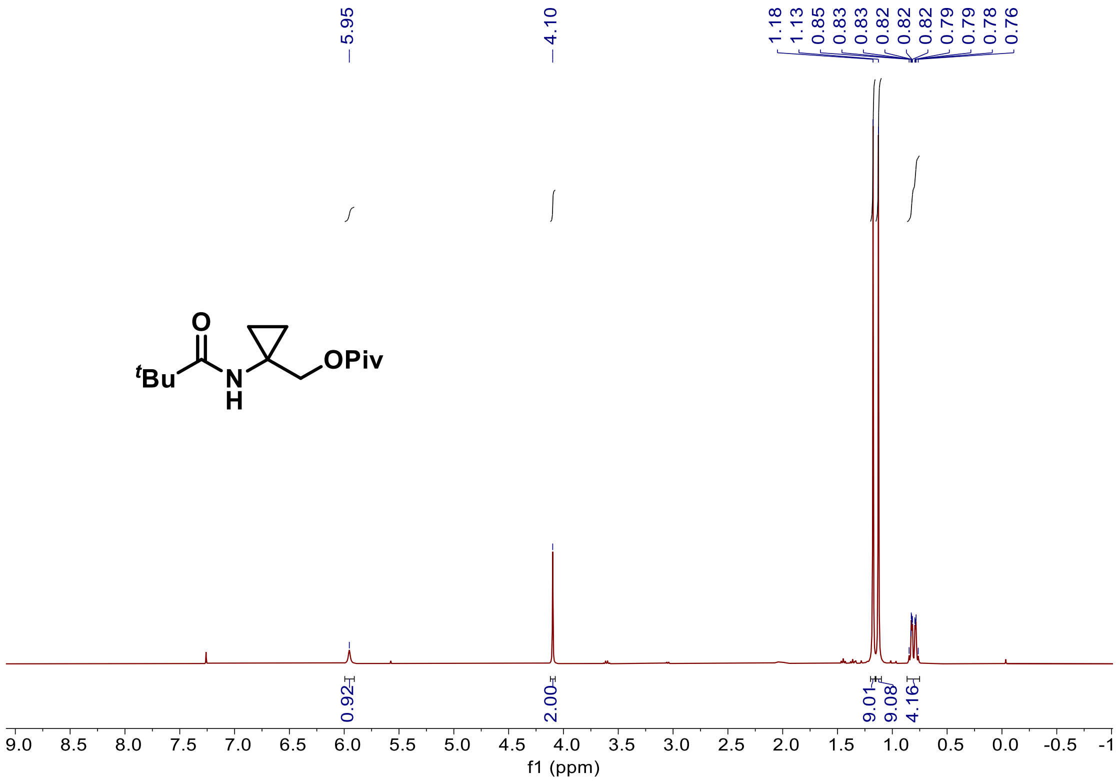

Supplementary Figure 56. ^{1}H NMR spectrum of **1w** (400 MHz, Chloroform-*d*)

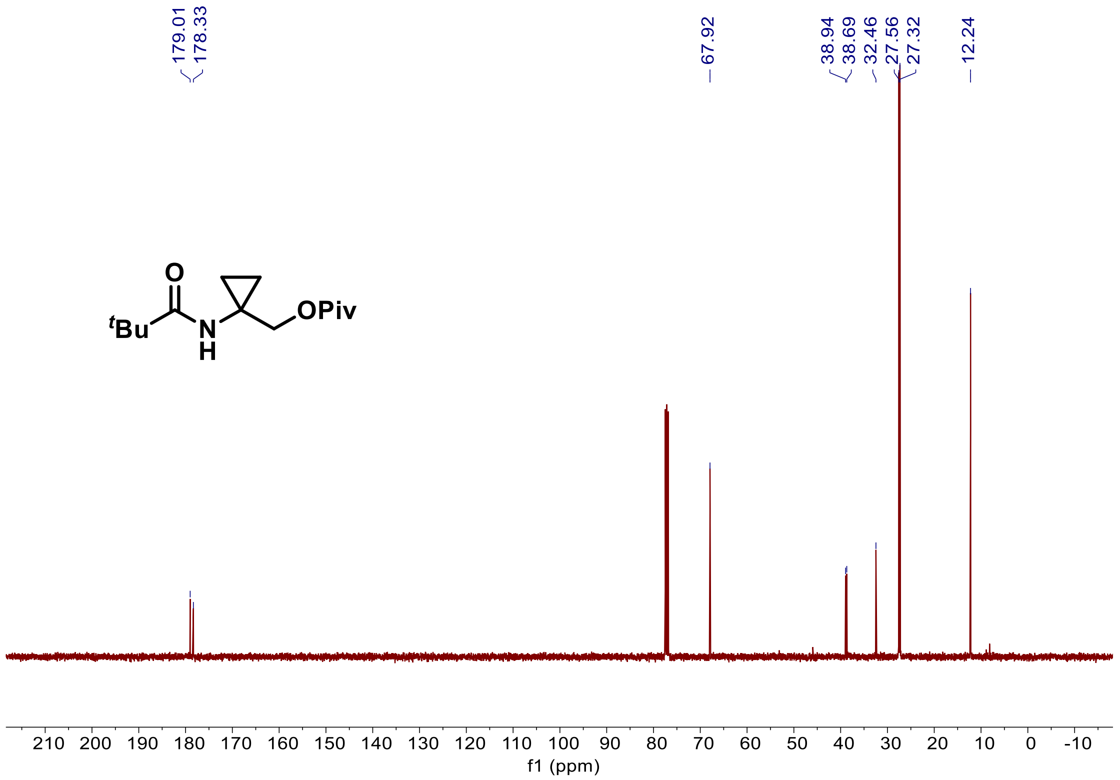

Supplementary Figure 57. $^{13}C\{^{1}H\}$ NMR spectrum of **1w** (101 MHz, Chloroform-*d*)

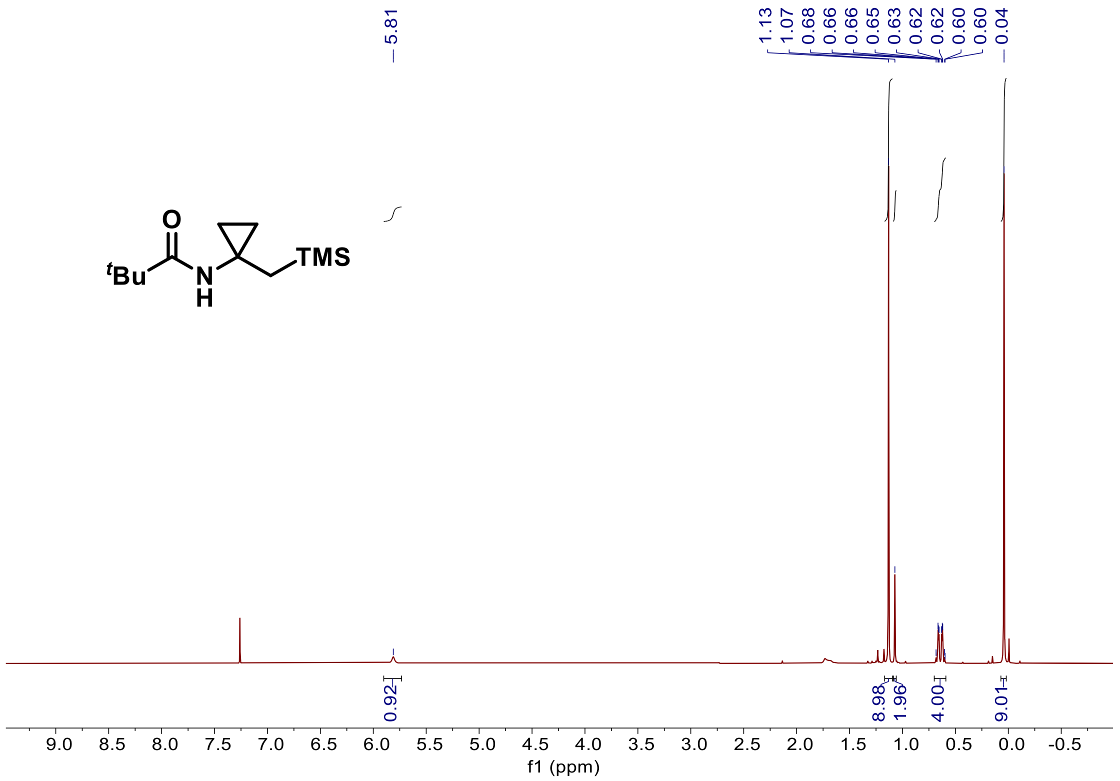

Supplementary Figure 58. ^{1}H NMR spectrum of **1x** (400 MHz, Chloroform-*d*)

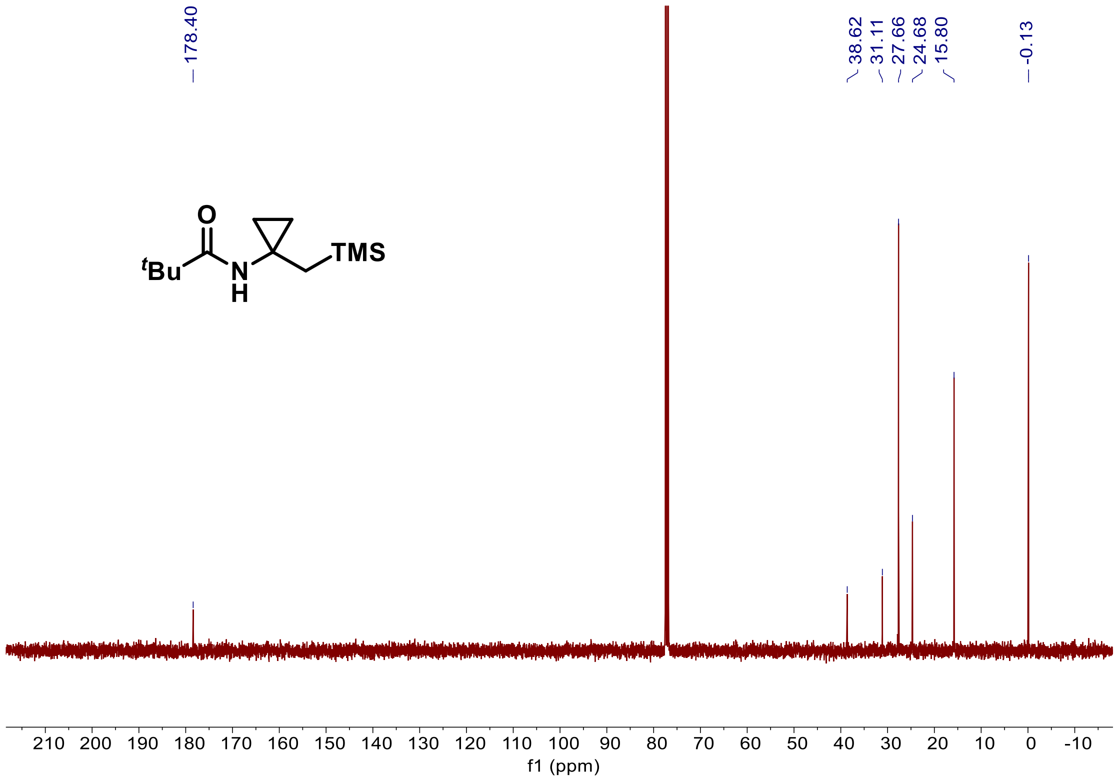

Supplementary Figure 59. $^{13}C\{^{1}H\}$ NMR spectrum of **1x** (101 MHz, Chloroform-*d*)

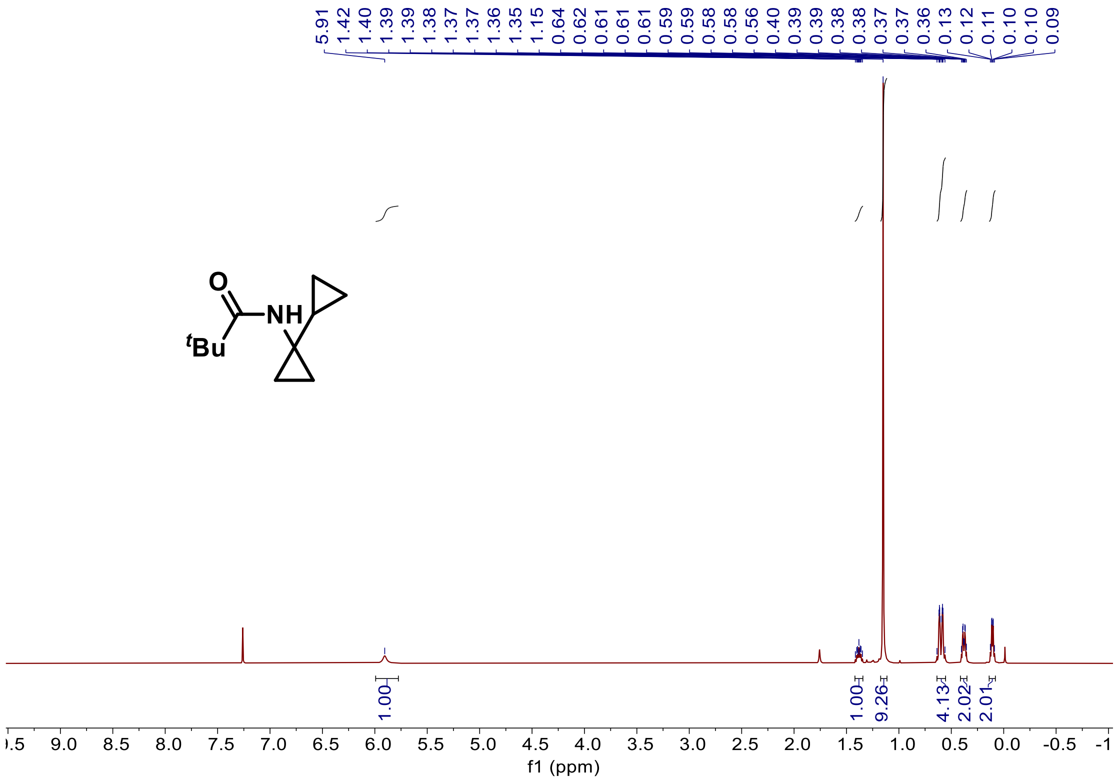

Supplementary Figure 60. ^{1}H NMR spectrum of **1y** (400 MHz, Chloroform-*d*)

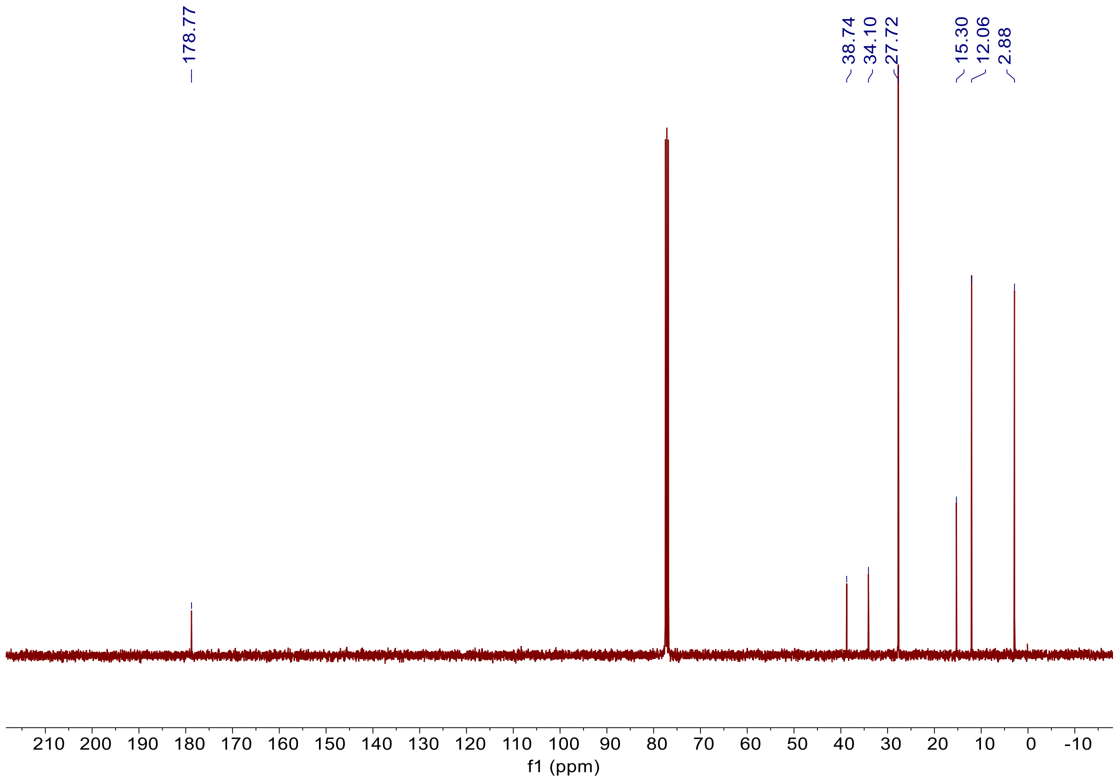

Supplementary Figure 61. $^{13}C\{^{1}H\}$ NMR spectrum of **1y** (101 MHz, Chloroform-*d*)

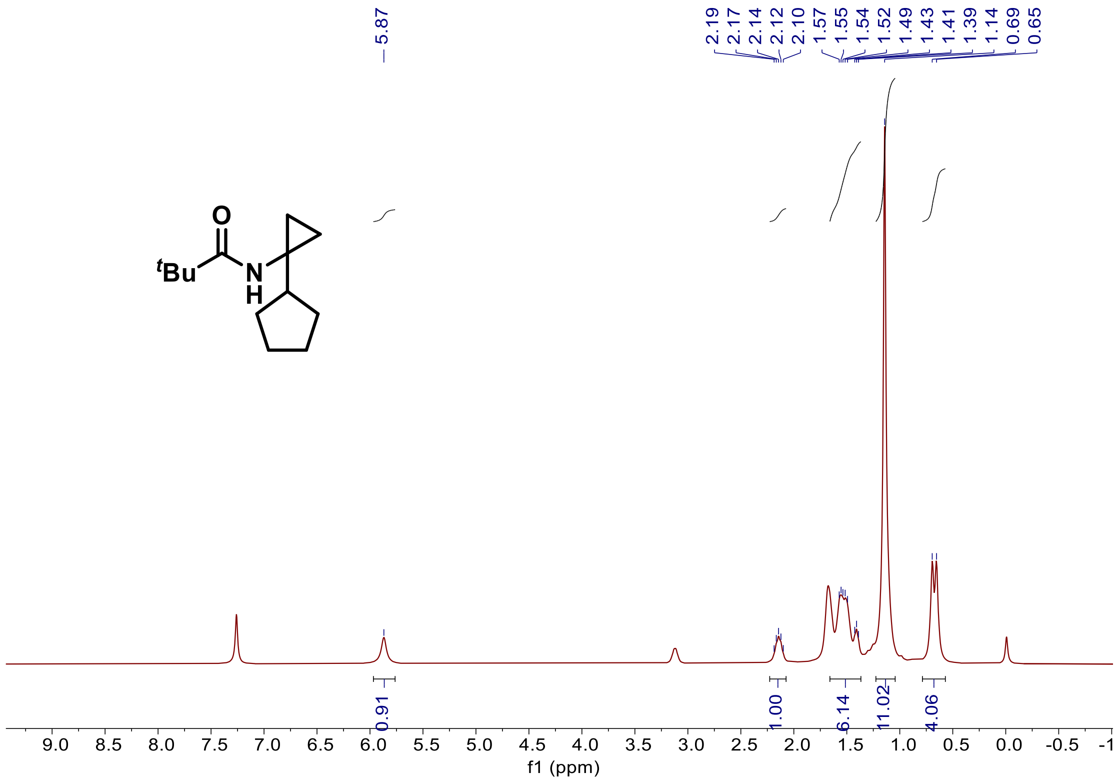

Supplementary Figure 62. ^{1}H NMR spectrum of **1z** (400 MHz, Chloroform-*d*)

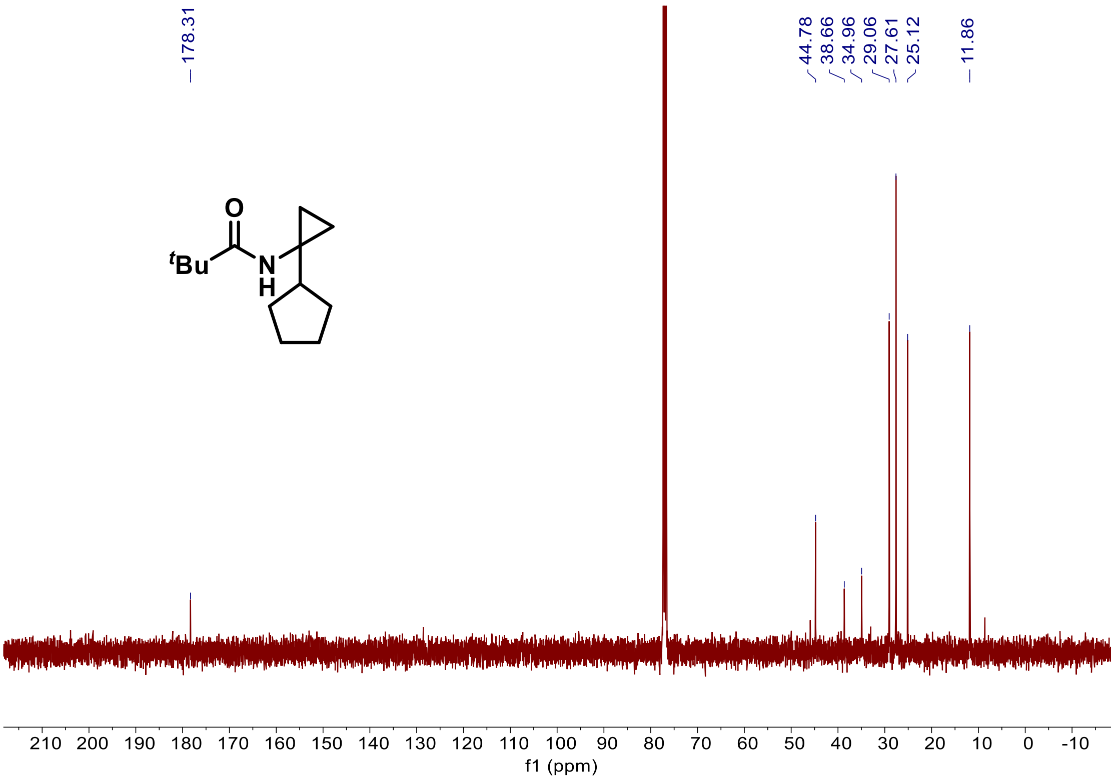

Supplementary Figure 63. $^{13}C\{^{1}H\}$ NMR spectrum of **1z** (101 MHz, Chloroform-*d*)

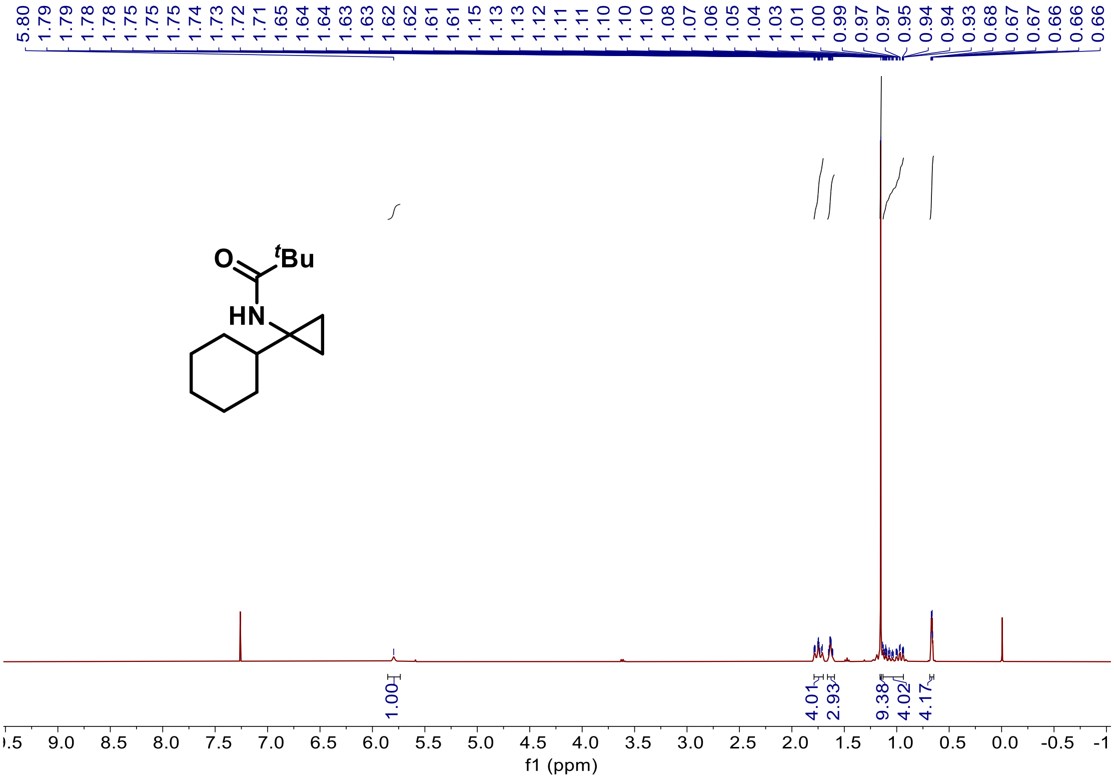

Supplementary Figure 64. ^{1}H NMR spectrum of **1aa** (400 MHz, Chloroform-*d*)

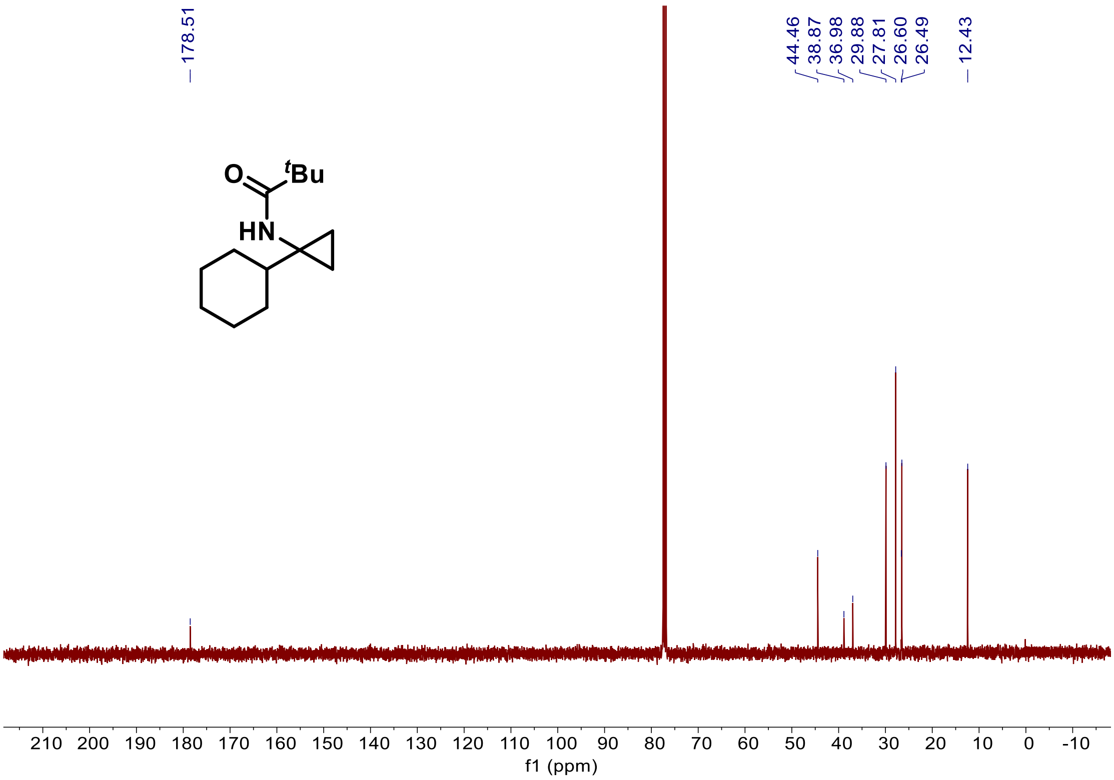

Supplementary Figure 65. $^{13}C\{^1H\}$ NMR spectrum of **1aa** (101 MHz, Chloroform-*d*)

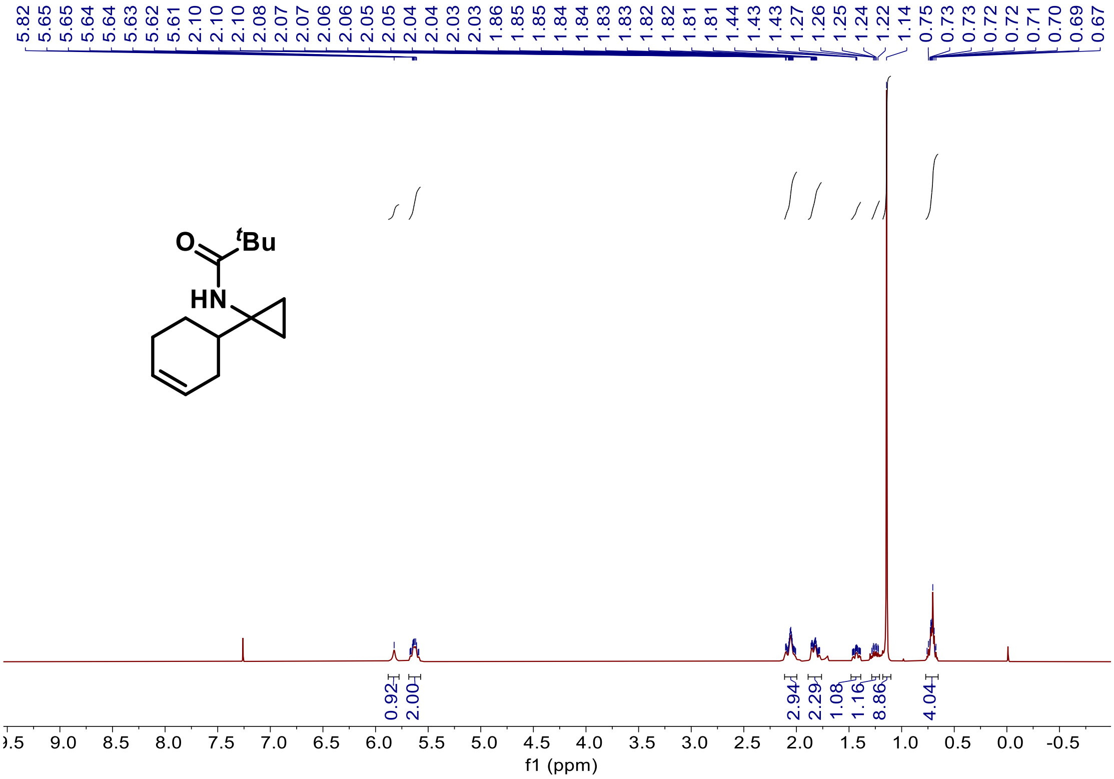

Supplementary Figure 66. ^1H NMR spectrum of **1ab** (400 MHz, Chloroform-*d*)

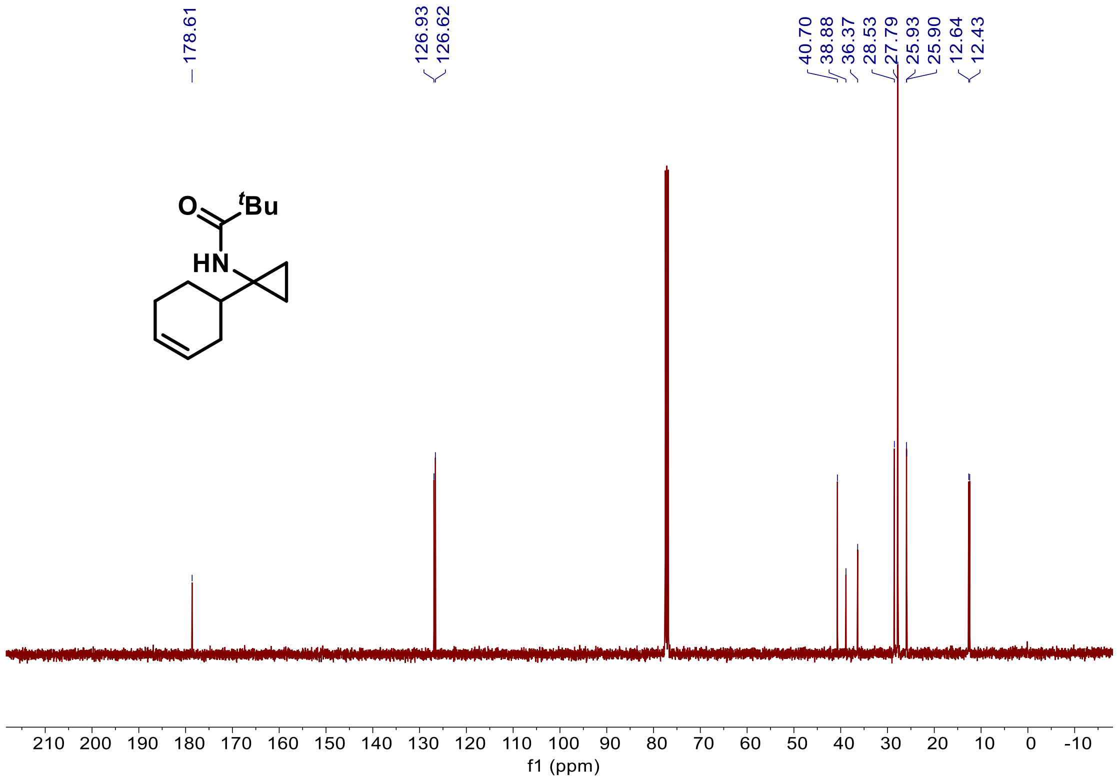

Supplementary Figure 67. $^{13}C\{^{1}H\}$ NMR spectrum of **1ab** (101 MHz, Chloroform-*d*)

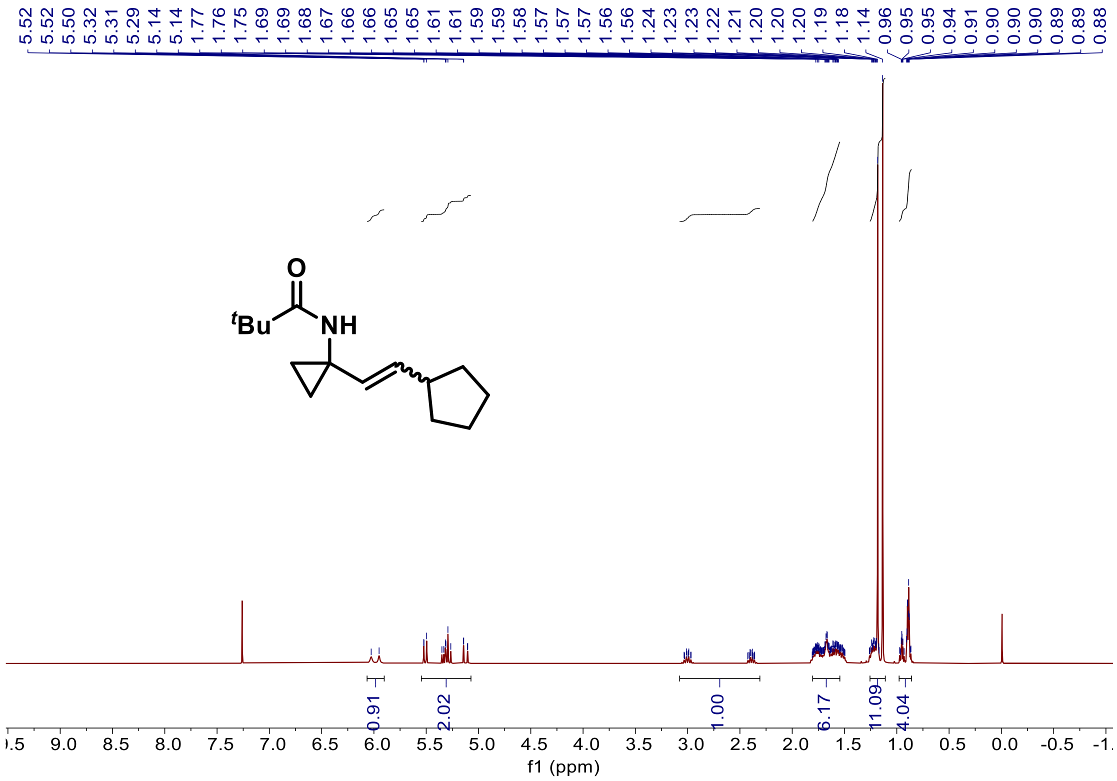

Supplementary Figure 68. ^{1}H NMR spectrum of **1ac** (400 MHz, Chloroform-*d*)

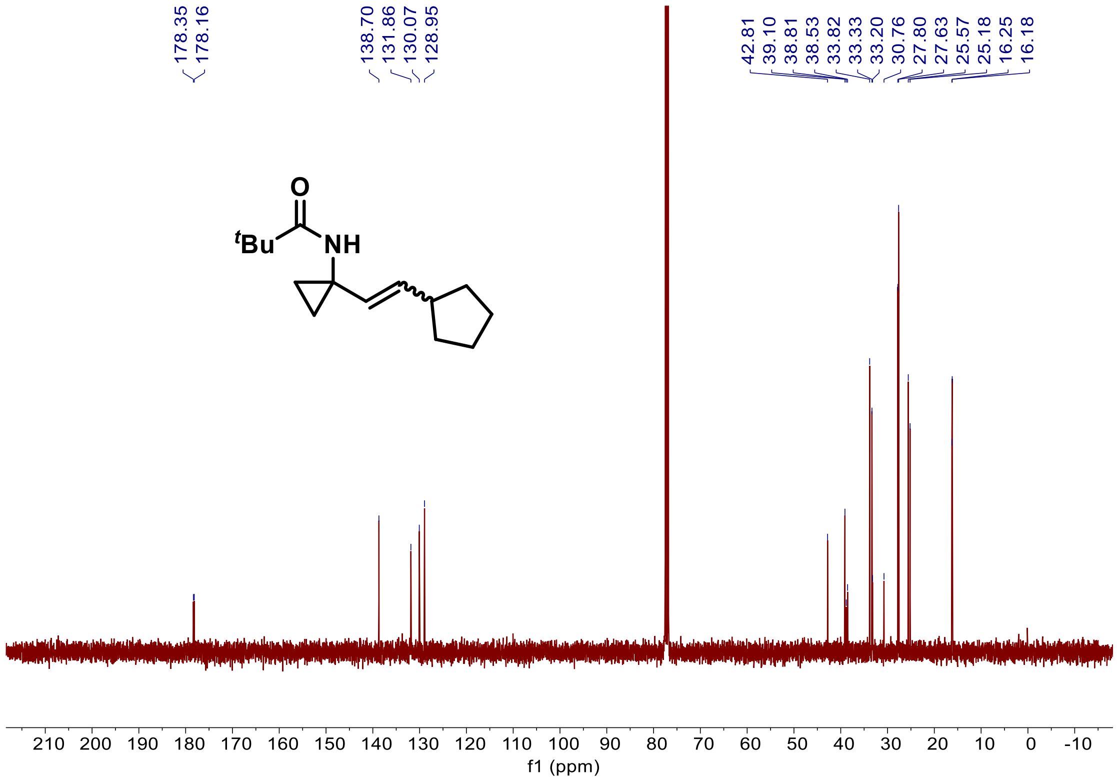

Supplementary Figure 69. $^{13}C\{^1H\}$ NMR spectrum of **1ac** (101 MHz, Chloroform-*d*)

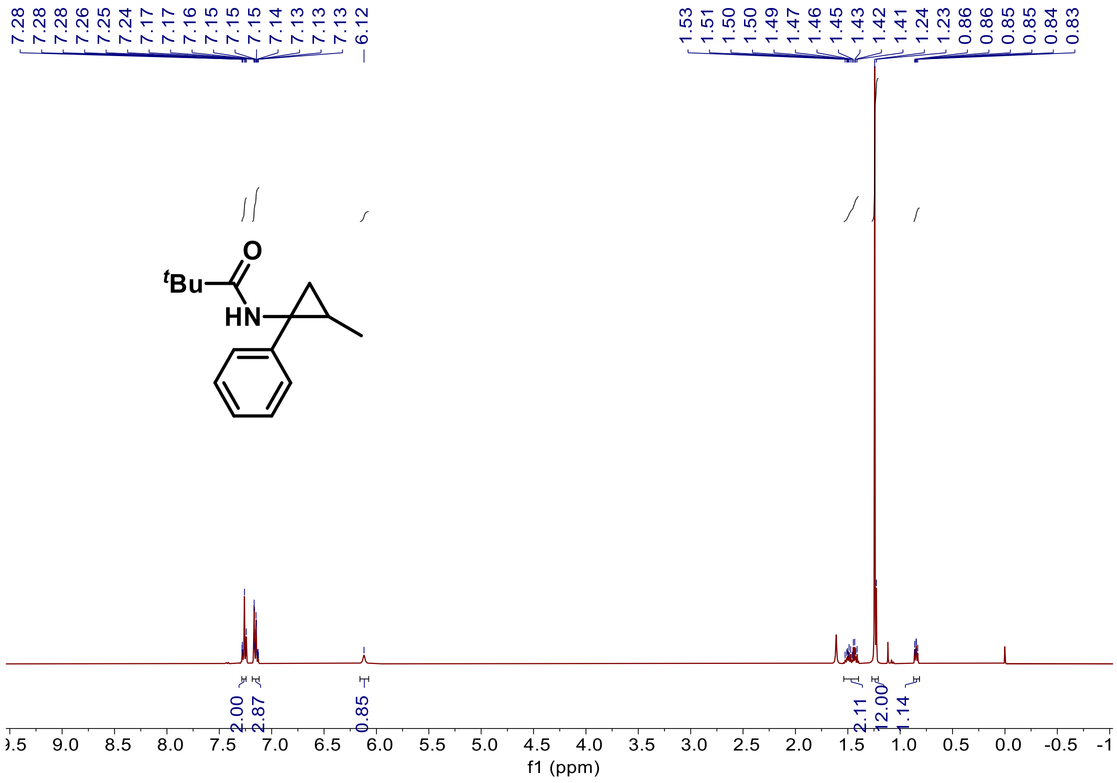

Supplementary Figure 70. ^1H NMR spectrum of **1ad** (400 MHz, Chloroform-*d*)

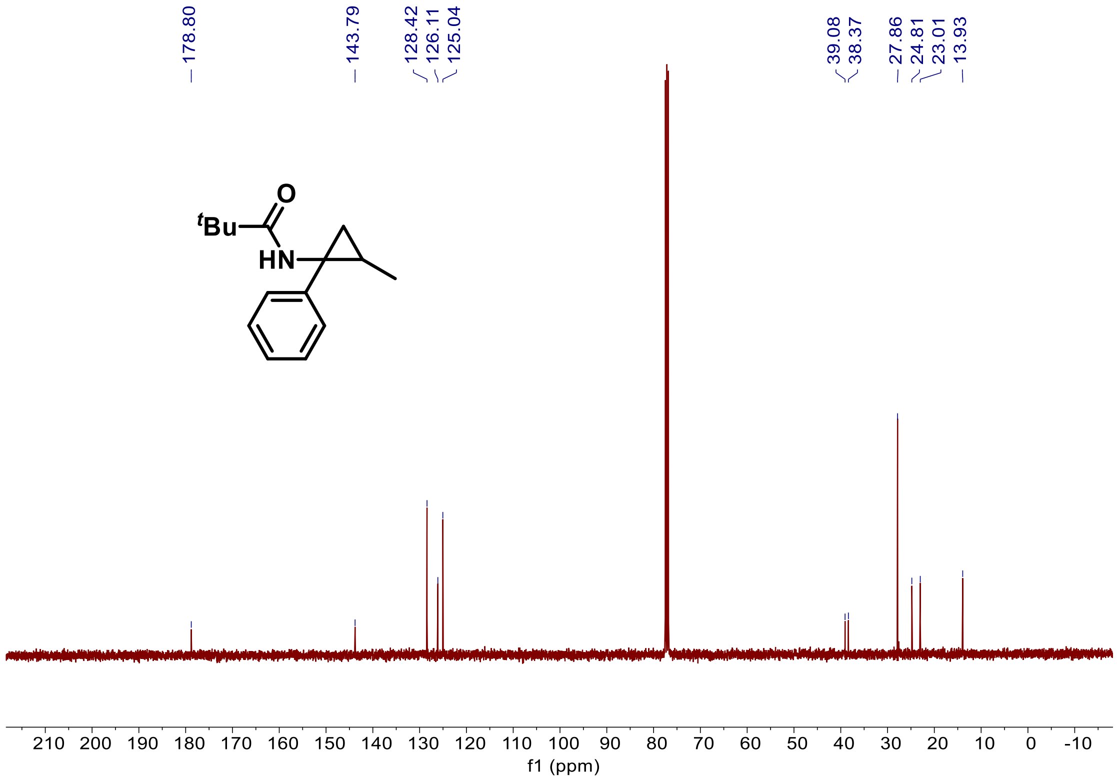

Supplementary Figure 71. $^{13}C\{^1H\}$ NMR spectrum of **1ad** (101 MHz, Chloroform-*d*)

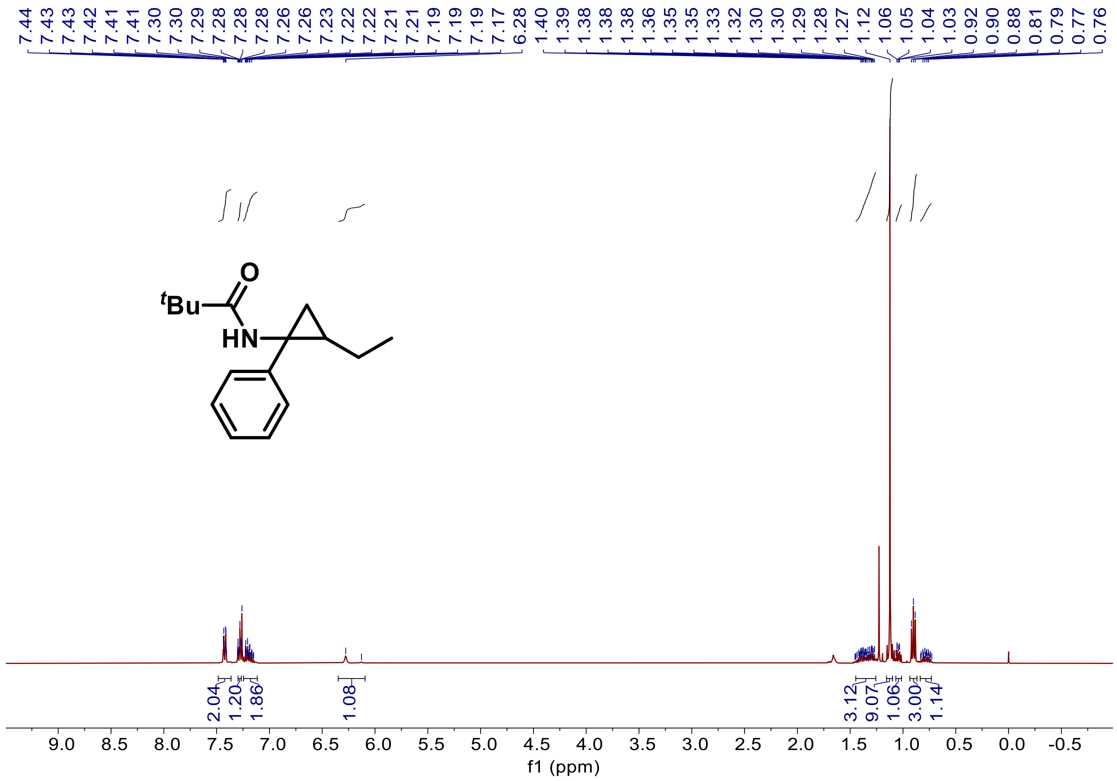

Supplementary Figure 72. ^{1}H NMR spectrum of **1ae** (400 MHz, Chloroform-*d*)

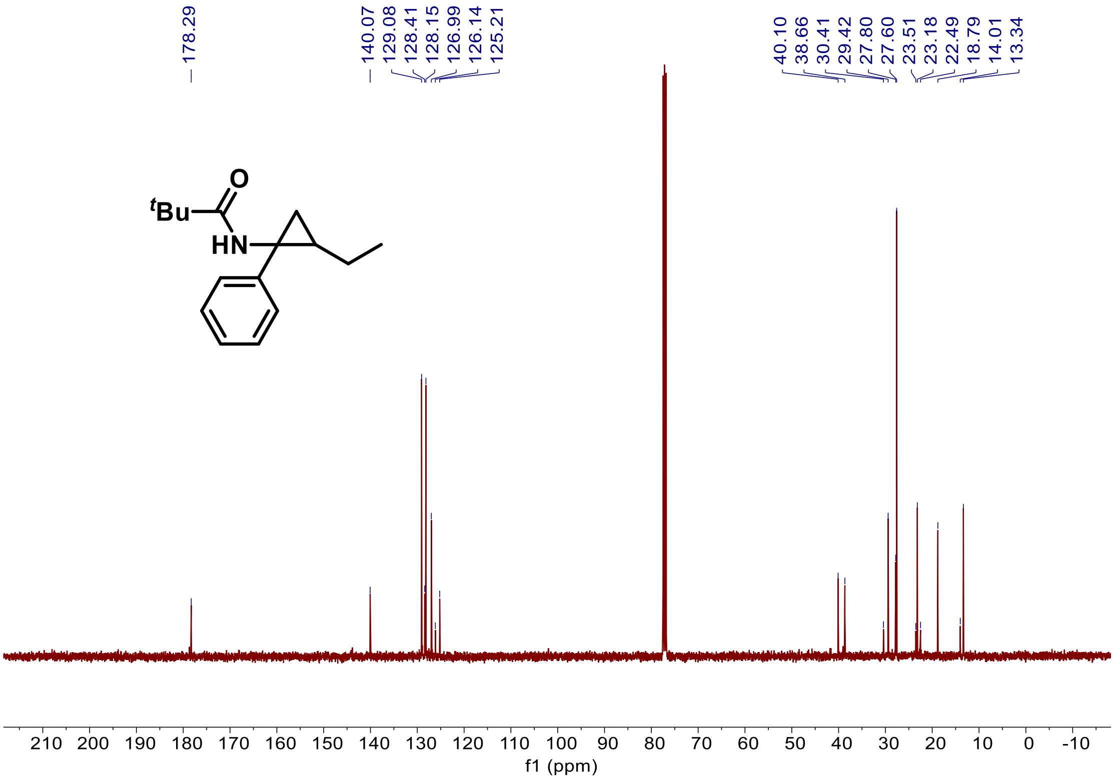

Supplementary Figure 73. $^{13}C\{^{1}H\}$ NMR spectrum of **1ae** (101 MHz, Chloroform-*d*)

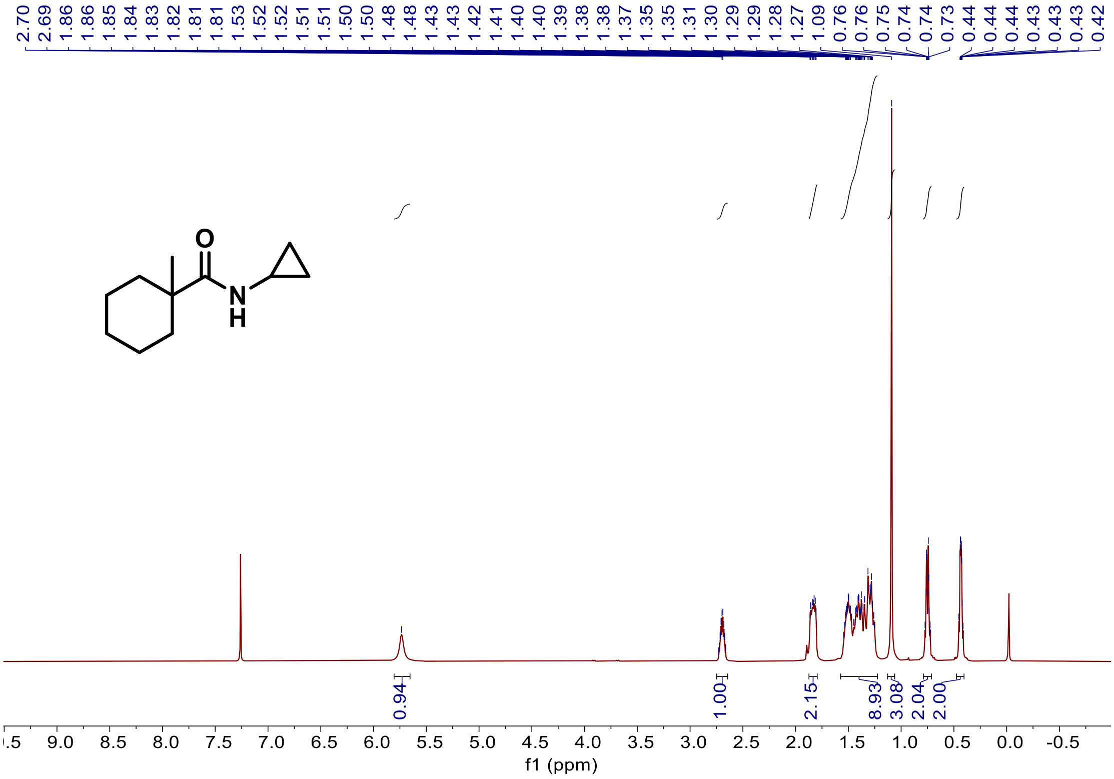

Supplementary Figure 74. ^{1}H NMR spectrum of **1af** (400 MHz, Chloroform-*d*)

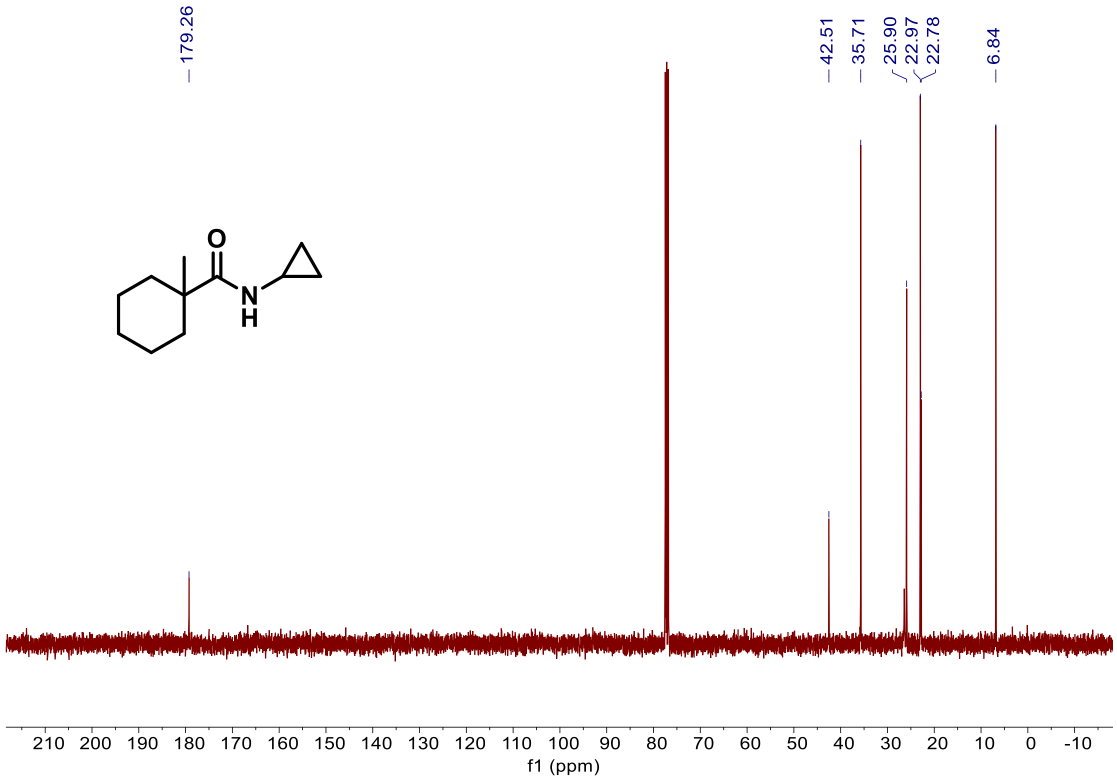

Supplementary Figure 75. $^{13}C\{^{1}H\}$ NMR spectrum of **1af** (101 MHz, Chloroform-*d*)

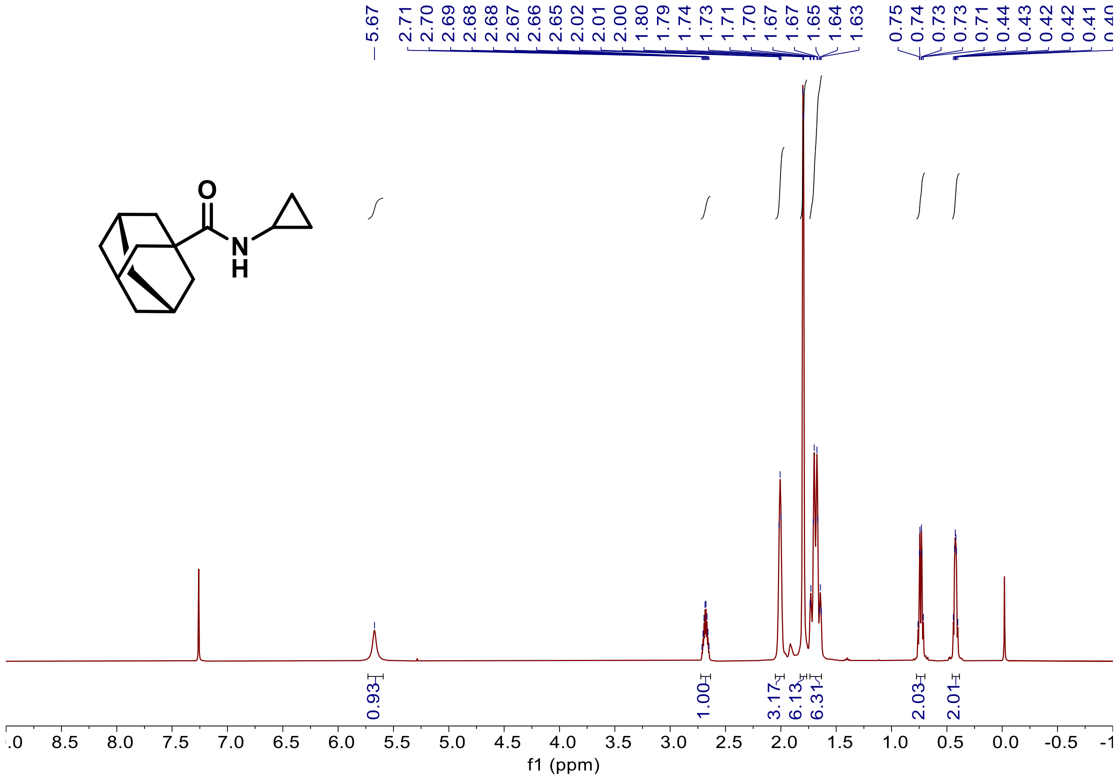

Supplementary Figure 76. ^{1}H NMR spectrum of **1ag** (400 MHz, Chloroform-*d*)

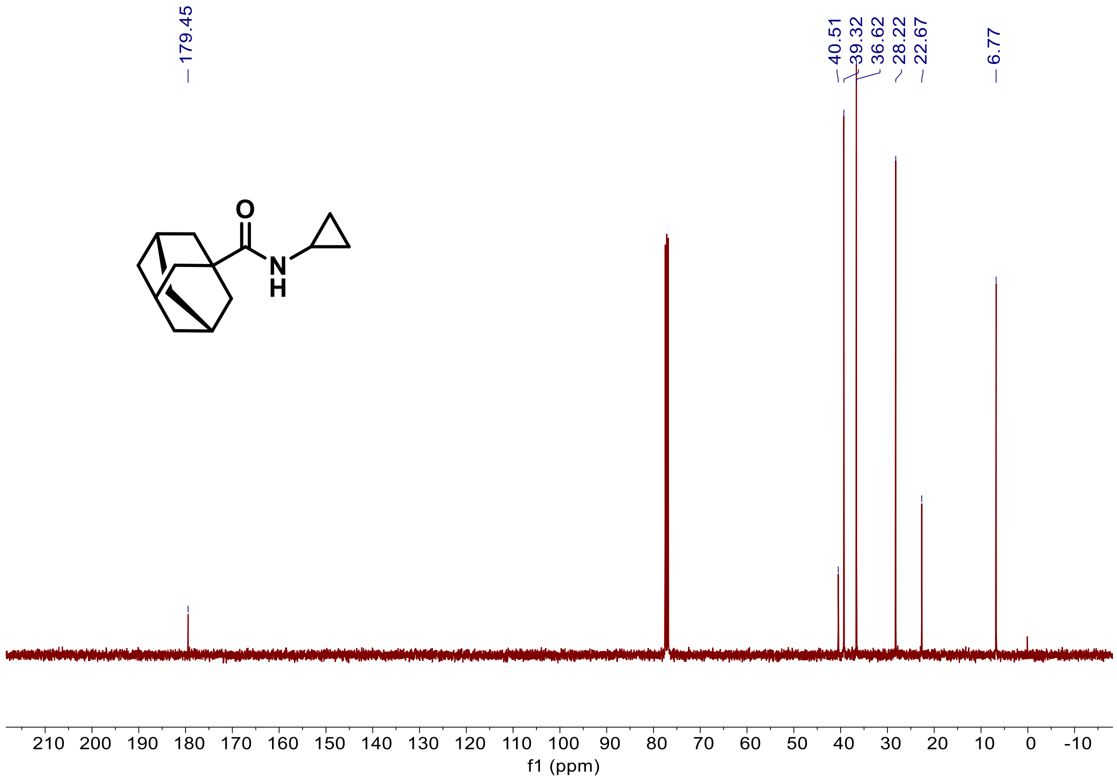

Supplementary Figure 77. $^{13}C\{^{1}H\}$ NMR spectrum of **1ag** (101 MHz, Chloroform-*d*)

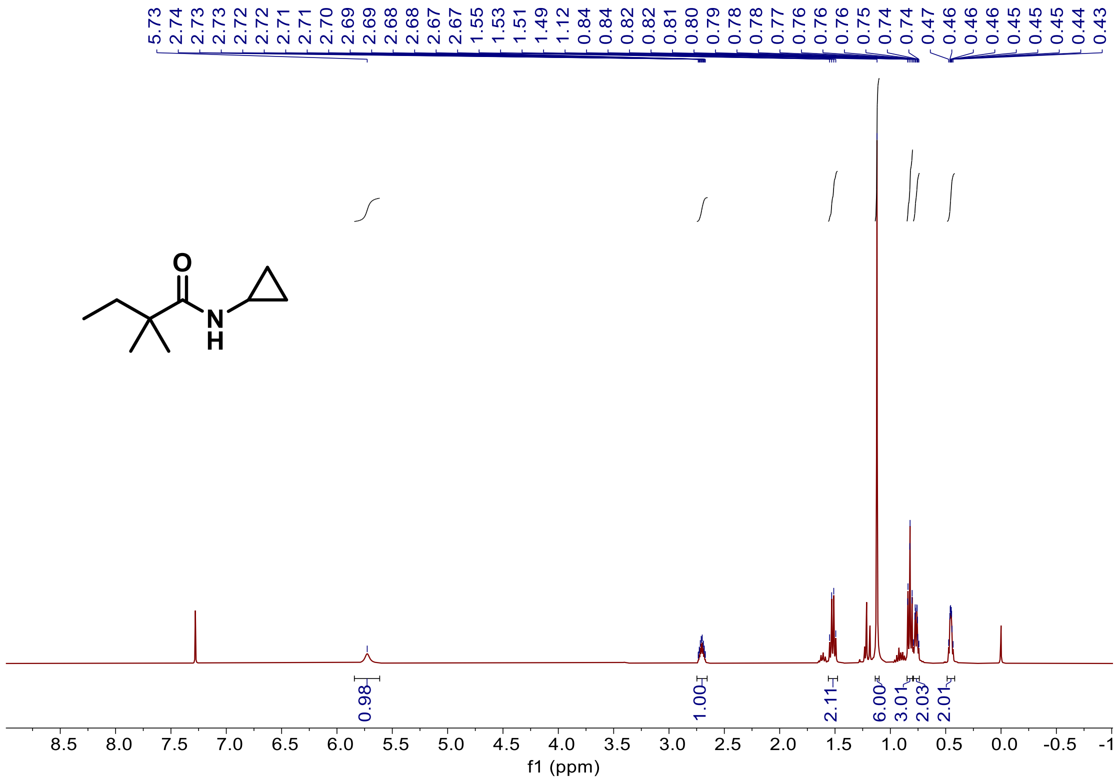

Supplementary Figure 78. ^{1}H NMR spectrum of **1ah** (400 MHz, Chloroform-*d*)

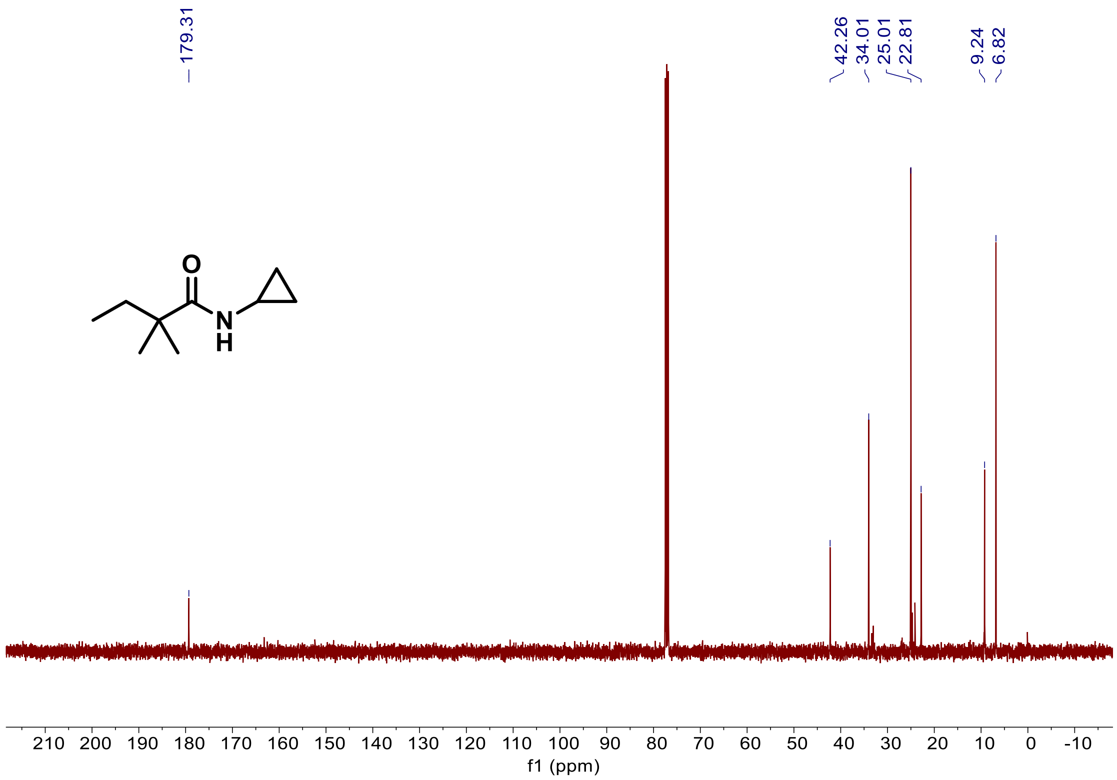

Supplementary Figure 79. $^{13}C\{^{1}H\}$ NMR spectrum of **1ah** (101 MHz, Chloroform-*d*)

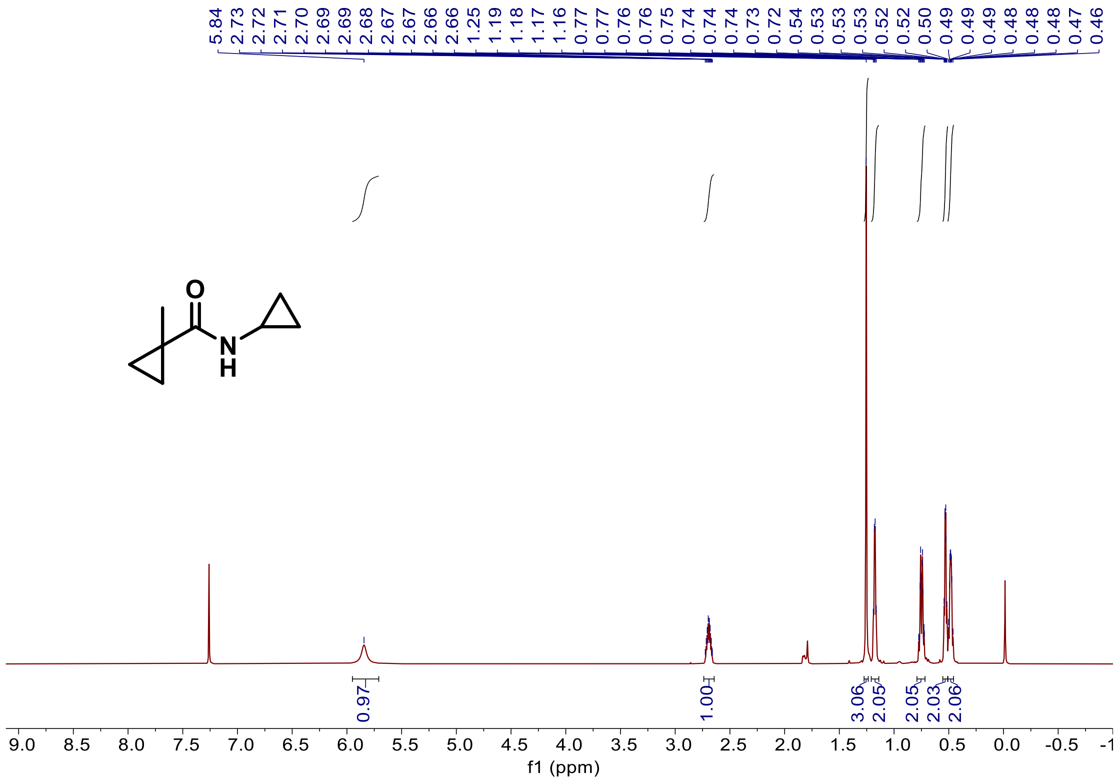

Supplementary Figure 80. ^{1}H NMR spectrum of **1ai** (400 MHz, Chloroform-*d*)

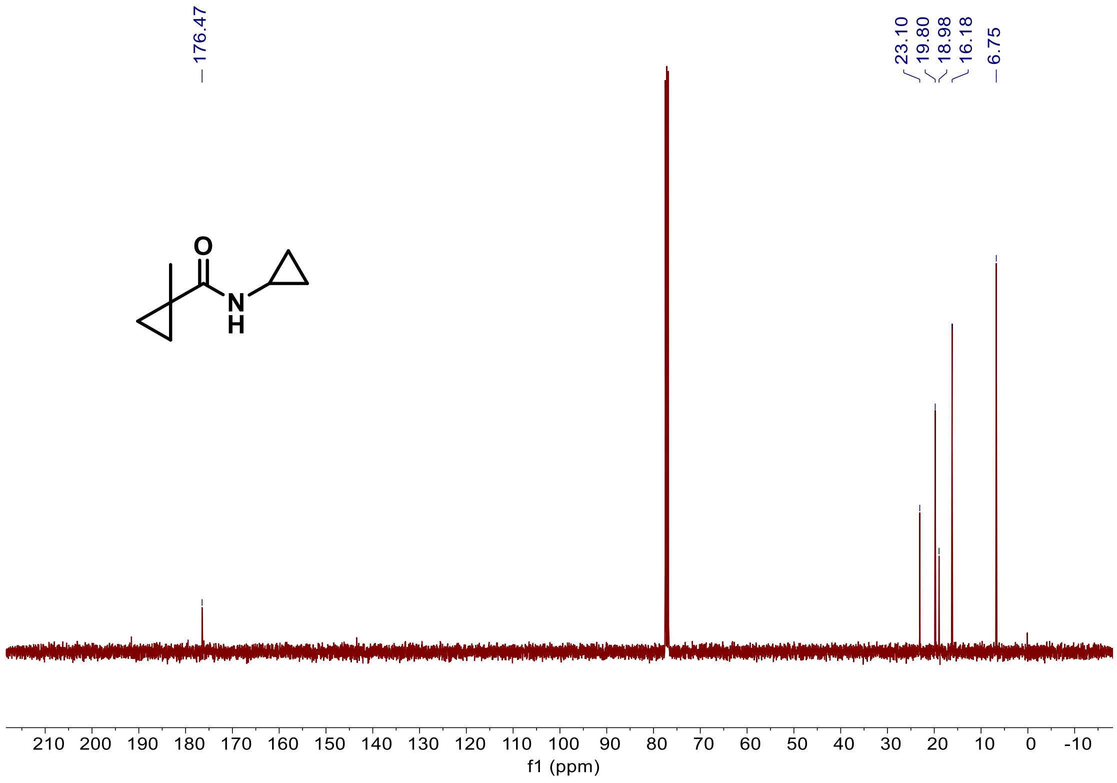

Supplementary Figure 81. $^{13}C\{^{1}H\}$ NMR spectrum of **1ai** (101 MHz, Chloroform-*d*)

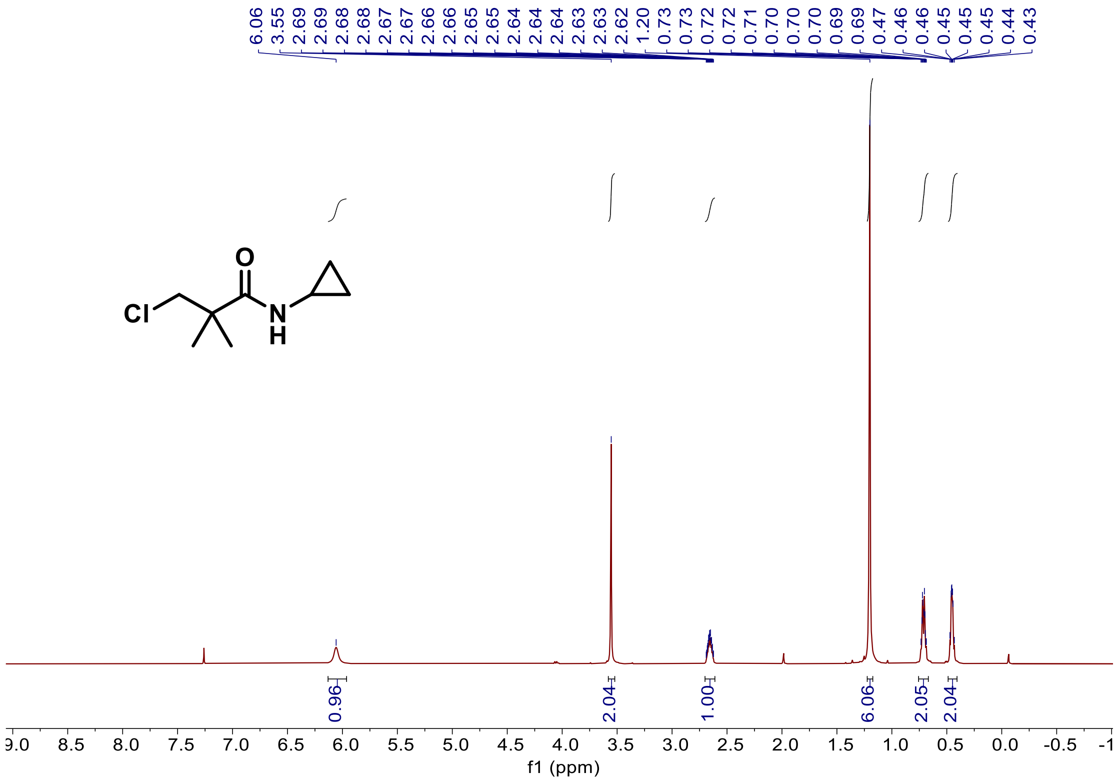

Supplementary Figure 82. ^{1}H NMR spectrum of **1aj** (400 MHz, Chloroform-*d*)

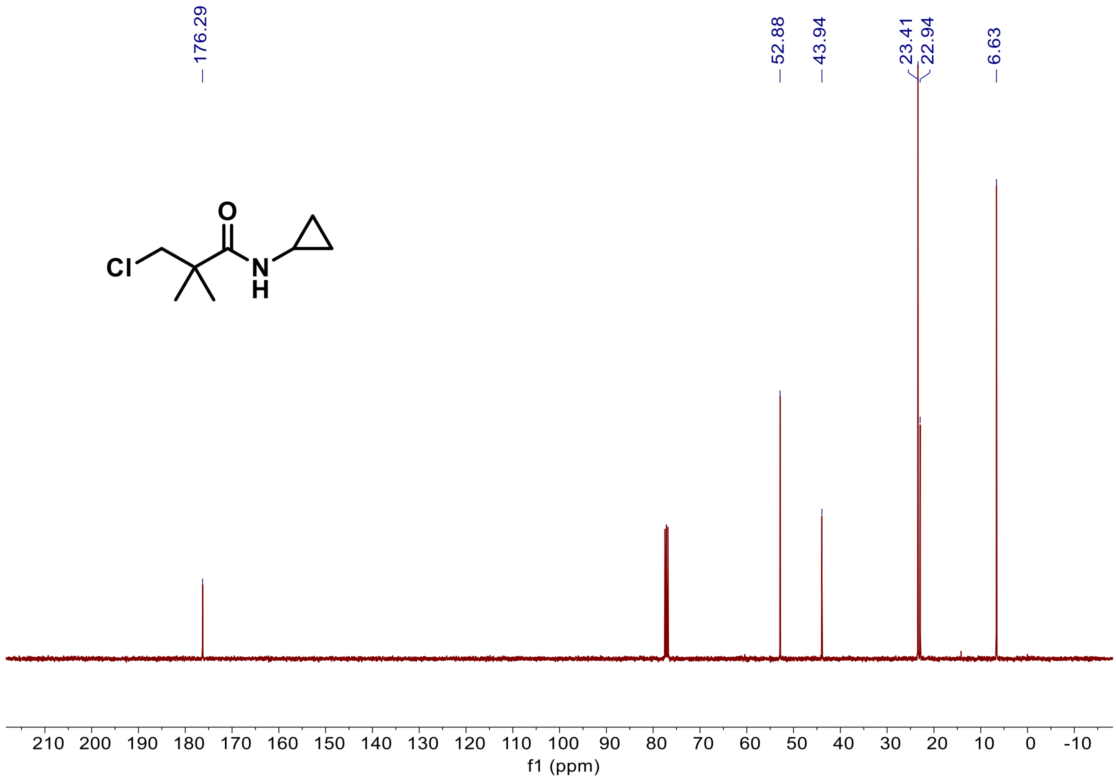

Supplementary Figure 83. $^{13}C\{^1H\}$ NMR spectrum of **1aj** (101 MHz, Chloroform-*d*)

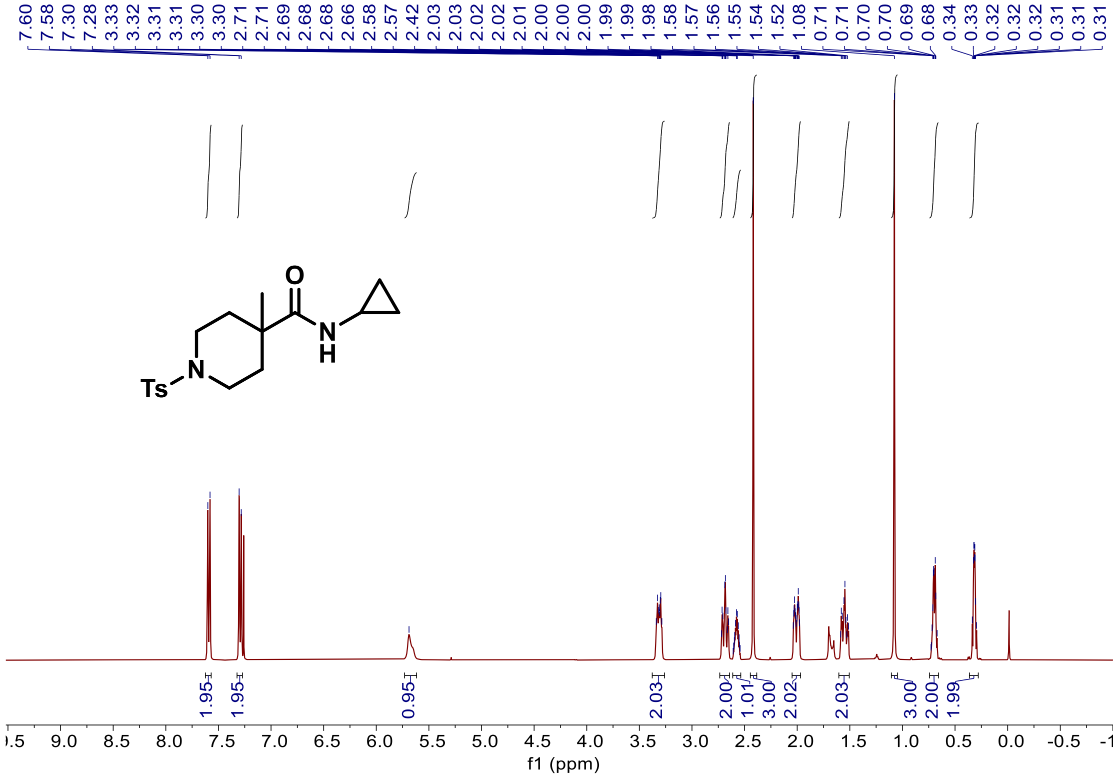

Supplementary Figure 84. ^1H NMR spectrum of **1ak** (400 MHz, Chloroform-*d*)

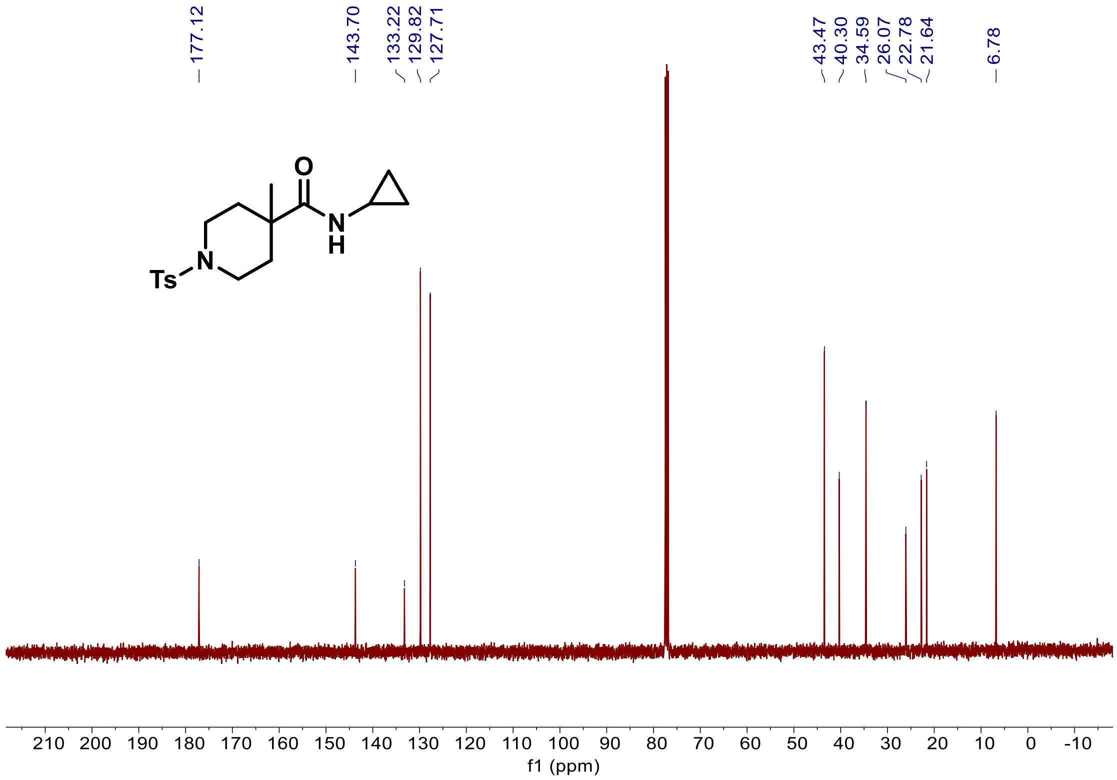

Supplementary Figure 85. $^{13}C\{^1H\}$ NMR spectrum of **1ak** (101 MHz, Chloroform-*d*)

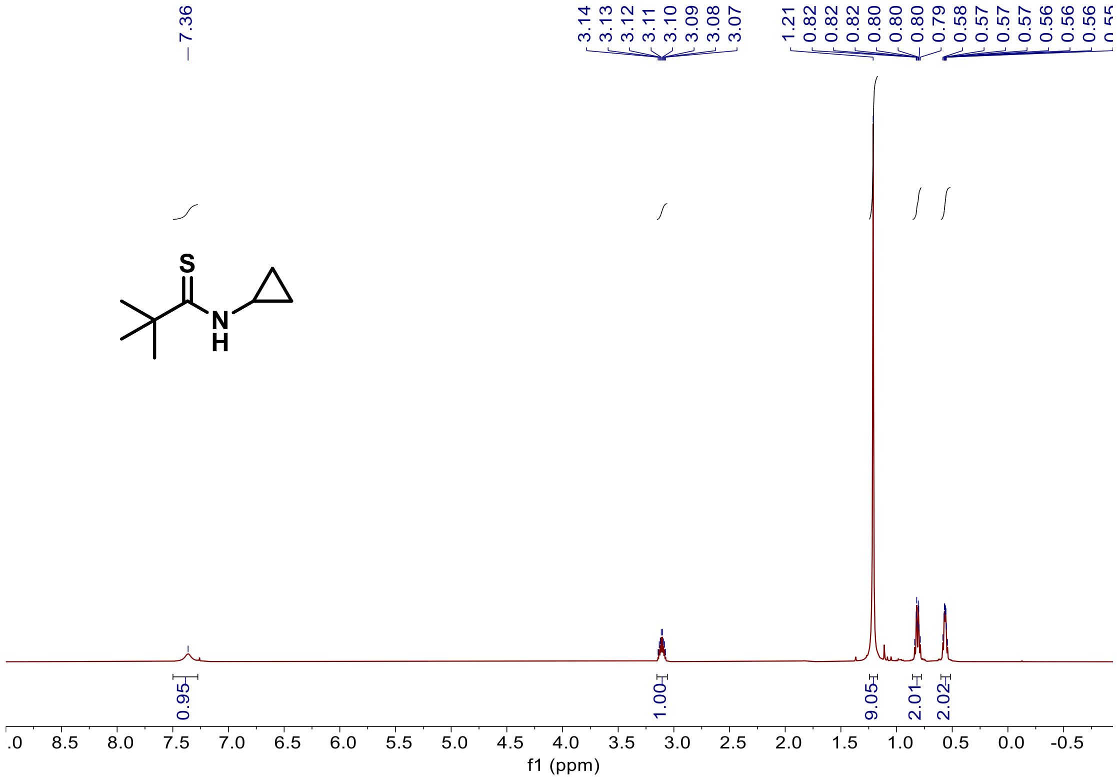

Supplementary Figure 86. ^1H NMR spectrum of **1al** (400 MHz, Chloroform-*d*)

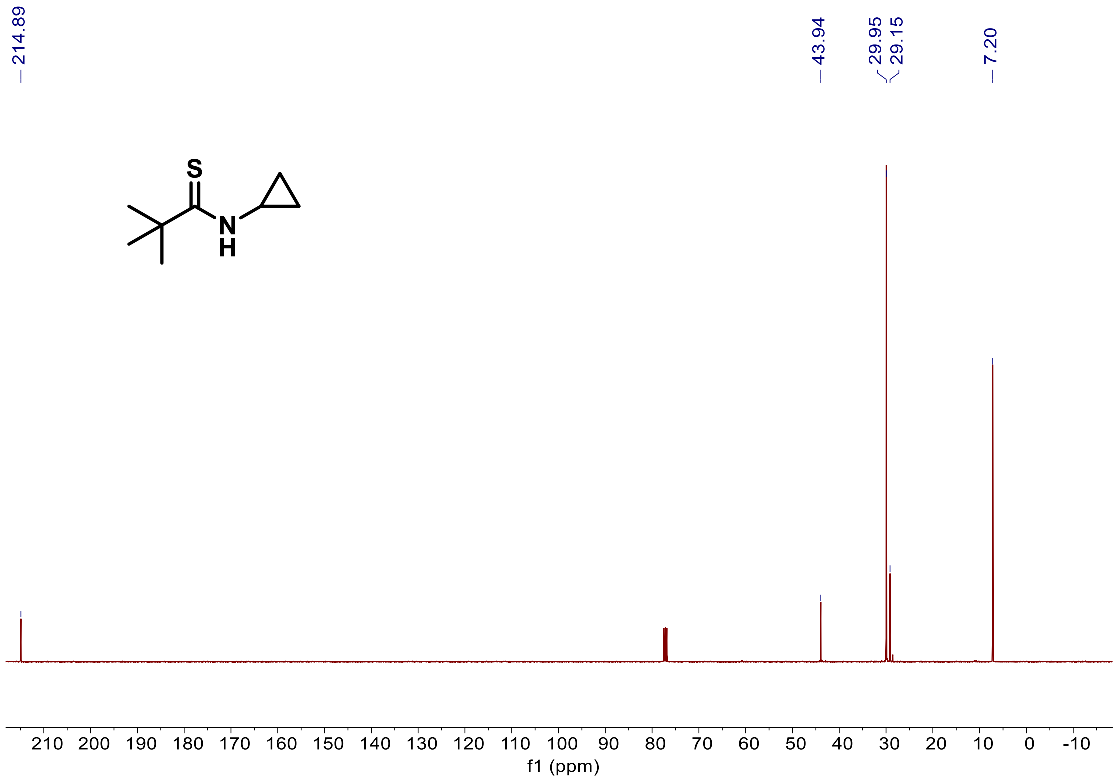

Supplementary Figure 87. $^{13}C\{^1H\}$ NMR spectrum of **1al** (101 MHz, Chloroform-*d*)

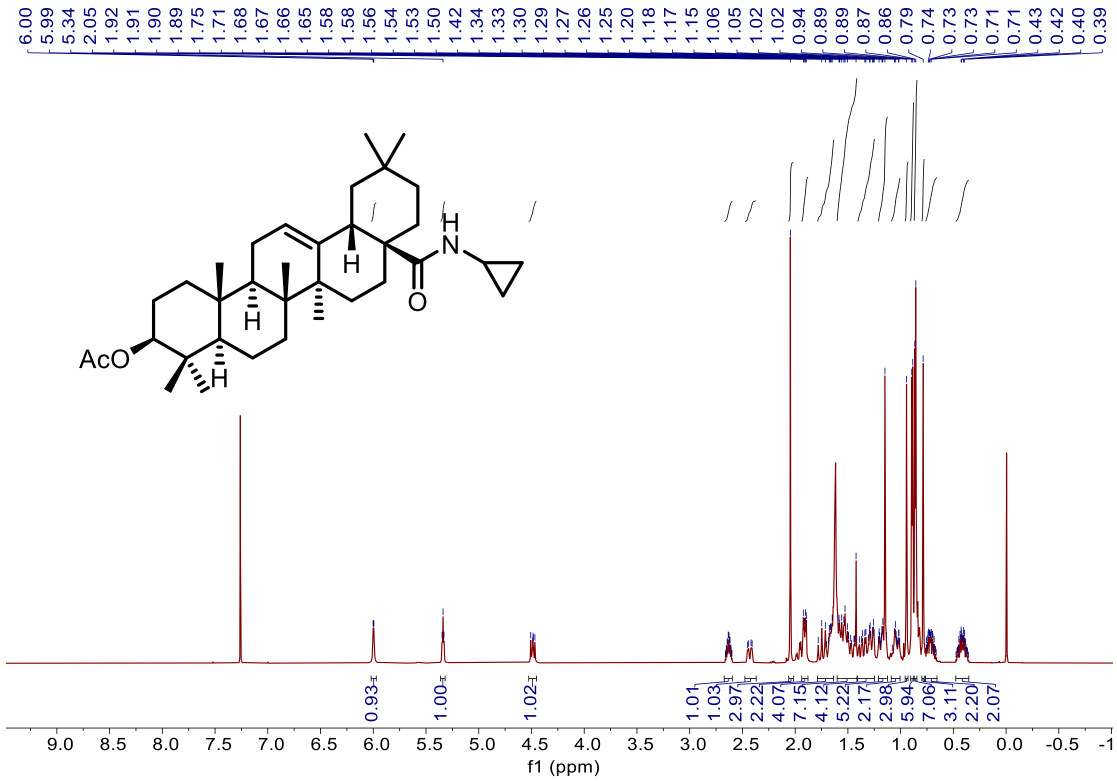

Supplementary Figure 88. ^{1}H NMR spectrum of **1am** (400 MHz, Chloroform-*d*)

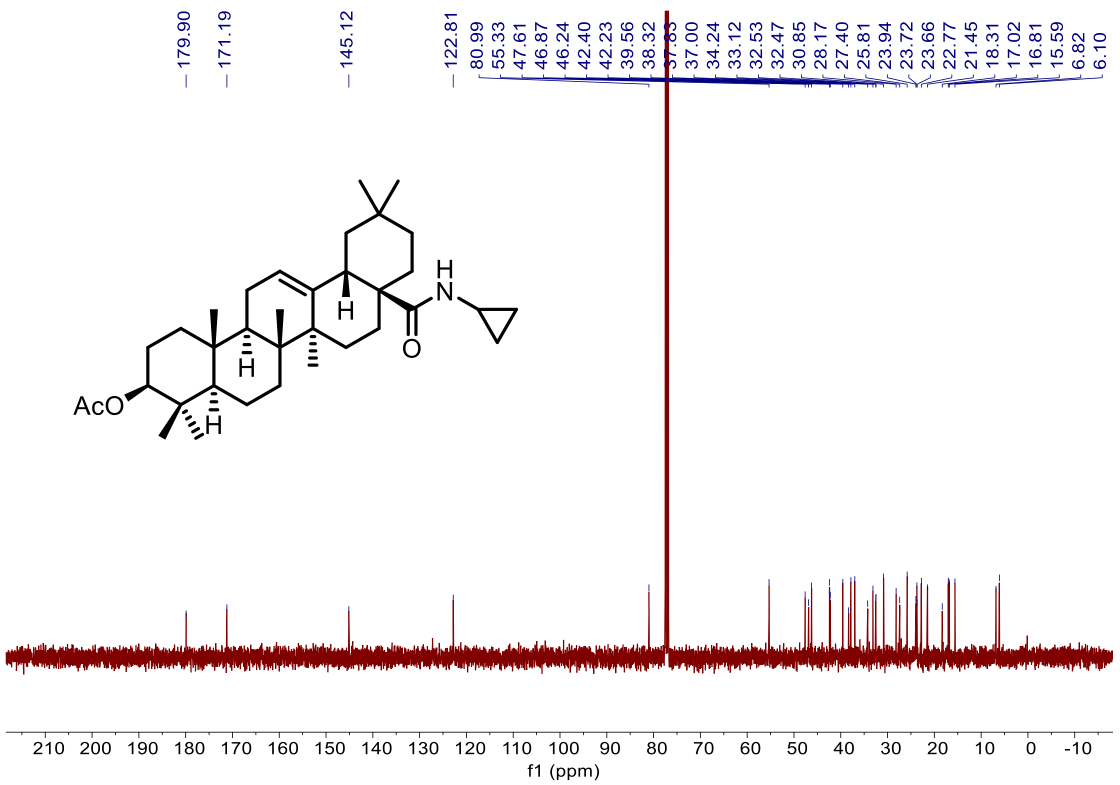

Supplementary Figure 89. $^{13}C\{^1H\}$ NMR spectrum of **1am** (101 MHz, Chloroform-*d*)

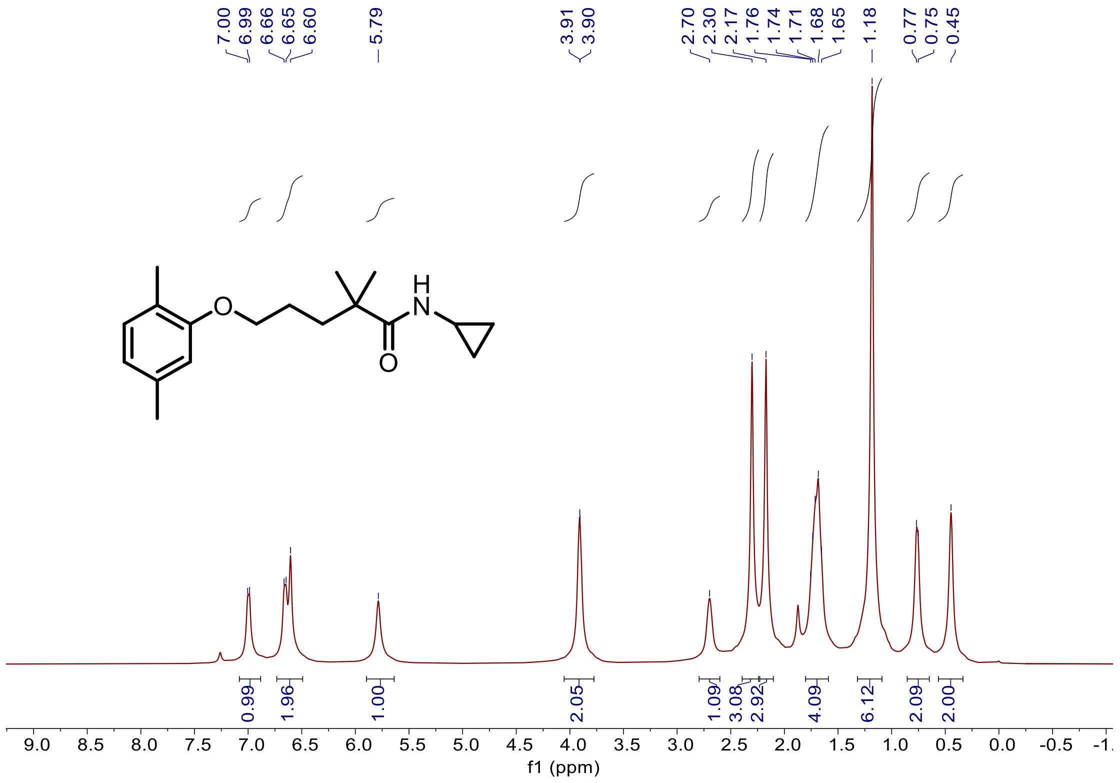

Supplementary Figure 90. ^1H NMR spectrum of **1an** (400 MHz, Chloroform-*d*)

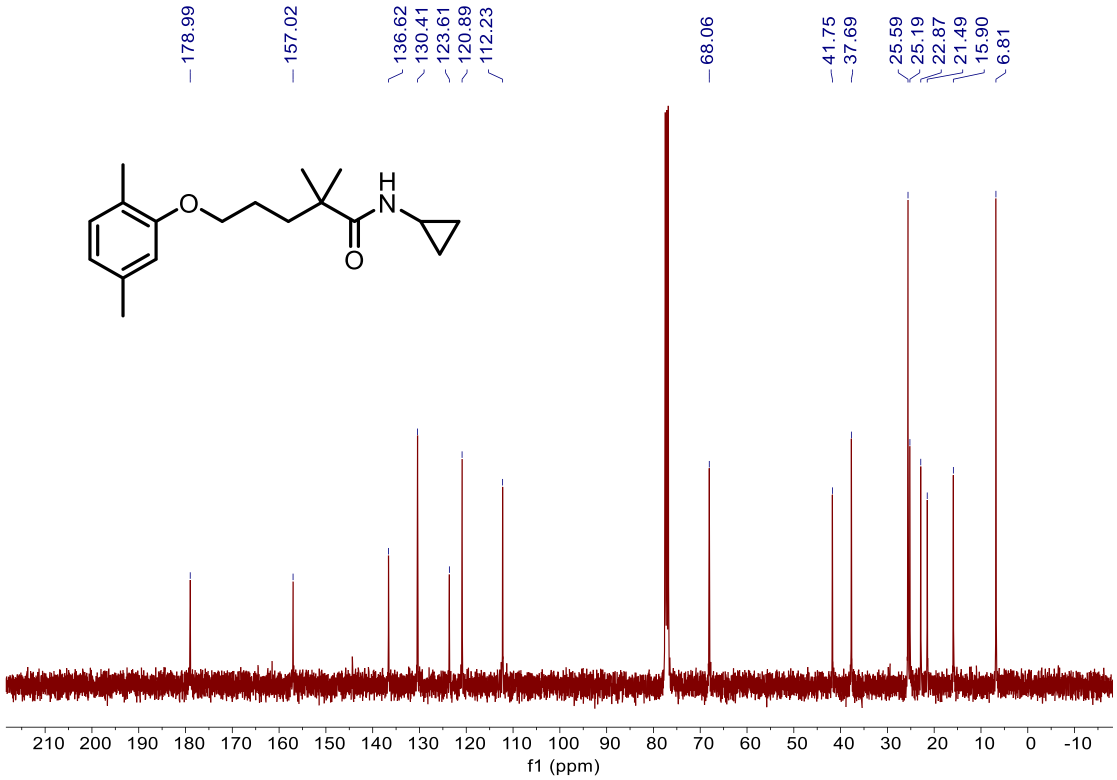

Supplementary Figure 91. $^{13}C\{^{1}H\}$ NMR spectrum of **1an** (101 MHz, Chloroform-*d*)

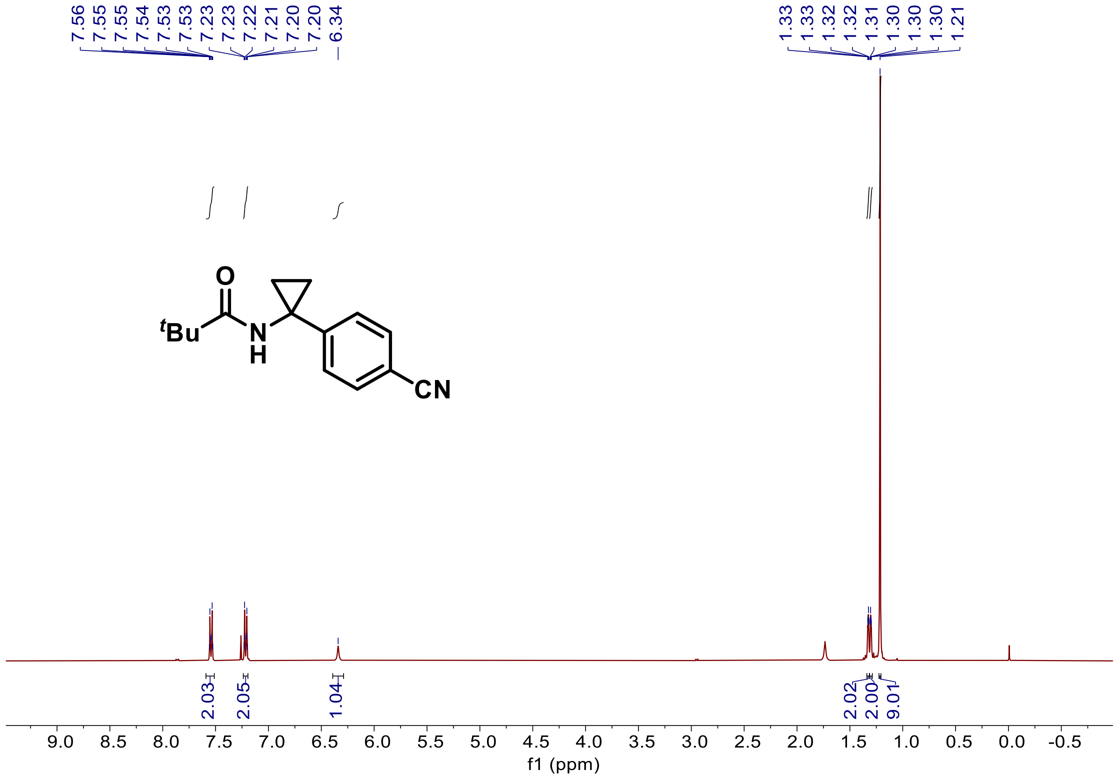

Supplementary Figure 92. ^{1}H NMR spectrum of **1ao** (400 MHz, Chloroform-*d*)

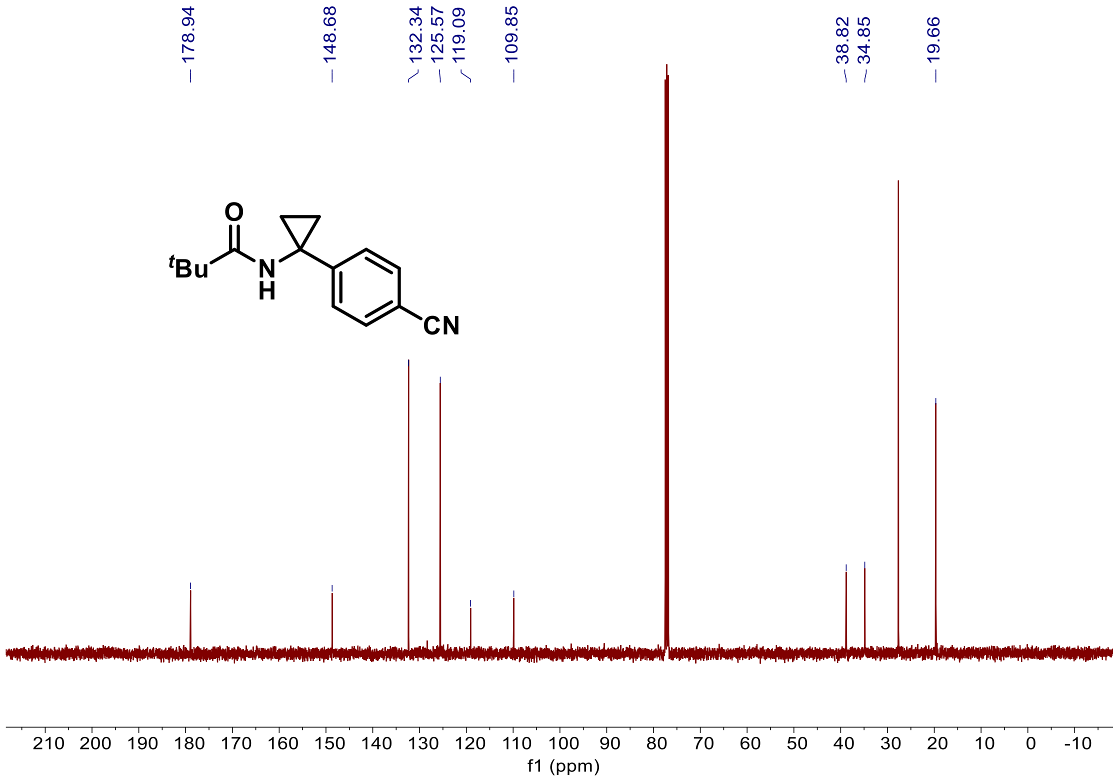

Supplementary Figure 93. $^{13}C\{^{1}H\}$ NMR spectrum of **1ao** (101 MHz, Chloroform-*d*)

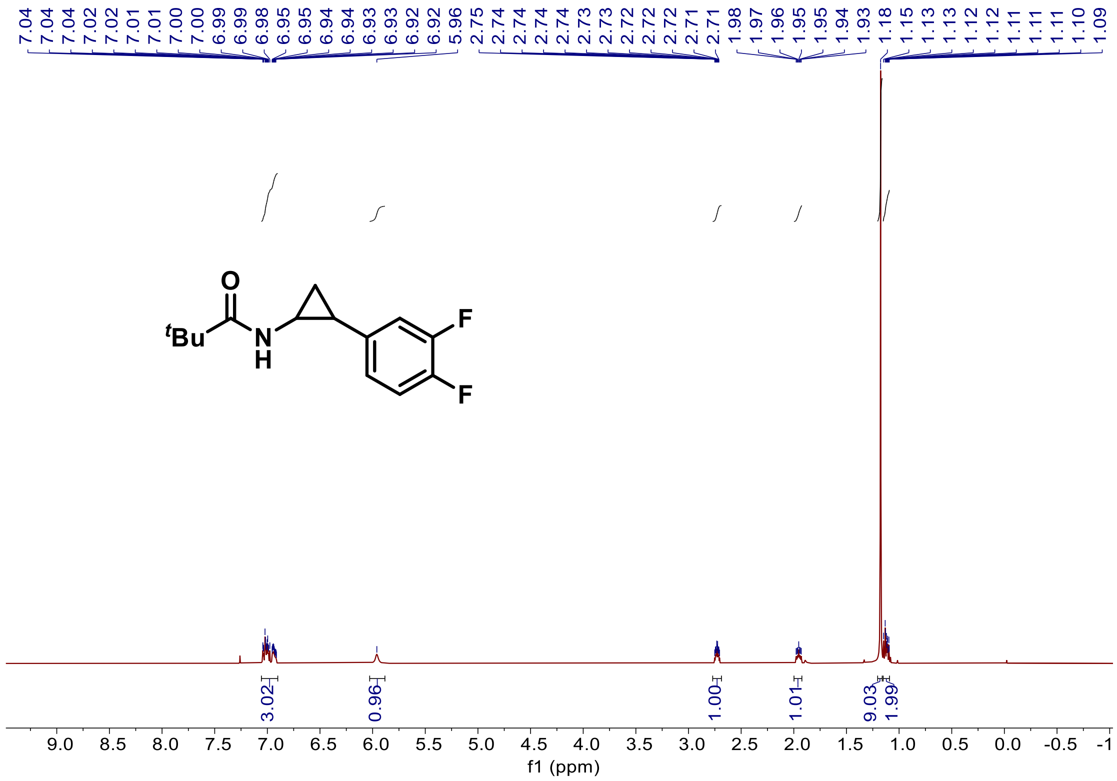

Supplementary Figure 94. ^{1}H NMR spectrum of **1ap** (400 MHz, Chloroform-*d*)

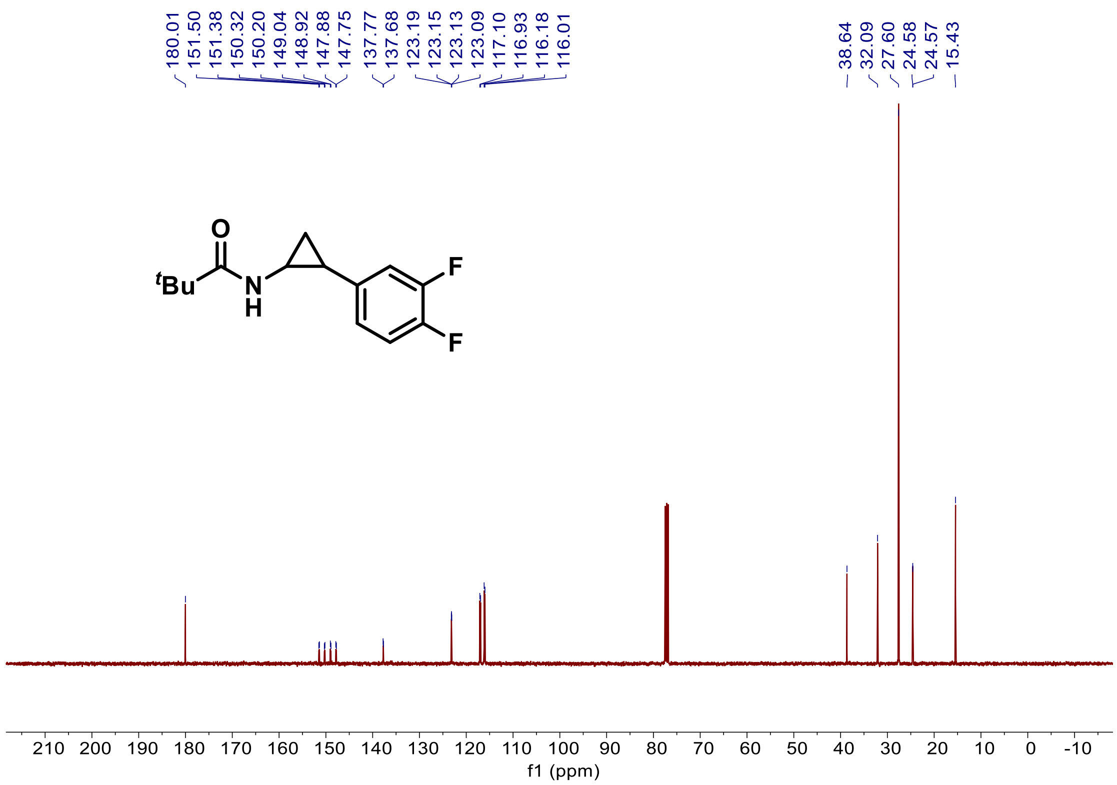

Supplementary Figure 95. $^{13}C\{^{1}H\}$ NMR spectrum of **1ap** (101 MHz, Chloroform-*d*)

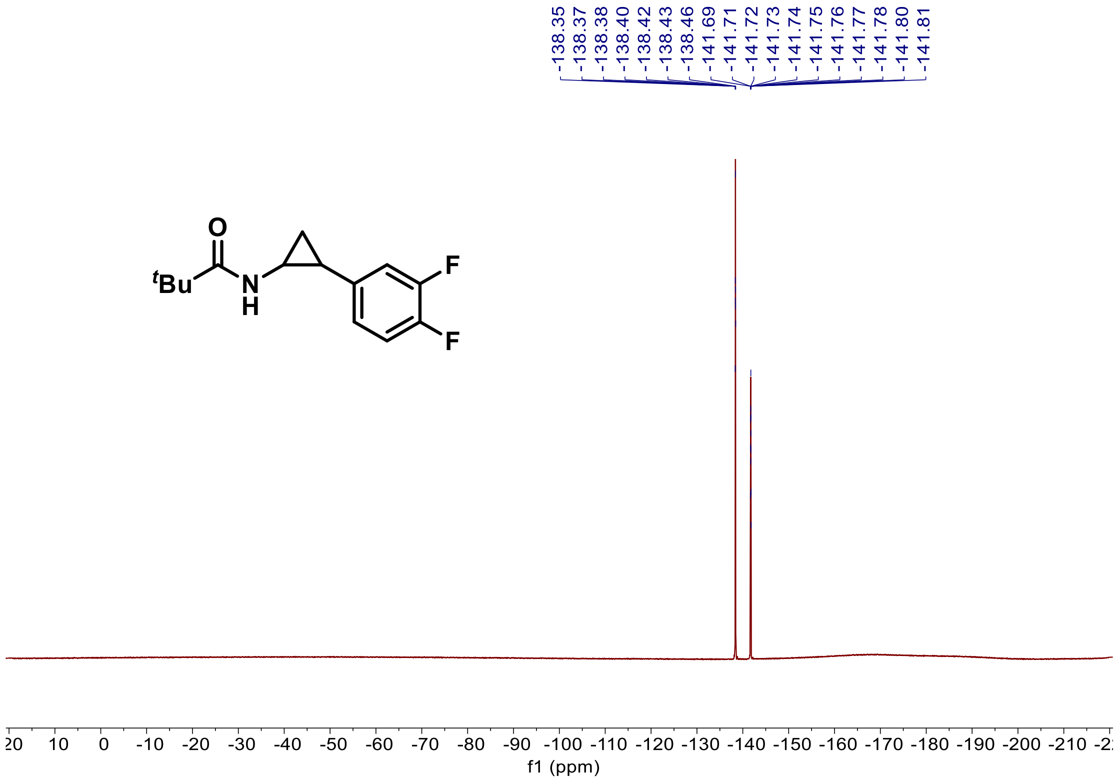

Supplementary Figure 96. ^{19}F NMR spectrum of **1ap** (376 MHz, Chloroform-*d*)

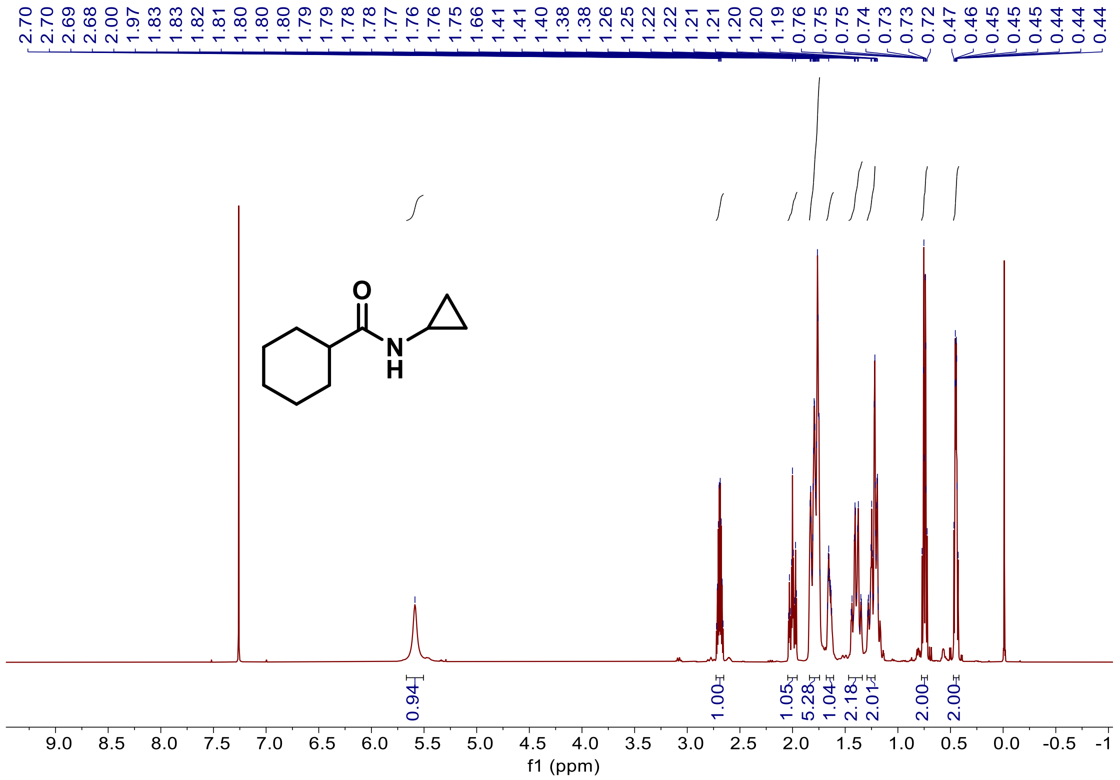

Supplementary Figure 97. ^{1}H NMR spectrum of **1aq** (400 MHz, Chloroform-*d*)

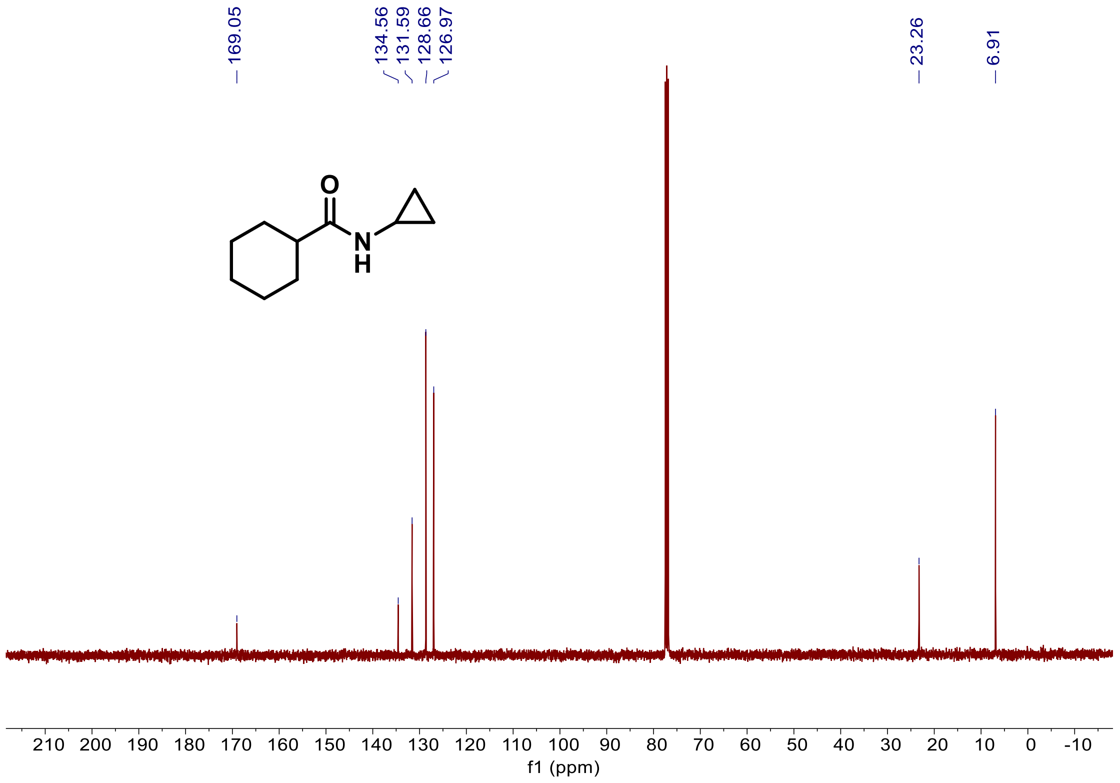

Supplementary Figure 98. ^{13}C{^{1}H} NMR spectrum of **1aq** (101 MHz, Chloroform-*d*)

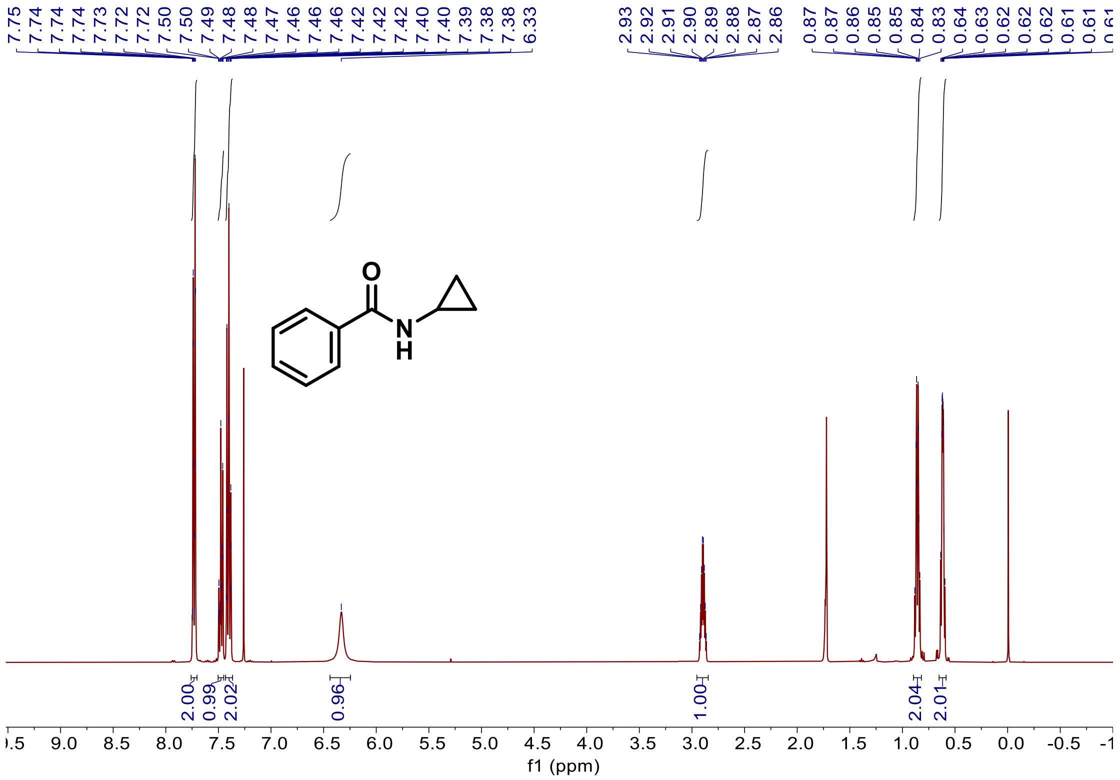

Supplementary Figure 99. ^{1}H NMR spectrum of **1ar** (400 MHz, Chloroform-*d*)

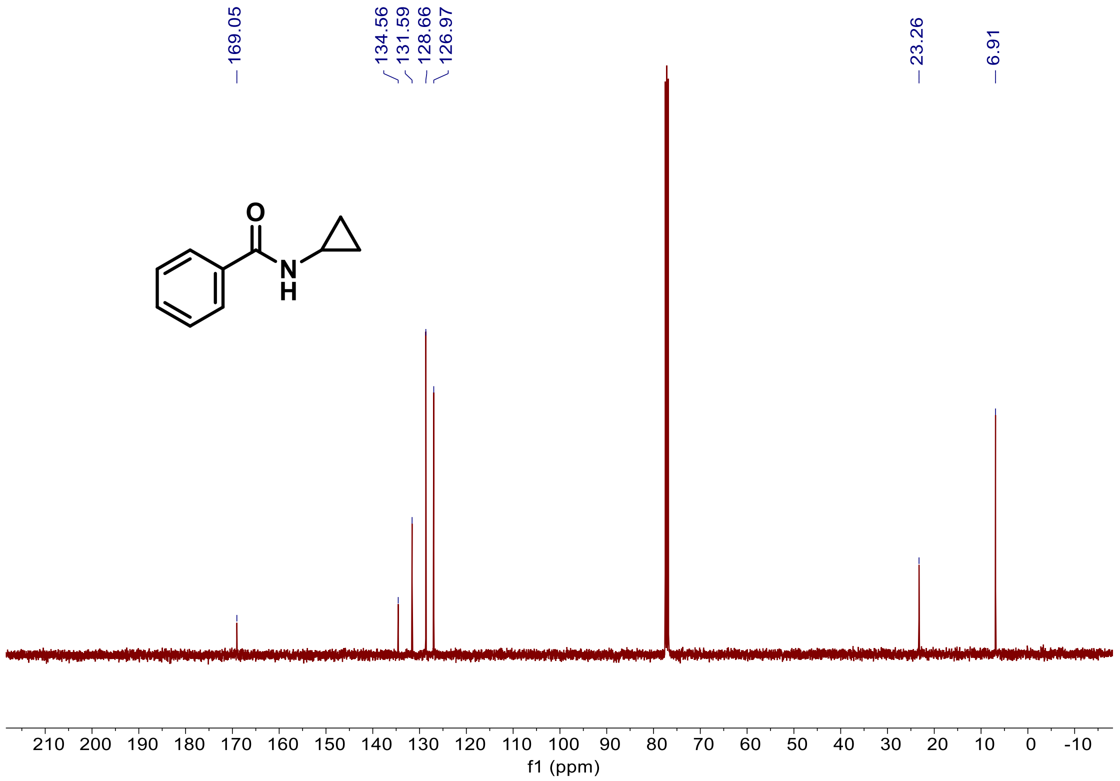

Supplementary Figure 100. ^{13}C{^{1}H} NMR spectrum of **1ar** (101 MHz, Chloroform-*d*)

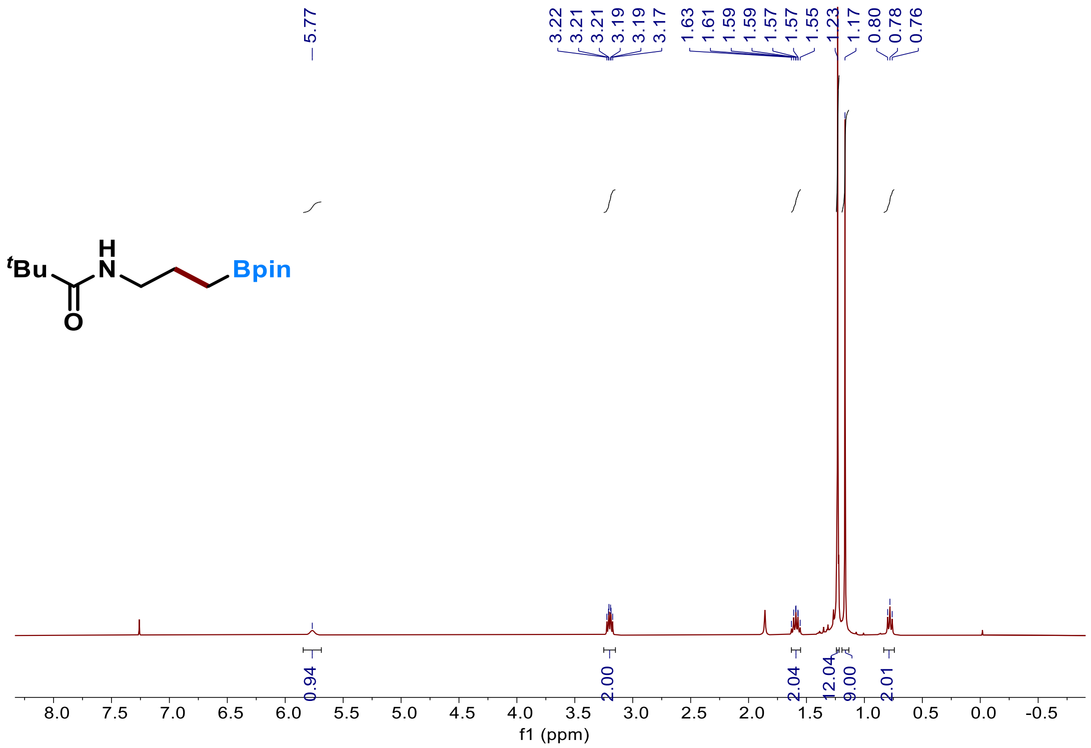

Supplementary Figure 101. ^{1}H NMR spectrum of **2a** (400 MHz, Chloroform-*d*)

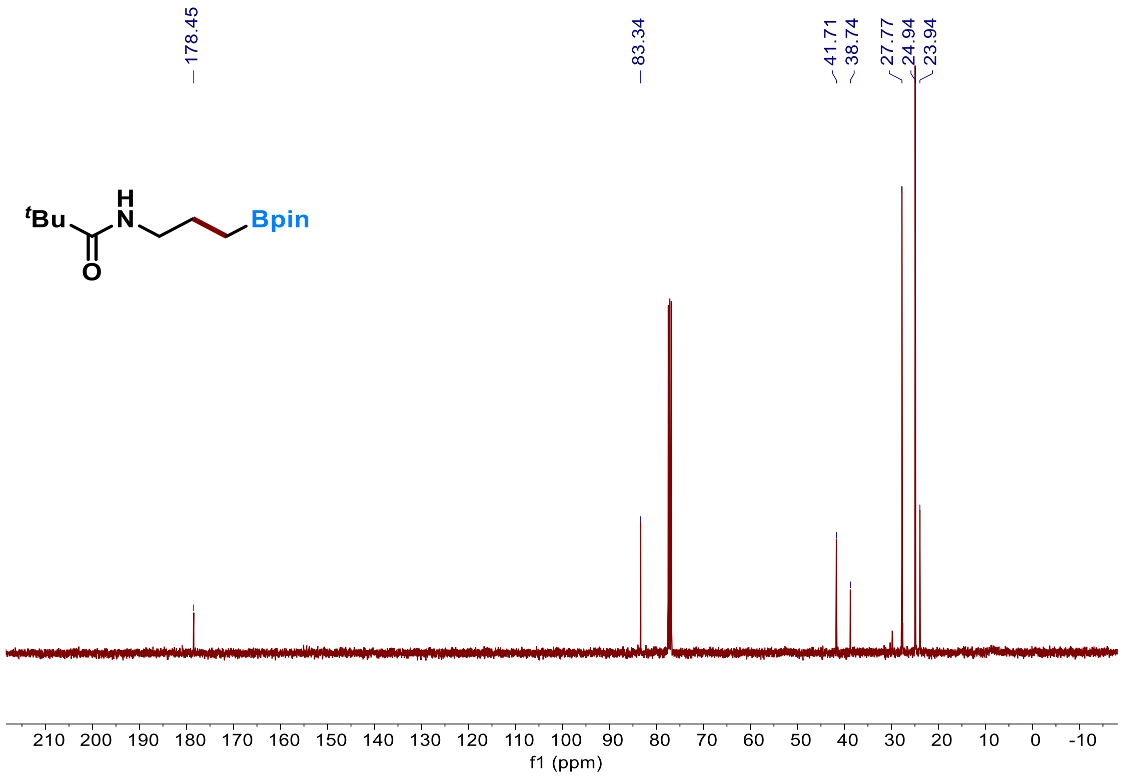

Supplementary Figure 102. ^{13}C{^{1}H} NMR spectrum of **2a** (101 MHz, Chloroform-*d*)

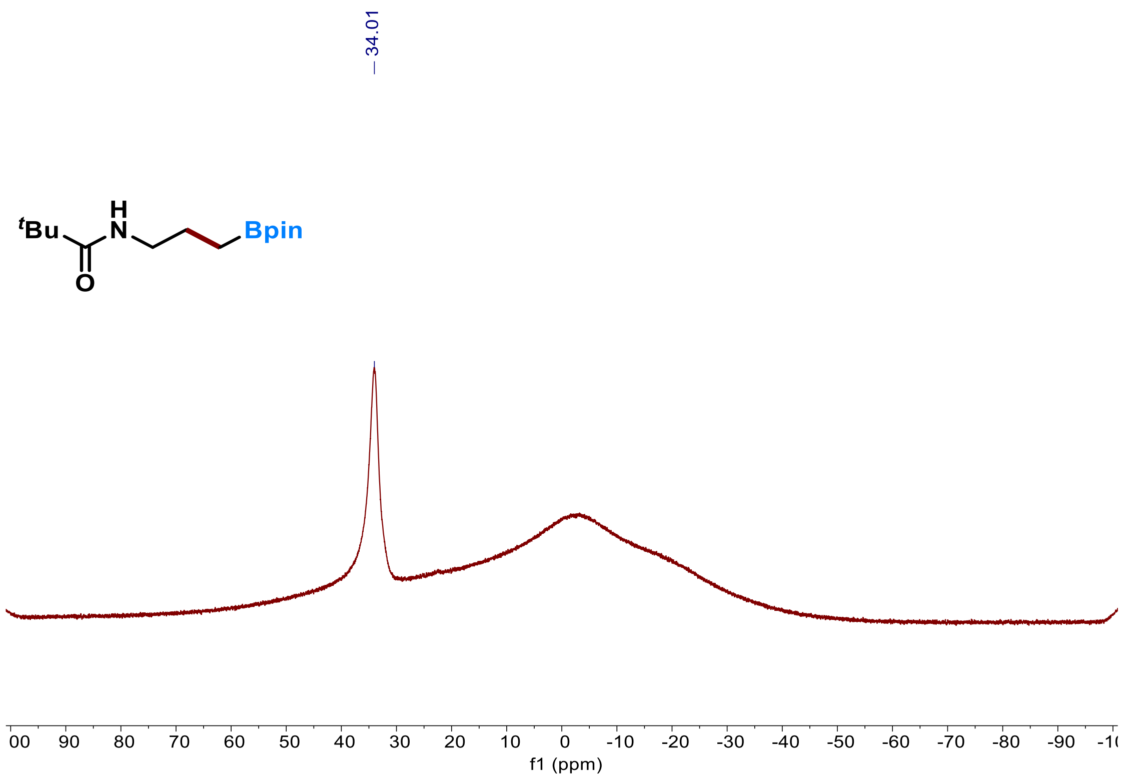

Supplementary Figure 103. ^{11}B NMR spectrum of **2a** (128 MHz, Chloroform-*d*)

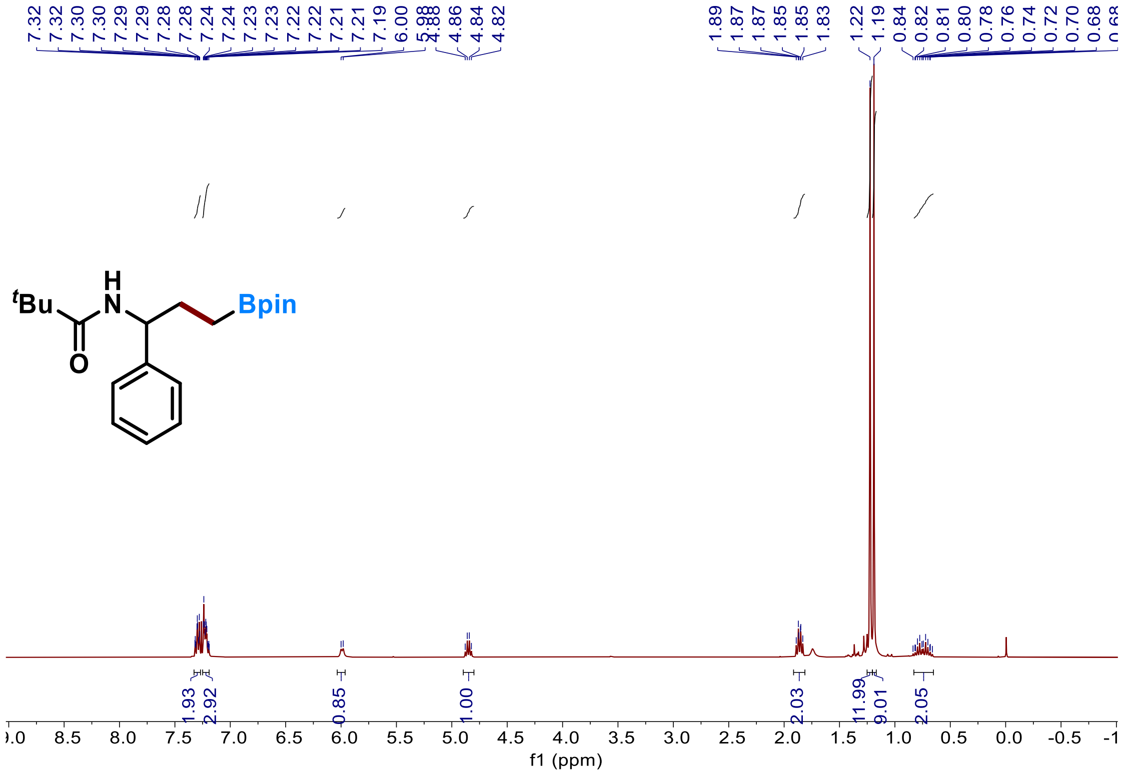

Supplementary Figure 104. ^{1}H NMR spectrum of **2b** (400 MHz, Chloroform-*d*)

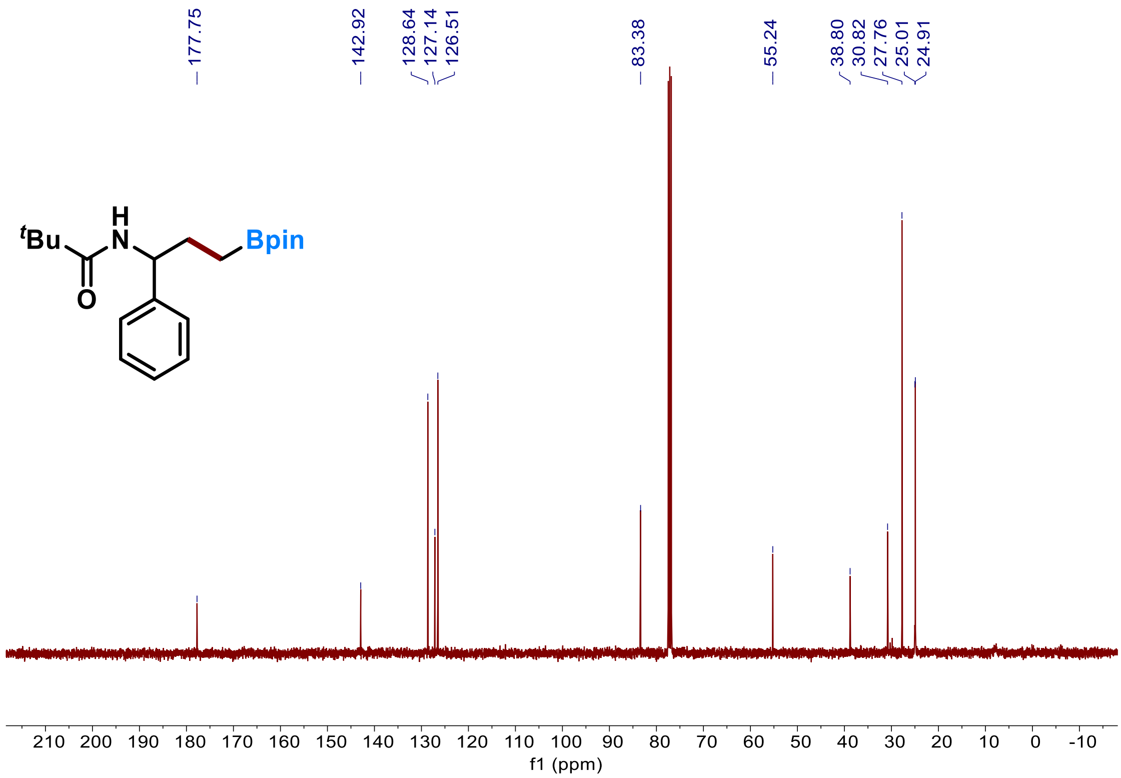

Supplementary Figure 105. $^{13}C\{^{1}H\}$ NMR spectrum of **2b** (101 MHz, Chloroform-*d*)

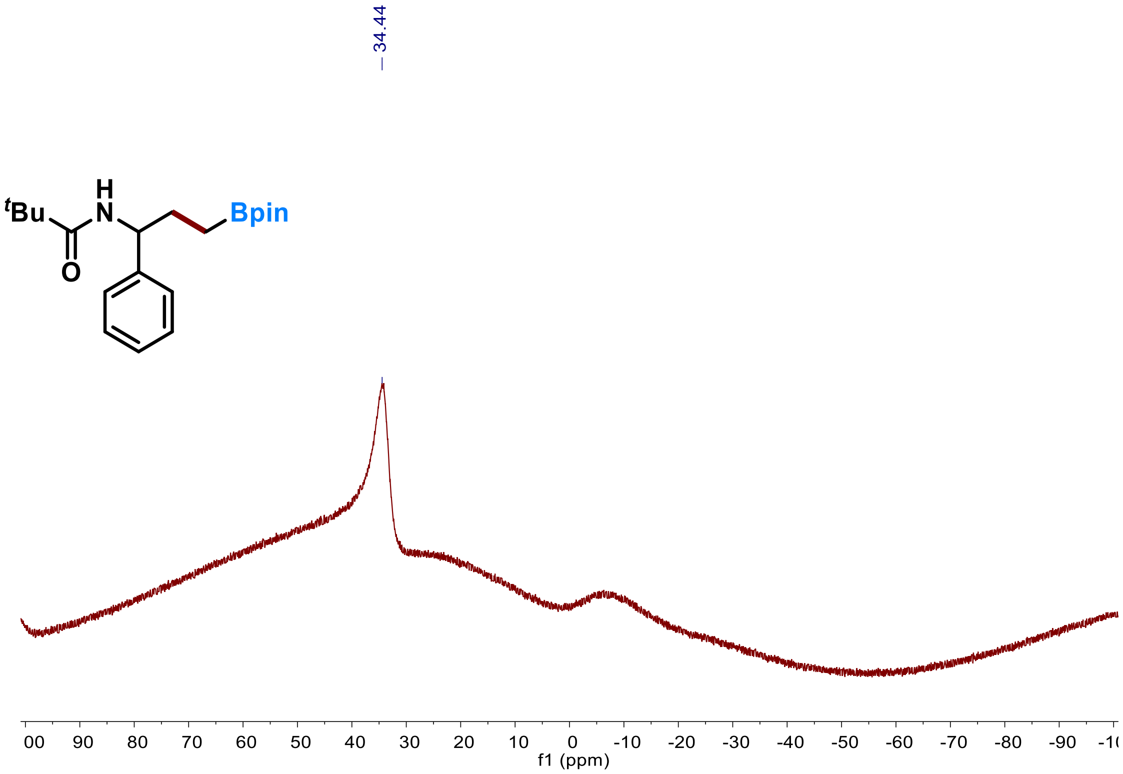

Supplementary Figure 106. ^{11}B NMR spectrum of **2b** (128 MHz, Chloroform-*d*)

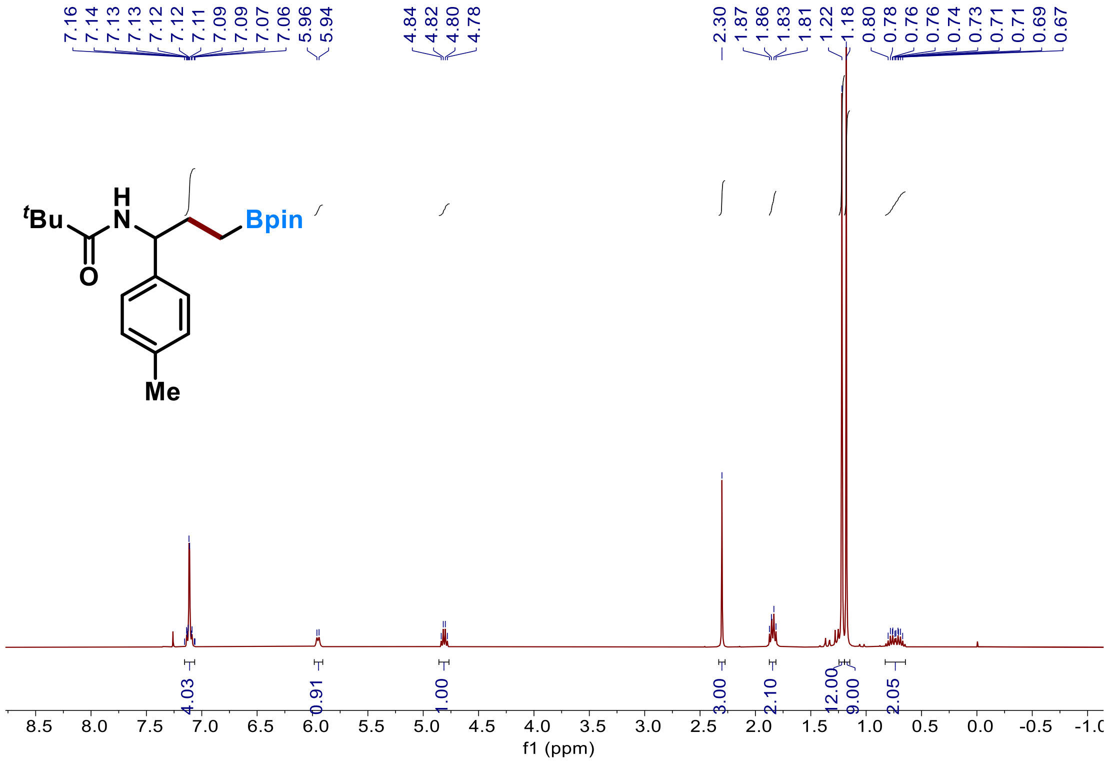

Supplementary Figure 107. ^{1}H NMR spectrum of **2c** (400 MHz, Chloroform-*d*)

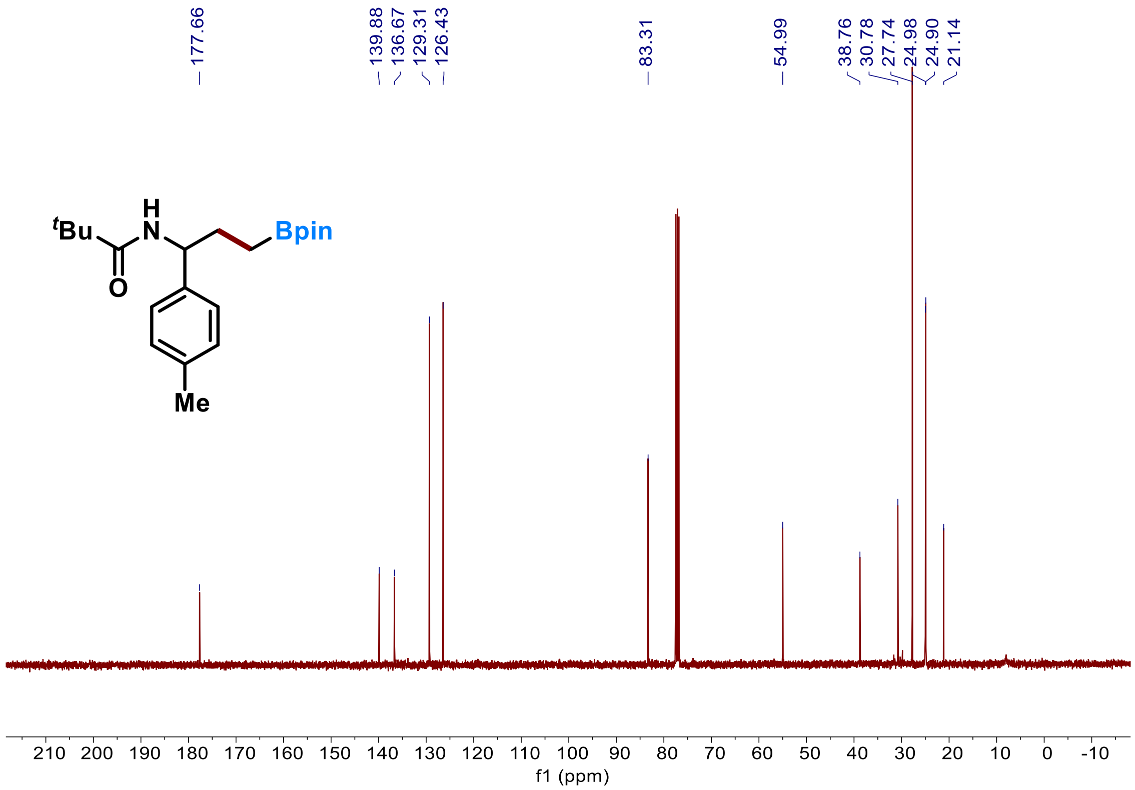

Supplementary Figure 108. $^{13}C\{^{1}H\}$ NMR spectrum of **2c** (101 MHz, Chloroform-*d*)

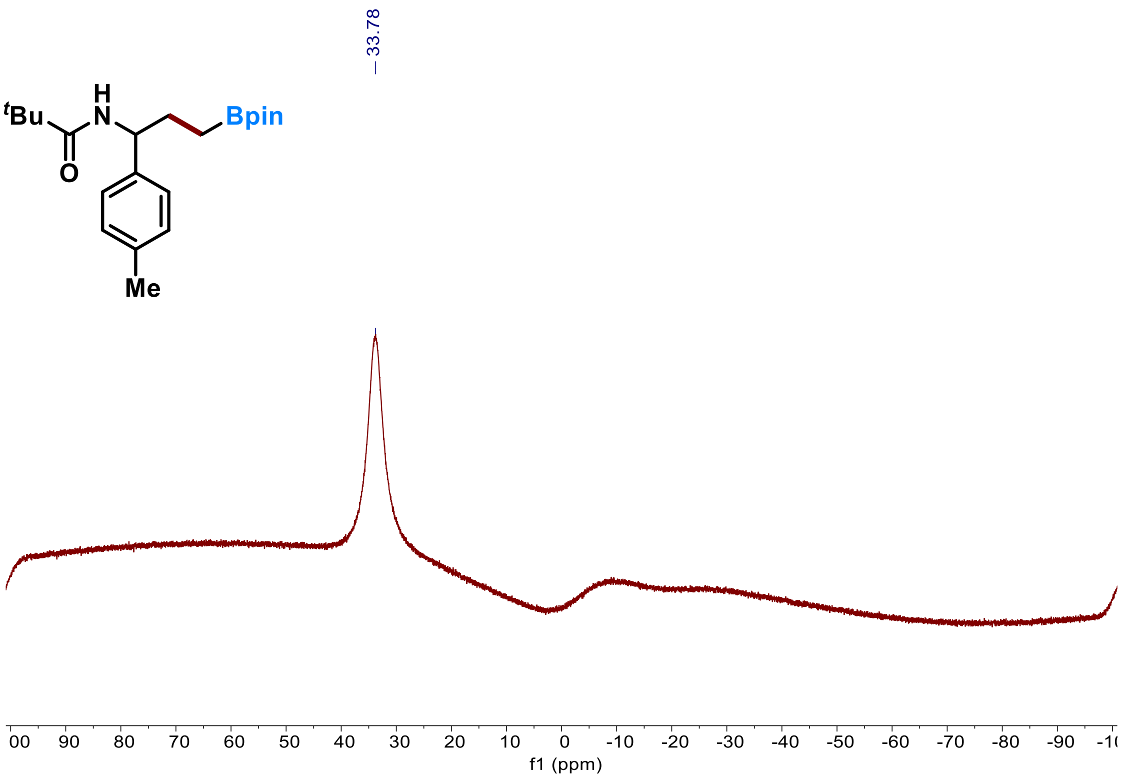

Supplementary Figure 109. ^{11}B NMR spectrum of **2c** (128 MHz, Chloroform-*d*)

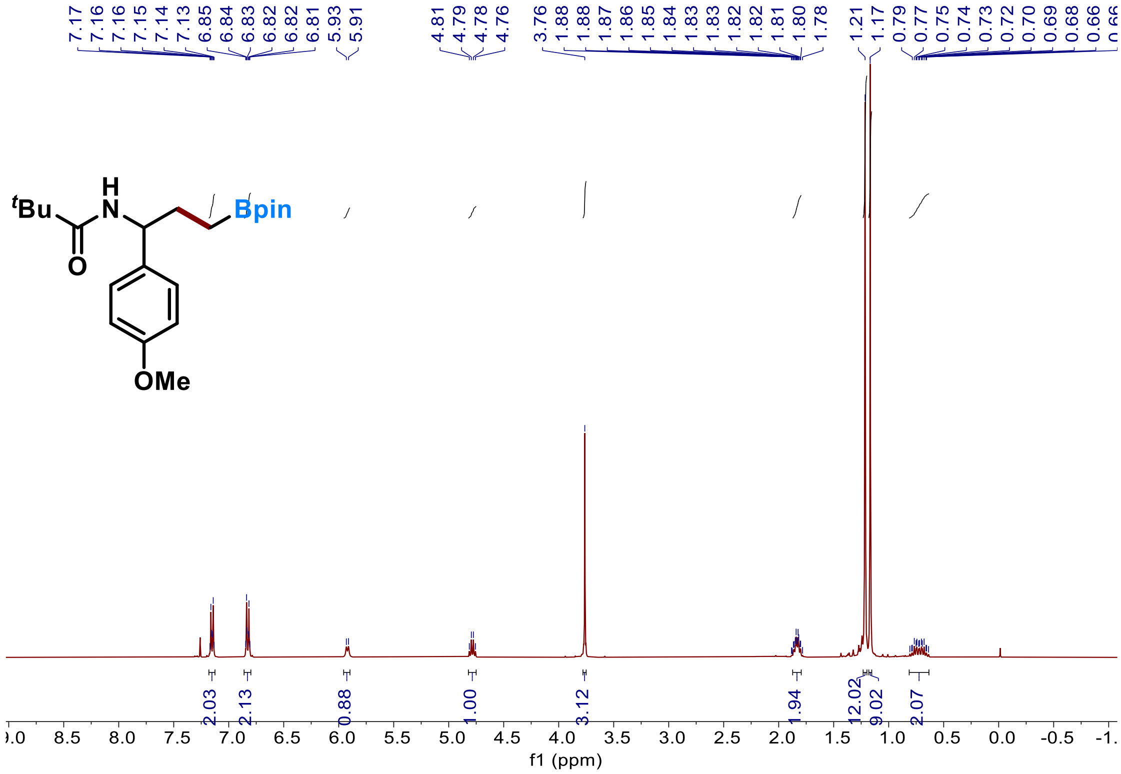

Supplementary Figure 110. ^{1}H NMR spectrum of **2d** (400 MHz, Chloroform-*d*)

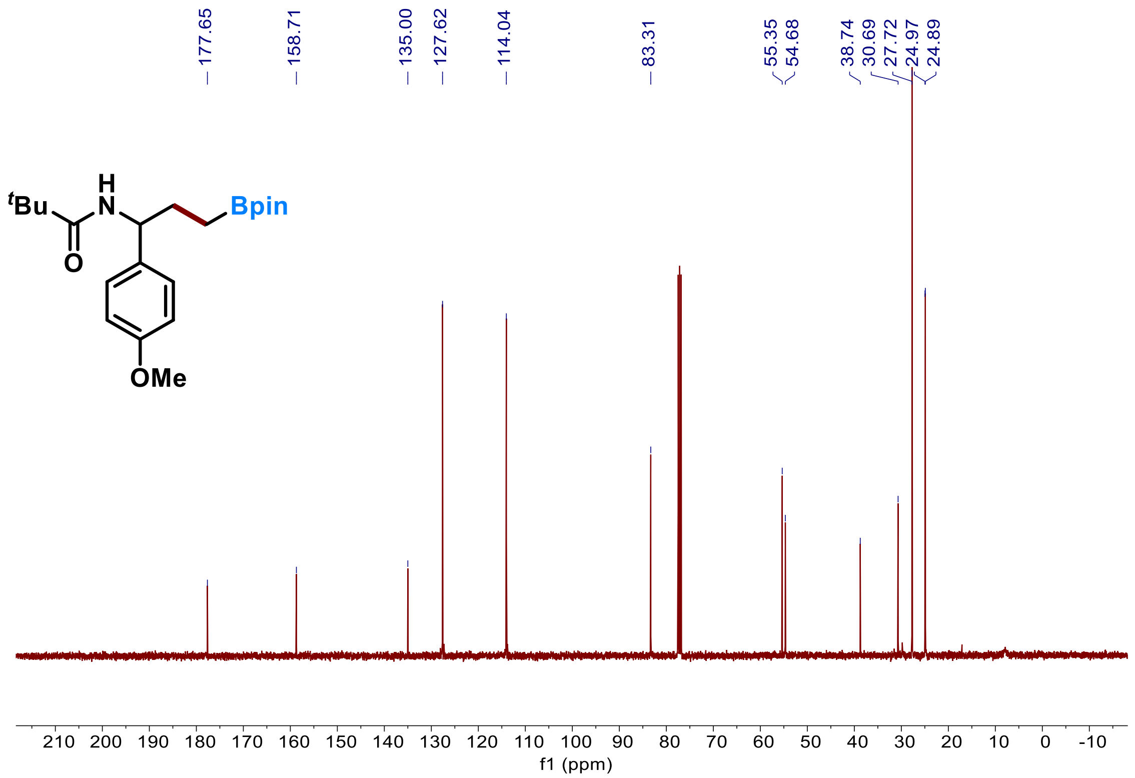

Supplementary Figure 111. $^{13}C\{^{1}H\}$ NMR spectrum of **2d** (101 MHz, Chloroform-*d*)

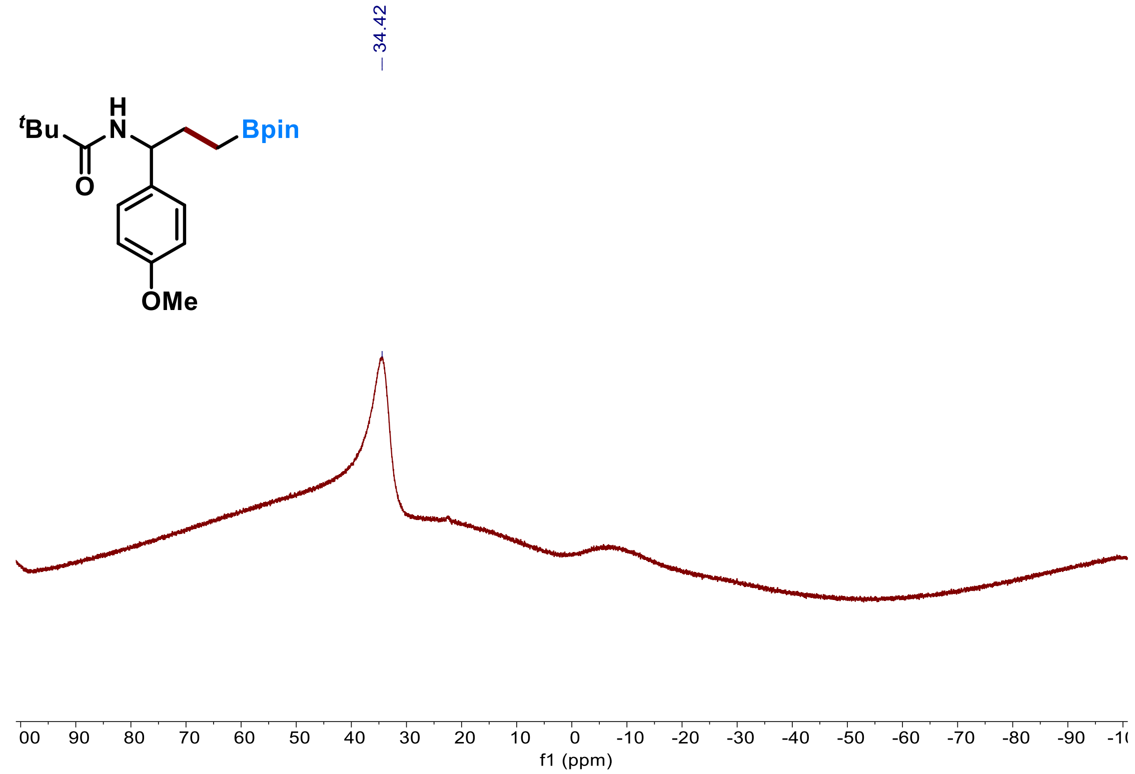

Supplementary Figure 112. ^{11}B NMR spectrum of **2d** (128 MHz, Chloroform-*d*)

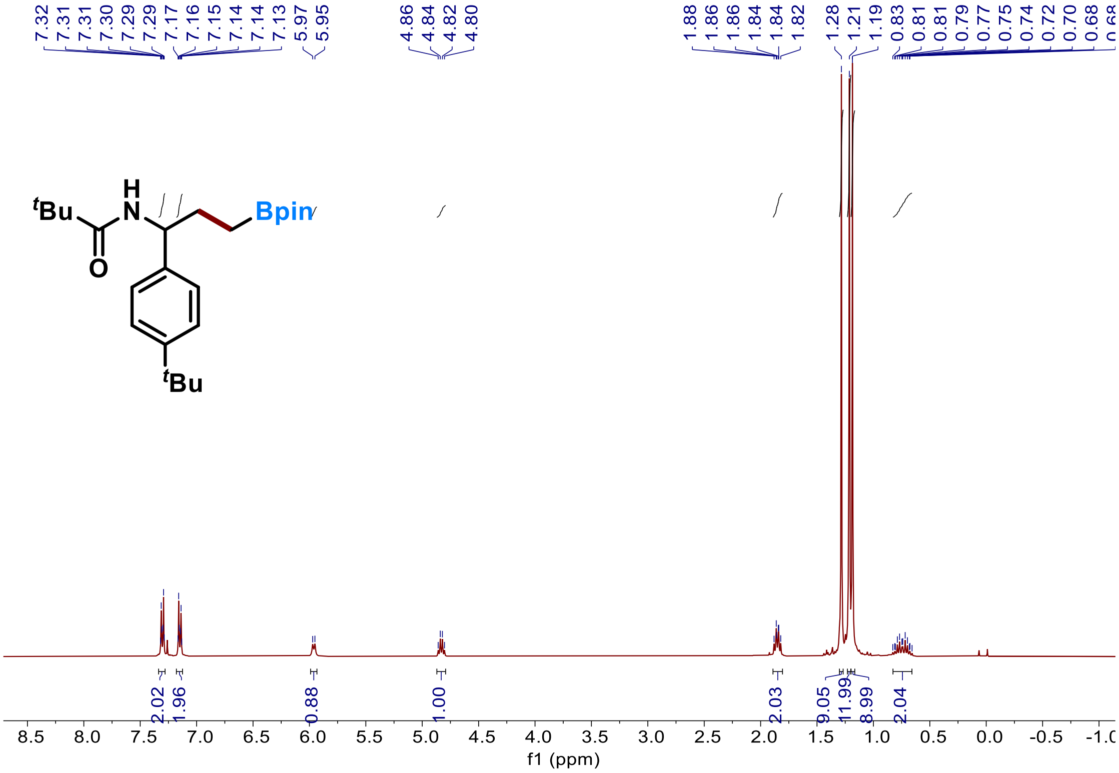

Supplementary Figure 113. ^{1}H NMR spectrum of **2e** (400 MHz, Chloroform-*d*)

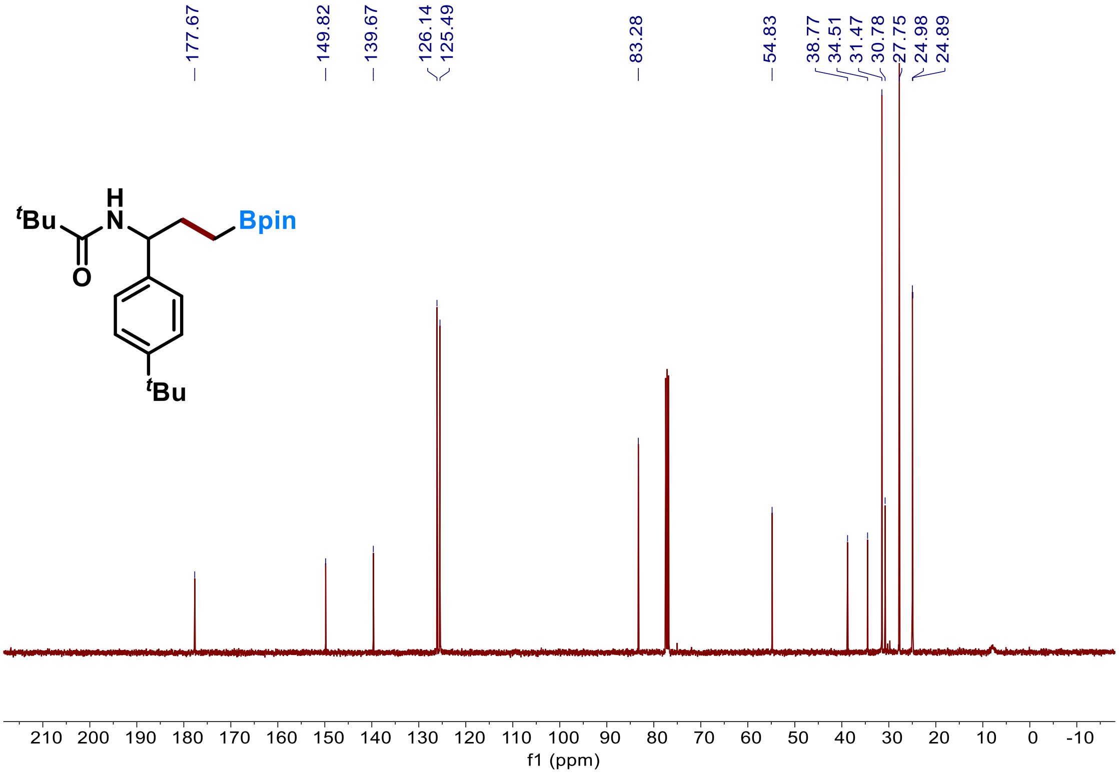

Supplementary Figure 114. $^{13}C\{^{1}H\}$ NMR spectrum of **2e** (101 MHz, Chloroform-*d*)

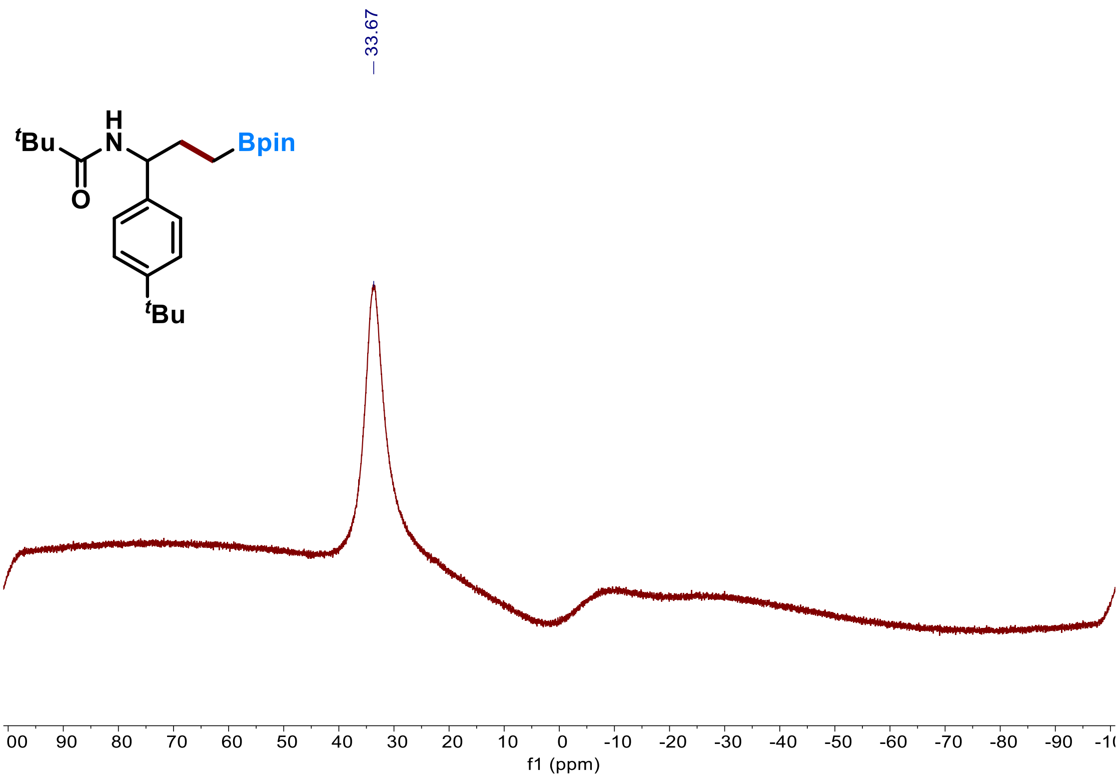

Supplementary Figure 115. ^{11}B NMR spectrum of **2e** (128 MHz, Chloroform-*d*)

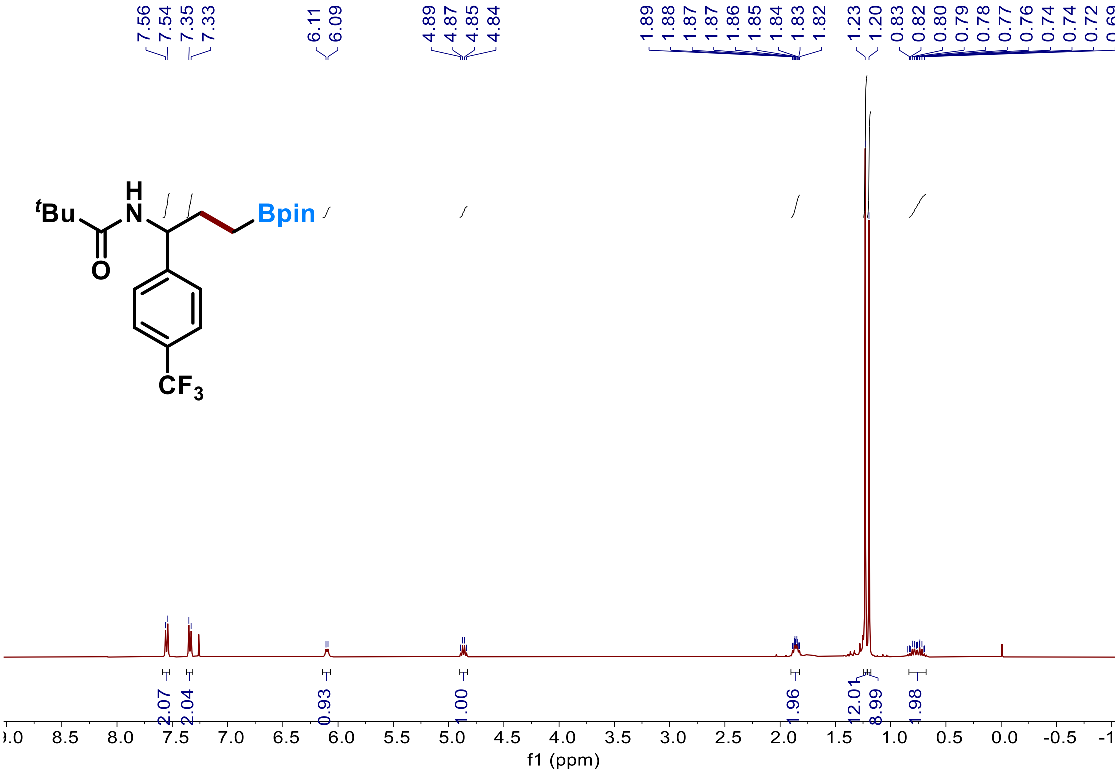

Supplementary Figure 116. ^{1}H NMR spectrum of **2f** (400 MHz, Chloroform-*d*)

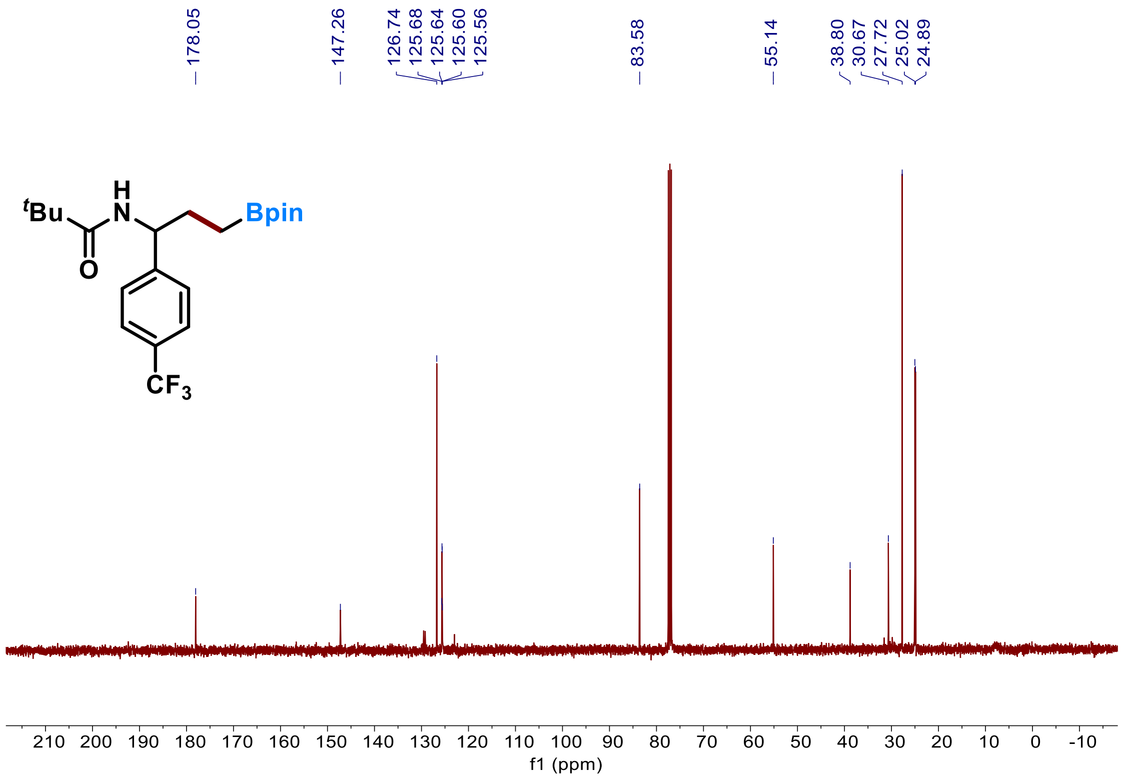

Supplementary Figure 117. $^{13}C\{^{1}H\}$ NMR spectrum of **2f** (101 MHz, Chloroform-*d*)

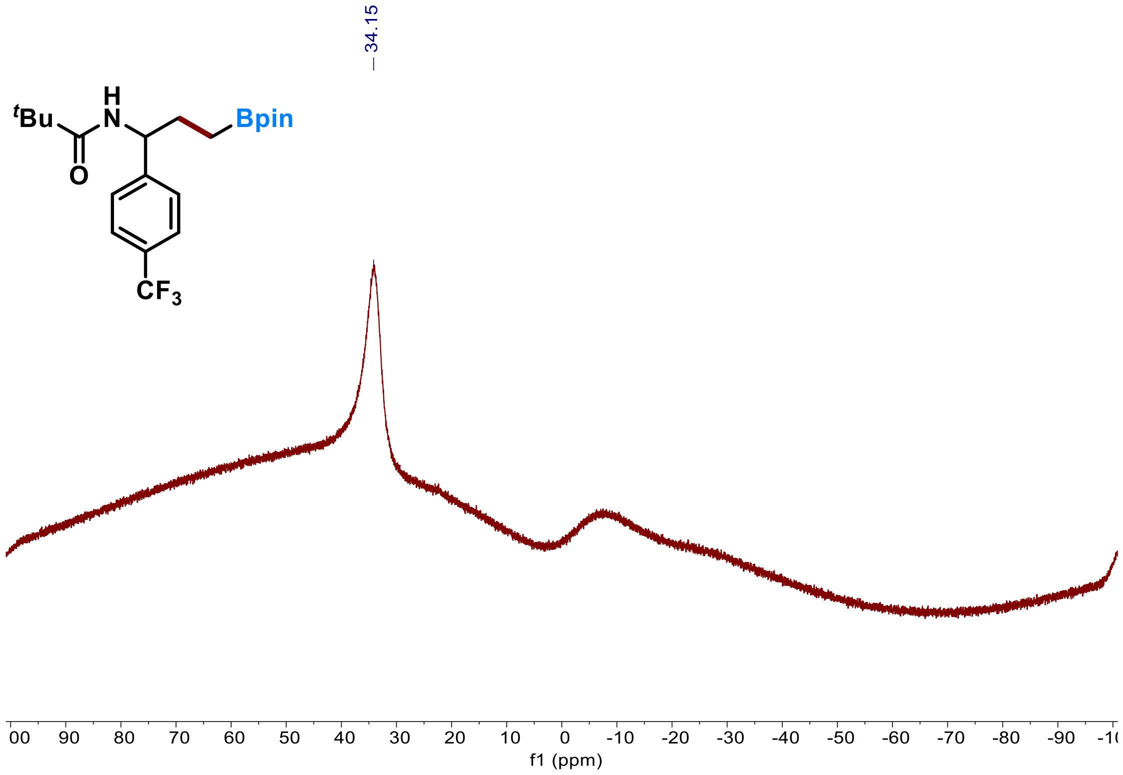

Supplementary Figure 118. ^{11}B NMR spectrum of **2f** (128 MHz, Chloroform-*d*)

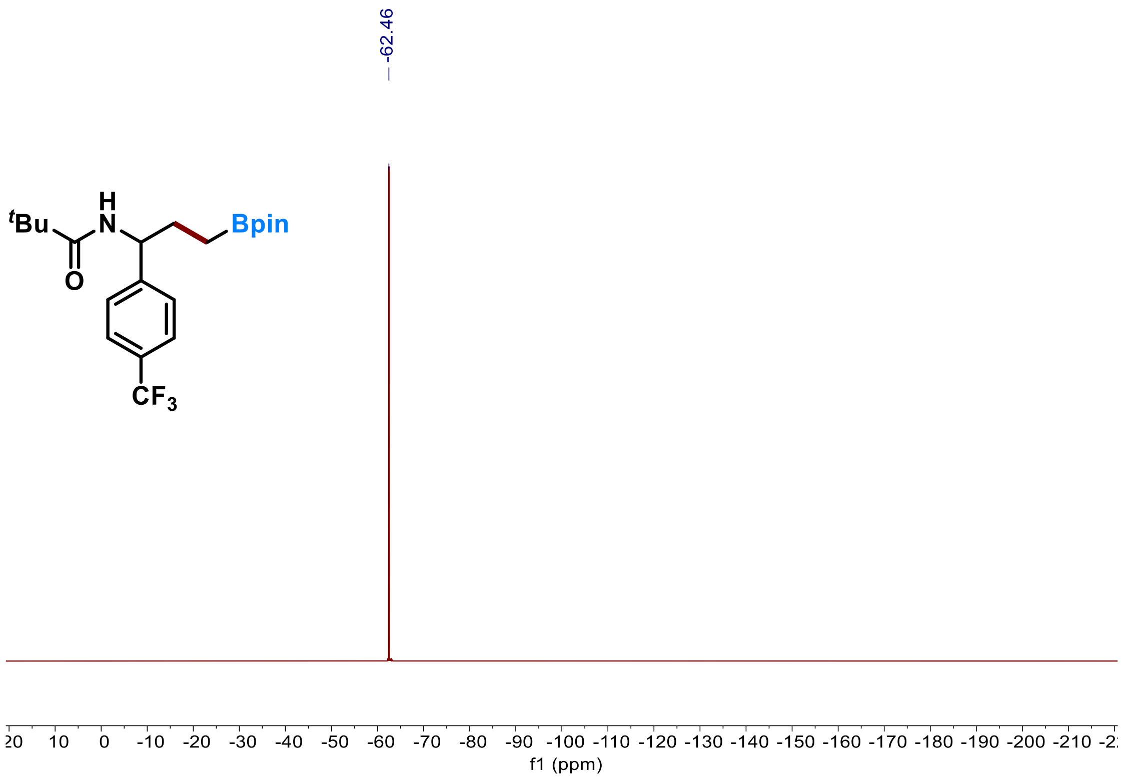

Supplementary Figure 119. ^{19}F NMR spectrum of **2f** (376 MHz, Chloroform-*d*)

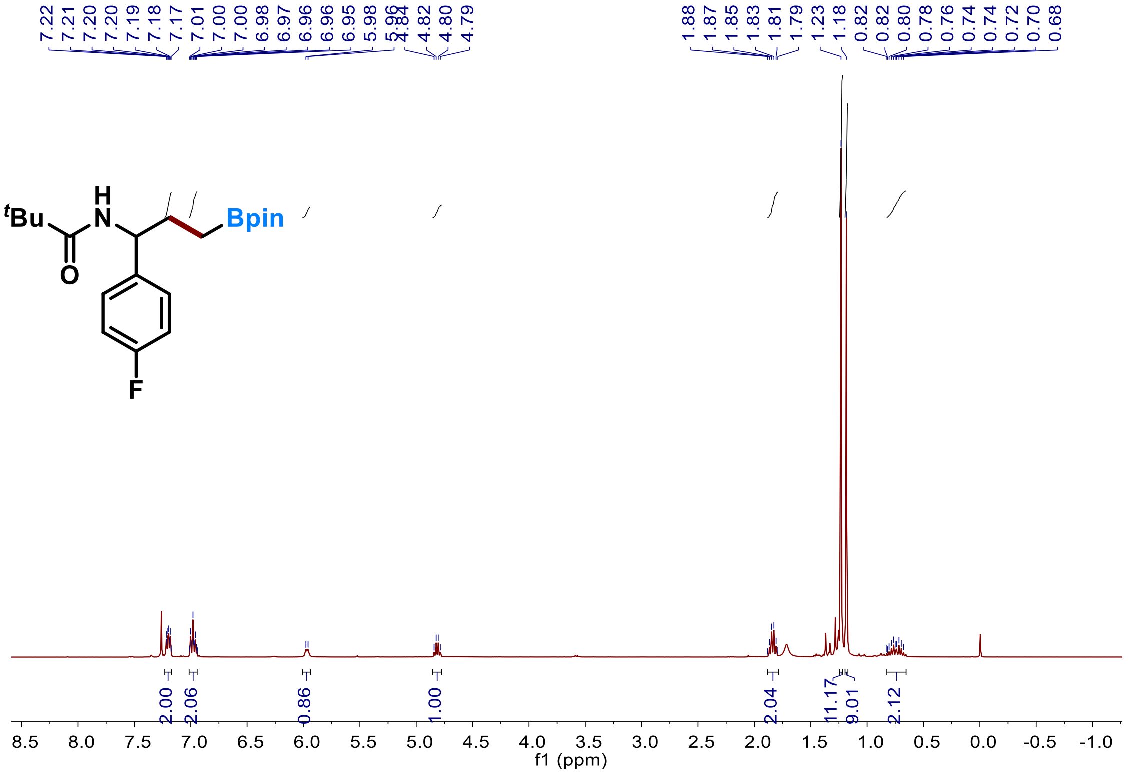

Supplementary Figure 120. ^{1}H NMR spectrum of **2g** (400 MHz, Chloroform-*d*)

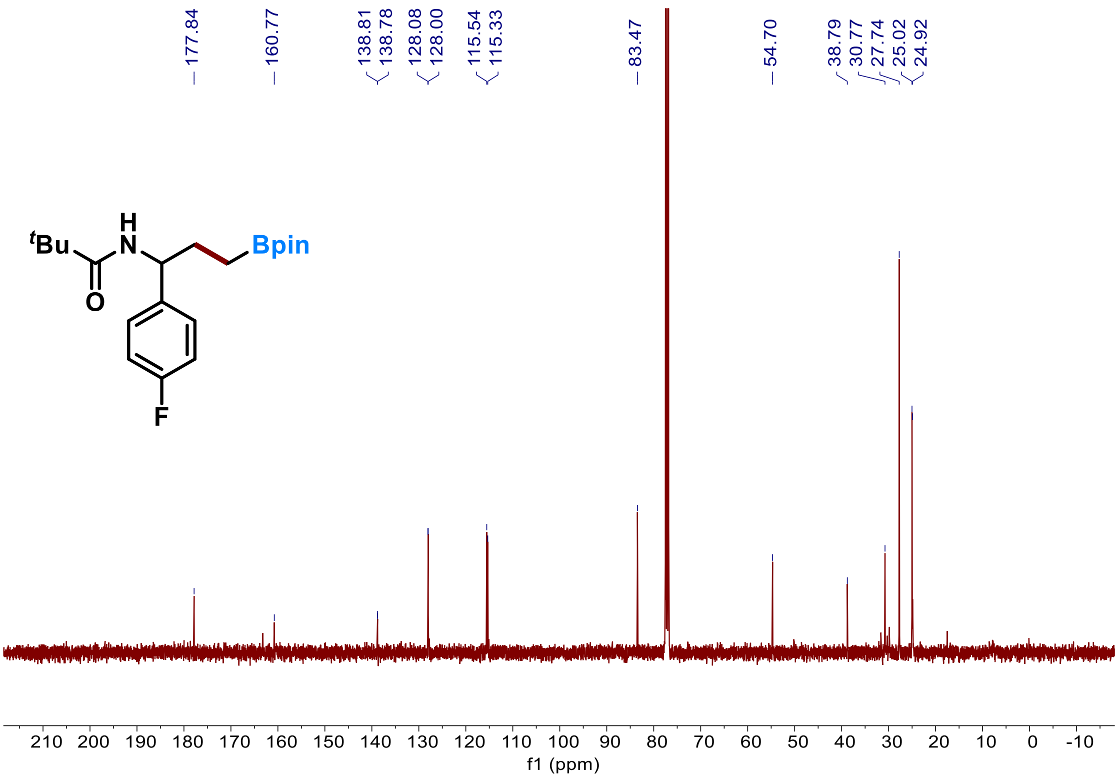

Supplementary Figure 121. $^{13}C\{^{1}H\}$ NMR spectrum of **2g** (101 MHz, Chloroform-*d*)

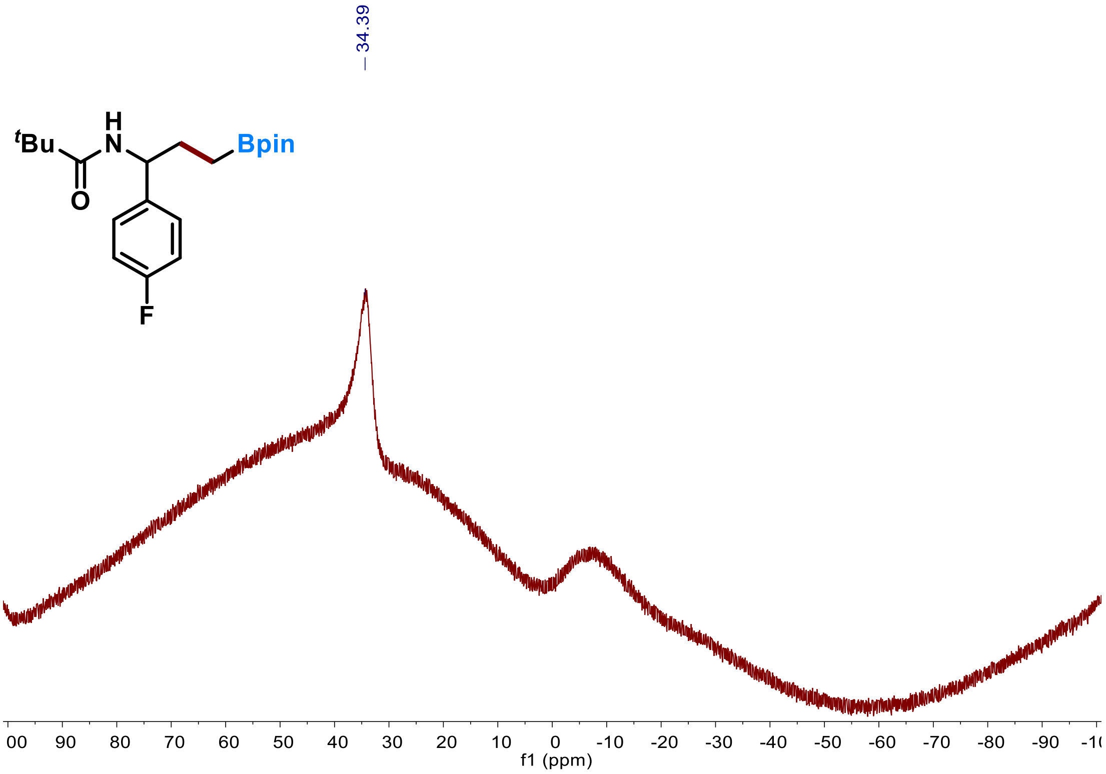

Supplementary Figure 122. ^{11}B NMR spectrum of **2g** (128 MHz, Chloroform-*d*)

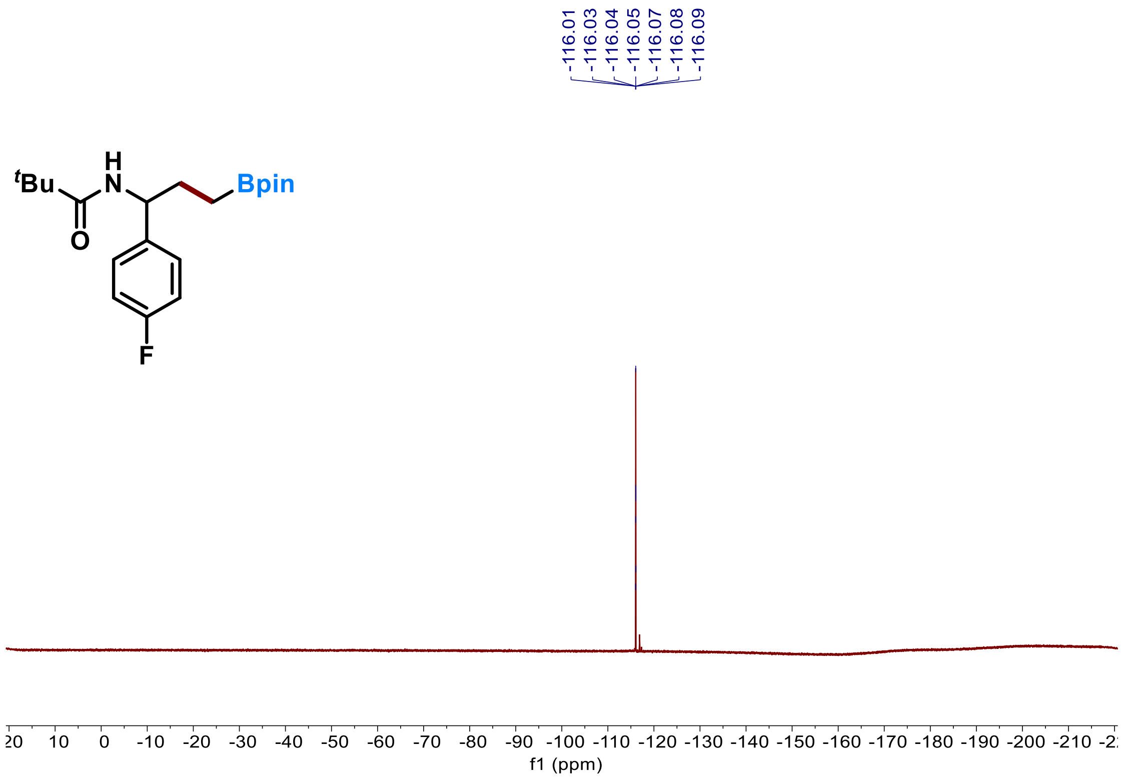

Supplementary Figure 123. ^{19}F NMR spectrum of **2g** (376 MHz, Chloroform-*d*)

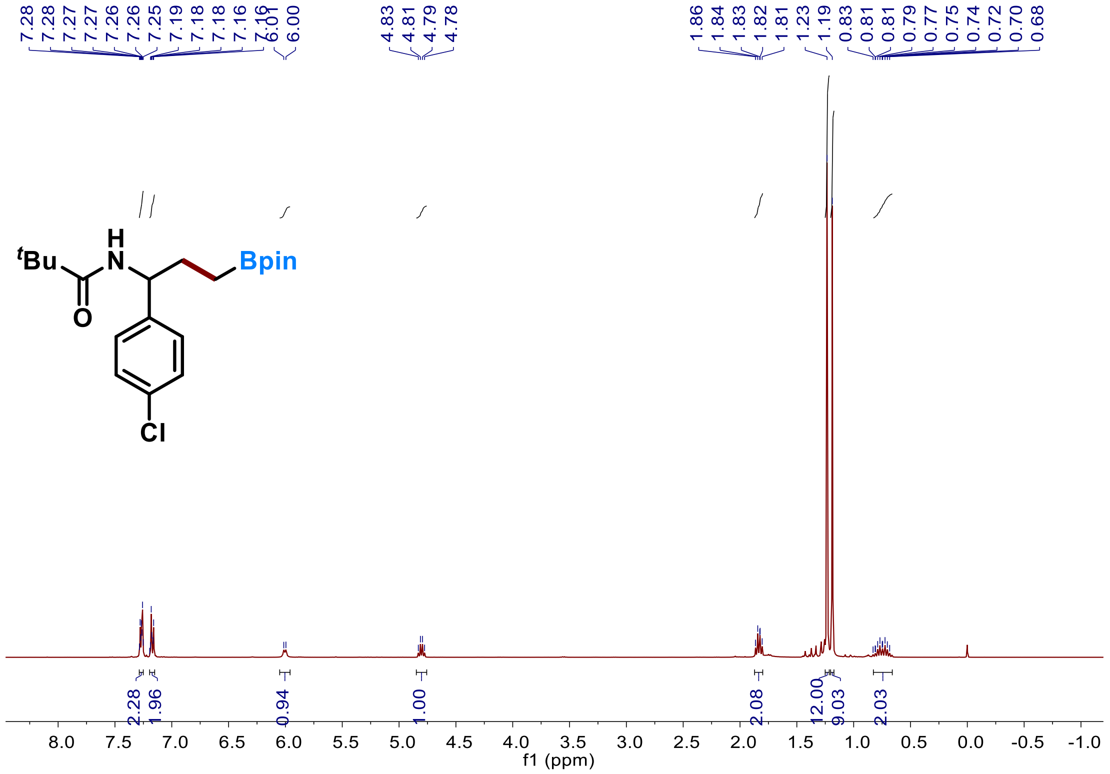

Supplementary Figure 124. ^{1}H NMR spectrum of **2h** (400 MHz, Chloroform-*d*)

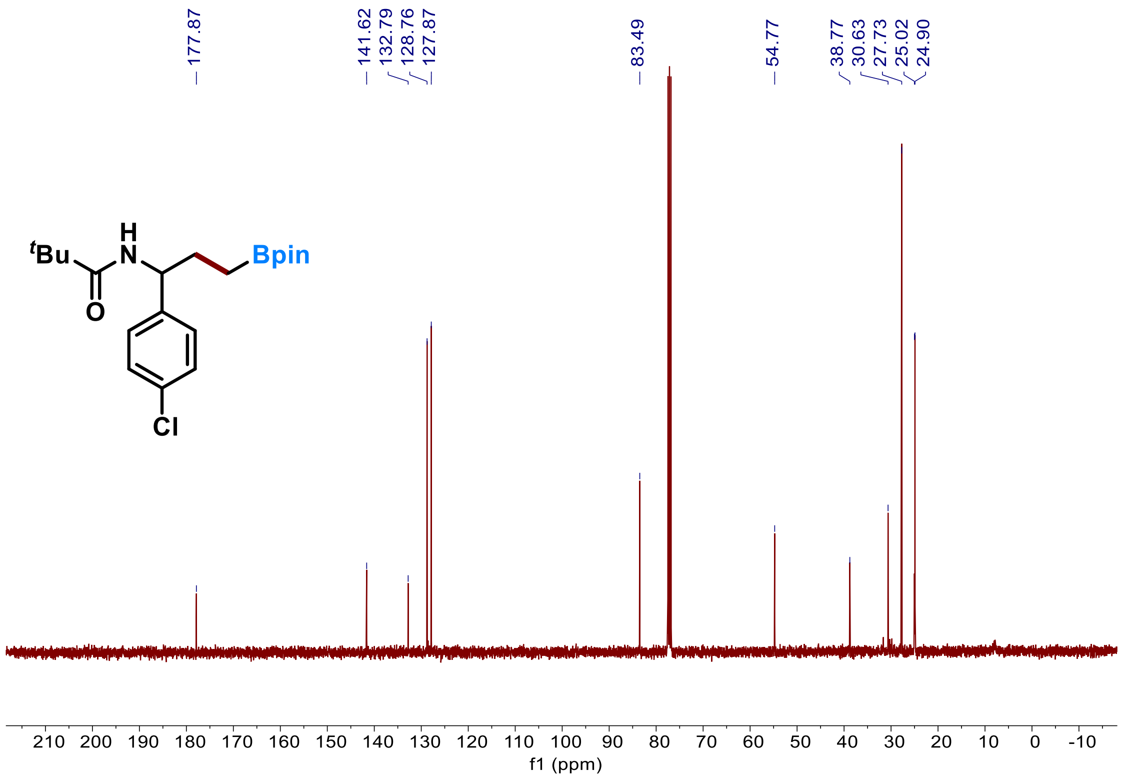

Supplementary Figure 125. $^{13}C\{^{1}H\}$ NMR spectrum of **2h** (101 MHz, Chloroform-*d*)

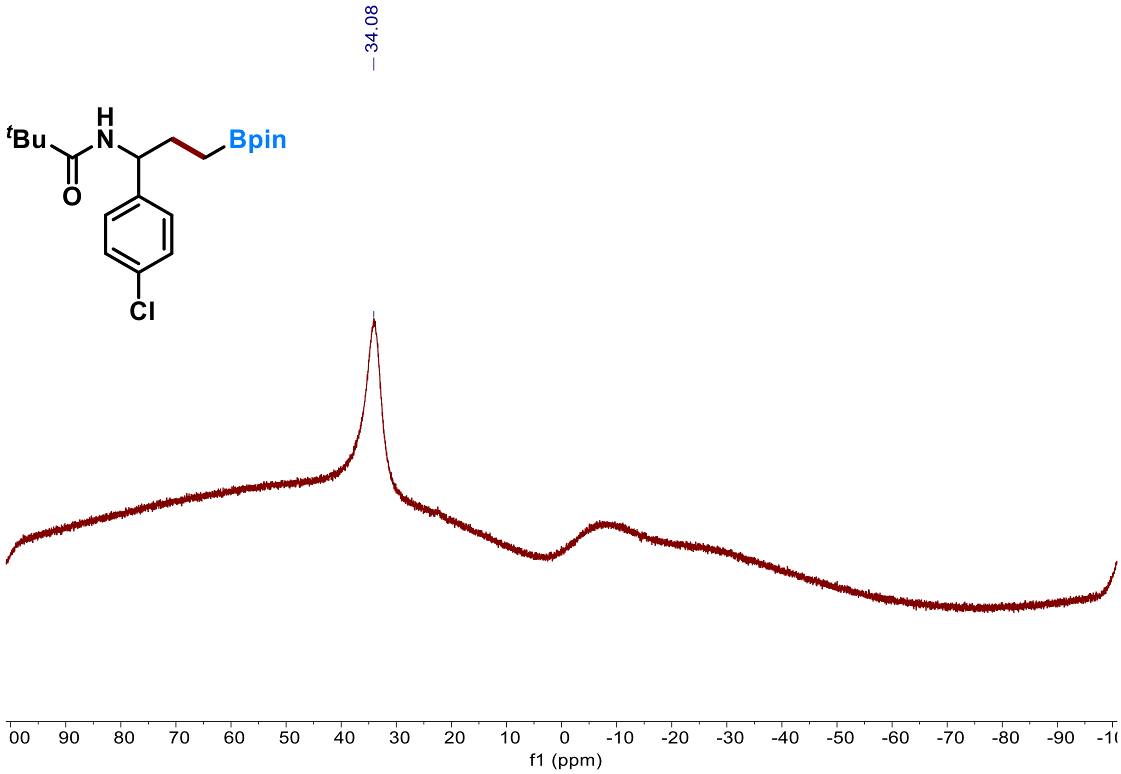

Supplementary Figure 126. ^{11}B NMR spectrum of **2h** (128 MHz, Chloroform-*d*)

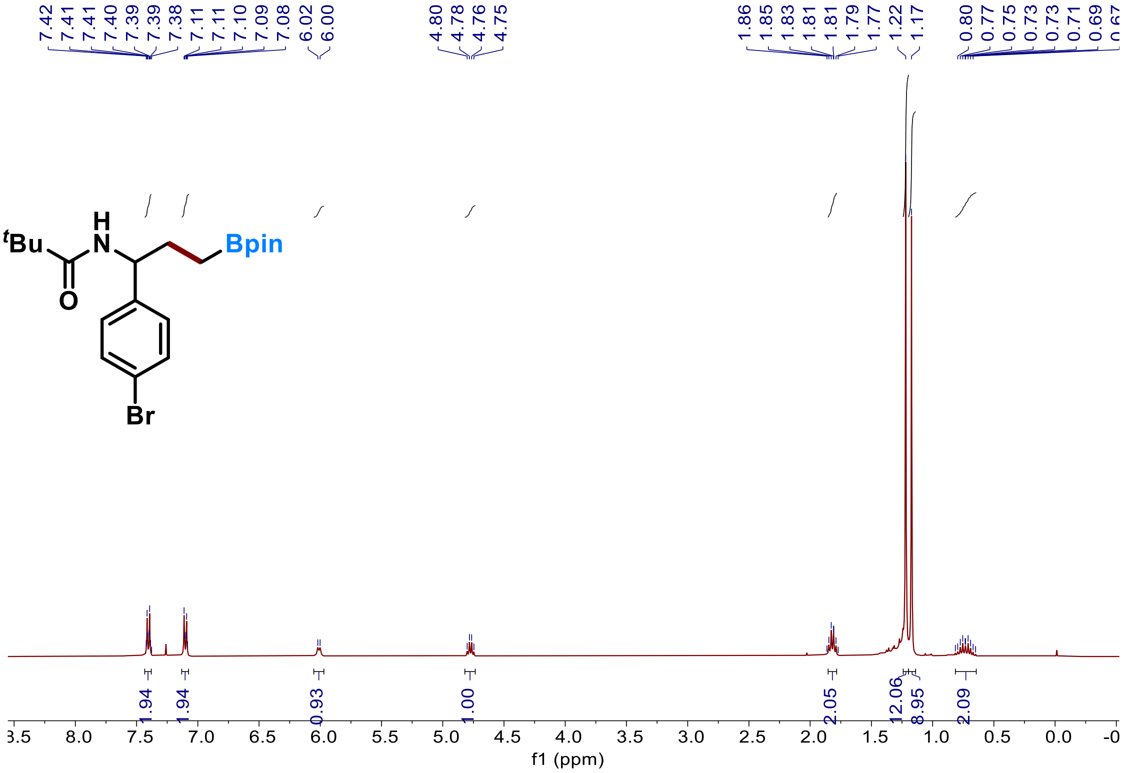

Supplementary Figure 127. ^{1}H NMR spectrum of **2i** (400 MHz, Chloroform-*d*)

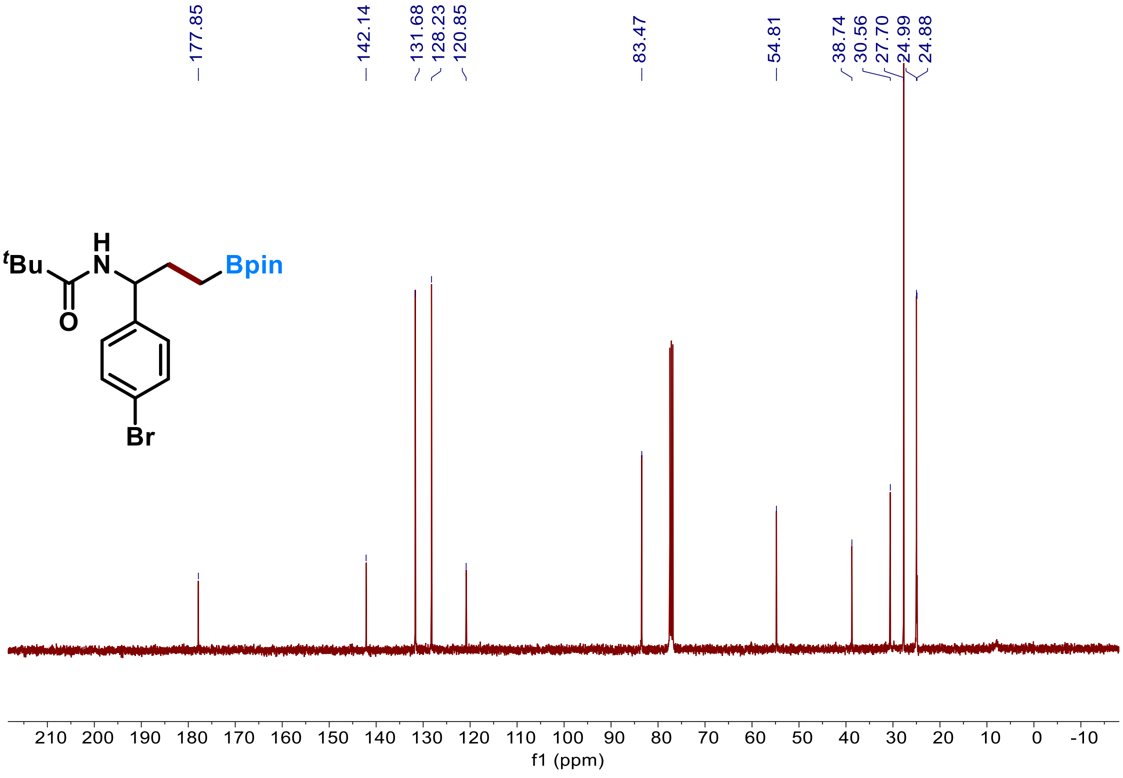

Supplementary Figure 128. ^{13}C{^{1}H} NMR spectrum of **2i** (101 MHz, Chloroform-*d*)

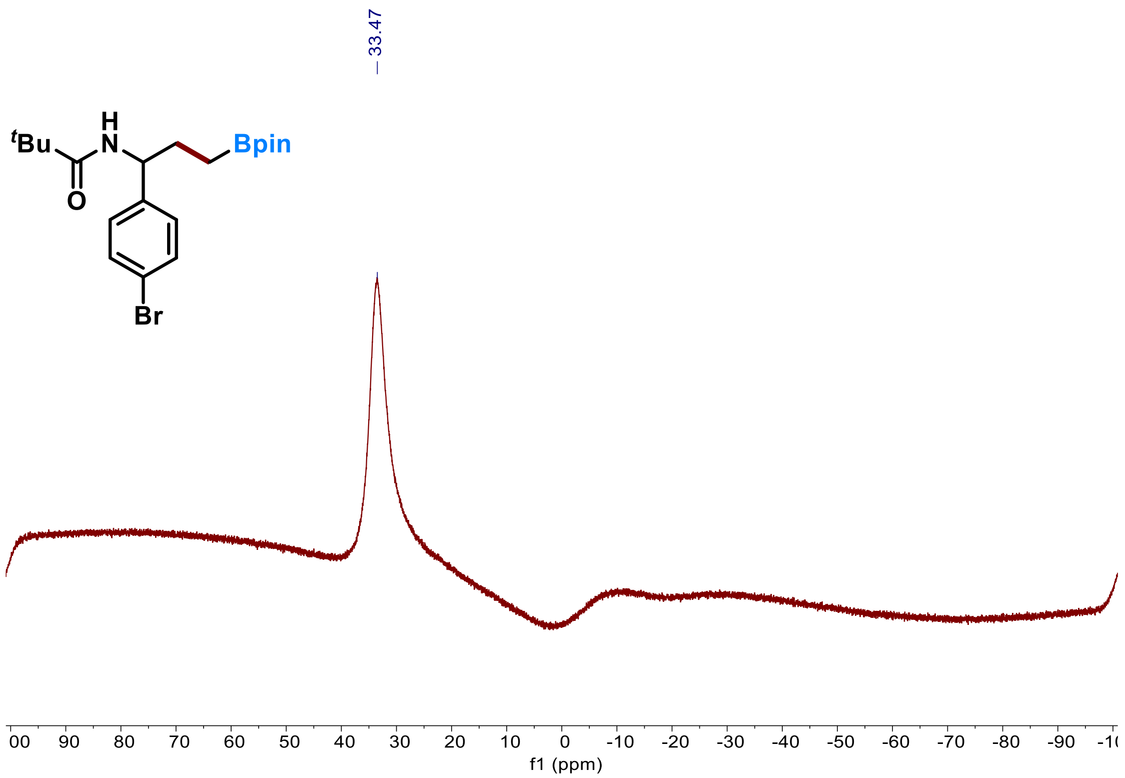

Supplementary Figure 129. ^{11}B NMR spectrum of **2i** (128 MHz, Chloroform-*d*)

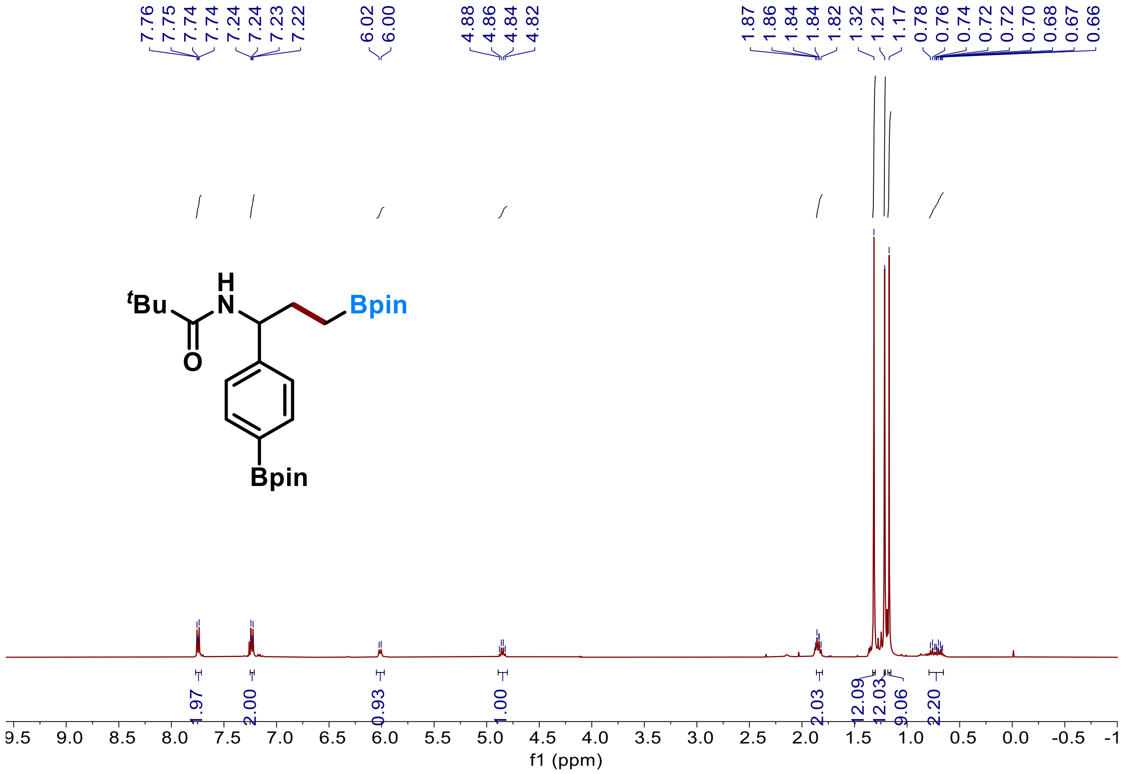

Supplementary Figure 130. ^{1}H NMR spectrum of **2j** (400 MHz, Chloroform-*d*)

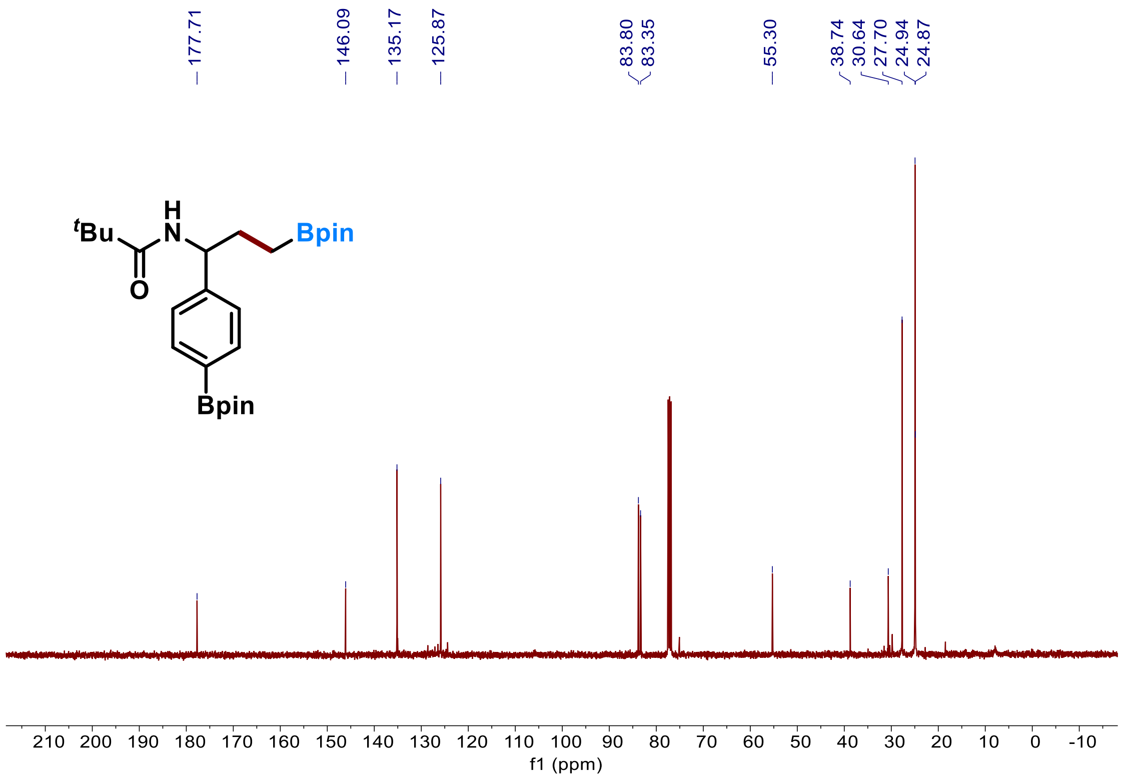

Supplementary Figure 131. $^{13}C\{^{1}H\}$ NMR spectrum of **2j** (101 MHz, Chloroform-*d*)

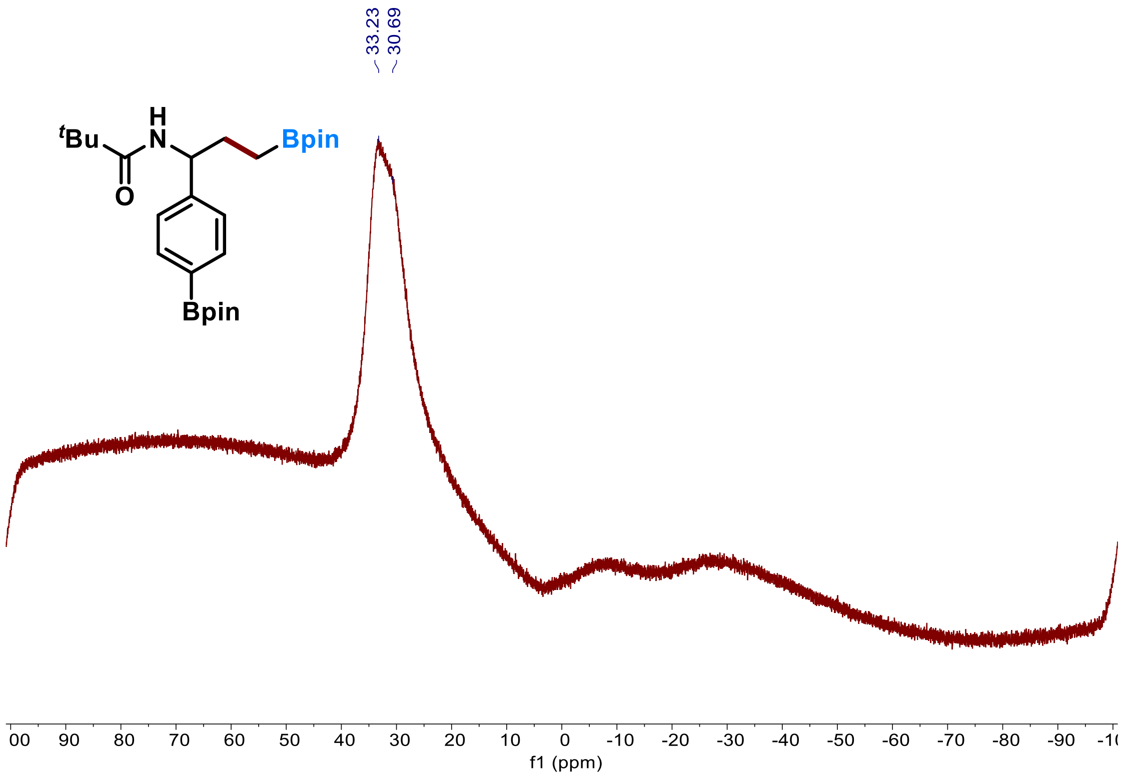

Supplementary Figure 132. ^{11}B NMR spectrum of **2j** (128 MHz, Chloroform-*d*)

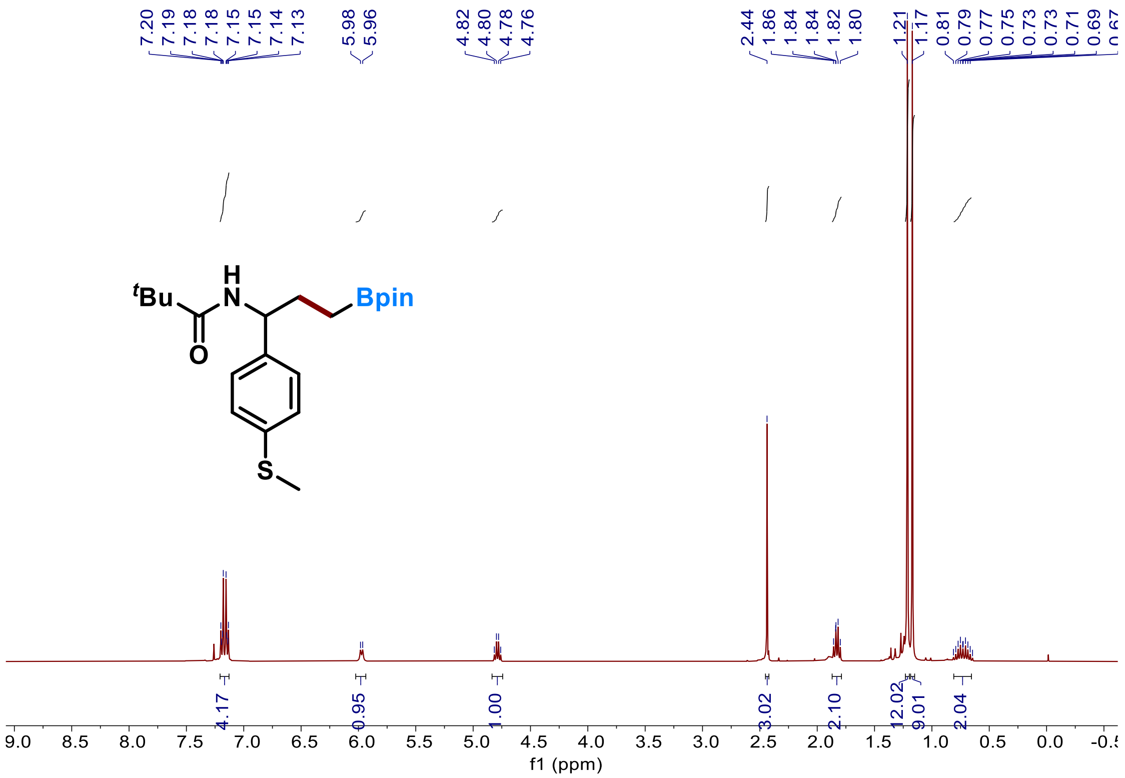

Supplementary Figure 133. ^{1}H NMR spectrum of **2k** (400 MHz, Chloroform-*d*)

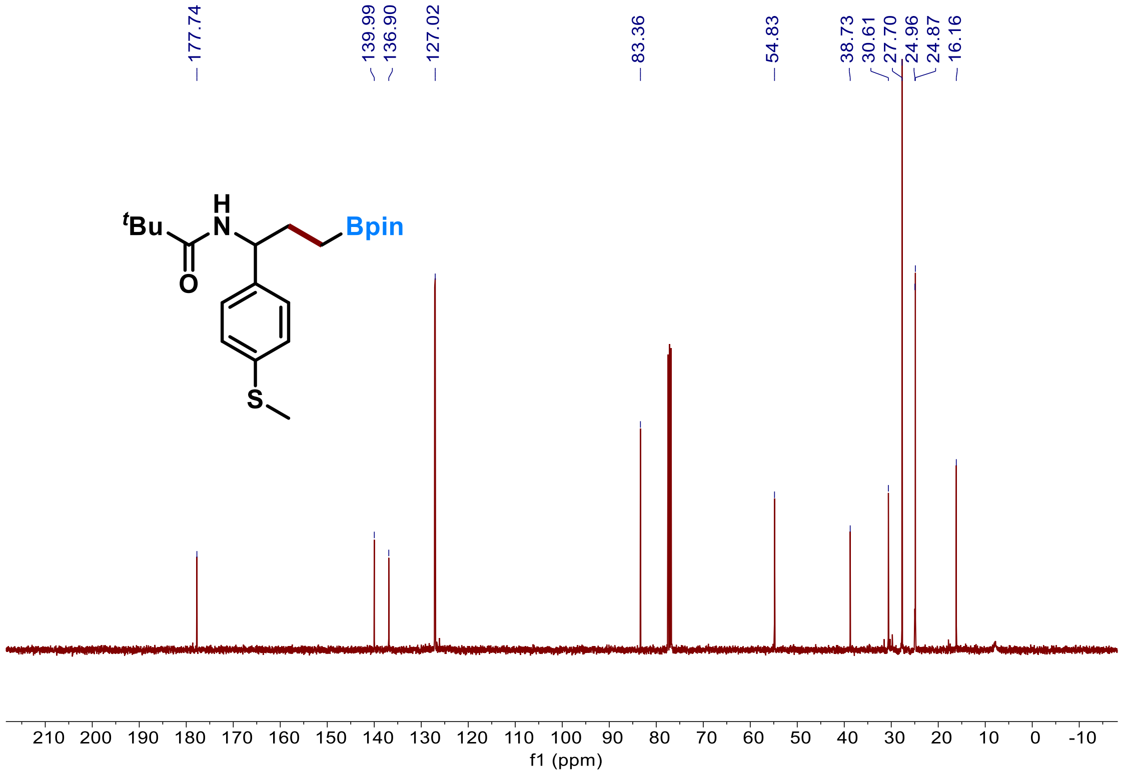

Supplementary Figure 134. $^{13}C\{^{1}H\}$ NMR spectrum of **2k** (101 MHz, Chloroform-*d*)

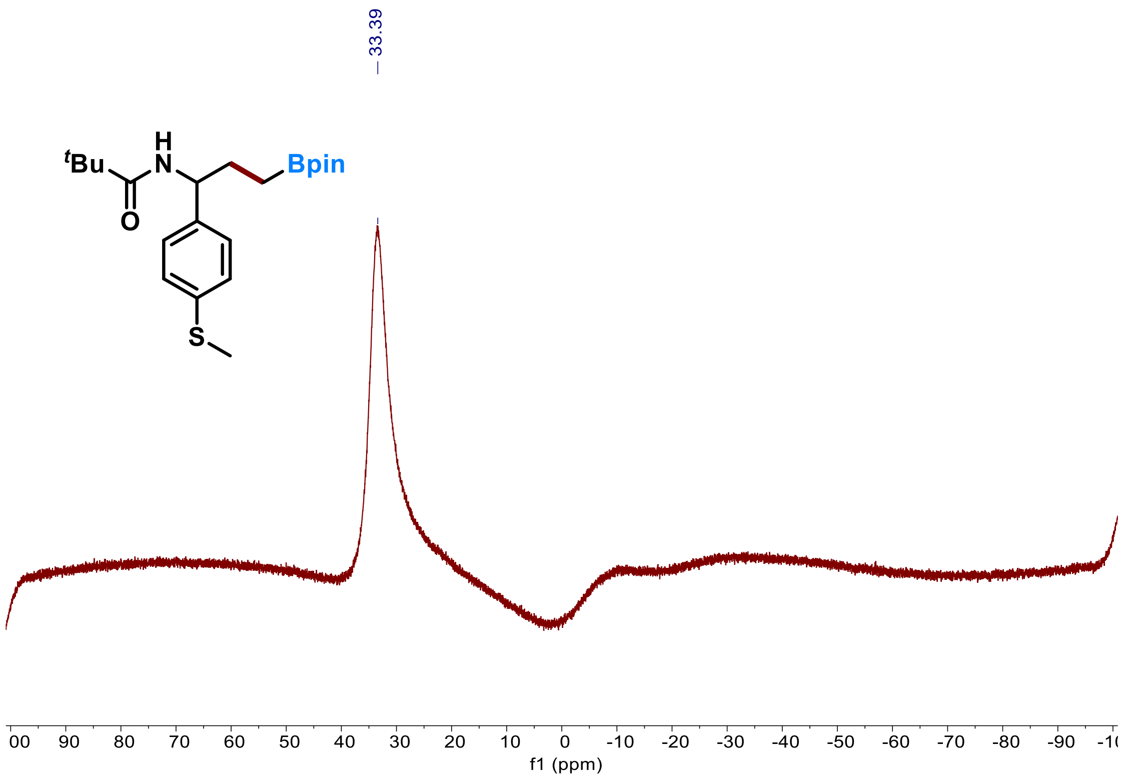

Supplementary Figure 135. ^{11}B NMR spectrum of **2k** (128 MHz, Chloroform-*d*)

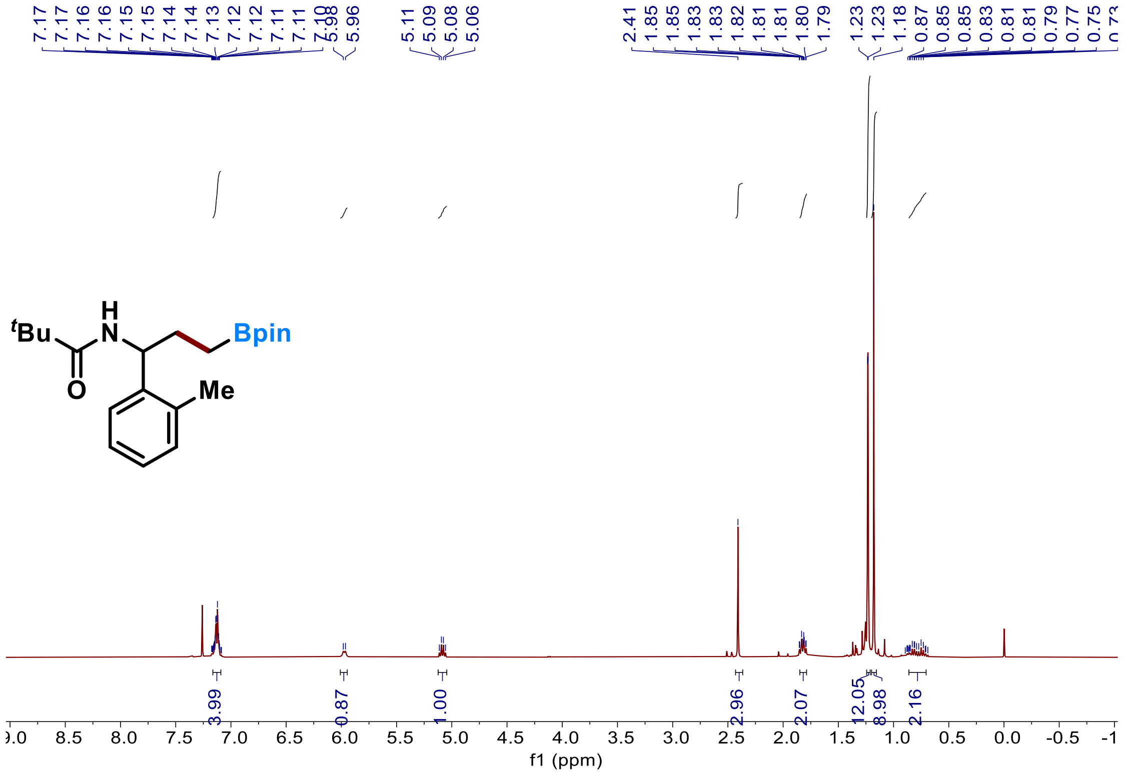

Supplementary Figure 136. ^{1}H NMR spectrum of **2l** (400 MHz, Chloroform-*d*)

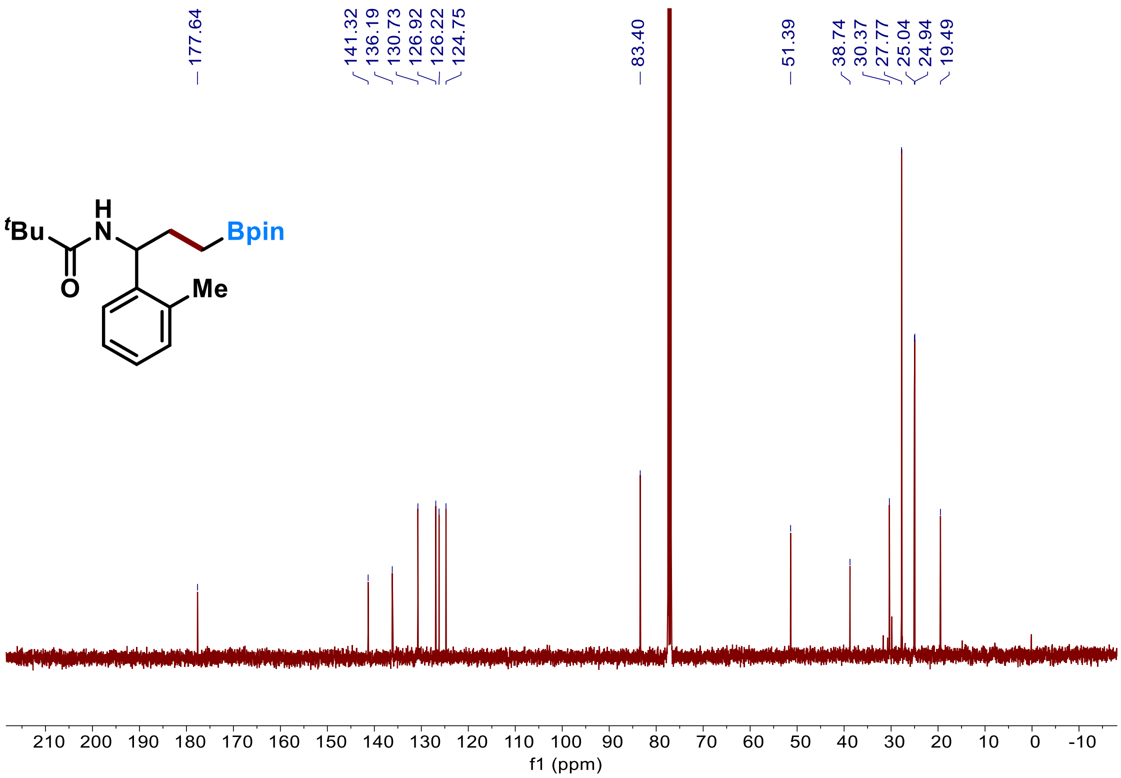

Supplementary Figure 137. $^{13}C\{^{1}H\}$ NMR spectrum of **2l** (101 MHz, Chloroform-*d*)

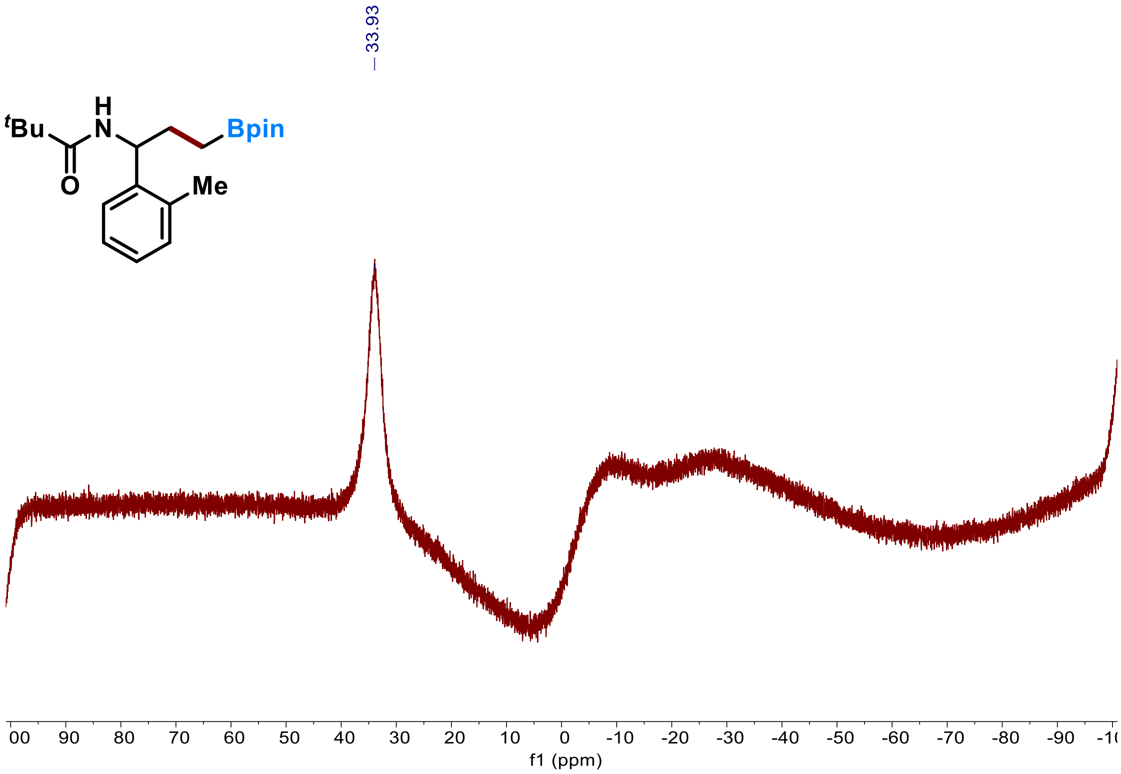

Supplementary Figure 138. ^{11}B NMR spectrum of **2l** (128 MHz, Chloroform-*d*)

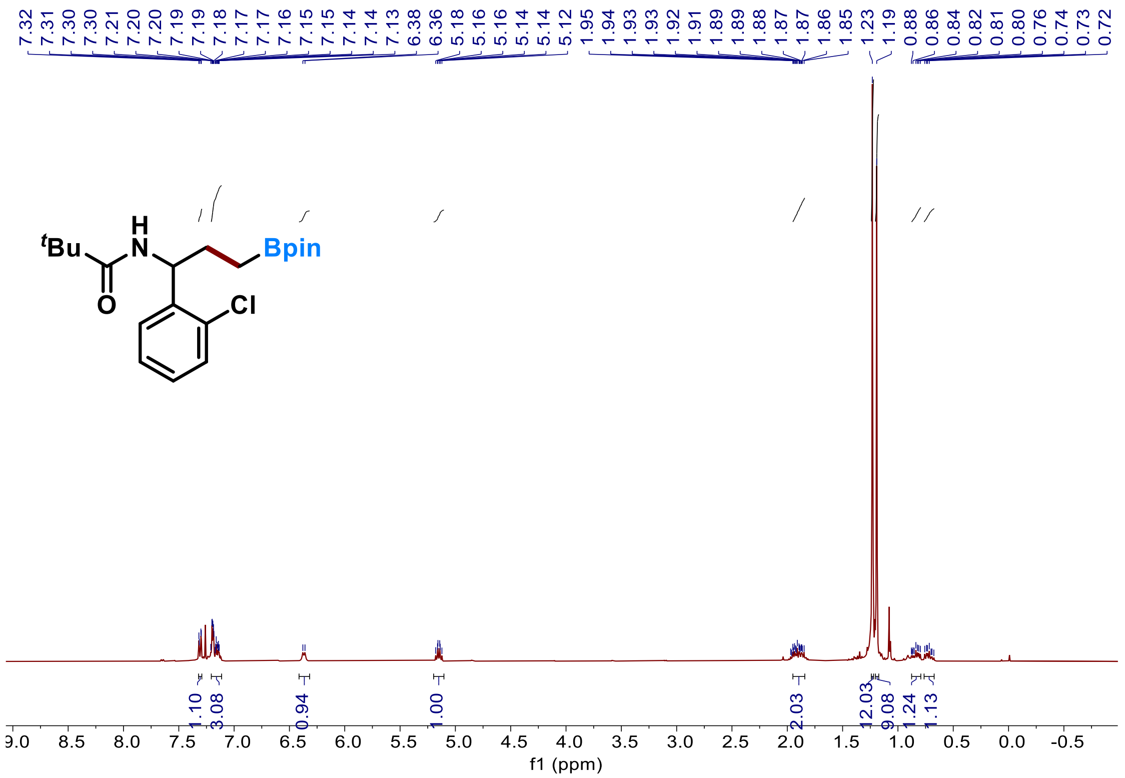

Supplementary Figure 139. ^{1}H NMR spectrum of **2m** (400 MHz, Chloroform-*d*)

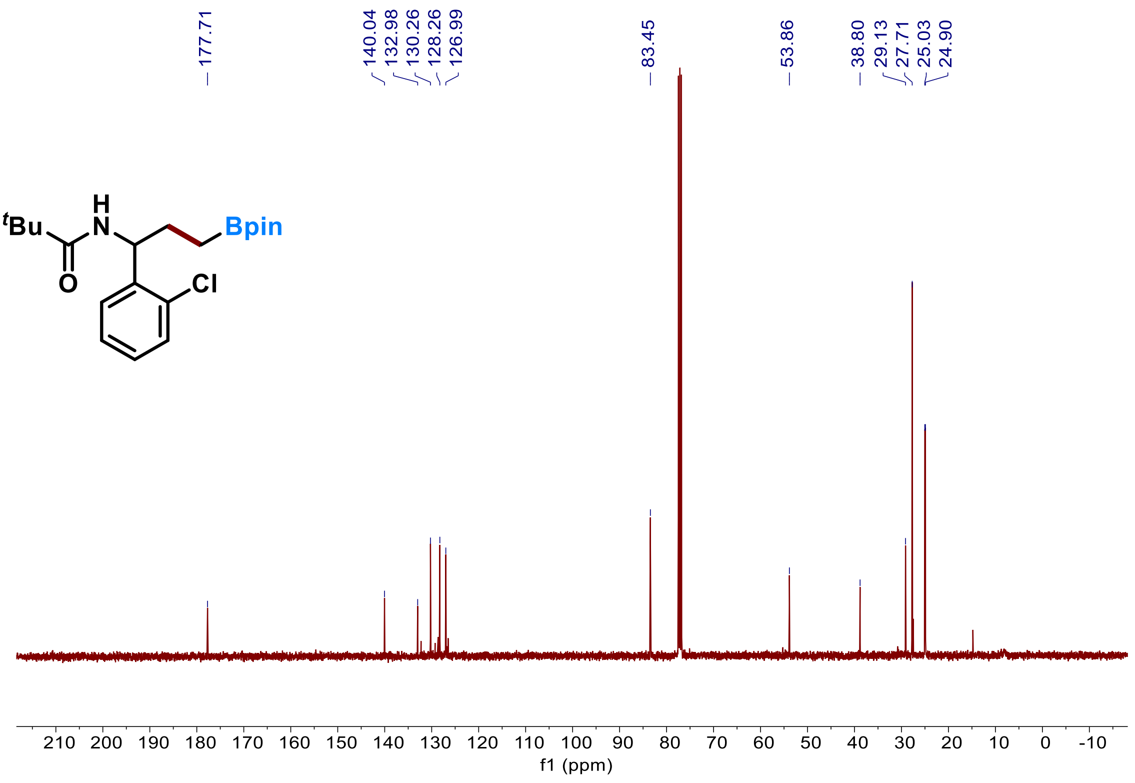

Supplementary Figure 140. ^{13}C{^{1}H} NMR spectrum of **2m** (101 MHz, Chloroform-*d*)

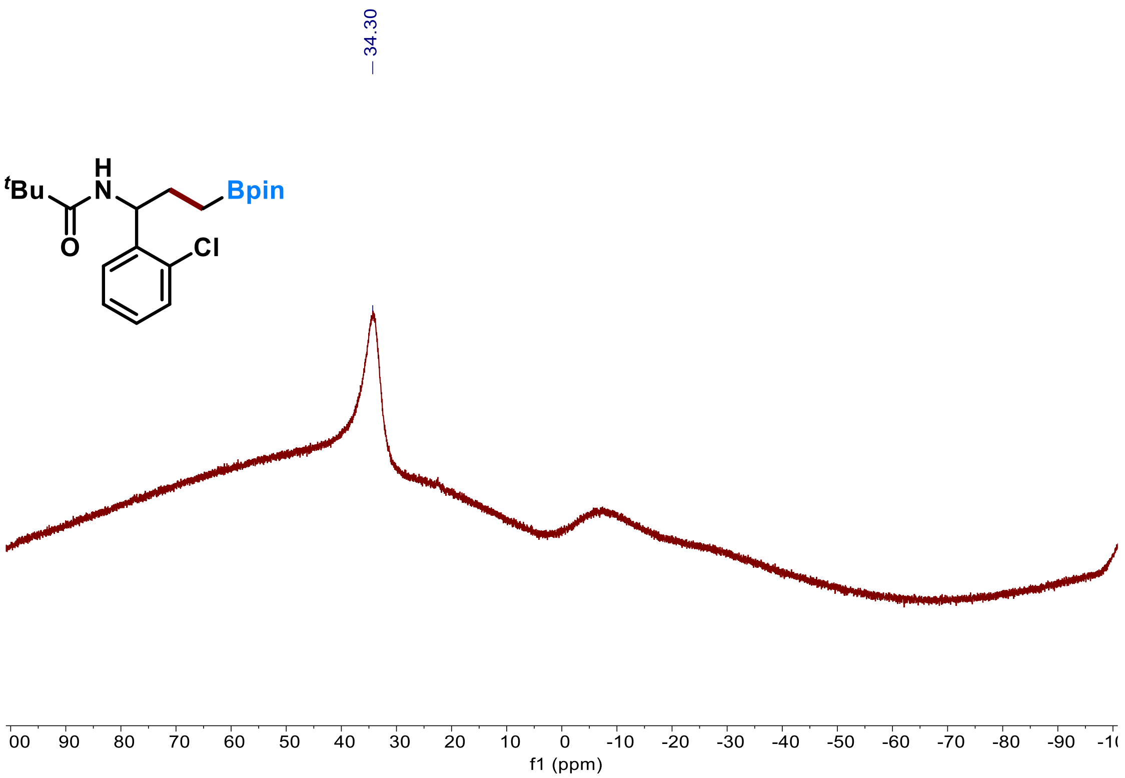

Supplementary Figure 141. ^{11}B NMR spectrum of **2m** (128 MHz, Chloroform-*d*)

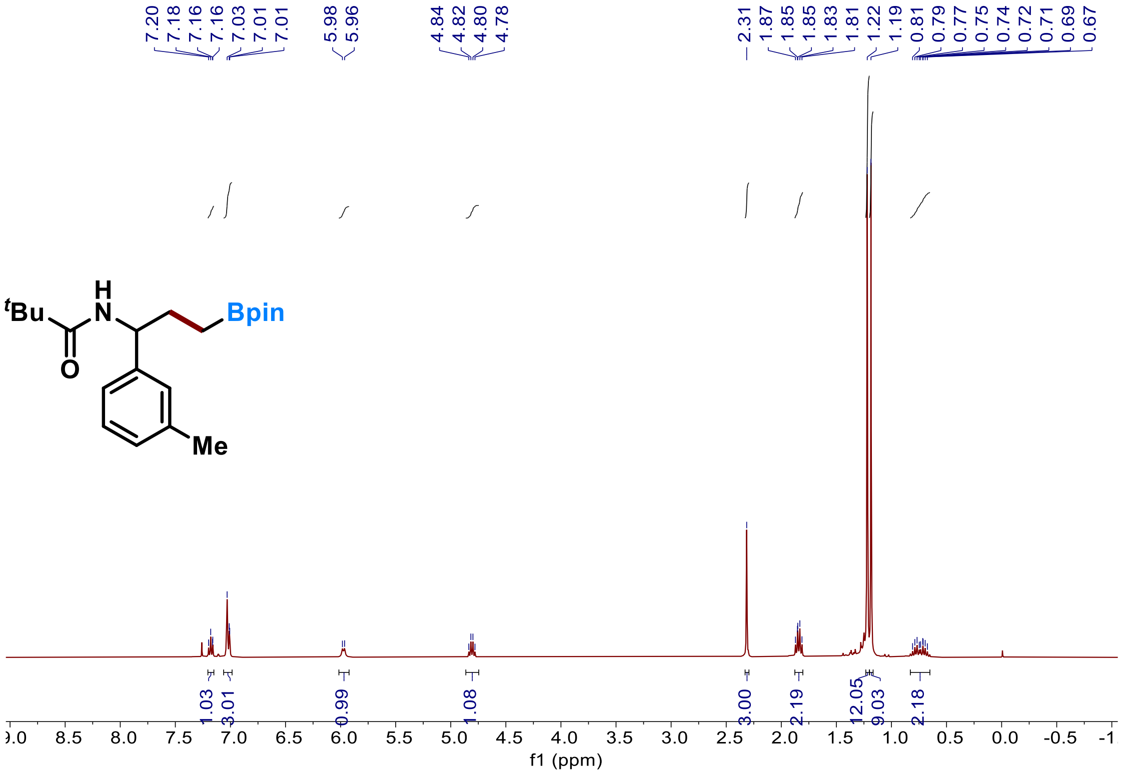

Supplementary Figure 142. ^{1}H NMR spectrum of **2n** (400 MHz, Chloroform-*d*)

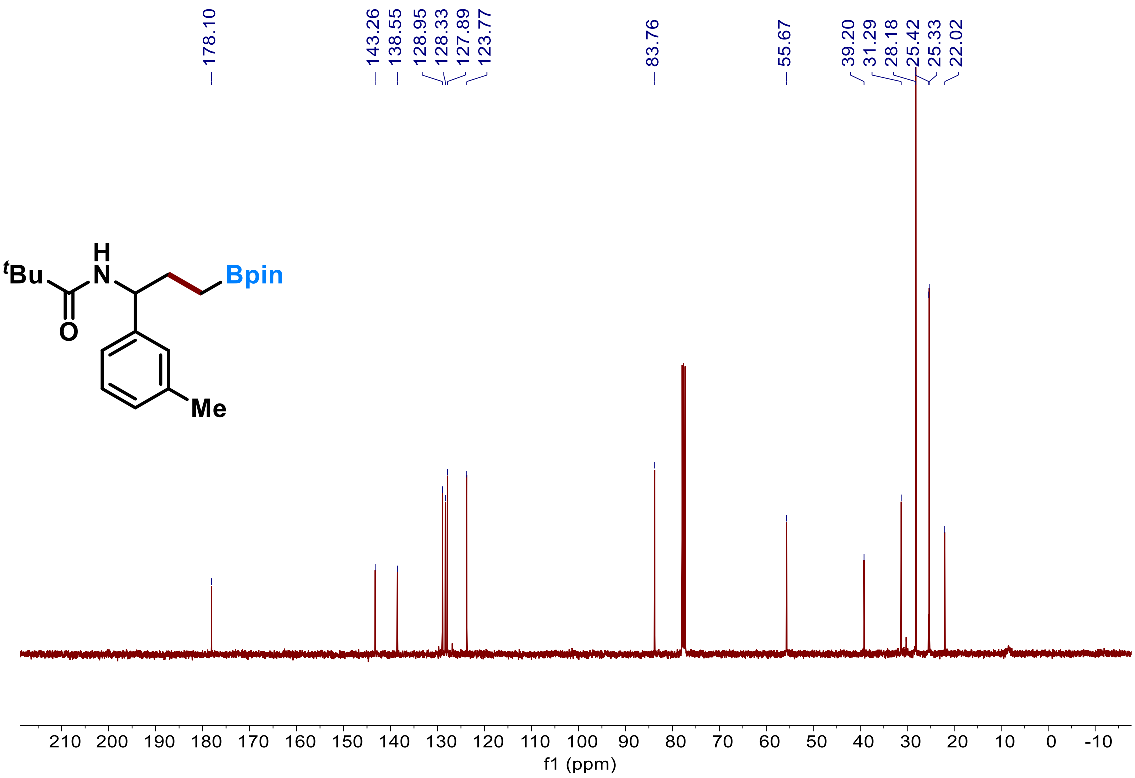

Supplementary Figure 143. $^{13}C\{^{1}H\}$ NMR spectrum of **2n** (101 MHz, Chloroform-*d*)

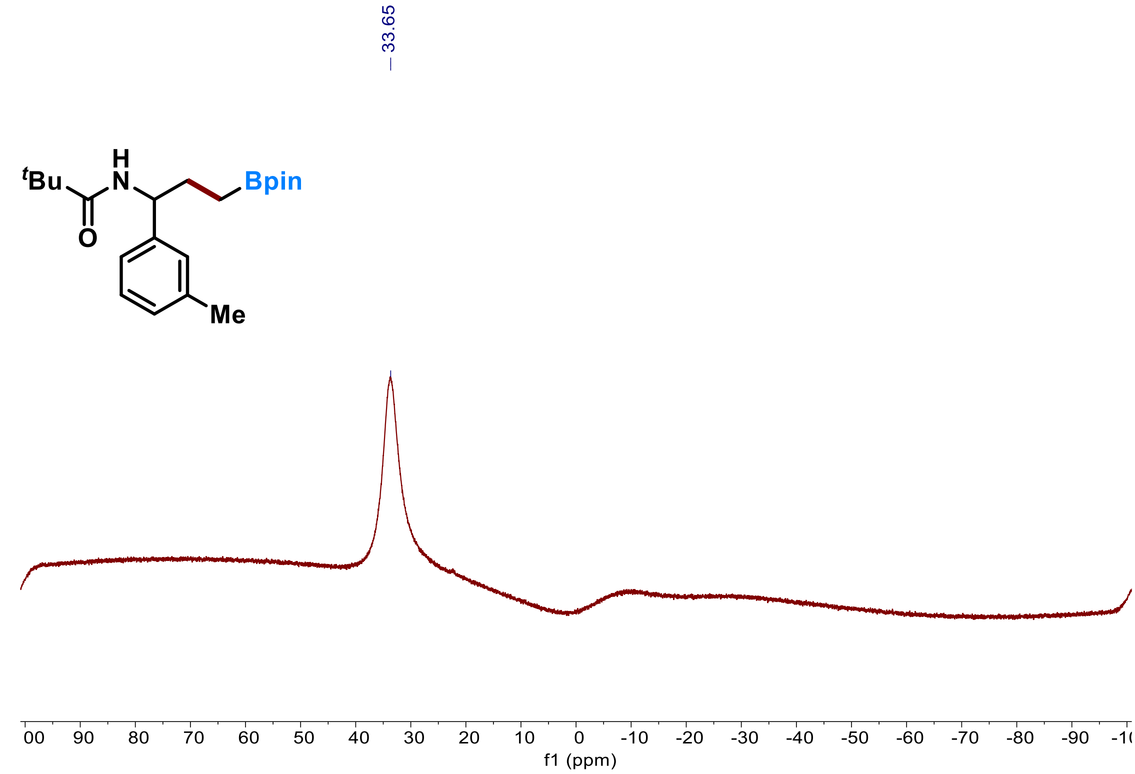

Supplementary Figure 144. ^{11}B NMR spectrum of **2n** (128 MHz, Chloroform-*d*)

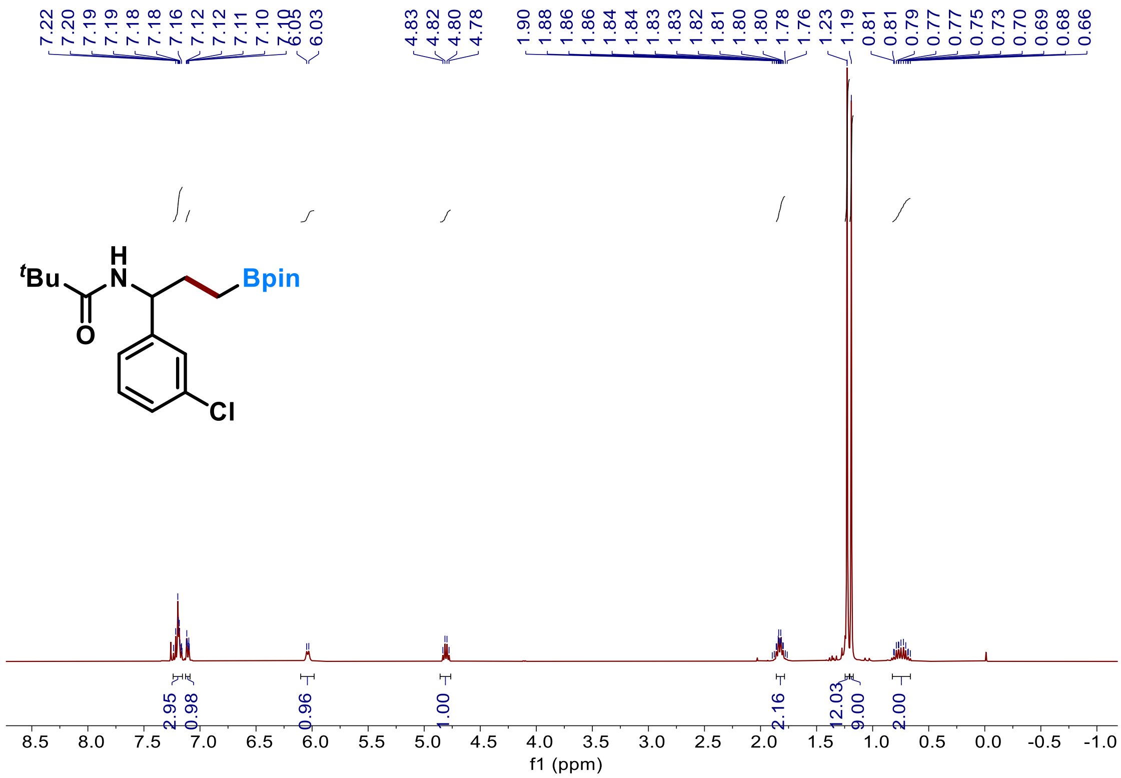

Supplementary Figure 145. ^{1}H NMR spectrum of **2o** (400 MHz, Chloroform-*d*)

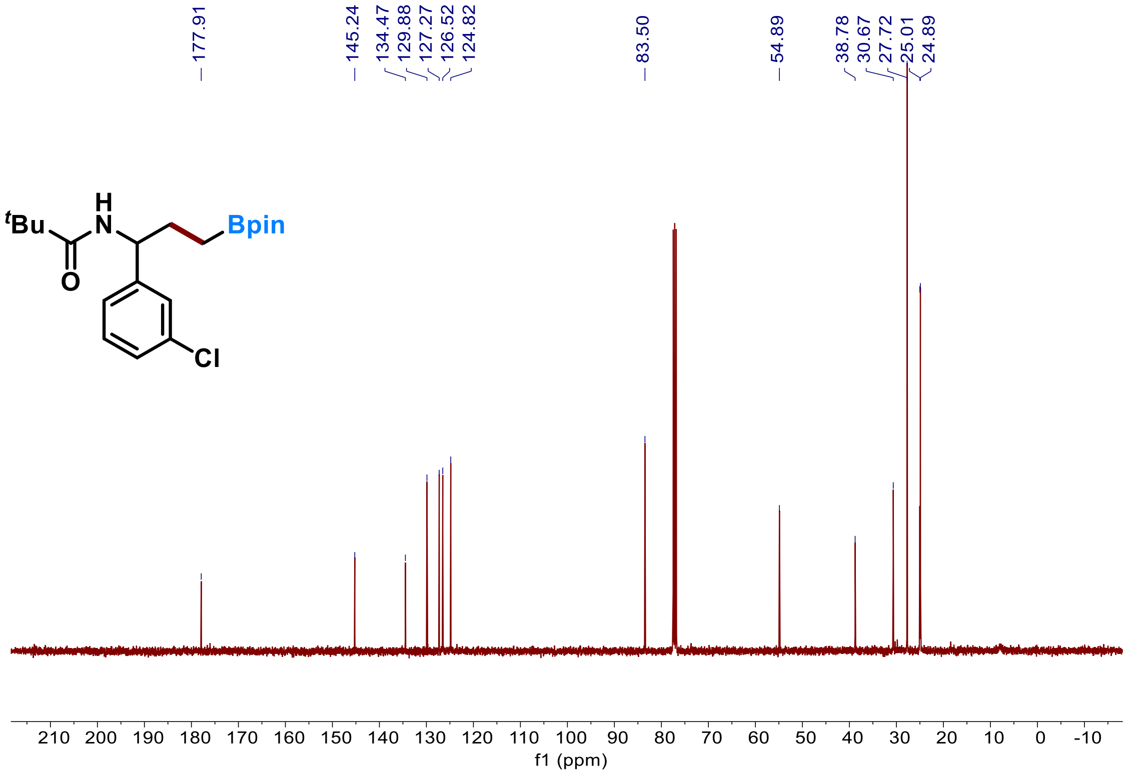

Supplementary Figure 146. ^{13}C{^{1}H} NMR spectrum of **2o** (101 MHz, Chloroform-*d*)

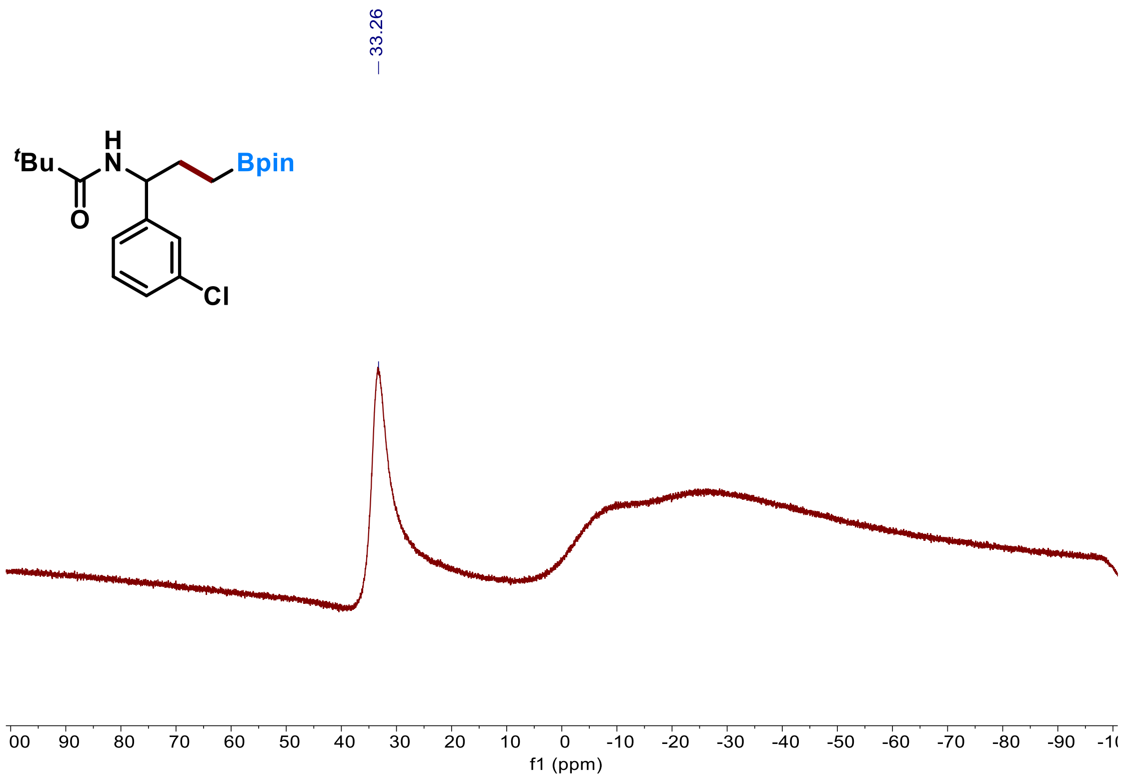

Supplementary Figure 147. ^{11}B NMR spectrum of **2o** (128 MHz, Chloroform-*d*)

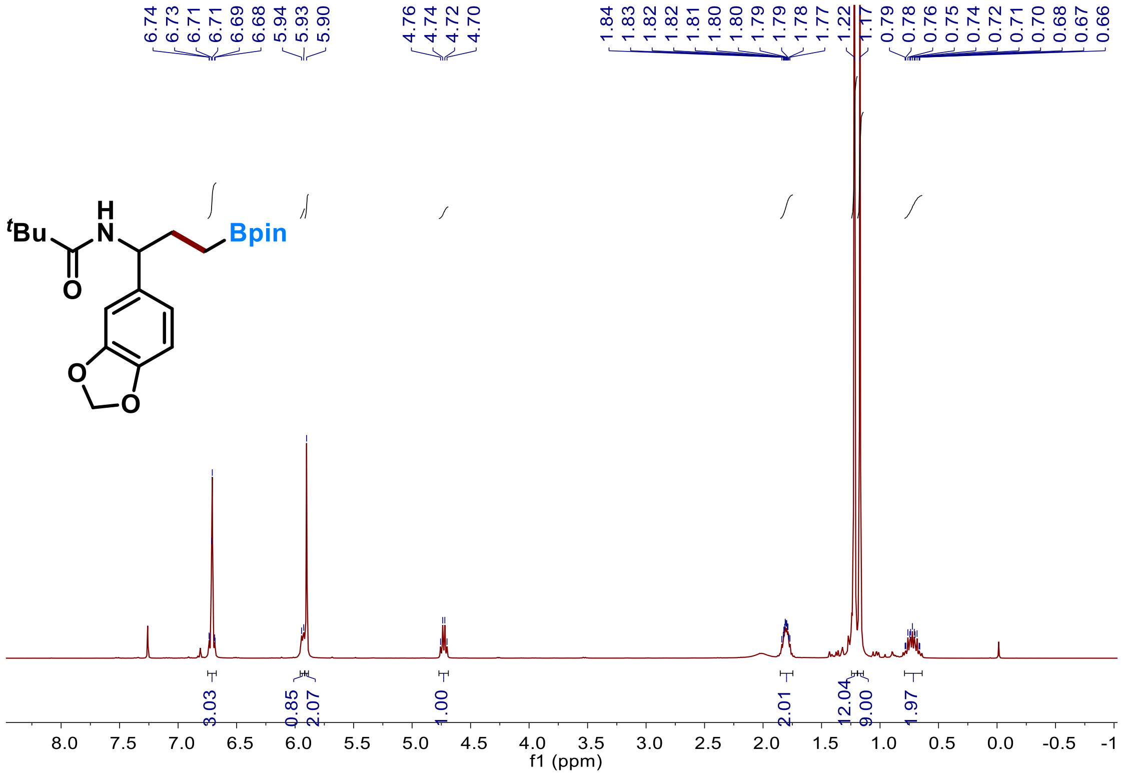

Supplementary Figure 148. ^{1}H NMR spectrum of **2p** (400 MHz, Chloroform-*d*)

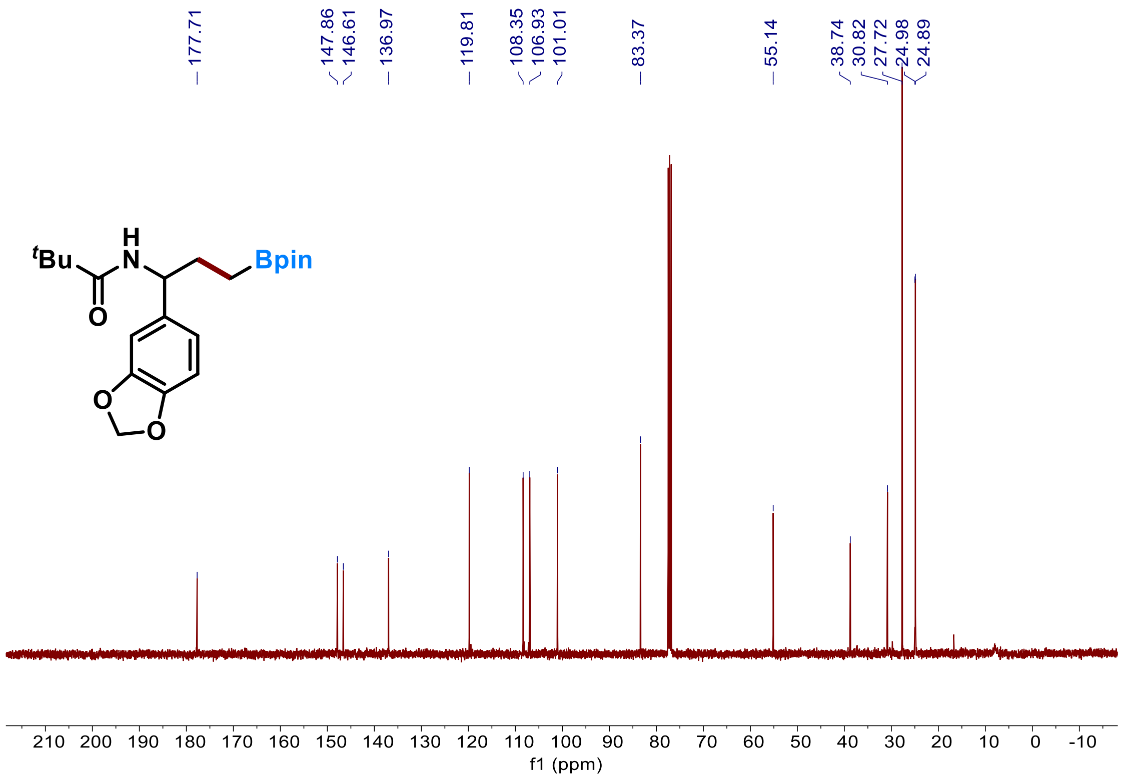

Supplementary Figure 149. $^{13}C\{^{1}H\}$ NMR spectrum of **2p** (101 MHz, Chloroform-*d*)

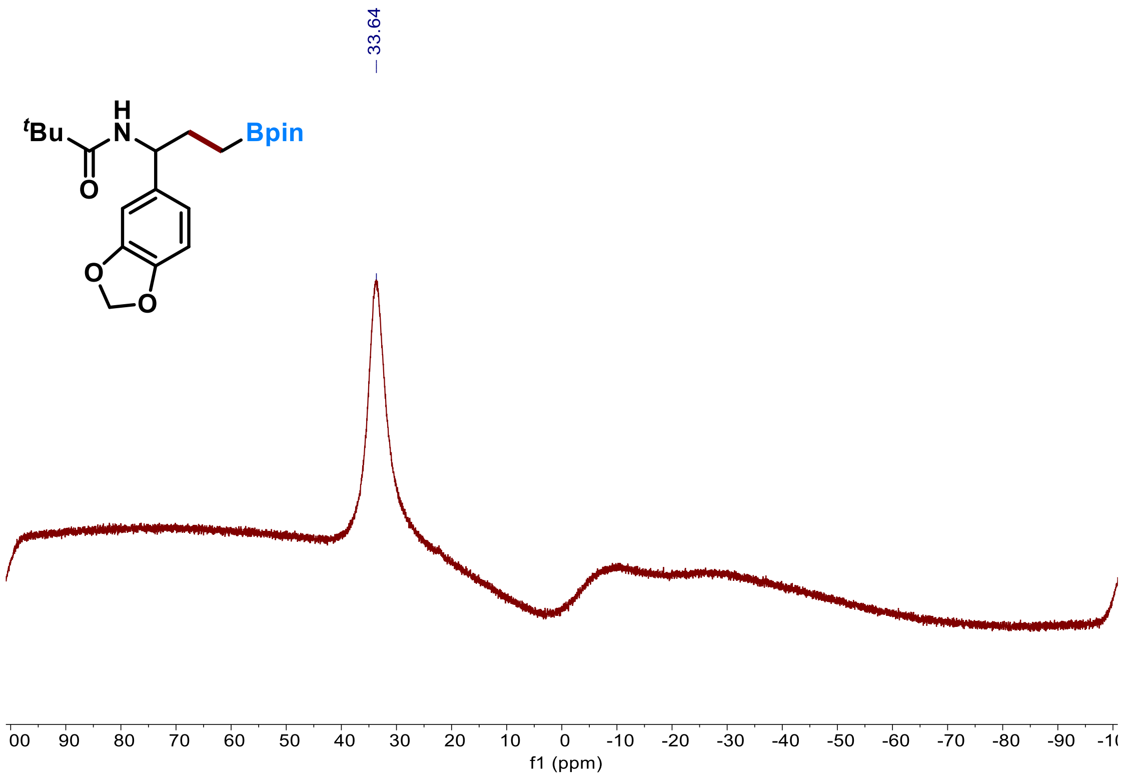

Supplementary Figure 150. ^{11}B NMR spectrum of **2p** (128 MHz, Chloroform-*d*)

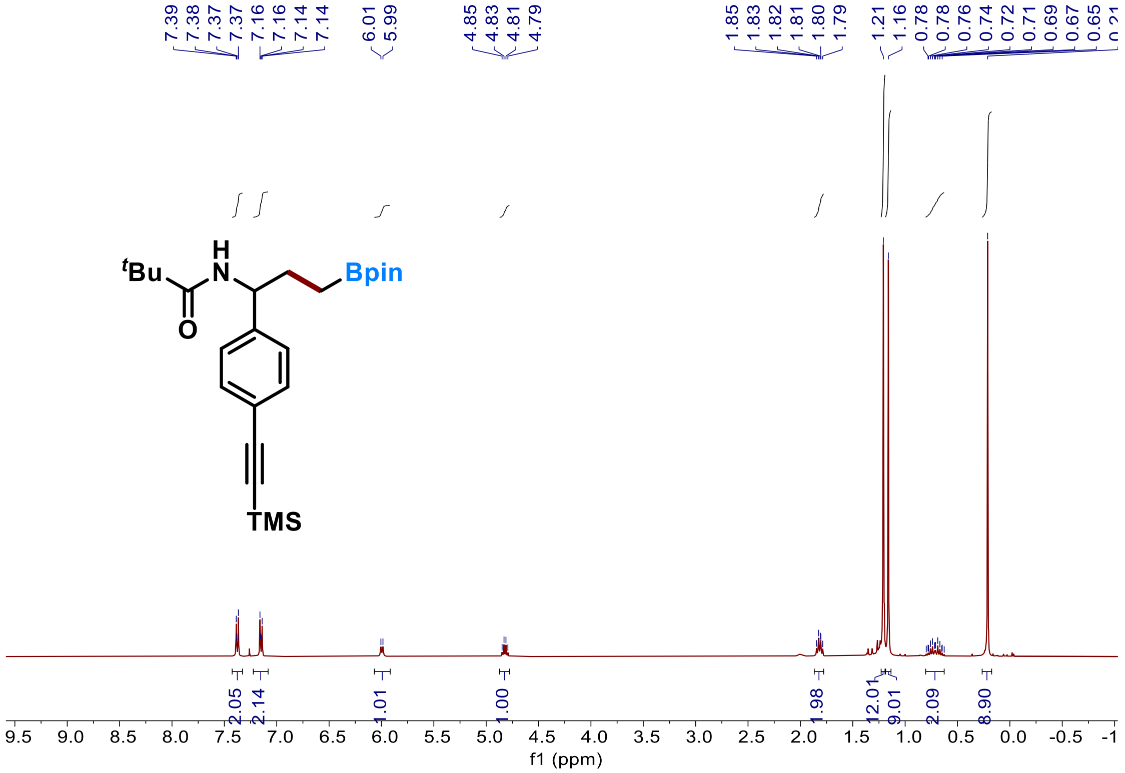

Supplementary Figure 151. ^{1}H NMR spectrum of **2q** (400 MHz, Chloroform-*d*)

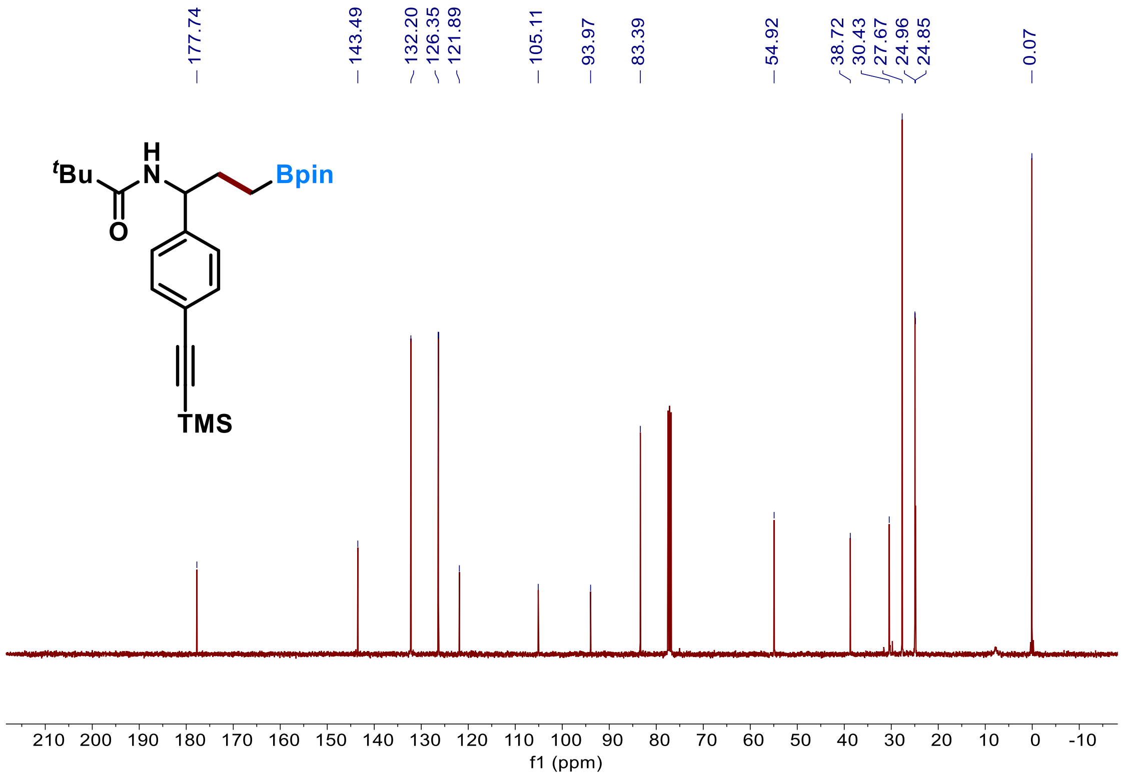

Supplementary Figure 152. ^{13}C{^{1}H} NMR spectrum of **2q** (101 MHz, Chloroform-*d*)

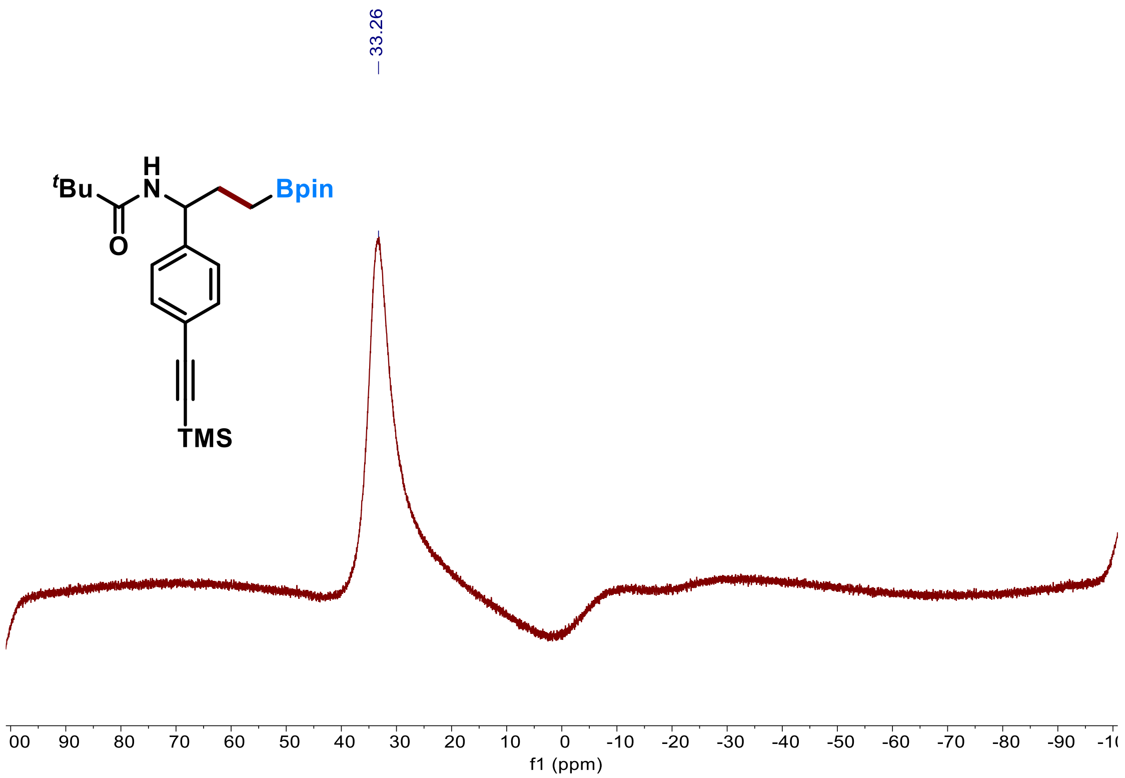

Supplementary Figure 153. ^{11}B NMR spectrum of **2q** (128 MHz, Chloroform-*d*)

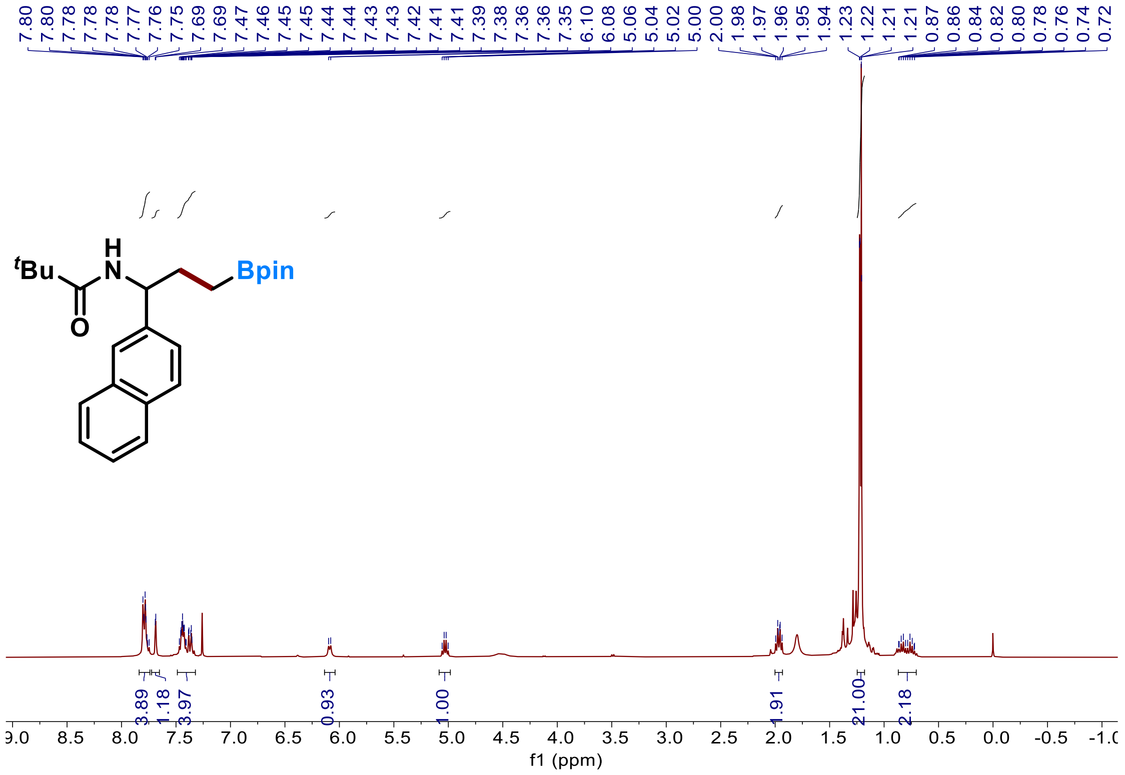

Supplementary Figure 154. ^{1}H NMR spectrum of **2r** (400 MHz, Chloroform-*d*)

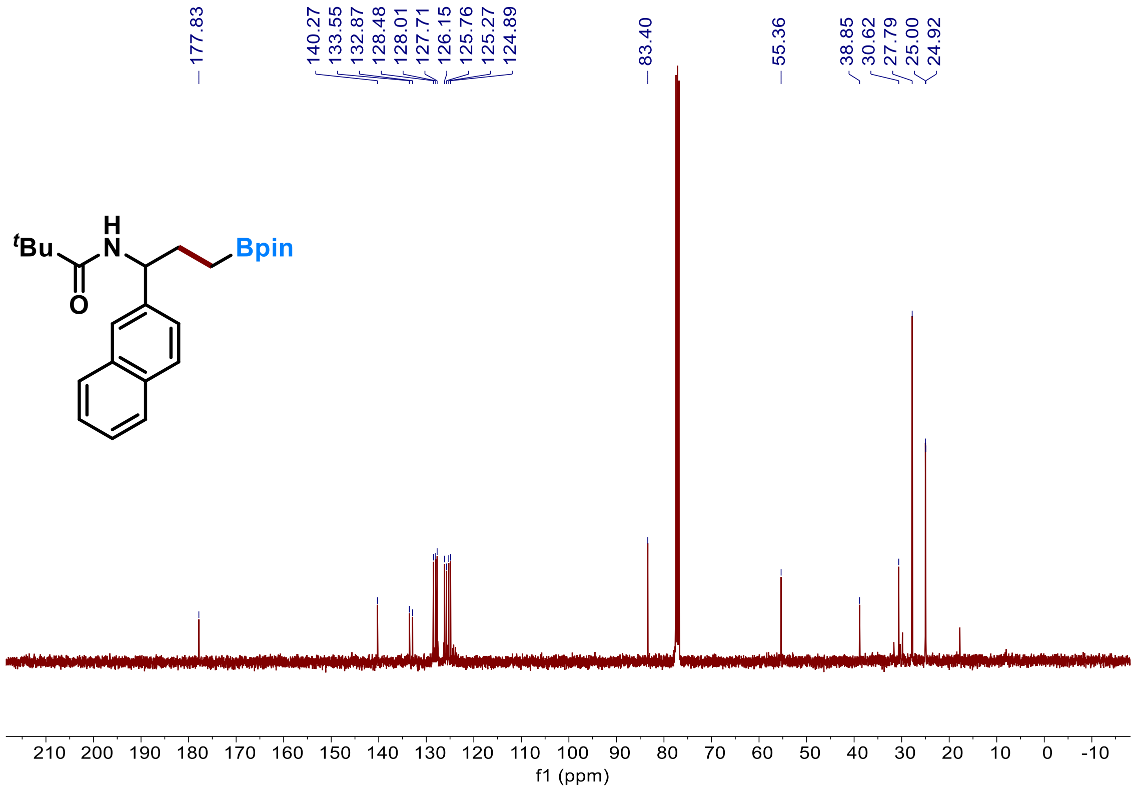

Supplementary Figure 155. $^{13}C\{^{1}H\}$ NMR spectrum of **2r** (101 MHz, Chloroform-*d*)

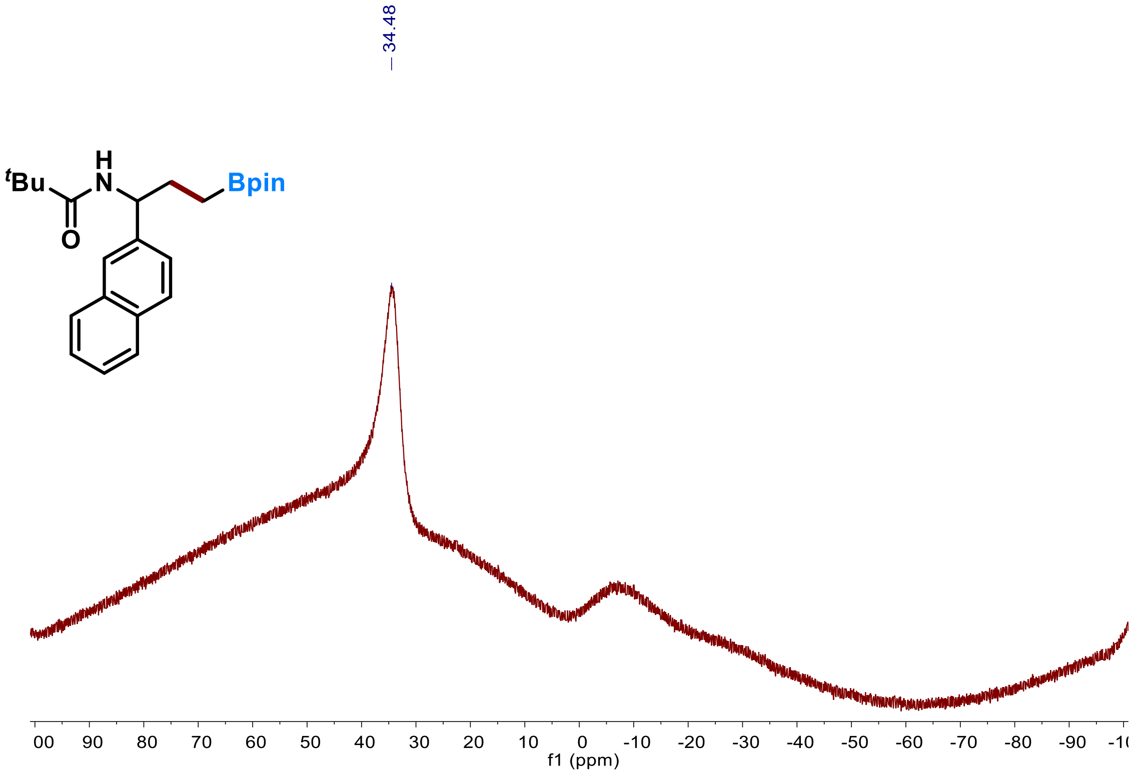

Supplementary Figure 156. ^{11}B NMR spectrum of **2r** (128 MHz, Chloroform-*d*)

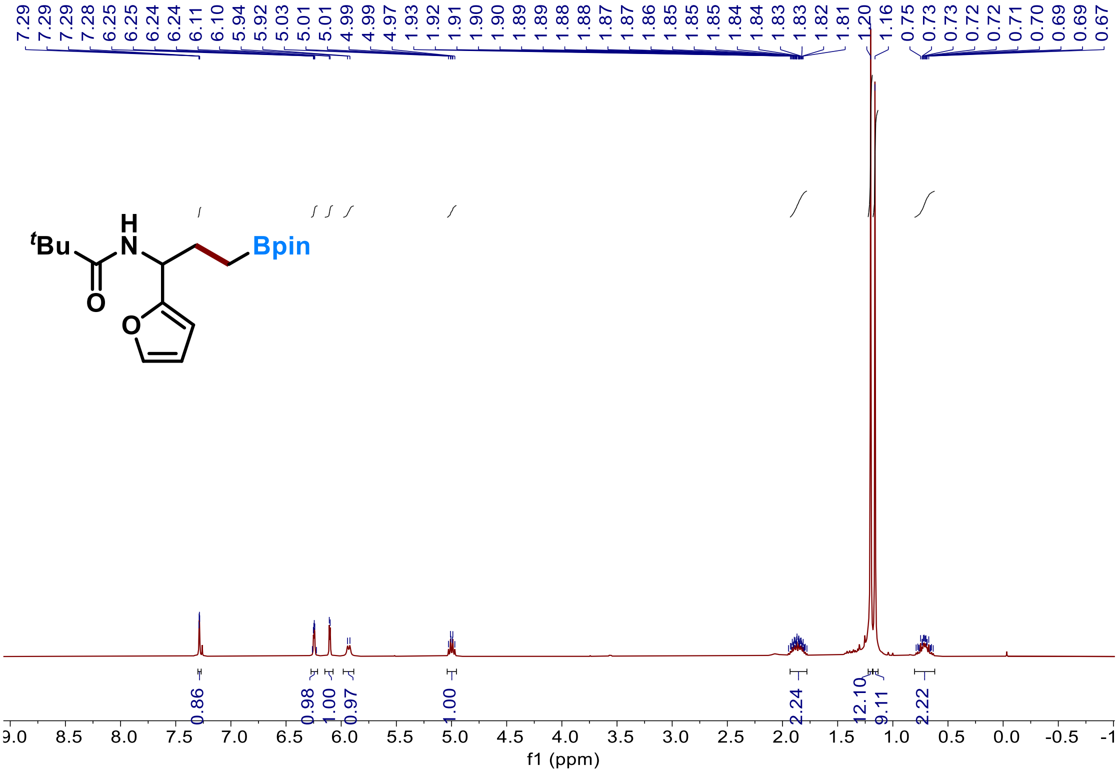

Supplementary Figure 157. ^{1}H NMR spectrum of **2s** (400 MHz, Chloroform-*d*)

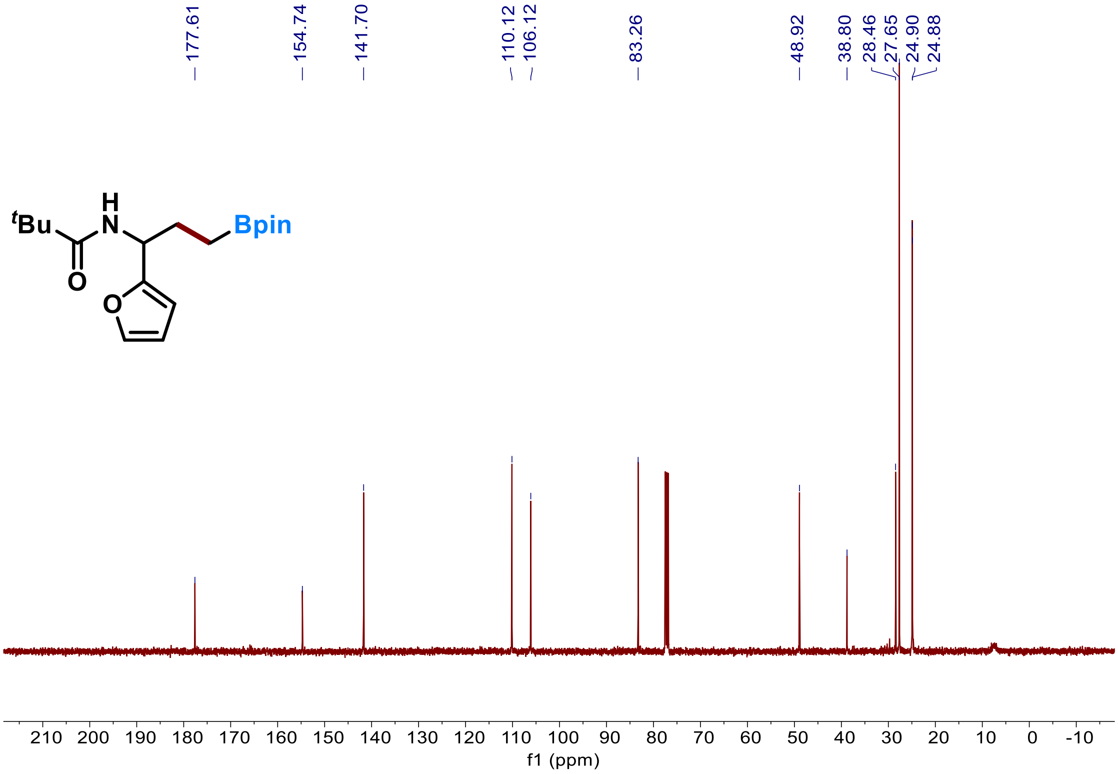

Supplementary Figure 158. $^{13}C\{^{1}H\}$ NMR spectrum of **2s** (101 MHz, Chloroform-*d*)

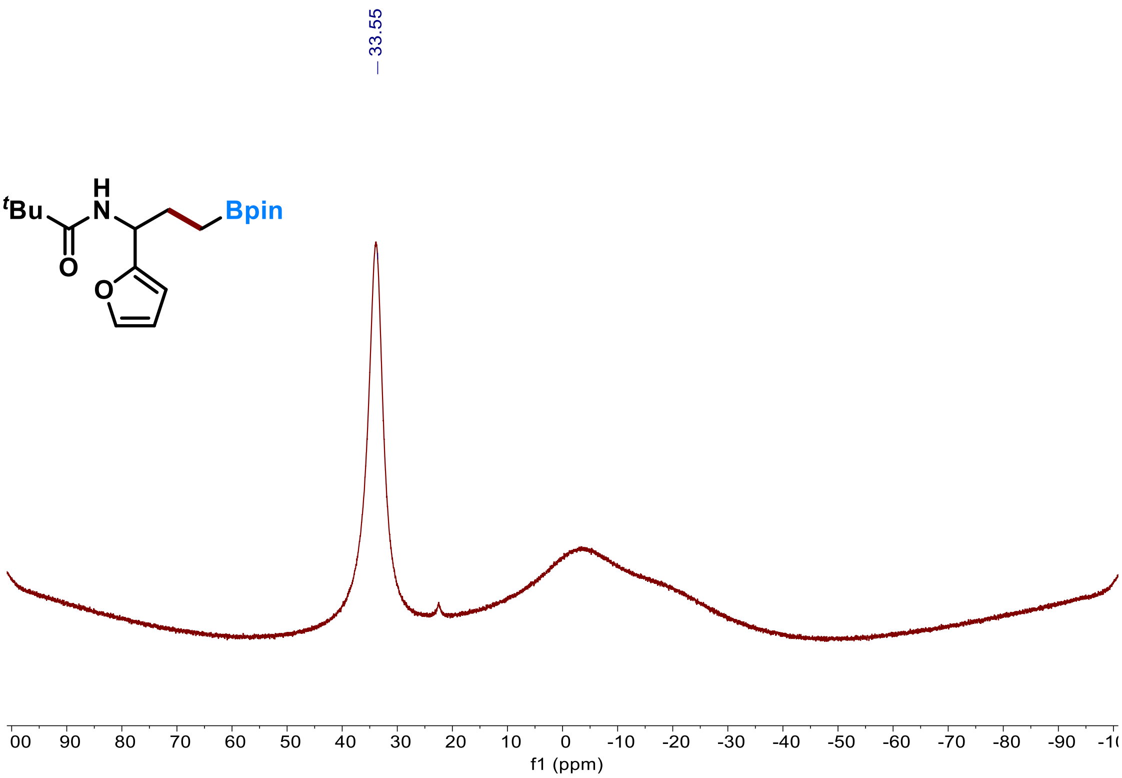

Supplementary Figure 159. ^{11}B NMR spectrum of **2s** (128 MHz, Chloroform-*d*)

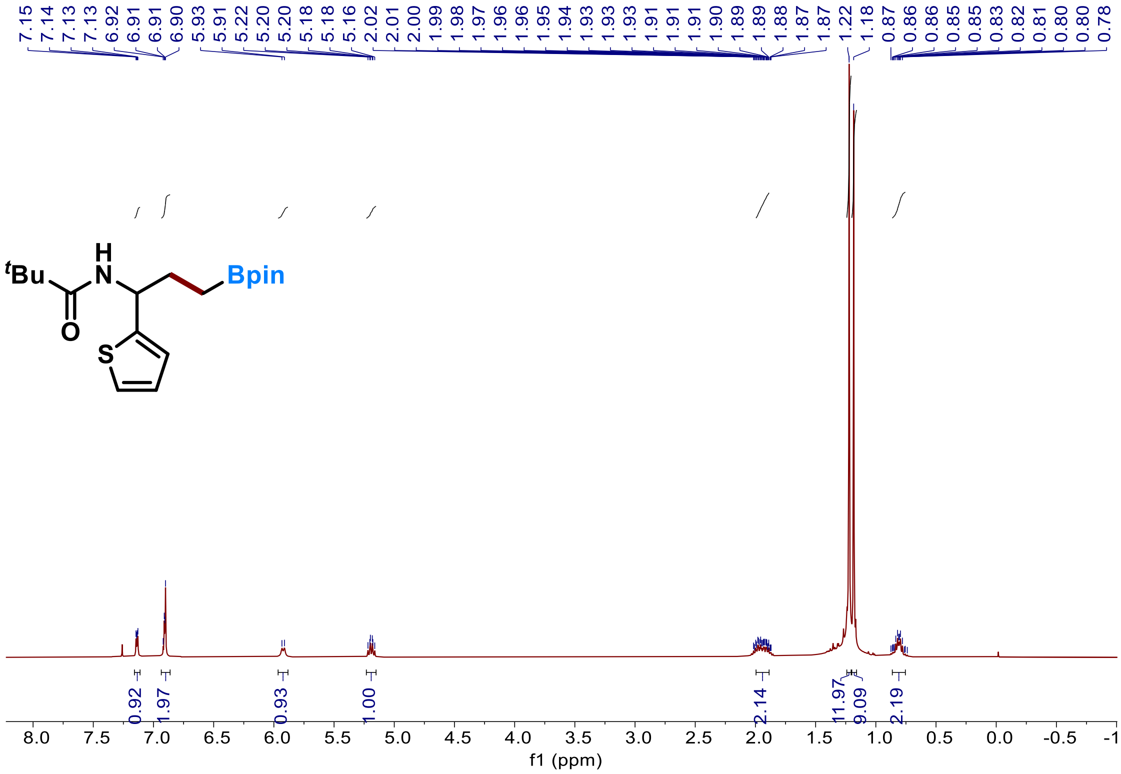

Supplementary Figure 160. ^{1}H NMR spectrum of **2t** (400 MHz, Chloroform-*d*)

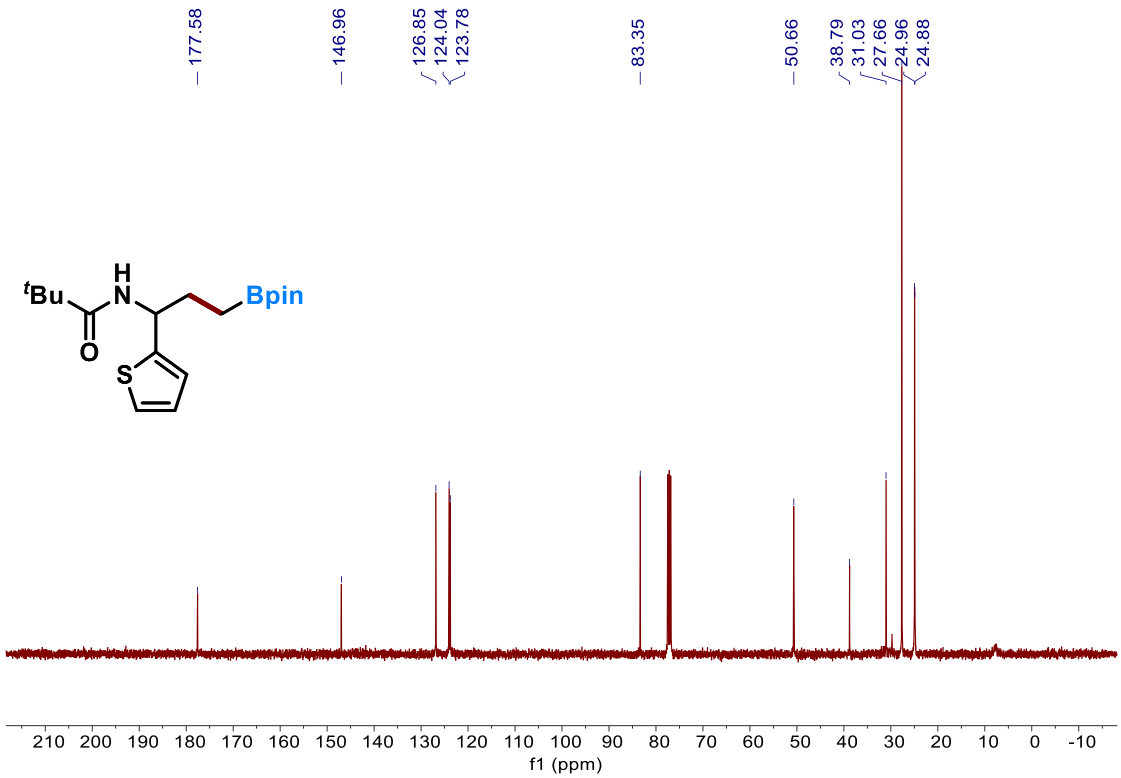

Supplementary Figure 161. $^{13}C\{^{1}H\}$ NMR spectrum of **2t** (101 MHz, Chloroform-*d*)

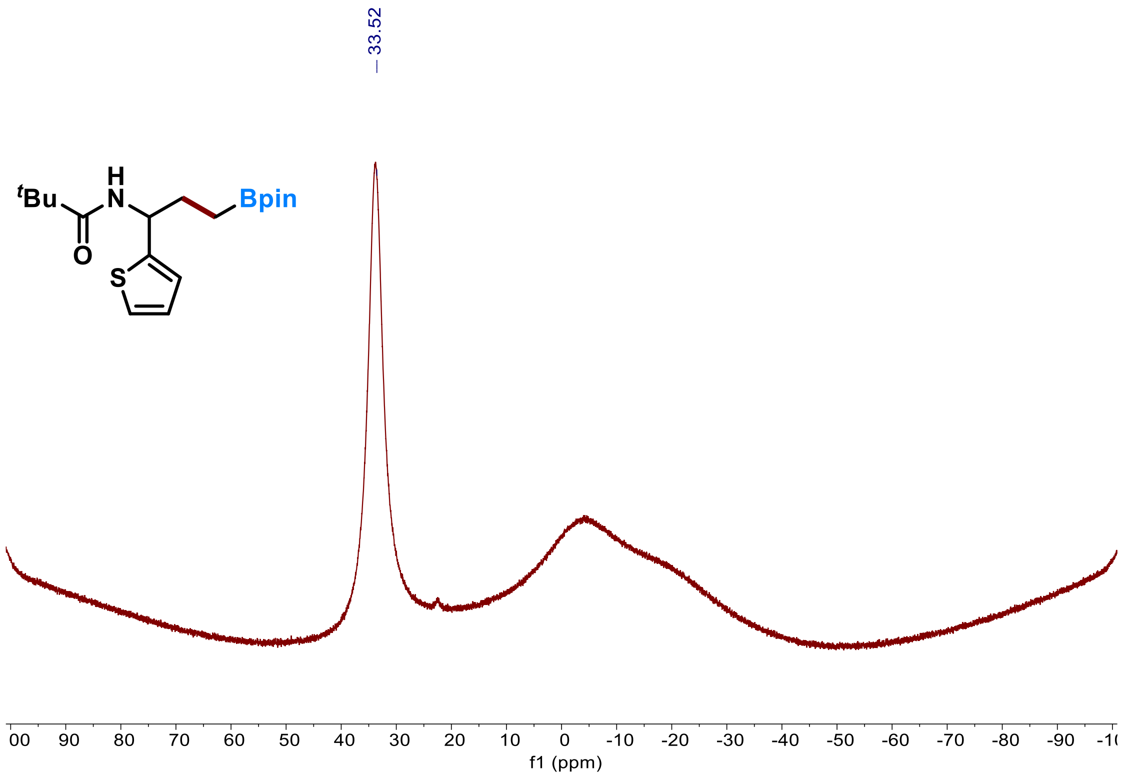

Supplementary Figure 162. ^{11}B NMR spectrum of **2t** (128 MHz, Chloroform-*d*)

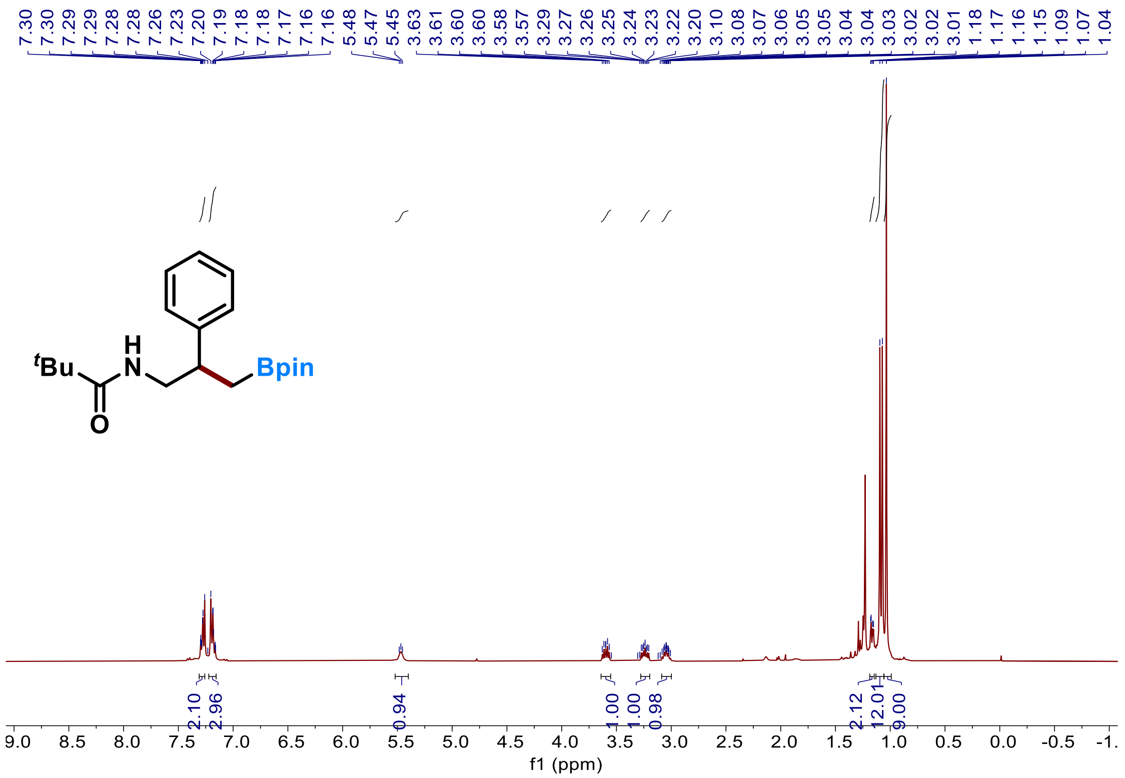

Supplementary Figure 163. ^{1}H NMR spectrum of **2u** (400 MHz, Chloroform-*d*)

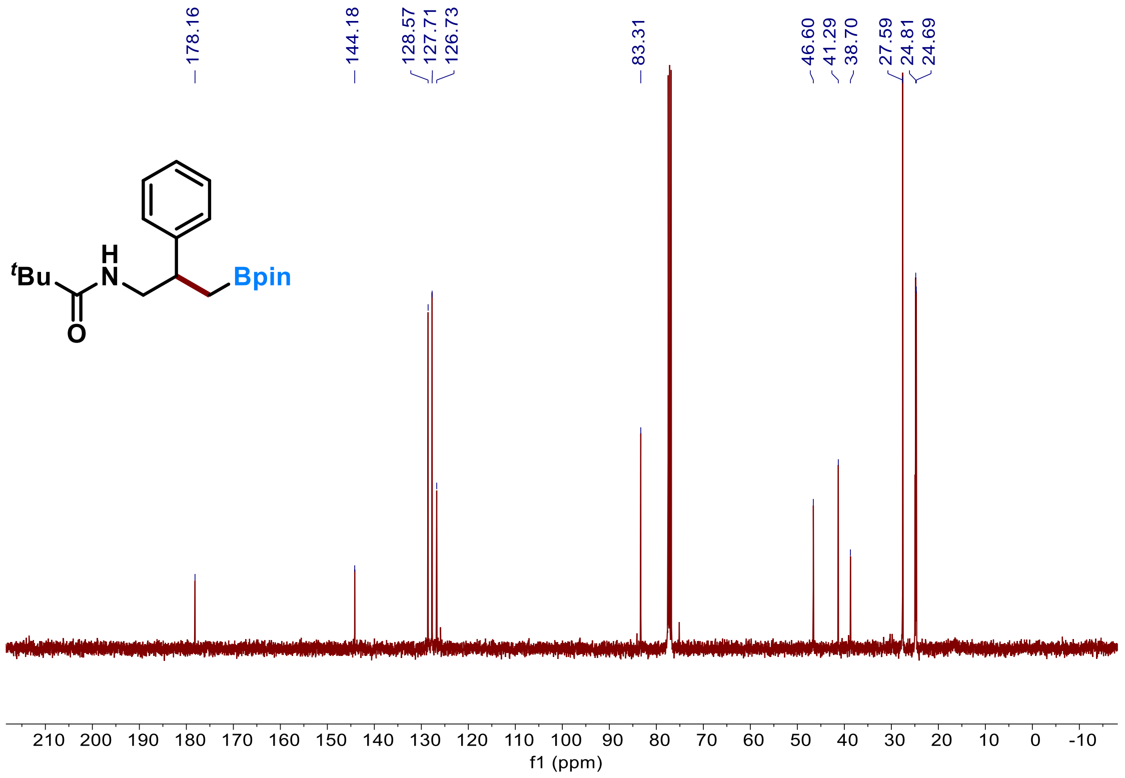

Supplementary Figure 164. ^{13}C{^{1}H} NMR spectrum of **2u** (101 MHz, Chloroform-*d*)

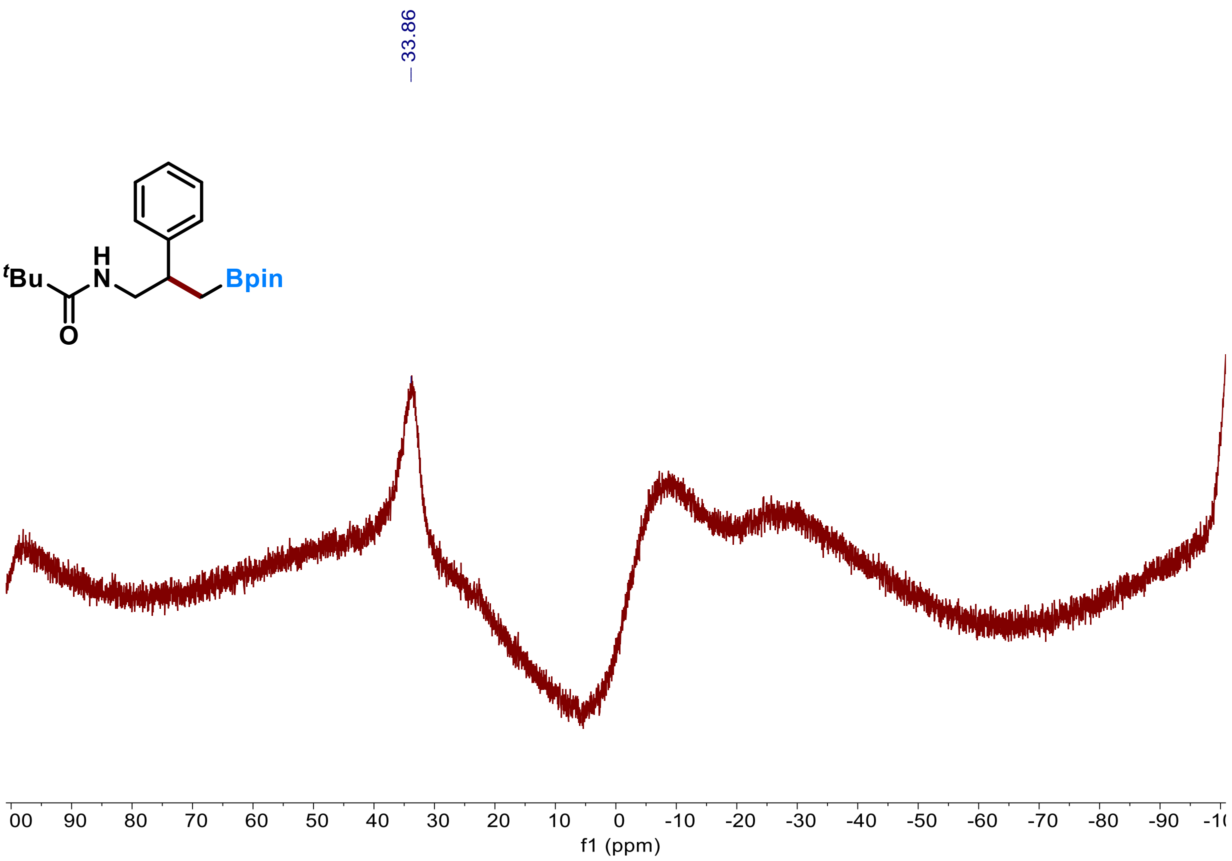

Supplementary Figure 165. ^{11}B NMR spectrum of **2u** (128 MHz, Chloroform-*d*)

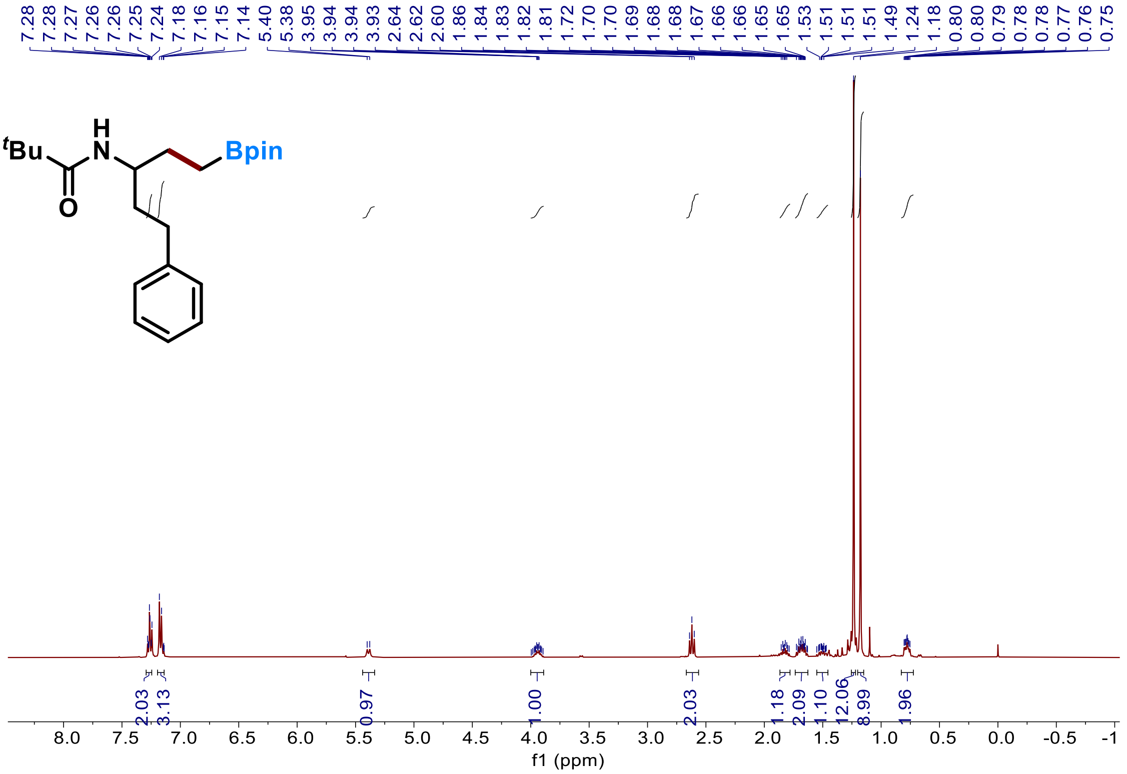

Supplementary Figure 166. ^{1}H NMR spectrum of **2v** (400 MHz, Chloroform-*d*)

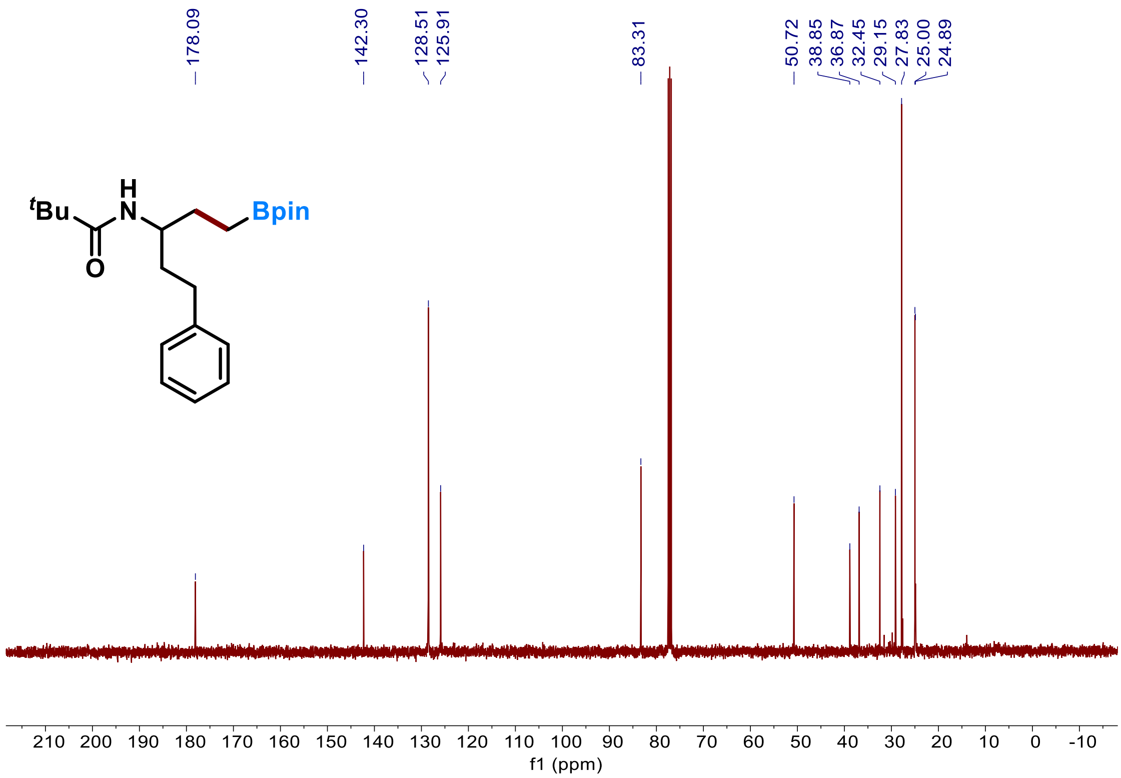

Supplementary Figure 167. $^{13}C\{^{1}H\}$ NMR spectrum of **2v** (101 MHz, Chloroform-*d*)

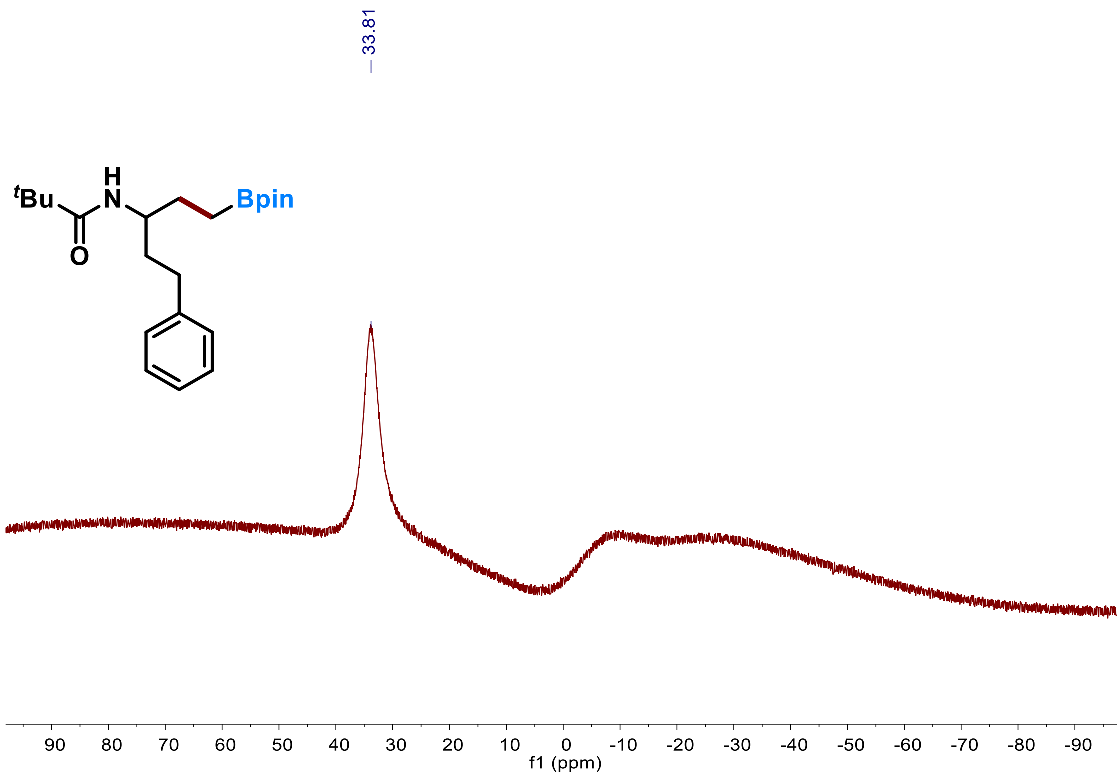

Supplementary Figure 168. ^{11}B NMR spectrum of **2v** (128 MHz, Chloroform-*d*)

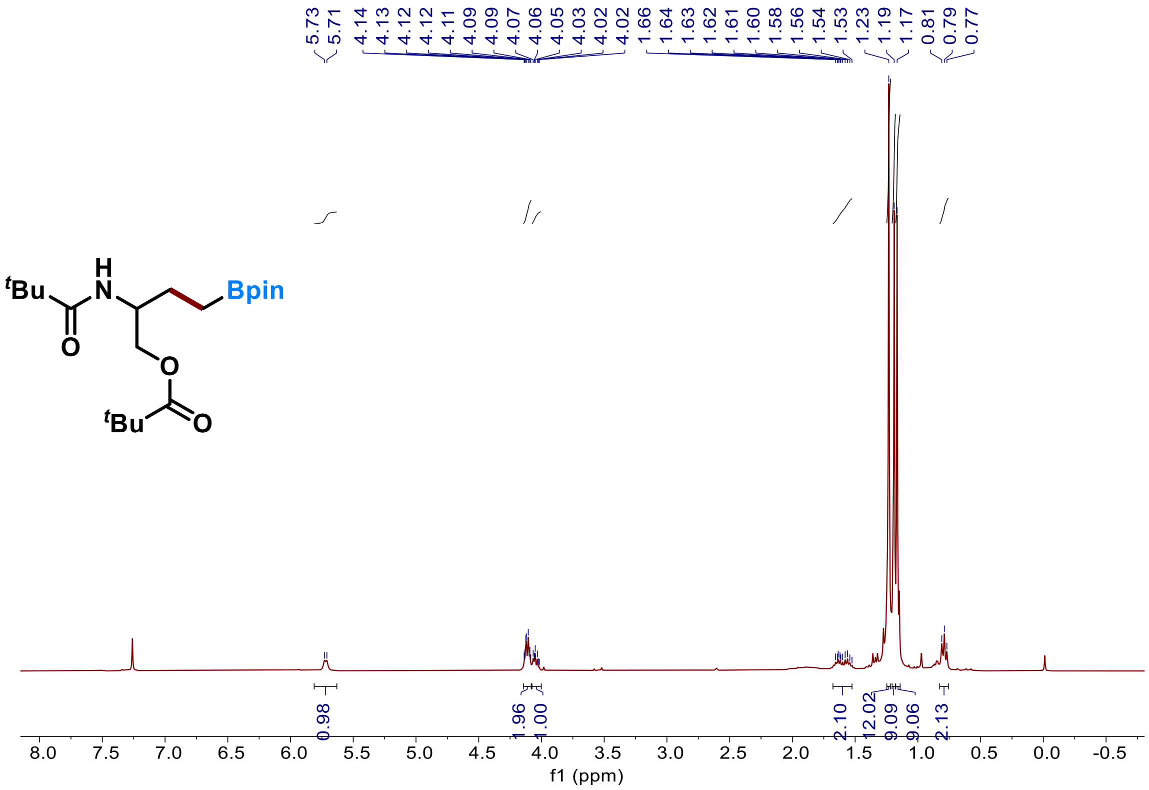

Supplementary Figure 169. ^{1}H NMR spectrum of **2w** (400 MHz, Chloroform-*d*)

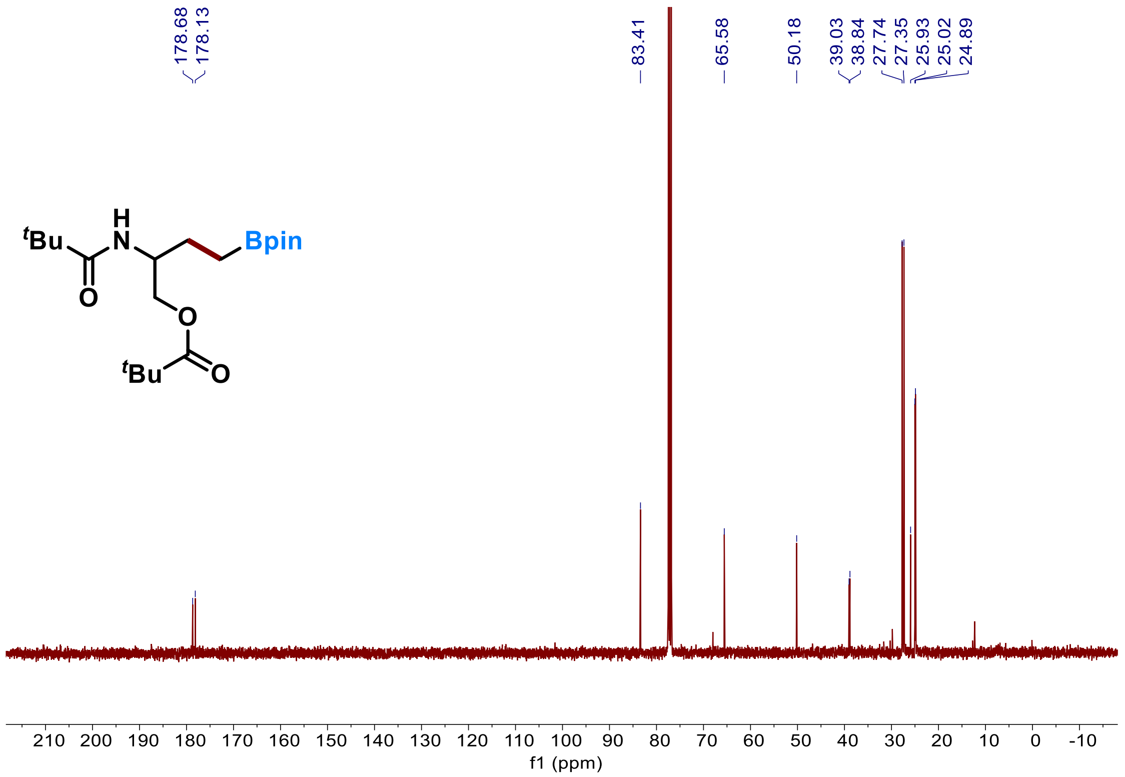

Supplementary Figure 170. $^{13}C\{^{1}H\}$ NMR spectrum of **2w** (101 MHz, Chloroform-*d*)

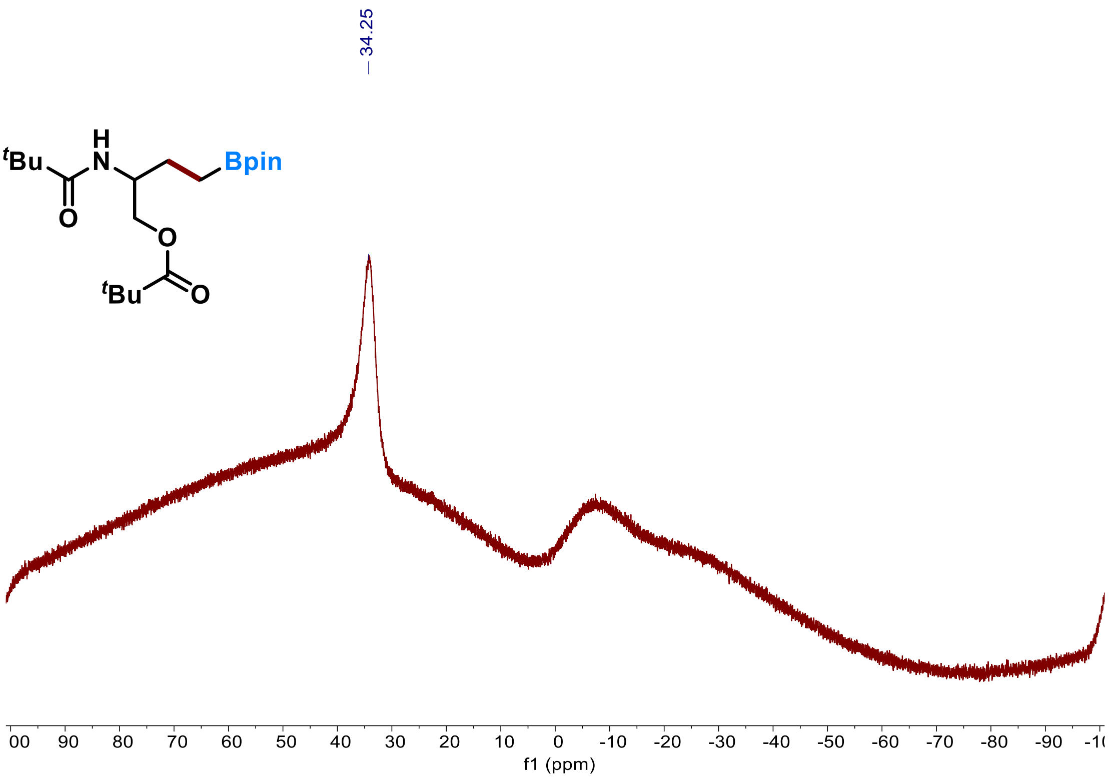

Supplementary Figure 171. ^{11}B NMR spectrum of **2w** (128 MHz, Chloroform-*d*)

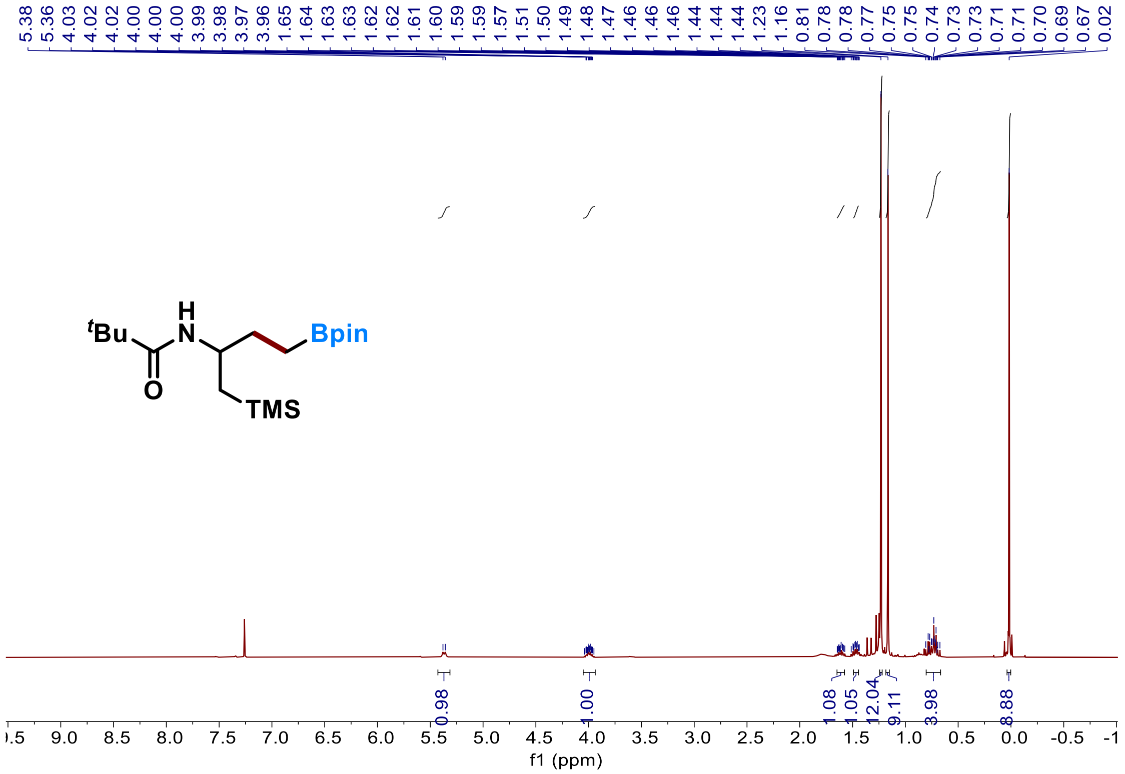

Supplementary Figure 172. ^{1}H NMR spectrum of **2x** (400 MHz, Chloroform-*d*)

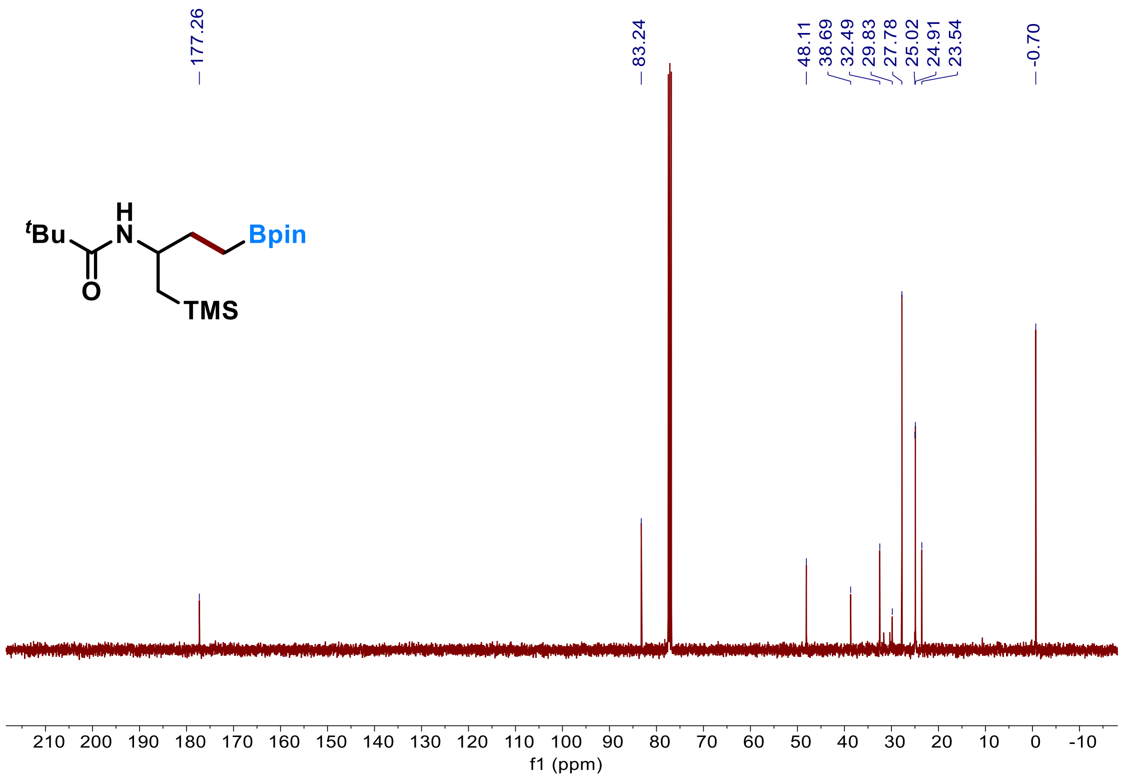

Supplementary Figure 173. $^{13}C\{^1H\}$ NMR spectrum of **2x** (101 MHz, Chloroform-*d*)

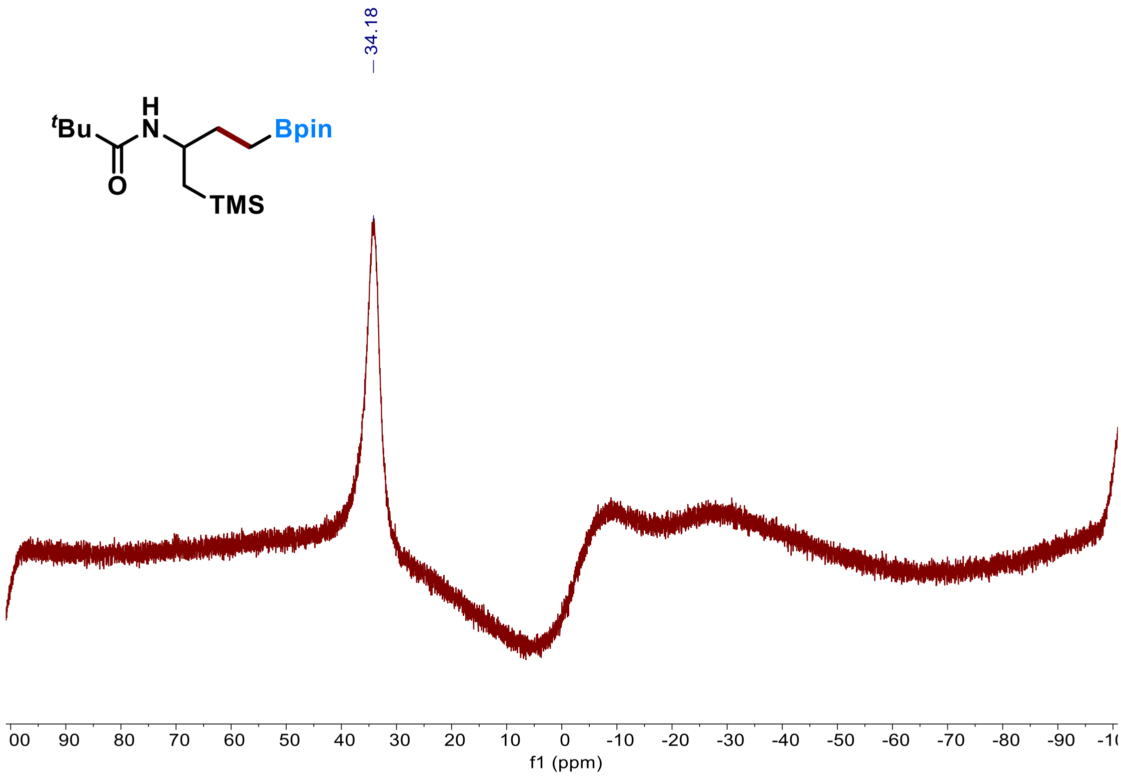

Supplementary Figure 174. ^{11}B NMR spectrum of **2x** (128 MHz, Chloroform-*d*)

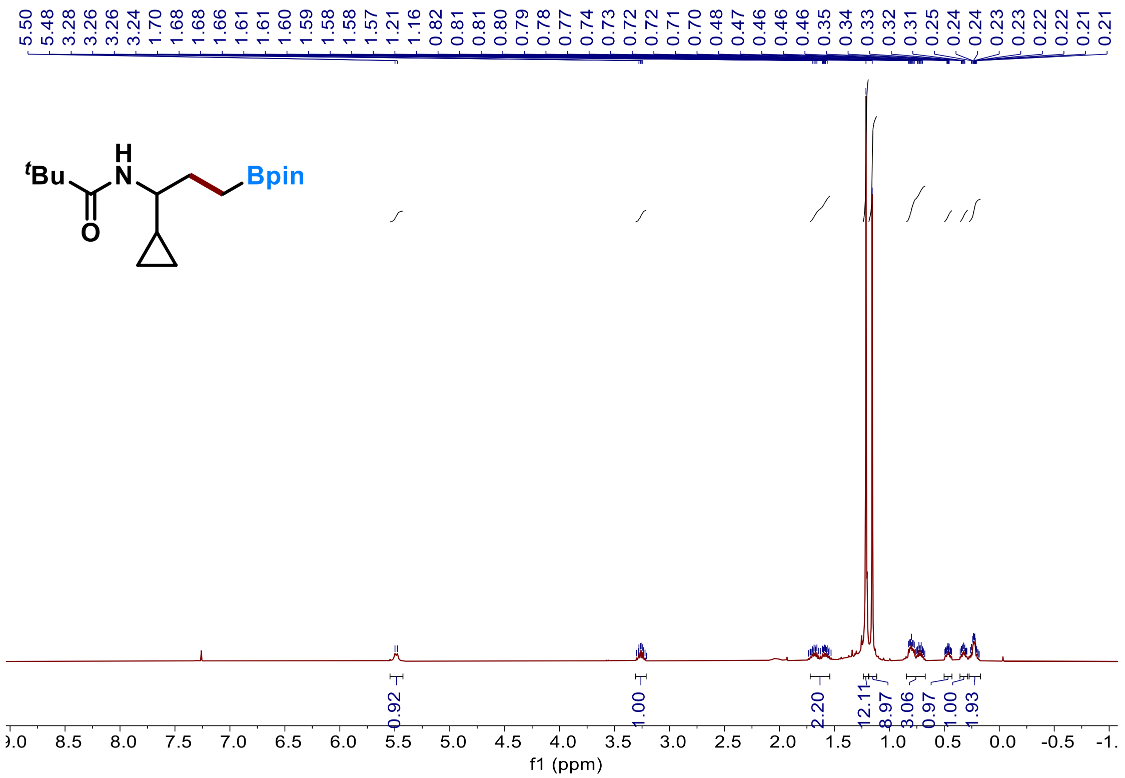

Supplementary Figure 175. ^{1}H NMR spectrum of **2y** (400 MHz, Chloroform-*d*)

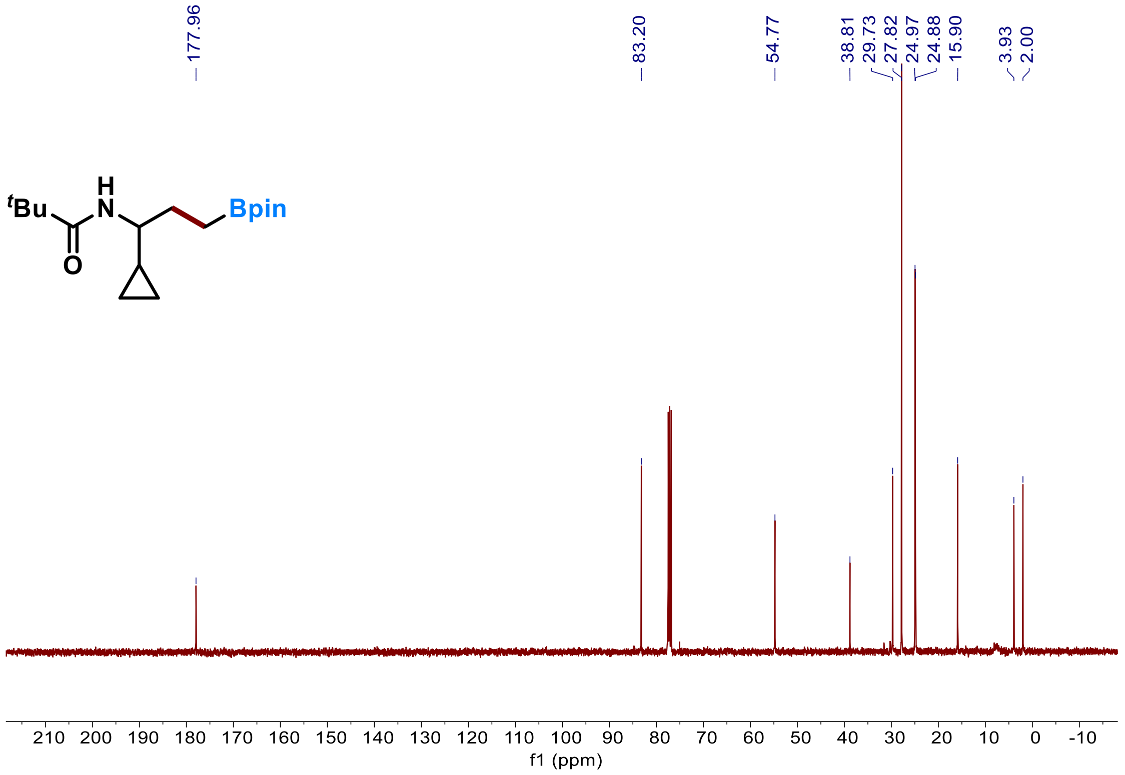

Supplementary Figure 176. ^{13}C{^{1}H} NMR spectrum of **2y** (101 MHz, Chloroform-*d*)

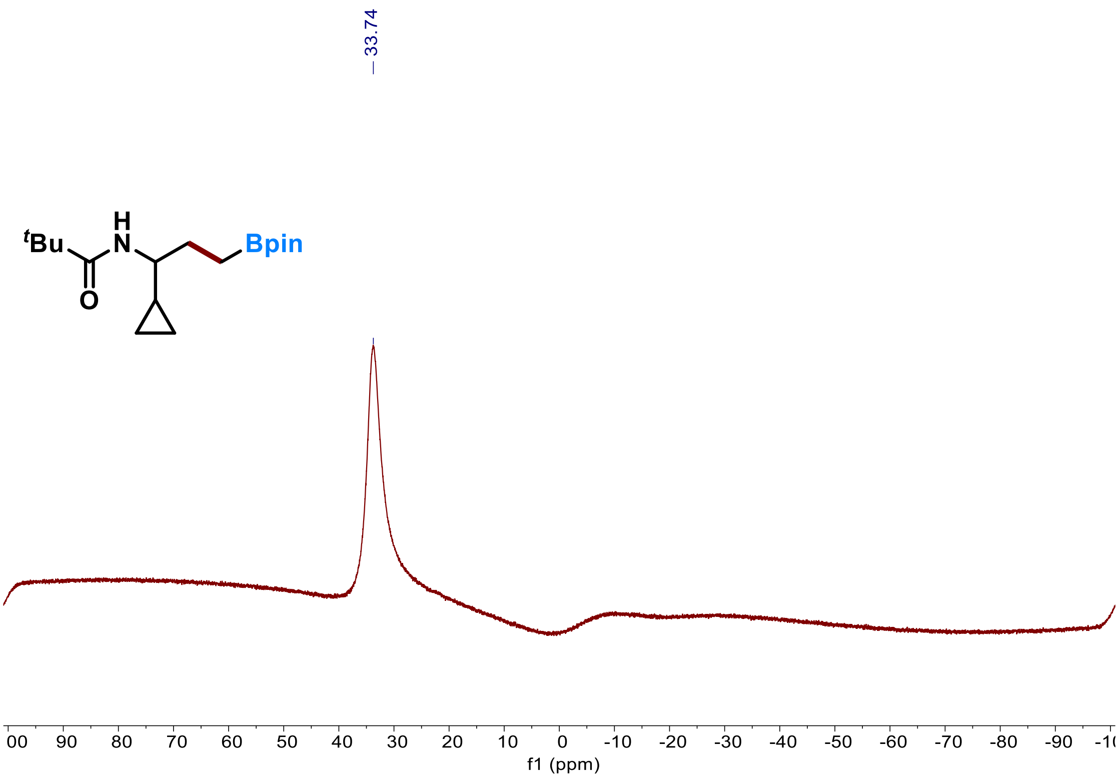

Supplementary Figure 177. ^{11}B NMR spectrum of **2y** (128 MHz, Chloroform-*d*)

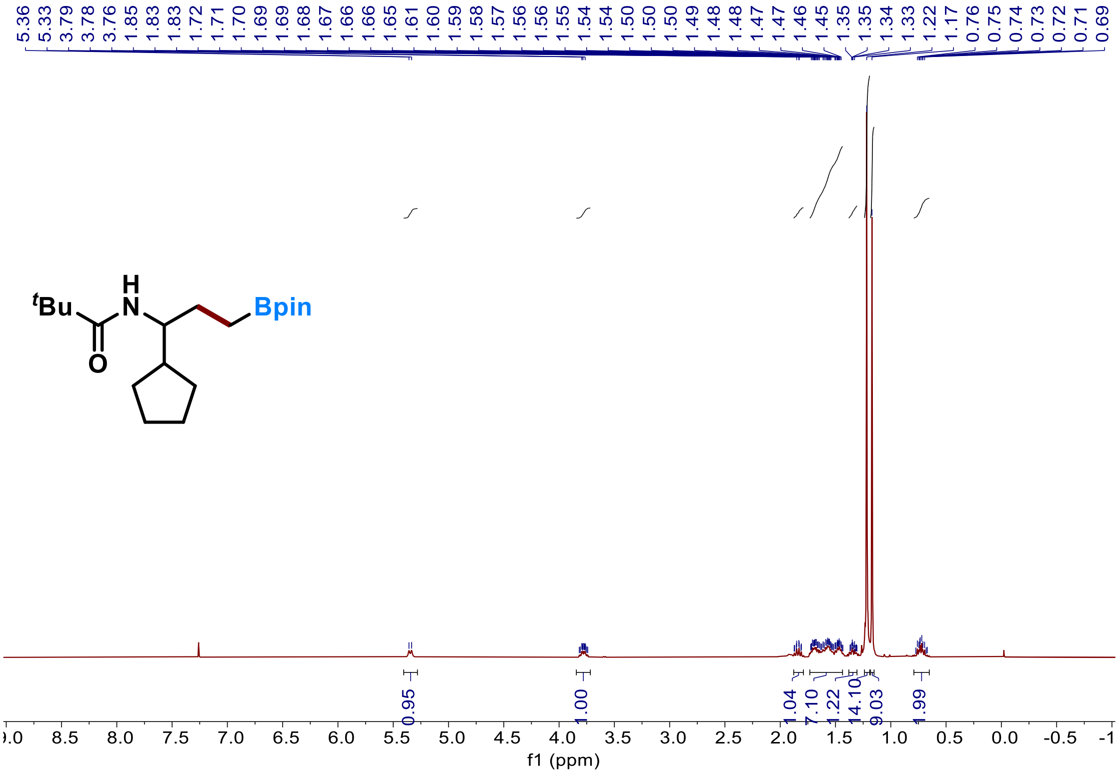

Supplementary Figure 178. ^{1}H NMR spectrum of **2z** (400 MHz, Chloroform-*d*)

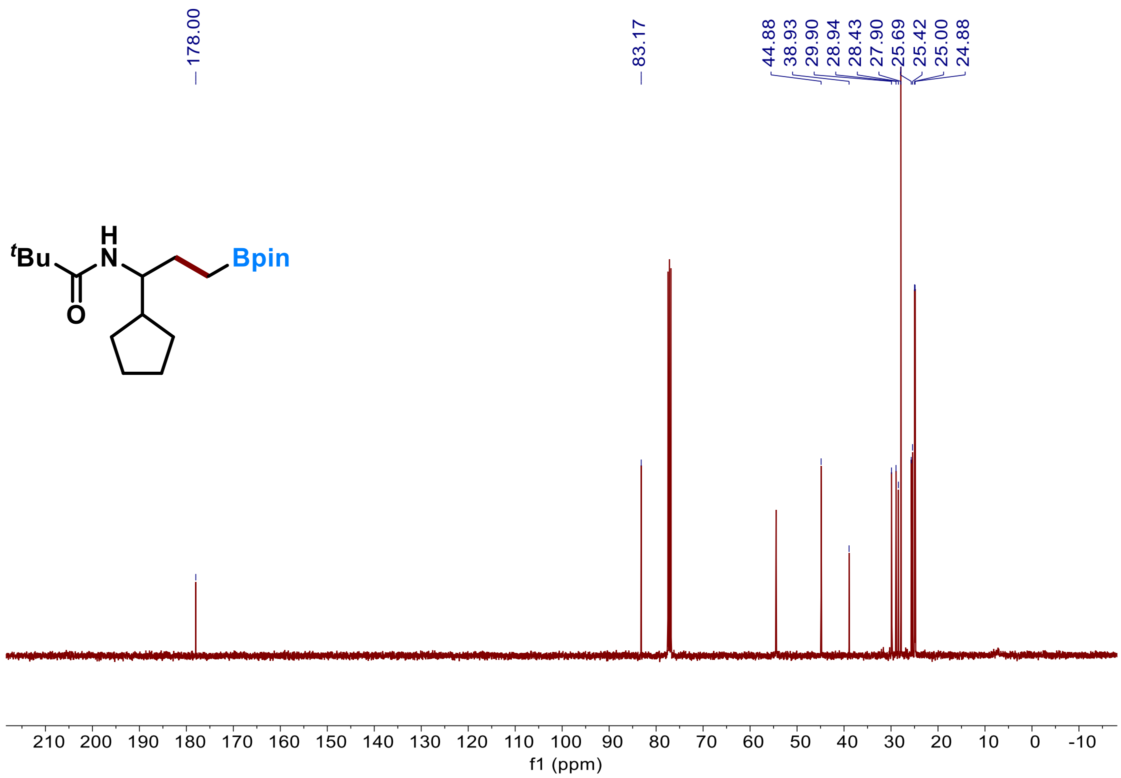

Supplementary Figure 179. $^{13}C\{^{1}H\}$ NMR spectrum of **2z** (101 MHz, Chloroform-*d*)

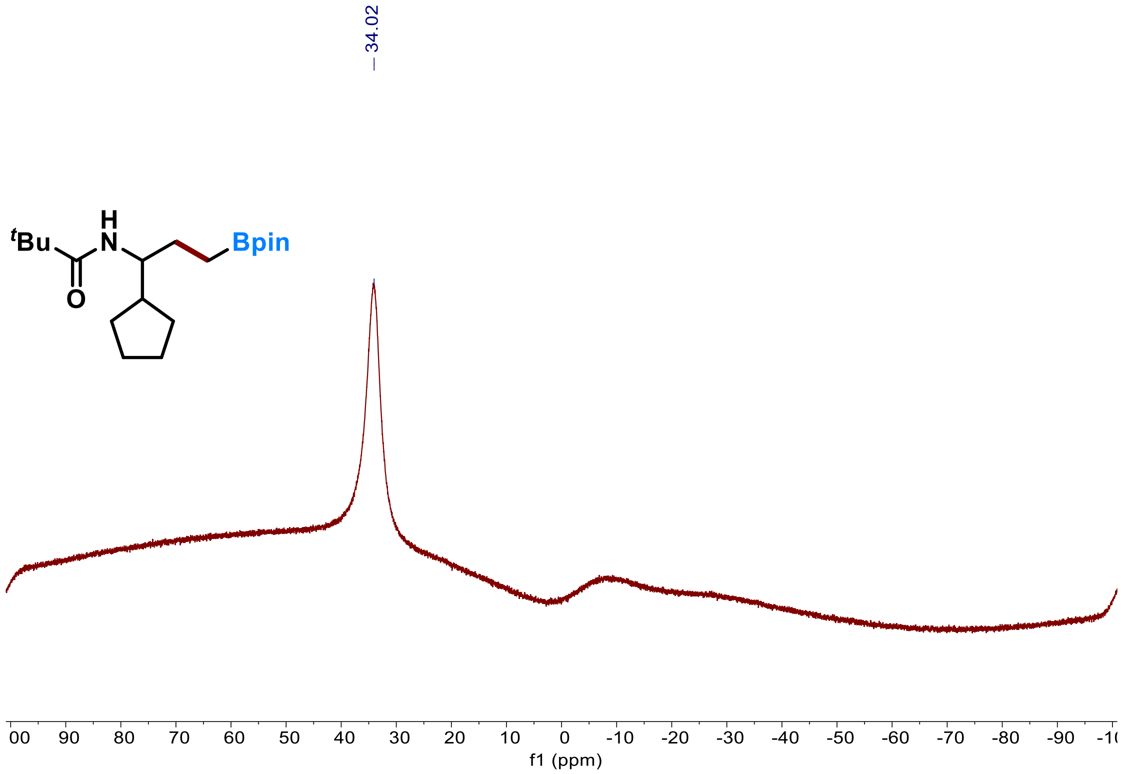

Supplementary Figure 180. ^{11}B NMR spectrum of **2z** (128 MHz, Chloroform-*d*)

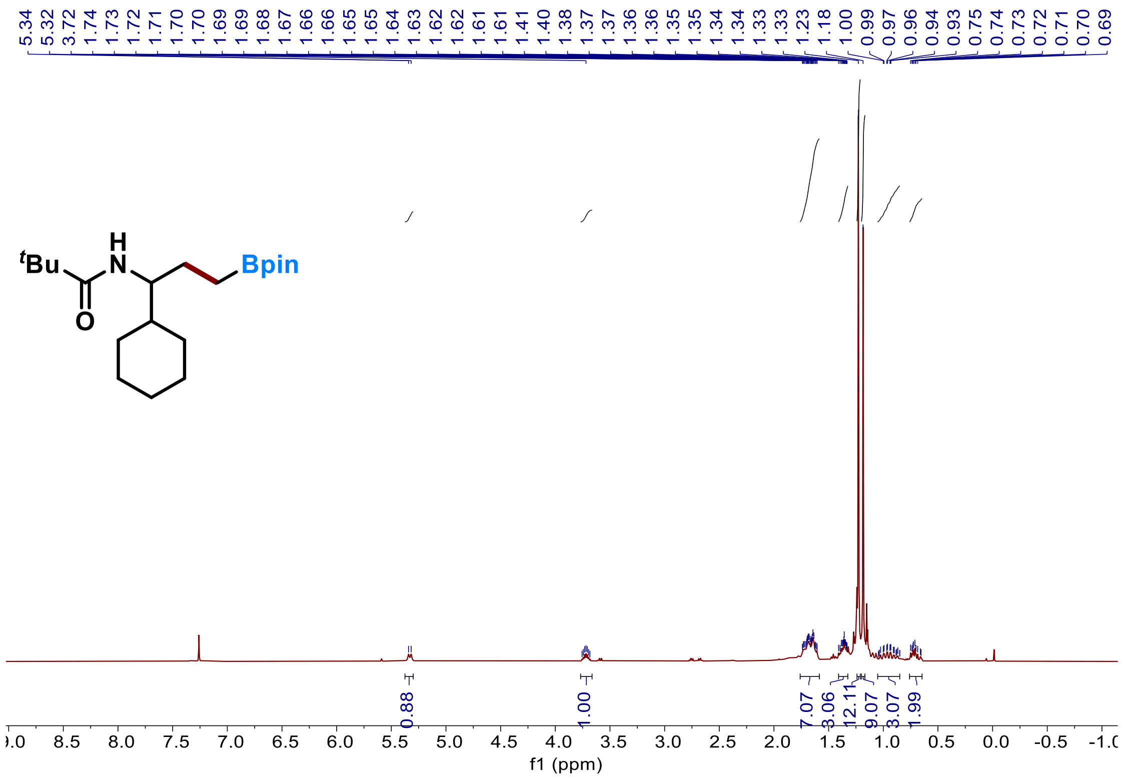

Supplementary Figure 181. ^{1}H NMR spectrum of **2aa** (400 MHz, Chloroform-*d*)

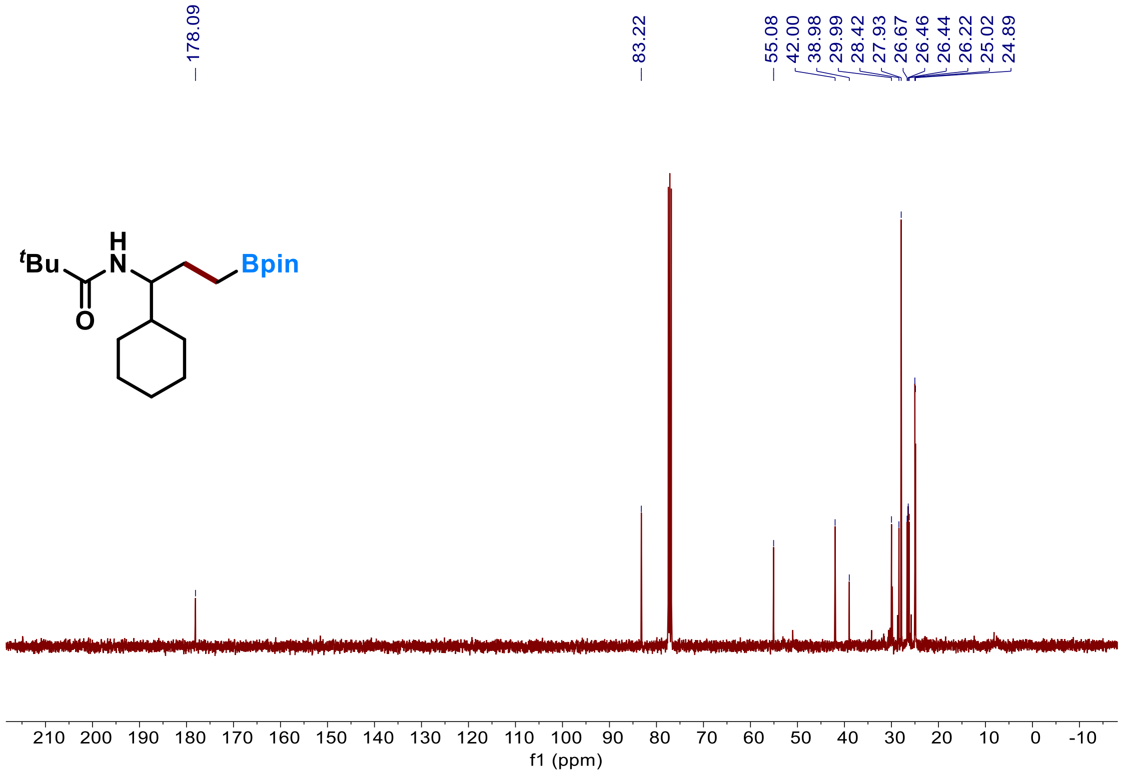

Supplementary Figure 182. $^{13}C\{^{1}H\}$ NMR spectrum of **2aa** (101 MHz, Chloroform-*d*)

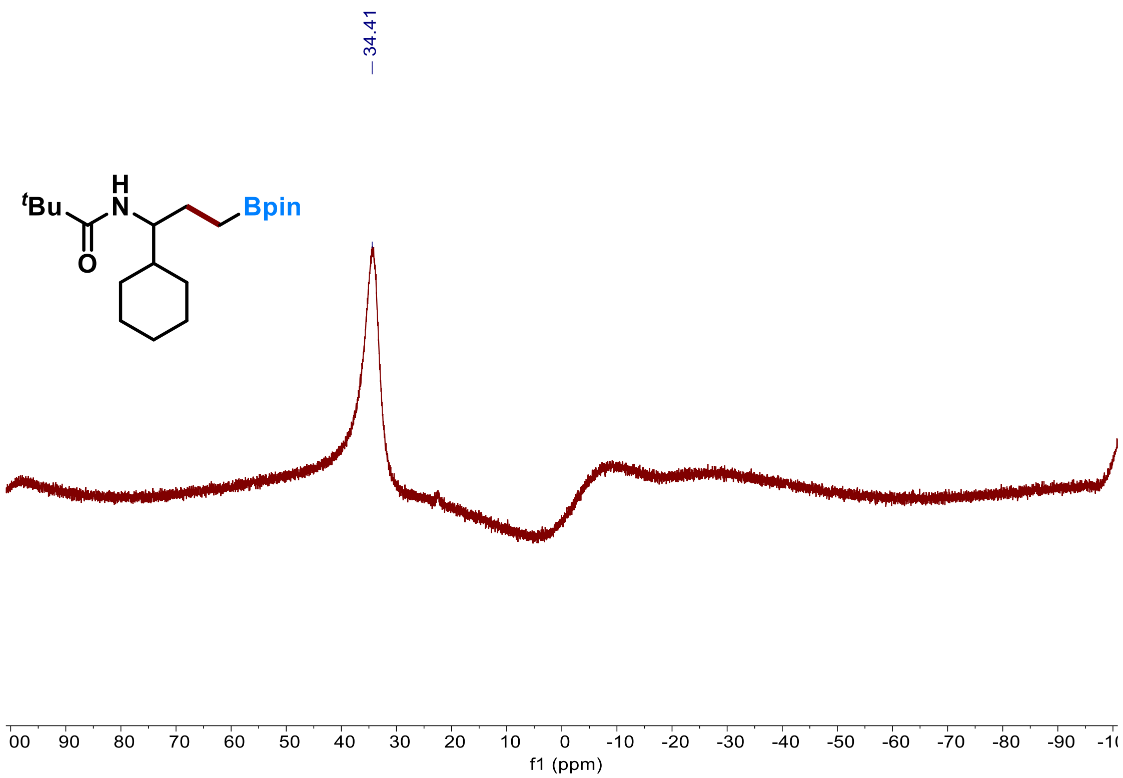

Supplementary Figure 183. ^{11}B NMR spectrum of **2aa** (128 MHz, Chloroform-*d*)

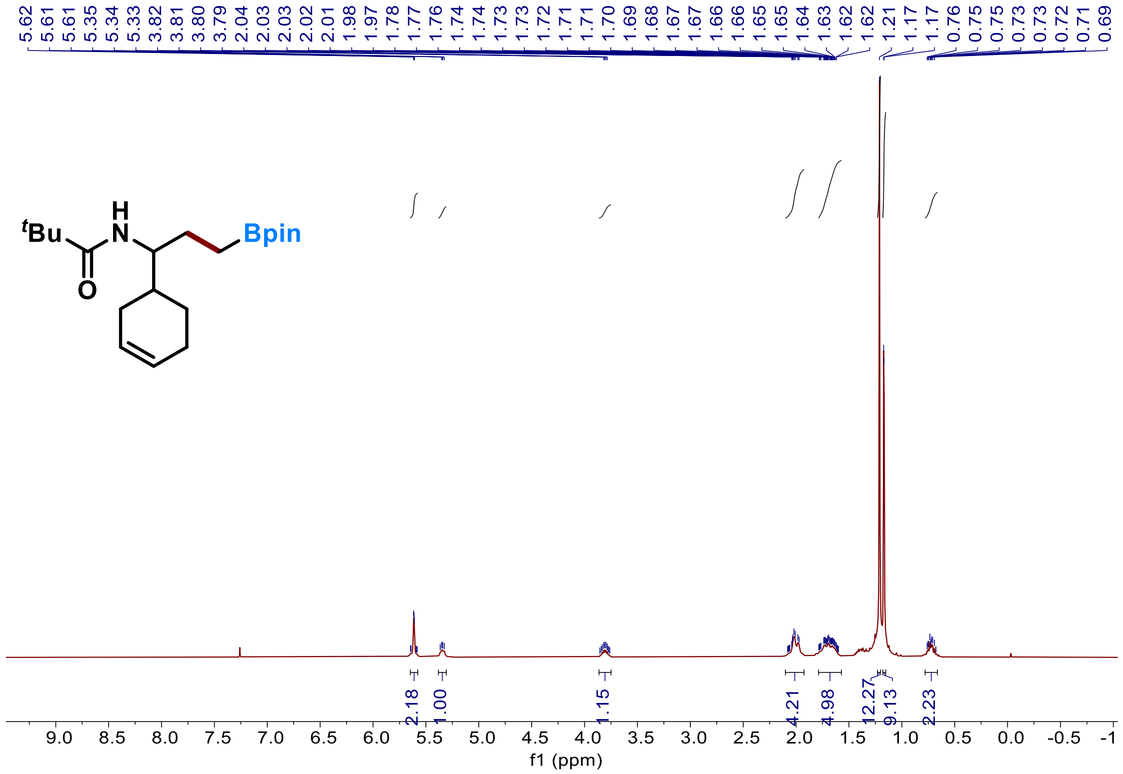

Supplementary Figure 184. ^{1}H NMR spectrum of **2ab** (400 MHz, Chloroform-*d*)

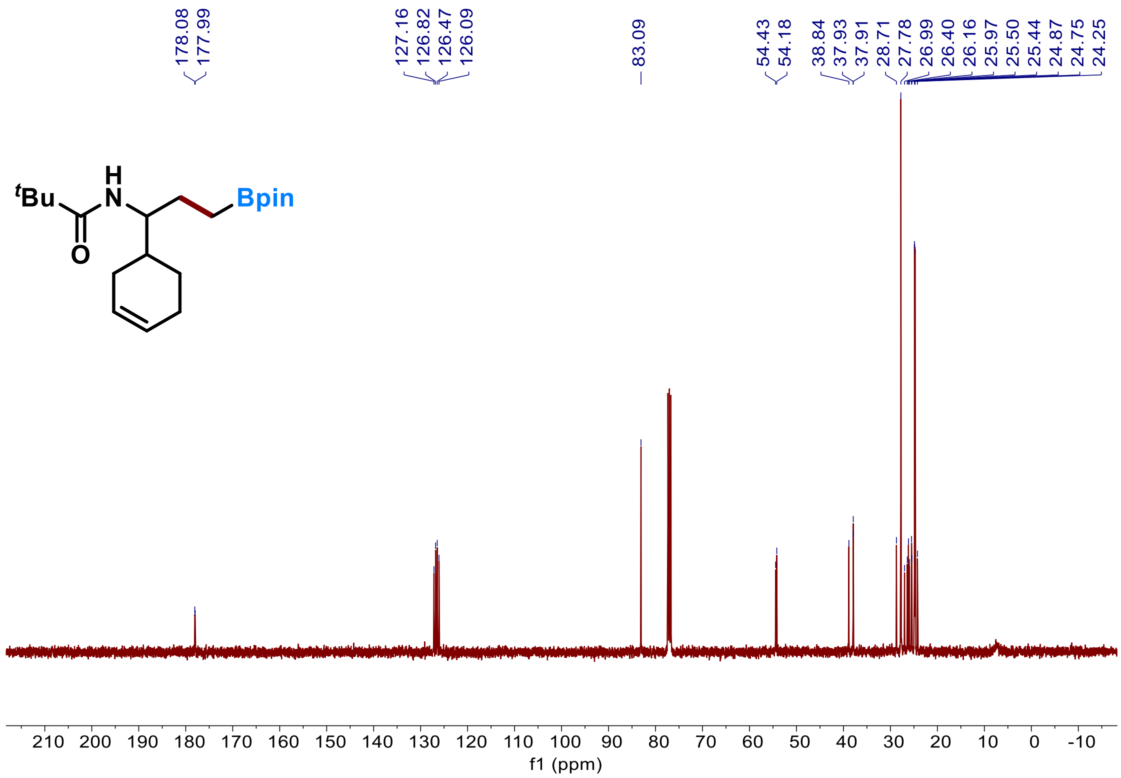

Supplementary Figure 185. $^{13}C\{^{1}H\}$ NMR spectrum of **2ab** (101 MHz, Chloroform-*d*)

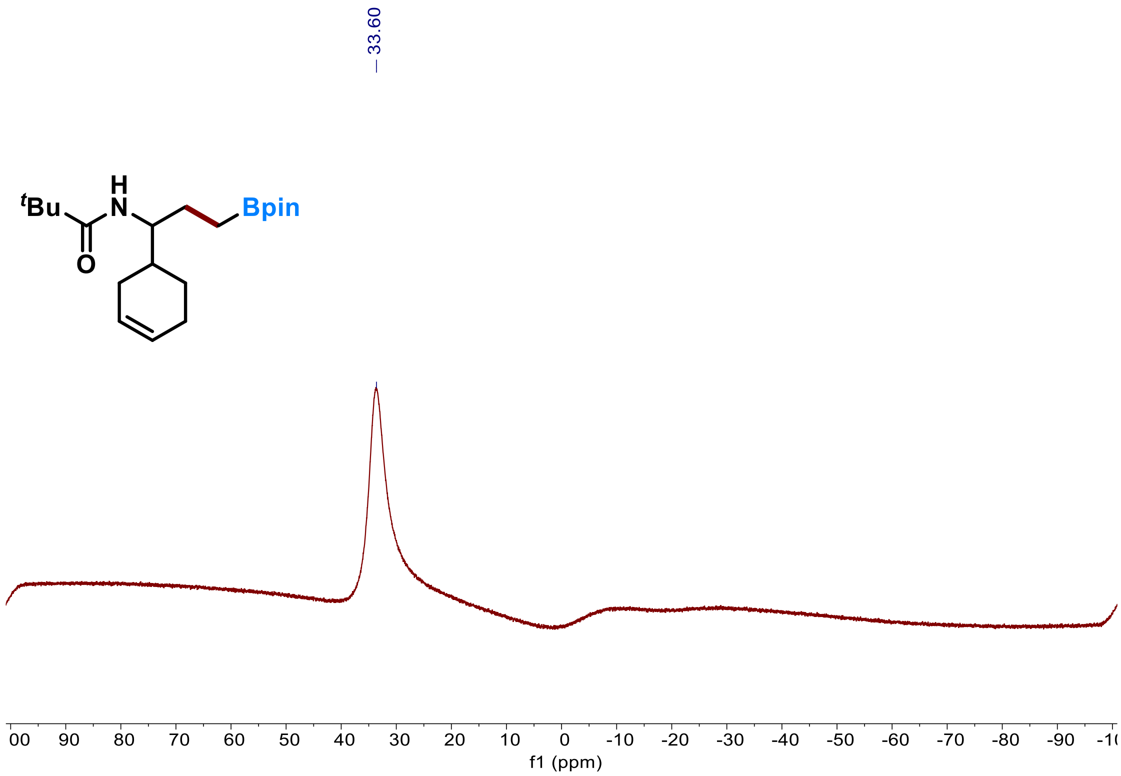

Supplementary Figure 186. ^{11}B NMR spectrum of **2ab** (128 MHz, Chloroform-*d*)

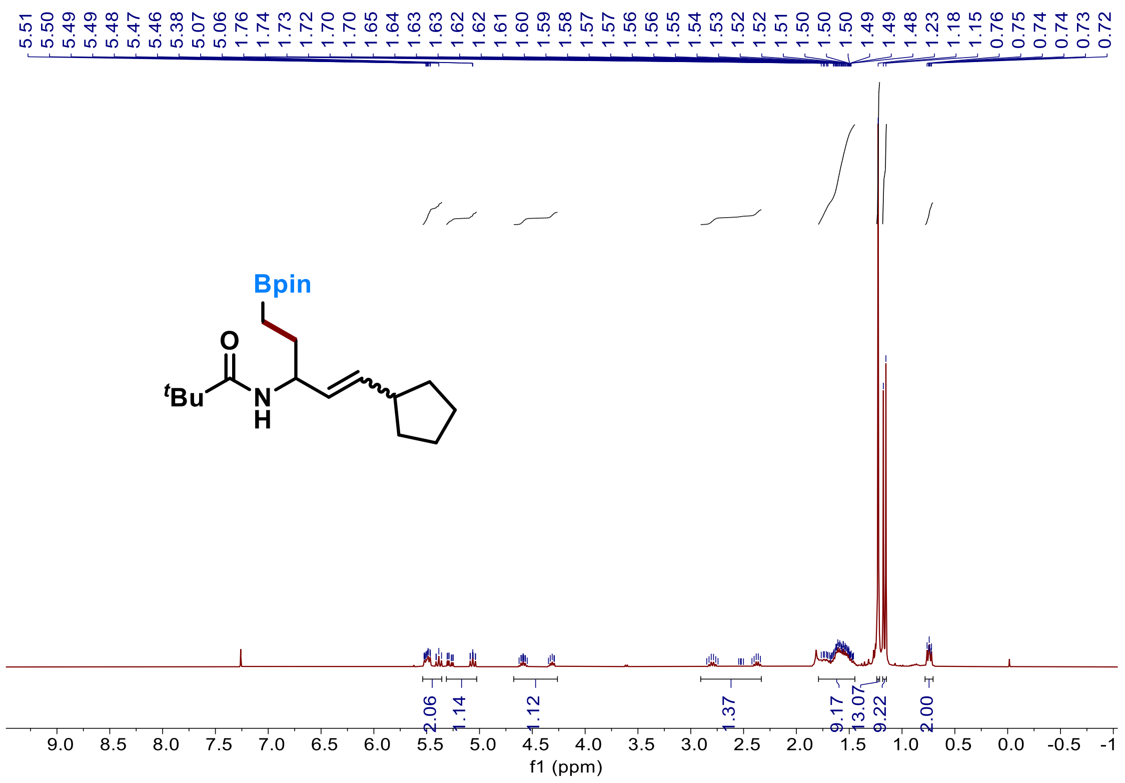

Supplementary Figure 187. ^{1}H NMR spectrum of **2ac** (400 MHz, Chloroform-*d*)

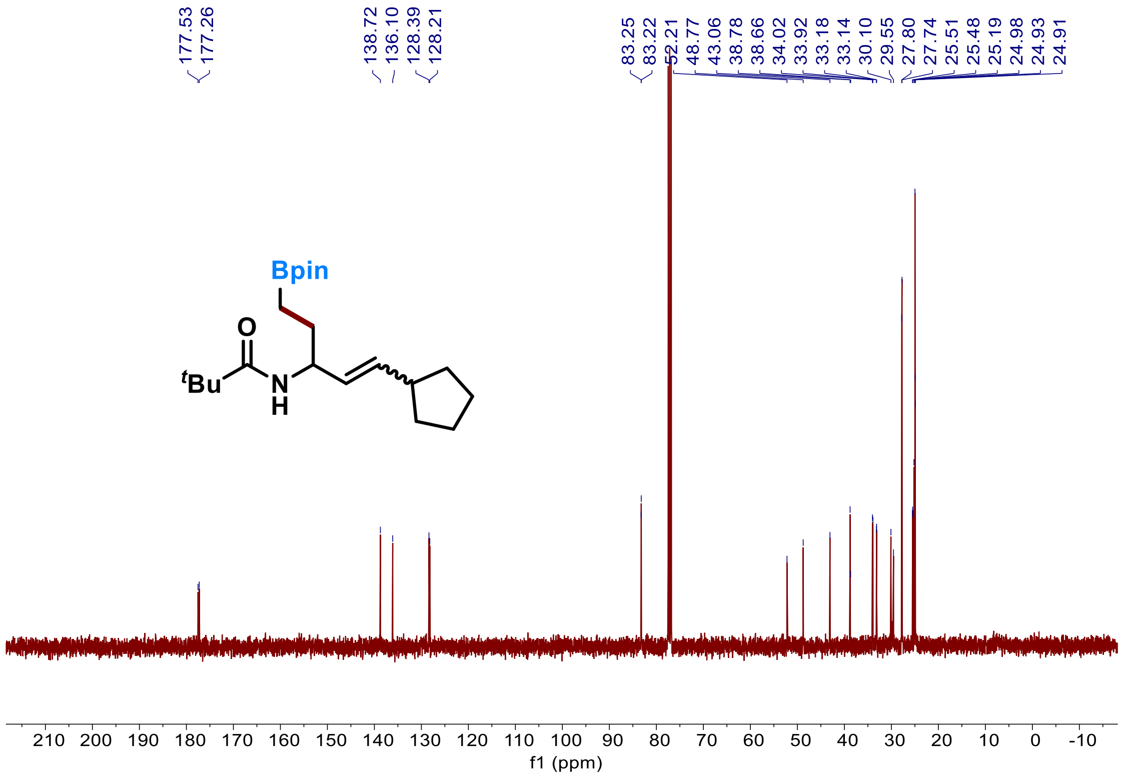

Supplementary Figure 188. ^{13}C{^{1}H} NMR spectrum of **2ac**(101 MHz, Chloroform-*d*)

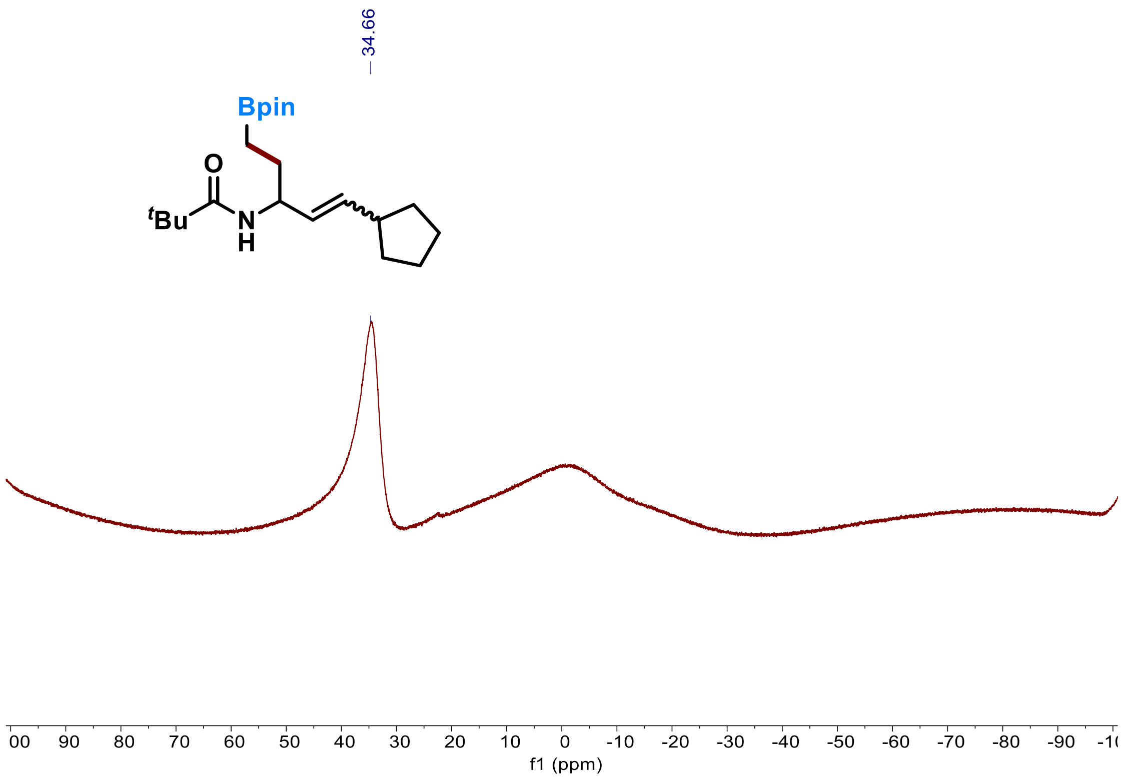

Supplementary Figure 189. ^{11}B NMR spectrum of **2ac** (128 MHz, Chloroform-*d*)

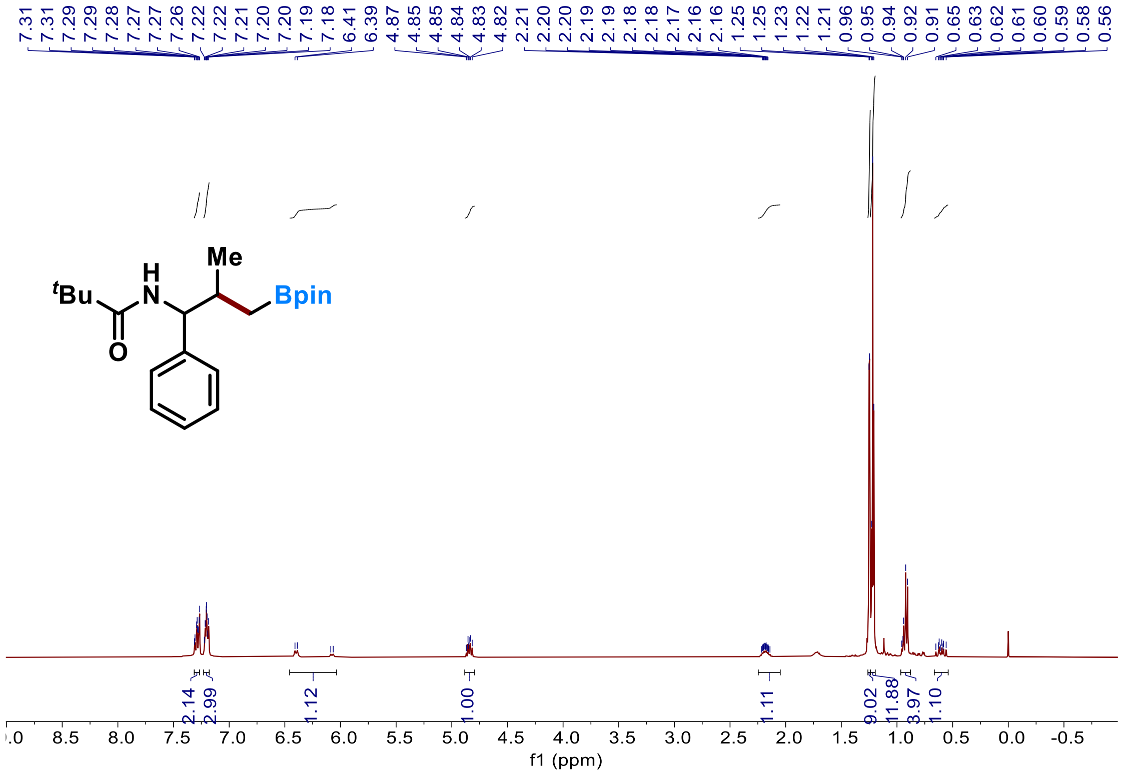

Supplementary Figure 190. ^{1}H NMR spectrum of **2ad** (400 MHz, Chloroform-*d*)

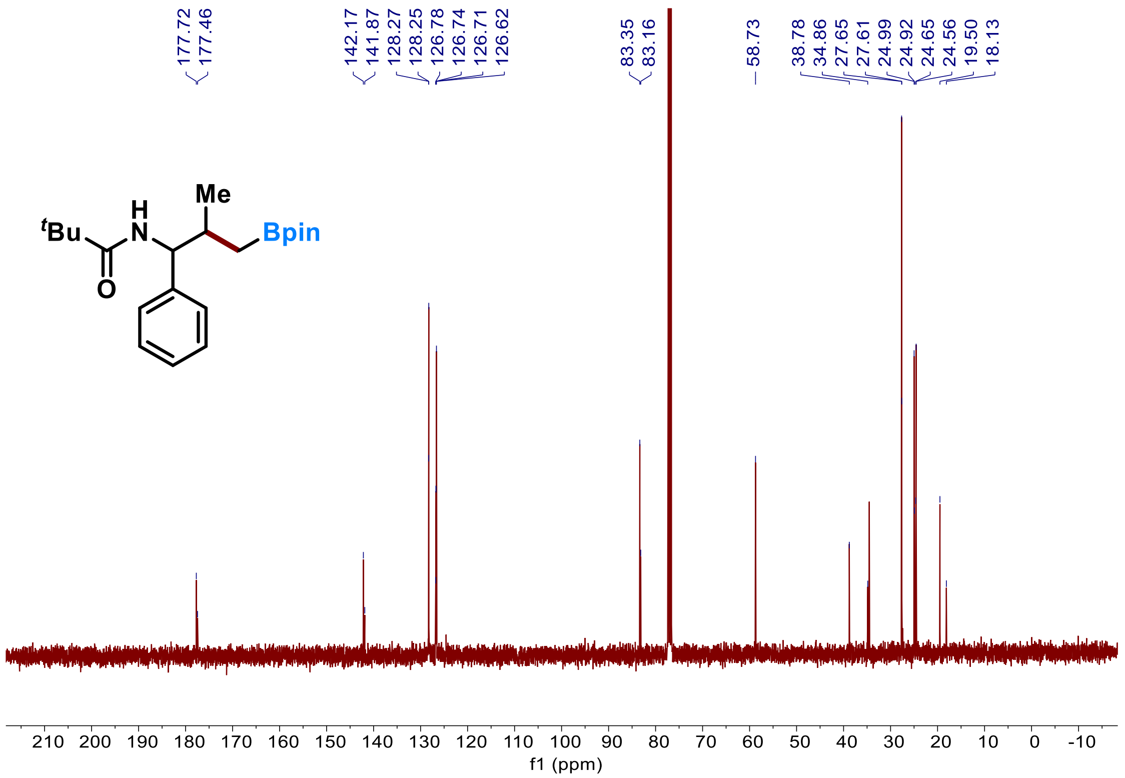

Supplementary Figure 191. $^{13}C\{^{1}H\}$ NMR spectrum of **2ad** (101 MHz, Chloroform-*d*)

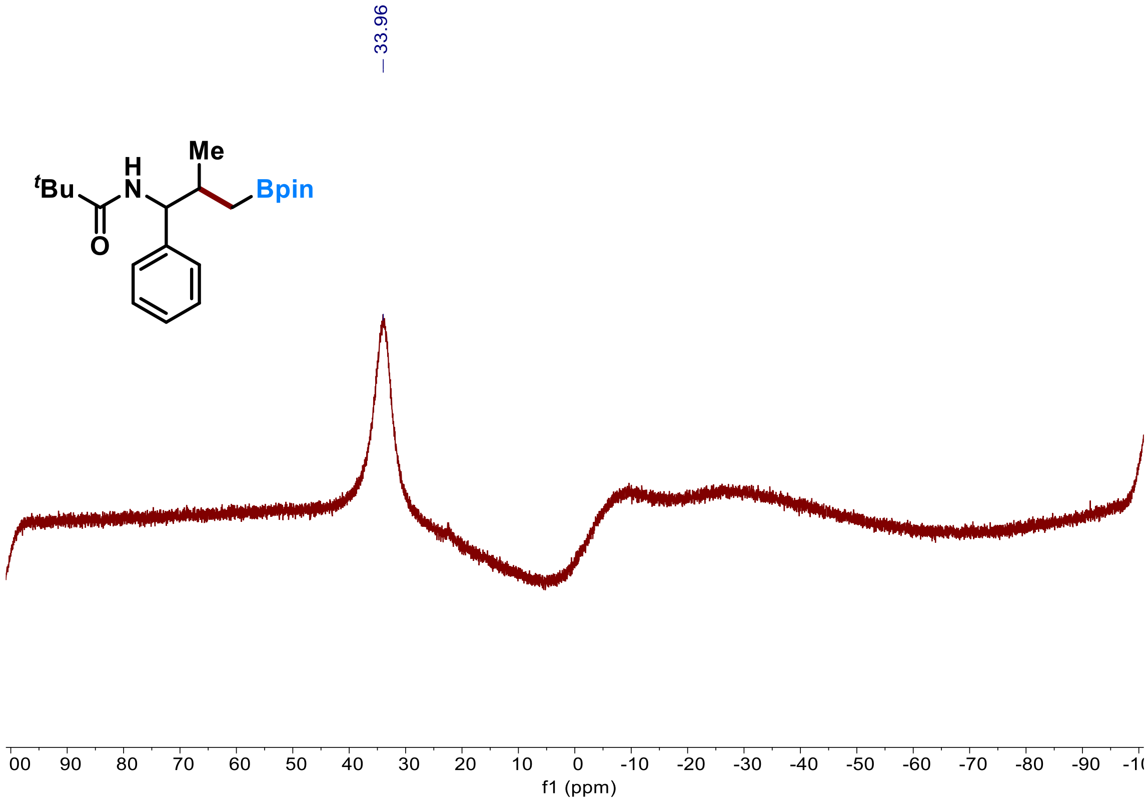

Supplementary Figure 192. ^{11}B NMR spectrum of **2ad** (128 MHz, Chloroform-*d*)

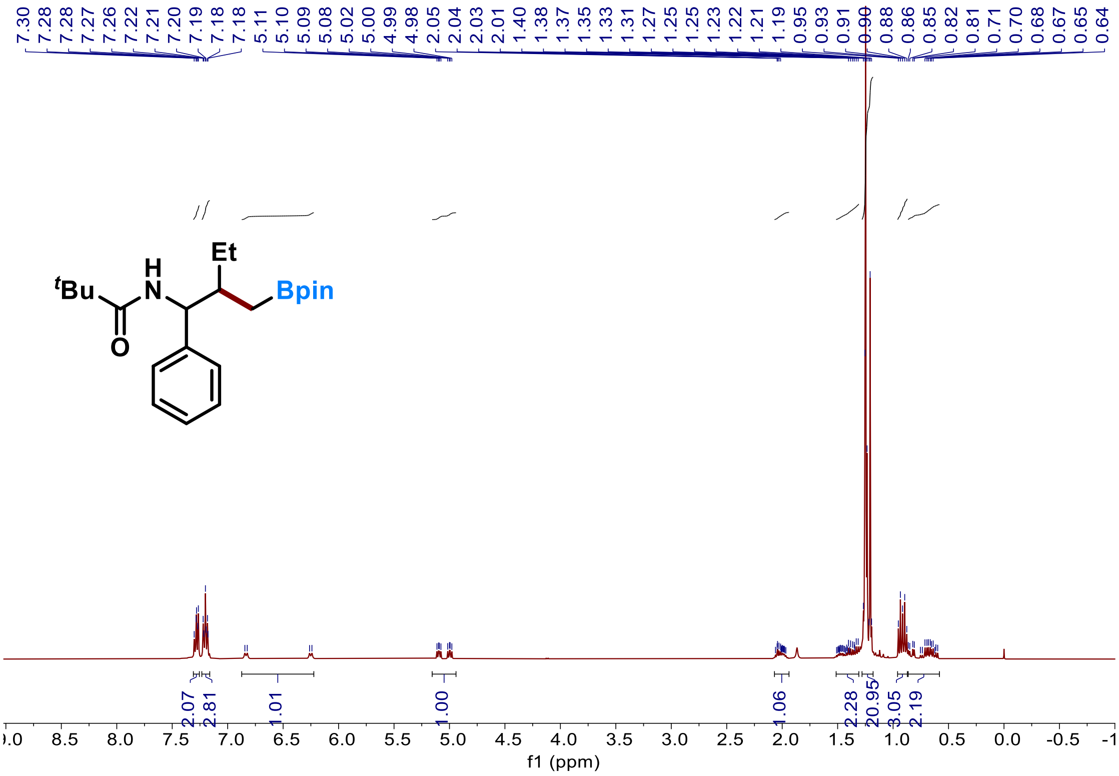

Supplementary Figure 193. ^{1}H NMR spectrum of **2ae** (400 MHz, Chloroform-*d*)

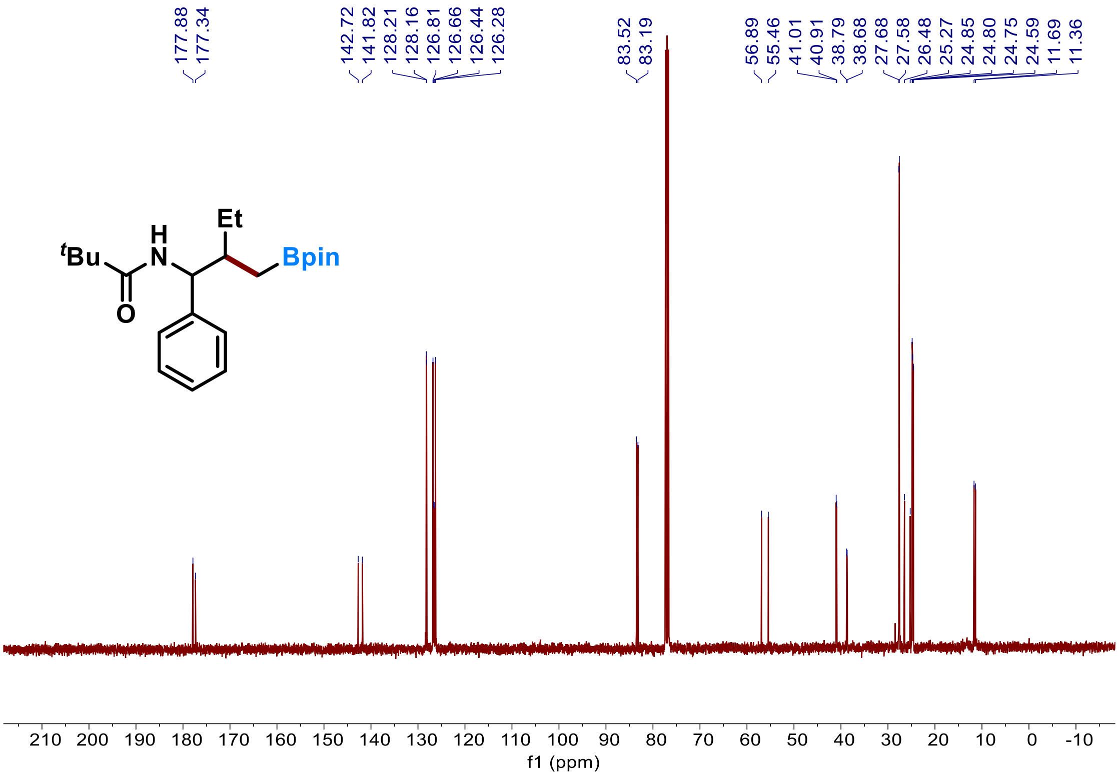

Supplementary Figure 194. $^{13}C\{^{1}H\}$ NMR spectrum of **2ae** (101 MHz, Chloroform-*d*)

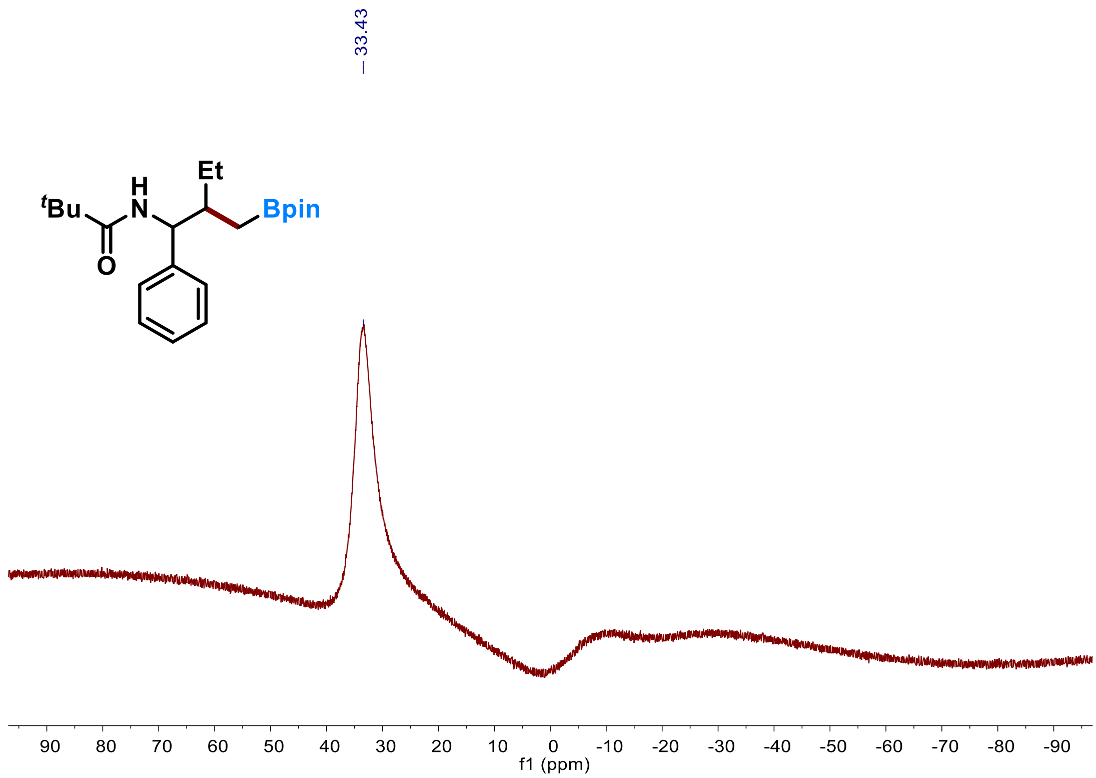

Supplementary Figure 195. ^{11}B NMR spectrum of **2ae** (128 MHz, Chloroform-*d*)

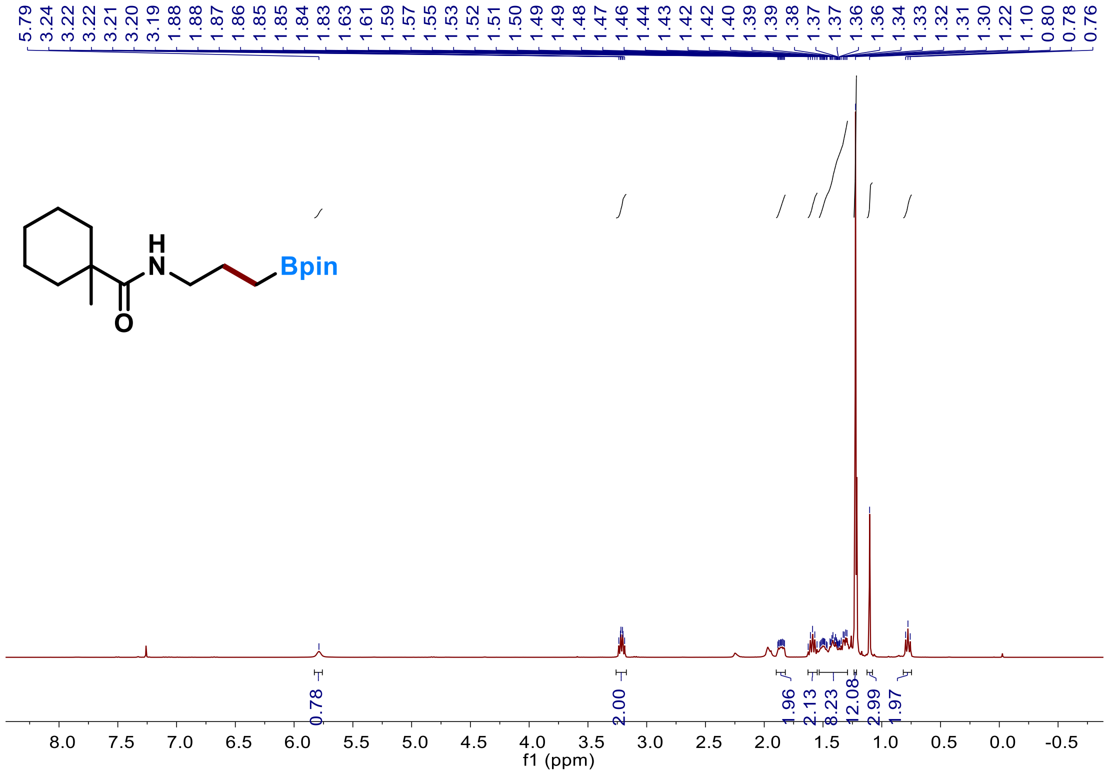

Supplementary Figure 196. ^{1}H NMR spectrum of **2af** (400 MHz, Chloroform-*d*)

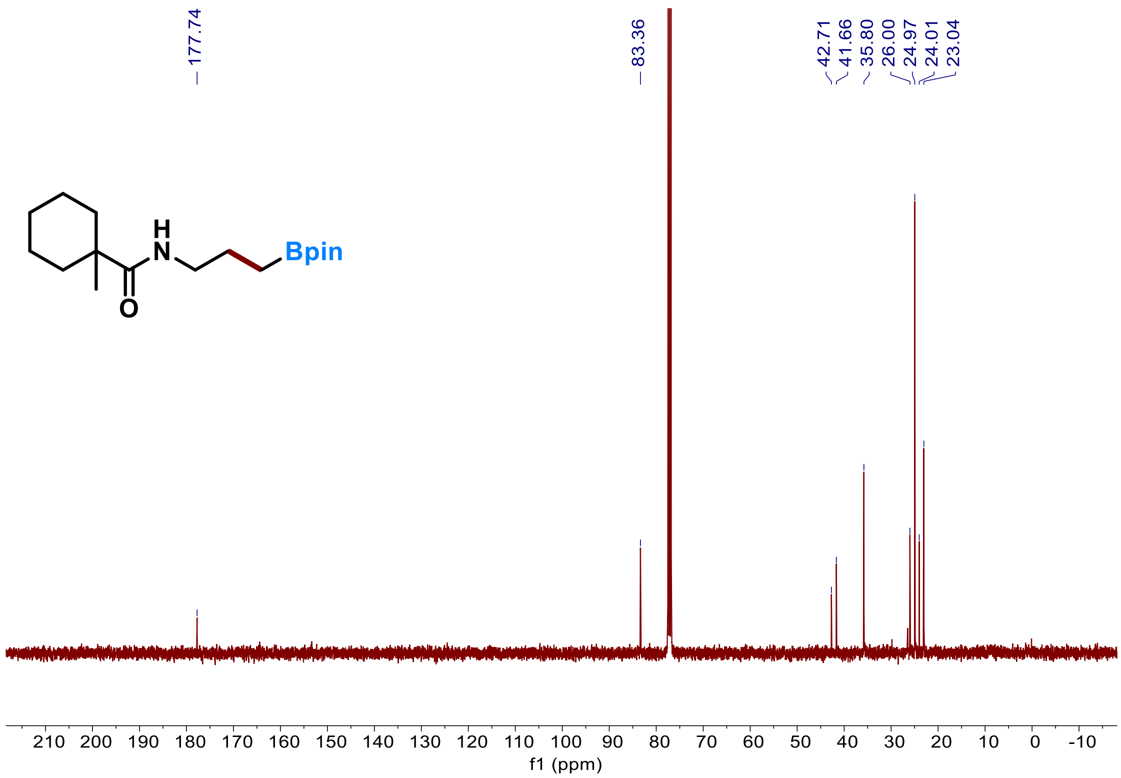

Supplementary Figure 197. $^{13}C\{^{1}H\}$ NMR spectrum of **2af** (101 MHz, Chloroform-*d*)

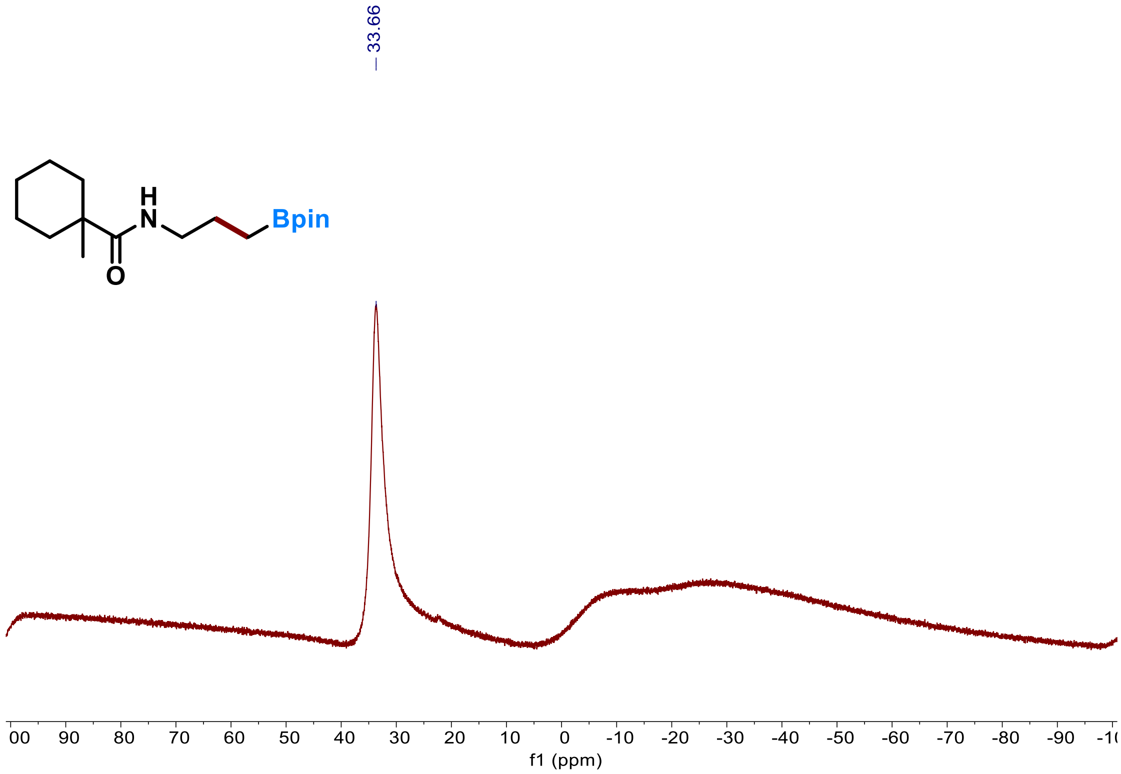

Supplementary Figure 198. ^{11}B NMR spectrum of **2af** (128 MHz, Chloroform-*d*)

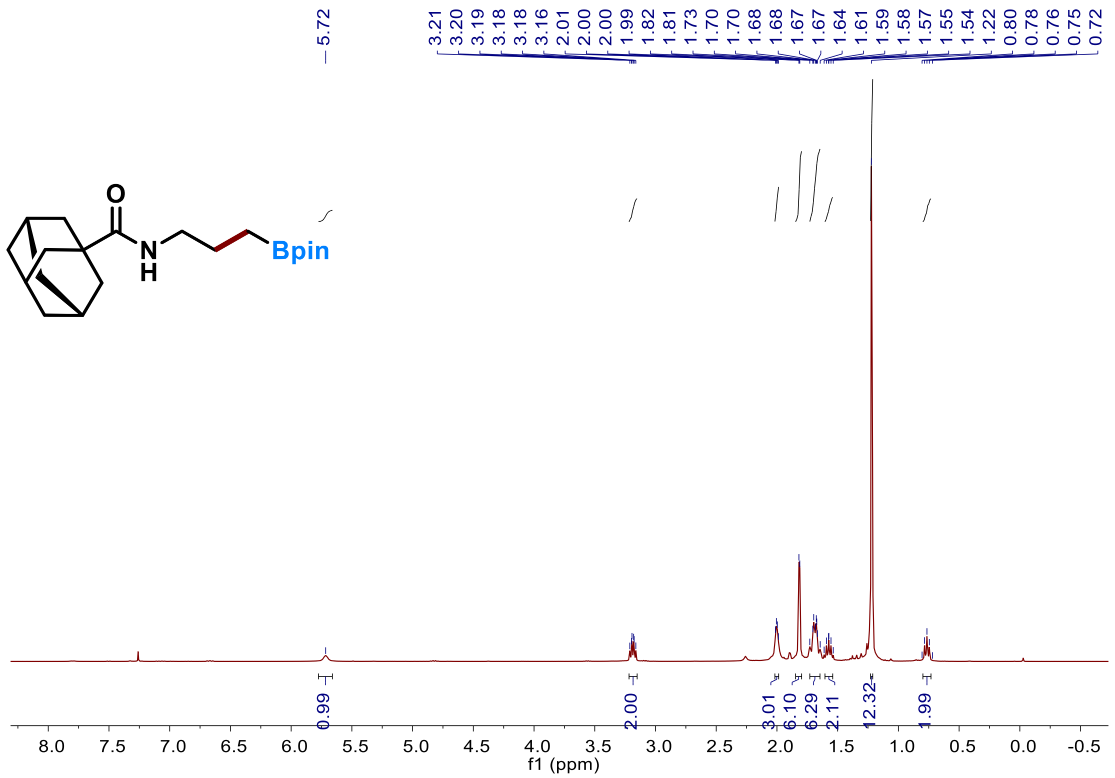

Supplementary Figure 199. ^{1}H NMR spectrum of **2ag** (400 MHz, Chloroform-*d*)

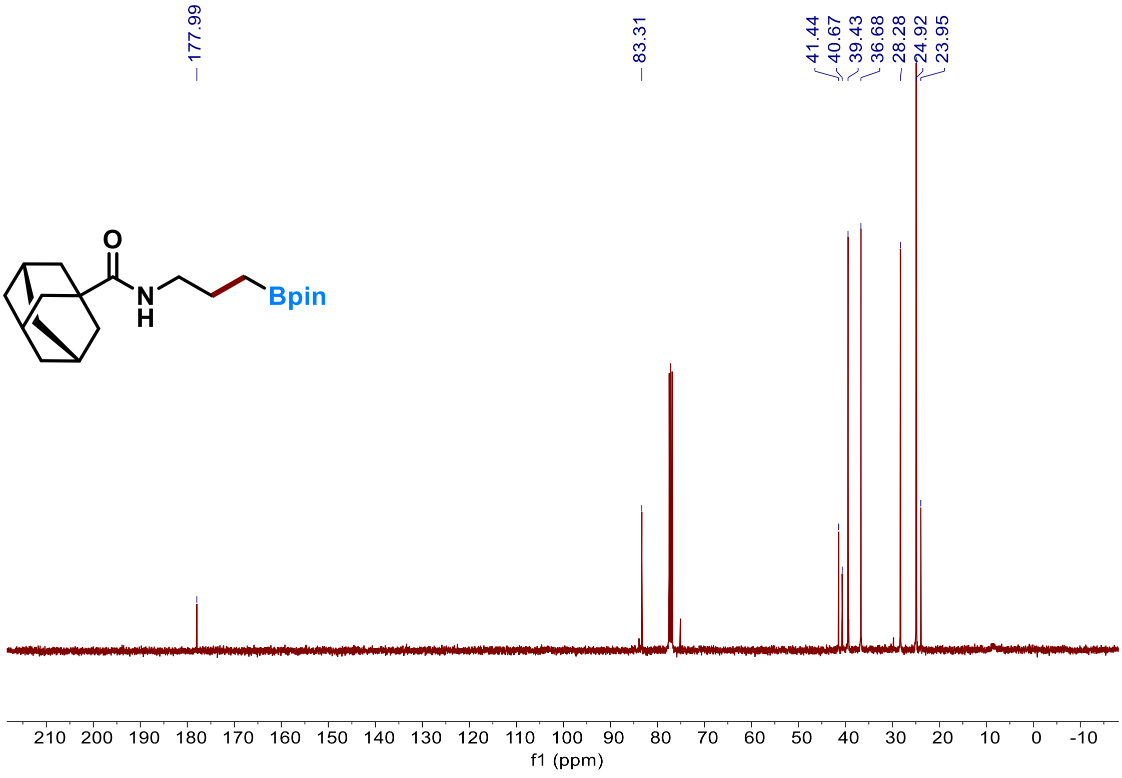

Supplementary Figure 200. $^{13}C\{^{1}H\}$ NMR spectrum of **2ag** (101 MHz, Chloroform-*d*)

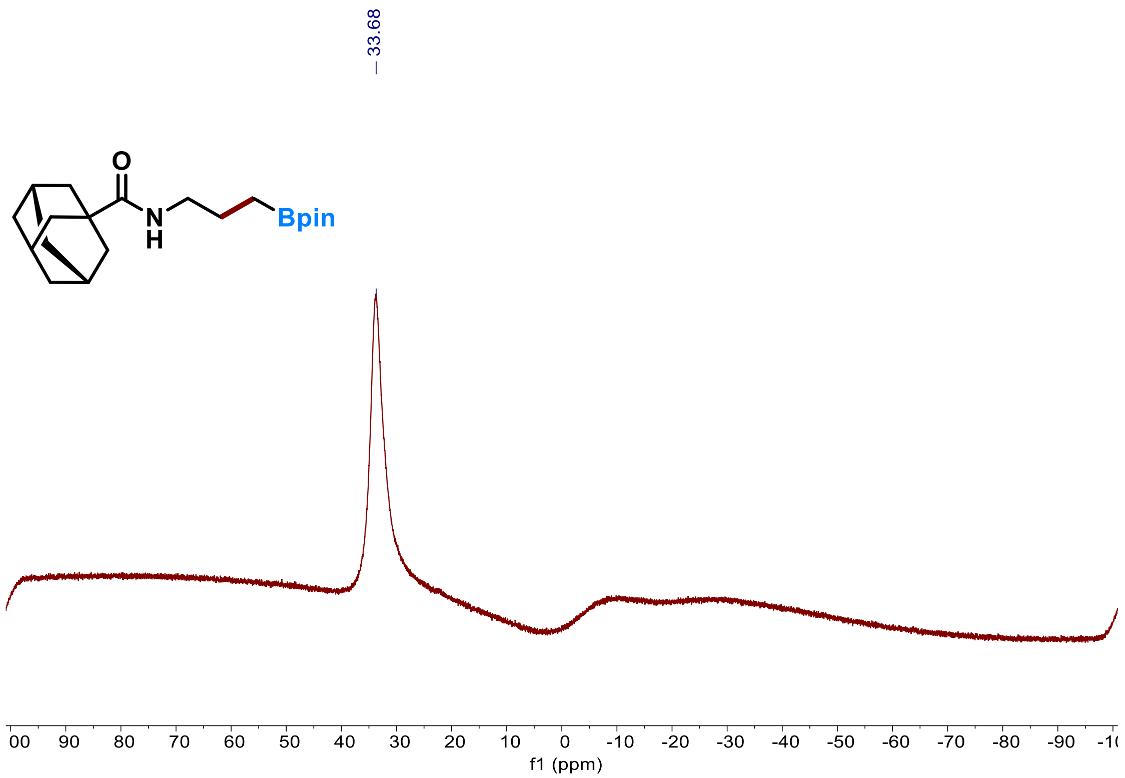

Supplementary Figure 201. ^{11}B NMR spectrum of **2ag** (128 MHz, Chloroform-*d*)

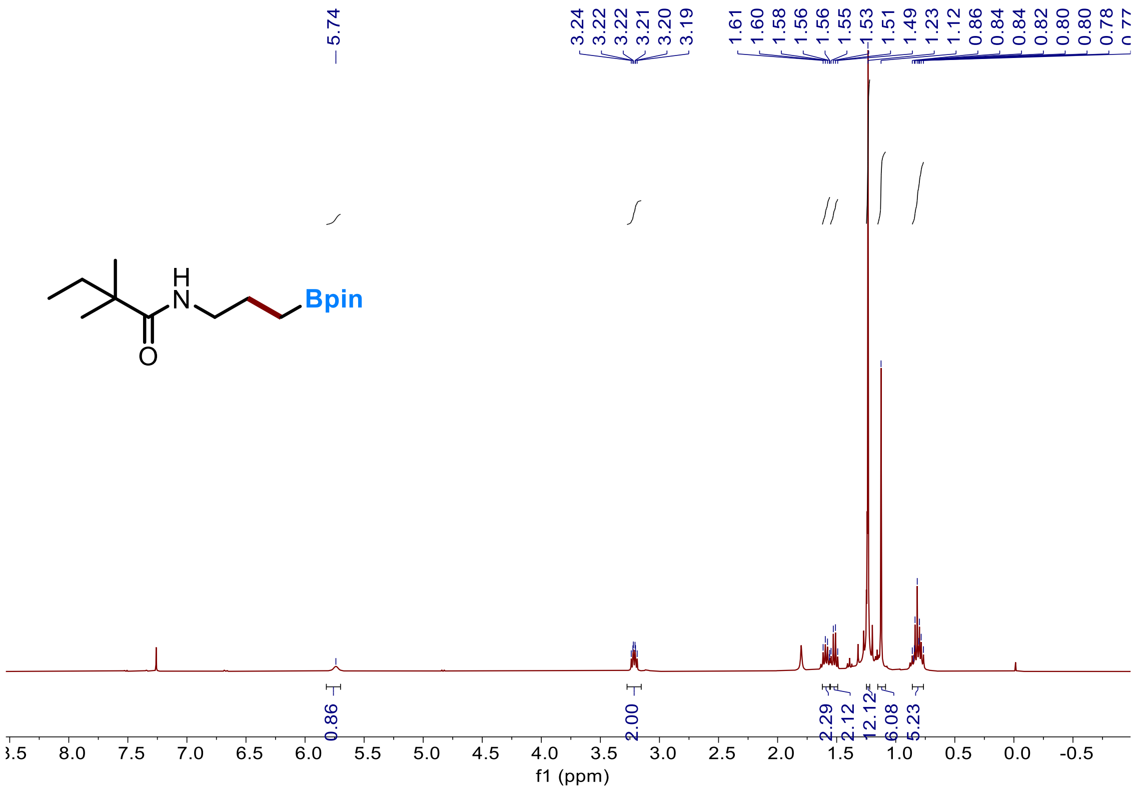

Supplementary Figure 202. ^{1}H NMR spectrum of **2ah** (400 MHz, Chloroform-*d*)

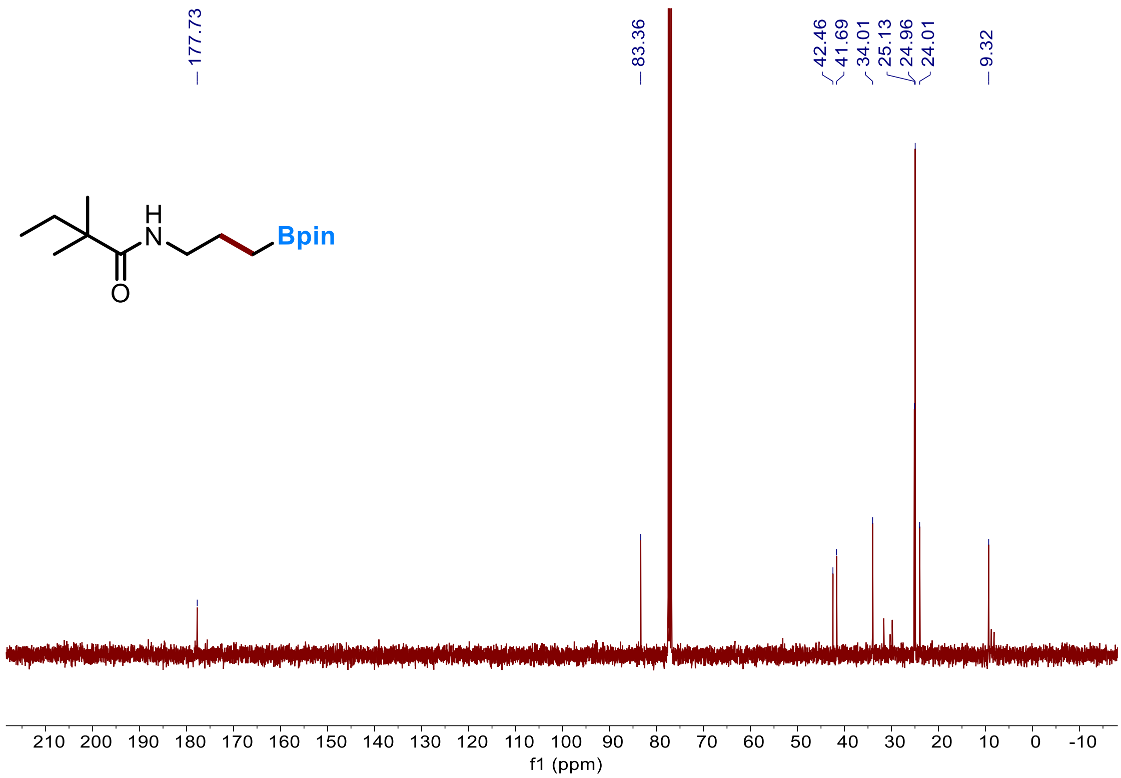

Supplementary Figure 203. $^{13}C\{^{1}H\}$ NMR spectrum of **2ah** (101 MHz, Chloroform-*d*)

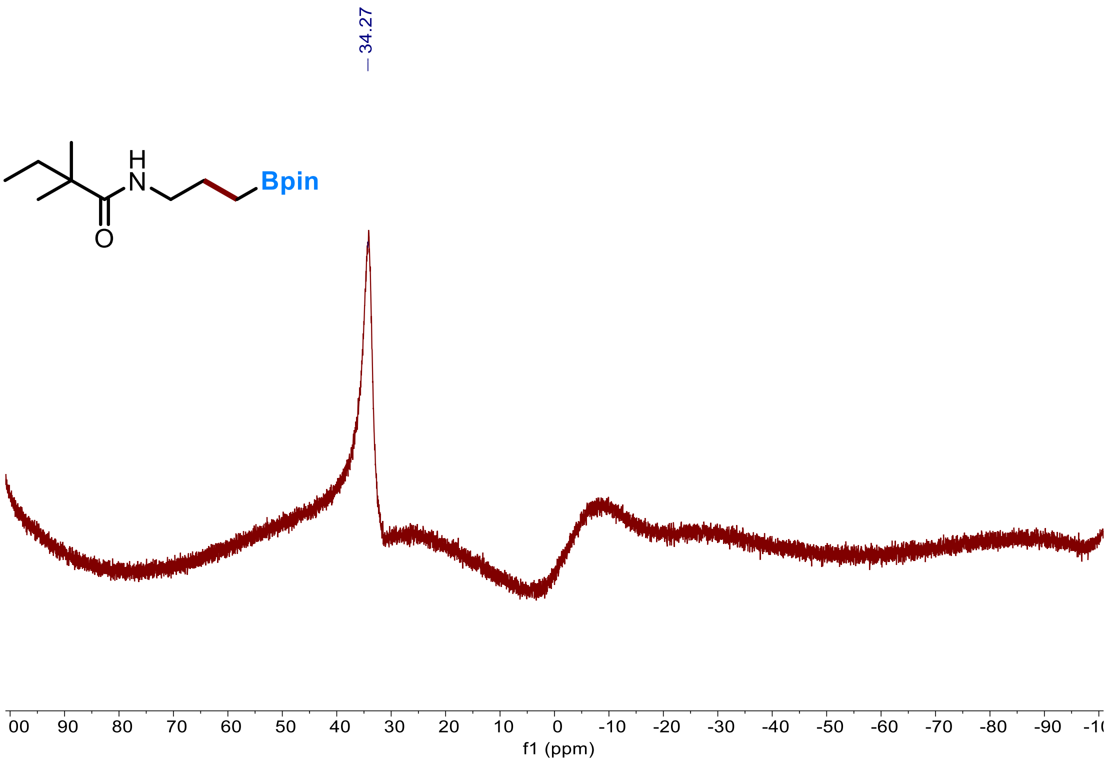

Supplementary Figure 204. ^{11}B NMR spectrum of **2ah** (128 MHz, Chloroform-*d*)

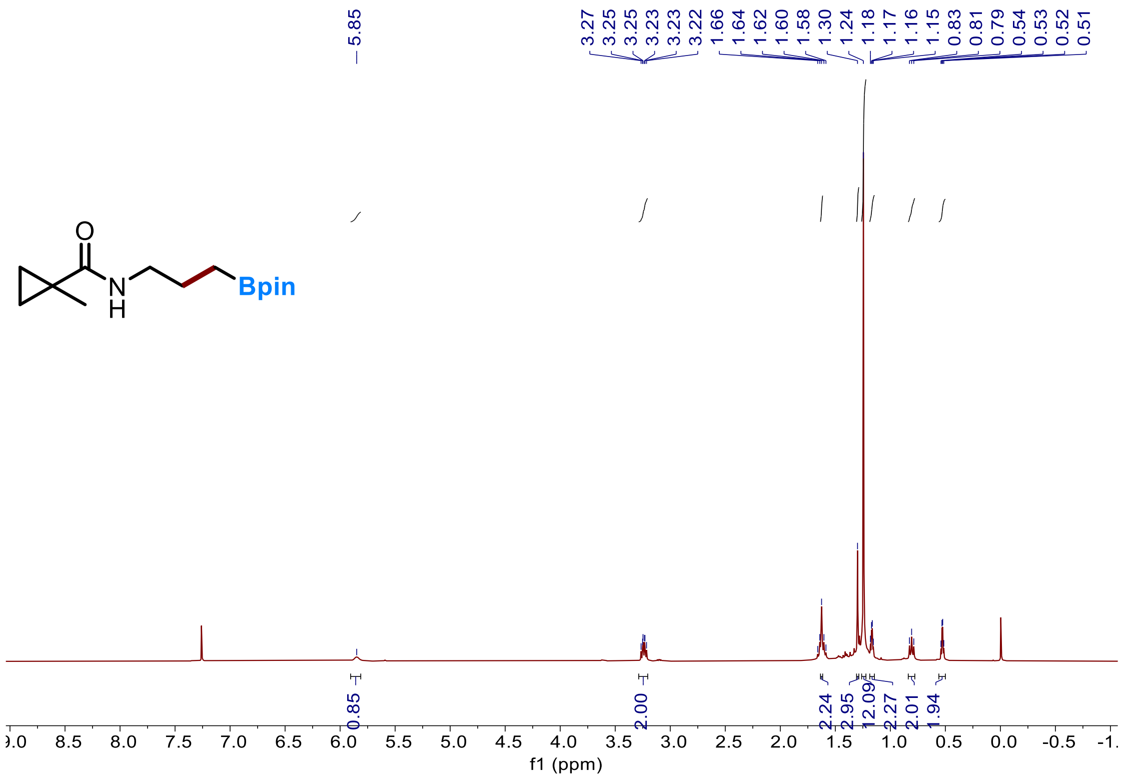

Supplementary Figure 205. ^{1}H NMR spectrum of **2ai** (400 MHz, Chloroform-*d*)

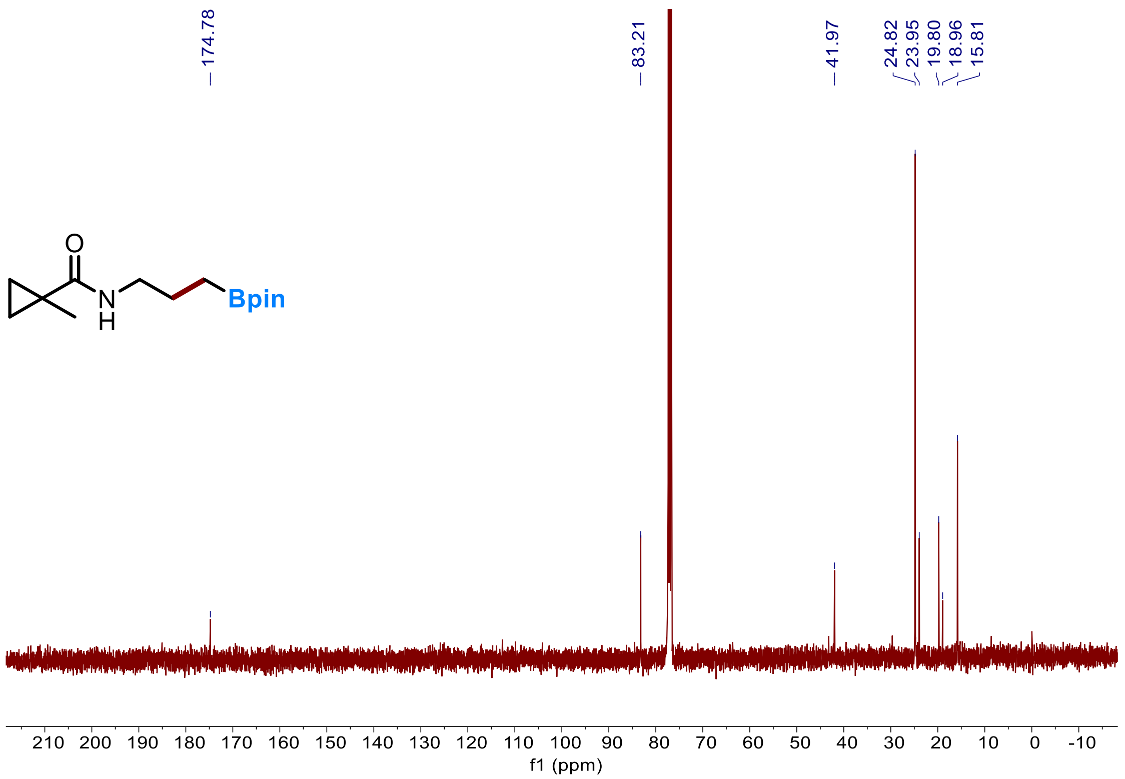

Supplementary Figure 206. ^{13}C{^{1}H} NMR spectrum of **2ai** (101 MHz, Chloroform-*d*)

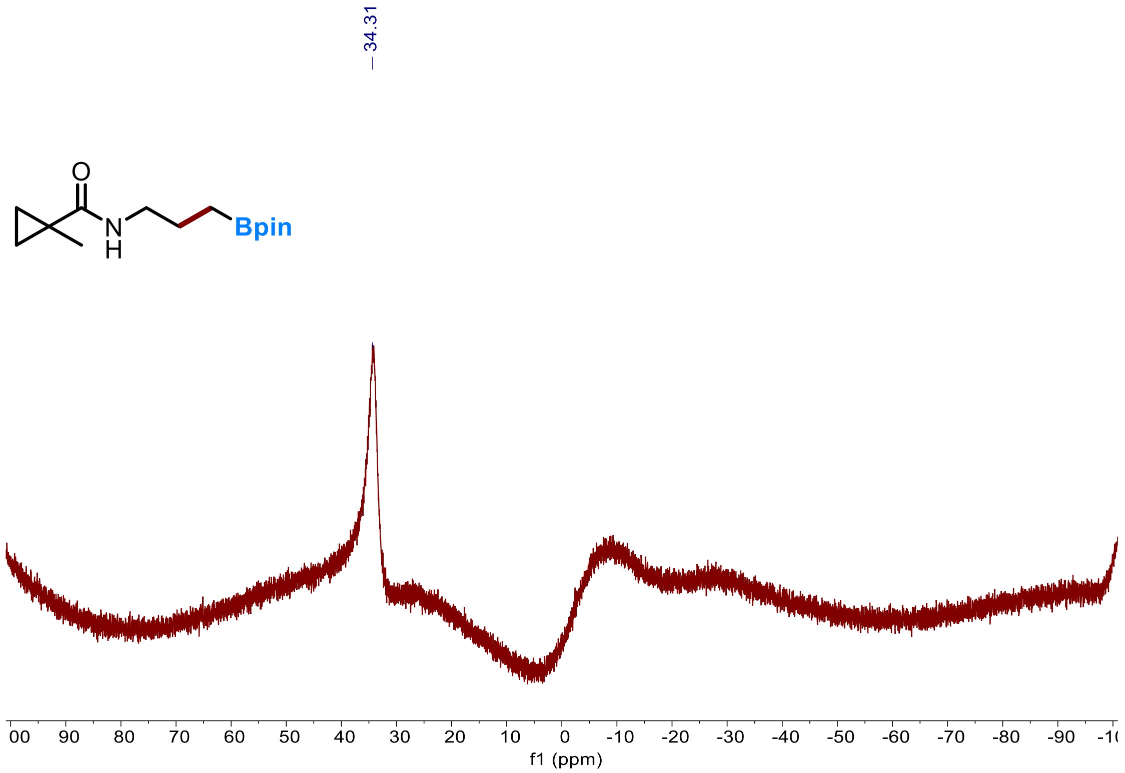

Supplementary Figure 207. ^{11}B NMR spectrum of **2ai** (128 MHz, Chloroform-*d*)

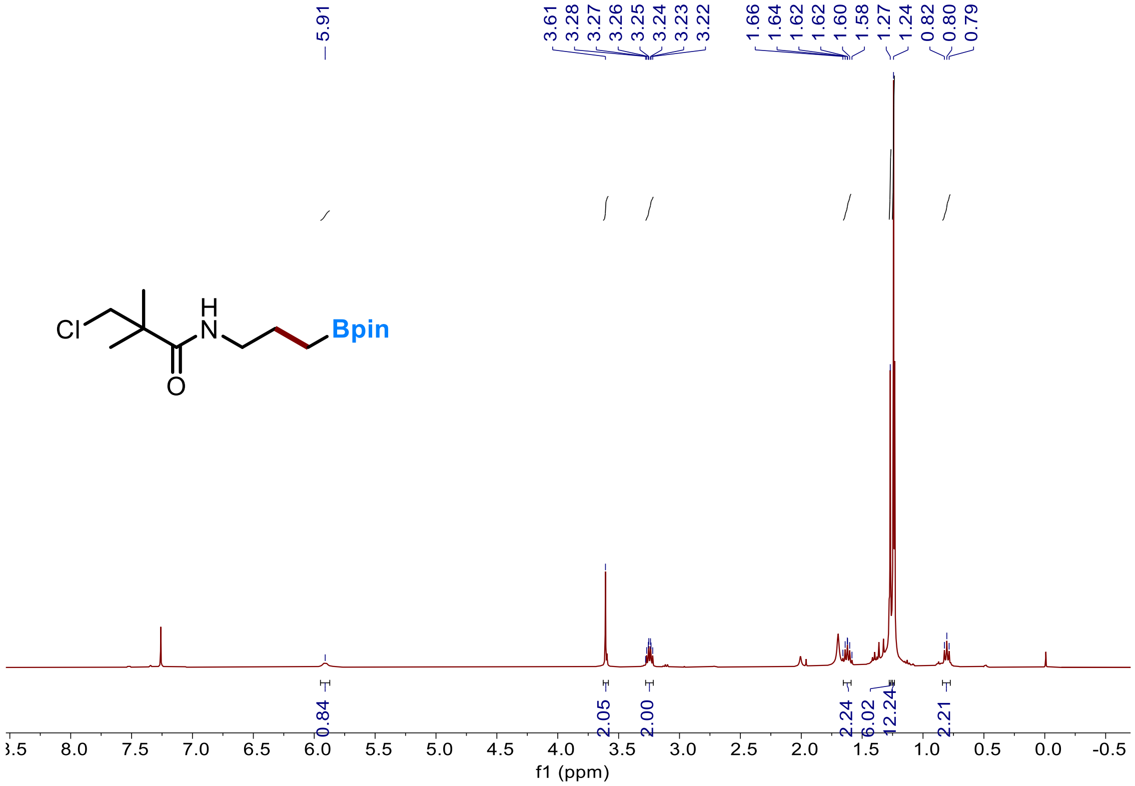

Supplementary Figure 208. ^{1}H NMR spectrum of **2aj** (400 MHz, Chloroform-*d*)

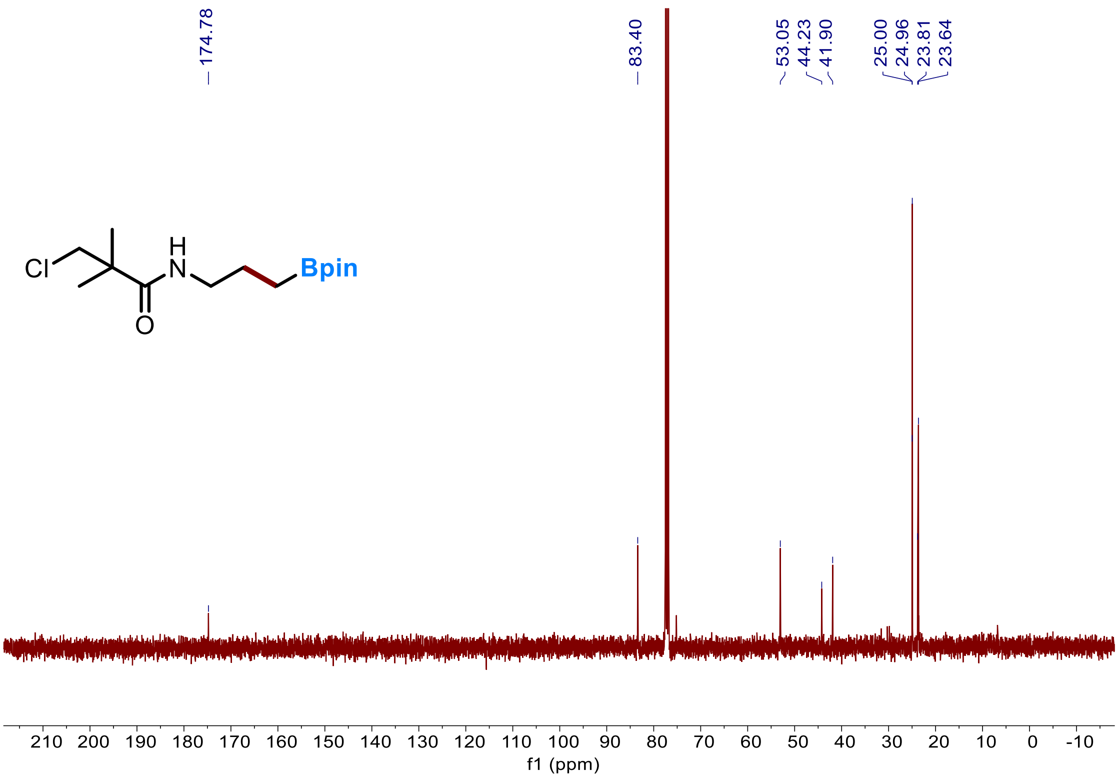

Supplementary Figure 209. $^{13}C\{^{1}H\}$ NMR spectrum of **2aj** (101 MHz, Chloroform-*d*)

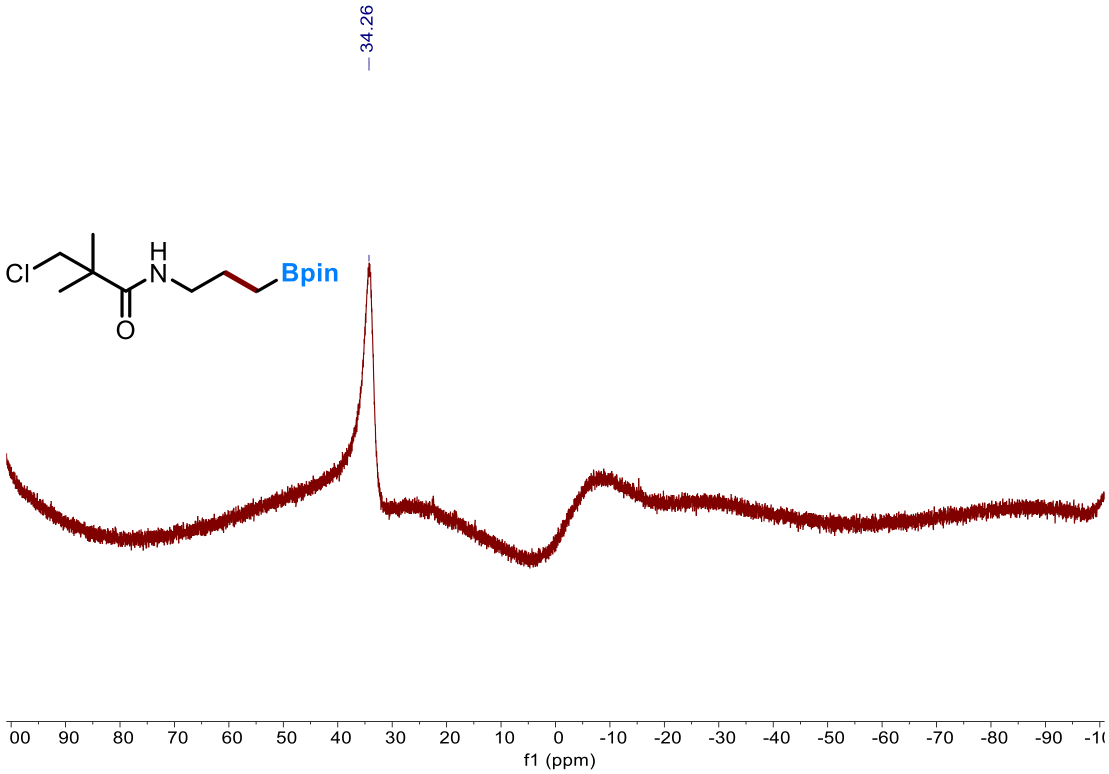

Supplementary Figure 210. ^{11}B NMR spectrum of **2aj** (128 MHz, Chloroform-*d*)

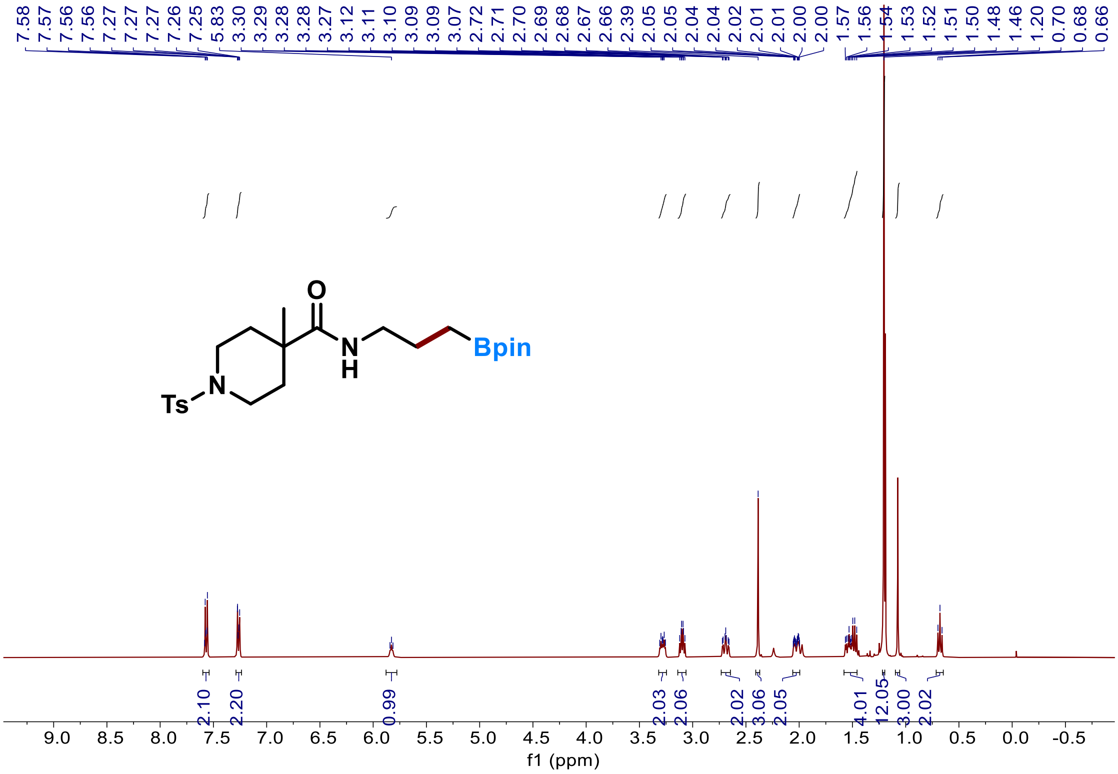

Supplementary Figure 211. ^{1}H NMR spectrum of **2ak** (400 MHz, Chloroform-*d*)

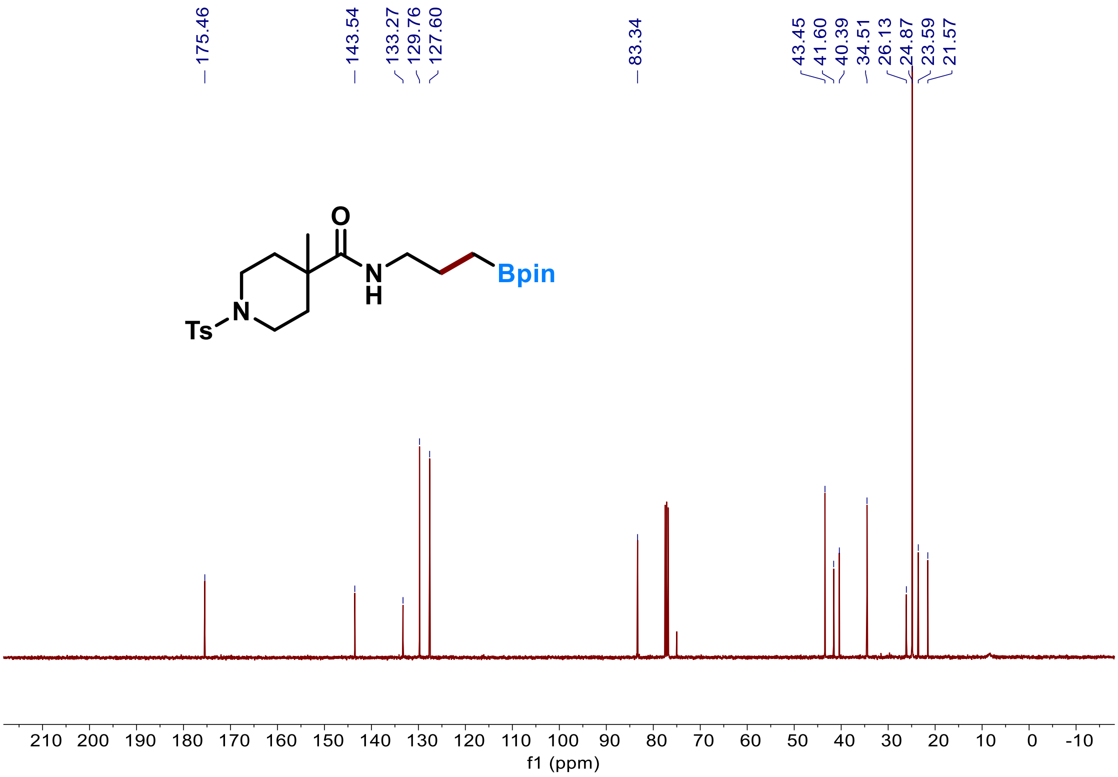

Supplementary Figure 212. $^{13}C\{^{1}H\}$ NMR spectrum of **2ak** (101 MHz, Chloroform-*d*)

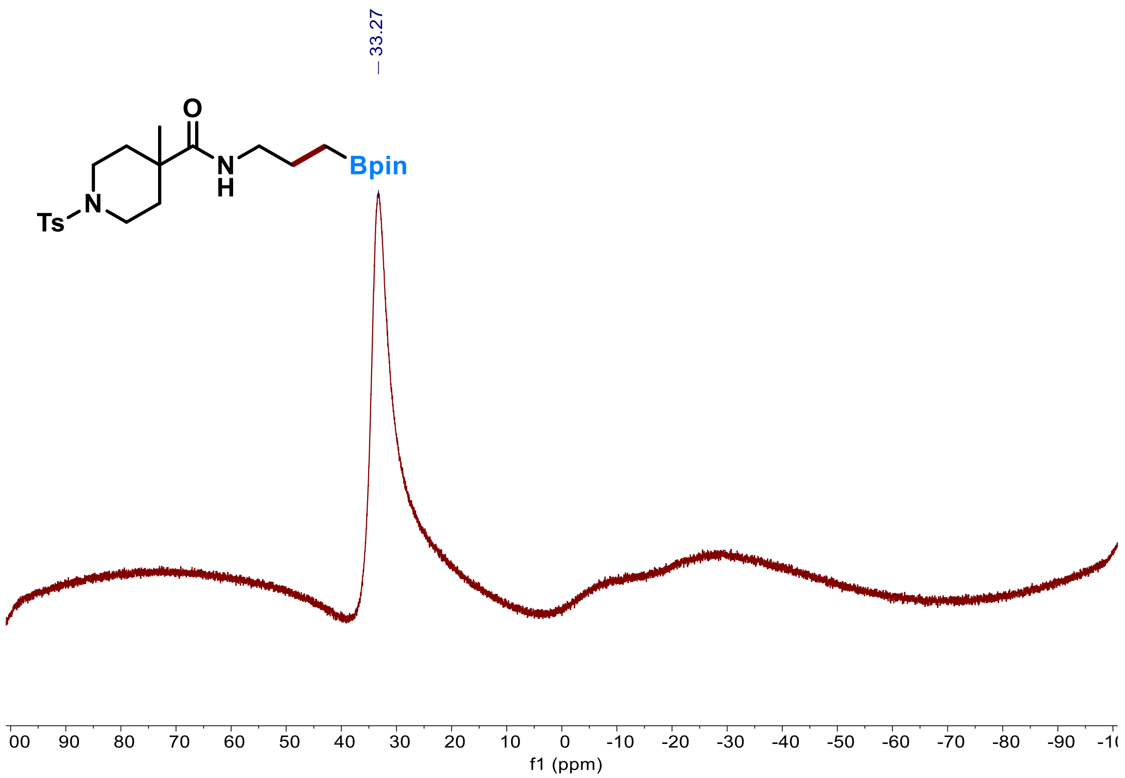

Supplementary Figure 213. ^{11}B NMR spectrum of **2ak** (128 MHz, Chloroform-*d*)

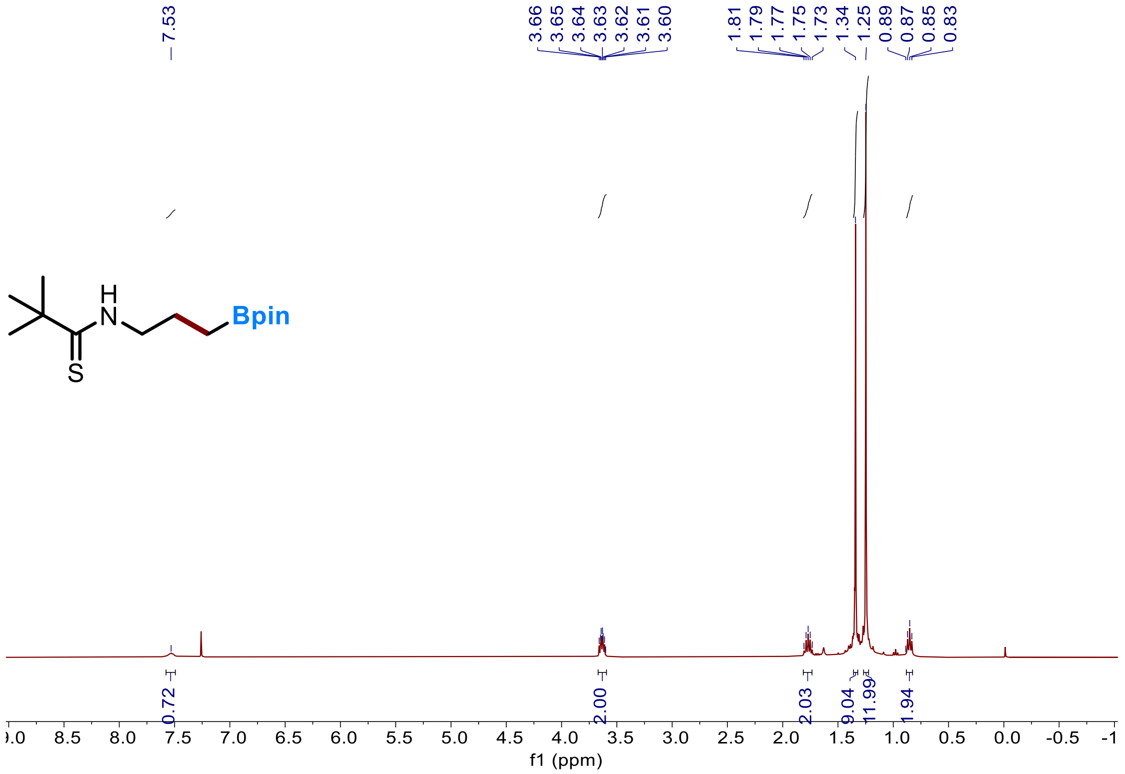

Supplementary Figure 214. ^{1}H NMR spectrum of **2al** (400 MHz, Chloroform-*d*)

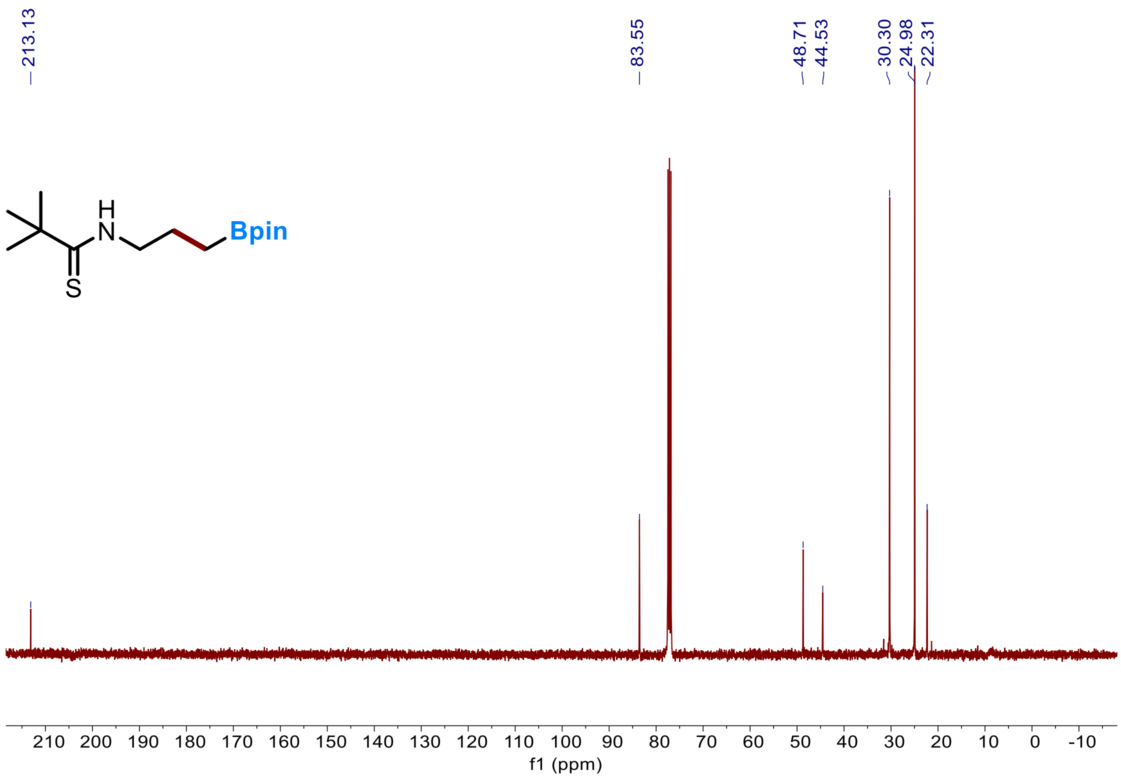

Supplementary Figure 215. $^{13}C\{^{1}H\}$ NMR spectrum of **2al** (101 MHz, Chloroform-*d*)

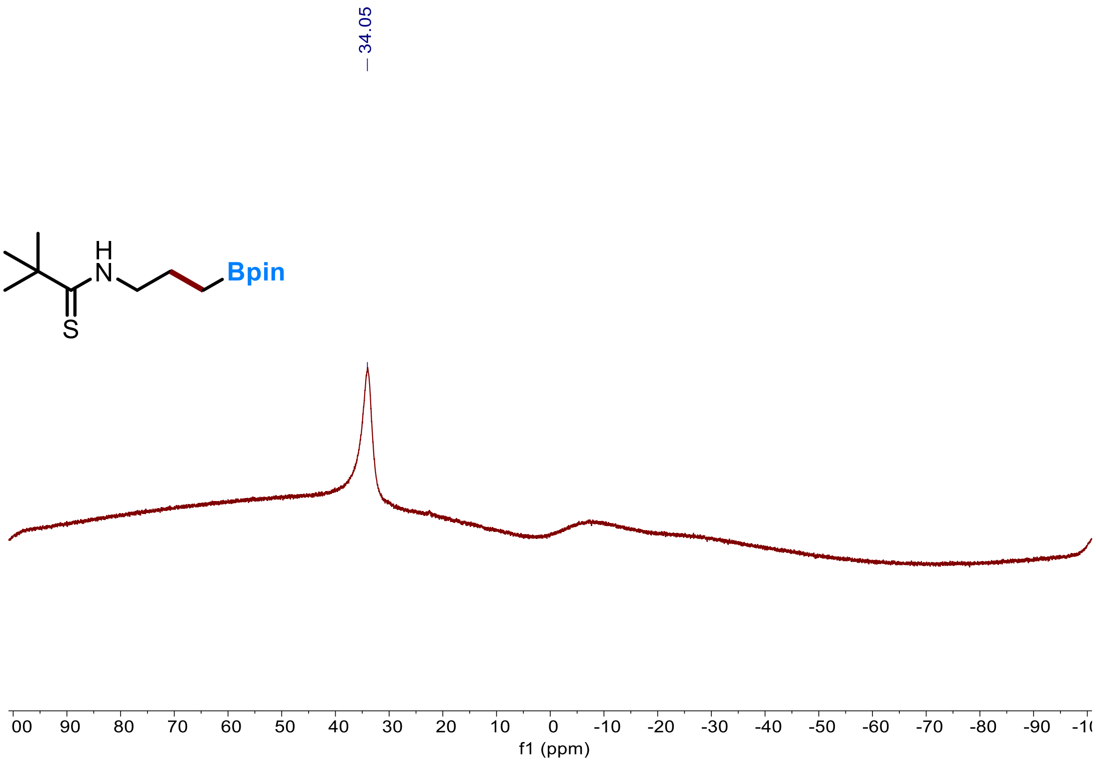

Supplementary Figure 216. ^{11}B NMR spectrum of **2al** (128 MHz, Chloroform-*d*)

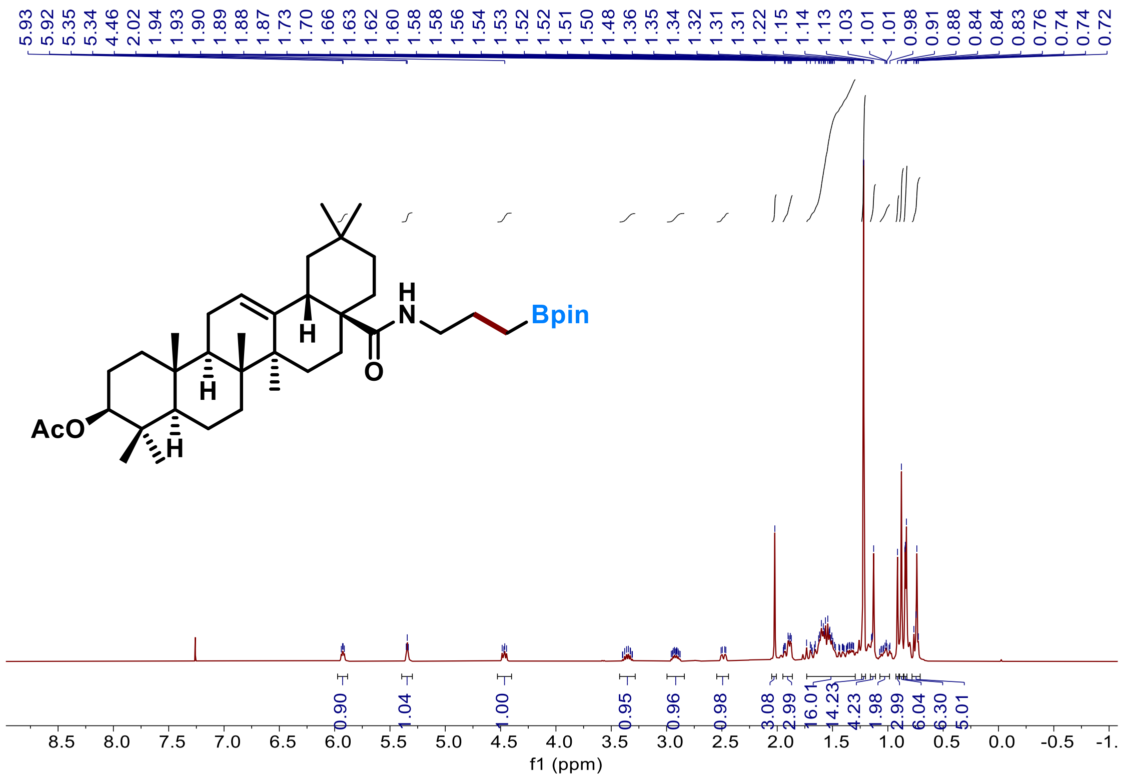

Supplementary Figure 217. ^{1}H NMR spectrum of **2am** (400 MHz, Chloroform-*d*)

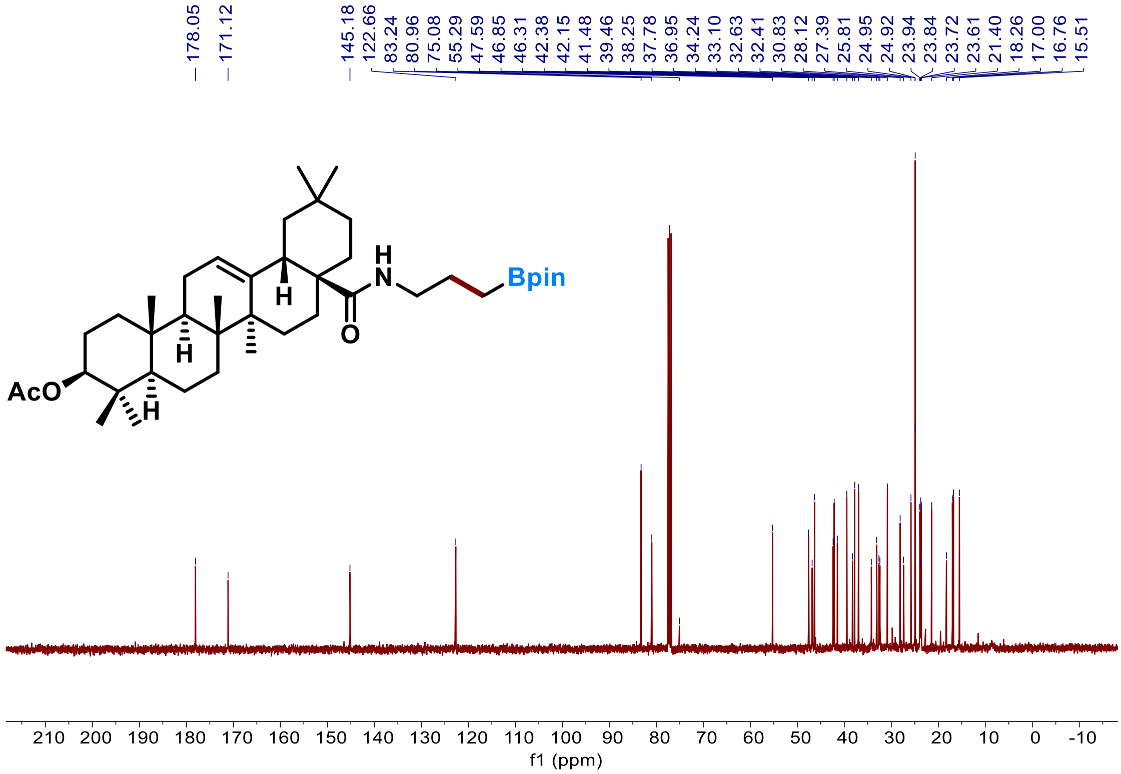

Supplementary Figure 218. ^{13}C{^{1}H} NMR spectrum of **2am** (101 MHz, Chloroform-*d*)

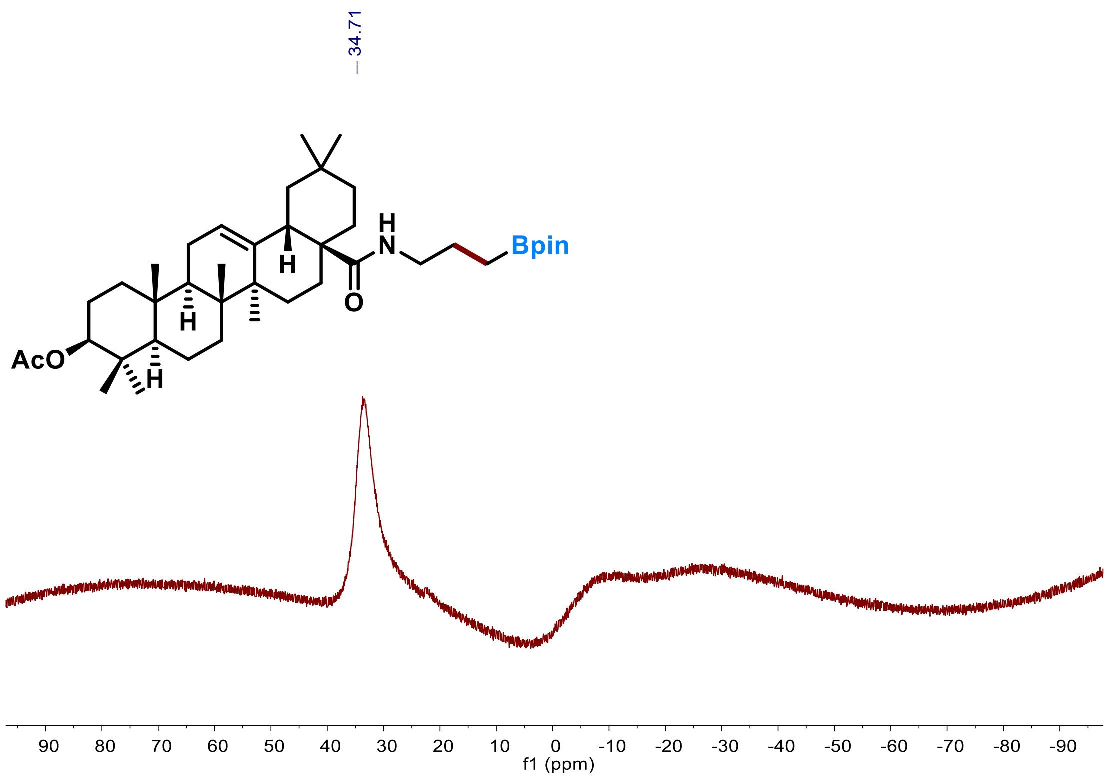

Supplementary Figure 219. ^{11}B NMR spectrum of **2an** (128 MHz, Chloroform-*d*)

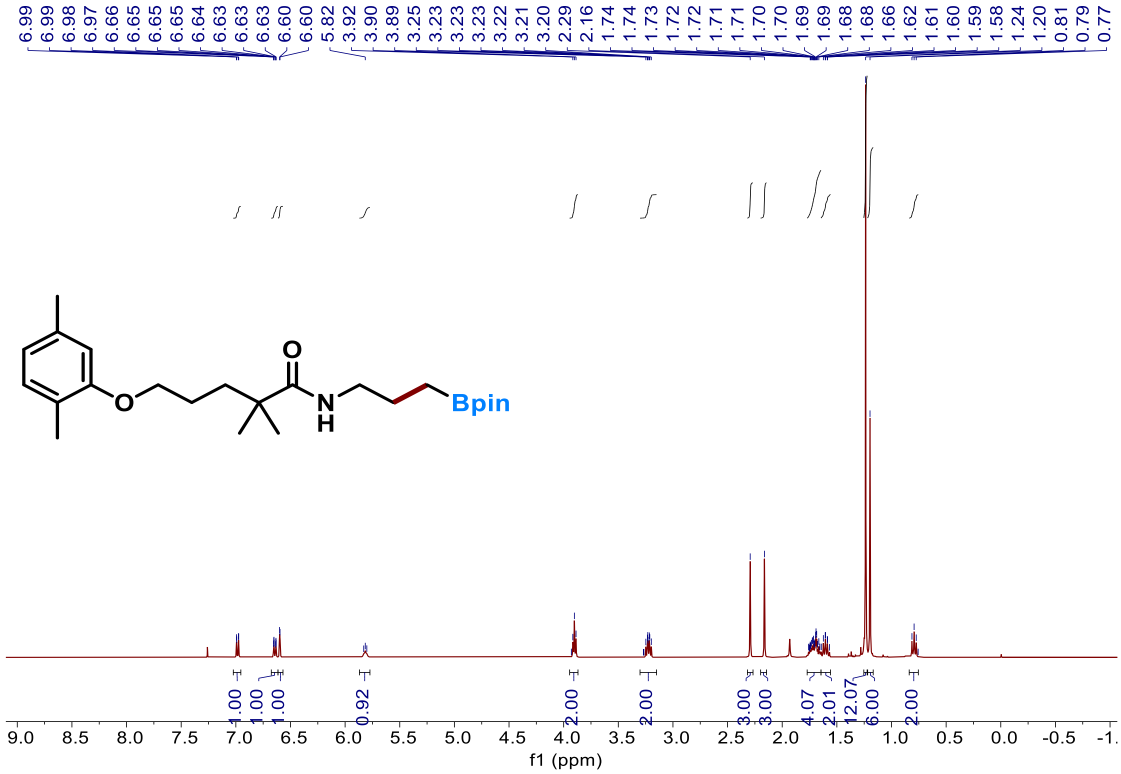

Supplementary Figure 220. ^{1}H NMR spectrum of **2an** (400 MHz, Chloroform-*d*)

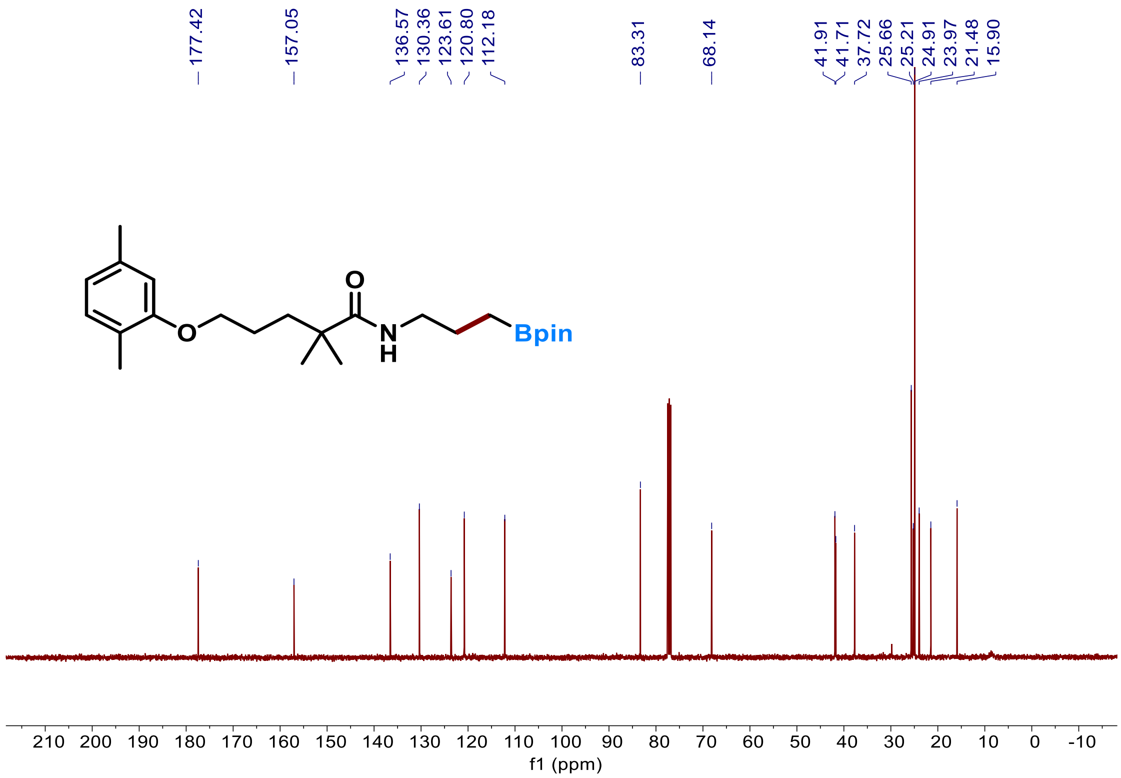

Supplementary Figure 221. $^{13}C\{^{1}H\}$ NMR spectrum of **2an** (101 MHz, Chloroform-*d*)

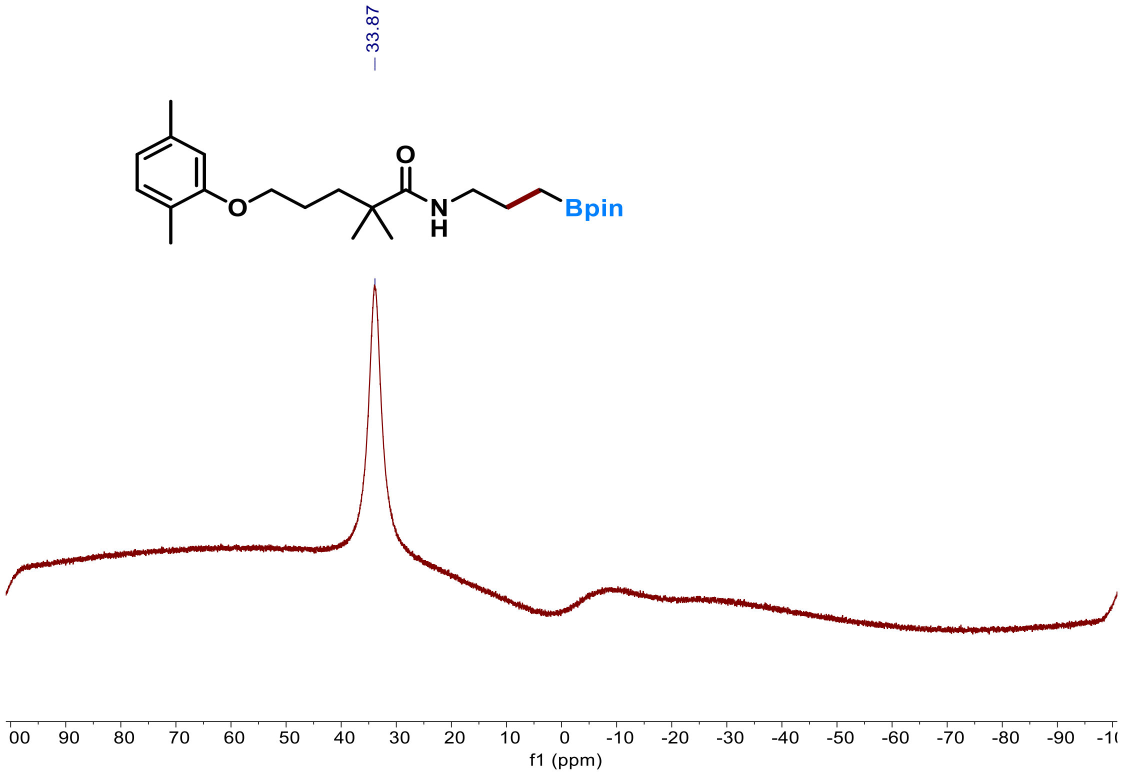

Supplementary Figure 222. ^{11}B NMR spectrum of **2an** (128 MHz, Chloroform-*d*)

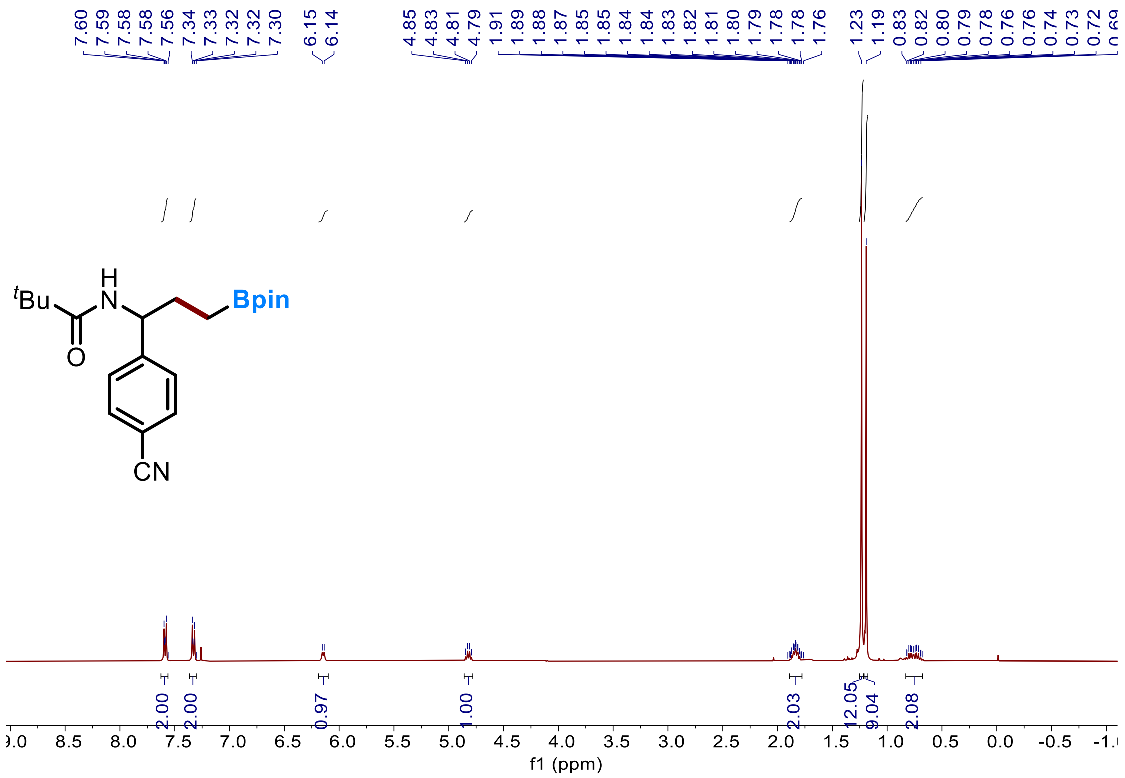

Supplementary Figure 223. ^{1}H NMR spectrum of **2ao** (400 MHz, Chloroform-*d*)

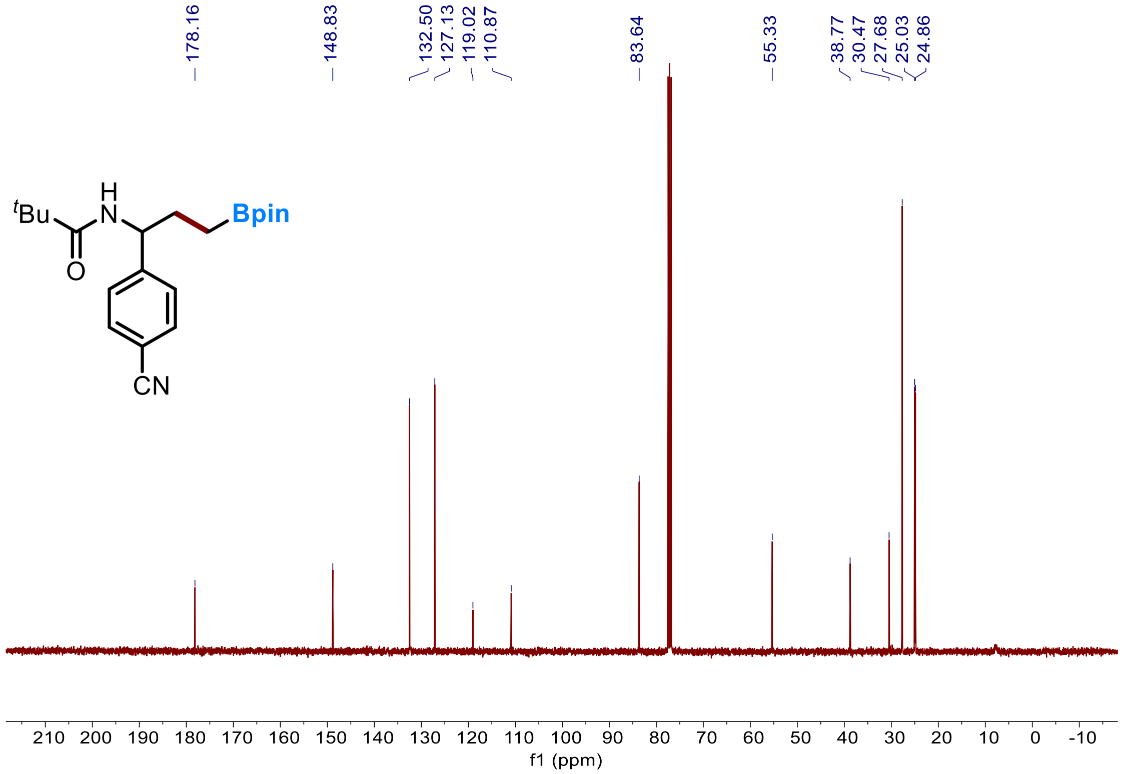

Supplementary Figure 224. $^{13}C\{^{1}H\}$ NMR spectrum of **2ao** (101 MHz, Chloroform-*d*)

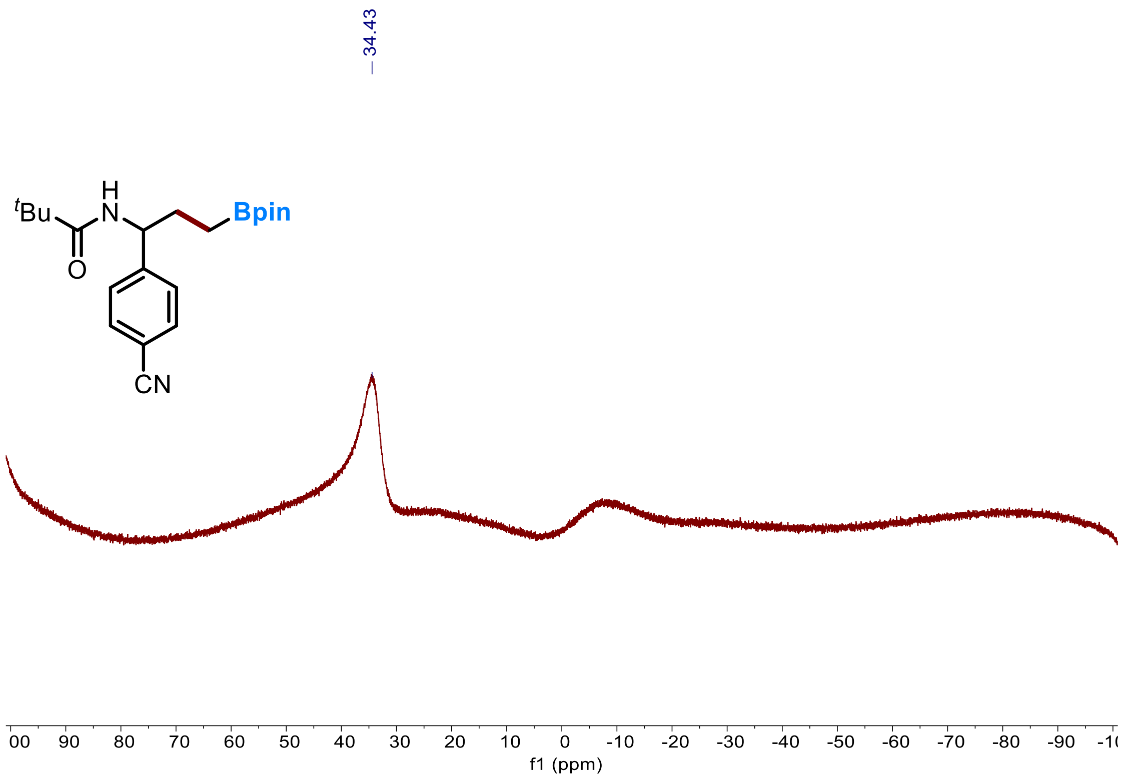

Supplementary Figure 225. ^{11}B NMR spectrum of **2ao** (128 MHz, Chloroform-*d*)

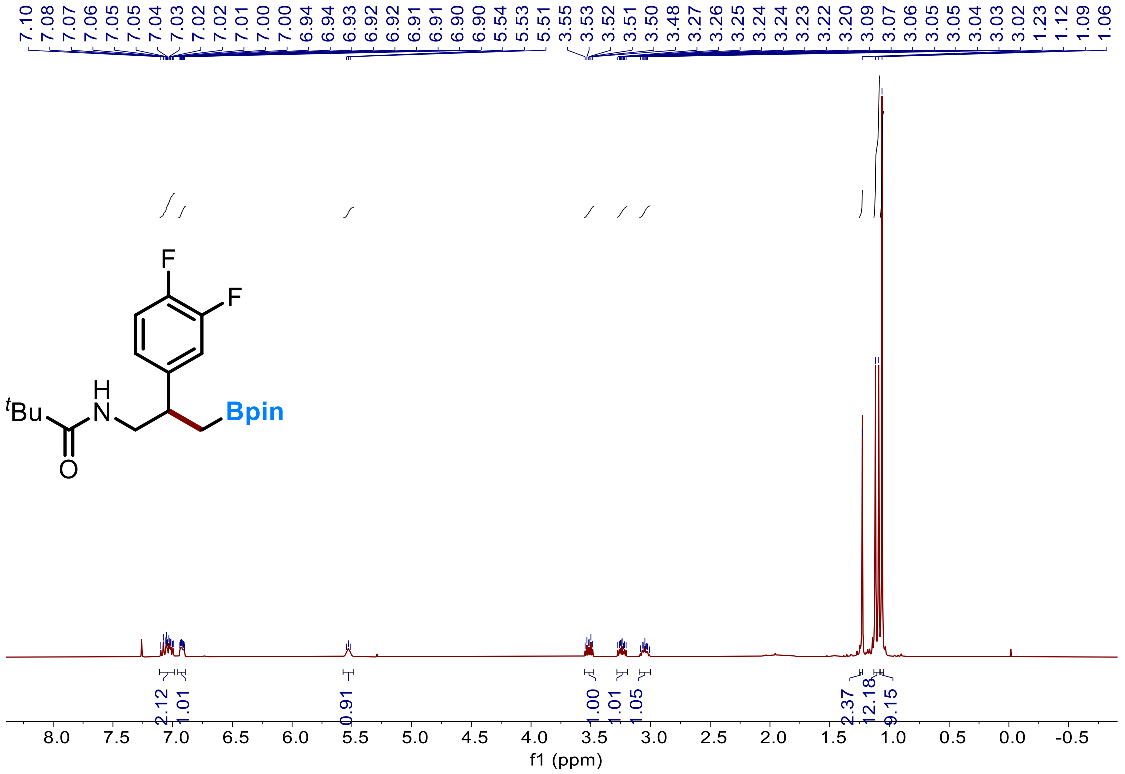

Supplementary Figure 226. ^{1}H NMR spectrum of **2ap** (400 MHz, Chloroform-*d*)

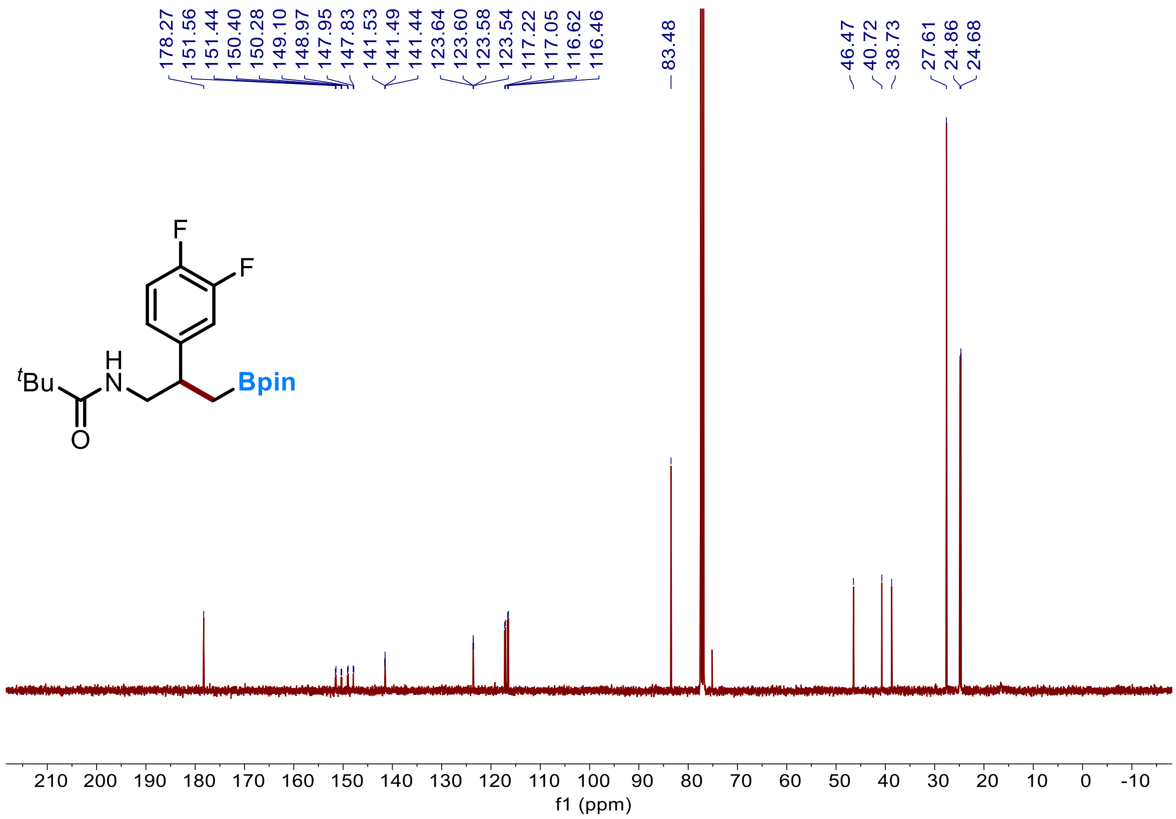

Supplementary Figure 227. $^{13}C\{^{1}H\}$ NMR spectrum of **2ap** (101 MHz, Chloroform-*d*)

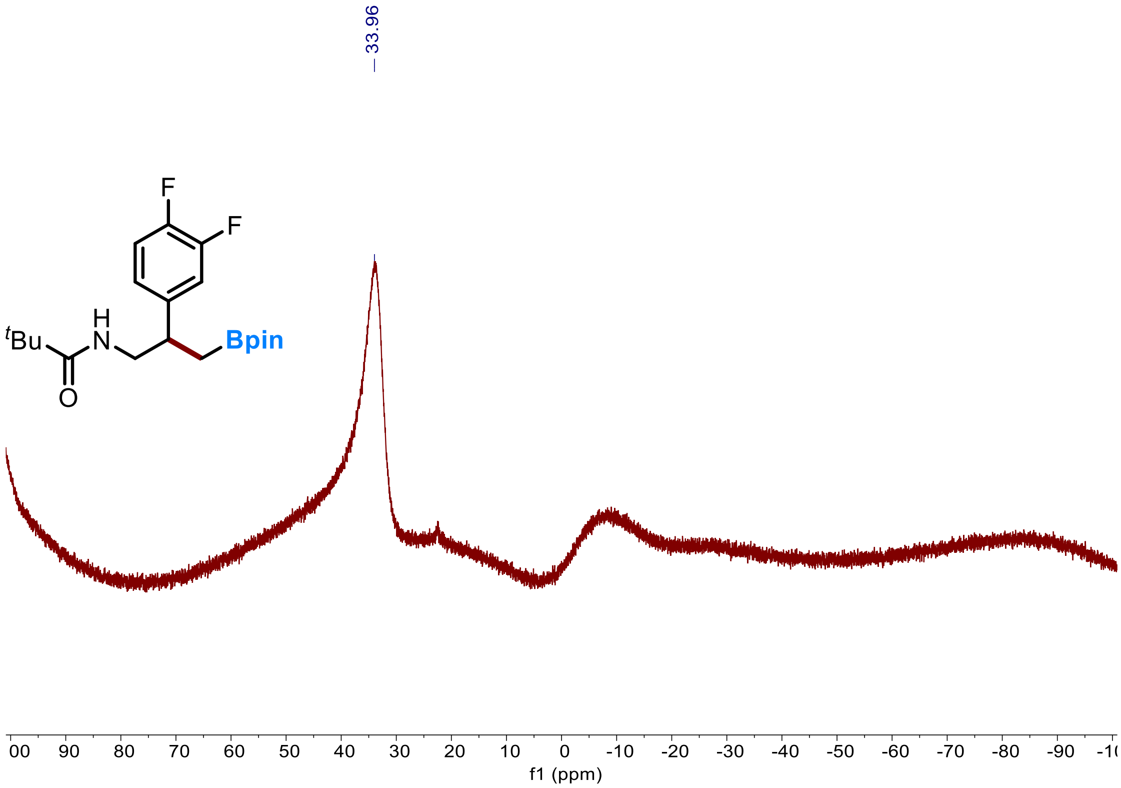

Supplementary Figure 228. ^{11}B NMR spectrum of **2ap** (128 MHz, Chloroform-*d*)

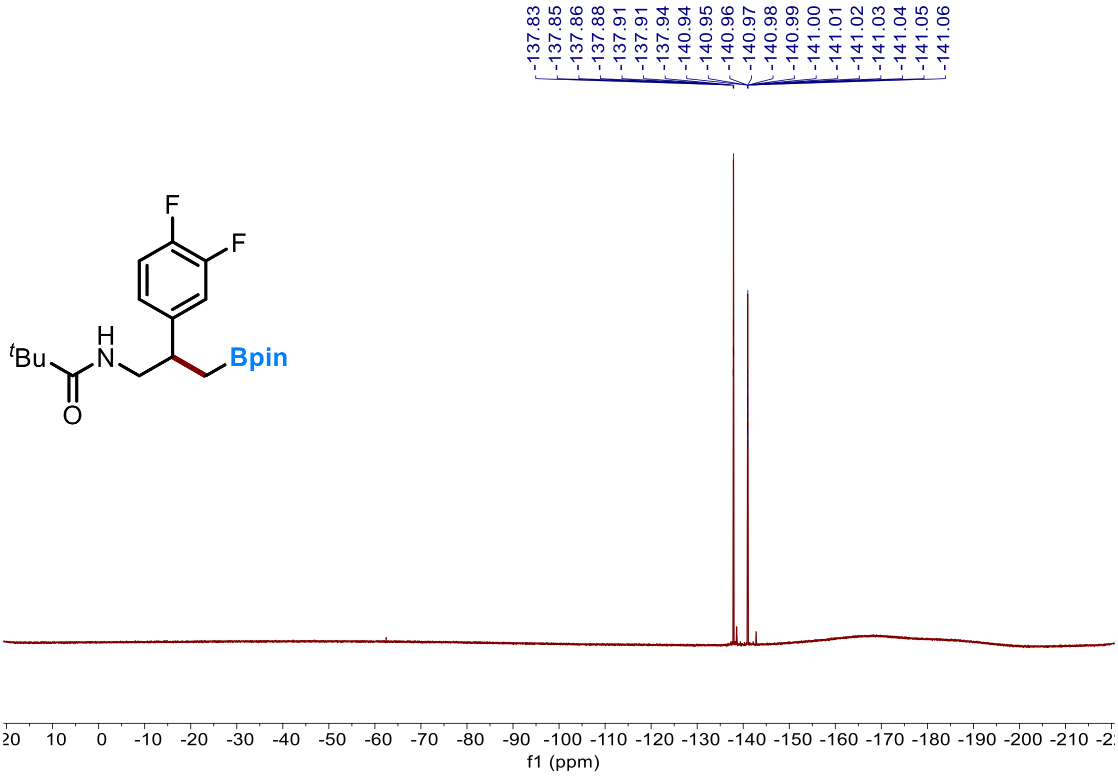

Supplementary Figure 229. ^{19}F NMR spectrum of **2ap** (376 MHz, Chloroform-*d*)

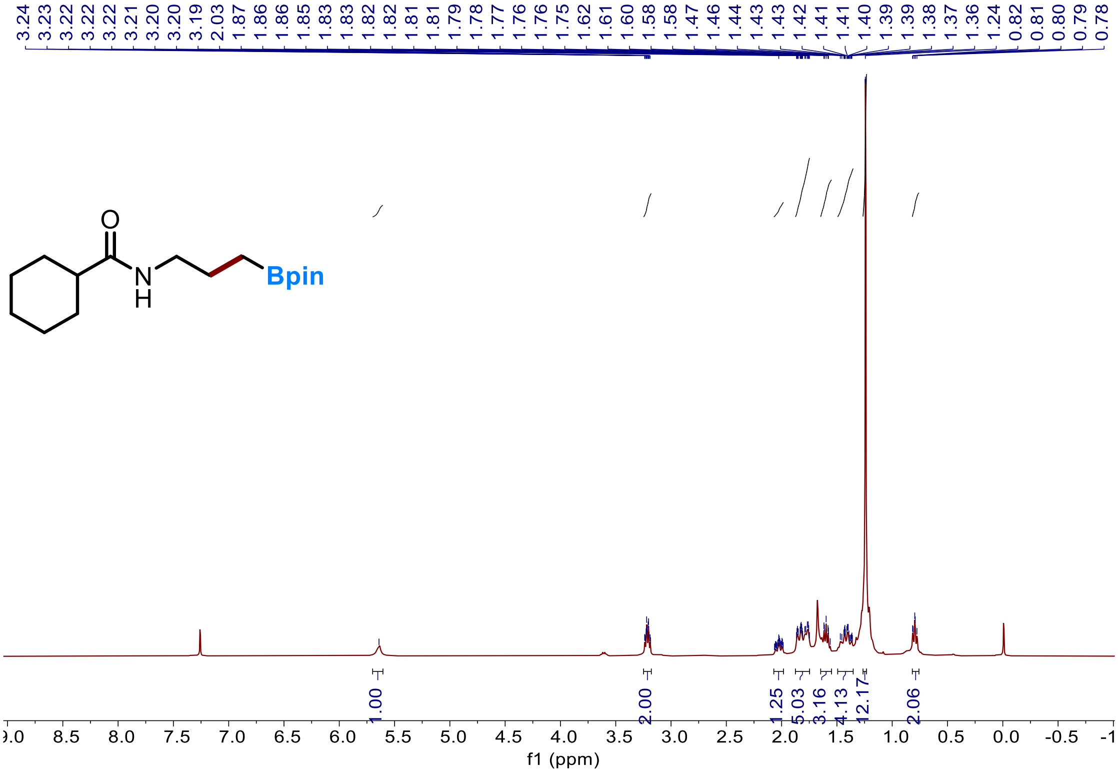

Supplementary Figure 230. ^{1}H NMR spectrum of **2aq** (400 MHz, Chloroform-*d*)

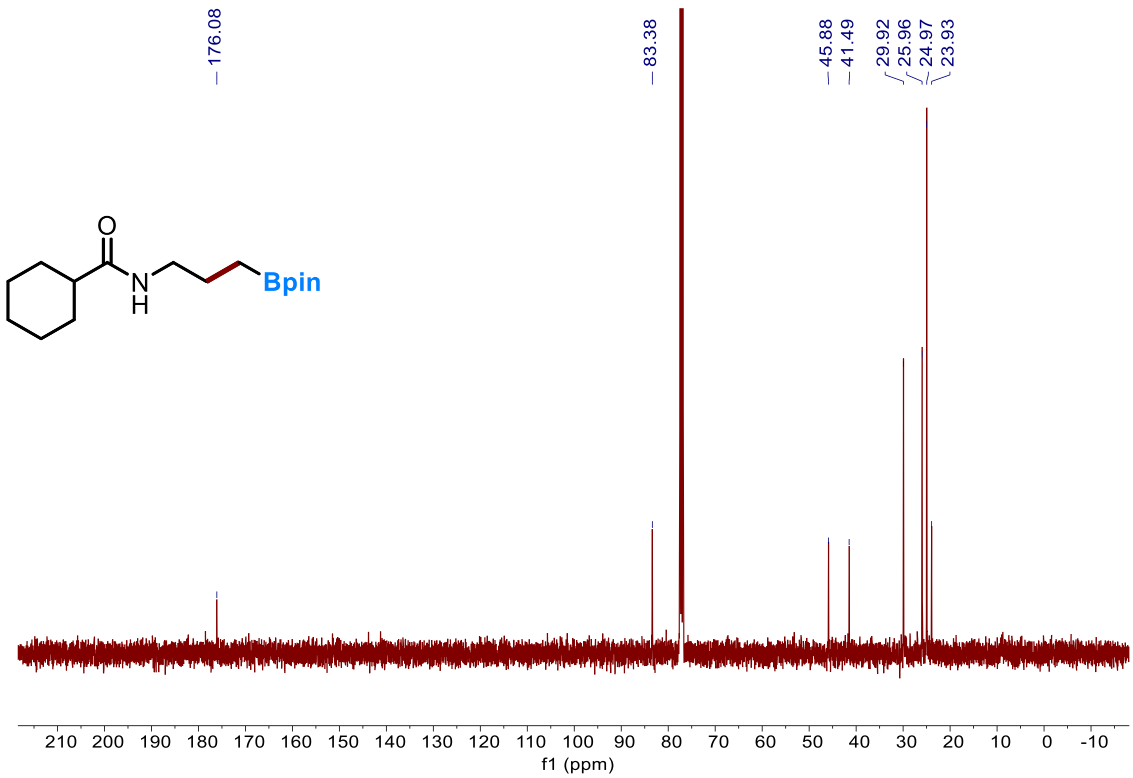

Supplementary Figure 231. $^{13}C\{^{1}H\}$ NMR spectrum of **2aq** (101 MHz, Chloroform-*d*)

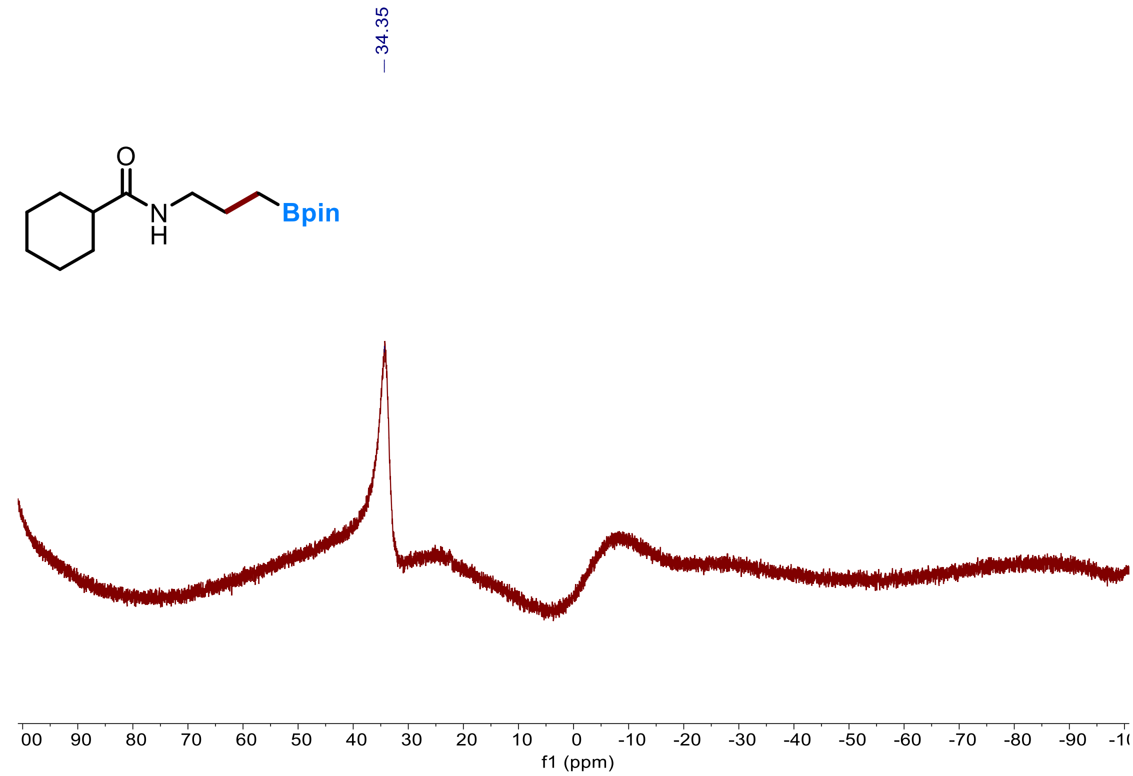

Supplementary Figure 232. ^{11}B NMR spectrum of **2aq** (128 MHz, Chloroform-*d*)

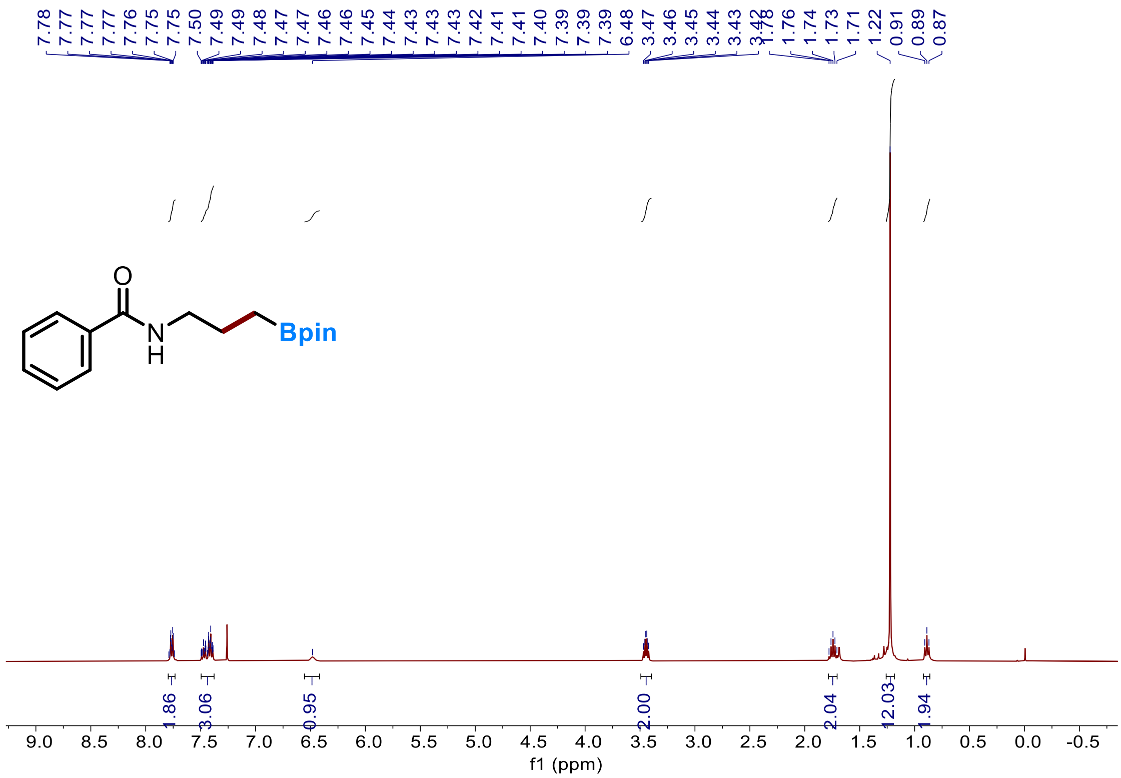

Supplementary Figure 233. ^{1}H NMR spectrum of **2ar** (400 MHz, Chloroform-*d*)

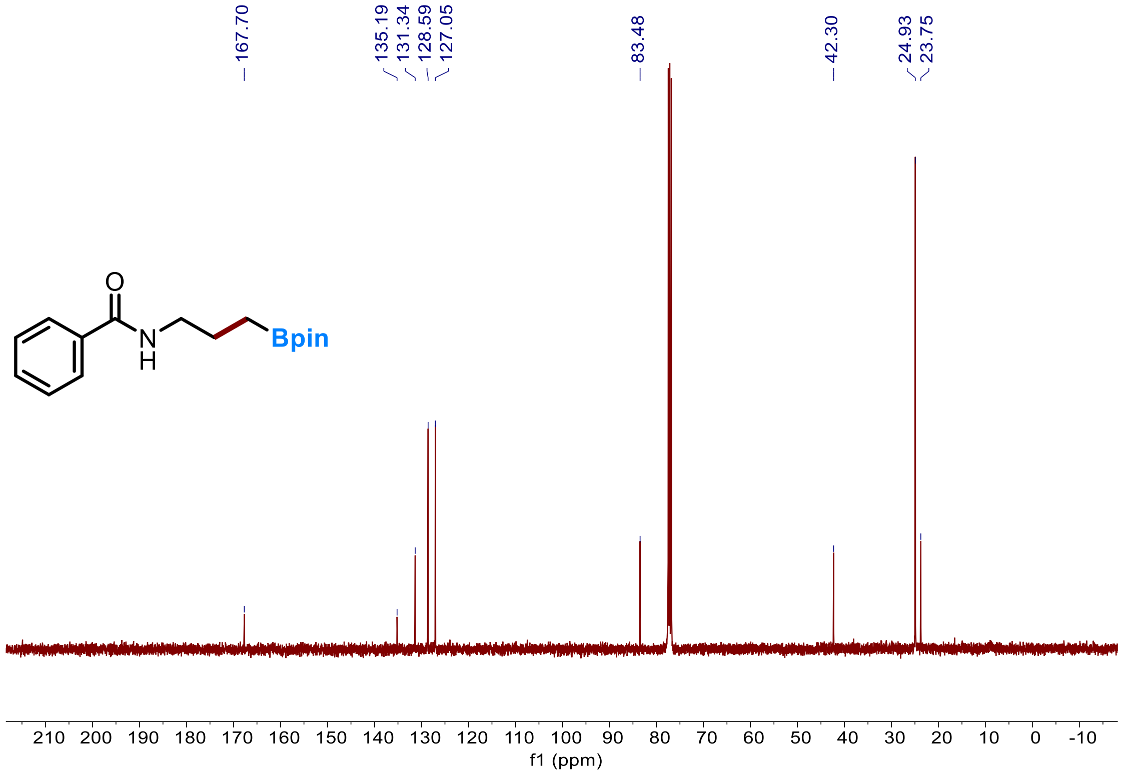

Supplementary Figure 234. ^{13}C{^{1}H} NMR spectrum of **2ar** (101 MHz, Chloroform-*d*)

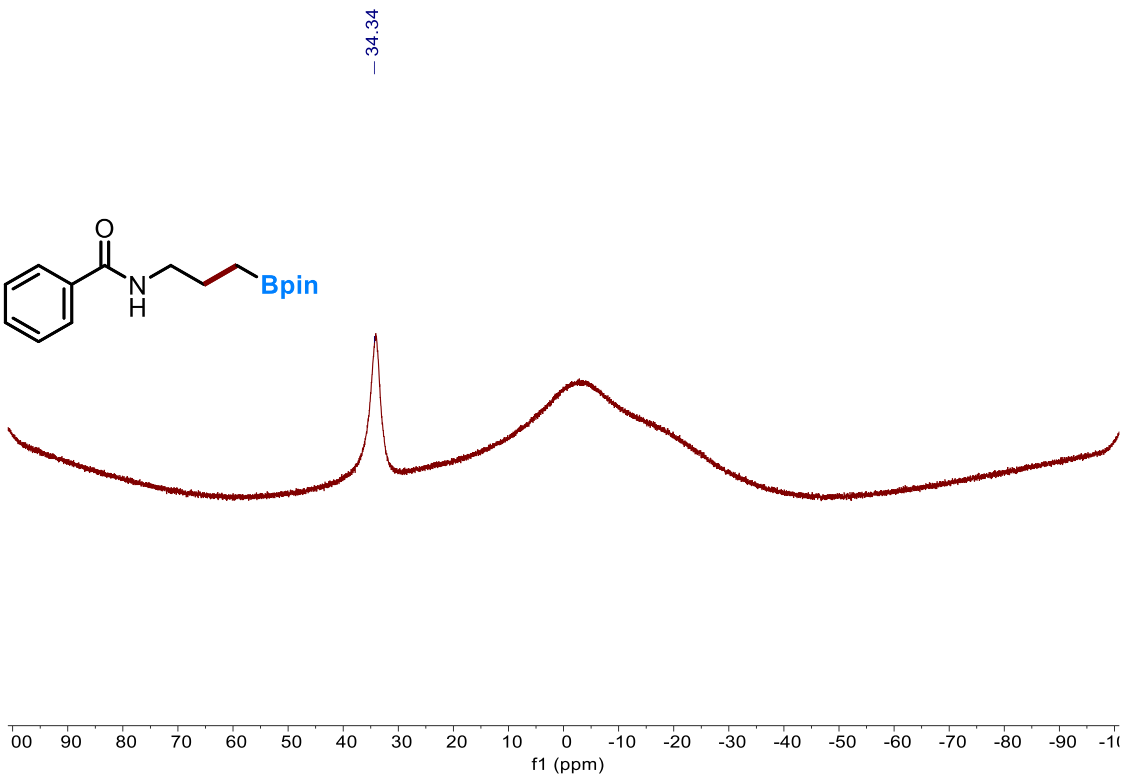

Supplementary Figure 235. ^{11}B NMR spectrum of **2ar** (128 MHz, Chloroform-*d*)

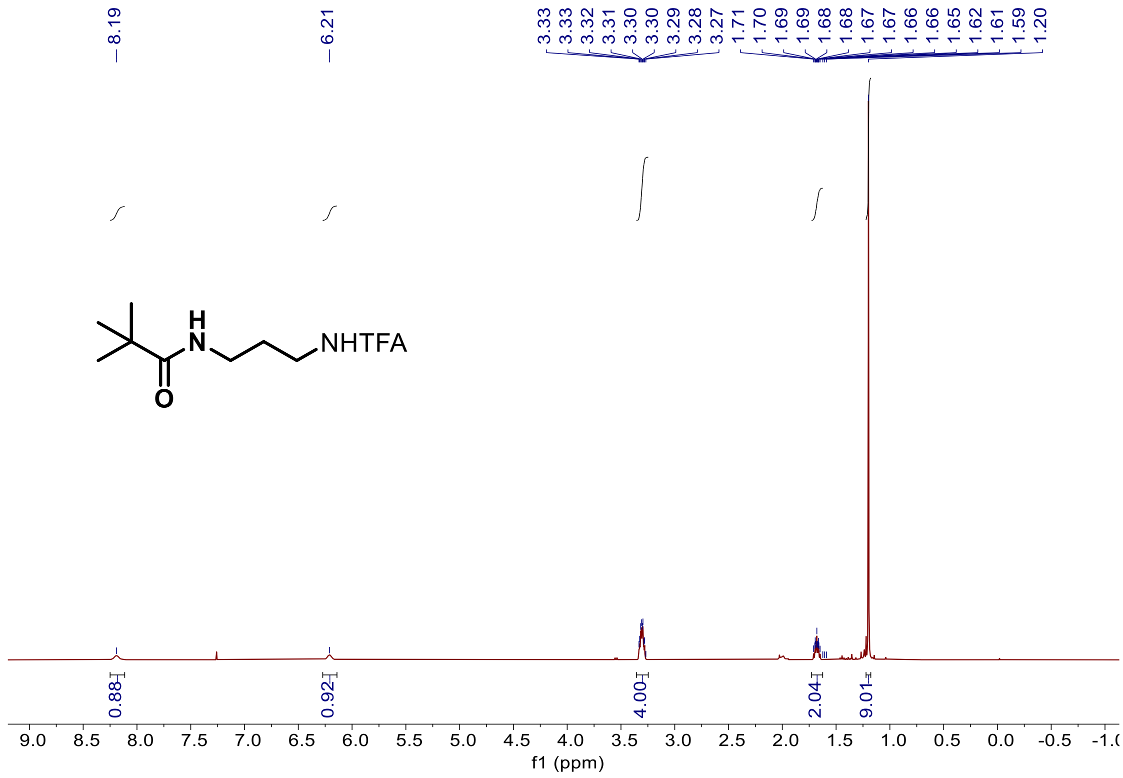

Supplementary Figure 236. ^{1}H NMR spectrum of **3a** (400 MHz, Chloroform-*d*)

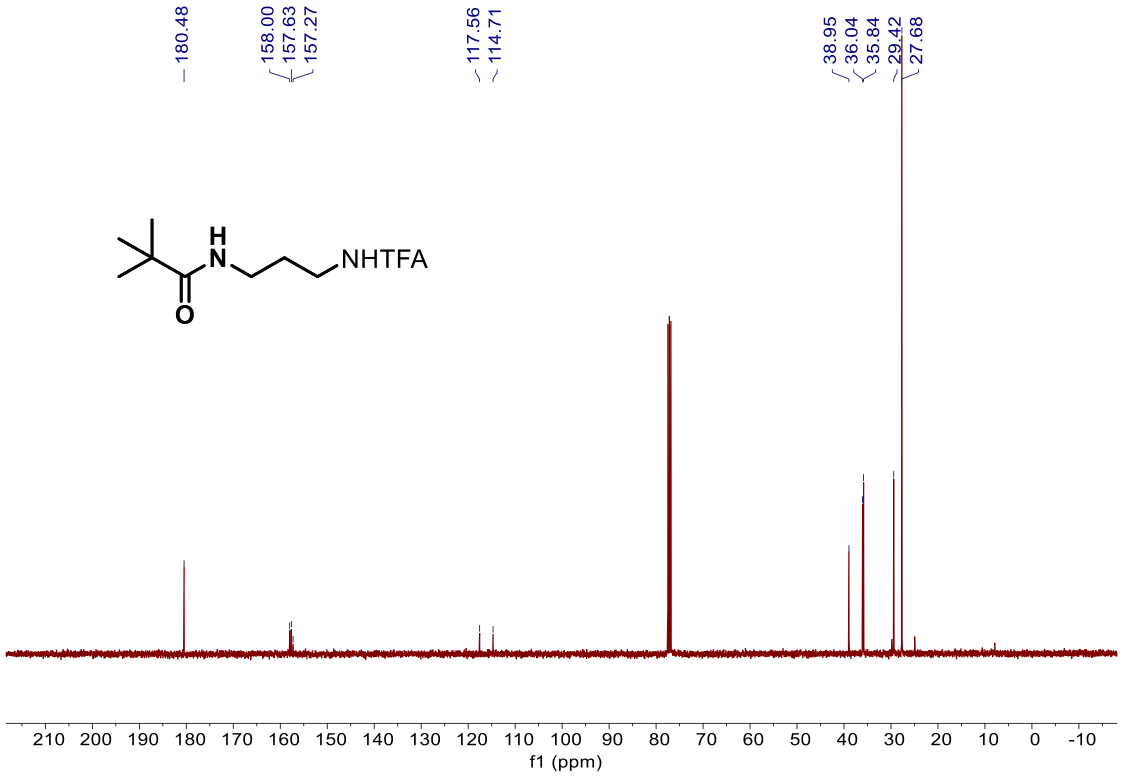

Supplementary Figure 237. $^{13}C\{^1H\}$ NMR spectrum of **3a** (101 MHz, Chloroform-*d*)

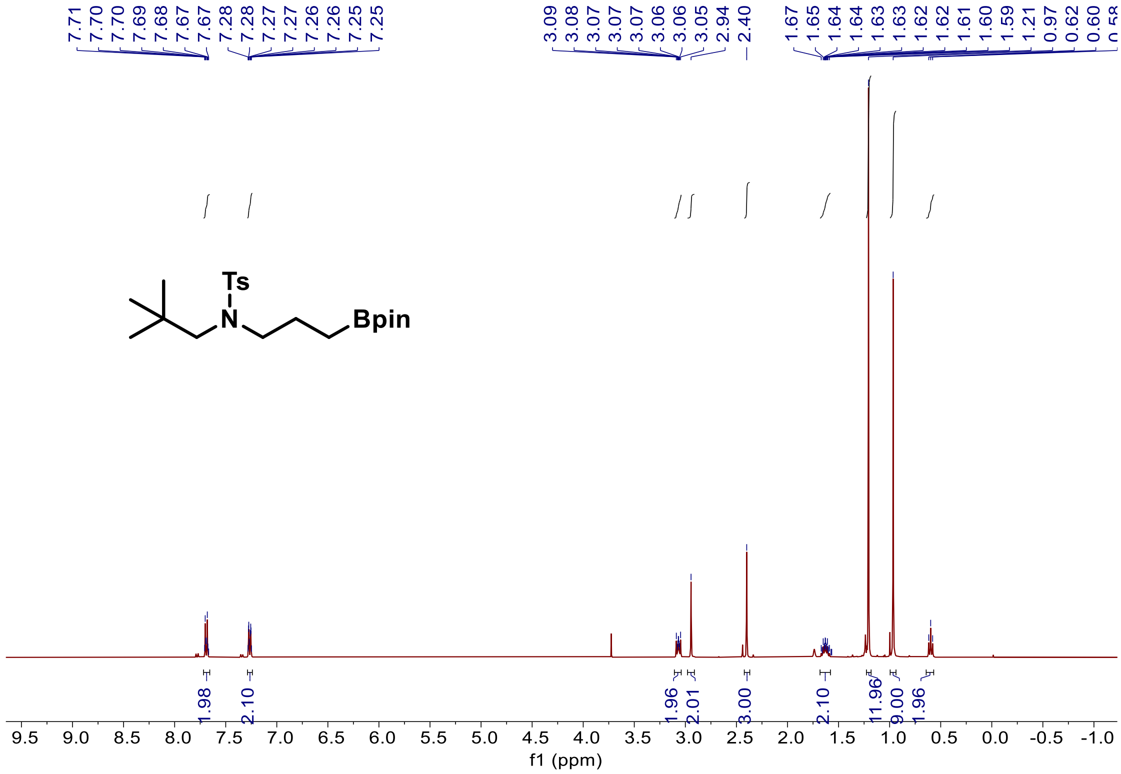

Supplementary Figure 238. ^1H NMR spectrum of **3b** (400 MHz, Chloroform-*d*)

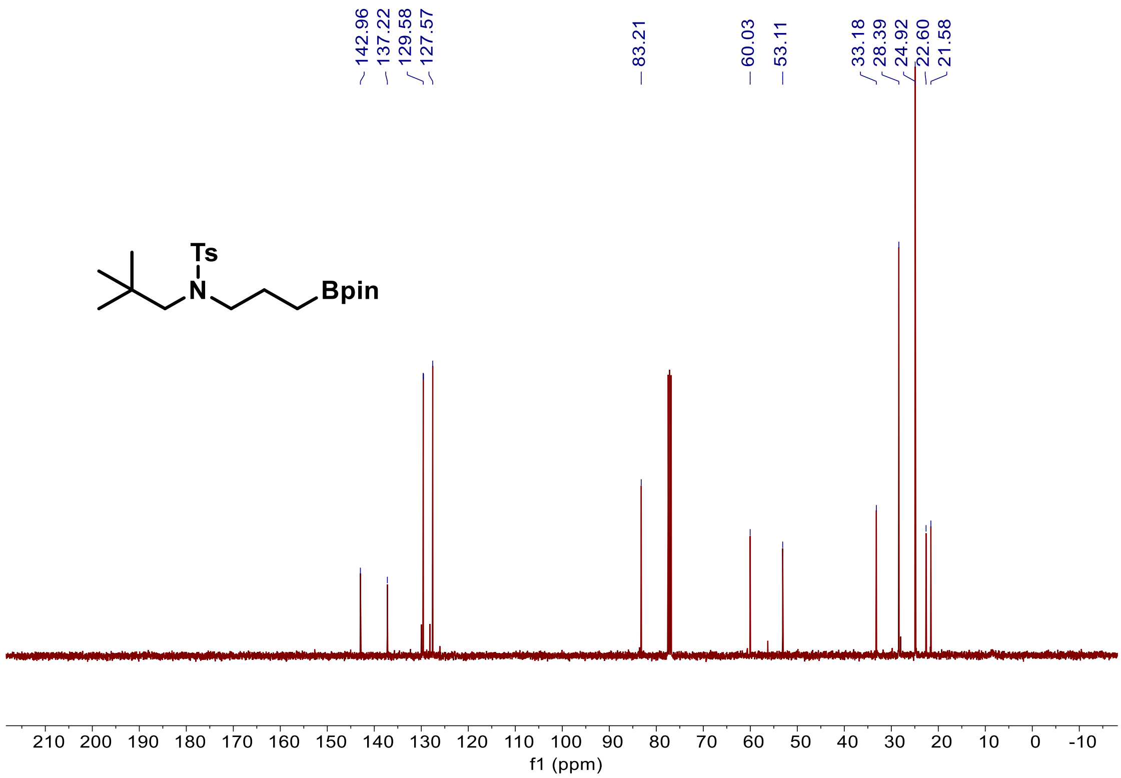

Supplementary Figure 239. $^{13}C\{^{1}H\}$ NMR spectrum of **3b** (101 MHz, Chloroform-*d*)

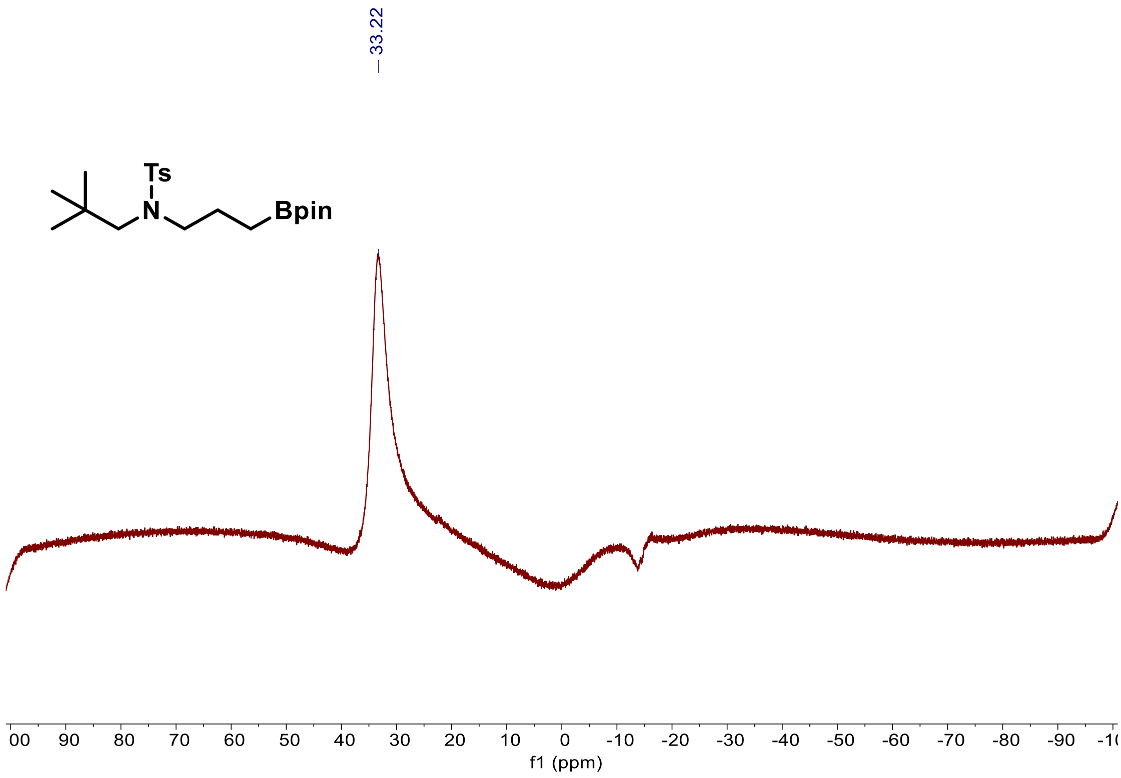

Supplementary Figure 240. ^{11}B NMR spectrum of **3b** (128 MHz, Chloroform-*d*)

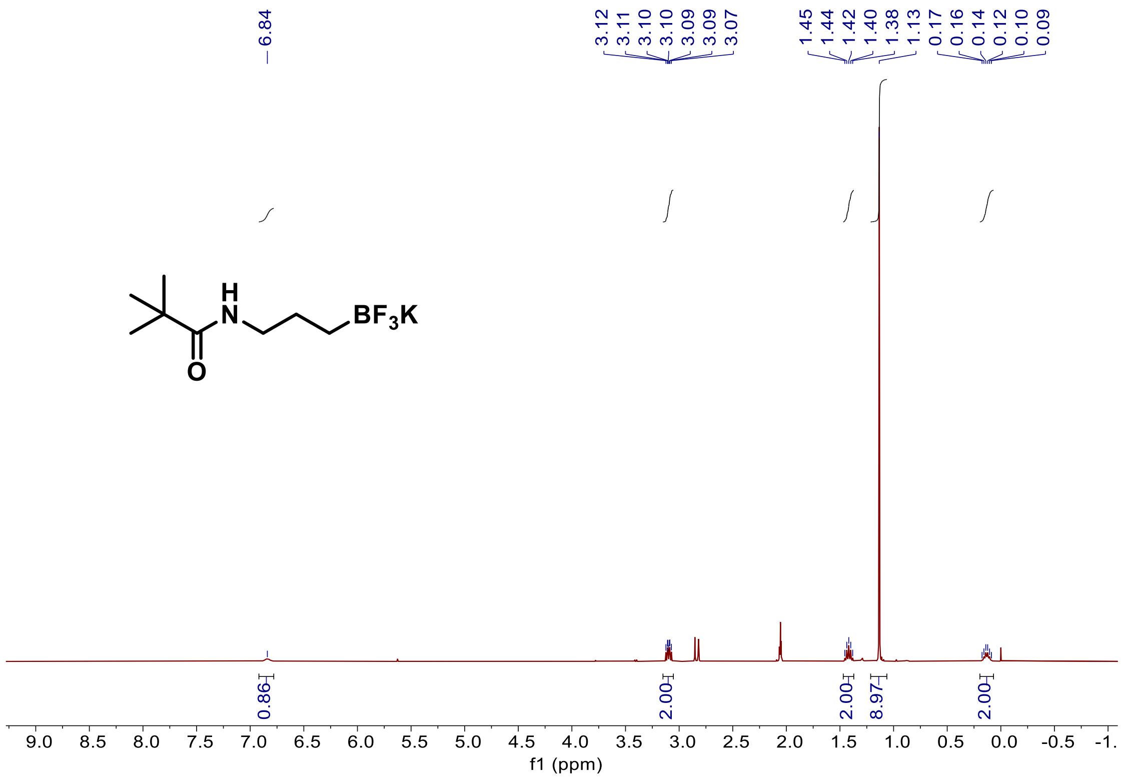

Supplementary Figure 241. ^{1}H NMR spectrum of **3c** (400 MHz, Acetone-*d6*)

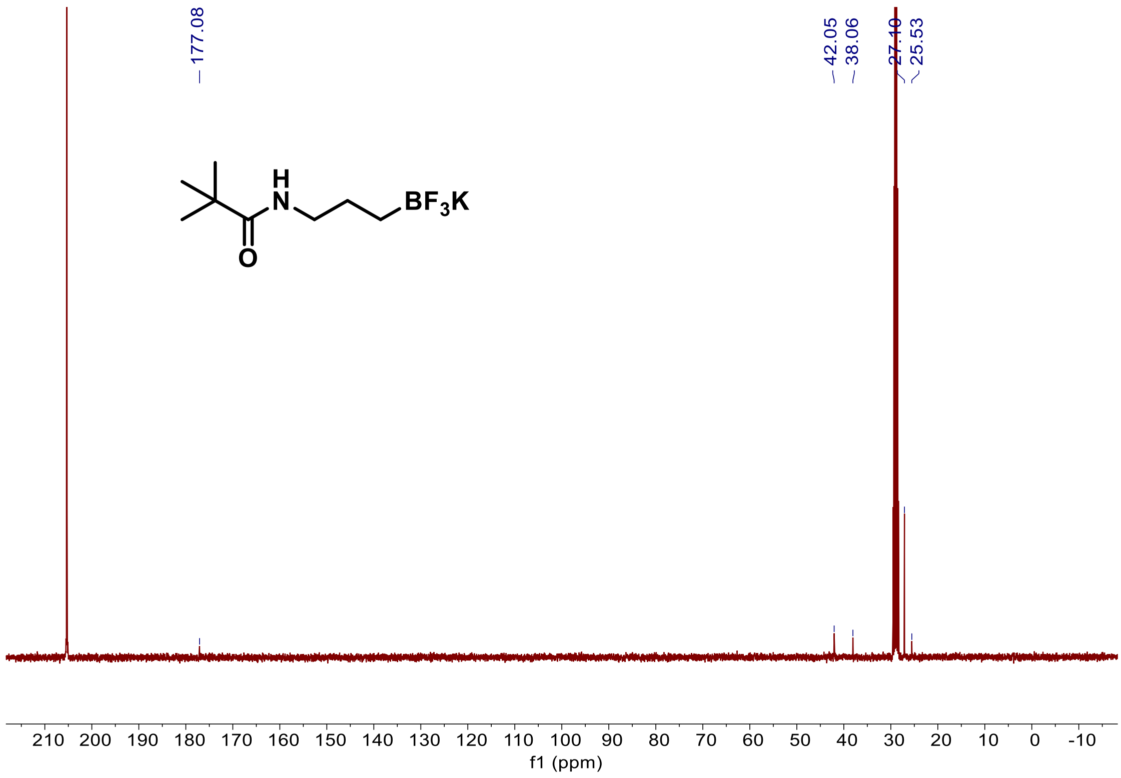

Supplementary Figure 242. $^{13}C\{^{1}H\}$ NMR spectrum of **3c** (101 MHz, Acetone-*d6*)

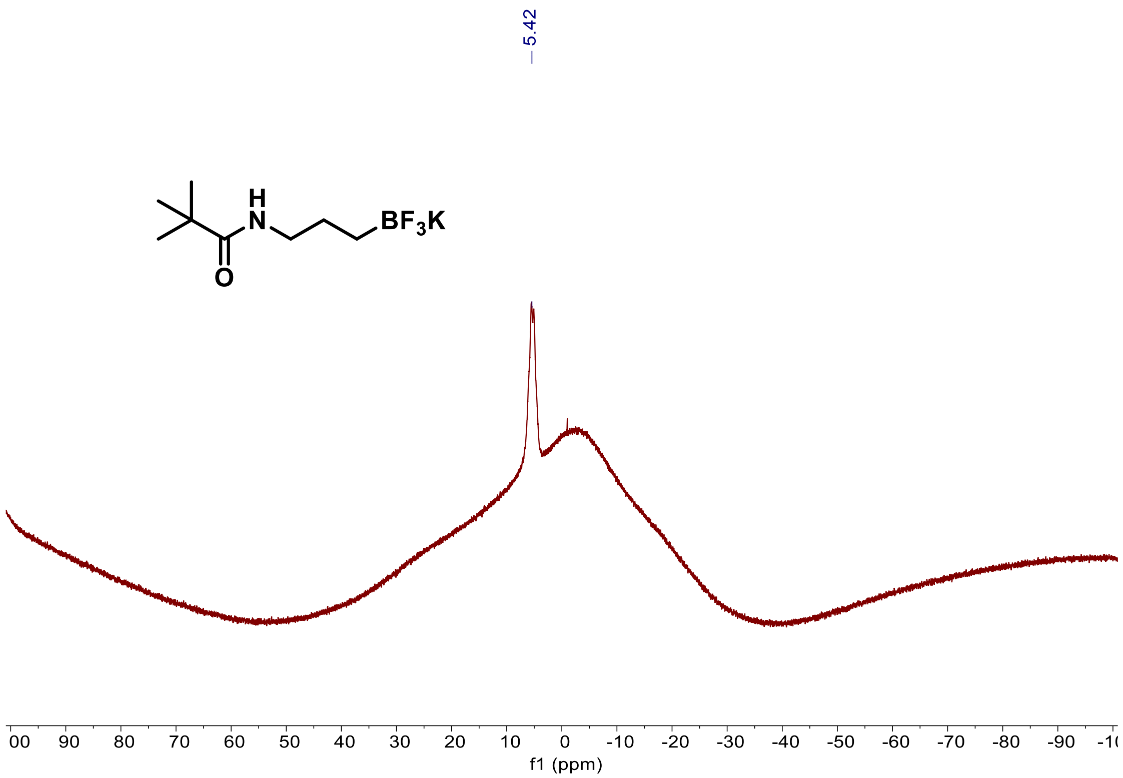

Supplementary Figure 243. ^{11}B NMR spectrum of **3c** (128 MHz, Acetone-*d6*)

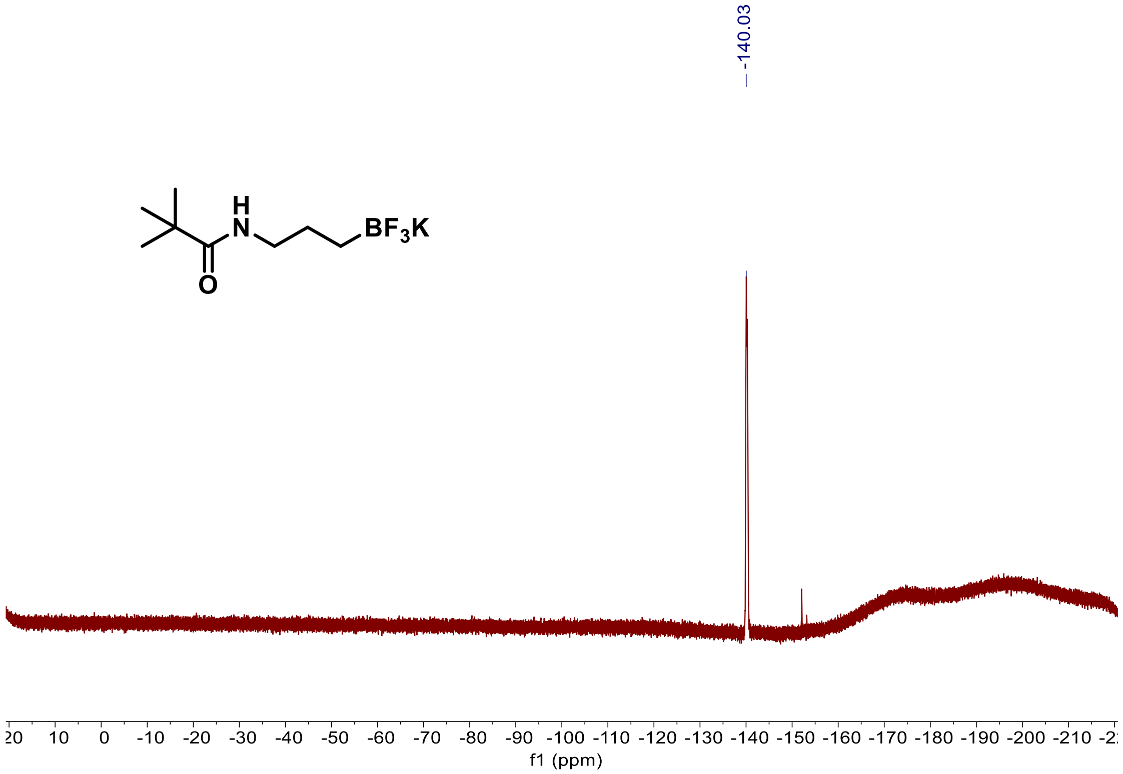

Supplementary Figure 244. ^{19}F NMR spectrum of **3c** (376 MHz, Acetone-*d6*)

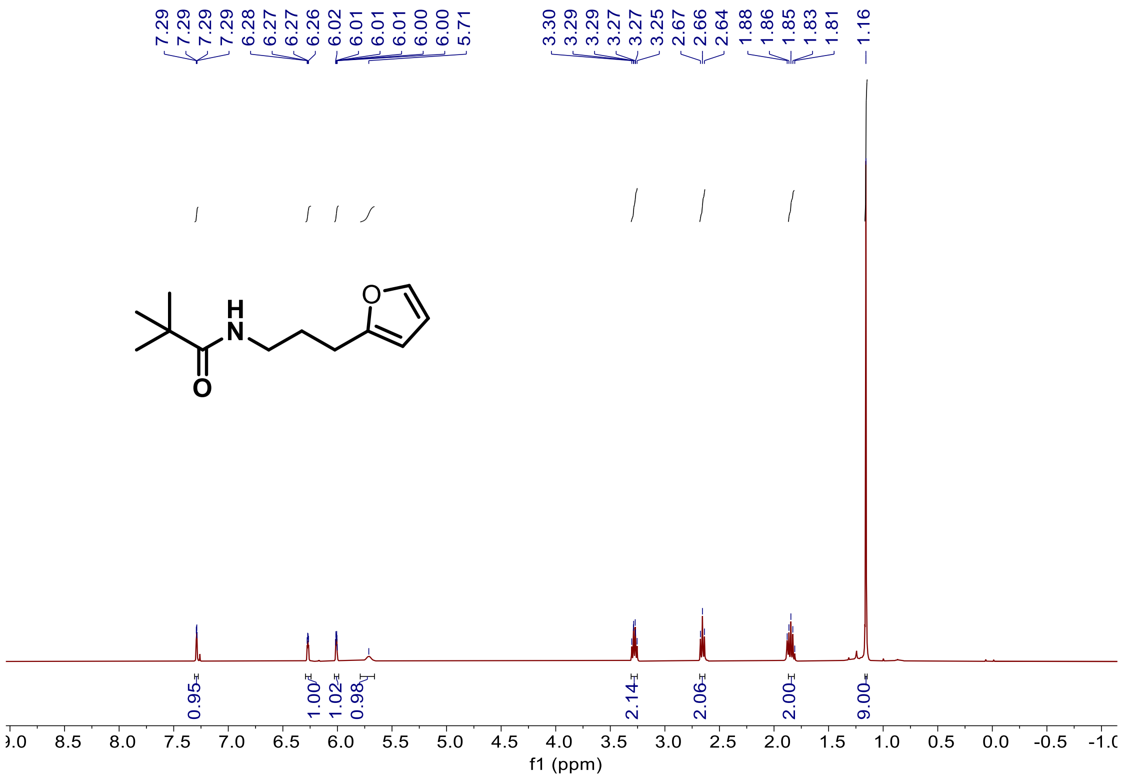

Supplementary Figure 245. ^{1}H NMR spectrum of **3d** (400 MHz, Chloroform-*d*)

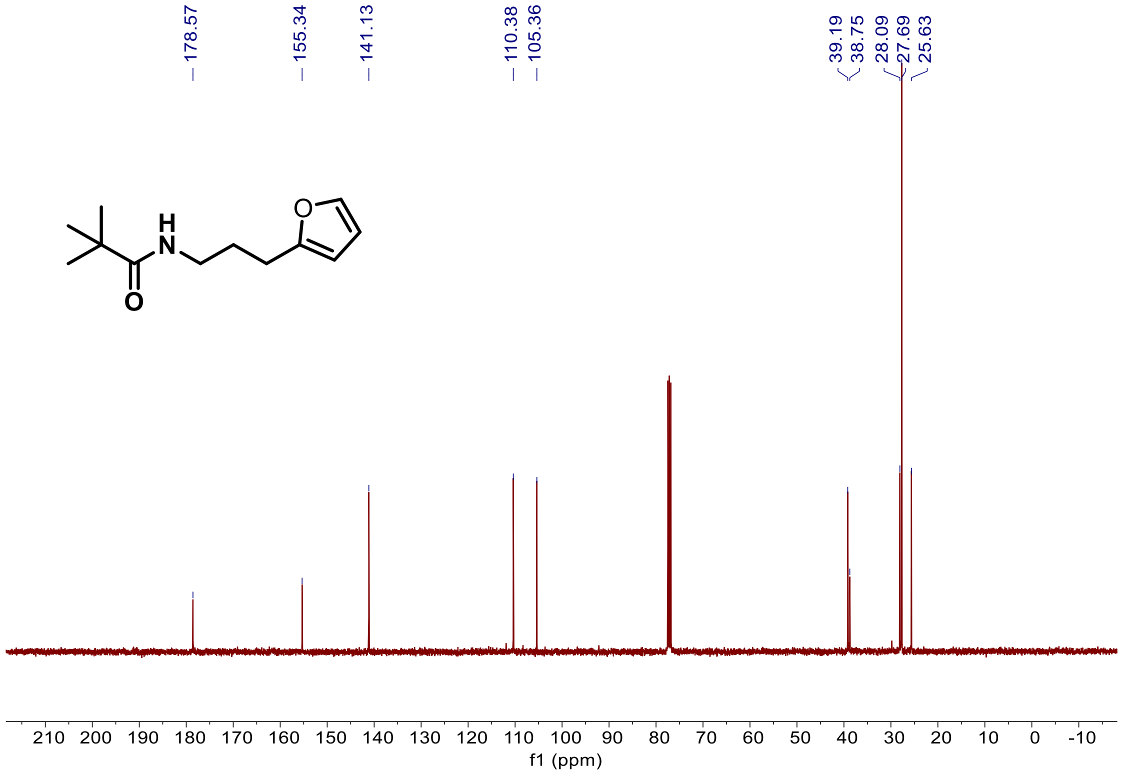

Supplementary Figure 246. ^{13}C{^{1}H} NMR spectrum of **3d** (101 MHz, Chloroform-*d*)

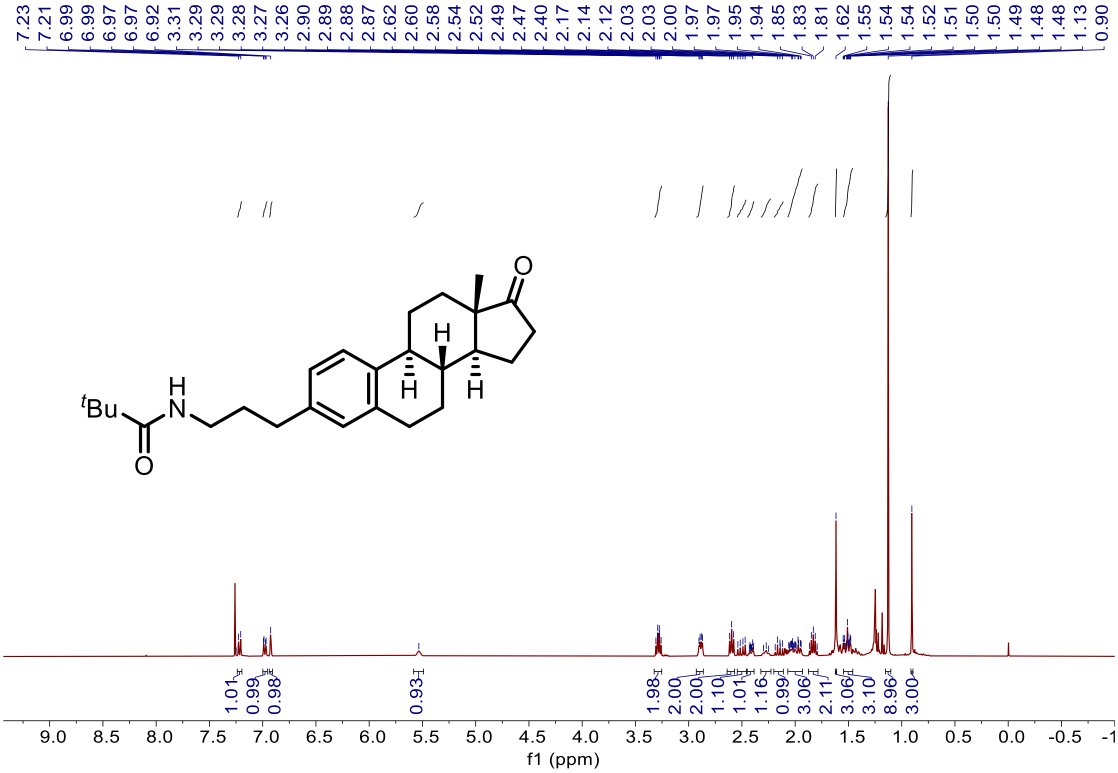

Supplementary Figure 247. ^{1}H NMR spectrum of **3e** (400 MHz, Chloroform-*d*)

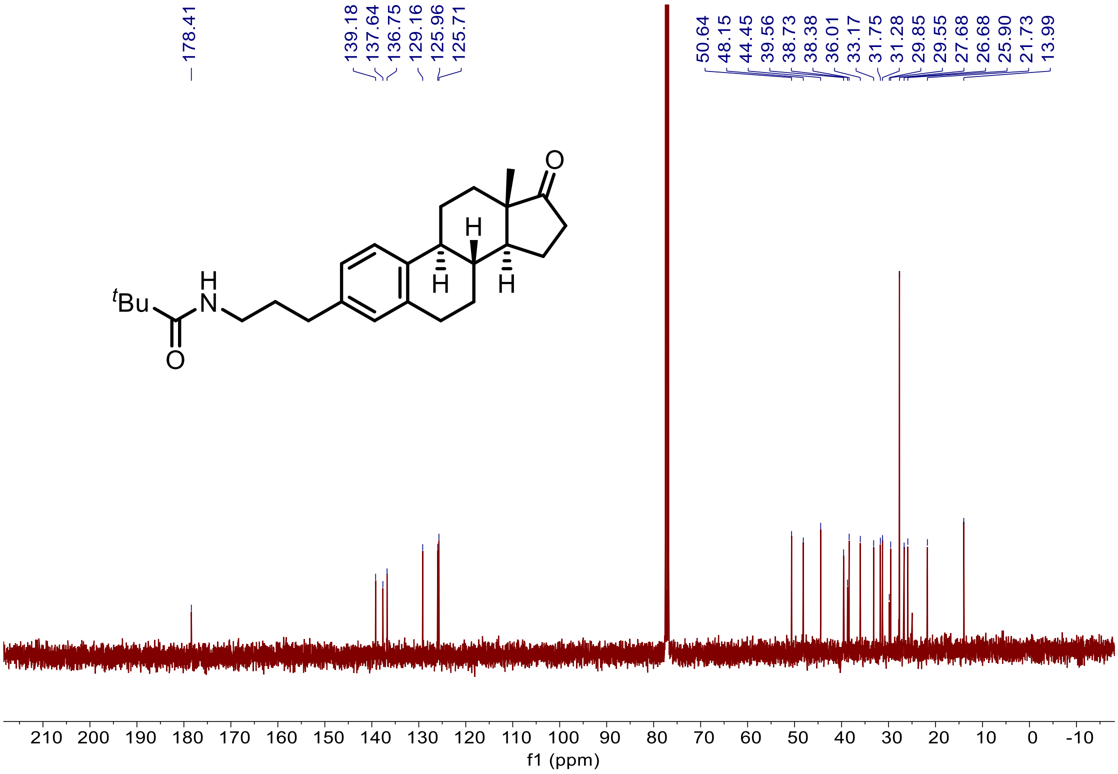

Supplementary Figure 248. $^{13}C\{^{1}H\}$ NMR spectrum of **3e** (101 MHz, Chloroform-*d*)

References

1 Bertus, P. & Szymoniak, J. A Direct Synthesis of 1-Aryl- and 1-Alkenylcyclopropylamines from Aryl and Alkenyl Nitriles. *J. Org. Chem.* **68**, 7133-7136 (2003).

2 Miyamura, S., Araki, M., Suzuki, T., Yamaguchi, J. & Itami, K. Stereodivergent Synthesis of Arylcyclopropylamines by Sequential C-H Borylation and Suzuki–Miyaura Coupling. *Angew. Chem. Int. Ed.* **54**, 846-851 (2015).

3 Kondo, H. *et al.* σ-Bond Hydroboration of Cyclopropanes. *J. Am. Chem. Soc.* **142**, 11306-11313 (2020).

4 Wang, Y. *et al.* Rhodium-catalysed selective C–C bond activation and borylation of cyclopropanes. *Chem. Sci.* **12**, 3599-3607 (2021).

5 Liu, X. *et al.* Aminoazanium of DABCO: An Amination Reagent for Alkyl and Aryl Pinacol Boronates. *Angew. Chem. Int. Ed.* **59**, 2745-2749 (2020).

6 Zhao, H., Gao, Q., Zhang, Y., Zhang, P. & Xu, S. Iridium-Catalyzed γ-Selective Hydroboration of γ-Substituted Allylic Amides. *Org. Lett.* **22**, 2861-2866 (2020).

7 Bohre, A., Saha, B. & Abu-Omar, M. M. Catalytic Upgrading of 5-Hydroxymethylfurfural to Drop-in Biofuels by Solid Base and Bifunctional Metal–Acid Catalysts. *ChemSusChem* **8**, 4022-4029 (2015).

8 Li, F. *et al.* Highly Efficient Transfer Hydrogenation of Levulinate Esters to γ-Valerolactone over Basic Zirconium Carbonate. *Ind. Eng. Chem. Res.* **57**, 10126-10136 (2018).

9 Wang, T. *et al.* Ligand cooperativity enables highly enantioselective C–C σ-bond hydroboration of cyclopropanes. *Chem* **9**, 130-142, (2023).

10 Kondo, H., Itami, K. & Yamaguchi, J. Rh-catalyzed regiodivergent hydrosilylation of acyl aminocyclopropanes controlled by monophosphine ligands. *Chem. Sci.* **8**, 3799-3803 (2017).
